# Sex- and age-specific reference intervals for diagnostic ratios reflecting relative activity of steroidogenic enzymes and pathways in adults

**DOI:** 10.1371/journal.pone.0253975

**Published:** 2021-07-08

**Authors:** Valentin Rousson, Daniel Ackermann, Belen Ponte, Menno Pruijm, Idris Guessous, Claudia H. d’Uscio, Georg Ehret, Geneviève Escher, Antoinette Pechère-Bertschi, Michael Groessl, Pierre-Yves Martin, Michel Burnier, Bernhard Dick, Murielle Bochud, Bruno Vogt, Nasser A. Dhayat

**Affiliations:** 1 Department of Epidemiology and Health Systems, Unisanté, Lausanne, Switzerland; 2 Department of Nephrology and Hypertension and Department of Clinical Research, Inselspital, Bern University Hospital, University of Bern, Bern, Switzerland; 3 Department of Specialties of Internal Medicine, Nephrology Service, University Hospital of Geneva, Geneva, Switzerland; 4 Nephrology Service, University Hospital of Lausanne, Lausanne, Switzerland; 5 Department of Community Medicine, Primary Care and Emergency Medicine, University Hospital of Geneva, Geneva, Switzerland; 6 Department of Specialties of Internal Medicine, Cardiology Service, University Hospital of Geneva, Geneva, Switzerland; 7 Department of Internal Medicine Specialties, Endocrinology Service, University Hospital of Geneva, Geneva, Switzerland; National Institute of Child Health and Human Development (NICHD), NIH, UNITED STATES

## Abstract

**Objective:**

Diagnostic ratios calculated from urinary steroid hormone metabolites are used as a measure for the relative activity of steroidogenic enzymes or pathways in the clinical investigation of steroid metabolism disorders. However, population-based sex- and age-specific reference intervals and day-night differences in adults are lacking.

**Methods:**

Sixty-five diagnostic ratios were calculated from steroid metabolites measured by GC-MS in day- and night-time and in 24-hour urine from 1128 adults recruited within the Swiss Kidney Project on Genes in Hypertension (SKIPOGH), a population-based, multicenter cohort study. Differences related to sex, age and day- and night-time were evaluated and reference curves in function of age and sex were modelled by multivariable linear mixed regression for diagnostic ratios and were compared to values from the literature.

**Results:**

Most ratios had sex- and age-specific relationships. For each ratio, percentiles were plotted in function of age and sex in order to create reference curves and sex- and age-specific reference intervals derived from 2.5^th^ and 97.5^th^ percentiles were obtained. Most ratios reflected a higher enzyme activity during the day compared to the night.

**Conclusions:**

Sex- and age-specific references for 24 hours, day and night urine steroid metabolite ratios may help distinguishing between health and disease when investigating human disorders affecting steroid synthesis and metabolism. The day-night differences observed for most of the diagnostic ratios suggest a circadian rhythm for enzymes involved in human steroid hormones metabolism.

## Introduction

A urinary steroid hormone profile provides a comprehensive overview about predominantly excreted precursors and products of progestins, androgens, estrogens, mineralocorticoids, and glucocorticoids in urine. Thanks to its growing importance in research and advances in analytical methods, urinary steroid hormone profiling becomes increasingly recognized and available for a wide range of clinical diagnoses [1–8].

**Fig 1:**
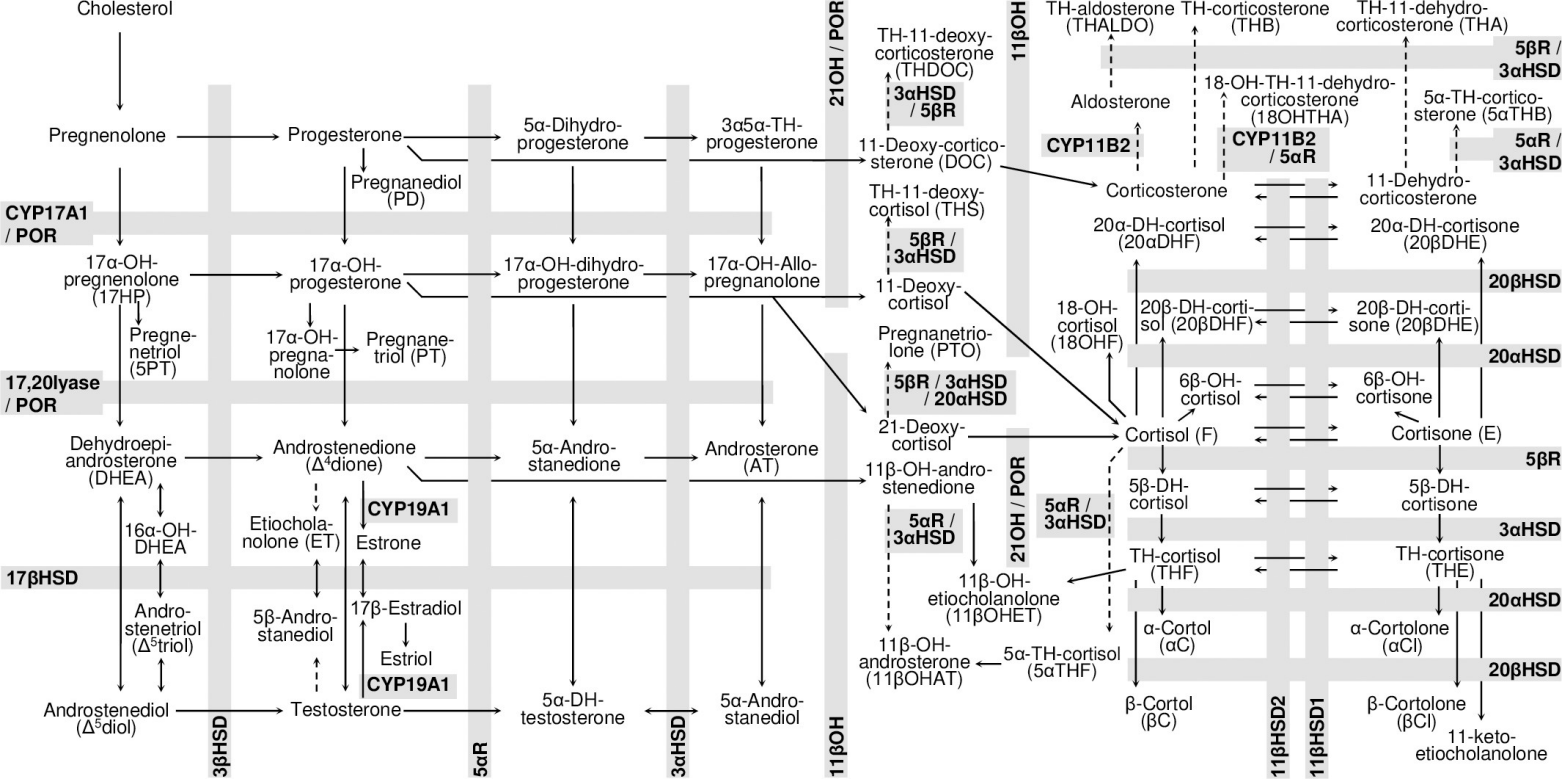
Enzymes involved in human steroid hormone biosynthesis. Steroidogenic enzymes from multiple endocrine cell types are combined in this overview. A small number of different enzyme activities, highlighted by gray bars, is involved in the metabolism of a large number of steroid hormone metabolites. The metabolism of one steroid hormone to another is indicated by an arrow and the enzyme activity involved in this metabolism is marked by the corresponding gray background of the arrow together with the enzyme name indicated. Solid arrows indicate direct metabolites while dotted arrows indicate indirect metabolites. Abbreviations: 21OH: 21-hydroxylase; 3βHSD: 3β-hydroxysteroid dehydrogenase; 11βOH: 11β-hydroxylase deficiency; CYP17A1: Cytochrome P450 17A1, POR: P450 oxidoreductase; 17βHSD: 17β-hydroxysteroid dehydrogenase; 5αR: 5α-reductase; 5βR: 5β-reductase; CYP19A1: Cytochrome P450 19A1/Aromatase; 11βHSD2: 11β-hydroxysteroid dehydrogenase type 2; 11βHSD1: 11β-hydroxysteroid dehydrogenase type 1; 20αHSD: 20α-hydroxysteroid dehydrogenase; 20βHSD: 20β-hydroxysteroid dehydrogenase; 3αHSD: 3α-hydroxysteroid dehydrogenase; 3βHSD: 3β-hydroxysteroid dehydrogenase; CYP11B2: aldosterone synthase; DH: dihydro; TH: tetrahydro.

The analysis of single steroid compounds or classes represents one component of the interpretation of urinary steroid hormone profiles and is commonly extended to the analysis of ratios of two or more steroid hormone metabolites. Ratios used in clinical practice and research often are calculated by dividing a steroid precursor by its product, yielding a measure of the relative activity of a steroidogenic enzyme or pathway. These ratios represent indirect activity markers in the sense that a high ratio indicates a low enzymatic activity and *vice versa*. Ratios can be diagnostically more indicative than a single steroid metabolite and some ratios have taken on a firmly established role as so-called diagnostic ratios in the diagnostic of a disease or condition. **Table 1** gives an overview about diagnostic ratios without this list being exhaustive. **Fig 1** shows pathways of steroid hormone biosynthesis and important steroidogenic enzymes reflected by diagnostic ratios in **Table 1**.

**Table 1:** Diagnostic ratios of urinary steroid hormone metabolites.

Enzymes, pathways and disorders	Ratios	Ratio ID	References
21-hydroxylase deficiency (21OHD)	PTO/THE	1	[9]
	PTO/(THE+THF+5αTHF)	2	[9–13]
	17HP/THE	3	[9]
	17HP/(THE+THF+5αTHF)	4	[9,10]
	PT/THE	5	[9]
	PT/(THE+THF+5αTHF)	6	[9,10,13]
	(PTO+17HP+PT)/THE	7	[9]
	(PTO+17HP+PT)/(THE+THF+5αTHF)	8	[9]
3β-hydroxysteroid dehydrogenase deficiency (3βHSDD)	5PT/THE	9	[9]
	5PT/(THE+THF+5αTHF)	10	[9–12]
	DHEA/THE	11	[9]
	DHEA/(THE+THF+5αTHF)	12	[9–13]
	(DHEA+16OHDHEA)/THE	13	[9]
	(DHEA+16OHDHEA)/(THE+THF+5αTHF)	14	[9]
Ratio to distinguish 3β-HSDD from 21OHD	5PT/PTO	15	[11,12]
11β-hydroxylase deficiency (11βOHD)	THS/THE	16	[9]
	THS/(THE+THF+5αTHF)	17	[9,10,13]
CYP17A1 global deficiency (CYP17A1GD)^a^	PD/(AT+ET)	18	[9]
	(THA+THB+5αTHB)/(AT+ET)	19	[9,10,13]
17α-hydroxylase global deficiency (17αOHGD)	(THA+THB+5αTHB)/THE	20	[9]
	(THA+THB+5αTHB)/(THE+THF+5αTHF)	21	[9–13]
17α-hydroxylase Δ^4^-pathway deficiency (17αOHΔ4D)	PD/17HP	22	[9]
	PD/PT	23	[9,10]
	PD/(PT+17HP)	24	[9]
17,20-lyase global deficiency (17,20LGD)	(AT+ET)/THE	25	[9]
	(AT+ET)/(THE+THF+5αTHF)	26^b^	[9,10,13]
17,20-lyase Δ^5^-pathway deficiency (17,20LΔ5D)	5PT/(DHEA+16OHDHEA)	27	[9]
	5PT/Δ^5^diol	28	[9]
	5PT/Δ^5^triol	29	
	5PT/(DHEA+16OHDHEA+Δ^5^diol+Δ^5^triol)	30	
17,20-lyase Δ^4^-pathway deficiency (17,20LΔ4D)	17HP/11βOHAT	31	[9]
	PT/11βOHAT	32	[9]
	(17HP+PT)/11βOHAT	33	[9]
	17HP/(AT+ET)	34	[9]
	PT/(AT+ET)	35	[9]
	(17HP+PT)/(AT+ET)	36	[9]
CYP17A1 global Δ^4^- vs. Δ^5^-pathway (CYP17A1Δ4vs.Δ5)	11βOHAT/(DHEA+16OHDHEA)	37	[9]
	11βOHAT/Δ^5^diol	38	[9]
	11βOHAT/(DHEA+16OHDHEA+Δ^5^diol)	39	
	11βOHAT/(DHEA+16OHDHEA+Δ^5^diol+Δ^5^triol)	40	
P450 oxidoreductase deficiency (PORD)	(17HP+PT)/THE	41	[9]
	(17HP+PT)/(THE+THF+5αTHF)	42	[9,11,12]
	PD/THE	43	[9]
	PD/(THE+THF+5αTHF)	44	[9,13]
17β-hydroxysteroid dehydrogenase (17βHSD)	(AT+ET)/(THE+THF+5αTHF)	45	[9,10]
Alternative androgen backdoor pathway vs. classic pathway (ABPvs.CP)	AT/ET	46	[9]
5α-reductase deficiency (5αRD)	ET/AT	47	[9–15]
	11βOHET/11βOHAT	48	[9–12]
	THF/5αTHF	49	[9–18]
	THB/5αTHB	50	[9–13]
CYP19A1/Aromatase deficiency (CYP19A1D)	testosterone/17β-estradiol	51	[9]
11β-hydroxysteroid dehydrogenase type 2 deficiency (11βHSD2D)^c^	F/E	52	[9,11,12,15,19,20]
	(THF+5αTHF)/THE	53	[9–13,15,17–21]
	(αC+βC)/(αCl+βCl)	54	[9]
	(F+E)/(THF+5αTHF+THE)	55	[9]
11β-hydroxysteroid dehydrogenase type 1 deficiency (11βHSD1D)^d^	THE/(THF+5αTHF)	56	[9–12,14,16]
	(αCl+βCl)/(αC+βC)	57	[9,11,12,16]
20α-hydroxysteroid dehydrogenase (20αHSD)	(THF+5αTHF+THE)/(αC+αCl)	58	[9]
20β-hydroxysteroid dehydrogenase (20βHSD)	(THF+5αTHF+THE)/βC+βCl	59	[9]
20αHSD vs. 20βHSD	(αC+αCl)/(βC+βCl)	60	[9]
3α-hydroxysteroid dehydrogenase (3αHSD)	20αDHF/(THF+5αTHF)	61	[9]
Polycystic ovary syndrome (PCOS)	(androstanediol^1.5^×20βDHE)/(20βDHE+[F×log(estriol)]	62	[9]
Glucocorticoid-remediable aldosteronism (GRA)^e^	F/18OHF	63	[12,13]
Hyper-/Pseudohypo-aldosteronism	THALDO×100/(THE+THF+5αTHF)	64	[10–12]
Hypoaldosteronism	18OHTHA/THALDO	65	[10]

^a^combined activity of 17α-hydroxylase and 17,20-lyase

^b^ratio 26 also used to assess 3βHSDD

^c^/apparent mineralocorticoid excess

^d^/cortisone reductase deficiency

^e^/familial hyperaldosteronism type I.

Abbreviations: Δ^5^diol, androstenediol; Δ^5^triol, androstenetriol, AT, androsterone; 11βOHAT, 11β-OH-androsterone; αC, α-cortol; βC, β-cortol; αCl, α-cortolone; βCl, β-cortolone; DHEA, dehydroepiandrosterone; 16OHDHEA, 16α-OH-dehydroepiandrosterone; E, cortisone; 20βDHE, 20β-DH-cortisone; F, cortisol; ET, etiocholanolone; 11βOHET, 11β-OH-etiocholanolone; 17HP, 17α-OH-pregnanolone; PD, pregnanediol; PT, pregnanetriol; 5PT, pregnenetriol; PTO, pregnanetriolone; THA, tetrahydro-11-dehydro-corticosterone; 18OHTHA, 18-OH-tetrahydro-11-dehydrocorticosterone; THB, tetrahydrocorticosterone; 5αTHB, 5α-tetrahydrocorticosterone; THE, tetrahydrocortisone; THF, tetrahydrocortisol; 5αTHF, 5α-tetrahydrocortisol; 18OHF, 18-OH-cortisol; THS, TH-11-deoxycortisol; THALDO, tetrahydroaldosterone.

Ratios are dimensionless and are commonly used to describe relative enzyme activities of steroidogenic pathways particularly in steroid hormone disorders. For this purpose, ratios of affected patients are compared with those not affected. Some ratios are not only used for one, but for different disorders, e.g. ratios with the ID numbers 26 and 45 are identical. References for ratios are indicated if available.

Based on 24-hour urinary steroid hormone profiles, we previously introduced population-based sex- and age-specific reference intervals for steroid hormone excretion values [22]. However, the availability of comparable reference intervals for diagnostic ratios largely remains an unmet need and the present work is suitable to fill this gap.

## Subjects and methods

The study was approved by the competent institutional ethics committees in Bern, Geneva, and Lausanne. In particular, these are in Bern the Kantonale Ethikkommission Bern, in Geneva the Commission Cantonale d’Ethique de la Recherche sur l’être humain (CCER), and in Lausanne the Commission cantonale d’éthique de la recherche sur l’être humain (CER-VD).The study adhered to the Declaration of Helsinki, and all participants provided written informed consent.

Detailed information about the study population, study visits, urine collection procedures, the quantification of steroid compounds by GC-MS in urine, quality controls of the GC-MS method, and inclusion and exclusion criteria of the reference sample group was previously published [22]. In brief, participants were recruited among the general Swiss population within the multicenter, family-based Swiss Kidney Project on Genes in Hypertension (SKIPOGH) cohort. Inclusion criteria were 1) an age ≥18 years, 2) European ancestry and 3) at least one and ideally three first-degree family members willing to participate as previously stated [22]. SKIPOGH recruited 1128 participants in the regions of Bern and Geneva and in the city of Lausanne.

Diabetes was defined as fasting glycemia ≥7 mmol/L or as reported or treated. Hypertension was defined as systolic blood pressure ≥140 mmHg or diastolic blood pressure ≥90 mmHg or as treated.

Day- and night-time urine samples were collected within 24 hours and urine aliquots were immediately stored at -80°C. Centralized urinary steroid hormone profiling was conducted by GC-MS by an established method in the steroid laboratory of the Department of Nephrology and Hypertension at the Bern University Hospital, Switzerland [22,23]. Participants with incorrect urine collections, missing urinary steroid hormone measurements, and conditions interfering with steroid hormone metabolism were excluded from the generation of reference intervals thereby reducing the reference sample group to 838 participants [22]. Participants were excluded due to pregnancy, self-reported bilateral oophorectomy or bilateral oophorectomy with hysterectomy, Addison’s disease, active malignant disease, liver disease or more than threefold elevated liver enzymes, estimated glomerular filtration rate less than 30 mL/min per 1.73 m^2^ calculated by CKD-EPI formula, missing data for serum creatinine or urinary creatinine, body mass index <16 kg/m^2^ or > 40 kg/m^2^, and intake of drugs able to alterate steroid hormone metabolism as previously specified [22].

Urinary steroid hormone metabolites measured in μg/day, μg/night and μg/24 hours were converted into nmol/day, nmol/night and nmol/24 hours using previously published conversion factors [22]) Diagnostic ratios linked to steroidogenic enzymes, pathways and disorders listed in **Table 1** were calculated. References obtained from a literature search for values on diagnostic ratios based on urinary steroid hormone metabolites published since 1986 were added to **Table 1** and published values were listed in **S1 Table**.

After a thorough exploration of outliers, only 53 values, out of 46’974 available observations, were detected as outliers and removed from subsequent analysis as described in **S1 Text**. Sex specific differences in diagnostic ratios based on steroid hormone metabolites measured in 24-hour urine were assessed by Mann-Whitney U test. Within-sex differences for diagnostic ratios from day- and night-time were assessed by Wilcoxon signed-rank test. Age- and sex-related changes of 24-hour diagnostic ratios were separately modelled for men and women by linear mixed regression models taking family and center effect into account [24]. Different percentiles were plotted from the best model selected. The model creation and selection process is described in detail in **S1 Text**. Sex- and age-specific reference intervals based on the 2.5^th^-97.5^th^ percentiles for each diagnostic ratio were estimated from the described statistical models for different ages. All statistical analyses were conducted using the R software, version 3.5.0 [25].

## Results

### Characteristics of the reference population

The reference sample group included 838 participants across a wide age range from 18.1–90.0 years (median age 49.6 years) among them 459 (54.8%) men. Study participants included smokers (n = 201, 24.0%) and a number of them were suffering from hypertension (n = 201, 24.0%) and diabetes (n = 39, 3.5%). Characteristics and reference intervals for quantitative urinary steroid hormone excretion values of the reference sample group have previously been described in detail [22].

### Descriptive statistics and day- and night-time correlations of diagnostics ratios

Diagnostic ratios described in **Table 1** and **Fig 1** were calculated for different urine sampling periods covering day- and night-time and 24-hour. Most diagnostic ratios showed a very distinct right-skewed distribution for all three sampling periods with the exception of ratio 55 (11βHSD2D) showing a slightly left-skewed distribution in women and an almost normal distribution in men (**S1 Fig** with outliers and **S2 Fig** without outliers). For all sampling periods a bimodal sex-related distribution was found for ratios representing 5α-reductase and for ratios including androgens, estrogens, 17α-OH-pregnanolone (17HP), pregnanediol (PD), TH-11-deoxycortisol (THS), α-cortol (αC), or α-cortolone (αCl). No major differences in the distributions of ratios were found across the study sites Bern, Geneva and Lausanne, or between day- and night-time or 24-hour sampling periods (**S1** and **S2 Figs**). Center accounted on average for only 1% of the variance of the ratios (**S1 Text**). Near normality of ratios calculated for all urine sampling periods was achieved by appropriate transformation [**S3(A)–S3(F) Fig**]. Transformed ratios at day- and night-time strongly positively correlated with each other yielding values for Spearman’s ρ from 0.724–0.969 in women and from 0.673–0.963 in men across all age groups [**S3(G)** and **S3(H) Fig**]. Highest correlations between day- and night-time diagnostic ratios in women and men were found for the ratios androsterone/etiocholanolone (ratio 46) and etiocholanolone/androsterone (ratio 47) representing the alternative androgen backdoor pathway vs. the classic pathway and 5α-reductase, respectively. Lowest correlations between day- and night-time ratios in women and men were found for the ratio 20αDHF/(THF+5αTHF) (ratio 61) representing 3α-hydroxysteroid dehydrogenase activity.

### Differences related to sex, age and day- and night-time

Inter-sex comparison of diagnostic ratios from 24-hour urine [**S2 Table** and **S3(A) Fig**] revealed sex-specific differences for almost all ratios, except for ratios reflecting global activity of the 17α-hydroxylase (ratio 20 und 21) and Δ^5^-pathway activity of the 17,20-lyase (ratios 27, 28, and 30). Diagnostic ratios significantly differed between day- and night-time collections for 60 out of 65 diagnostic ratios in women and for 55 out of 64 diagnostic ratios in men (**S3 Table**). Higher enzymatic activities at day-time compared to night-time were found for 21-hydroxylase, 3β-hydroxysteroid dehydrogenase, 11β-hydroxylase, 17α-hydroxylase, P450 oxidoreductase, 5α-reductase, 3β-hydroxysteroid dehydrogenase (in women), and CYP11B2. Lower enzymatic activities at day-time were found for 17,20-lyase, CYP17A1 of the Δ4-pathway vs. the Δ5-pathway, 20α-hydroxysteroid dehydrogenase (20αHSD) (in women), 20β-hydroxysteroid dehydrogenase (20βHSD), and 20αHSD versus 20βHSD. No difference between day- and night-time was found for aromatase activity.

### Reference intervals for diagnostics ratios

A nonparametric fit [**S3(B) Fig**] revealed sex- and age-specific relationships for most ratios. In order to create reference curves for each ratio from the selected models, percentiles were plotted as a function of age separately for women and men (**S4 Fig**). Sex- and age-specific reference intervals derived from 2.5^th^ and 97.5^th^ percentiles are shown for women in **Table 2** and men in **Table 3**. In addition, 1^st^-50^th^-99^th^ percentiles are listed for women in **S4 Table** and men in **S5 Table**. Patterns of reference curves for different ratios used to describe the same enzyme activities were not always consistent. In particular, higher values for ratios 1–8 representing 21-hydroxylase represent lower enzyme activity and were observed with increasing age in women and men for ratio 1 and ratio 2 whereas a higher activity was found with increasing age considering ratios 3–8. With regard to ratios 9–14 with lower values representing higher activity of 3β-hydroxysteroid dehydrogenase increasing enzymatic activity was found in the elderly. For ratio 15 used to distinguish 3β-hydroxysteroid dehydrogenase deficiency from 21-hydroxylase deficiency, reference curves showed an increased variation with advancing age. Ratios 16 and 17 reflecting 11β-hydroxylase showed similar sex-specific patterns. For ratios 18–40 reflecting single and combined 17α-hydroxylase and 17,20-lyase of the steroidogenic Δ^5^- and Δ^4^-pathways a variety of relationships with age was found. Ratio 18 reflecting global combined activities of these enzymes showed a higher variation in women at all ages compared to men. This also applied to ratios 22–24 reflecting the Δ^4^-pathway 17α-hydroxylase activity and to ratios 43 and 44 reflecting P450 oxidoreductase deficiency. The higher variation in women was driven by pregnanediol shared in the numerator of these ratios. Overall, the combined 17α-hydroxylase and 17,20-lyase activity (ratios 18 and 19) appeared to decrease with aging in both sexes and a higher activity within the 17α-hydroxylase Δ^4^-pathway was found in men compared to women across all age groups (ratios 22–24). Aging appeared to further increase global 17,20-lyase activity (ratios 25 and 26), but to have comparatively little impact on the 17,20-lyase Δ^5^-pathway (ratios 27–30). The association with ratios reflecting the 17,20-lyase Δ^4^-pathway varied depending on the denominator used, i. e. 11β-OH-androsterone in ratios 31–33 and androsterone+etiocholanolone in ratios 34–36. The Δ^5^-pathway of the combined 17α-hydroxylase and 17,20-lyase was found to be most active in youngest women and in men at their thirties whereas the corresponding Δ^4^-pathway appeared to be most active in the elderly in both sexes (ratios 37–40). From early adult life to old age the 17β-hydroxysteroid dehydrogenase activity (ratio 45) markedly decreased, and the activity of the alternative backdoor pathway towards androgen biosynthesis versus the classic pathway (ratio 46) as well as the 5α-reductase activity (ratios 47–50) slightly decreased. The aromatase activity (ratio 51) increased with aging in men, but not in women. A decreasing 11β-hydroxysteroid dehydrogenase type 2 activity (ratios 52–54) was found with increasing age and vice versa for 11β-hydroxysteroid dehydrogenase type 1 activity (ratios 56 and 57) in women and men. In women, similar patterns of 20α- and 20β-hydroxysteroid dehydrogenase activities (ratios 58 and 59) related to age were found. Accordingly, the ratio of both enzyme activities to each other (ratio 60) revealed no change with age in women. In men, a higher activity of 20α- versus 20β-hydroxysteroid dehydrogenase was found with increasing age. For 3α-hydroxysteroid dehydrogenase (ratio 61) no change with age was found in men and an increasing activity with aging in women. Ratio 62 used to predict the presence of polycystic ovary syndrome (PCOS) in women decreased with age. Ratio 63 used to diagnose glucocorticoid-remediable aldosteronism showed a positive association with age in men, but not in women. Ratio 64 used to diagnose hyper-/pseudohypo-aldosteronism showed no significant association with age in men and a negative association with age in women. Ratio 65 used to diagnose hypoaldosteronism showed no significant association with age in men and a positive association with age in women.

**Table 2:** Reference intervals for diagnostic ratios of 24-hour urine steroid hormone metabolites in women of different ages.

Women		Age, years						
Ratio ID	N	20	30	40	50	60	70	80
**1**	360	0.0026–0.0435	0.0023–0.0309	0.0022–0.0266	0.0022–0.0267	0.0023–0.0315	0.0026–0.0451	0.0031–0.0867
**2**	329	0.0014–0.0238	0.0012–0.0169	0.0012–0.0144	0.0012–0.0143	0.0012–0.0165	0.0014–0.0227	0.0016–0.0404
**3**	356	0.0106–0.200	0.0163–0.308	0.0152–0.286	0.0085–0.160	0.0046–0.0861	0.0037–0.0700	0.0037–0.0700
**4**	328	0.0059–0.137	0.0085–0.226	0.0078–0.200	0.0046–0.0961	0.0027–0.0467	0.0022–0.0371	0.0022–0.0371
**5**	348	0.0980–0.742	0.125–0.944	0.113–0.855	0.0728–0.552	0.0462–0.350	0.0397–0.300	0.0397–0.300
**6**	323	0.0547–0.414	0.0668–0.506	0.0594–0.450	0.0383–0.290	0.0244–0.185	0.0211–0.159	0.0211–0.159
**7**	344	0.124–0.897	0.158–1.14	0.144–1.04	0.0935–0.674	0.0596–0.430	0.0513–0.370	0.0513–0.370
**8**	322	0.0688–0.543	0.0837–0.692	0.0751–0.605	0.0501–0.369	0.0336–0.227	0.0296–0.194	0.0296–0.194
**9**	359	0.0167–0.441	0.0089–0.321	0.0048–0.239	0.0026–0.184	0.0015–0.147	0.0010–0.123	0.0007–0.107
**10**	328	0.0092–0.238	0.0047–0.169	0.0024–0.123	0.0013–0.0934	0.0007–0.0739	0.0004–0.0616	0.0003–0.0543
**11**	358	0.0099–1.03	0.0107–1.18	0.0090–0.900	0.0060–0.470	0.0038–0.235	0.0028–0.148	0.0023–0.114
**12**	329	0.0085–1.34	0.0058–0.729	0.0041–0.411	0.0029–0.239	0.0021–0.143	0.0015–0.0873	0.0011–0.0548
**13**	358	0.0366–1.63	0.0405–1.81	0.0306–1.37	0.0158–0.703	0.0083–0.370	0.0067–0.299	0.0067–0.299
**14**	329	0.0238–1.14	0.0209–1.01	0.0149–0.714	0.0085–0.408	0.0048–0.228	0.0032–0.152	0.0025–0.121
**15**	378	0.858–62.6	1.12–70.1	0.718–58.0	0.220–36.7	0.0802–26.1	0.0544–23.2	0.0544–23.2
**16**	360	0.0121–0.0579	0.0123–0.0598	0.0126–0.0617	0.0128–0.0637	0.0131–0.0658	0.0134–0.0680	0.0136–0.0703
**17**	329	0.0066–0.0326	0.0066–0.0326	0.0066–0.0326	0.0066–0.0326	0.0066–0.0326	0.0066–0.0326	0.0066–0.0326
**18**	327	0.0237–0.475	0.0376–1.14	0.0442–1.58	0.0373–1.12	0.0302–0.748	0.0309–0.778	0.0398–1.28
**19**	330	0.0340–0.284	0.0415–0.385	0.0510–0.534	0.0632–0.756	0.0791–1.10	0.100–1.65	0.128–2.56
**20**	360	0.0912–0.403	0.0980–0.425	0.0962–0.420	0.0861–0.386	0.0764–0.354	0.0733–0.343	0.0733–0.343
**21**	329	0.0596–0.221	0.0555–0.209	0.0516–0.197	0.0479–0.186	0.0444–0.176	0.0411–0.166	0.0380–0.157
**22**	372	1.10–14.9	1.19–16.2	1.23–16.7	1.22–16.5	1.14–15.5	1.03–13.9	0.879–11.9
**23**	357	0.217–2.59	0.282–4.61	0.301–5.37	0.261–3.86	0.219–2.63	0.207–2.34	0.207–2.34
**24**	353	0.189–1.87	0.248–3.30	0.268–3.88	0.233–2.88	0.196–2.01	0.185–1.80	0.185–1.80
**25**	320	0.673–5.88	0.601–5.37	0.459–4.33	0.296–3.06	0.183–2.09	0.125–1.56	0.0964–1.27
**26**	297	0.439–2.92	0.366–2.58	0.258–2.05	0.154–1.46	0.0921–1.06	0.0588–0.809	0.0408–0.654
**27**	376	0.0398–2.37	0.0280–1.88	0.0246–1.73	0.0274–1.85	0.0383–2.31	0.0476–2.67	0.0476–2.67
**28**	377	0.202–6.08	0.166–5.51	0.147–5.20	0.142–5.11	0.150–5.24	0.171–5.60	0.211–6.22
**29**	377	0.0229–1.87	0.0229–1.87	0.0229–1.87	0.0229–1.87	0.0229–1.87	0.0229–1.87	0.0229–1.87
**30**	375	0.0084–0.532	0.0097–0.555	0.0111–0.578	0.0126–0.602	0.0143–0.626	0.0161–0.651	0.0181–0.677
**31**	372	0.0414–0.996	0.0556–1.50	0.0502–1.30	0.0309–0.668	0.0190–0.347	0.0163–0.282	0.0163–0.282
**32**	357	0.437–3.04	0.438–3.04	0.381–2.65	0.288–2.00	0.196–1.36	0.149–1.04	0.132–0.920
**33**	353	0.491–4.01	0.513–4.24	0.456–3.67	0.347–2.62	0.237–1.65	0.184–1.22	0.168–1.09
**34**	328	0.0059–0.116	0.0088–0.198	0.0099–0.235	0.0085–0.190	0.0072–0.152	0.0079–0.172	0.0112–0.278
**35**	324	0.0638–0.373	0.0749–0.469	0.0827–0.541	0.0854–0.568	0.0844–0.558	0.0912–0.625	0.111–0.835
**36**	322	0.0719–0.454	0.0892–0.622	0.0990–0.726	0.0975–0.709	0.0954–0.687	0.104–0.781	0.127–1.05
**37**	374	0.0829–4.20	0.147–6.20	0.252–9.01	0.421–12.9	0.685–18.3	1.09–25.7	1.70–35.5
**38**	375	0.723–15.1	1.08–20.4	1.60–27.3	2.33–36.3	3.34–47.8	4.73–62.4	6.62–81.1
**39**	374	0.0860–2.95	0.145–4.27	0.240–6.12	0.385–8.65	0.607–12.1	0.937–16.7	1.42–22.8
**40**	373	0.0976–1.35	0.141–1.95	0.205–2.83	0.297–4.10	0.430–5.94	0.623–8.60	0.902–12.5
**41**	344	0.116–0.873	0.152–1.14	0.138–1.04	0.0877–0.661	0.0544–0.410	0.0463–0.349	0.0463–0.349
**42**	322	0.0639–0.529	0.0801–0.700	0.0721–0.614	0.0470–0.362	0.0308–0.216	0.0268–0.183	0.0268–0.183
**43**	358	0.0314–1.33	0.0519–2.78	0.0500–2.64	0.0284–1.15	0.0157–0.493	0.0130–0.378	0.0130–0.378
**44**	327	0.0153–0.725	0.0261–1.61	0.0256–1.56	0.0145–0.671	0.0080–0.283	0.0066–0.215	0.0066–0.215
**45**	297	0.439–2.92	0.366–2.58	0.258–2.05	0.154–1.46	0.0921–1.06	0.0588–0.809	0.0408–0.654
**46**	330	0.512–2.71	0.421–2.15	0.364–1.81	0.331–1.62	0.315–1.53	0.315–1.53	0.331–1.62
**47**	330	0.369–1.95	0.466–2.38	0.553–2.75	0.618–3.02	0.654–3.17	0.654–3.17	0.618–3.02
**48**	375	0.0065–1.27	0.0362–1.53	0.0707–1.73	0.0946–1.84	0.0994–1.86	0.0835–1.79	0.0522–1.63
**49**	339	0.630–4.17	0.592–3.86	0.689–4.65	0.792–5.50	0.861–6.09	0.886–6.30	0.886–6.30
**50**	379	0.201–1.14	0.234–1.36	0.261–1.55	0.281–1.69	0.290–1.76	0.287–1.74	0.272–1.63
**51**	366	0.781–38.5	0.324–21.4	0.233–17.2	0.307–20.7	0.468–27.3	0.536–29.9	0.536–29.9
**52**	379	0.254–1.36	0.260–1.40	0.266–1.44	0.272–1.48	0.279–1.52	0.286–1.57	0.293–1.61
**53**	329	0.405–1.21	0.511–1.49	0.531–1.54	0.513–1.49	0.517–1.50	0.545–1.58	0.600–1.72
**54**	353	0.196–0.523	0.237–0.660	0.256–0.724	0.249–0.699	0.242–0.676	0.240–0.668	0.240–0.668
**55**	329	0.712–0.952	0.689–0.929	0.703–0.943	0.717–0.957	0.725–0.965	0.728–0.968	0.728–0.968
**56**	329	0.828–2.47	0.672–1.96	0.649–1.88	0.670–1.95	0.665–1.93	0.634–1.83	0.581–1.67
**57**	353	1.91–5.10	1.51–4.21	1.38–3.91	1.43–4.02	1.48–4.14	1.50–4.17	1.50–4.17
**58**	326	0.522–1.95	0.825–2.59	0.909–2.75	0.945–2.82	0.967–2.86	0.974–2.88	0.974–2.88
**59**	337	0.762–3.82	1.12–4.72	1.31–5.14	1.36–5.27	1.40–5.34	1.41–5.37	1.41–5.37
**60**	353	0.872–3.07	0.872–3.07	0.872–3.07	0.872–3.07	0.872–3.07	0.872–3.07	0.872–3.07
**61**	339	0.0104–0.113	0.0096–0.102	0.0089–0.0922	0.0082–0.0835	0.0076–0.0756	0.0070–0.0686	0.0065–0.0623
**62**	367	37.8–1986	28.9–1519	22.1–1161	16.9–888	12.9–679	9.89–519	7.56–397
**63**	344	0.138–3.10	0.138–3.10	0.138–3.10	0.138–3.10	0.138–3.10	0.138–3.10	0.138–3.10
**64**	329	0.169–4.13	0.154–3.46	0.140–2.91	0.128–2.47	0.117–2.11	0.107–1.80	0.0984–1.55
**65**	341	0.223–16.5	0.237–18.1	0.252–19.8	0.267–21.8	0.284–24.0	0.302–26.4	0.322–29.1

Reference intervals are given as 2.5^th^-97.5^th^ percentiles and were obtained from the described statistical models. Three significant digits were indicated for most percentiles. N represents the sample number per ratio. The corresponding 1^st^-, 50^th^- and 99^th^-percentiles are shown in **S4 Table**.

**Table 3:** Reference intervals for diagnostic ratios of 24-hour urine steroid hormone metabolites in men of different ages.

Men		Age, years						
Ratio ID	N	20	30	40	50	60	70	80
**1**	407	0.0020–0.0194	0.0020–0.0192	0.0020–0.0202	0.0021–0.0228	0.0023–0.0277	0.0025–0.0371	0.0029–0.0567
**2**	321	0.0010–0.0096	0.0010–0.0103	0.0010–0.0110	0.0011–0.0117	0.0011–0.0125	0.0011–0.0135	0.0012–0.0145
**3**	402	0.0226–0.222	0.0211–0.207	0.0196–0.193	0.0183–0.180	0.0170–0.167	0.0159–0.156	0.0148–0.145
**4**	320	0.0125–0.112	0.0114–0.102	0.0104–0.0934	0.0095–0.0853	0.0087–0.0778	0.0079–0.0711	0.0072–0.0649
**5**	362	0.124–0.770	0.112–0.698	0.102–0.633	0.0923–0.574	0.0837–0.521	0.0759–0.472	0.0688–0.428
**6**	303	0.0693–0.408	0.0620–0.357	0.0555–0.313	0.0498–0.275	0.0447–0.242	0.0401–0.213	0.0361–0.188
**7**	360	0.160–0.940	0.146–0.862	0.134–0.791	0.123–0.725	0.113–0.665	0.104–0.610	0.0950–0.560
**8**	303	0.0900–0.499	0.0811–0.441	0.0731–0.391	0.0660–0.346	0.0597–0.307	0.0540–0.273	0.0489–0.243
**9**	402	0.0222–0.428	0.0279–0.486	0.0154–0.351	0.0080–0.249	0.0047–0.191	0.0032–0.161	0.0027–0.149
**10**	321	0.0110–0.226	0.0123–0.241	0.0075–0.183	0.0033–0.121	0.0017–0.0878	0.0010–0.0715	0.0009–0.0663
**11**	395	0.0153–4.29	0.0241–20.4	0.0171–6.06	0.0094–1.07	0.0062–0.399	0.0049–0.228	0.0043–0.178
**12**	315	0.0073–2.81	0.0109–12.2	0.0078–3.55	0.0044–0.615	0.0029–0.212	0.0021–0.107	0.0017–0.0712
**13**	389	0.0496–3.04	0.0765–5.89	0.0510–3.17	0.0257–1.14	0.0161–0.583	0.0122–0.394	0.0111–0.342
**14**	312	0.0241–1.66	0.0365–3.15	0.0243–1.68	0.0121–0.591	0.0073–0.284	0.0053–0.176	0.0044–0.136
**15**	448	3.12–95.3	4.41–114	2.12–78.6	0.883–52.4	0.398–37.5	0.208–29.1	0.137–25.0
**16**	407	0.0100–0.0459	0.0088–0.0395	0.0095–0.0434	0.0106–0.0491	0.0117–0.0551	0.0128–0.0612	0.0139–0.0674
**17**	321	0.0055–0.0227	0.0046–0.0176	0.0051–0.0205	0.0058–0.0241	0.0062–0.0267	0.0064–0.0276	0.0064–0.0276
**18**	361	0.0209–0.101	0.0210–0.102	0.0220–0.107	0.0240–0.117	0.0274–0.133	0.0325–0.158	0.0402–0.195
**19**	359	0.0494–0.243	0.0524–0.267	0.0570–0.307	0.0637–0.370	0.0734–0.472	0.0874–0.644	0.108–0.962
**20**	404	0.114–0.408	0.102–0.375	0.0950–0.354	0.0916–0.344	0.0917–0.344	0.0953–0.355	0.103–0.376
**21**	320	0.0601–0.177	0.0577–0.171	0.0545–0.163	0.0506–0.154	0.0462–0.143	0.0453–0.141	0.0521–0.158
**22**	451	0.504–4.17	0.471–3.83	0.453–3.65	0.449–3.61	0.459–3.71	0.482–3.95	0.523–4.37
**23**	395	0.131–0.631	0.150–0.721	0.152–0.730	0.140–0.675	0.139–0.670	0.153–0.734	0.185–0.888
**24**	392	0.102–0.495	0.114–0.546	0.115–0.552	0.108–0.522	0.108–0.520	0.116–0.555	0.136–0.634
**25**	340	0.943–4.74	0.784–4.05	0.649–3.46	0.536–2.94	0.441–2.49	0.361–2.11	0.294–1.78
**26**	280	0.536–2.38	0.435–1.99	0.352–1.66	0.283–1.37	0.226–1.14	0.180–0.936	0.143–0.768
**27**	427	0.0628–1.72	0.0407–1.11	0.0472–1.29	0.0680–1.86	0.0847–2.32	0.0911–2.49	0.0911–2.49
**28**	446	0.350–4.20	0.290–3.85	0.261–3.67	0.257–3.64	0.278–3.77	0.328–4.07	0.415–4.56
**29**	447	0.0677–1.96	0.0924–2.20	0.0675–1.95	0.0485–1.74	0.0391–1.62	0.0362–1.58	0.0362–1.58
**30**	426	0.0291–0.449	0.0250–0.420	0.0247–0.419	0.0284–0.444	0.0370–0.500	0.0432–0.538	0.0432–0.538
**31**	443	0.0681–0.637	0.0618–0.599	0.0560–0.563	0.0507–0.529	0.0458–0.496	0.0412–0.465	0.0371–0.436
**32**	391	0.397–2.45	0.358–2.21	0.324–2.00	0.292–1.80	0.264–1.63	0.238–1.47	0.215–1.33
**33**	389	0.502–3.01	0.455–2.73	0.413–2.47	0.375–2.24	0.340–2.03	0.308–1.84	0.279–1.67
**34**	358	0.0099–0.0884	0.0114–0.0966	0.0130–0.105	0.0148–0.115	0.0168–0.125	0.0190–0.136	0.0214–0.147
**35**	336	0.0849–0.289	0.0856–0.292	0.0887–0.308	0.0944–0.337	0.104–0.387	0.117–0.467	0.138–0.597
**36**	334	0.104–0.355	0.105–0.363	0.110–0.384	0.118–0.421	0.131–0.479	0.149–0.569	0.176–0.705
**37**	424	0.120–6.34	0.0547–3.76	0.100–5.60	0.289–11.5	0.591–19.0	0.918–26.0	1.12–29.9
**38**	445	0.877–20.0	0.508–13.5	0.704–17.1	1.37–27.9	2.32–41.1	3.43–55.3	4.54–68.4
**39**	424	0.120–4.31	0.0597–2.66	0.100–3.79	0.256–7.35	0.496–11.9	0.766–16.3	0.968–19.4
**40**	423	0.107–1.71	0.0684–1.23	0.104–1.68	0.215–2.93	0.366–4.42	0.526–5.87	0.650–6.94
**41**	360	0.155–0.944	0.142–0.861	0.129–0.785	0.118–0.716	0.107–0.653	0.0979–0.595	0.0893–0.543
**42**	303	0.0877–0.498	0.0788–0.438	0.0708–0.386	0.0638–0.341	0.0575–0.302	0.0518–0.267	0.0468–0.237
**43**	407	0.0316–0.293	0.0262–0.253	0.0227–0.226	0.0207–0.210	0.0198–0.202	0.0199–0.203	0.0211–0.213
**44**	321	0.0156–0.133	0.0138–0.123	0.0122–0.114	0.0107–0.105	0.0094–0.0964	0.0082–0.0887	0.0072–0.0814
**45**	280	0.536–2.38	0.435–1.99	0.352–1.66	0.283–1.37	0.226–1.14	0.180–0.936	0.143–0.768
**46**	361	0.610–2.89	0.570–2.76	0.533–2.62	0.498–2.50	0.465–2.38	0.433–2.26	0.403–2.15
**47**	361	0.346–1.64	0.363–1.75	0.381–1.88	0.400–2.01	0.421–2.15	0.443–2.31	0.466–2.48
**48**	447	0.0479–1.19	0.0227–0.989	0.0403–1.14	0.0640–1.29	0.0815–1.40	0.0879–1.43	0.0879–1.43
**49**	351	0.466–2.23	0.518–2.65	0.565–3.05	0.603–3.40	0.627–3.64	0.637–3.73	0.631–3.67
**50**	457	0.165–0.829	0.186–0.983	0.201–1.10	0.208–1.15	0.206–1.13	0.210–1.16	0.239–1.40
**51**	451	7.62–97.1	5.93–83.6	4.55–71.6	3.44–61.1	2.56–51.8	1.87–43.7	1.33–36.6
**52**	456	0.276–1.21	0.325–1.42	0.309–1.36	0.299–1.31	0.306–1.34	0.331–1.45	0.378–1.66
**53**	321	0.598–1.54	0.622–1.63	0.647–1.73	0.674–1.83	0.702–1.95	0.731–2.07	0.763–2.21
**54**	398	0.241–0.640	0.268–0.741	0.271–0.753	0.265–0.729	0.267–0.735	0.276–0.771	0.295–0.843
**55**	321	0.614–0.900	0.619–0.905	0.624–0.910	0.629–0.915	0.634–0.920	0.639–0.925	0.645–0.930
**56**	321	0.650–1.67	0.614–1.61	0.579–1.55	0.546–1.48	0.513–1.43	0.482–1.37	0.452–1.31
**57**	398	1.56–4.16	1.35–3.73	1.33–3.68	1.37–3.77	1.36–3.75	1.30–3.62	1.19–3.39
**58**	328	1.28–3.38	1.43–3.98	1.41–3.89	1.37–3.75	1.35–3.67	1.35–3.65	1.35–3.65
**59**	345	1.38–5.00	1.45–5.21	1.52–5.42	1.59–5.65	1.66–5.88	1.74–6.13	1.82–6.38
**60**	398	0.642–2.16	0.681–2.26	0.721–2.36	0.763–2.47	0.807–2.58	0.853–2.70	0.902–2.82
**61**	351	0.0057–0.0398	0.0057–0.0398	0.0057–0.0398	0.0057–0.0398	0.0057–0.0398	0.0057–0.0398	0.0057–0.0398
**63**	424	0.147–2.93	0.154–3.10	0.160–3.28	0.167–3.47	0.174–3.68	0.182–3.89	0.190–4.13
**64**	320	0.0773–1.13	0.0773–1.13	0.0773–1.13	0.0773–1.13	0.0773–1.13	0.0773–1.13	0.0773–1.13
**65**	432	0.470–32.3	0.470–32.3	0.470–32.3	0.470–32.3	0.470–32.3	0.470–32.3	0.470–32.3

Reference intervals are given as 2.5^th^-97.5^th^ percentiles and were obtained from the described statistical models. Three significant digits were indicated for most percentiles. N represents the sample number per ratio. The corresponding 1^st^-, 50^th^- and 99^th^-percentiles are shown in **S5 Table**.

### Comparison with published diagnostic ratios from the literature

As shown in **S1 Table,** mean values and ranges of ratios published by C. H. Shackleton in 1986 for women and men aged 22–50 years old were located within the values for the 2.5^th^-97.5^th^ percentiles published in the current study (hereafter called current reference intervals), except for a lower ratio 23 in women and a higher ratio 64 in men [10]. Among joint values for women and men on 16 diagnostic ratios published by C. H. Shackleton later on in 2006 and 2008 the value for ratio 64 was then within current reference intervals [11,12]. Age- and sex-related values provided for ratio 47 and ratio 49, both reflecting 5α-reductase deficiency, published by C. W. Weykamp et al. in 1989 were all within current reference intervals [14]. The same applied for values obtained from different publications: (1) by Farese et al. in 1991 concerning ratios 49, 56 and 57, the latter two reflecting 11β-HSD type 1 deficiency, (2) by Soro et al. in 1995 concerning ratio 49 and ratio 53 (11β-HSD type 1 deficiency), (3) by Finken et al. in 1999 concerning ratio 49 and ratio 52 (11β-HSD type 1 deficiency), and (4) by Vulto et al. in 2020 concerning ratio 52 and ratio 53 [15–17,20]. Values published by Finken et al. in 1999 for ratio 53 were remarkably higher than the current reference intervals for both sexes and also higher than all other corresponding values cited in **S1 Table**. Values of 2.5^th^-97.5^th^ percentiles for ratio 49 from a population from Hong Kong published by Chan et al. in 2008 were slightly shifted towards lower values in women compared to the current reference intervals and the same applied for ratio 53 in men [18]. Finally, sex- and age related values for twelve ratios published by de Jong in 2017 were very similar to the current reference intervals [13].

## Discussion

We created sex- and age-specific reference intervals for diagnostic ratios based on the urinary steroid hormone profile measured by GC-MS in a large number of thoroughly characterized women and men of European descent randomly selected from the general adult population aged 18 years and over. To our knowledge, this is the largest and most comprehensive study of this kind based on a population-based sample from the general population, thereby enhancing external validity of the findings. Compared to previous work in this field the present study was large enough to model the relationship of steroidogenic ratios with age across the whole age spectrum in adults stratified by sex.

Unlike immunoassays, chromatography-mass spectrometry techniques are known for their high specificity and are increasingly preferred for steroid hormone diagnostic in clinic and research. With regard to absolute urinary steroid excretion values, laboratories, using different in-house adapted GC-MS methods, should create their own laboratory specific reference intervals as recently pointed out [22]. However, what is striking in the current study is the fact that the overwhelming majority of values for diagnostic ratios published from 1986 to 2020 lies within or very close to the current reference intervals estimated from statistical models. Accordingly, values for diagnostic ratios previously published from different laboratories are also very similar to each other. Thus, diagnostic ratios derived from urinary steroid hormone profiles measured by different in-house adapted GC-MS methods in different laboratories show a very high degree of comparability–in general a higher degree than the respective underlying steroid hormone metabolites.

The concept of steroidogenic ratios in health assumes the presence of a reproducible ratio between the involved steroidogenic metabolites within certain limits and a marked change of this ratio in certain conditions or diseases. The size of this change is part of the diagnostic value of the ratio. As our published results are similar to the vast majority of previously published diagnostic ratios they are at least in accordance with this concept. Significant deviations from reference intervals for one or more diagnostic ratios should consequently foster diagnostic workup for the underlying cause. Beside this, the high levels of agreement in healthy people make diagnostic ratios suitable as a means of quality control for underlying steroid hormone metabolites measured by different methods.

Nevertheless, as mentioned in the results section, there are some differences between previously and currently published reference intervals. Possible causes, like differences in the recruited reference population or technical method used, remain speculative. At this point, we would like to emphasize the fact that we calculated diagnostic ratios from urinary steroid hormone metabolites after conversion from mass to molar mass thereby taking account of the molar ratio between steroid metabolites. Thus, differences may be explained by the different ways of calculations in some cases.

Several strengths with regard to methodological aspects were already previously discussed within the publication of reference intervals for the urinary steroid hormone profile and they also apply to the current study [22]. In addition, most ratios presented bimodal sex-related distributions and different kinds of relationships with age for both sexes stressing the importance to model ratios as a function of age separately for each sex. The high similarity in distributions of ratios derived from 24-hour, day- and night-time urine across centers can be interpreted as an additional post-analytical quality control. It argues against the presence of systematic center-specific differences in the pre-analytical processes, specifically in the collection, handling, and storage of samples for urine analysis. It also argues against population-related or regional circumstances that may have systematically influenced the results of urinary steroid hormone measurement and thus steroid ratios in different directions. By contrast, a much higher proportion of variance was attributable to a family effect, thereby highlighting the importance of shared genetic and/or environmental effects.

Apart from predefined types of models which were flexible enough to provide a good approximation of the reality in most cases, no further model assumptions were made as described in the methods section and in **S1 Text**. Even whenever several ratios were assigned to the same enzyme activity, sex- and age-dependency of ratios were not modelled in groups of enzyme activities, but each ratio was modelled independently. Thus, the selected models and derived reference curves were not forced to behave uniformly but according to the underlying data, resulting in part in different sex- and age-related behaviors for ratios assigned to the same enzyme activity, e.g. for ratios 1–8 all estimating 21-hydroxylase activity.

However, this does not need to be a contradiction in term, as the steroids used in the numerator of these ratios are metabolized by steroidogenic enzymes following different sequences. In particular, 17α-OH-pregnanolone (17HP) used in ratios 3 and 4 is metabolized by enzymes in the following order through the Δ^5^-pathway: *17α-hydroxylase/P450 oxidoreductase→3β-hydroxysteroid dehydrogenase→5β-reductase→3α-hydroxysteroid dehydrogenase*. 17HP is further metabolized by the action of 20α-hydroxysteroid dehydrogenase to pregnanetriol (PT) used in ratios 5 and 6. In contrast to 17HP and PT the metabolism of pregnanetriolone (PTO) used in ratios 1 and 2 follows a different sequence of enzyme activities: *17α-hydroxylase/P450 oxidoreductase→3β-hydroxysteroid dehydrogenase→11β-hydroxylase→5β-reductase→3α-hydroxysteroid dehydrogenase→20α-hydroxysteroid dehydrogenase*. Thus, intermediary metabolisms of 17HP and PT are more similar to each other compared to PTO, and this most likely influences the behavior of modelled ratios. All three metabolites can be used in the numerator of ratios together with glucocorticoid metabolites as denominators to assess 21-hydroxylase activity/deficiency, because each of the metabolites 17HP, PT, and PTO will usually increase if 21-hydroxylase deficiency is present, whereas glucocorticoid metabolites will usually decrease resulting in throughout higher values for ratios 1–8. Thus, different behaviors of modelled reference curves of ratios used to assess the same enzyme activity also reflects different intermediary pathways and the resulting reference curves remain still useful to assess 21-hydroxylase activity/deficiency. Besides, reference curves for ratios 7 and 8, both containing the sum of 17HP, PT, and PTO in the numerator, are similar to reference curves for ratios 3–6 but not to those for ratios 1–2, as PTO constitutes to the sum of the three metabolites only about 3% in women and less than 2% in men [22].

Diagnostic ratios significantly differed between day- and night-time collections for 60 out of 65 diagnostic ratios in women and for 55 out of 64 diagnostic ratios in men. The day-night differences observed for most of the diagnostic ratios suggest a circadian rhythm for enzymes involved in human steroid hormones metabolism. This is not a surprise because of the known interplay between circadian rhythm and steroid hormones [26,27] and the role of altered circadian rhythm in selected diseases and conditions [28]. Given the importance of circadian rhythms in mammals in general [29,30], the ability to detect abnormal circadian rhythm of steroid hormones metabolism in humans could help further our understanding and clinical diagnosis of a wide range of disorders.

The inverse association of age with ratio 62 used to detect PCOS extend previous results from a SKIPOGH-subgroup analysis [9]. In this previous study an inverse association between age and ratio 62 was found in healthy women aged ≤ 46 years whereas in women with PCOS this association was not significant but tended to be positive. Under the assumption that this association with age does not rigorously change in postmenopausal women with PCOS, the present results may indicate that the diagnostic accuracy of ratio 62 for PCOS may improve with increasing age even beyond the age of 46 years old. This may be of clinical value as there is still no conclusive test to diagnose PCOS, and it is well known that after menopause metabolic consequences of PCOS do not disappear at all [31].

A current weakness is, that this study created reference intervals for 24-hour urinary steroid hormone metabolite ratios taking into account sex and age but no other potentially influencing factors like body mass index or obesity, kidney function, smoking, hypertension, or dyslipidemia. In addition, information about the phase of menstrual cycle is not available. This work still needs to be done and may further increase clinical utility of urinary steroid hormone profiling. We emphasize the need for this work to continue as from the clinical point of view there is much in the area of urinary steroid profiling that has not yet been sufficiently in the general adult population. However, the present work adds a useful tool to the powerful toolbox of urinary steroid profiling in routine clinical work and research and we hope that this tool may help to distinguish between health and disease when investigating human disorders affecting steroid synthesis and metabolism.

## Supporting information

S1 Fig Distribution of diagnostic urinary steroid hormone metabolite ratios. Kernel density plot histograms of diagnostic ratios are shown from left to right for 24-hour urine collections (left panels), daytime urine collections (center panels), and night-time urine collections (right panels) and from top to the bottom for the entire cohort (upper panels), separated for women and men (center panels), and separated for the study centers in Bern (BE), Geneva (GE), and Lausanne (LS).
(PDF)

S2 Fig Distribution of diagnostic urinary steroid hormone metabolite ratios without outliers. Figures from **S1 Fig** were remade and the tail from extremely right-skewed distributions were truncated after visual inspection for improved visibility.
(PDF)

S3 Fig Descriptive analyses of diagnostic urinary steroid hormone metabolite ratios. A descriptive analysis of 65 calculated ratios is shown including one ratio per page. Panel (a): boxplots, ratio (log-scale) by sex; Panel (b): Gasser-Müller nonparametric fit and scatter plot, ratio (log-scale) by age and by sex; Panel (c) boxplot, transformed ratio for men according to optimal power transformation, outliers are plotted as black dots; Panel (d) boxplot, transformed ratio for women according to optimal power transformation, outliers are plotted as black dots; Panel (e): ratio (log-scale) by day and night time for men; Panel (f): ratio (log-scale) by day and night time for women; Panel (g): Spearman rank correlation of transformed ratio night vs day for men; Panel (h): Spearman rank correlation of transformed steroid night vs day for women. Abbreviations used: M = men; W = women; D = day; N = night; TR = optimal power transformation; nout = number of outliers; ku = kurtosis; sk = skewness; delta = robust estimate of mean difference expressed in standard deviations; rho = Spearman rank correlation coefficient; n = sample size.
(PDF)

S4 Fig Reference curves of diagnostic urinary steroid hormone metabolite ratios. Reference curves of 65 calculated ratios are shown including one ratio per page. The percentiles 2.5, 10, 25, 50, 75, 90 and 97.5 of the ratios in function of age and sex are shown on a log-scale. To facilitate comparison the same scale has been used for men and women.
(PDF)

S1 Table Published diagnostic ratios based on urinary steroid hormone metabolites. Underlying metabolites were mainly measured by gas chromatography–mass spectrometry (GC-MS). Ratios from healthy women (W) and men (M) have been considered since 1986. Ratios are dimensionless and have been described by descriptive statistics as indicated (type of data). The methods of analysis and the units in which metabolites have been measured in order to create ratios are also indicated. The population and subgroups ratios derives from are further described where available. Abbreviations: SD, standard deviation; SEM; standard error of the mean.
(PDF)

S2 Table Sex specific differences in diagnostic ratios based on steroid hormone metabolites measured in 24-hour urine. The available number of participants is indicated for each diagnostic ratio. Diagnostic ratios are stratified for sex and are described by their median;25^th^-75^th^ percentile. Between-group differences were determined by Mann–Whitney U test, and the corresponding *p* values are indicated.
(PDF)

S3 Table Day- and night-time specific differences for diagnostic ratios based on steroid hormone metabolites measured in day and nighttime urine. The available number of participants is indicated for each diagnostic ratio stratified for sex. Diagnostic ratios are described by their median;25^th^-75^th^ percentile. Within-sex differences were determined by Wilcoxon signed-rank test, and the corresponding *p* values are indicated.
(PDF)

S4 Table 1^st^-50^th^-99^th^ percentiles for diagnostic ratios of 24-hour urine steroid hormone metabolites in women of different ages. Percentiles were obtained from the described statistical models. Three significant digits were indicated for most percentiles. N represents the sample number per ratio.
(PDF)

S5 Table 1^st^-50^th^-99^th^ percentiles for diagnostic ratios of 24-hour urine steroid hormone metabolites in men of different ages. Percentiles were obtained from the described statistical models. Three significant digits were indicated for most percentiles. N represents the sample number per ratio.
(PDF)

S1 Text Statistical methods. The statistical methods are described in detail.
(DOCX)
