# Supplementary figures and images for: Sex- and age-specific reference intervals for diagnostic ratios reflecting relative activity of steroidogenic enzymes and pathways in adults
Source: PLoS One. 2021 Jul 8;16(7):e0253975. doi: 10.1371/journal.pone.0253975 (PMC8266106; doi:10.1371/journal.pone.0253975)

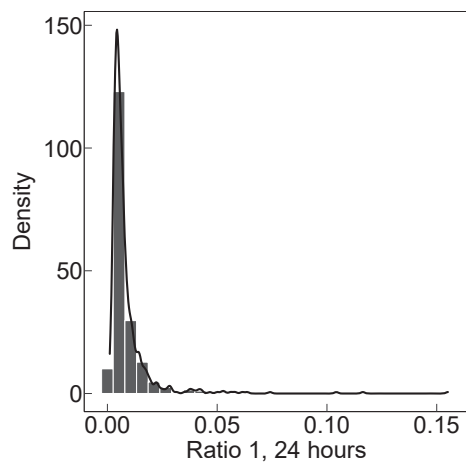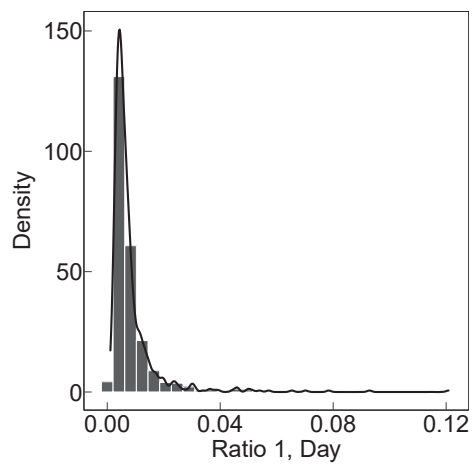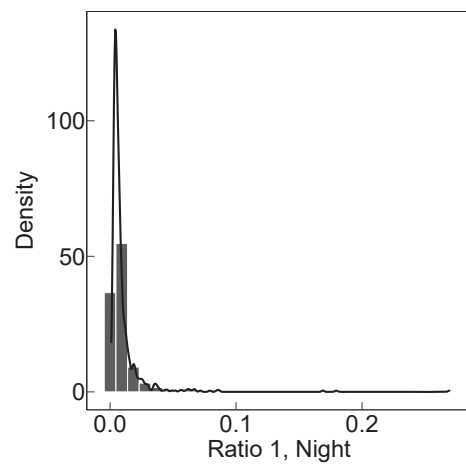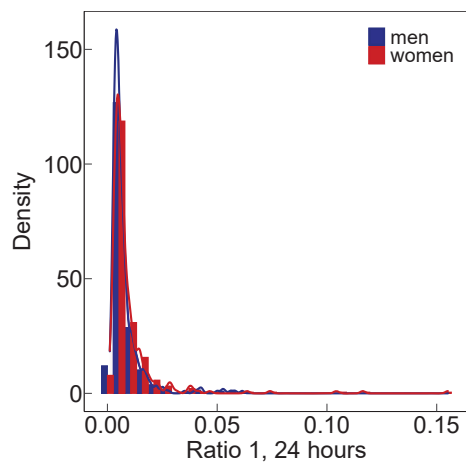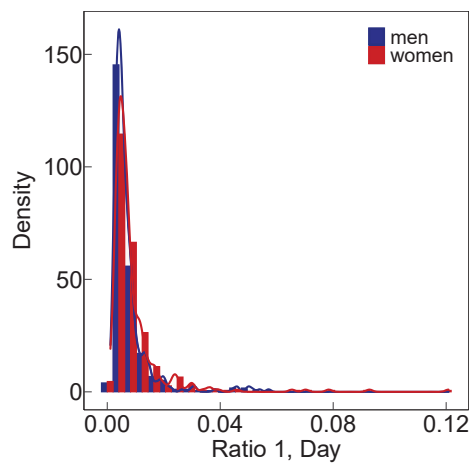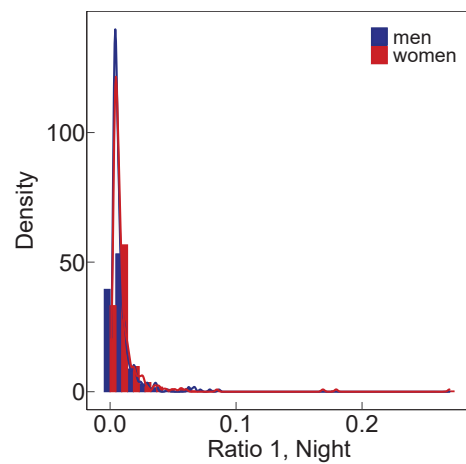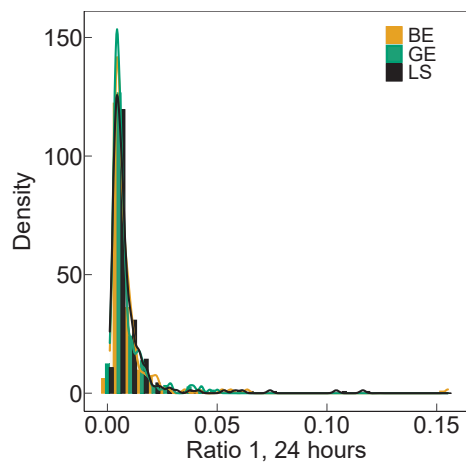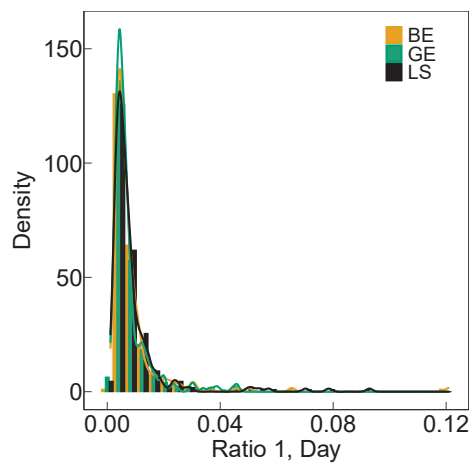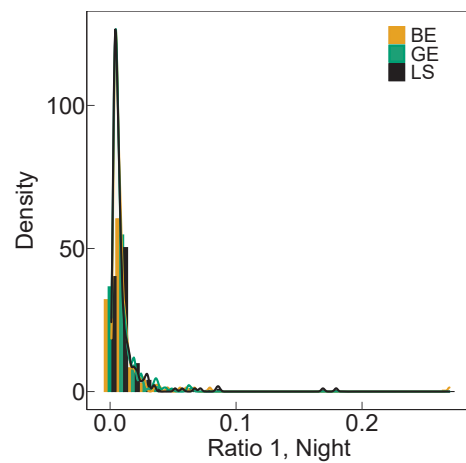

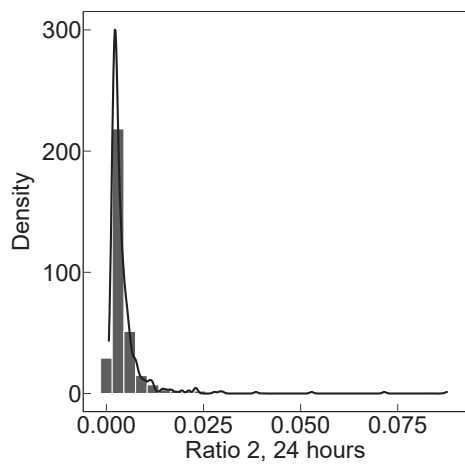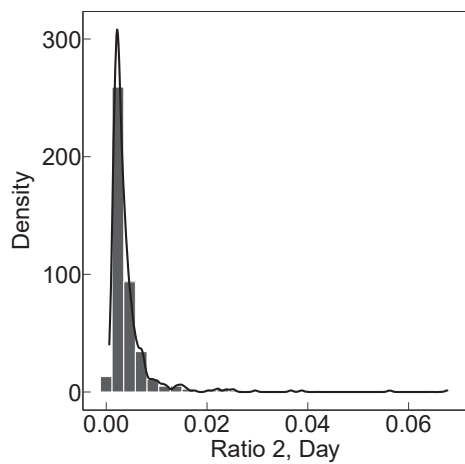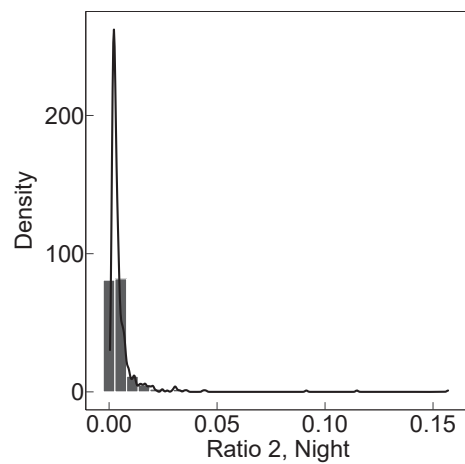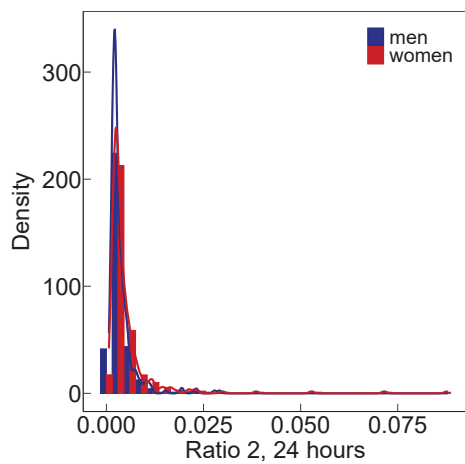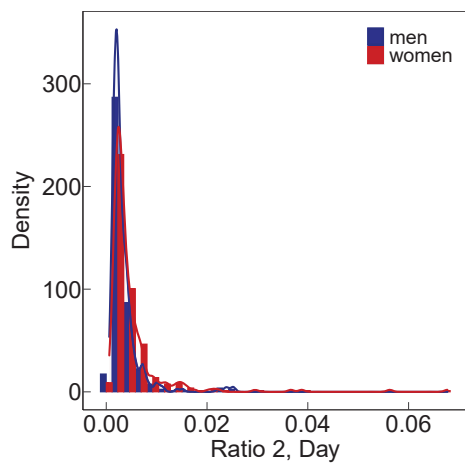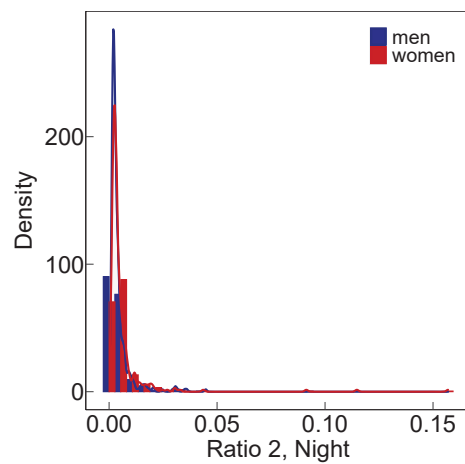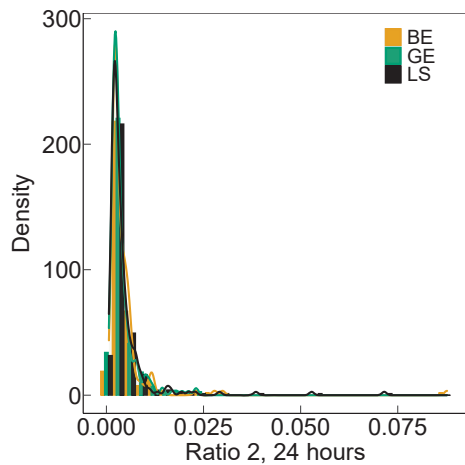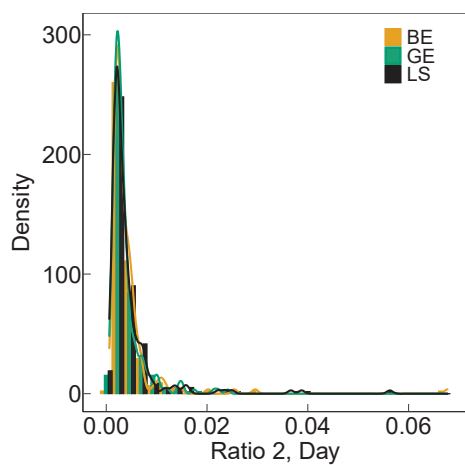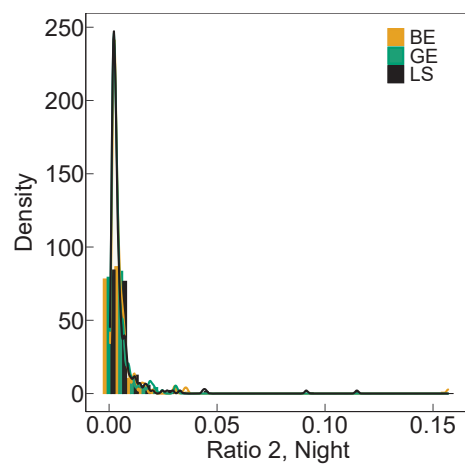

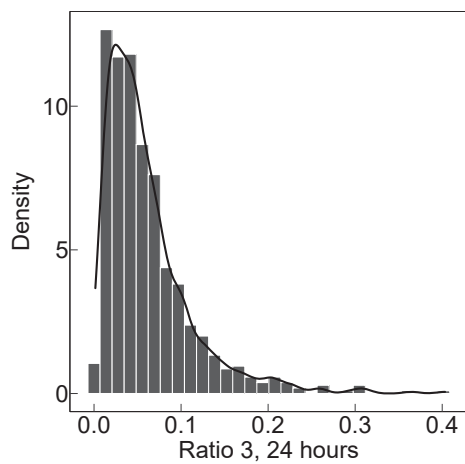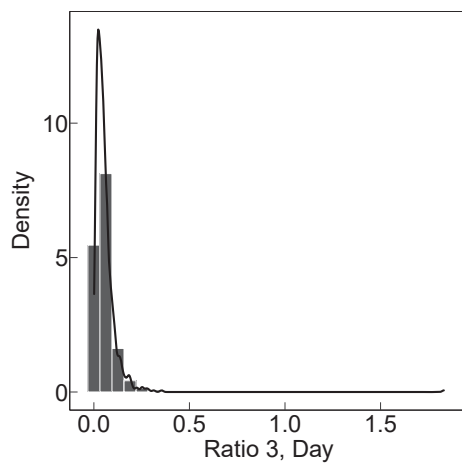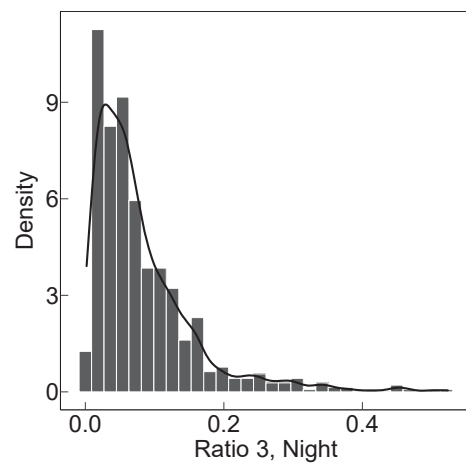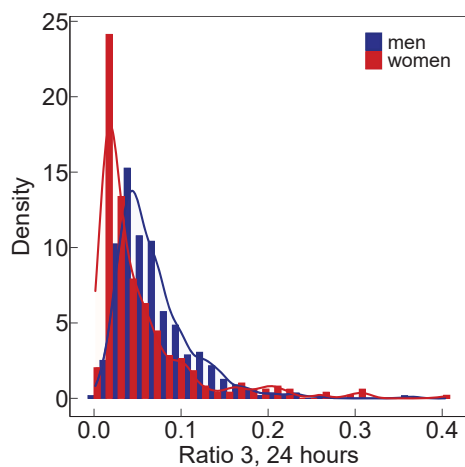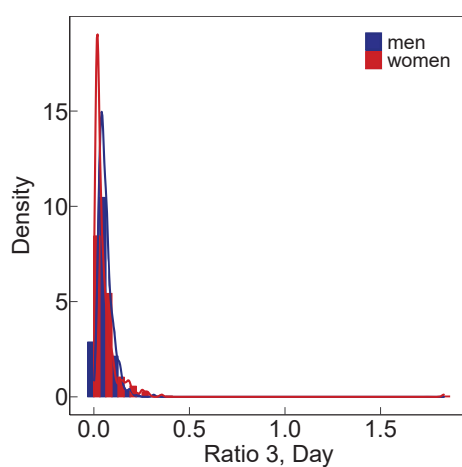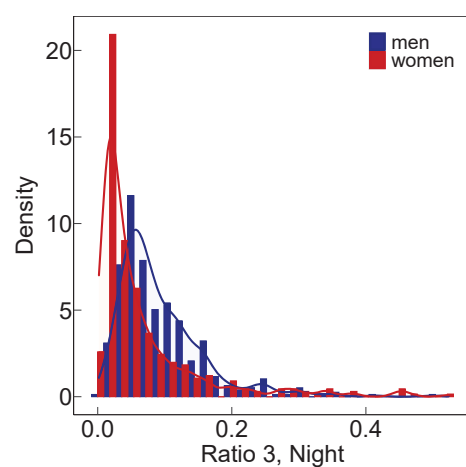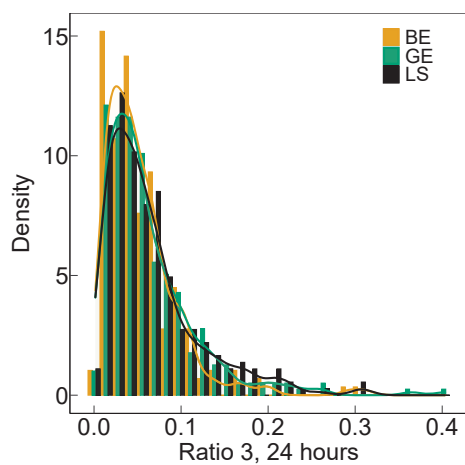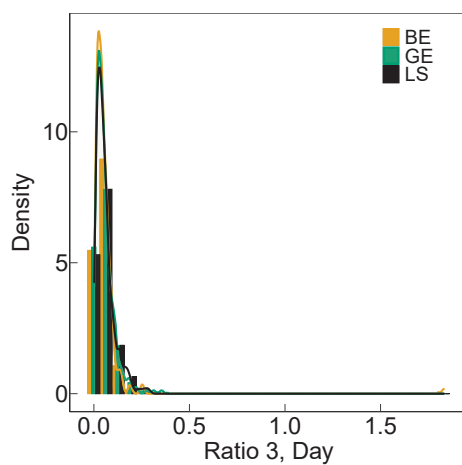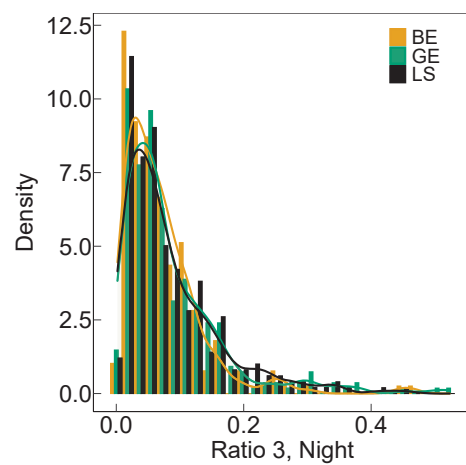

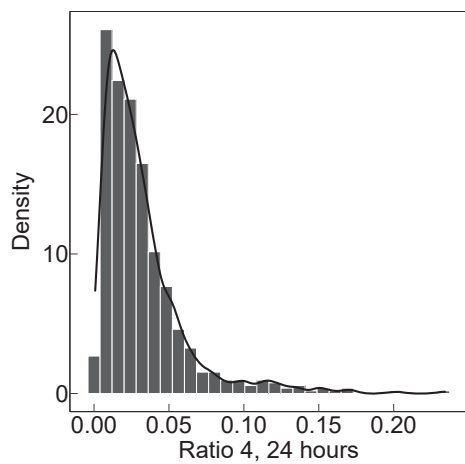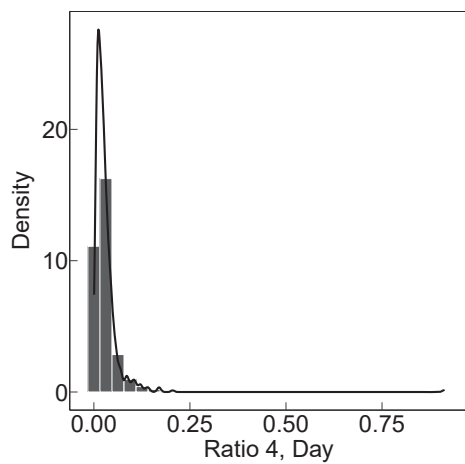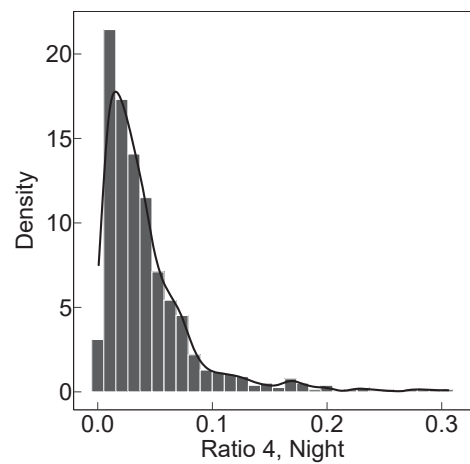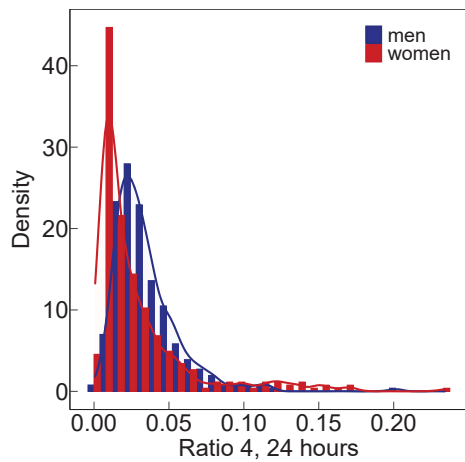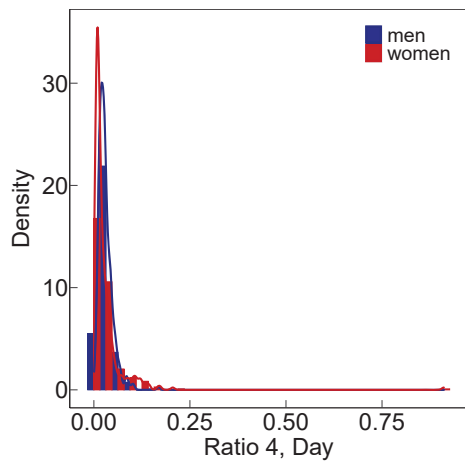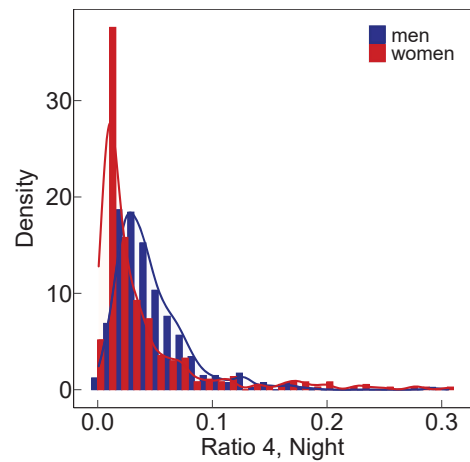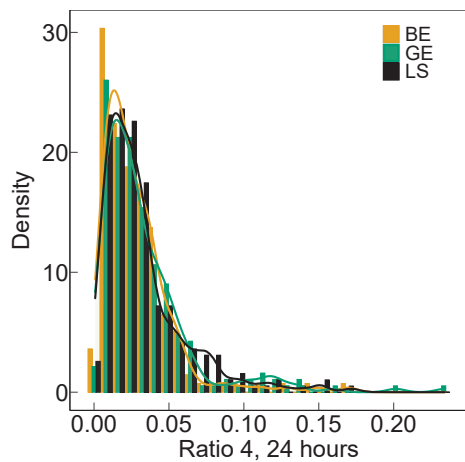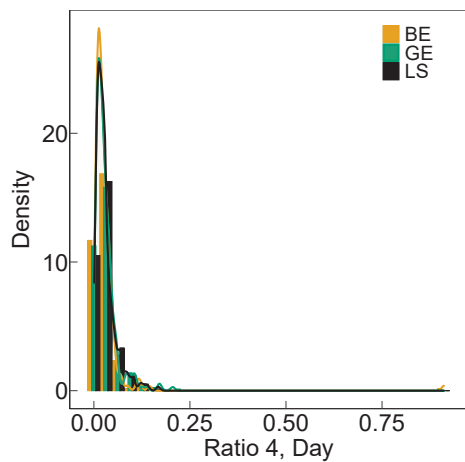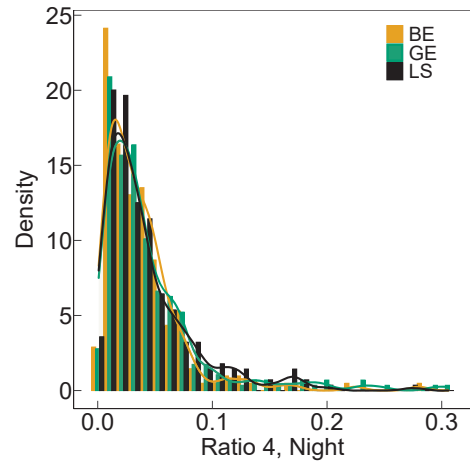

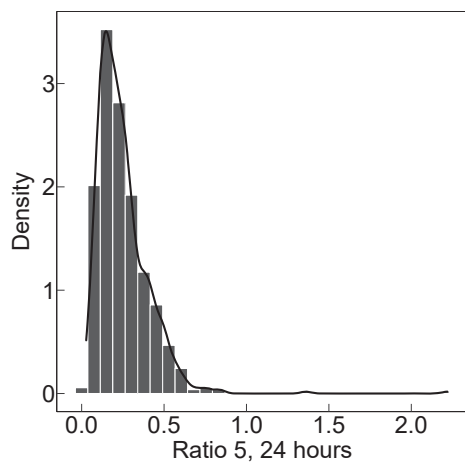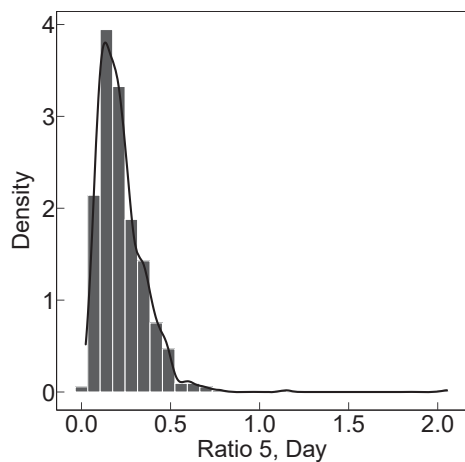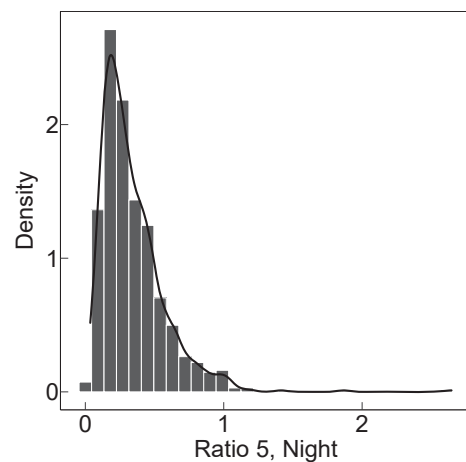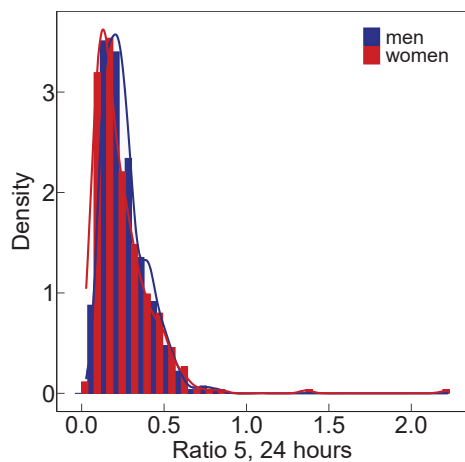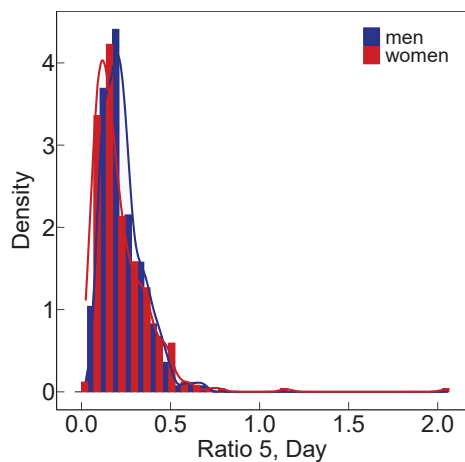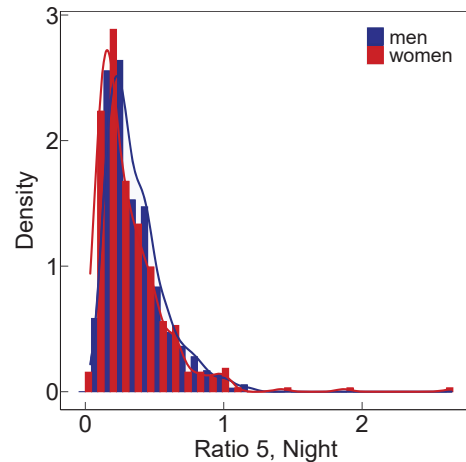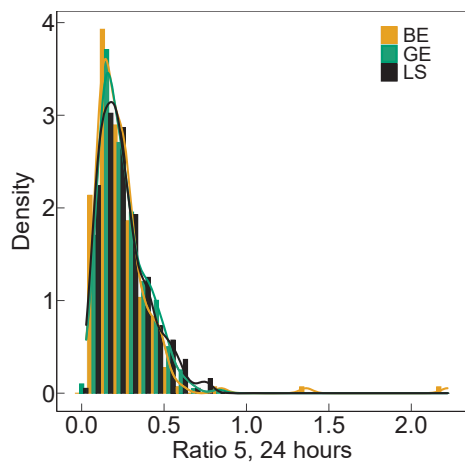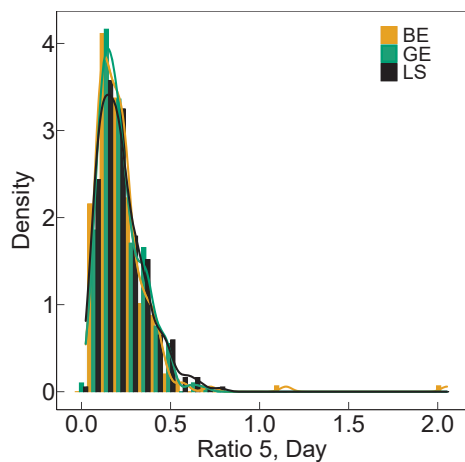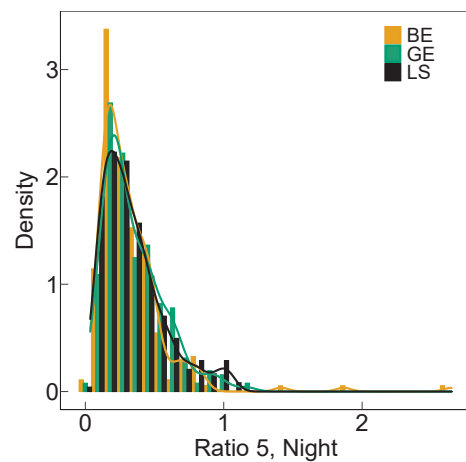

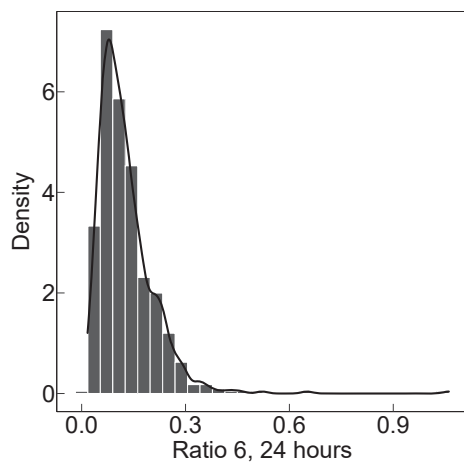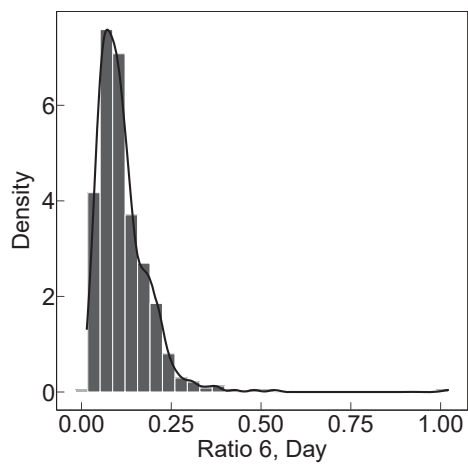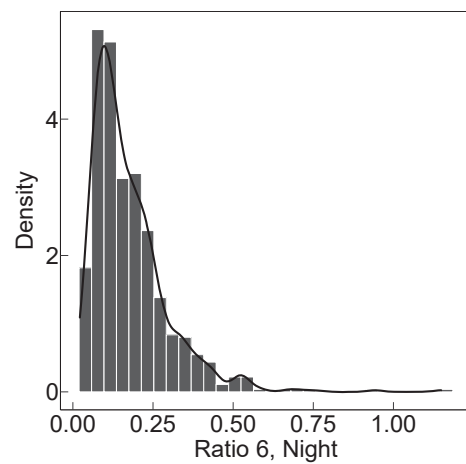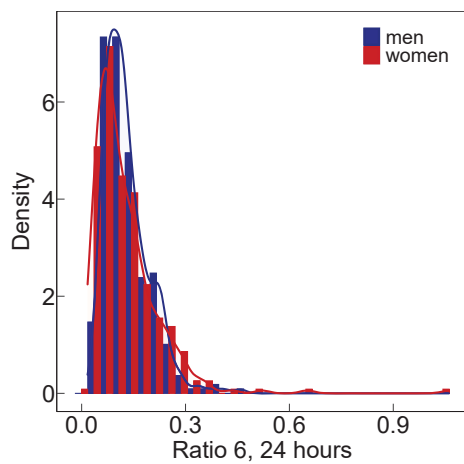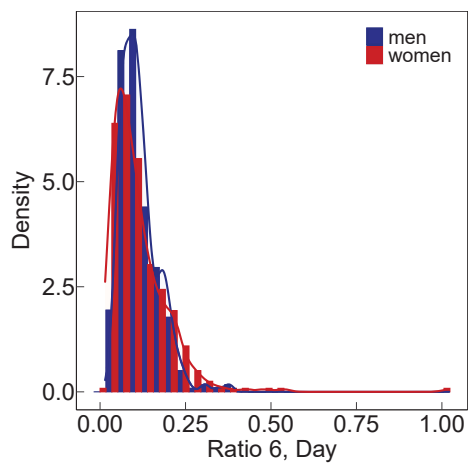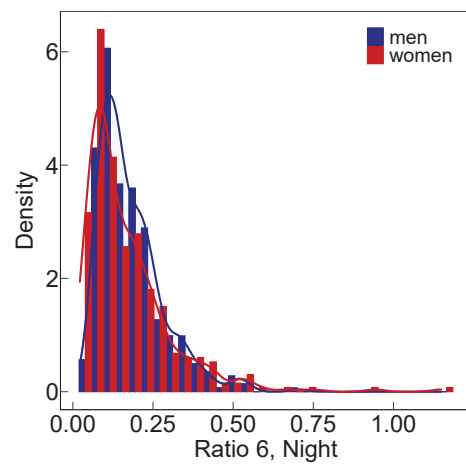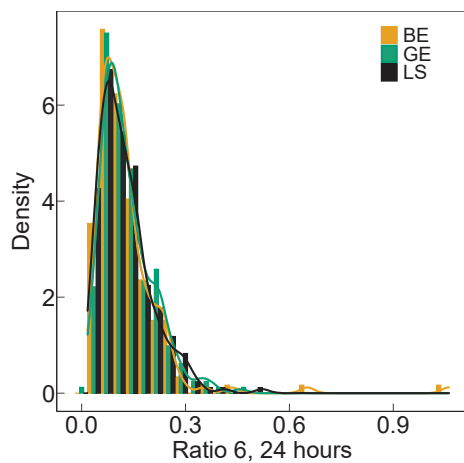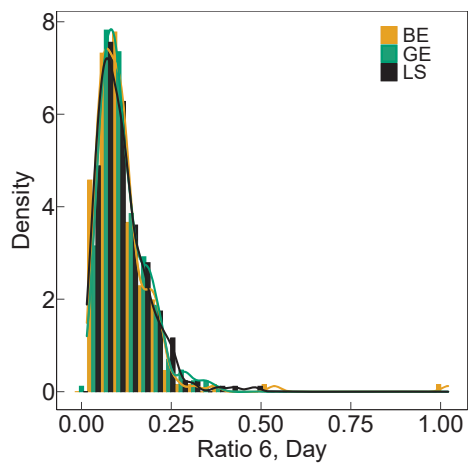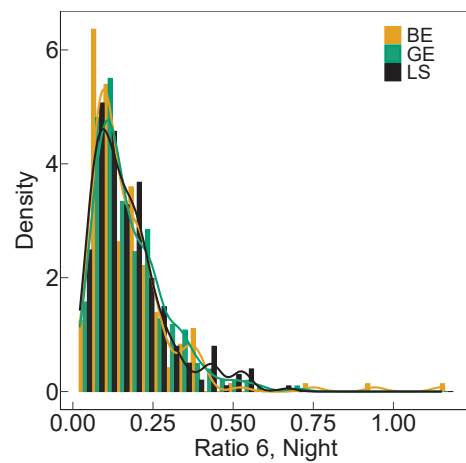

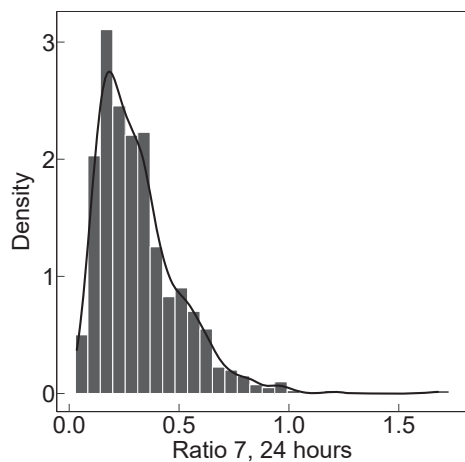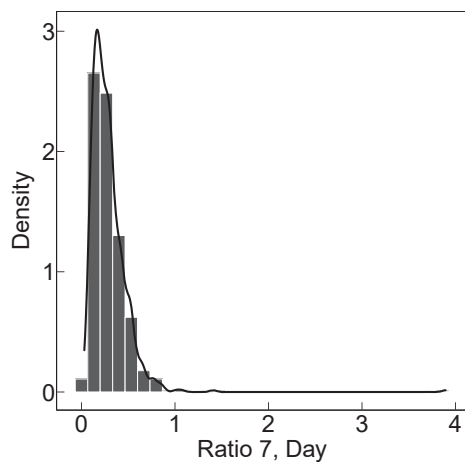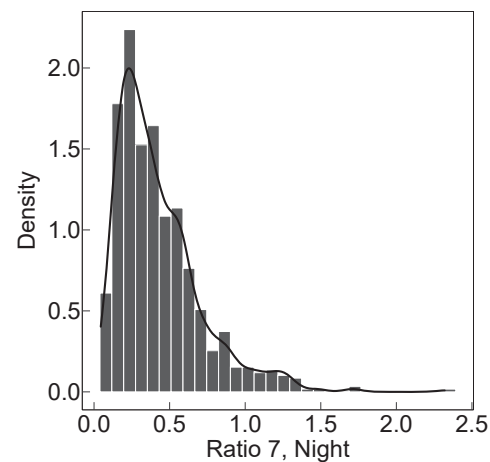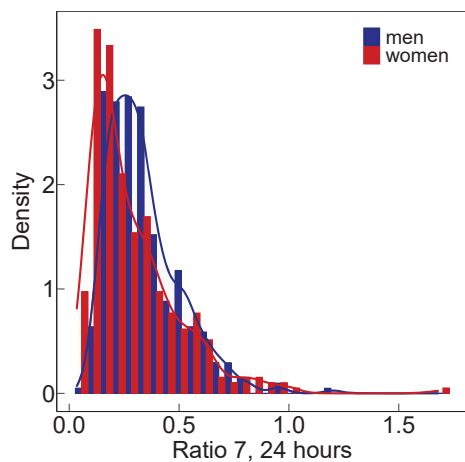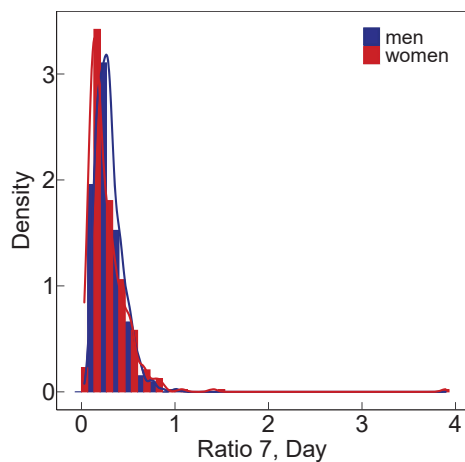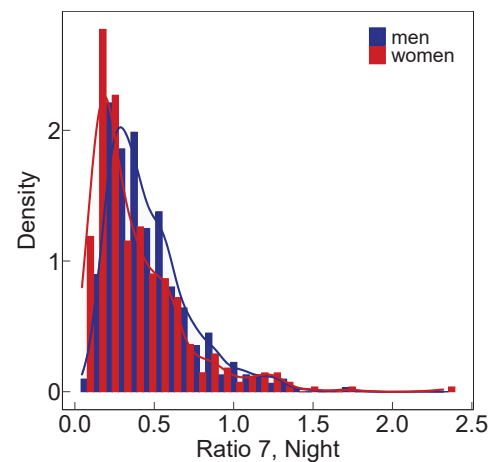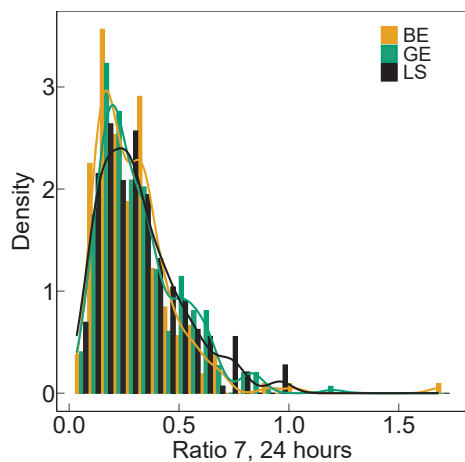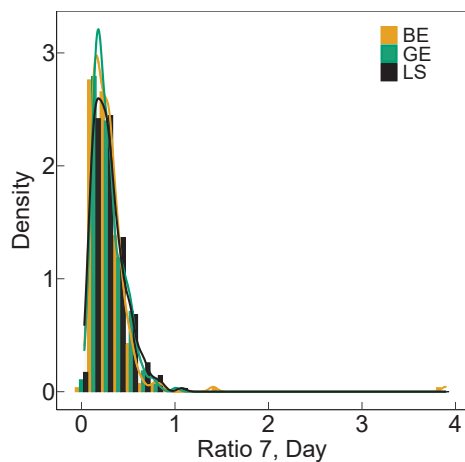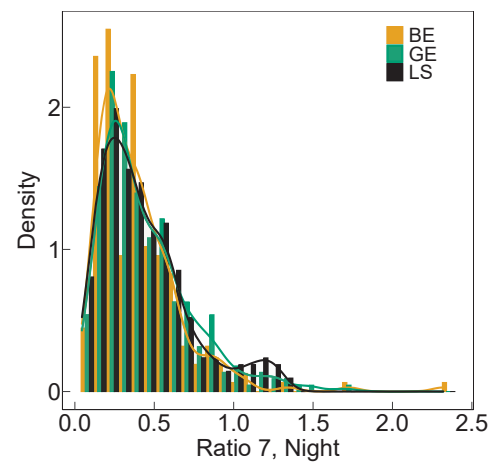

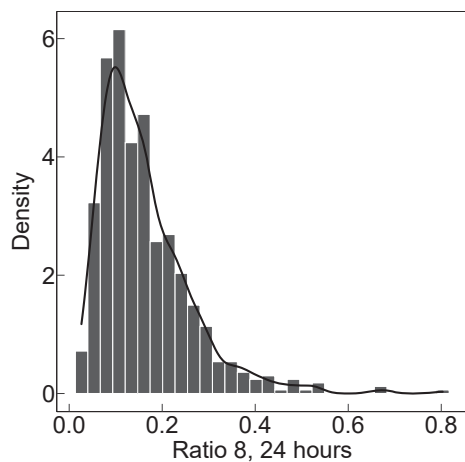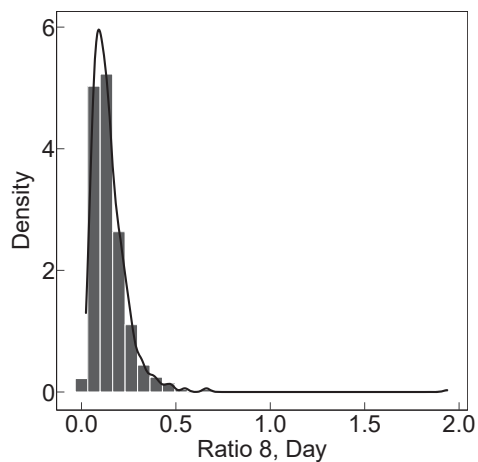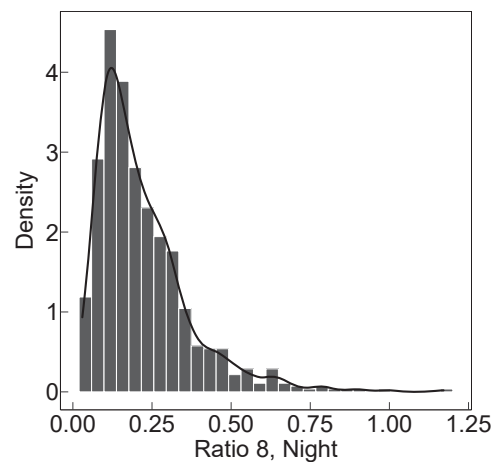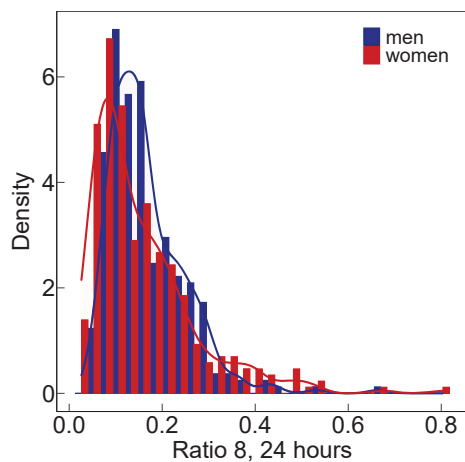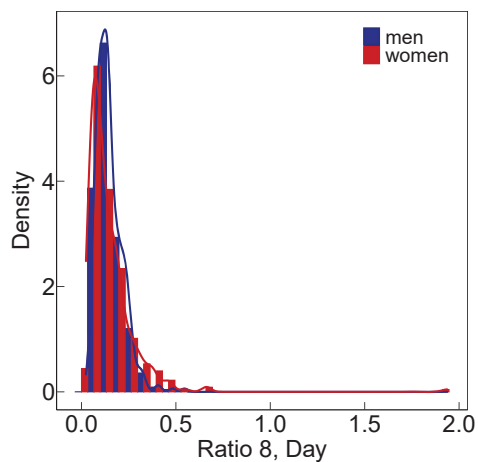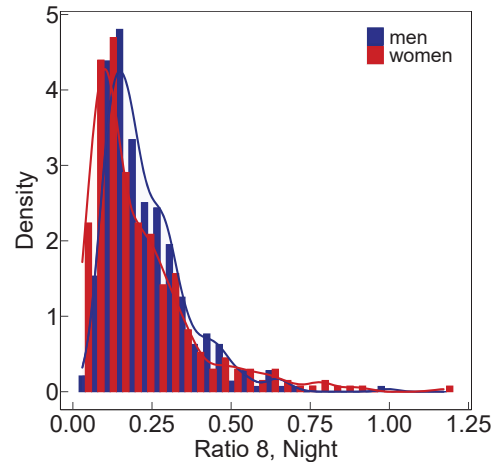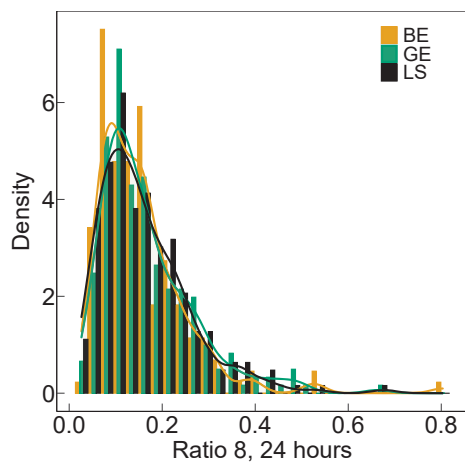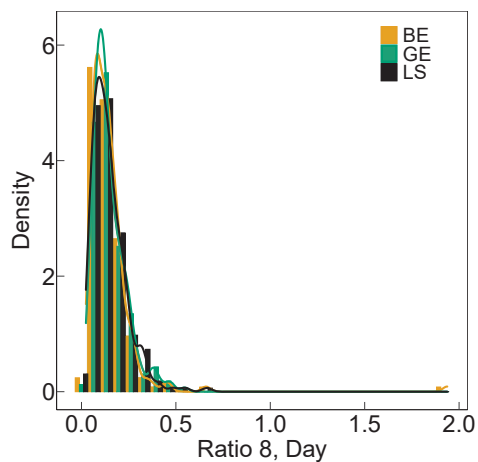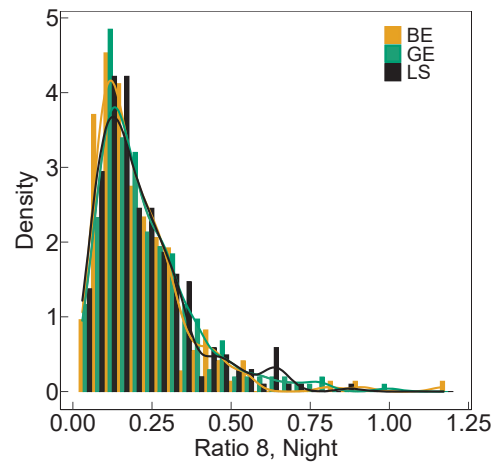

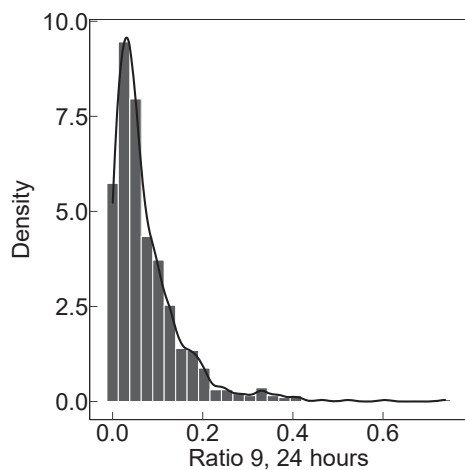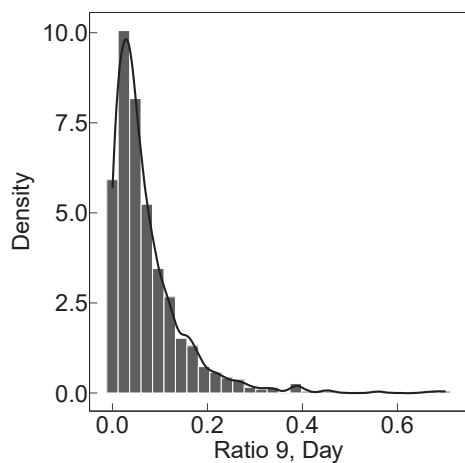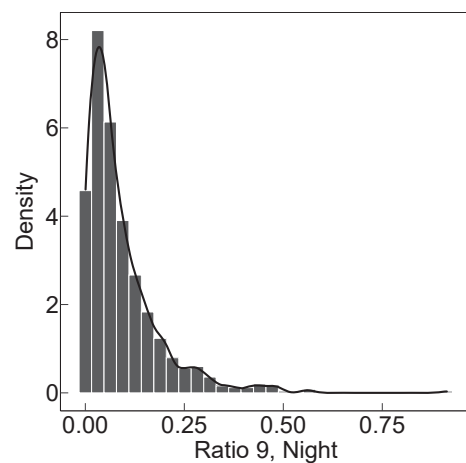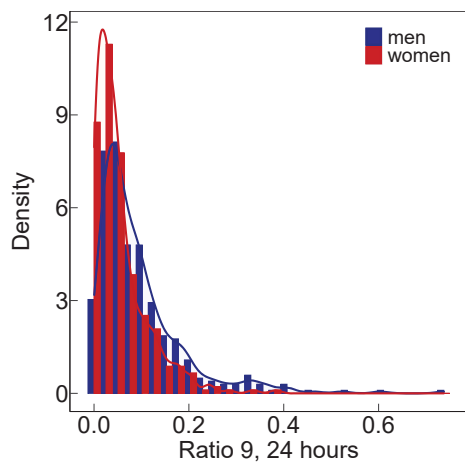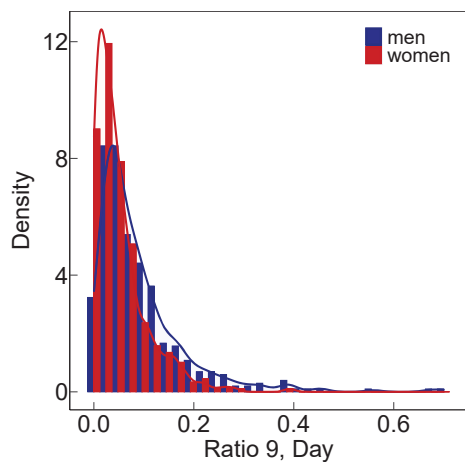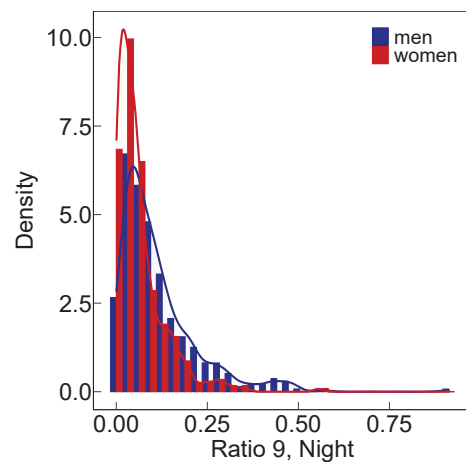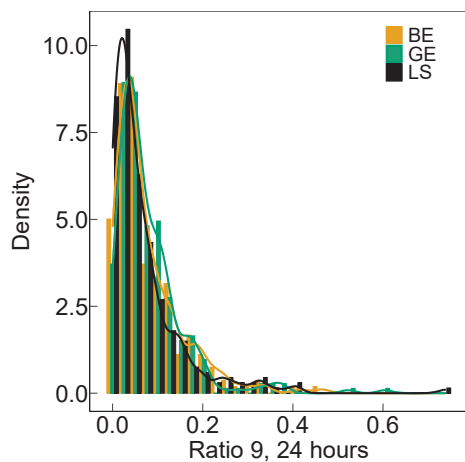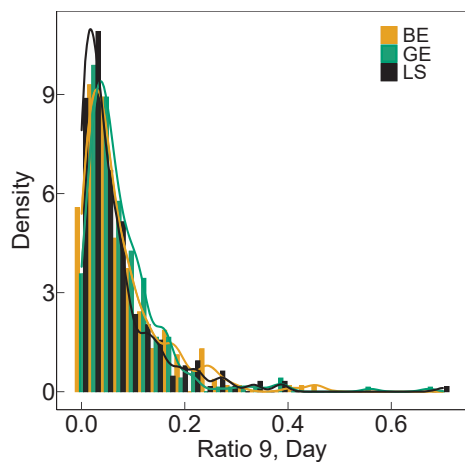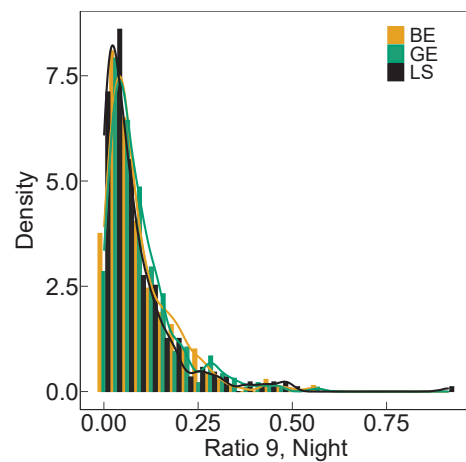

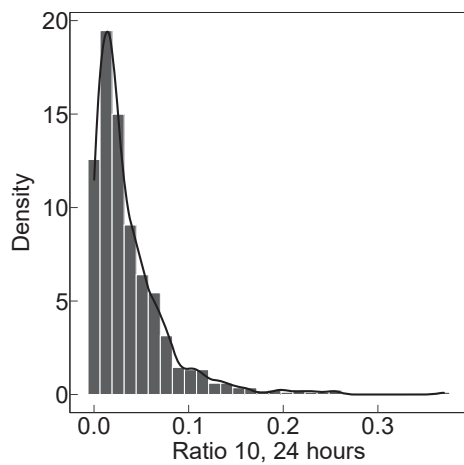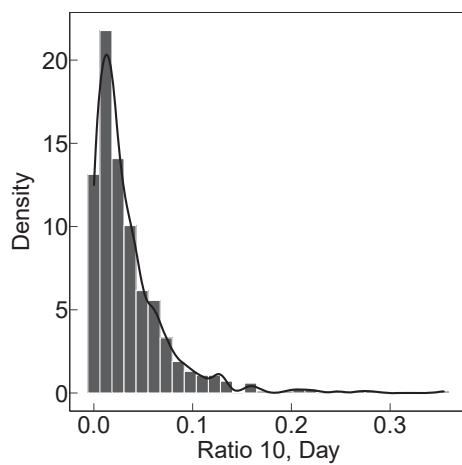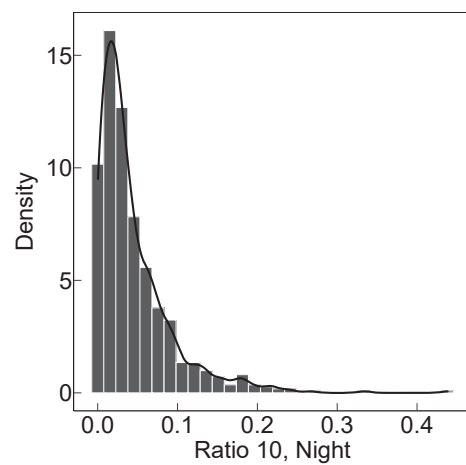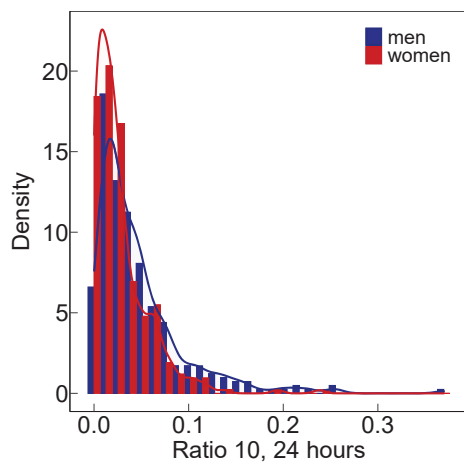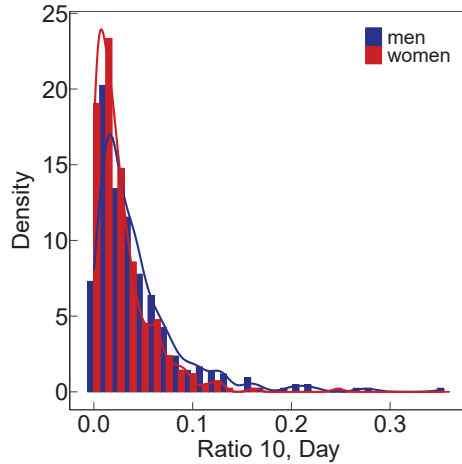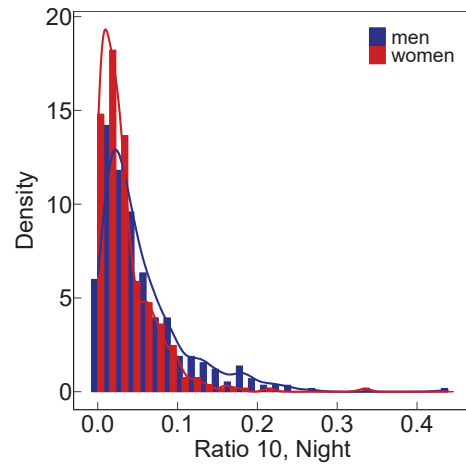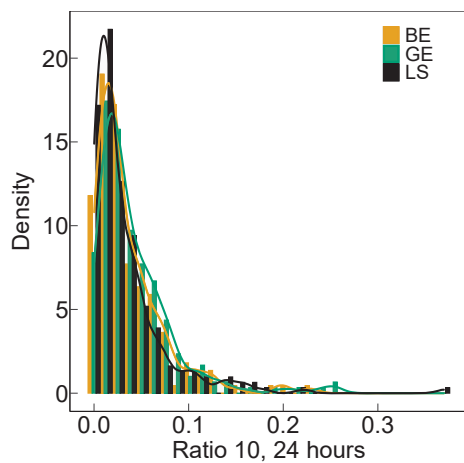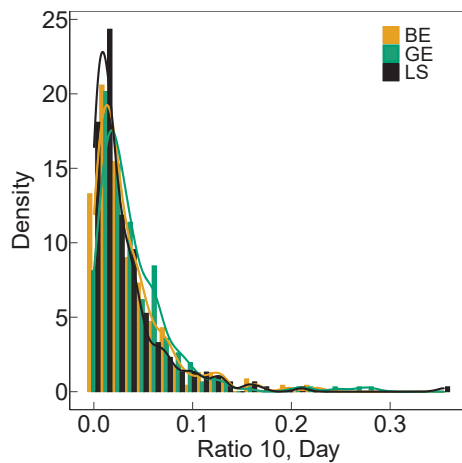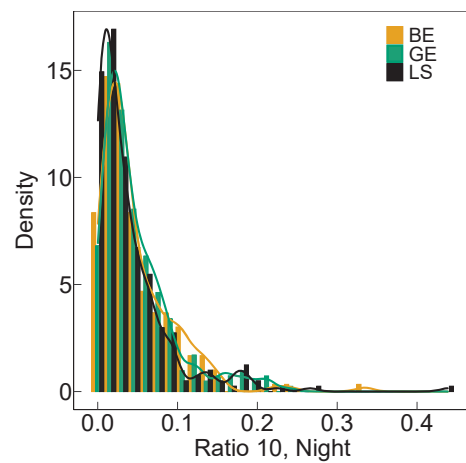

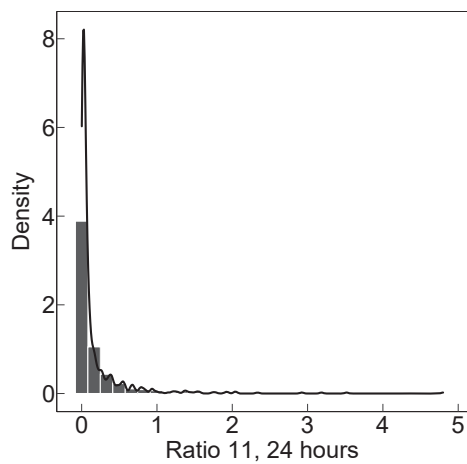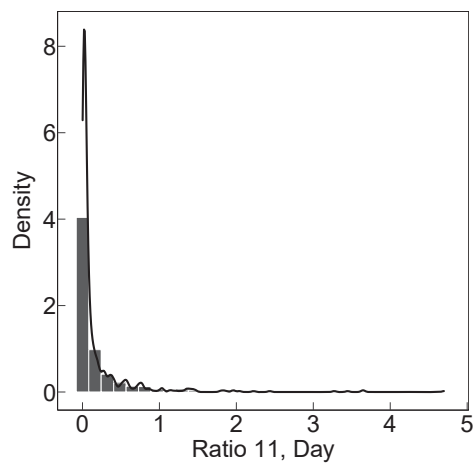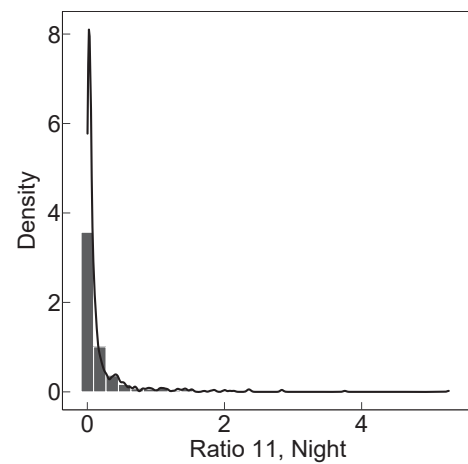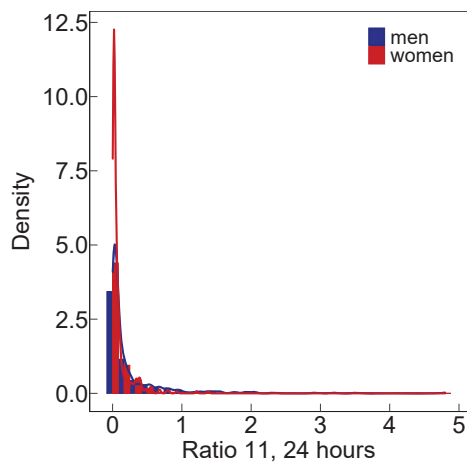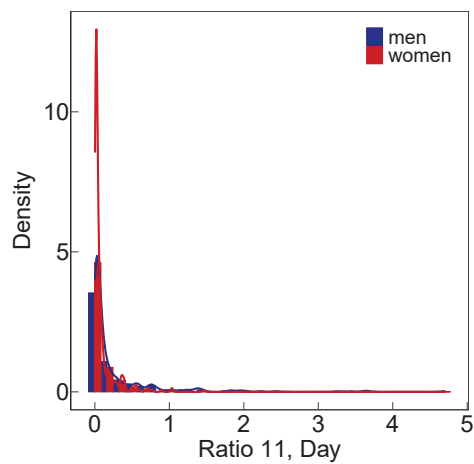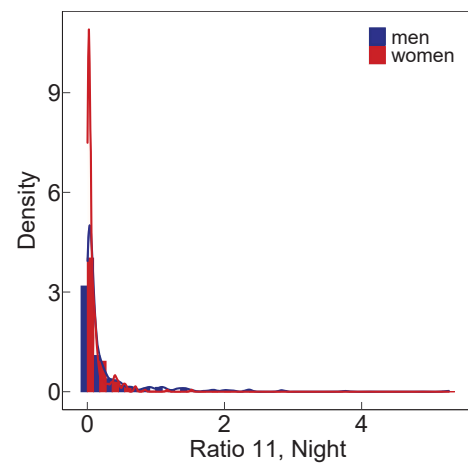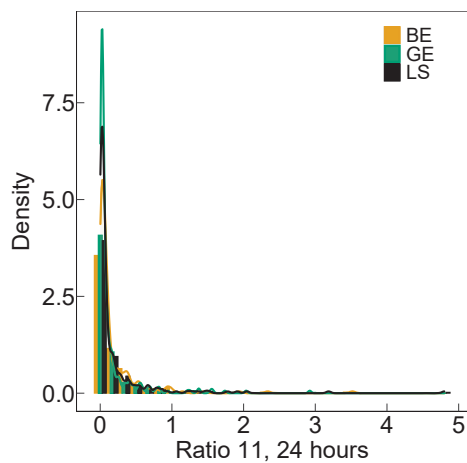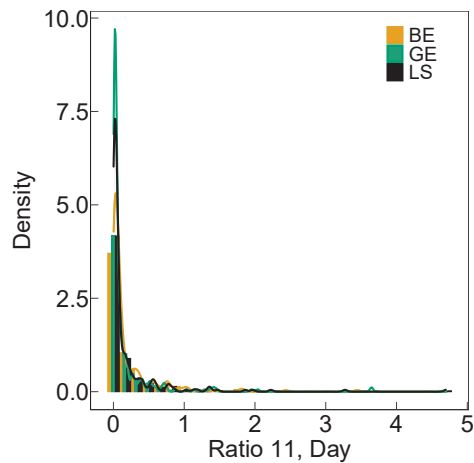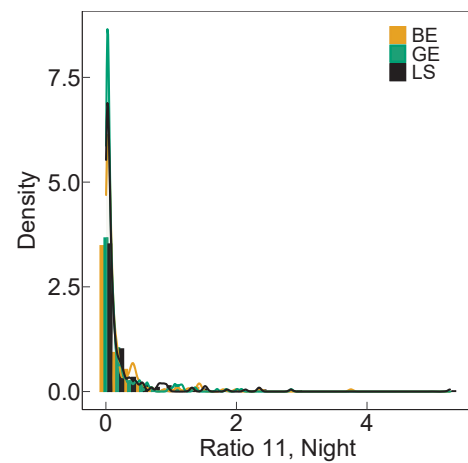

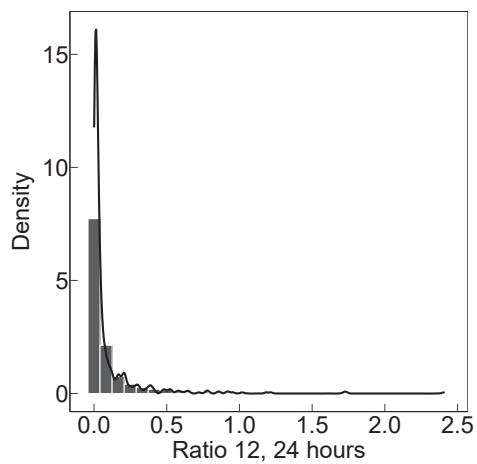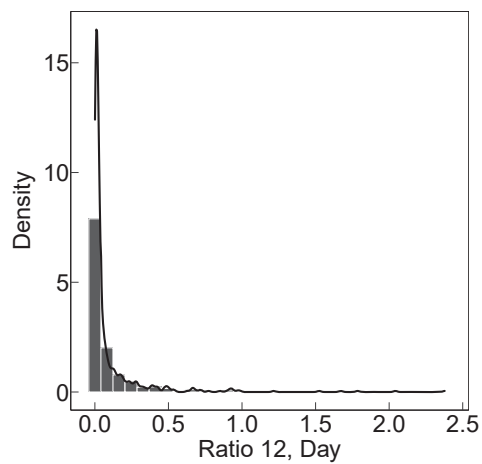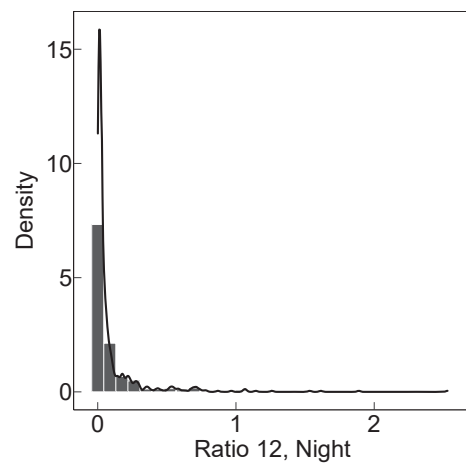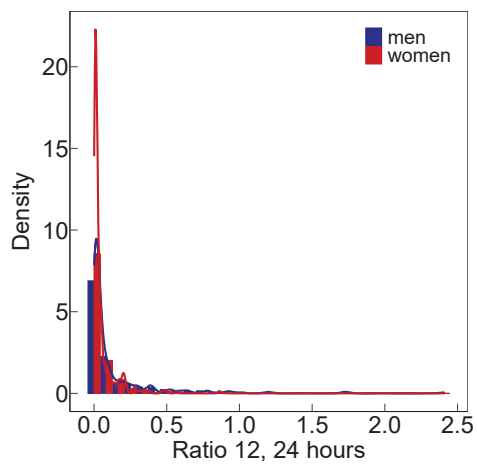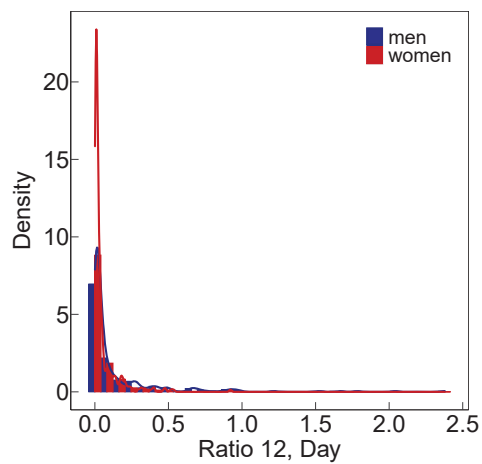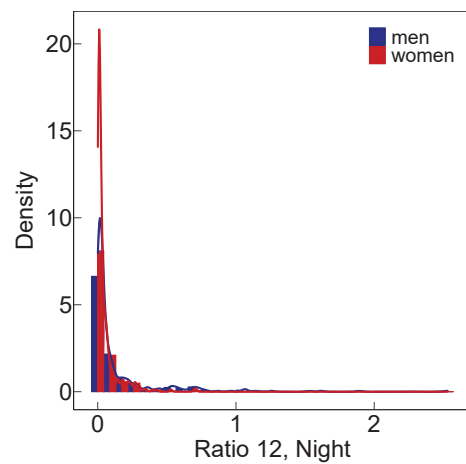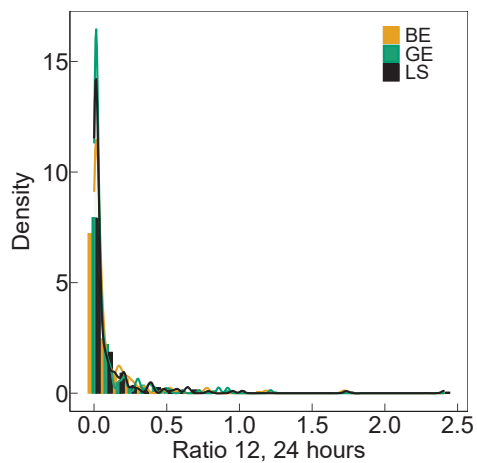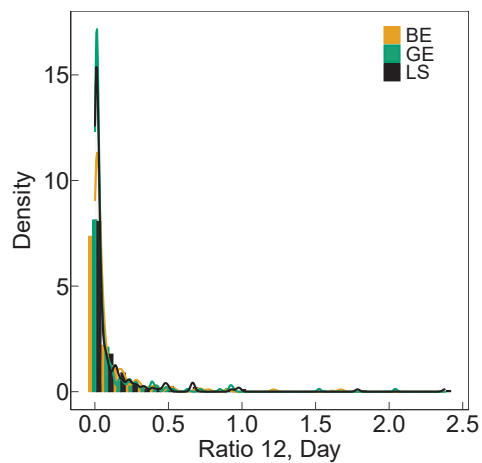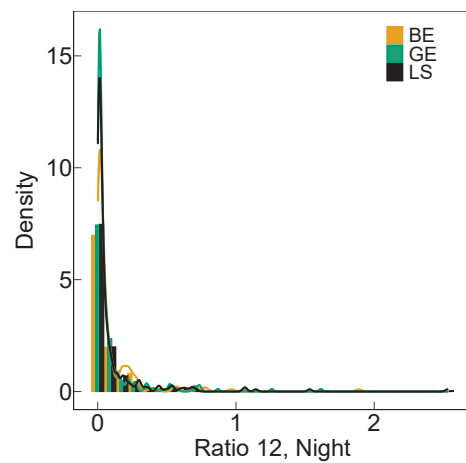

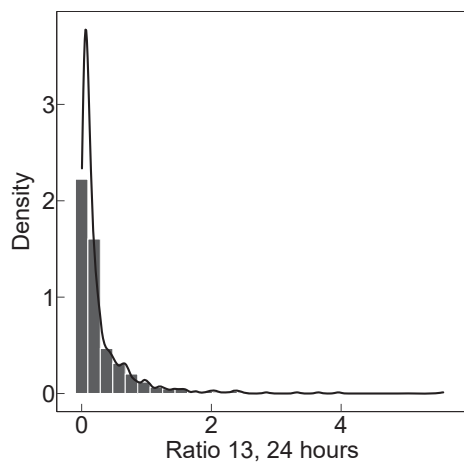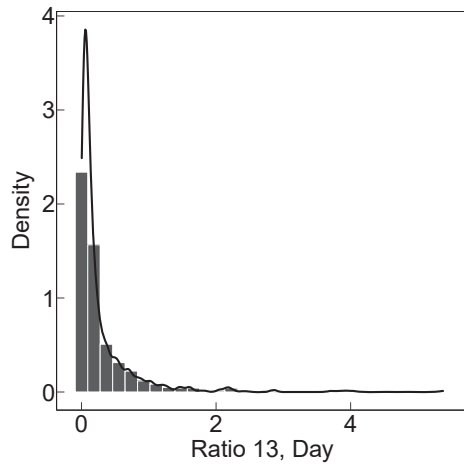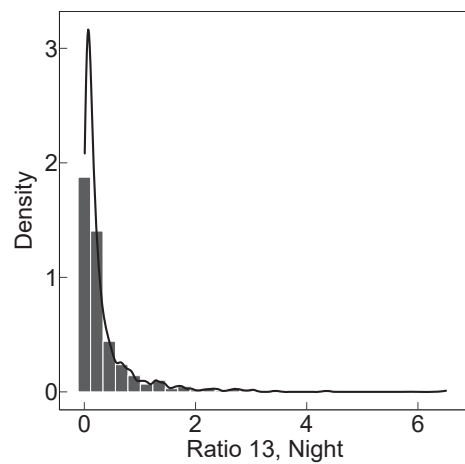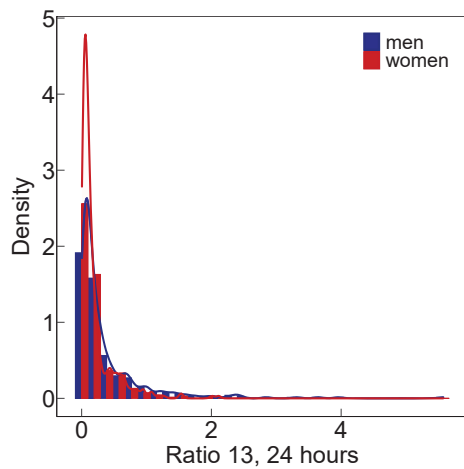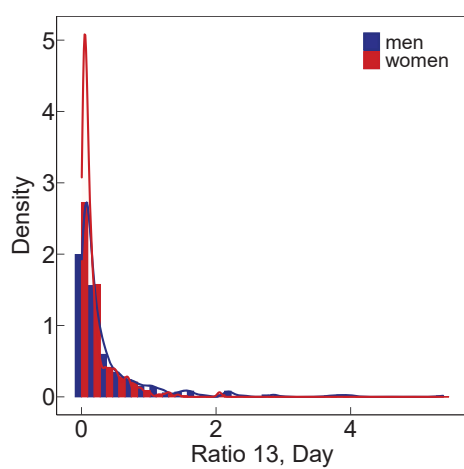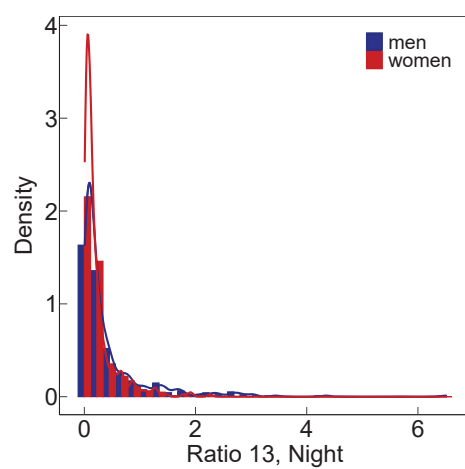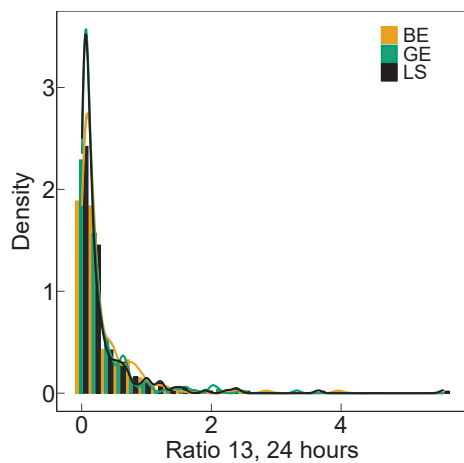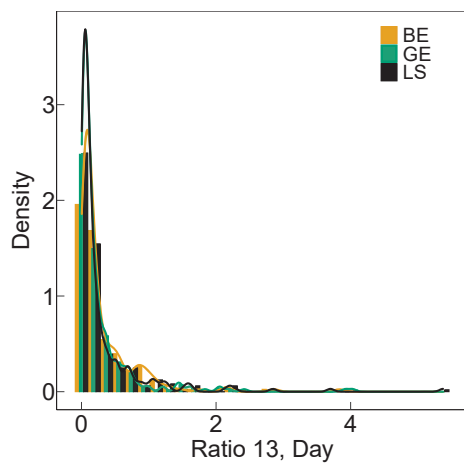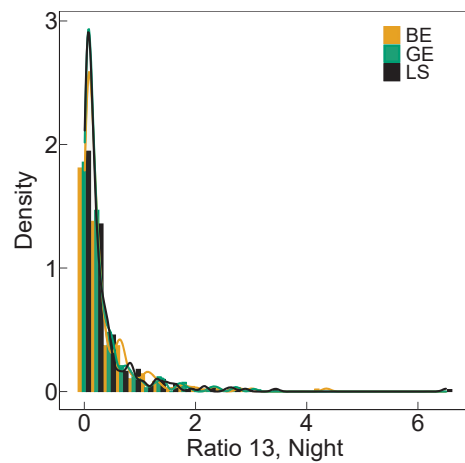

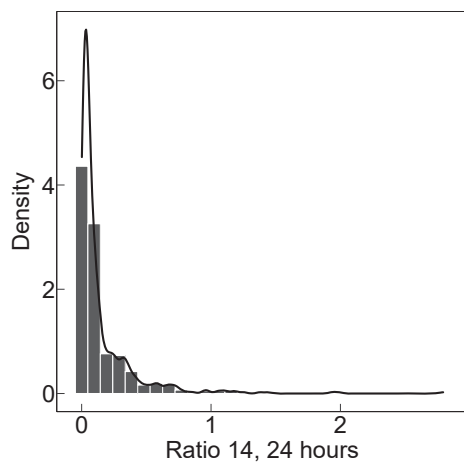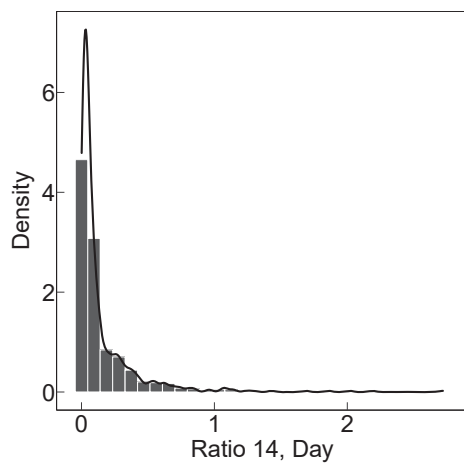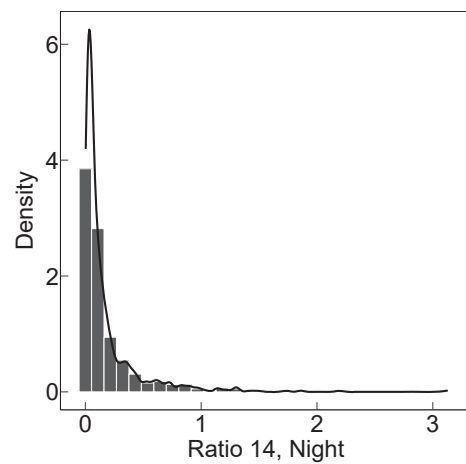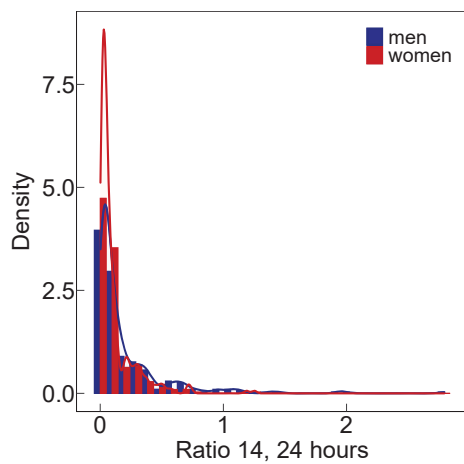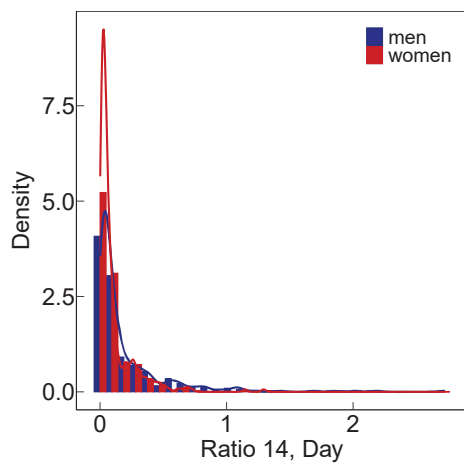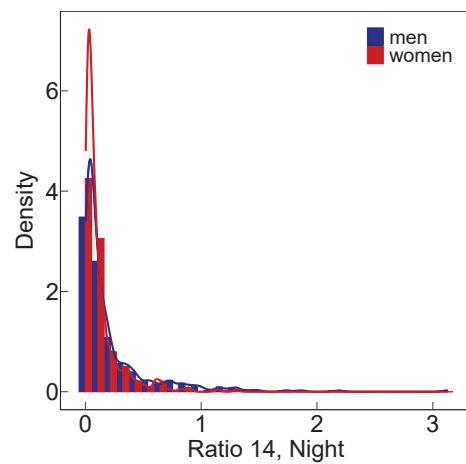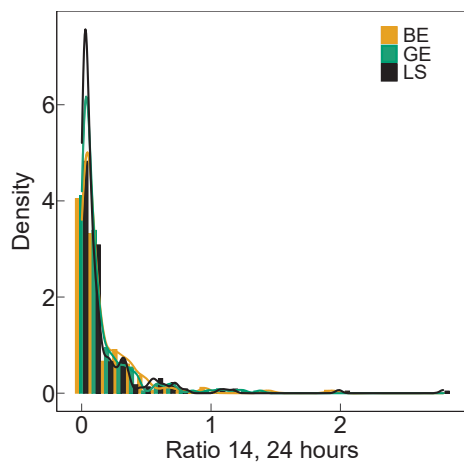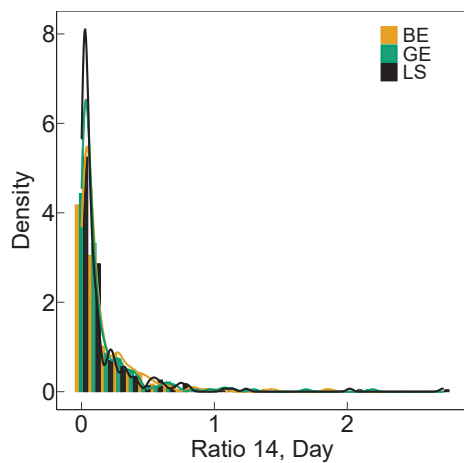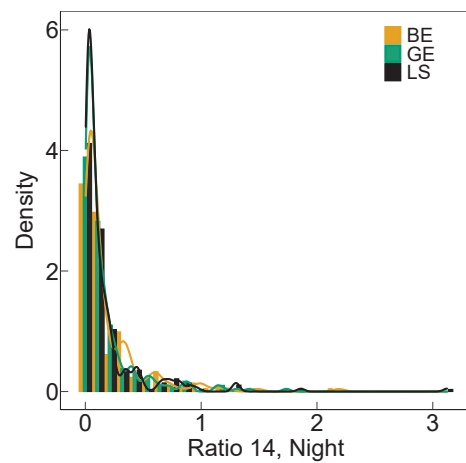

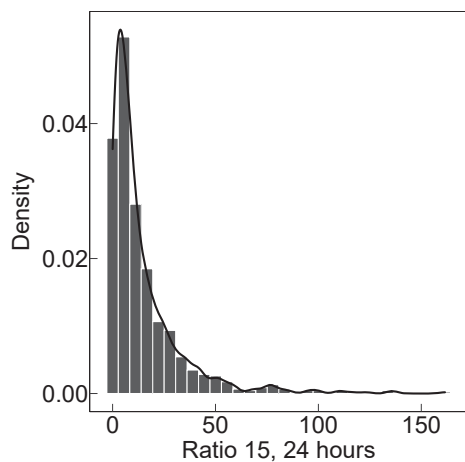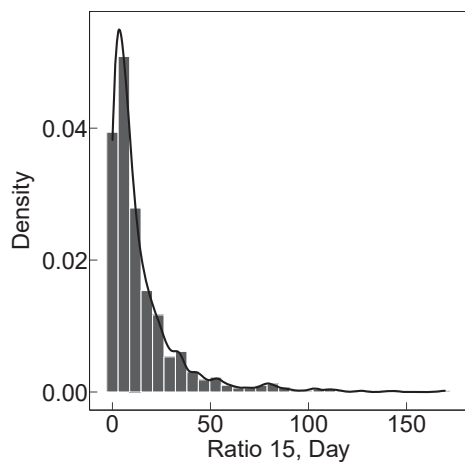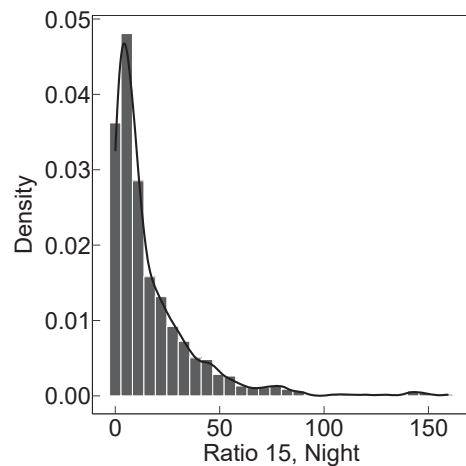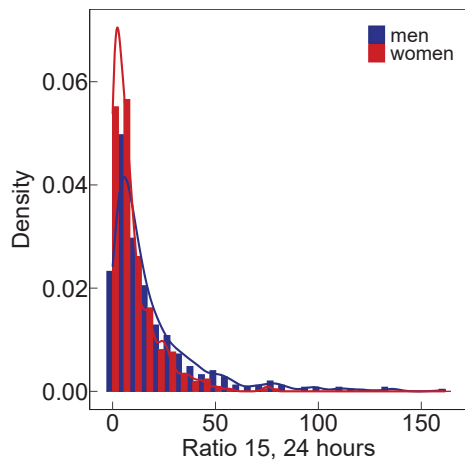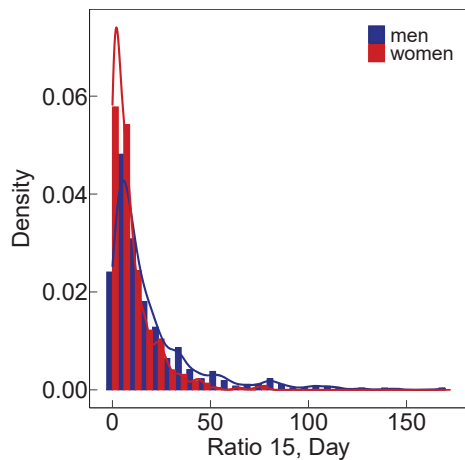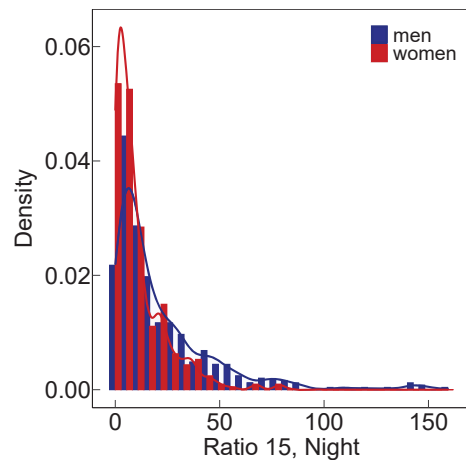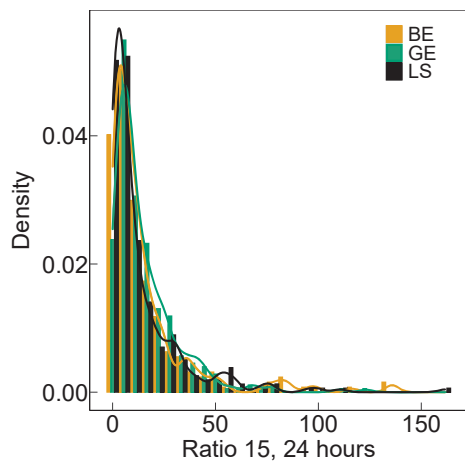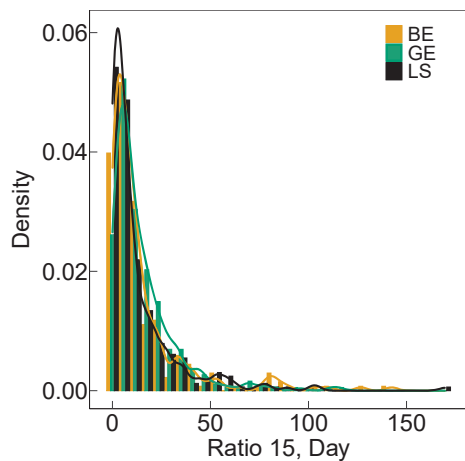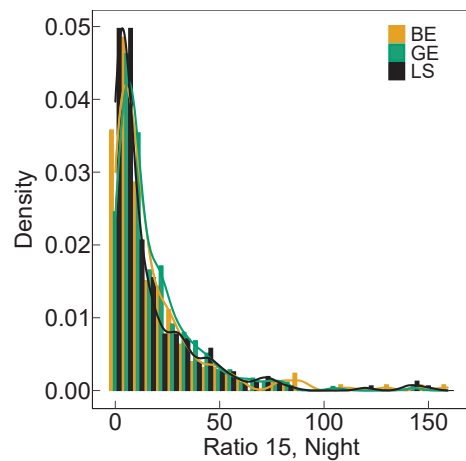

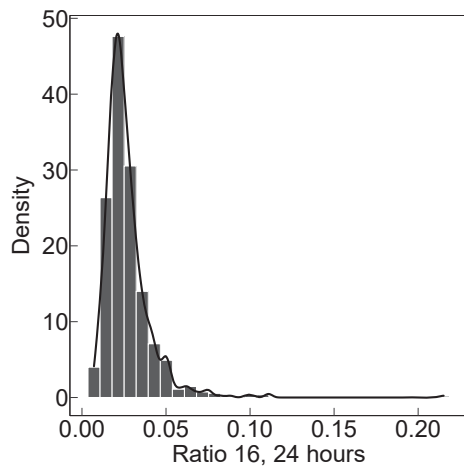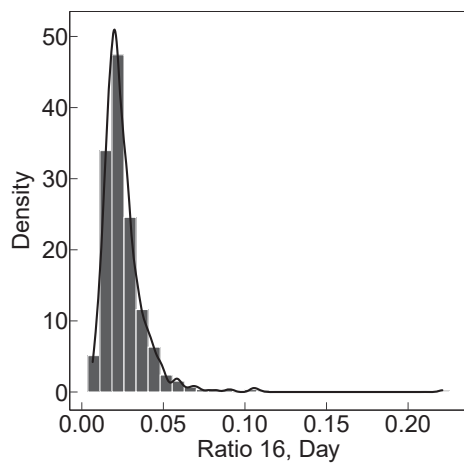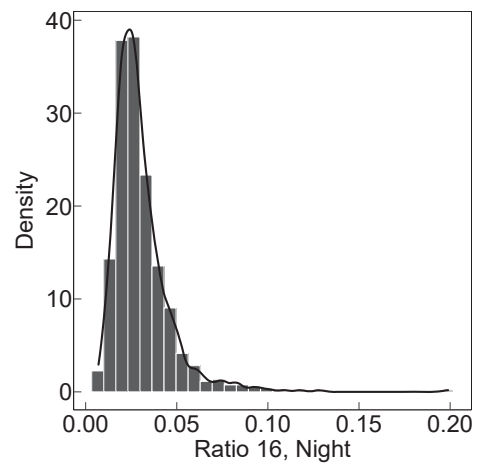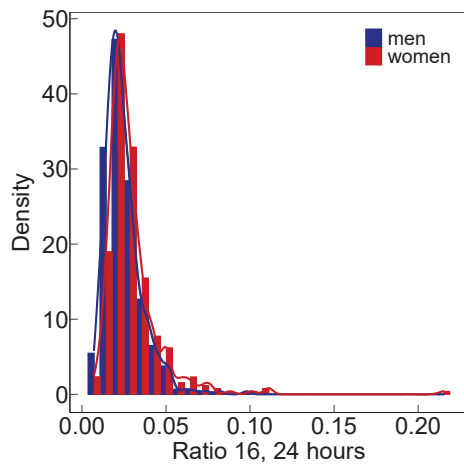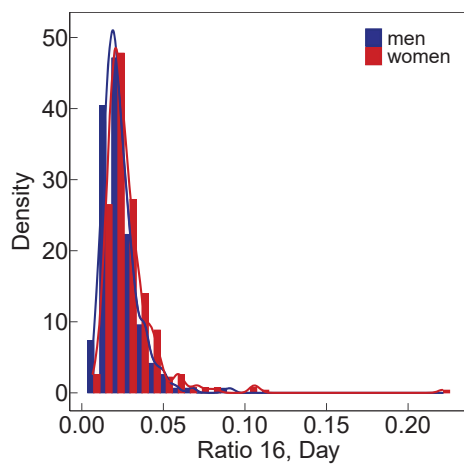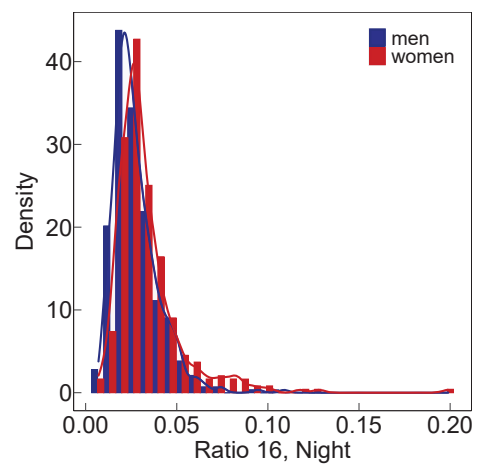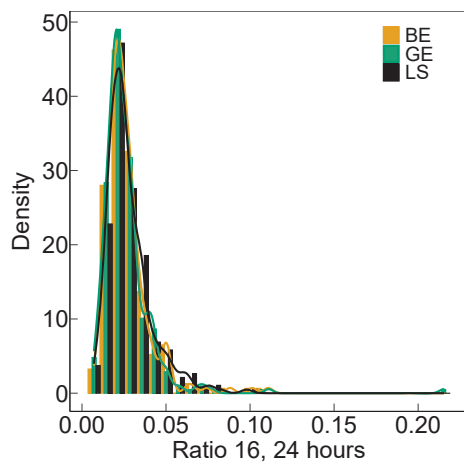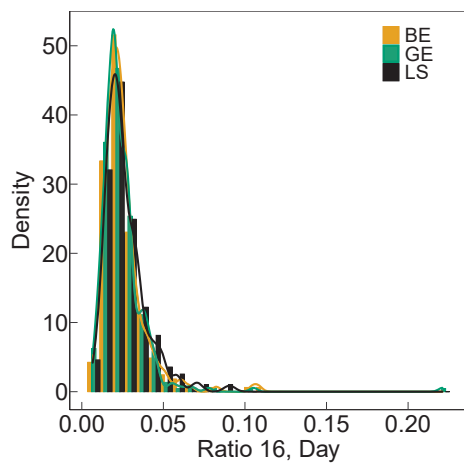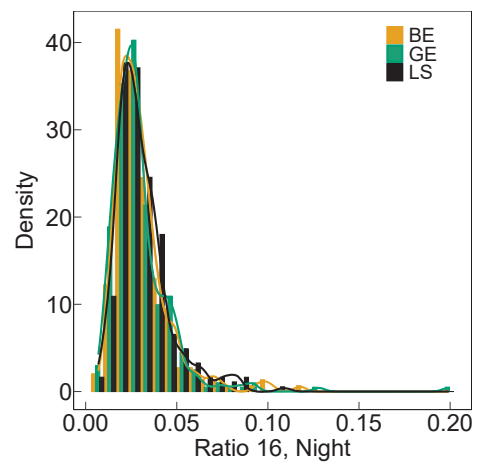

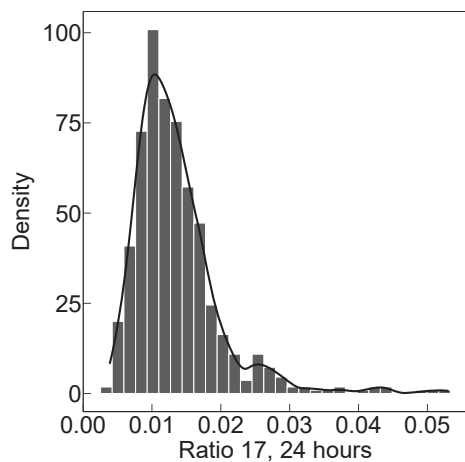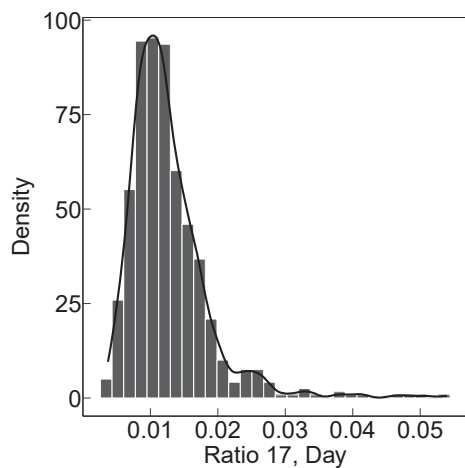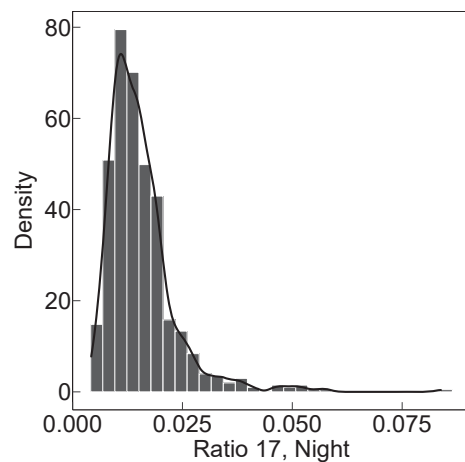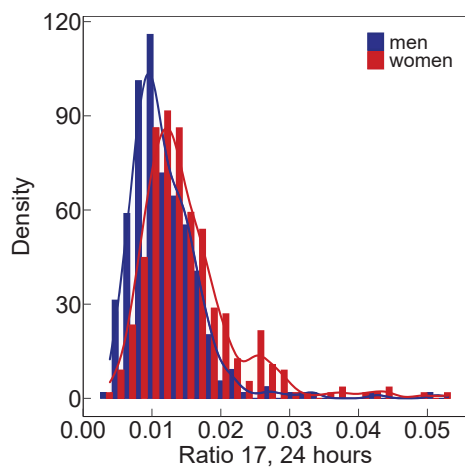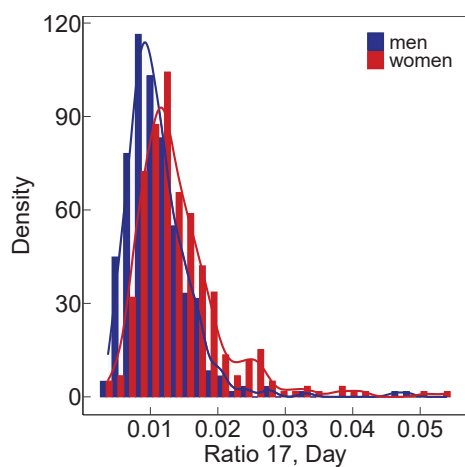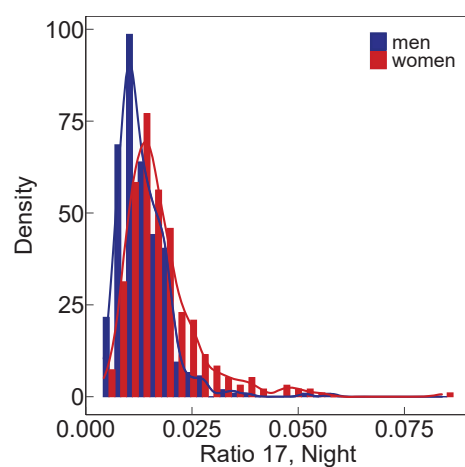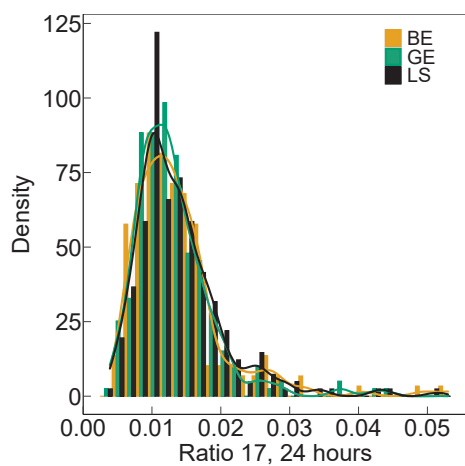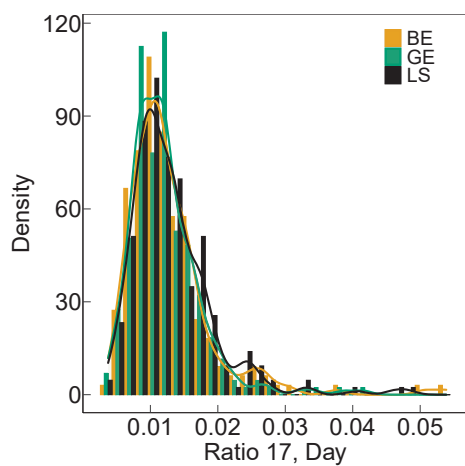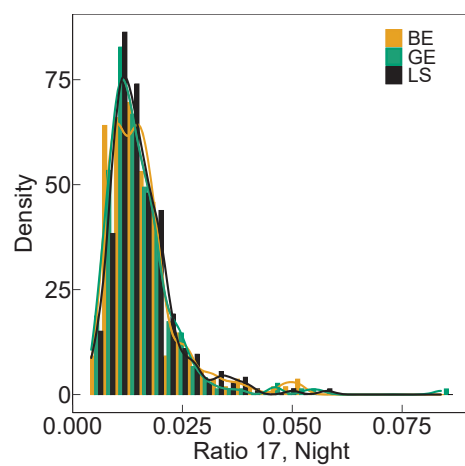

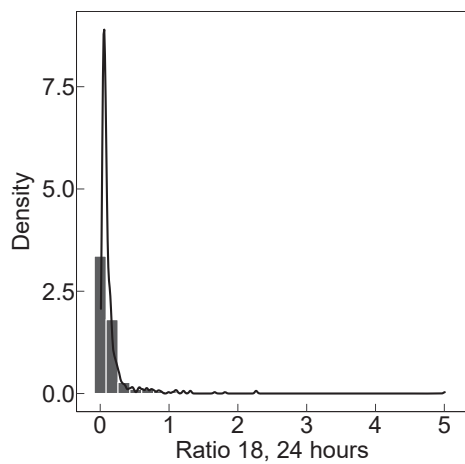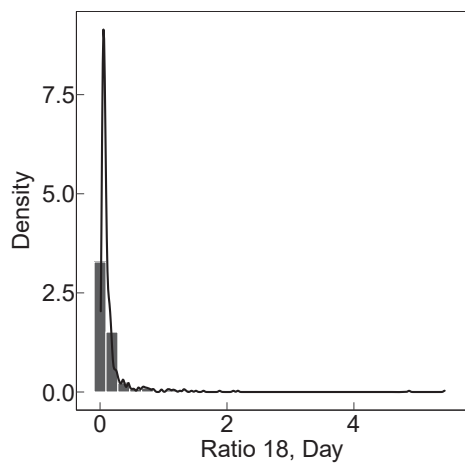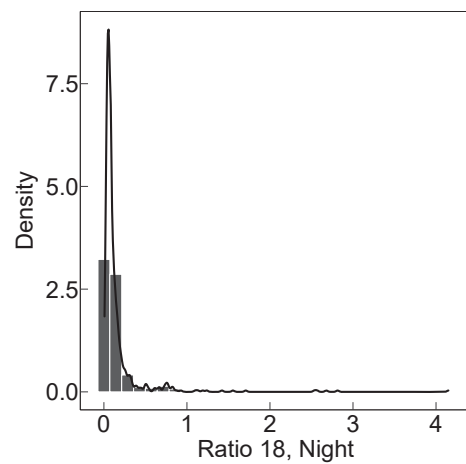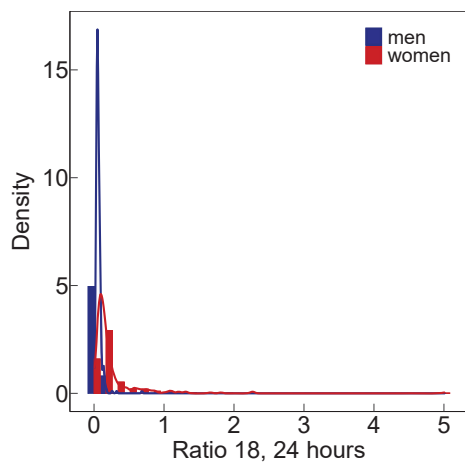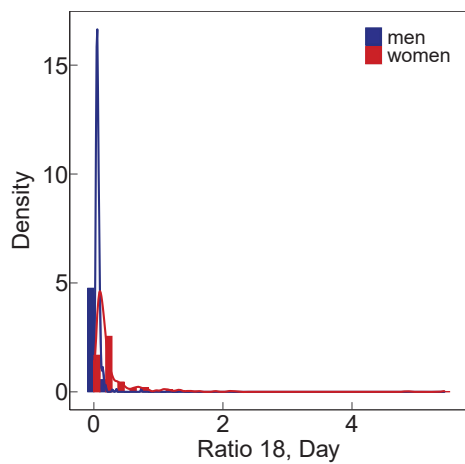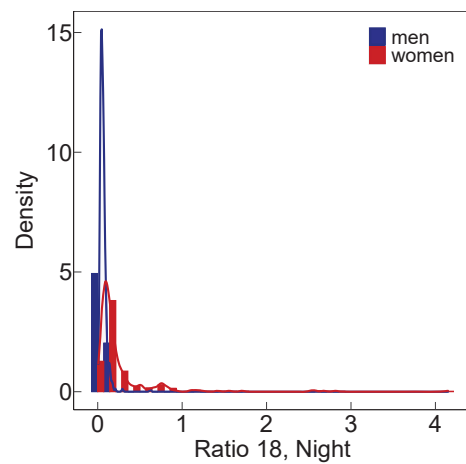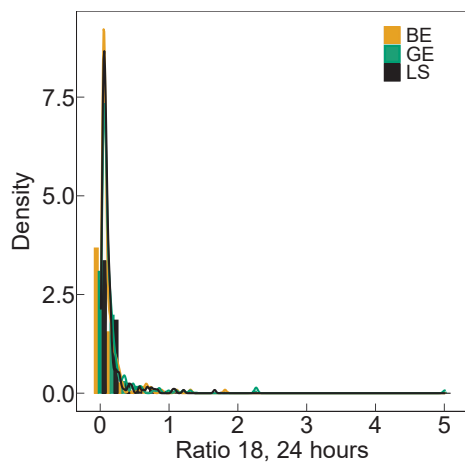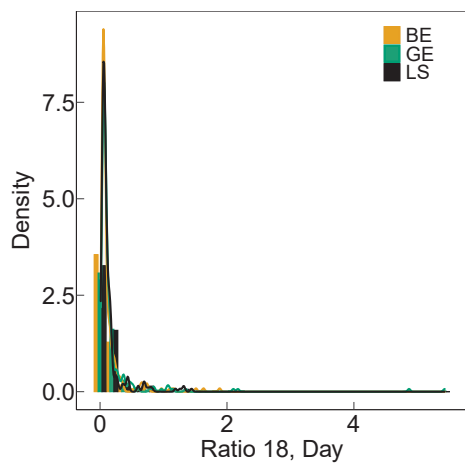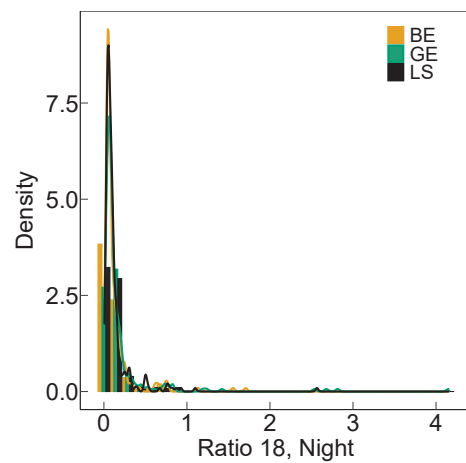

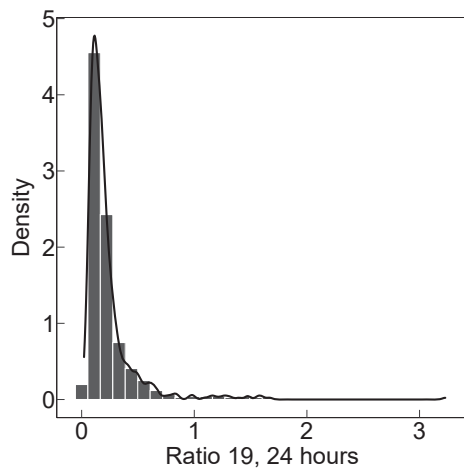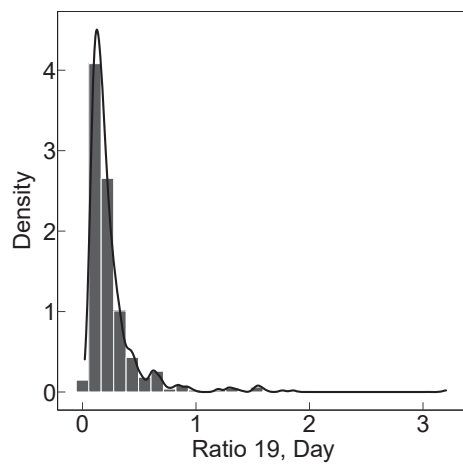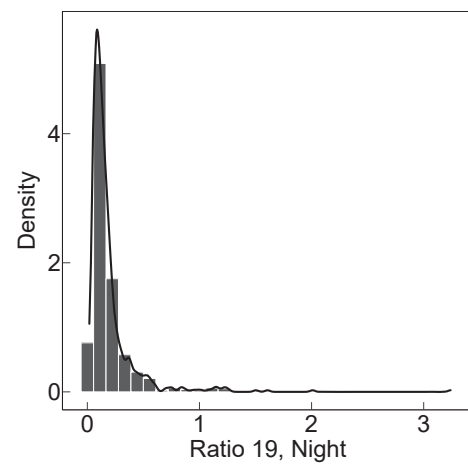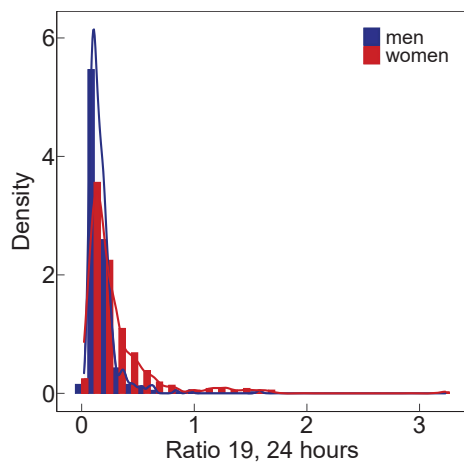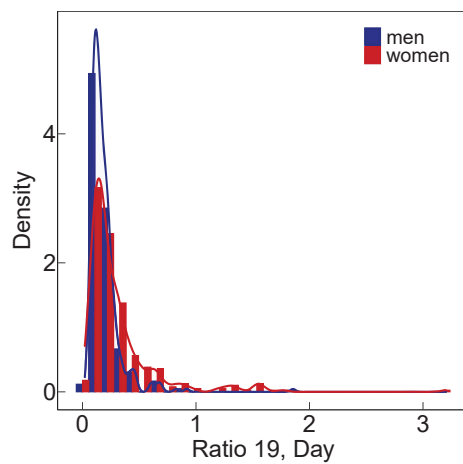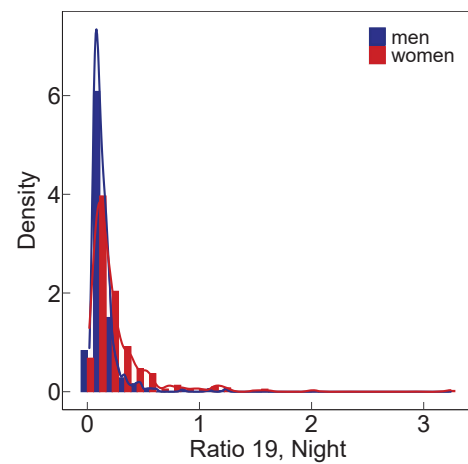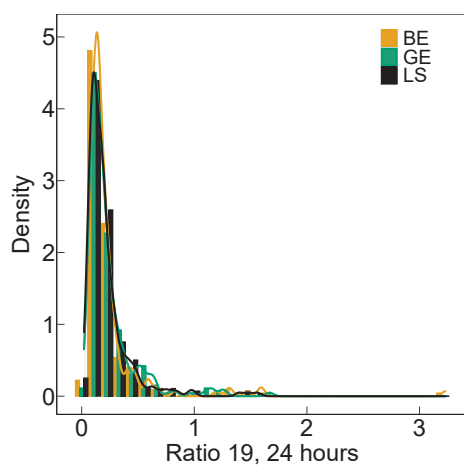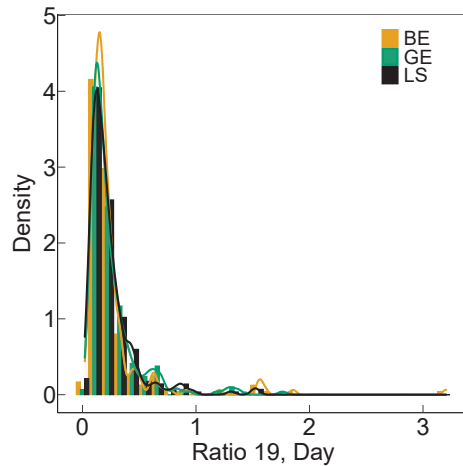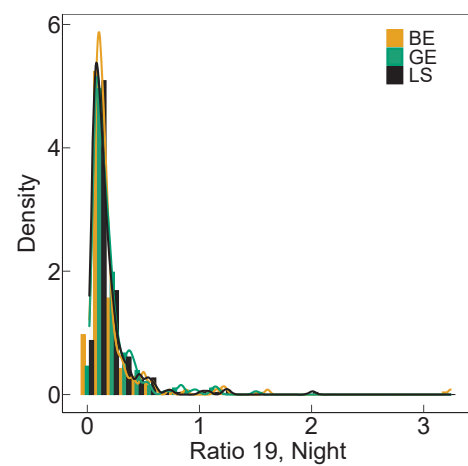

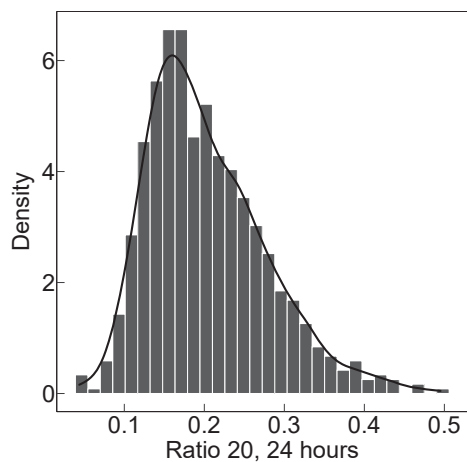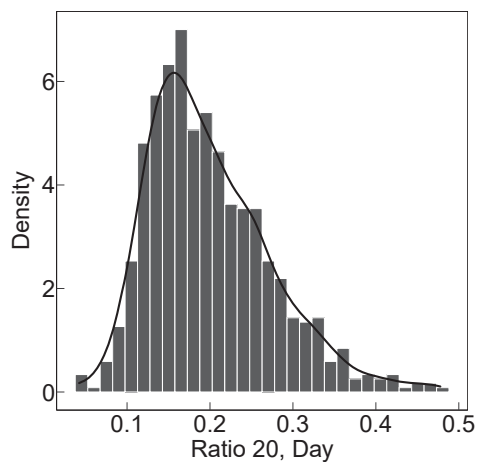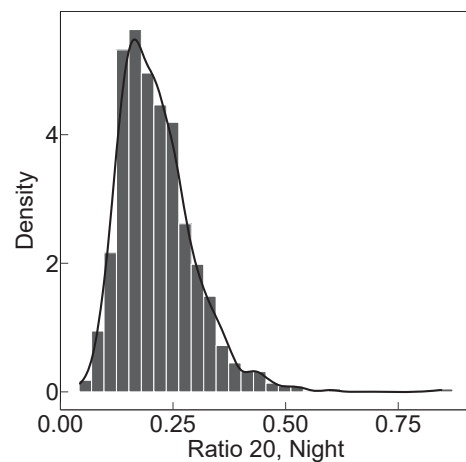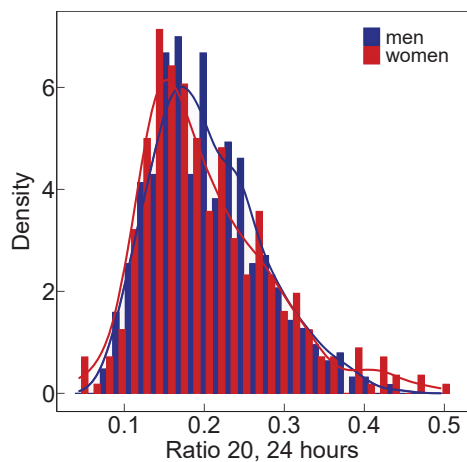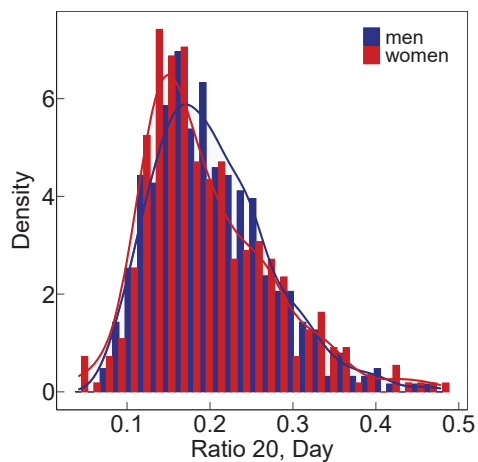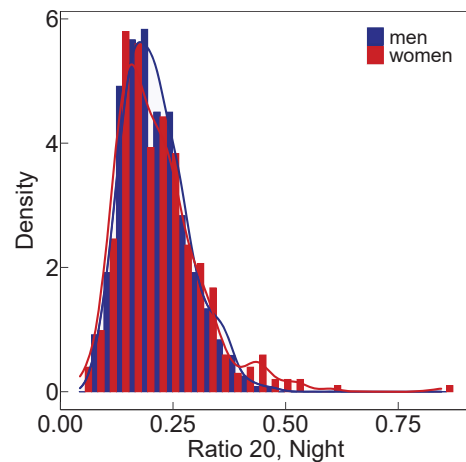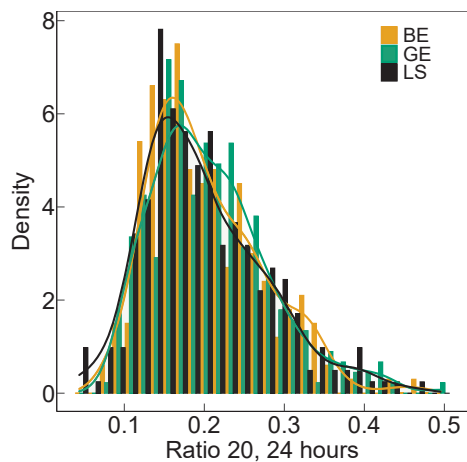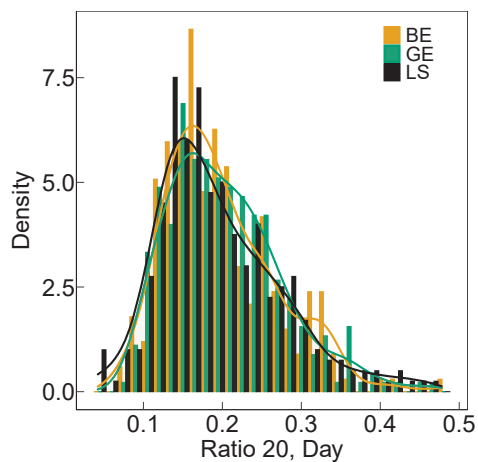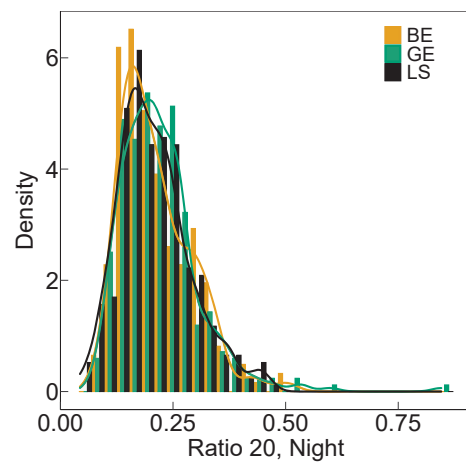

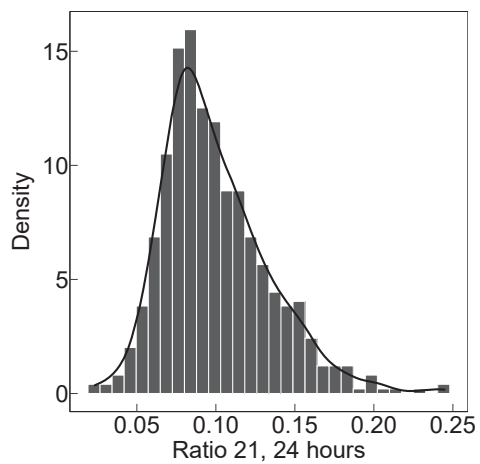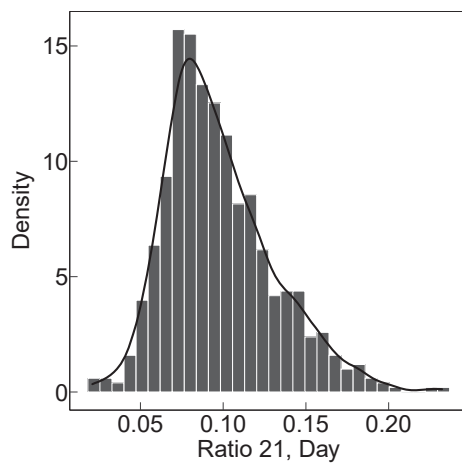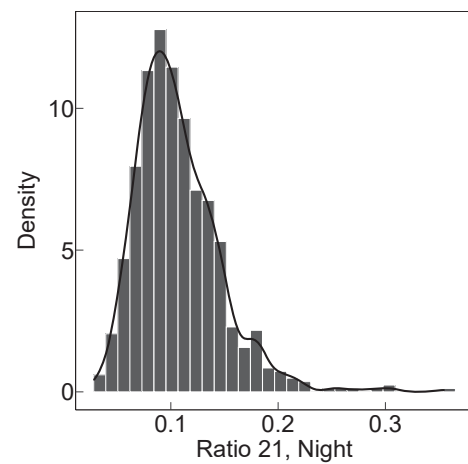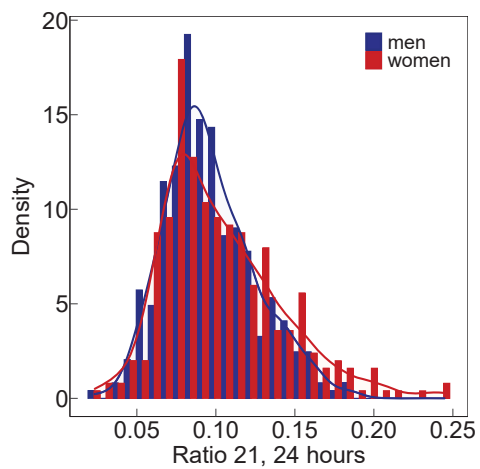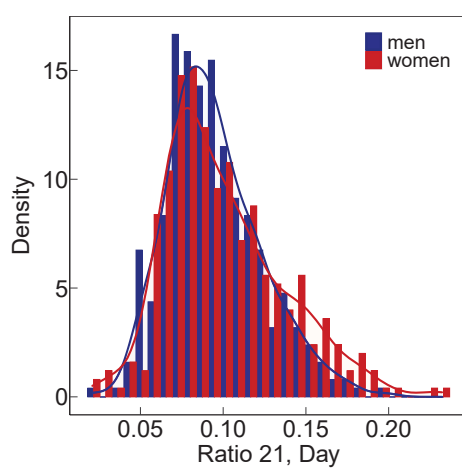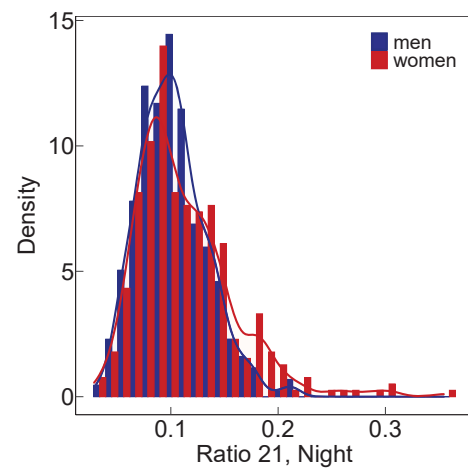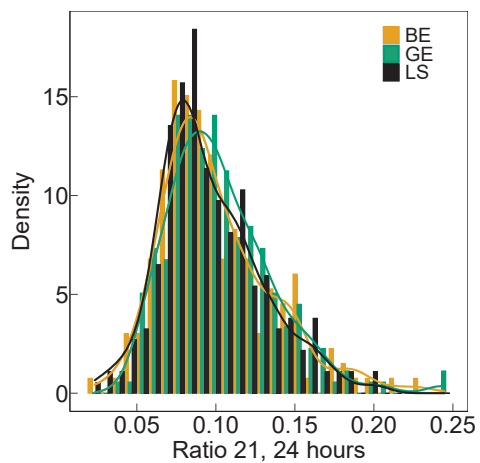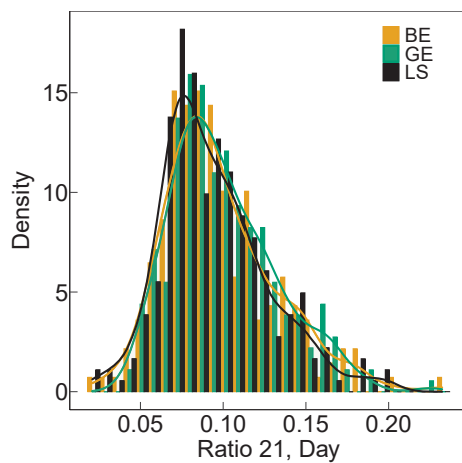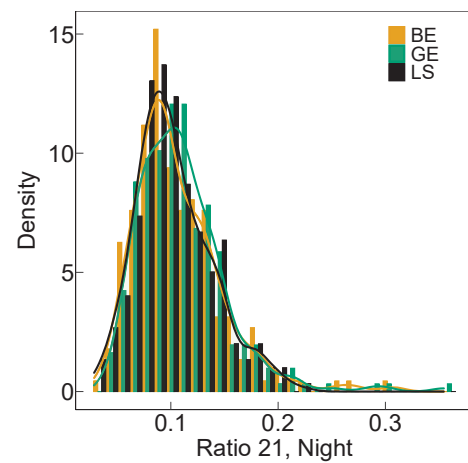

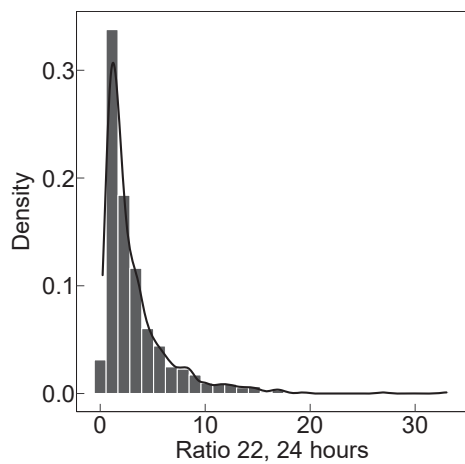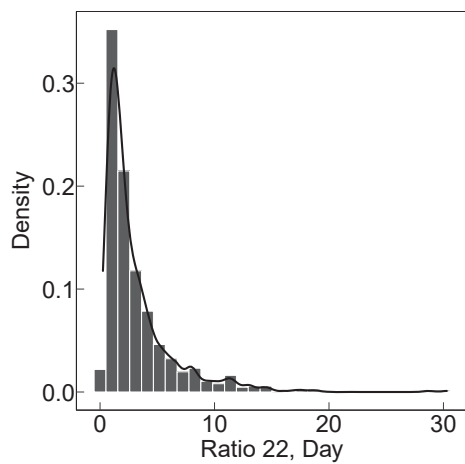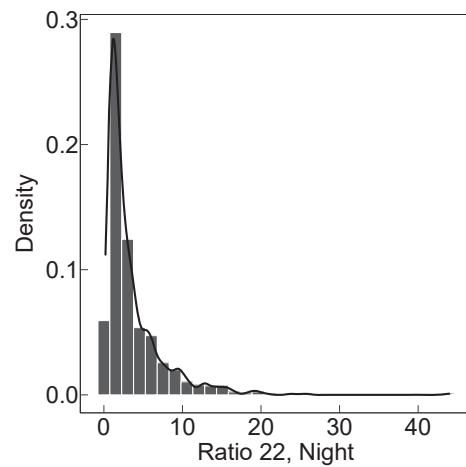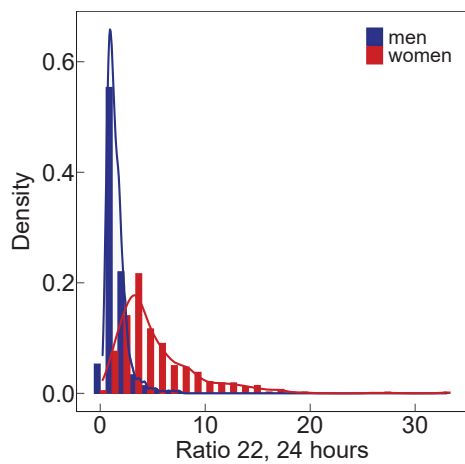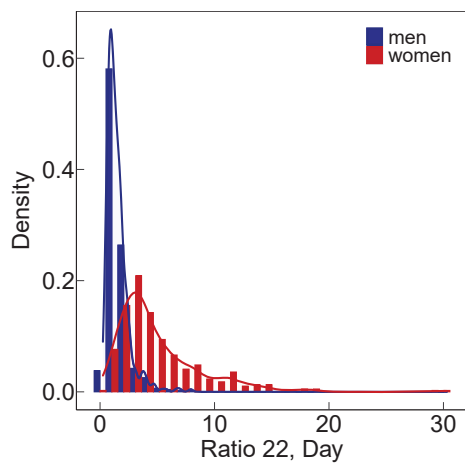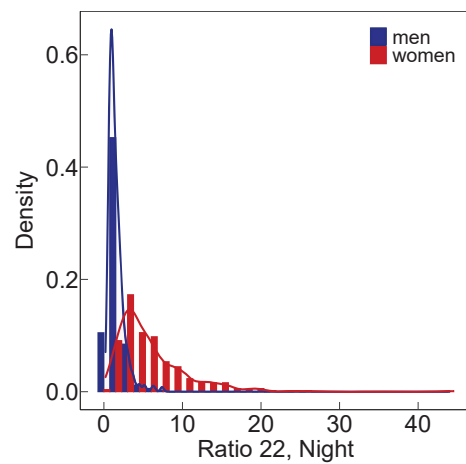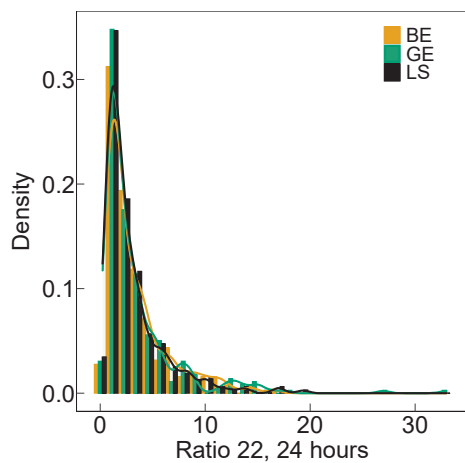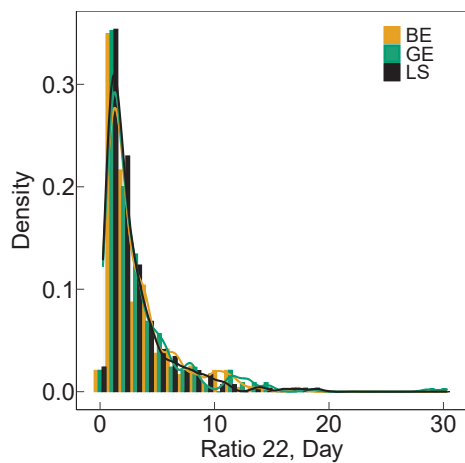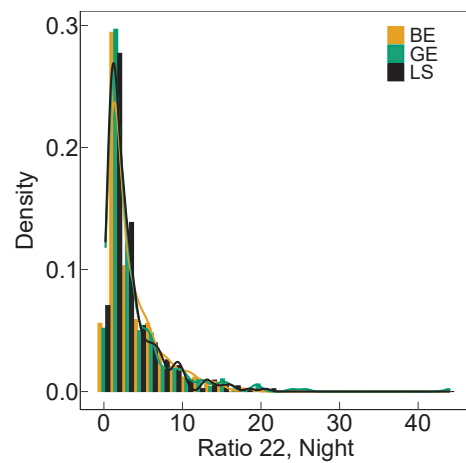

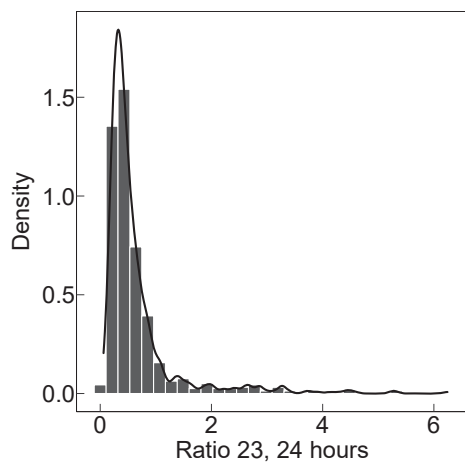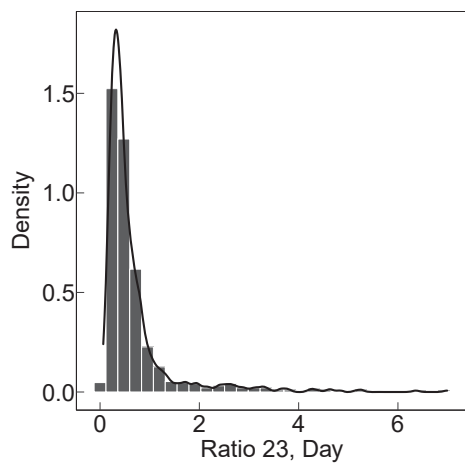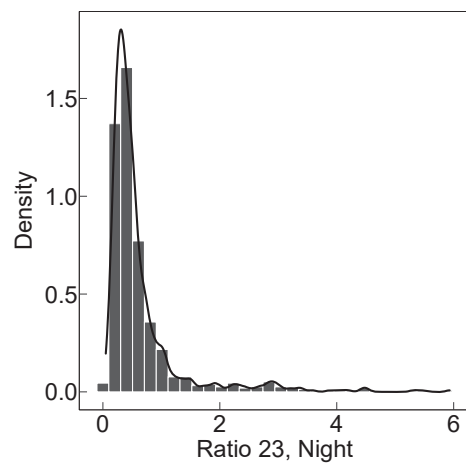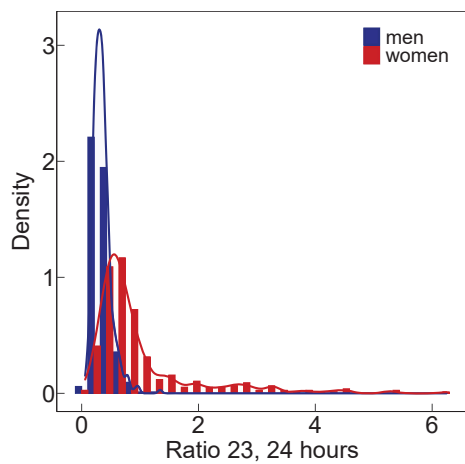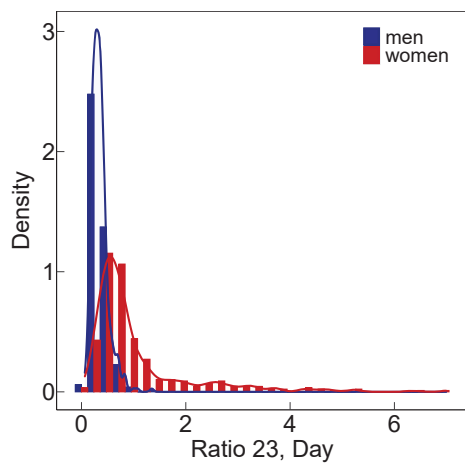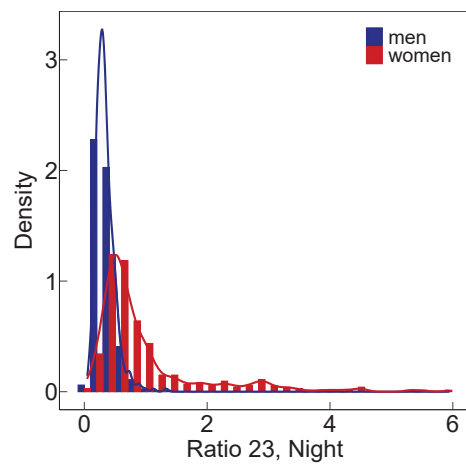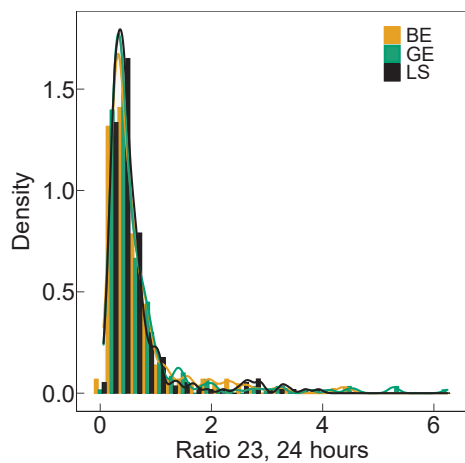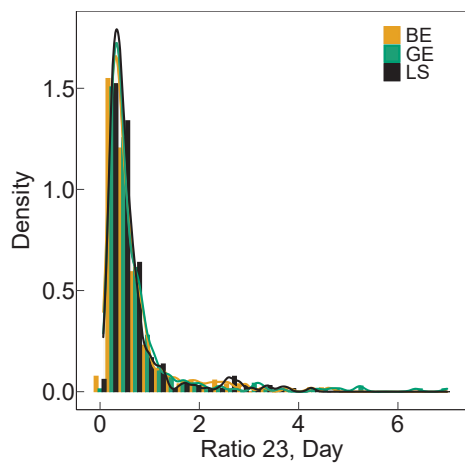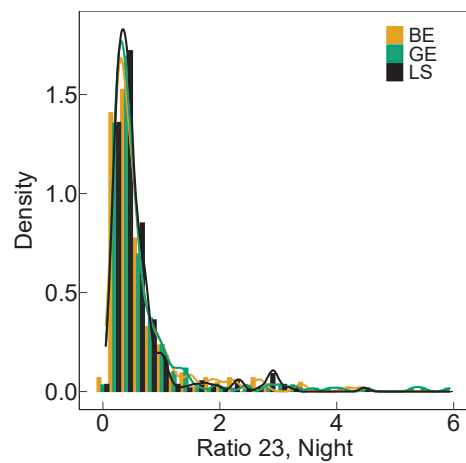

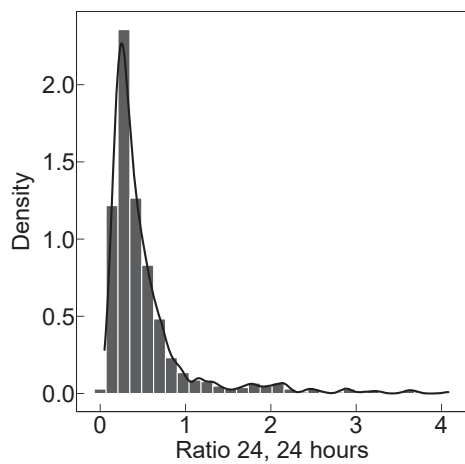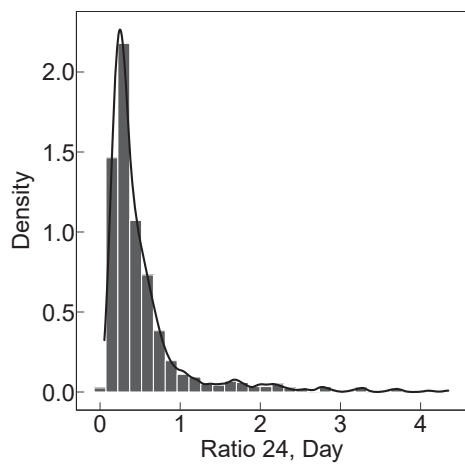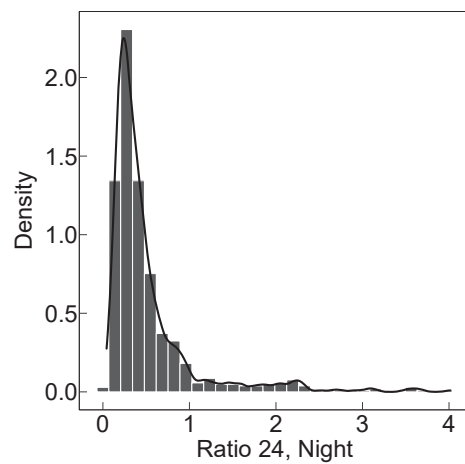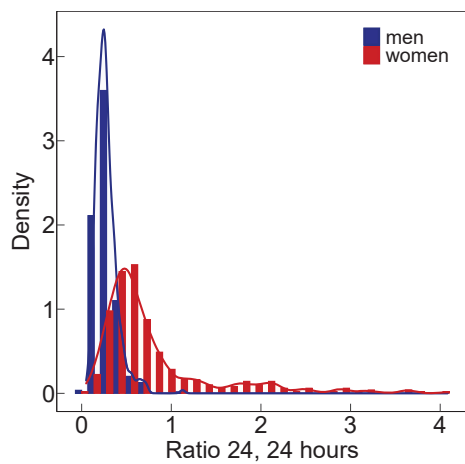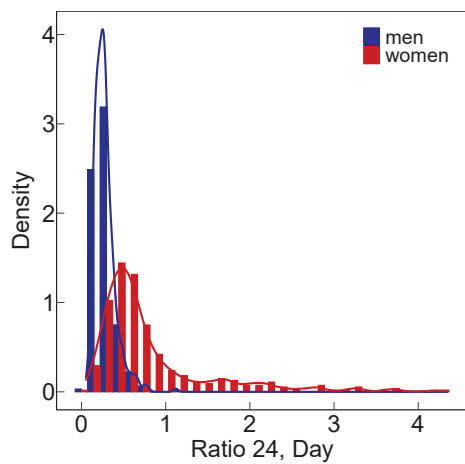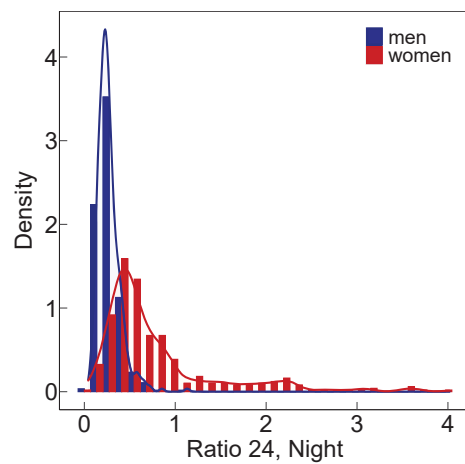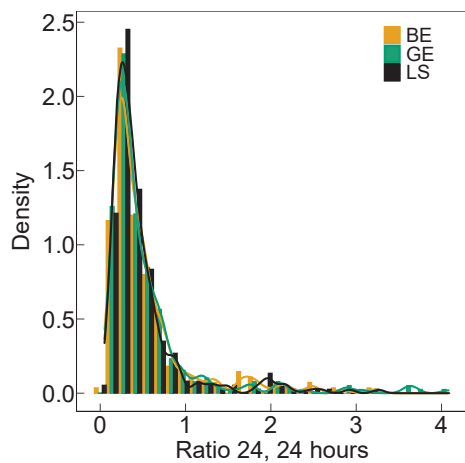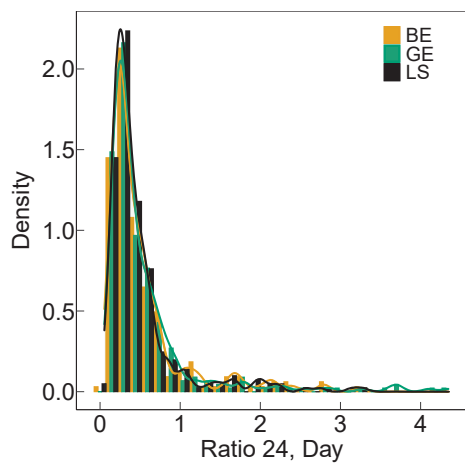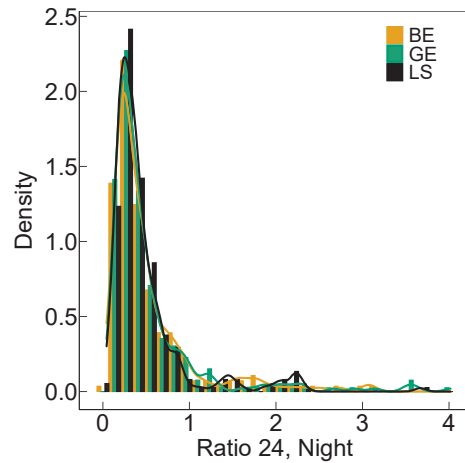

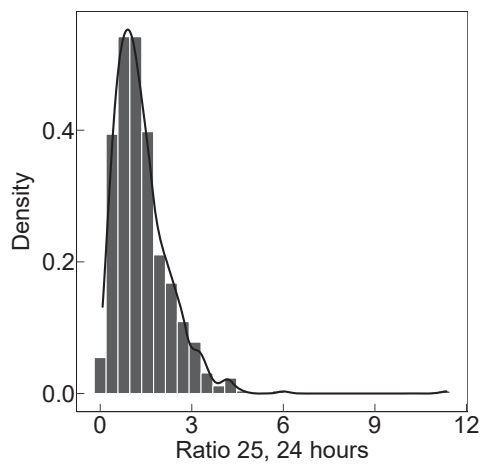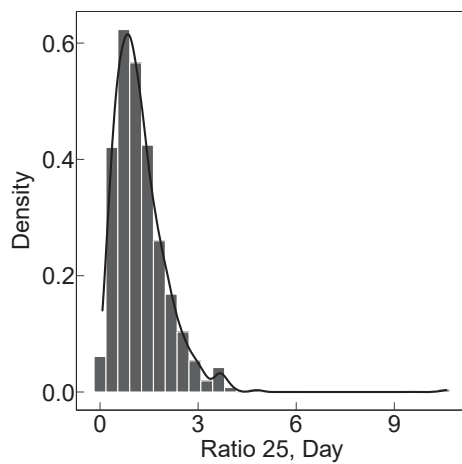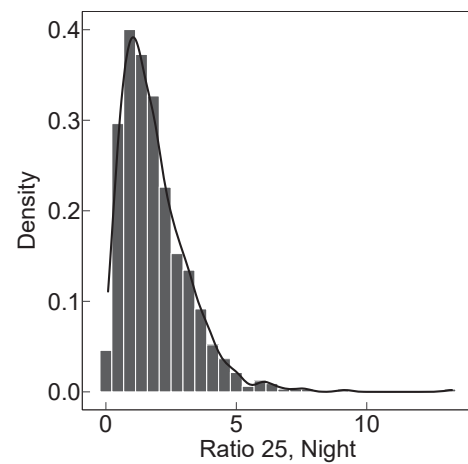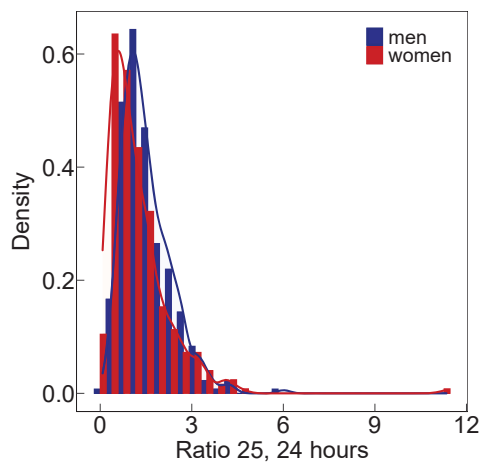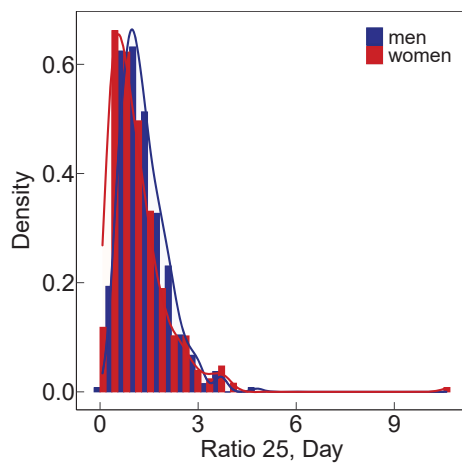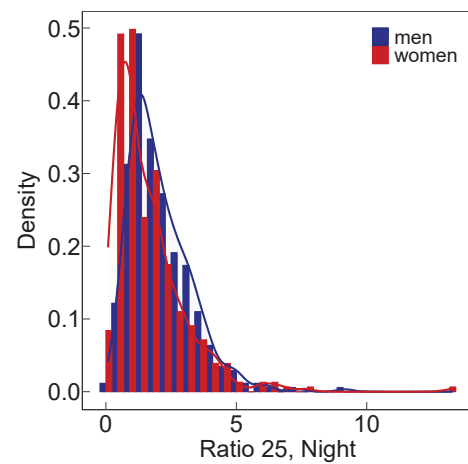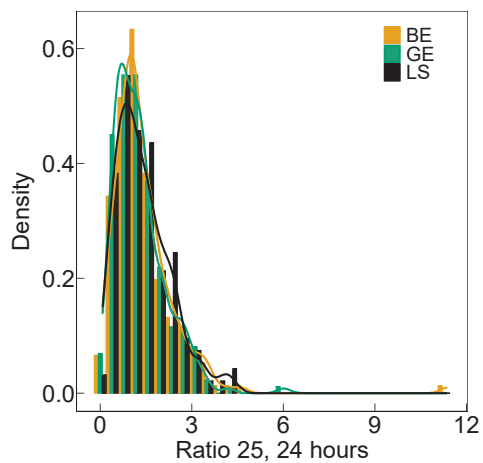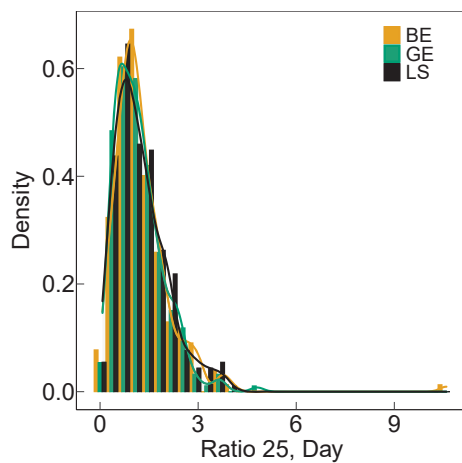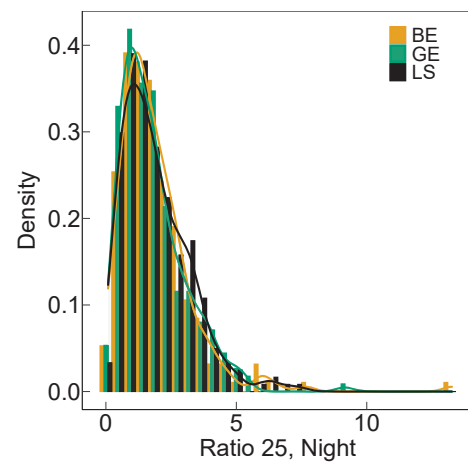

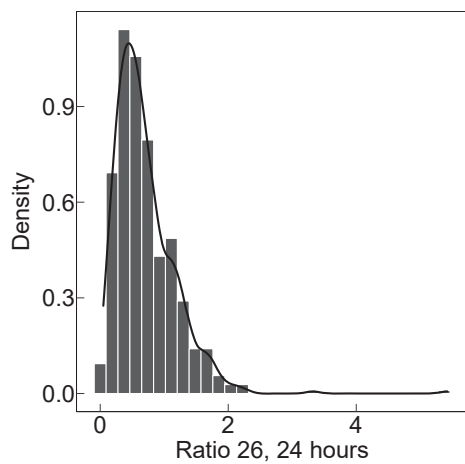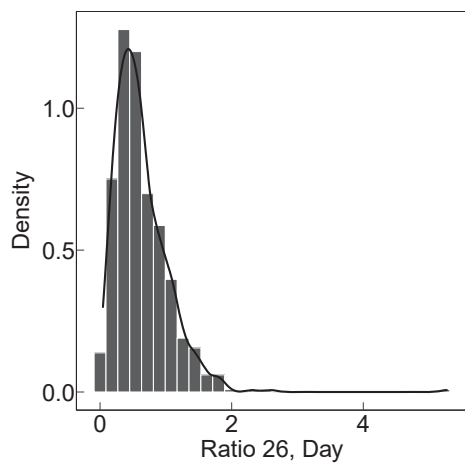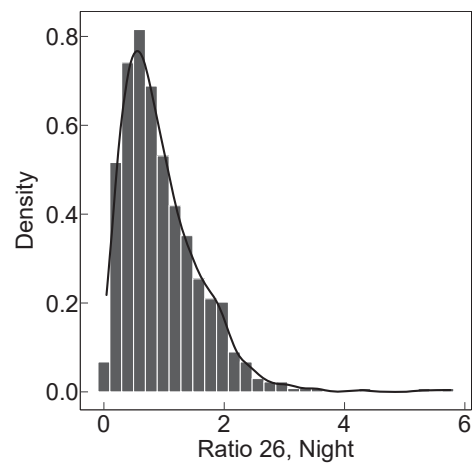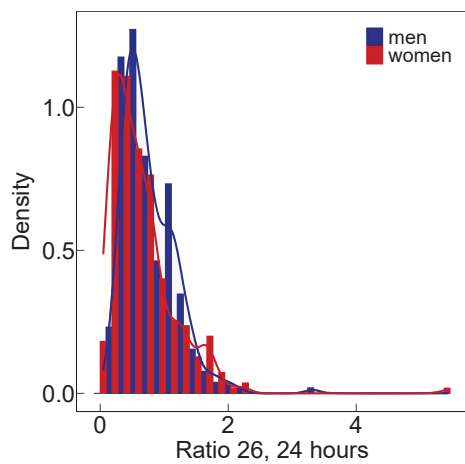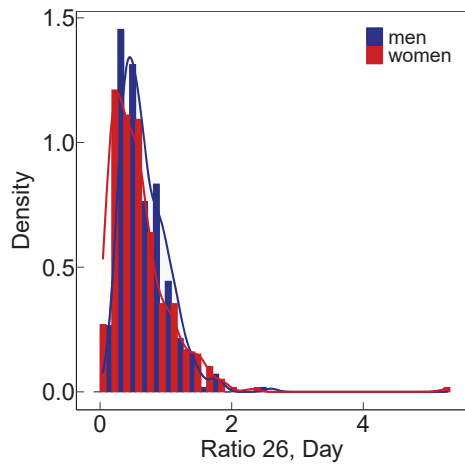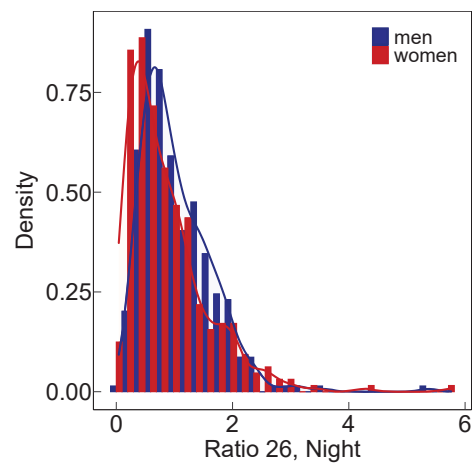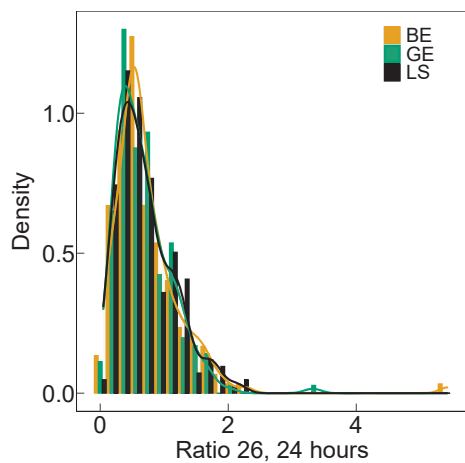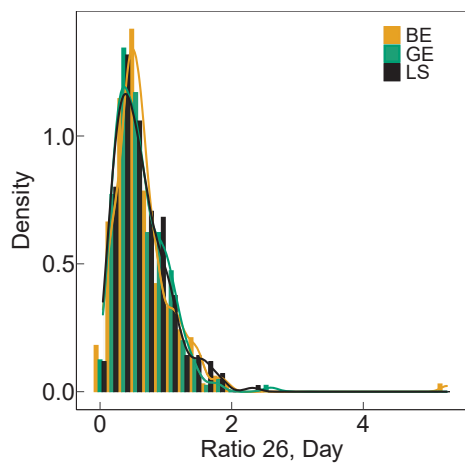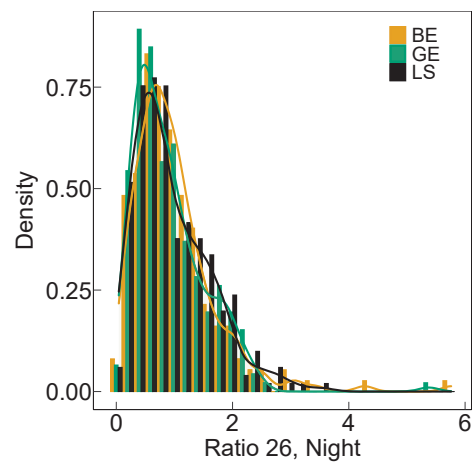

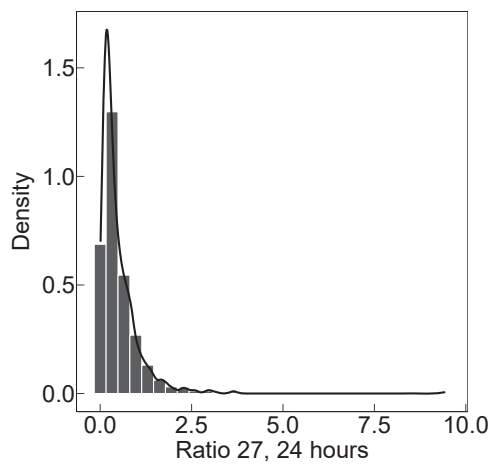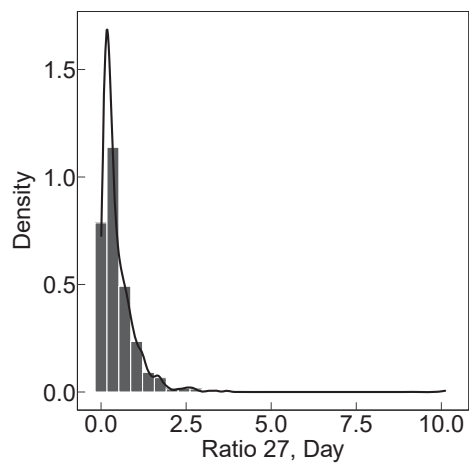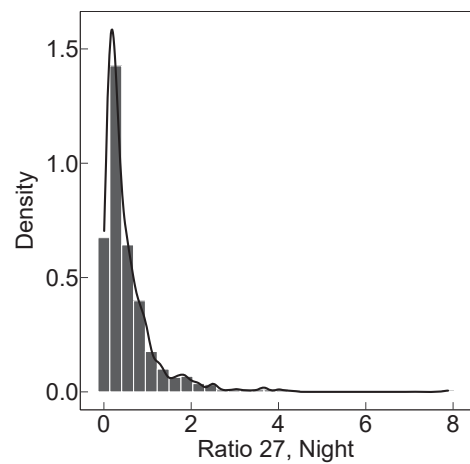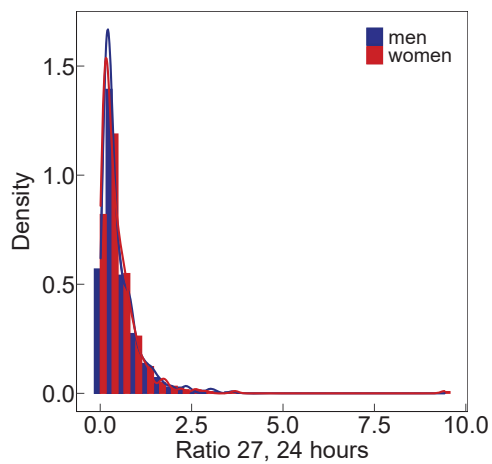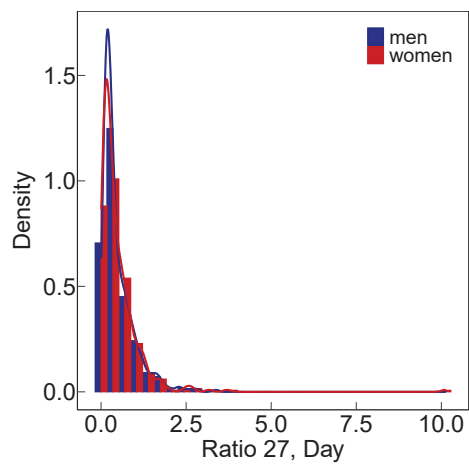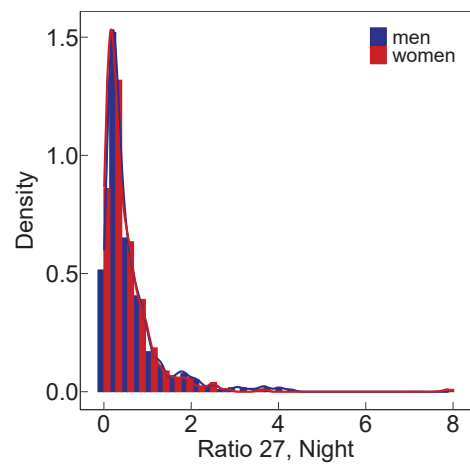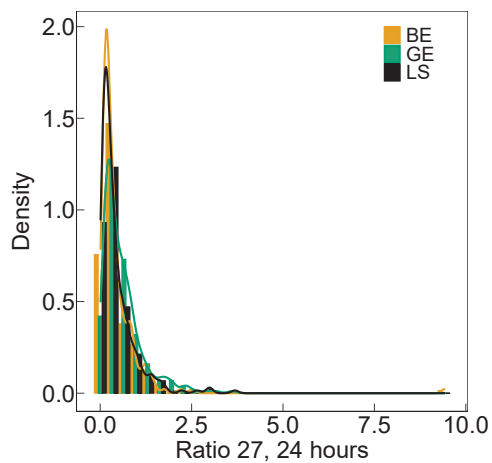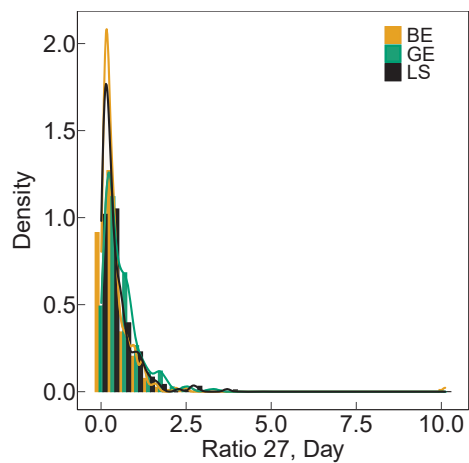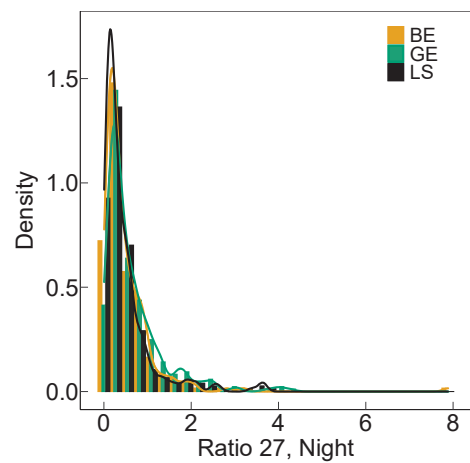

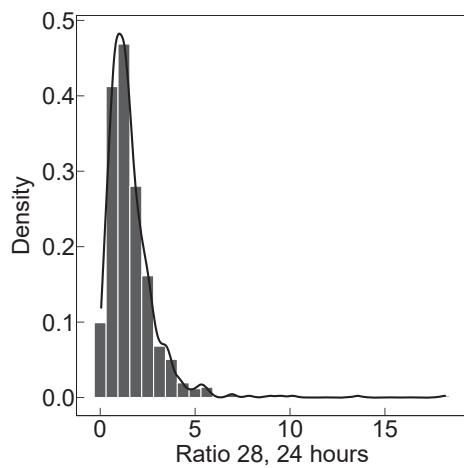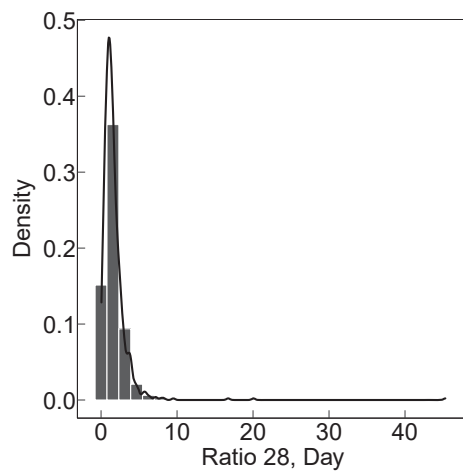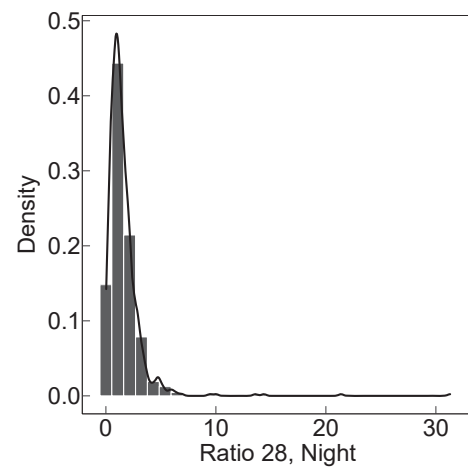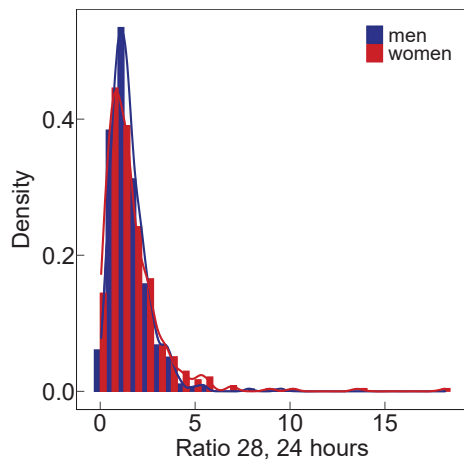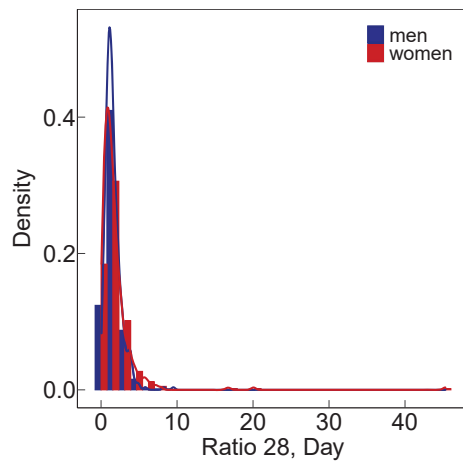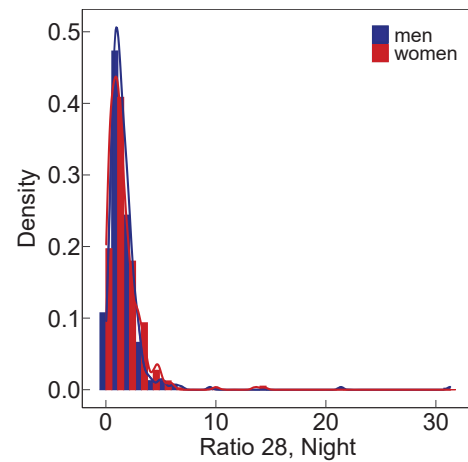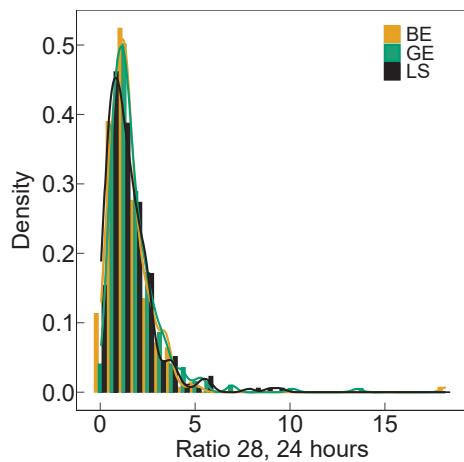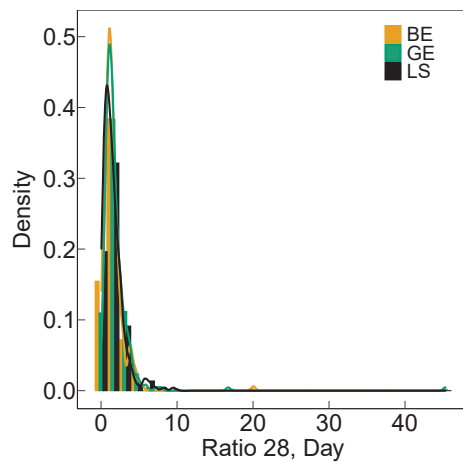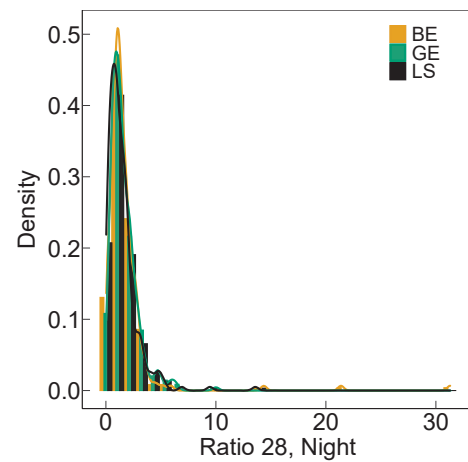

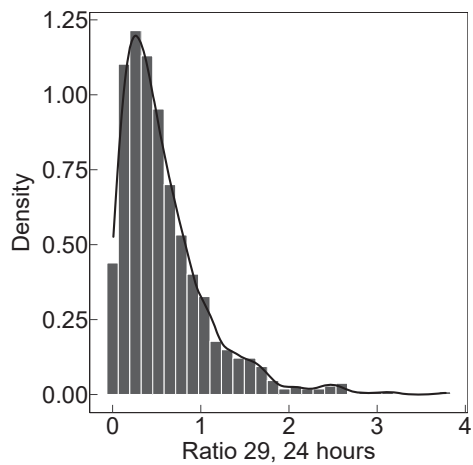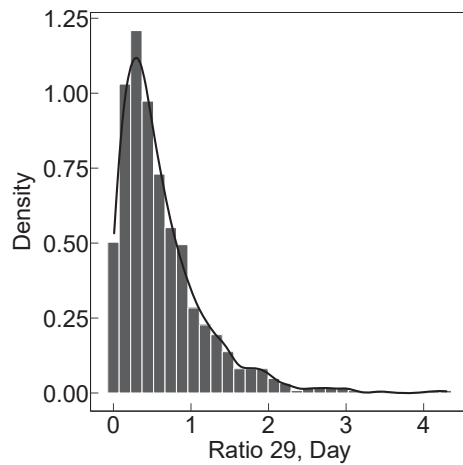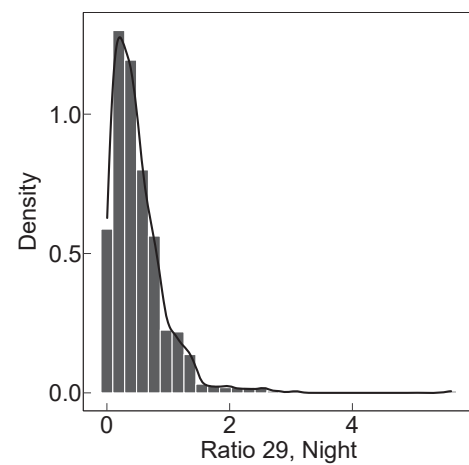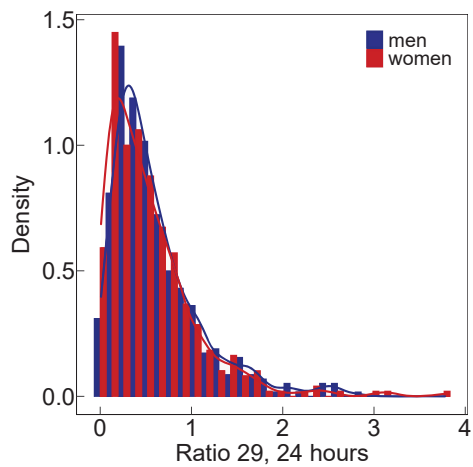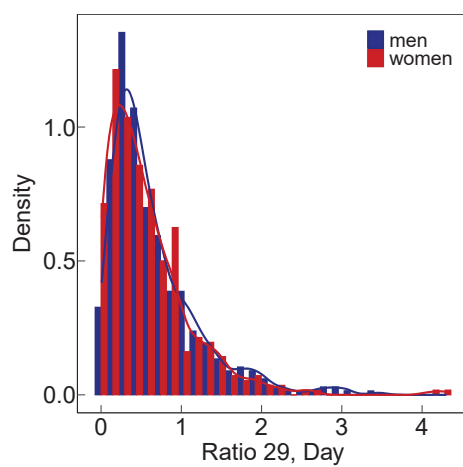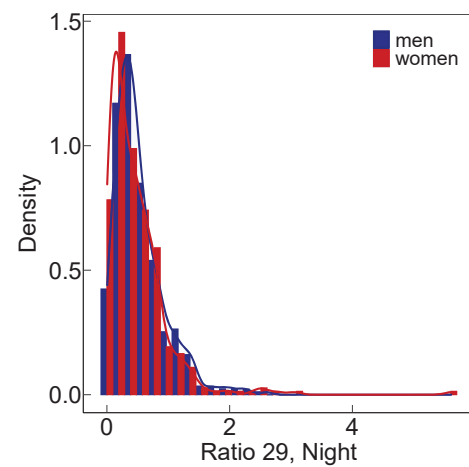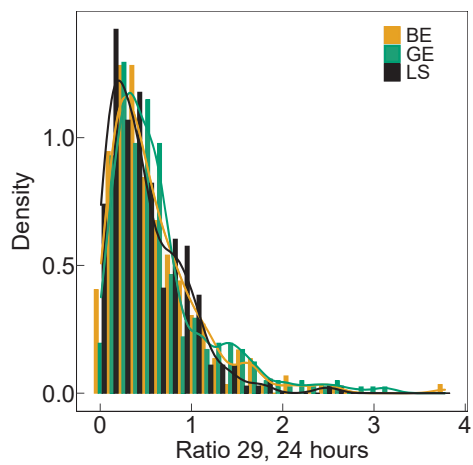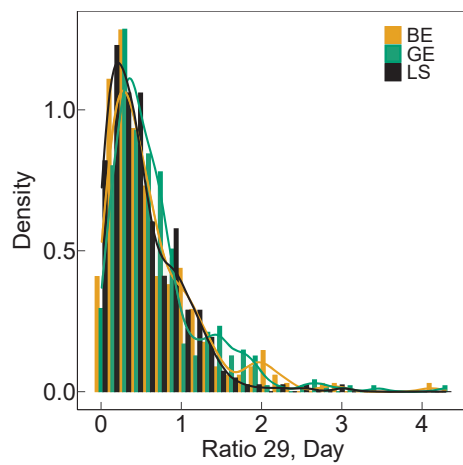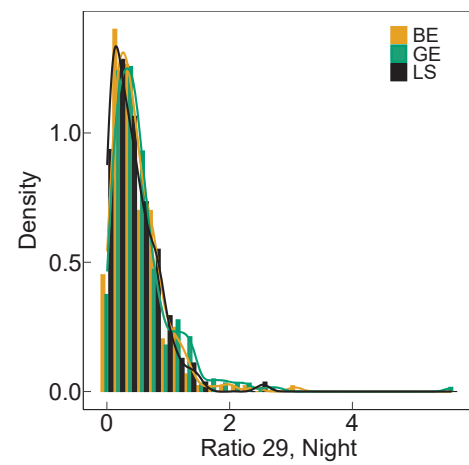

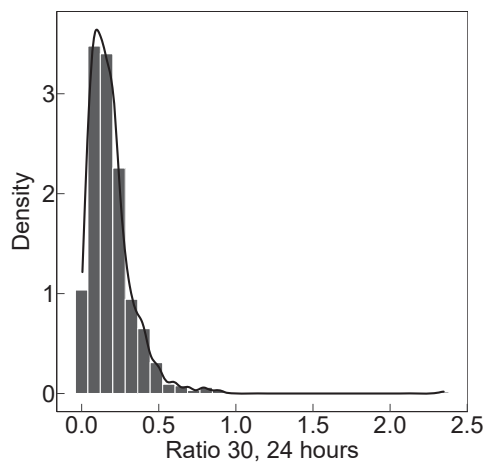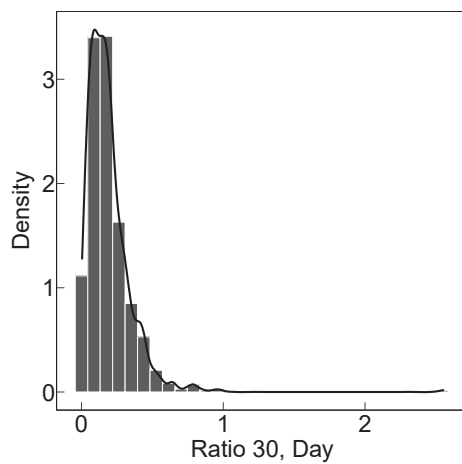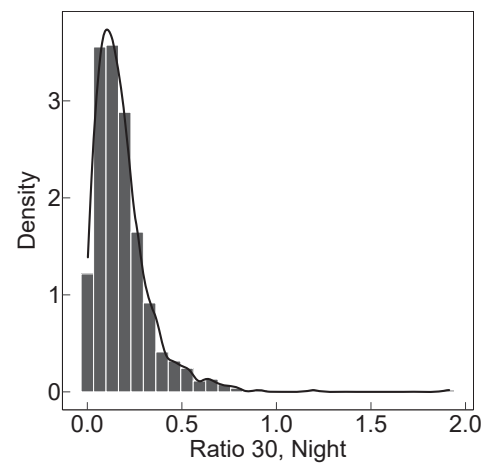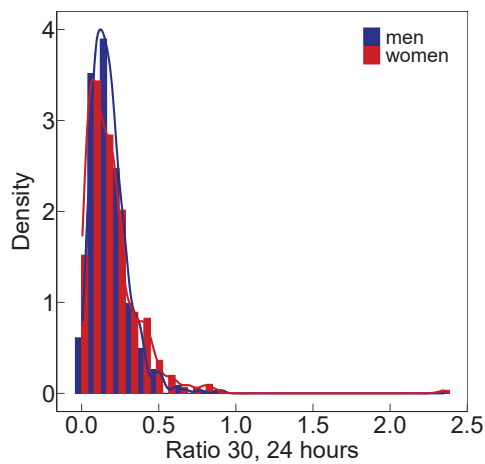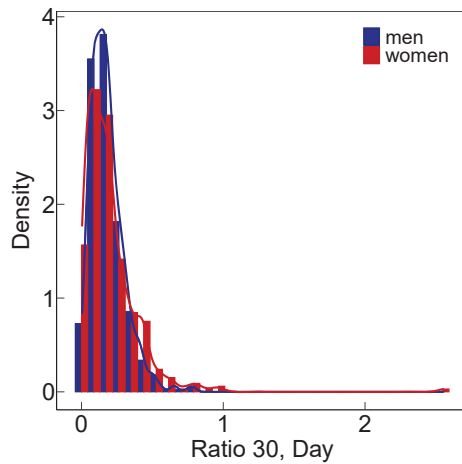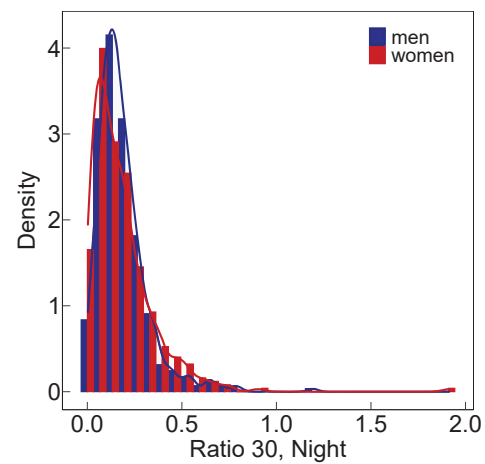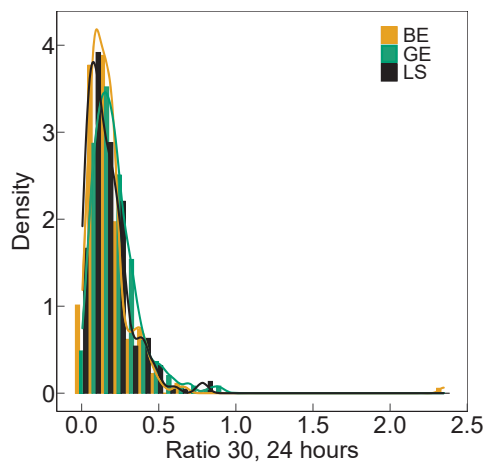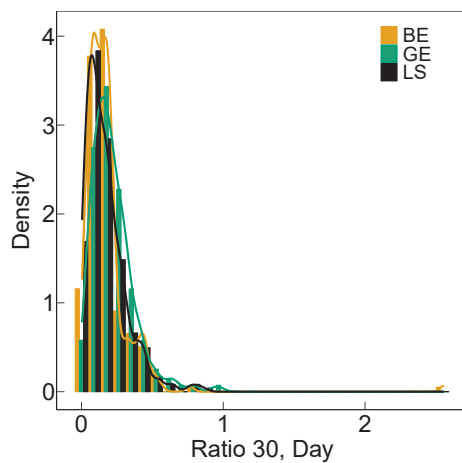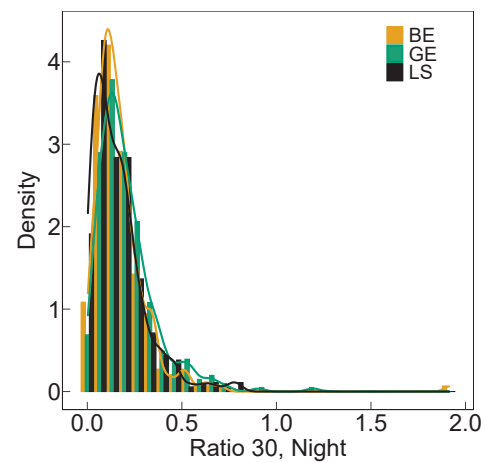

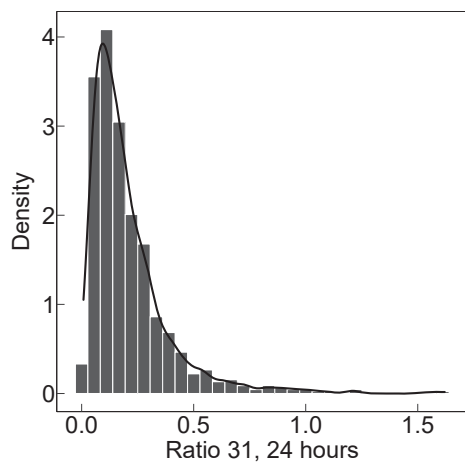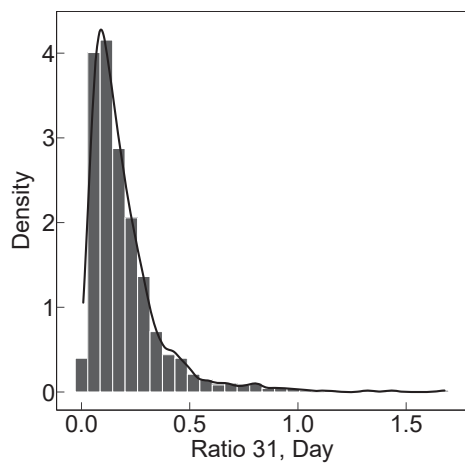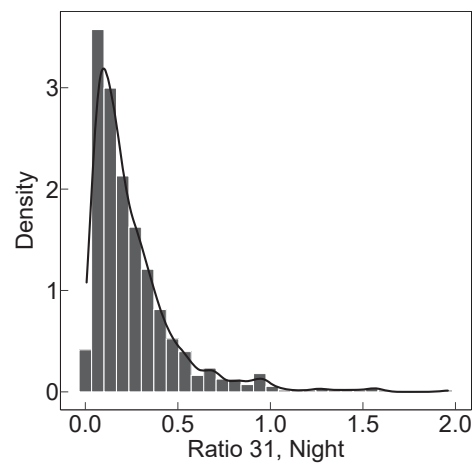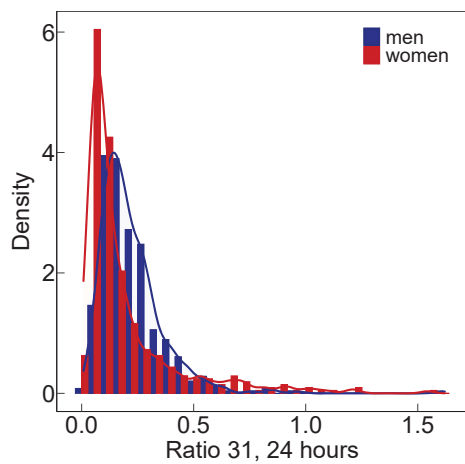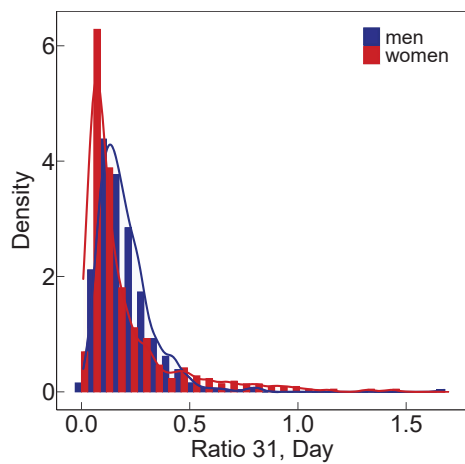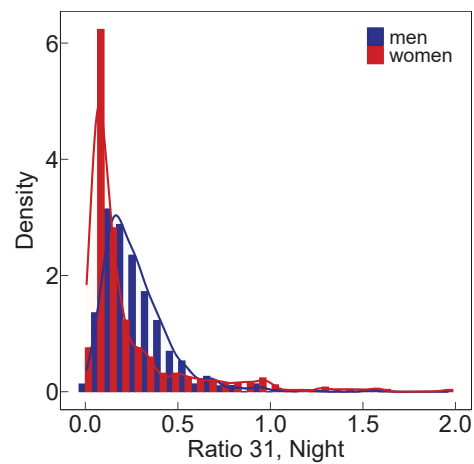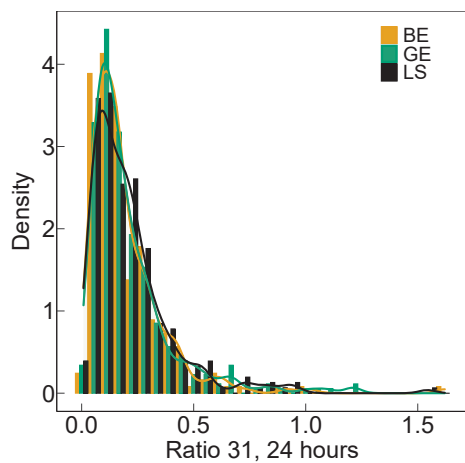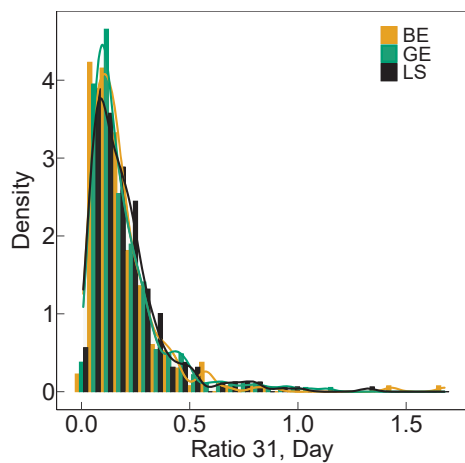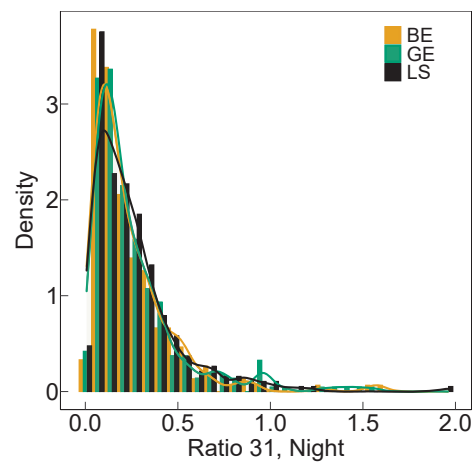

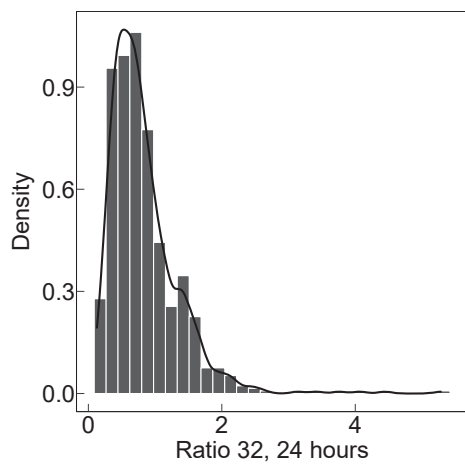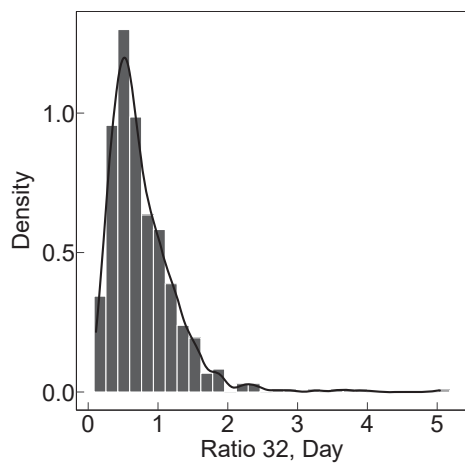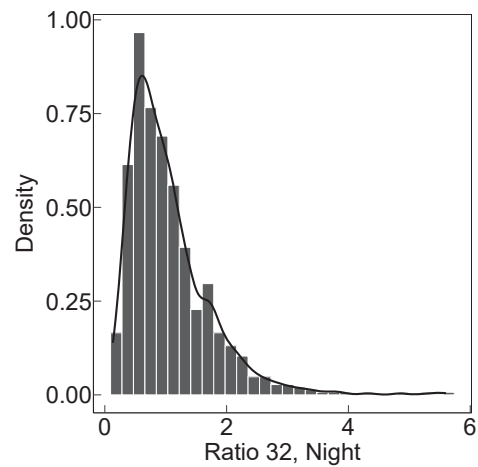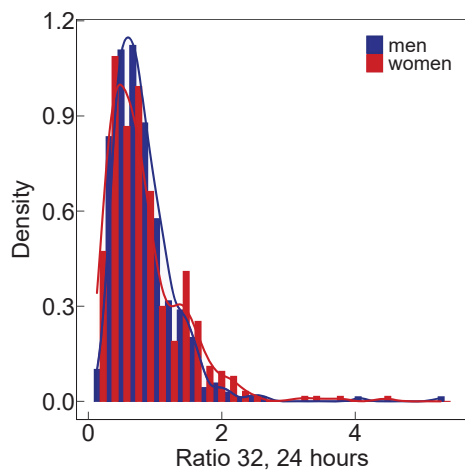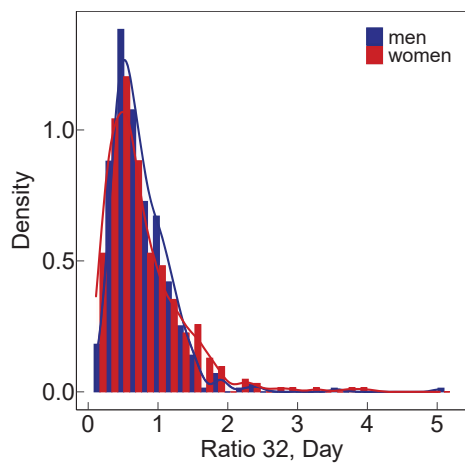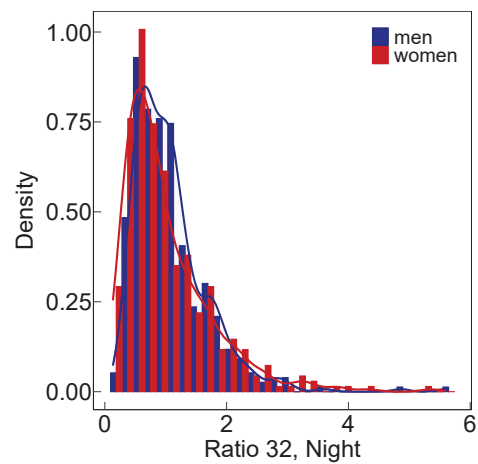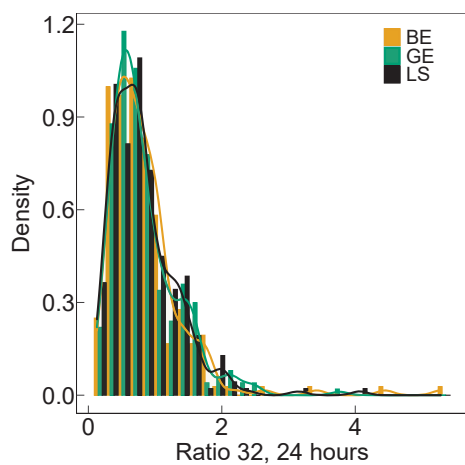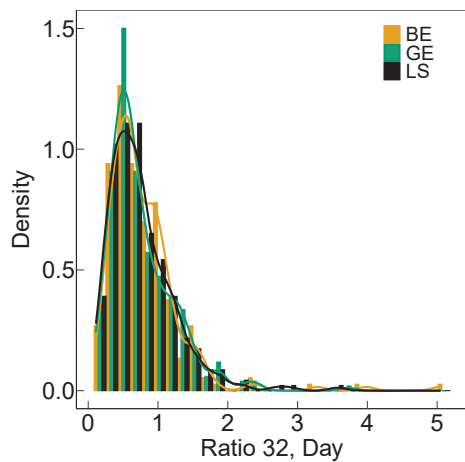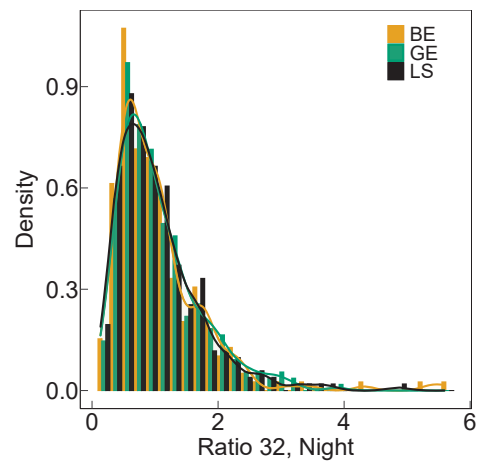

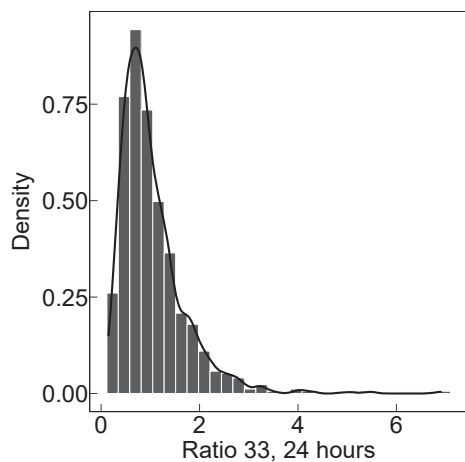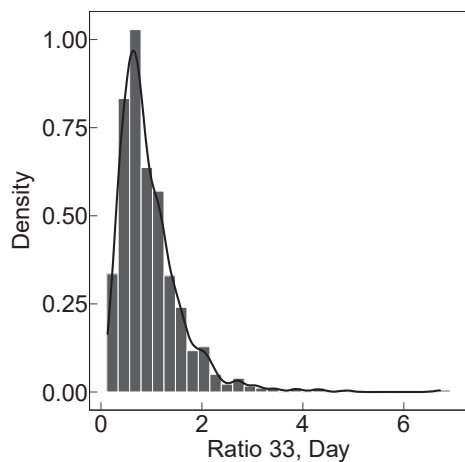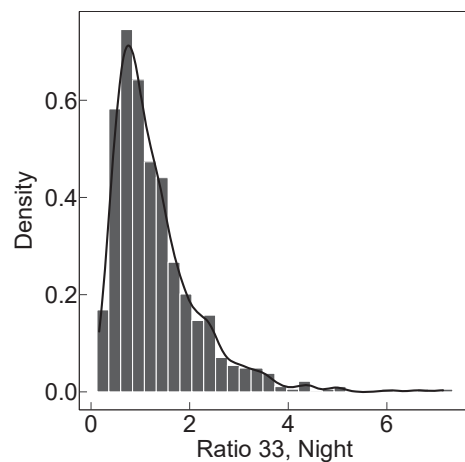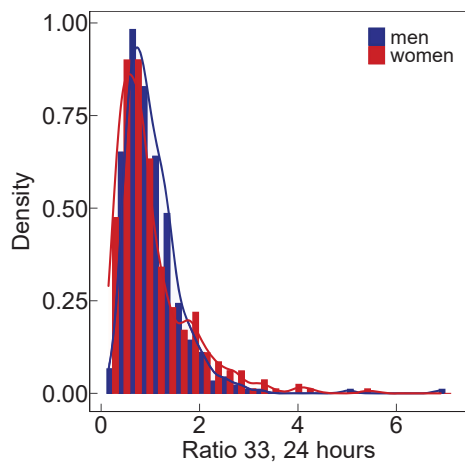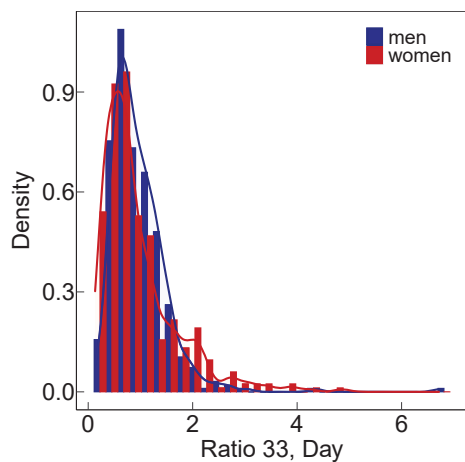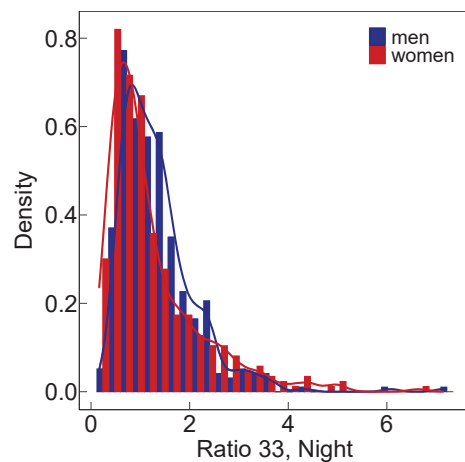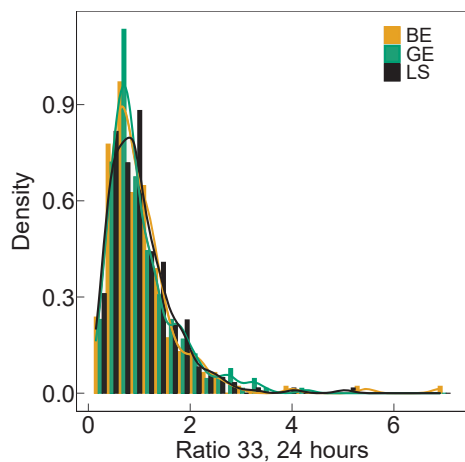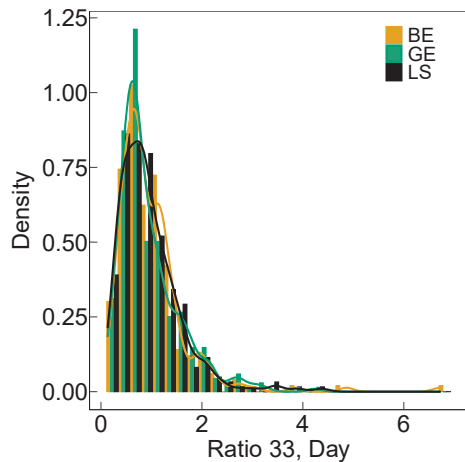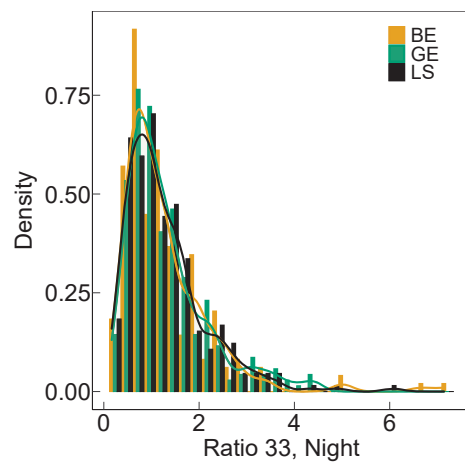

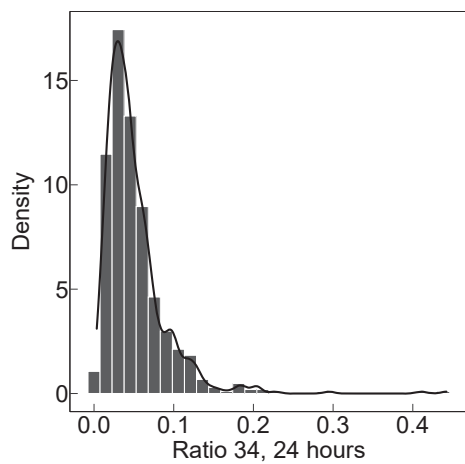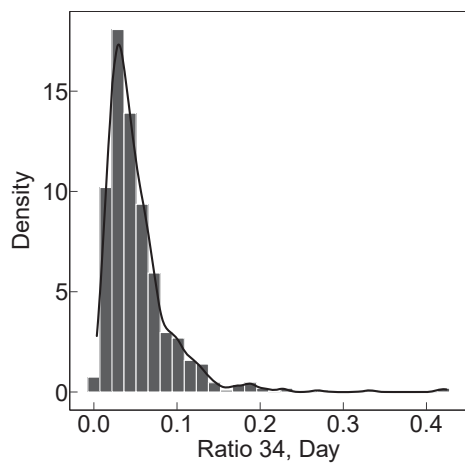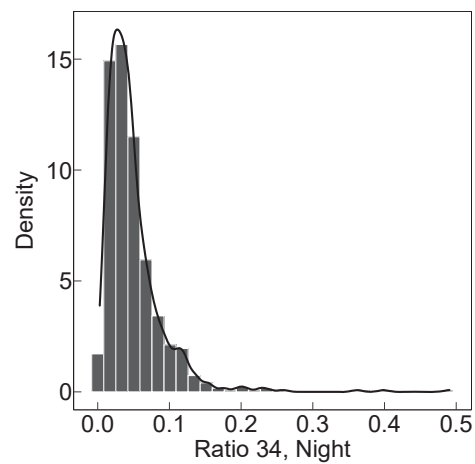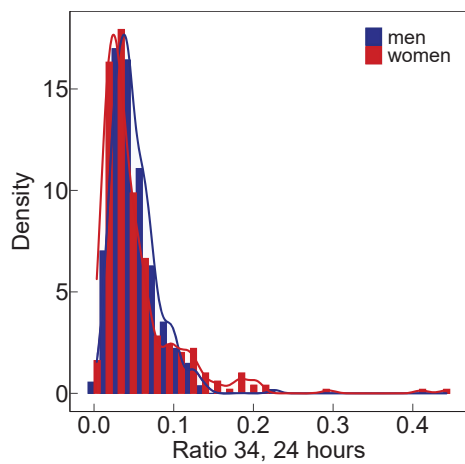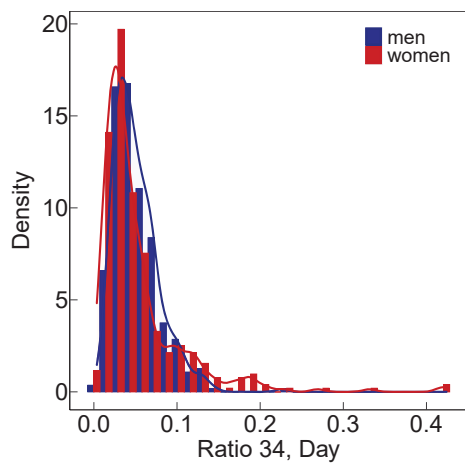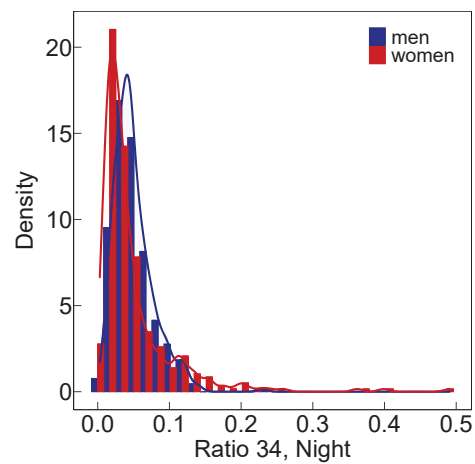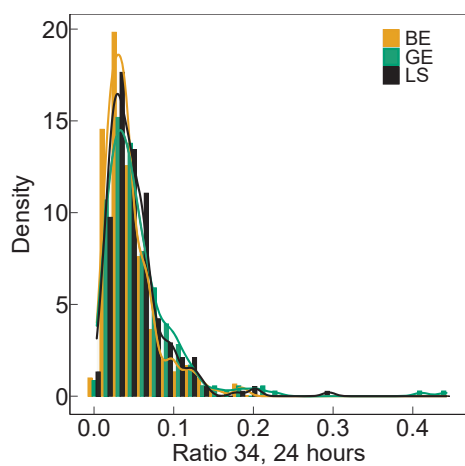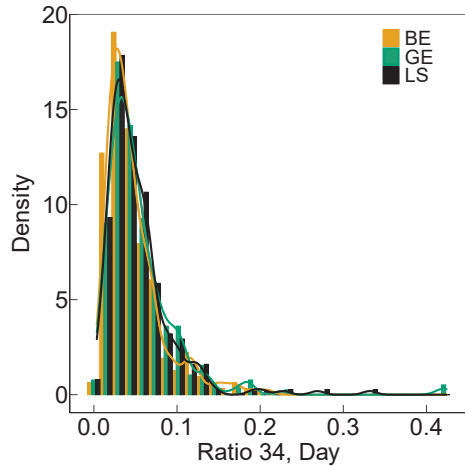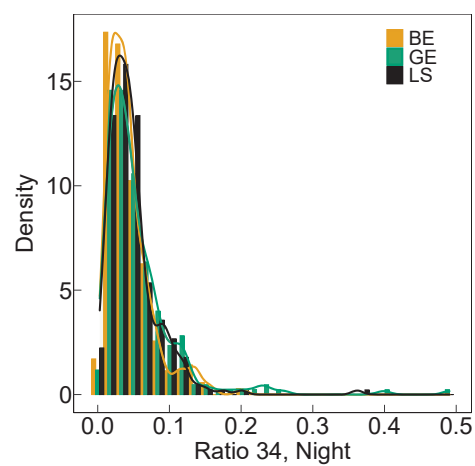

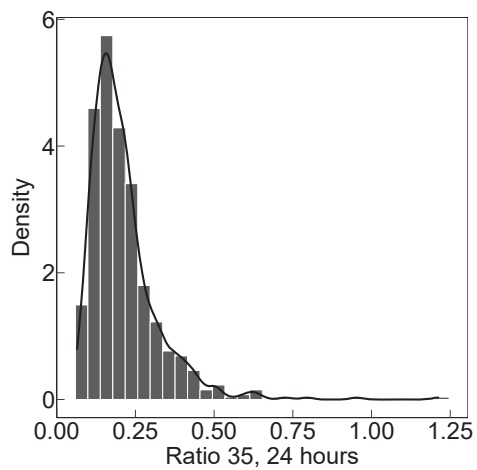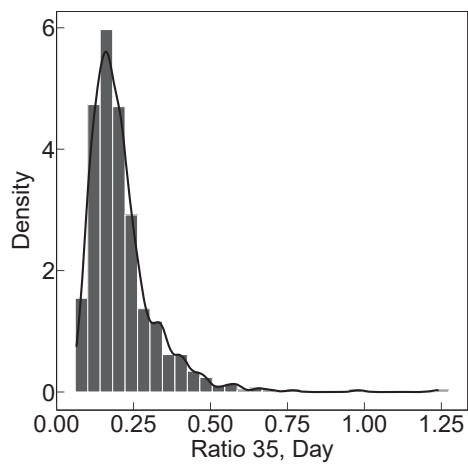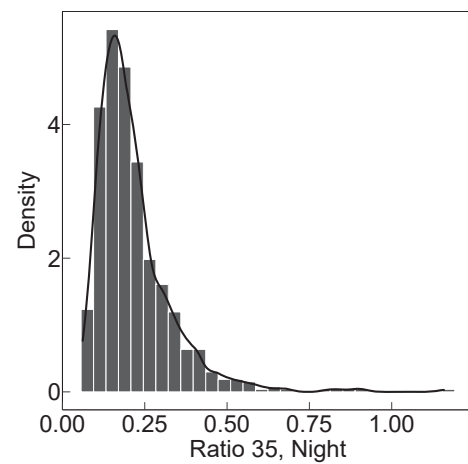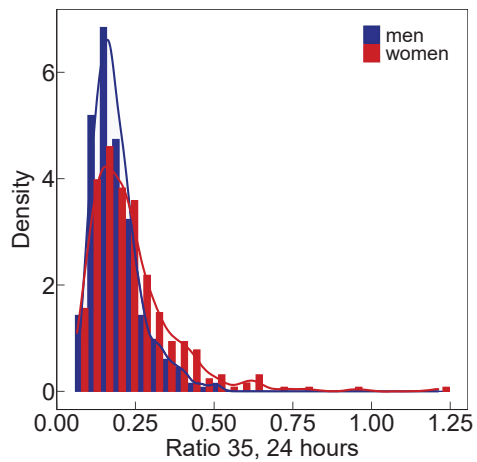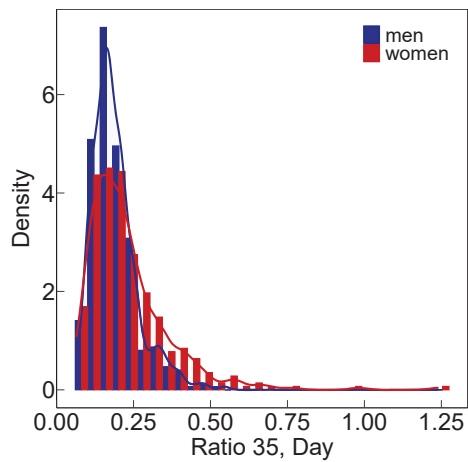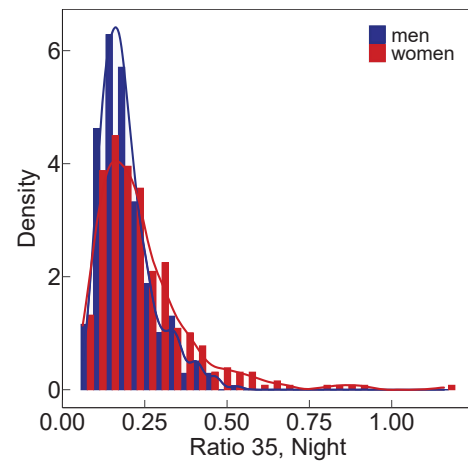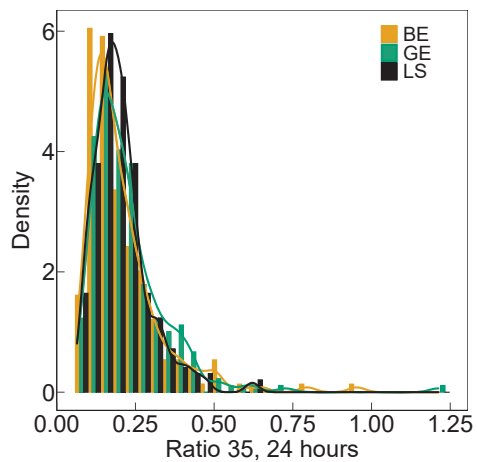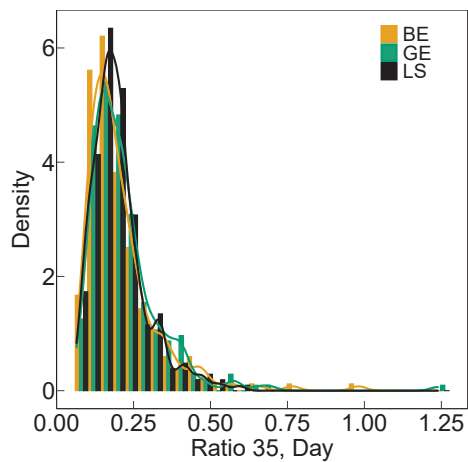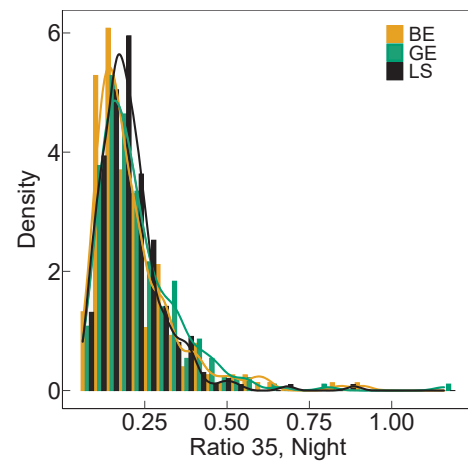

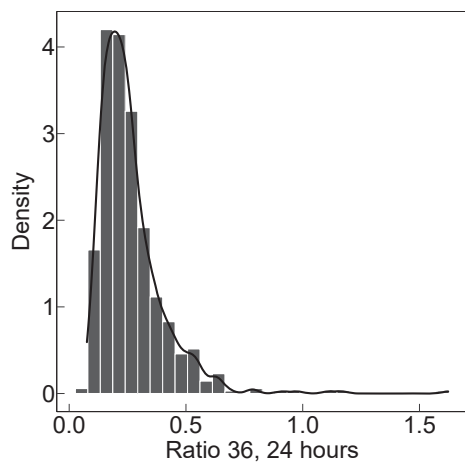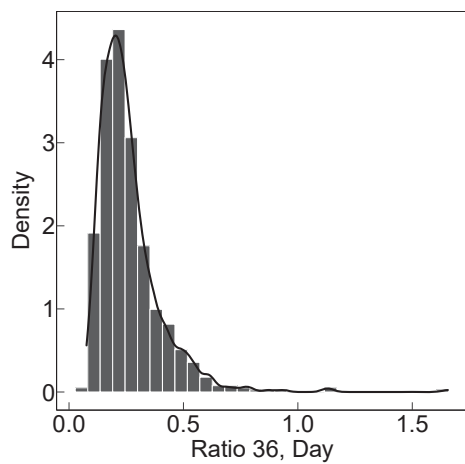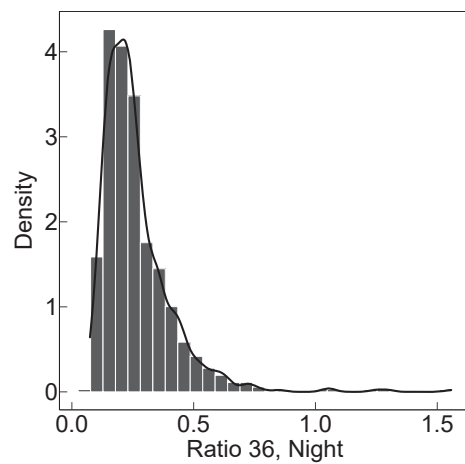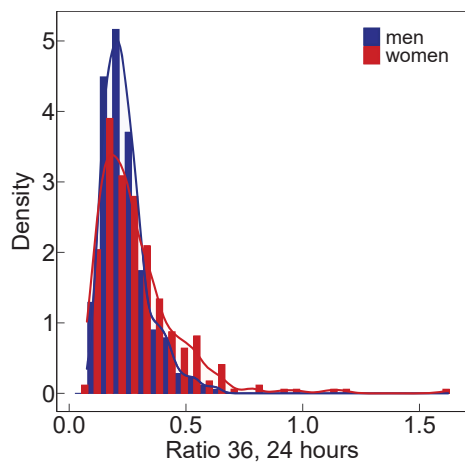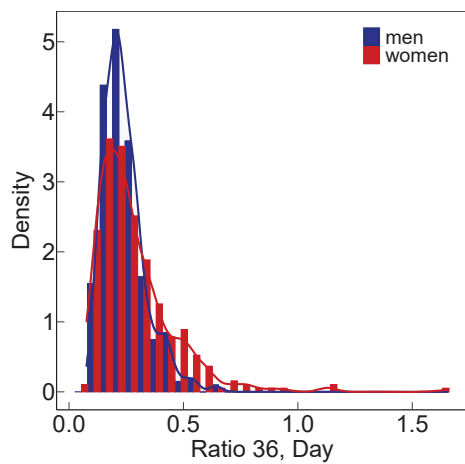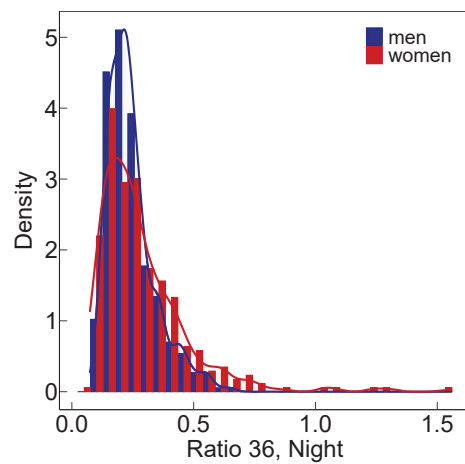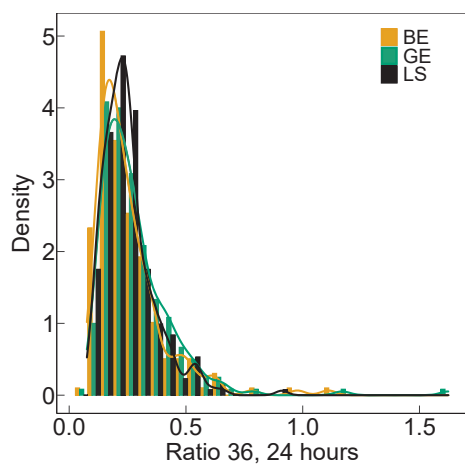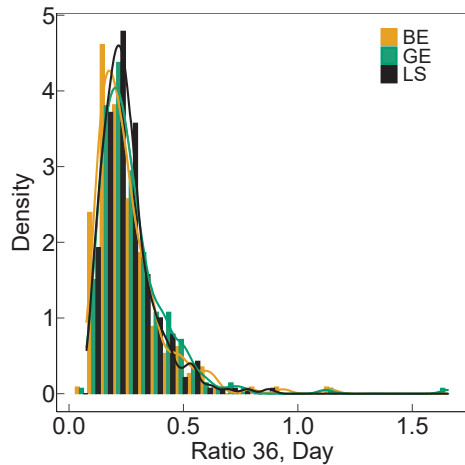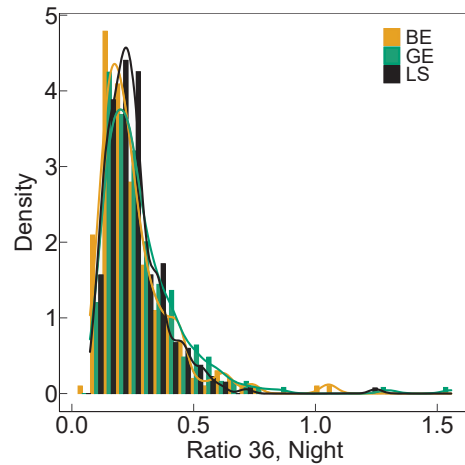

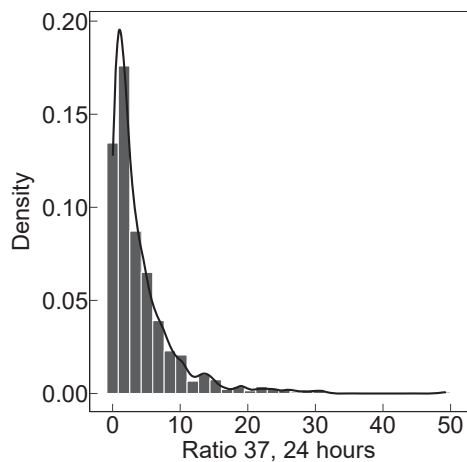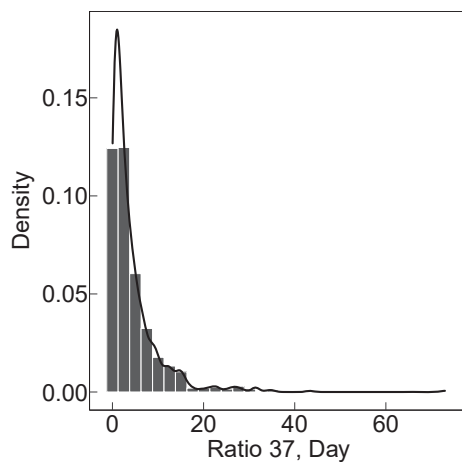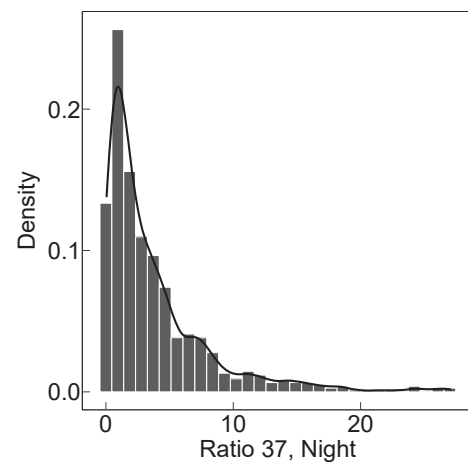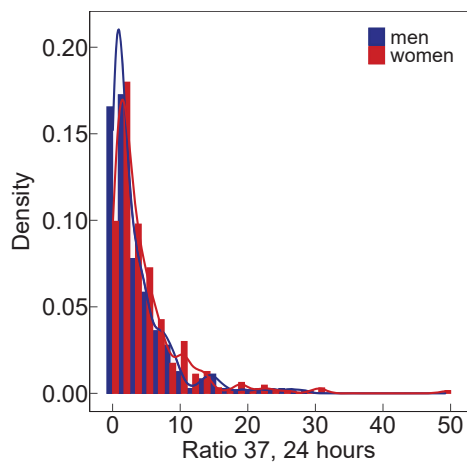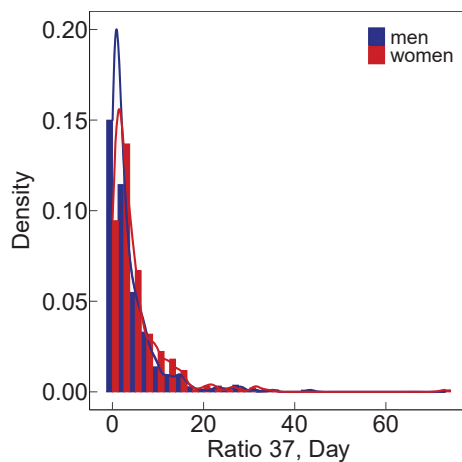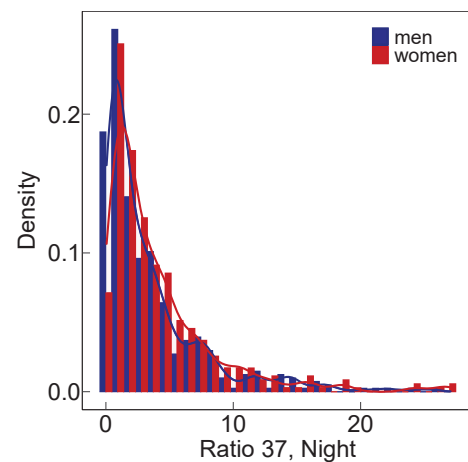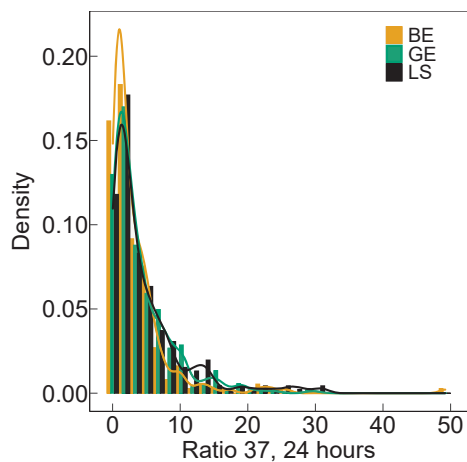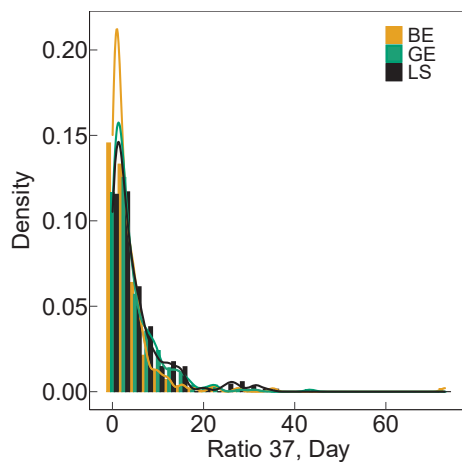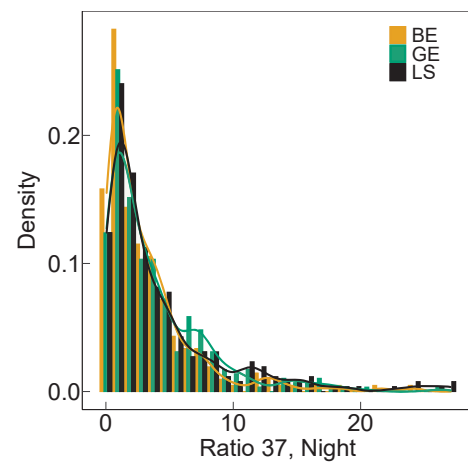

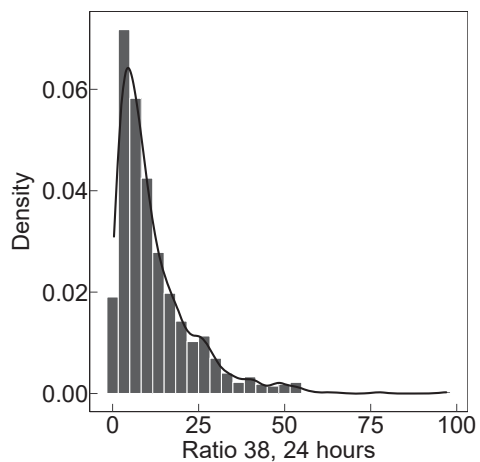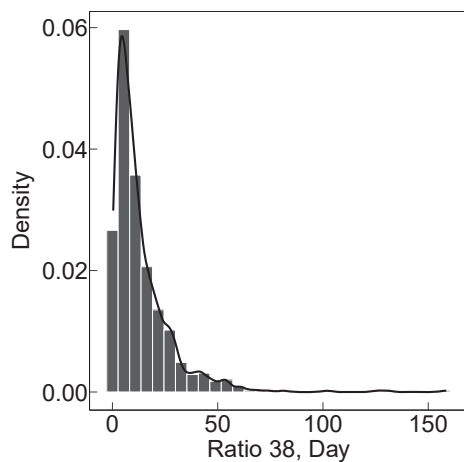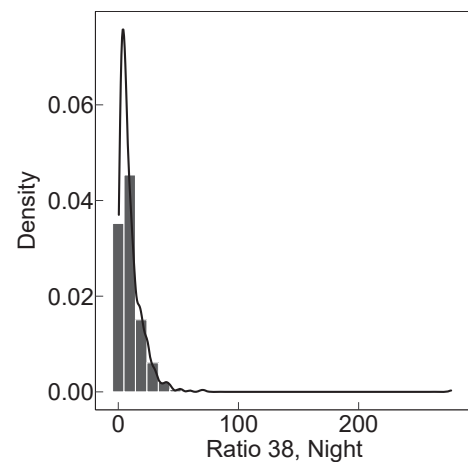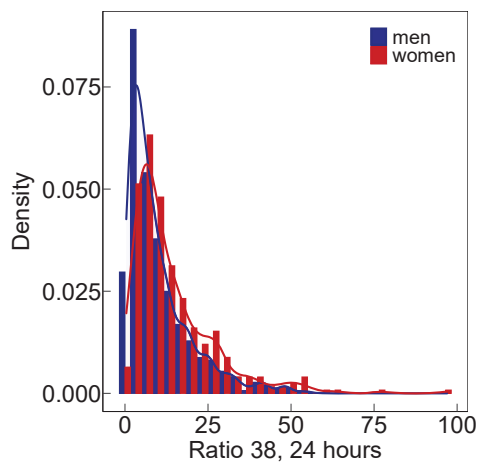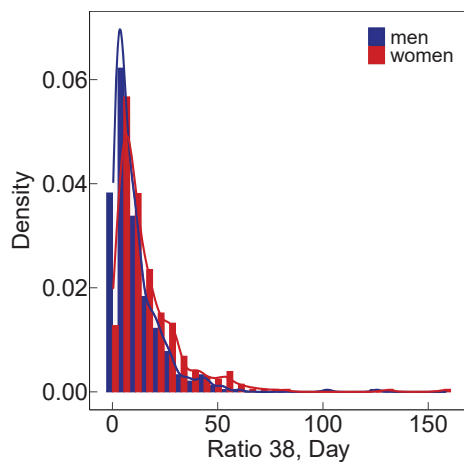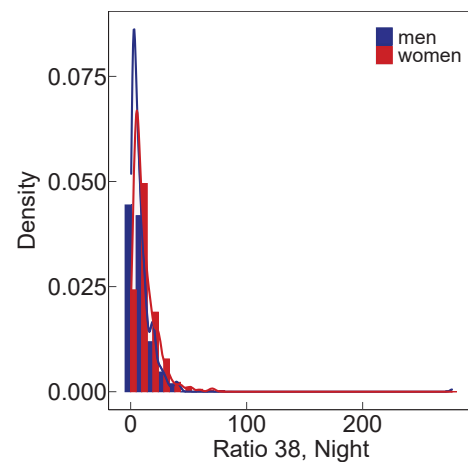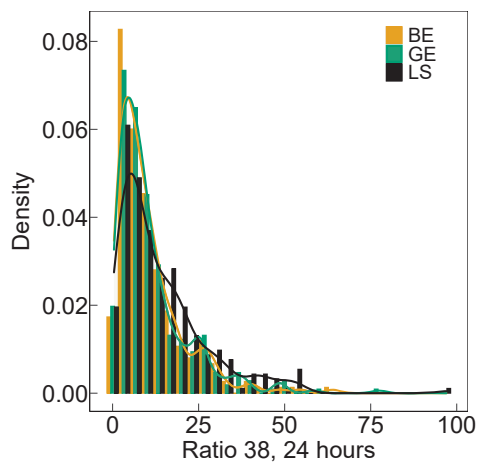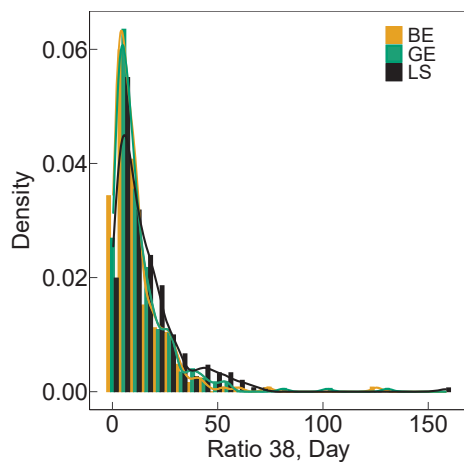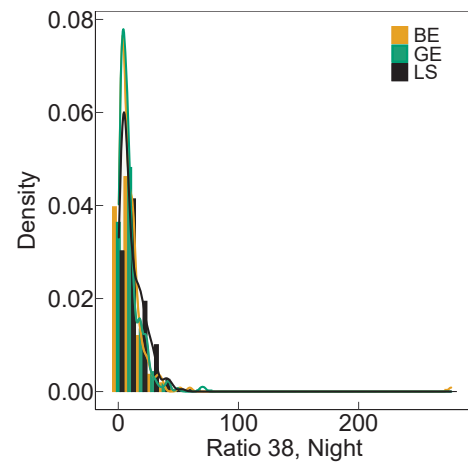

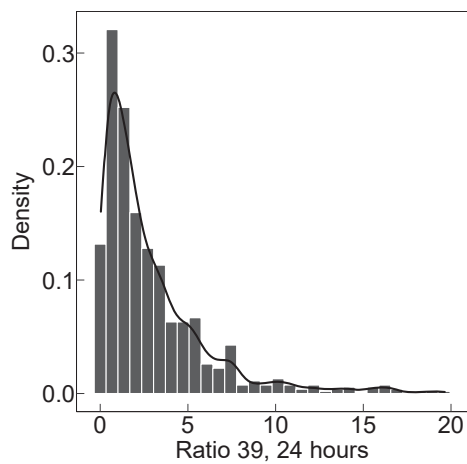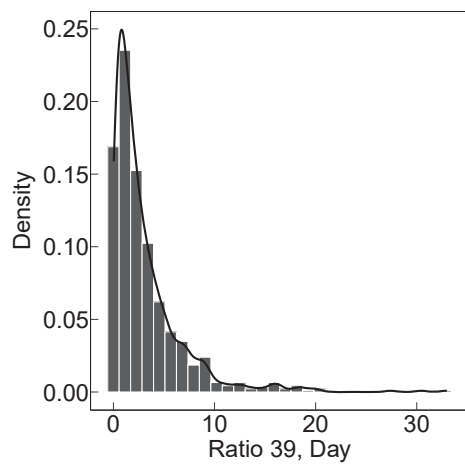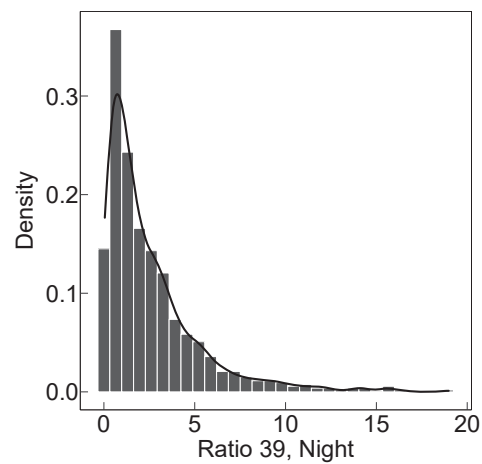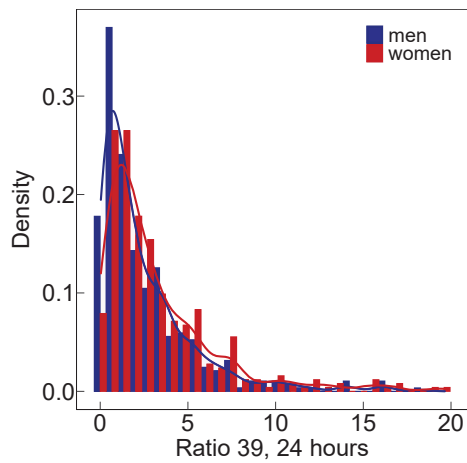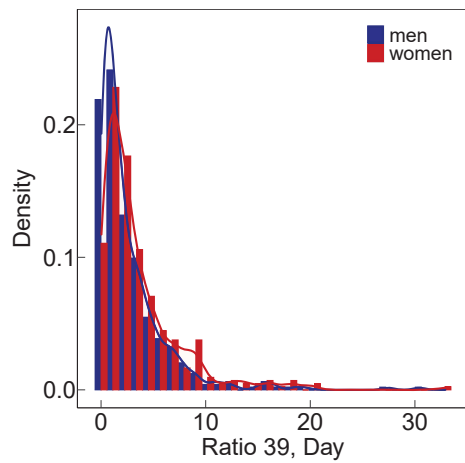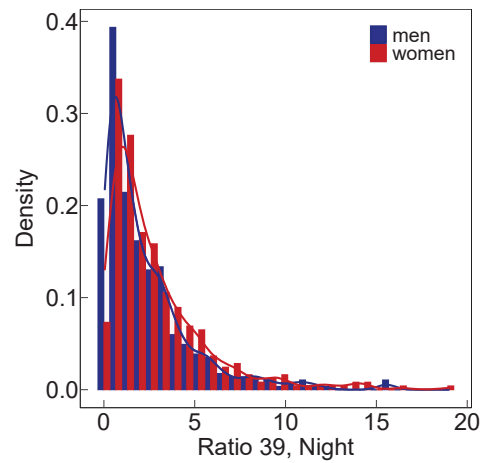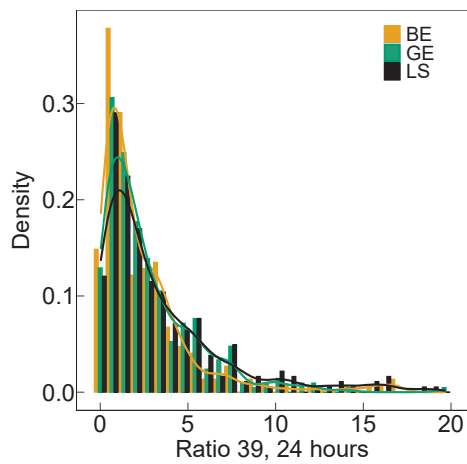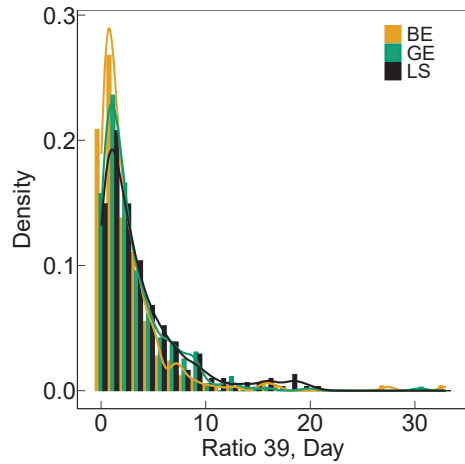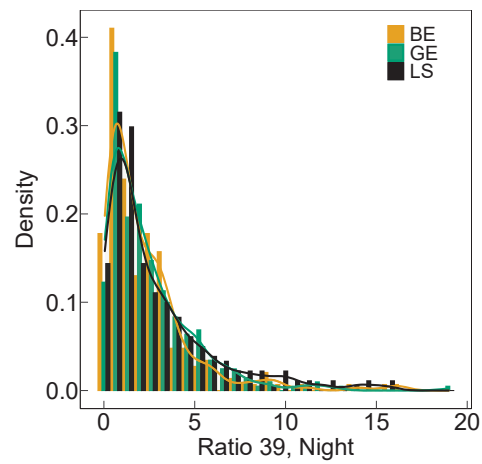

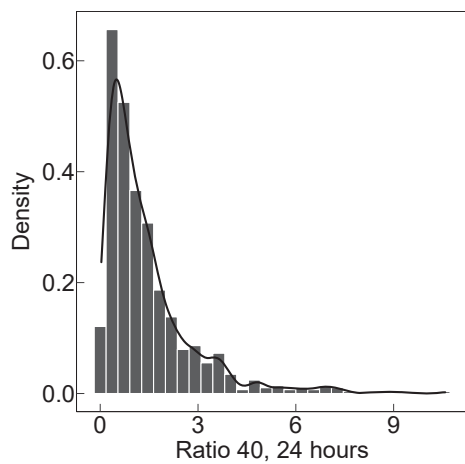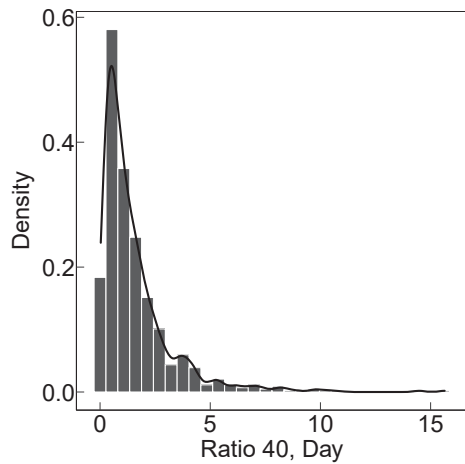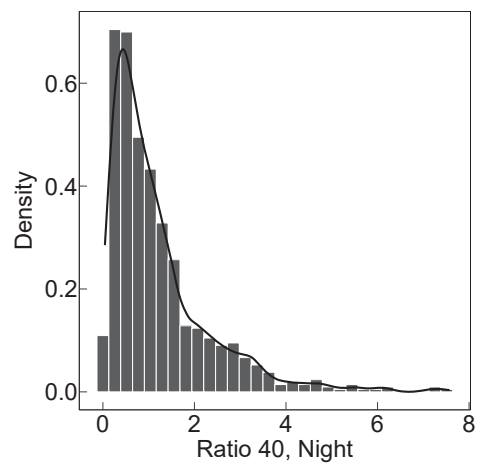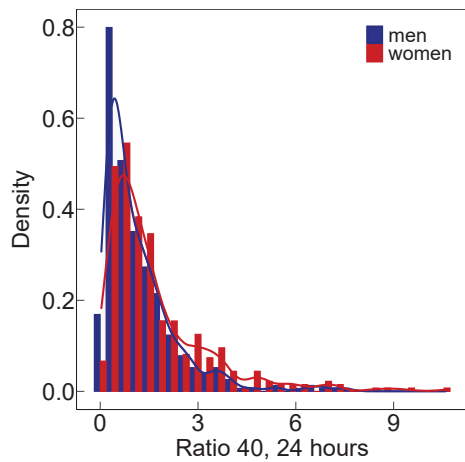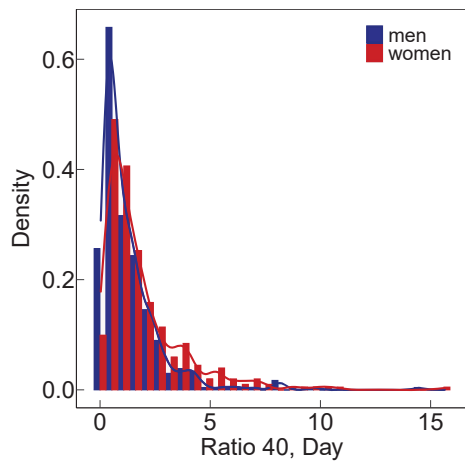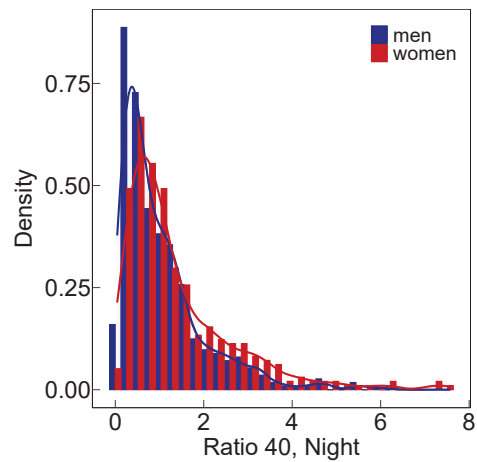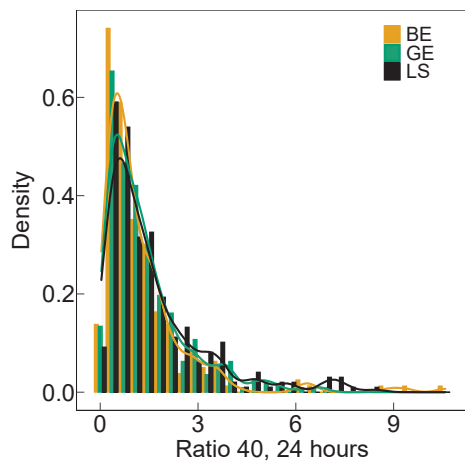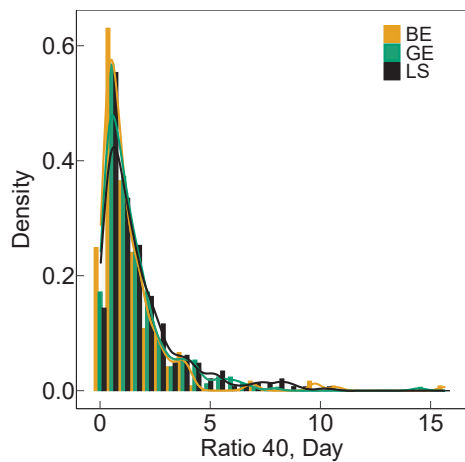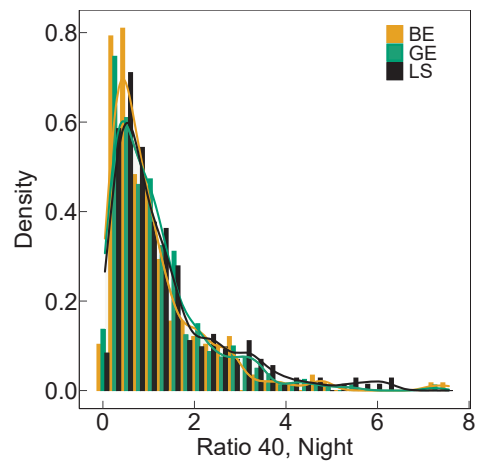

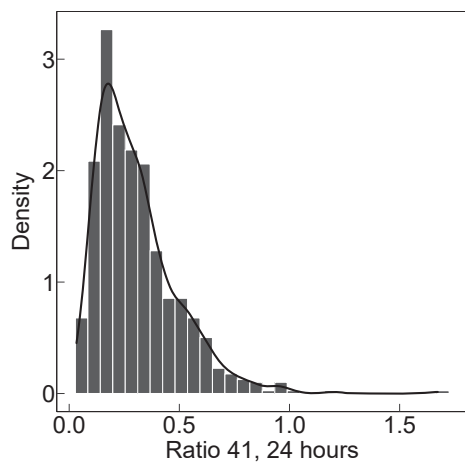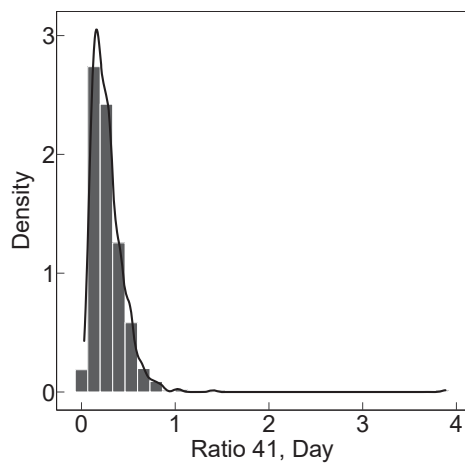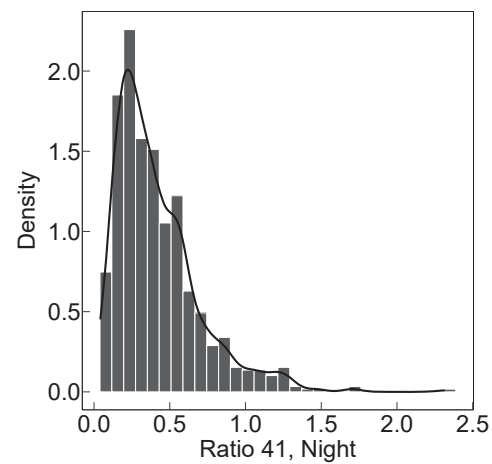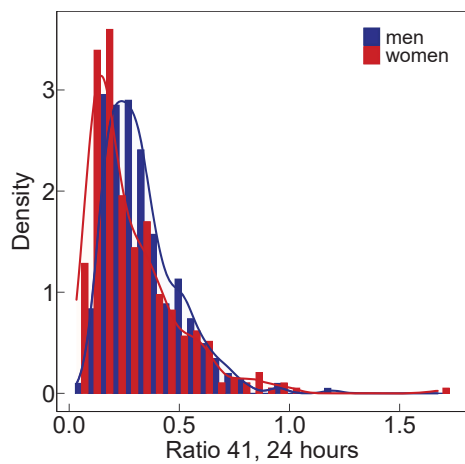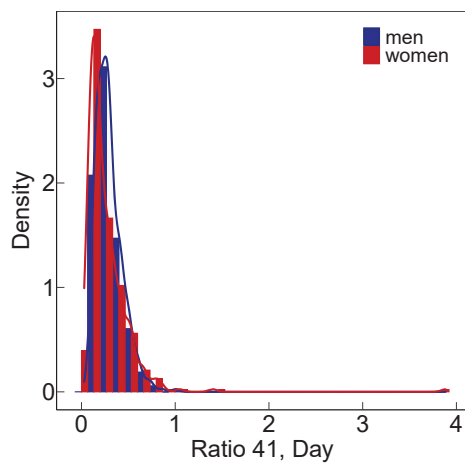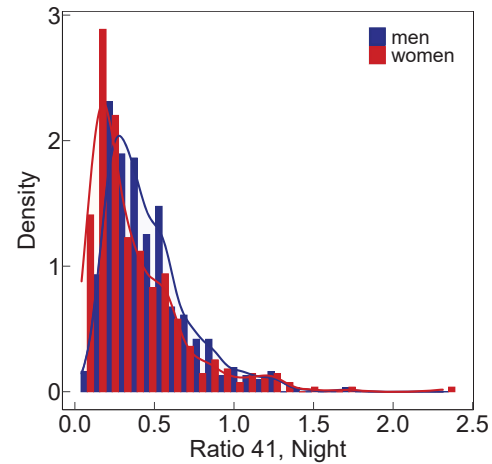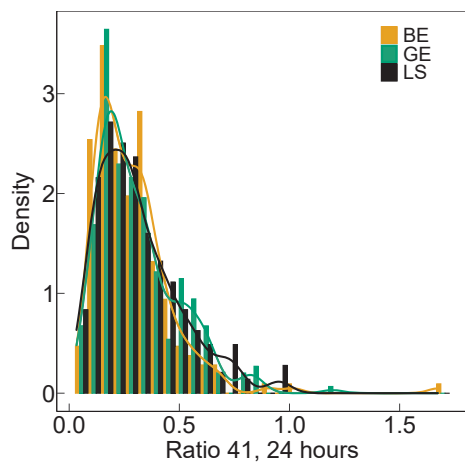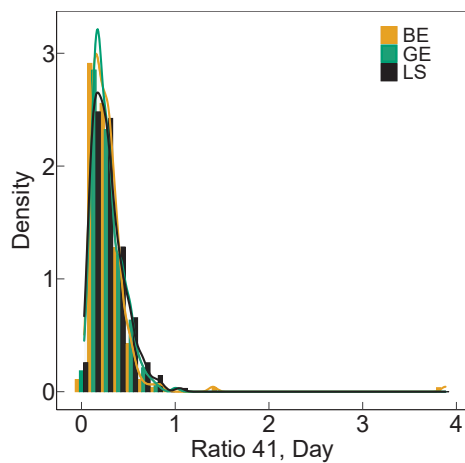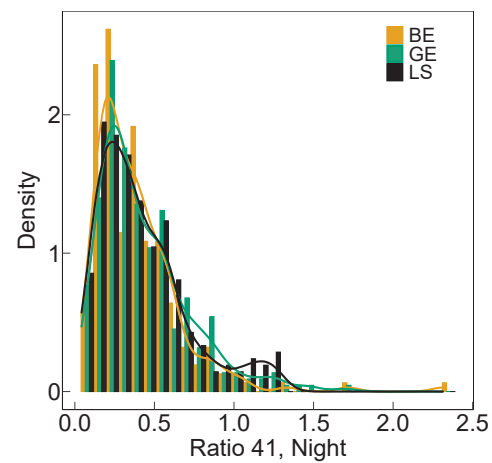

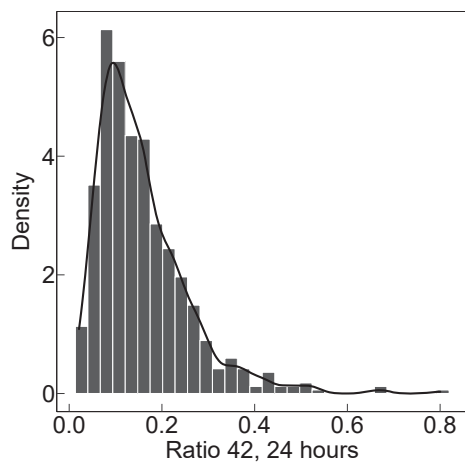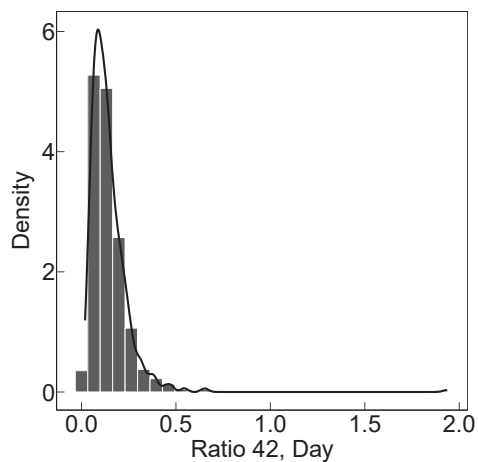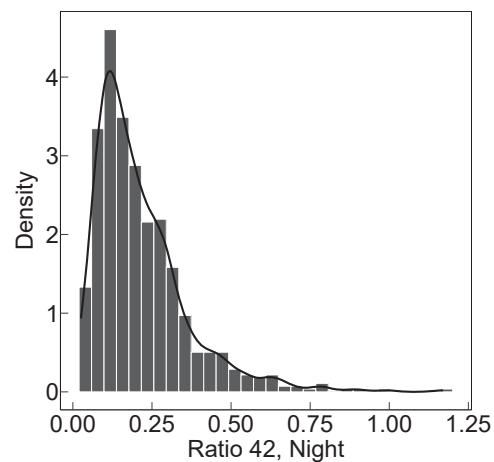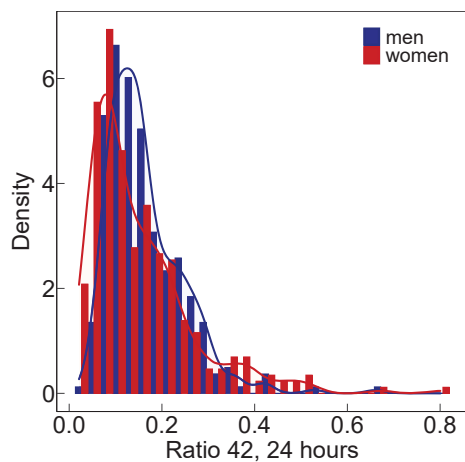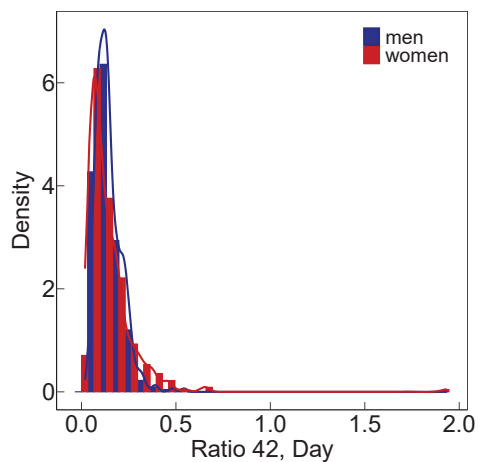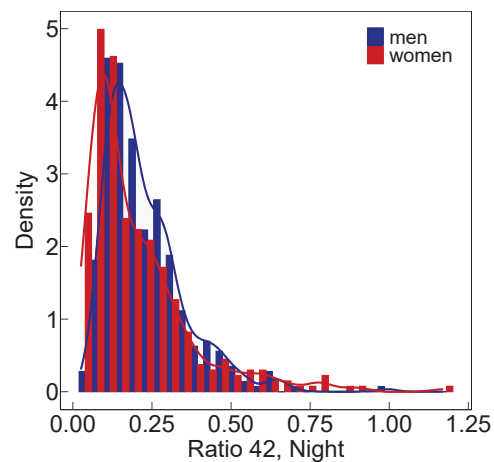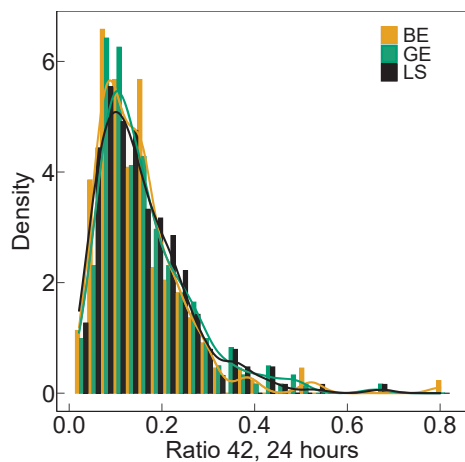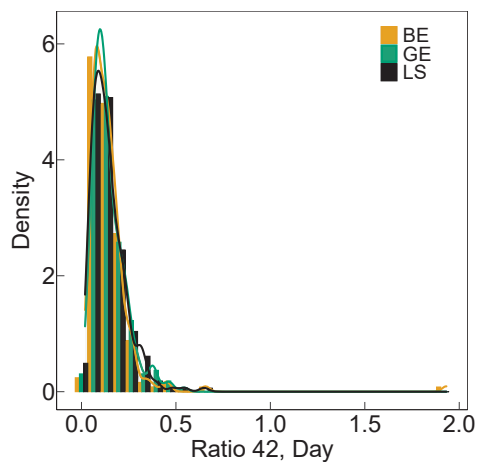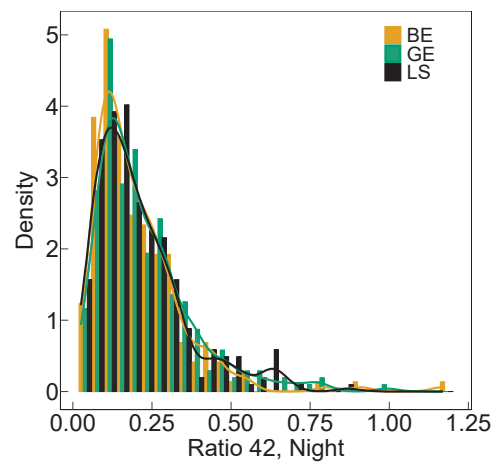

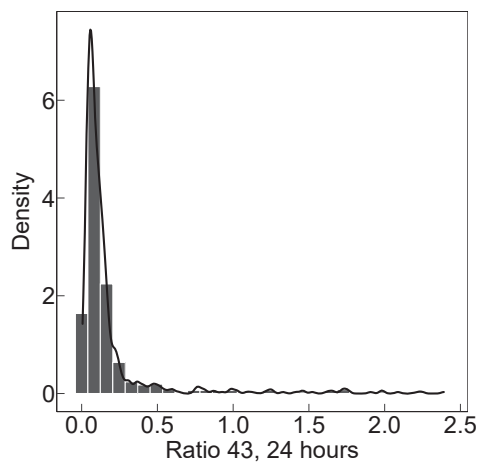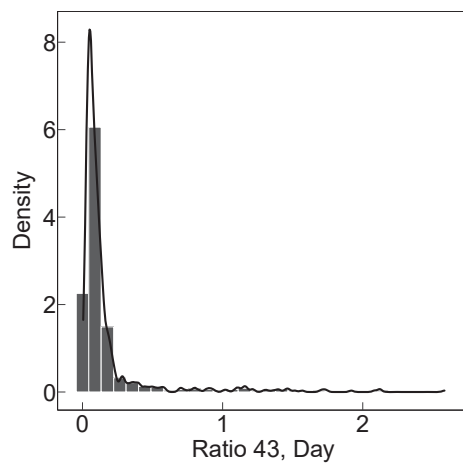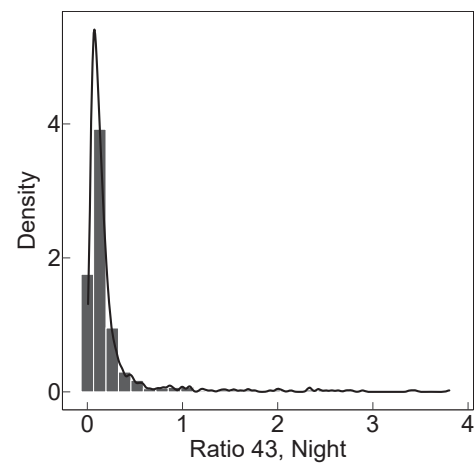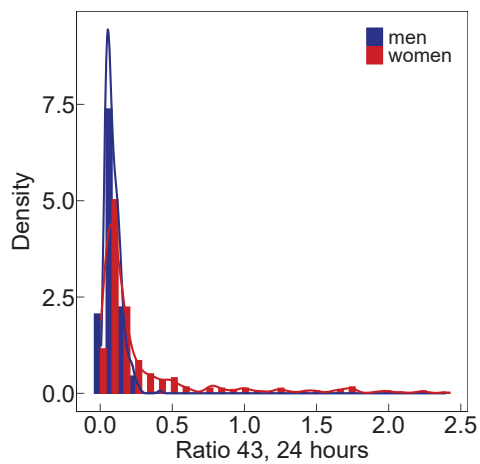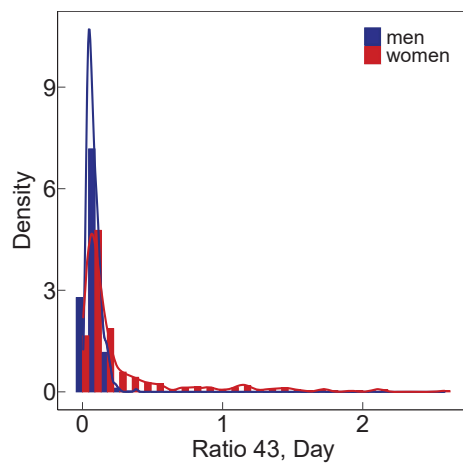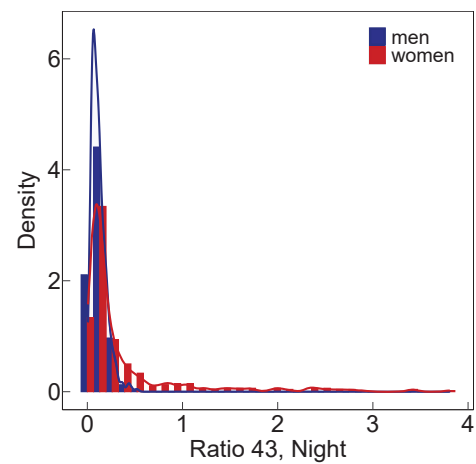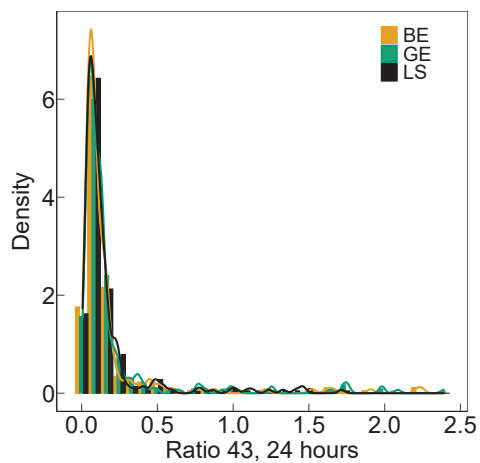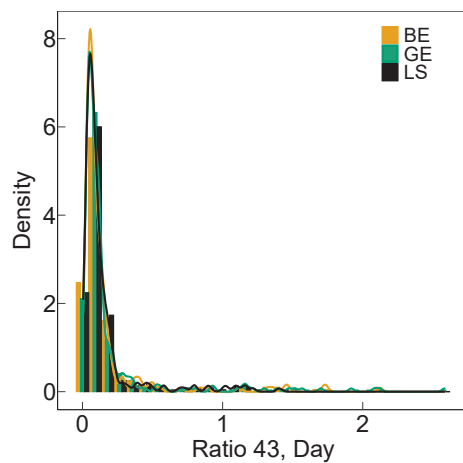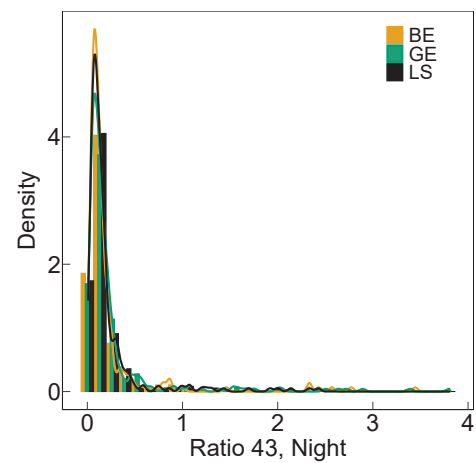

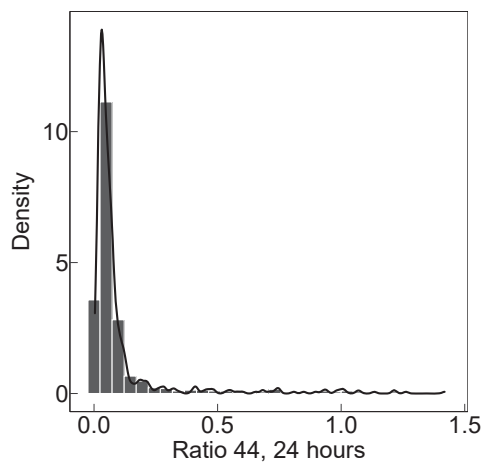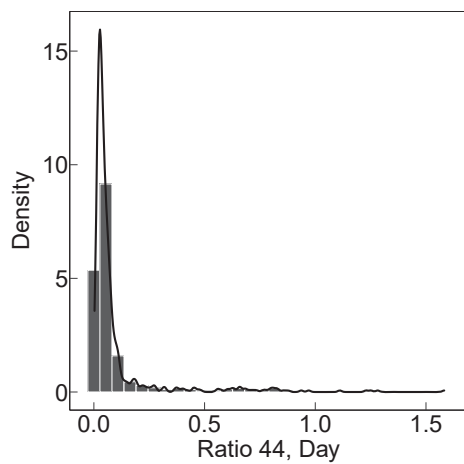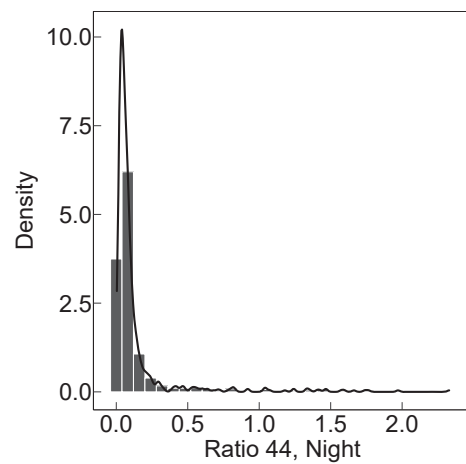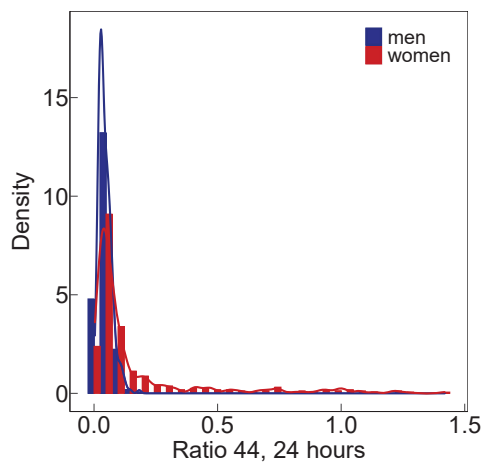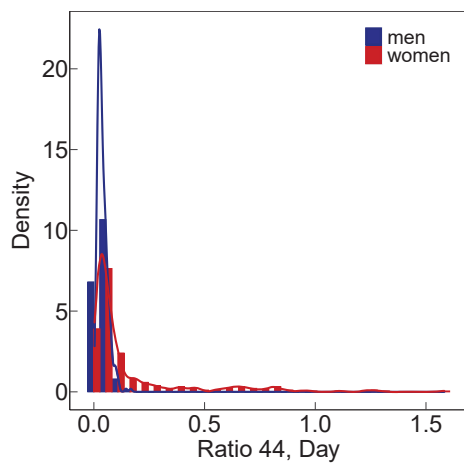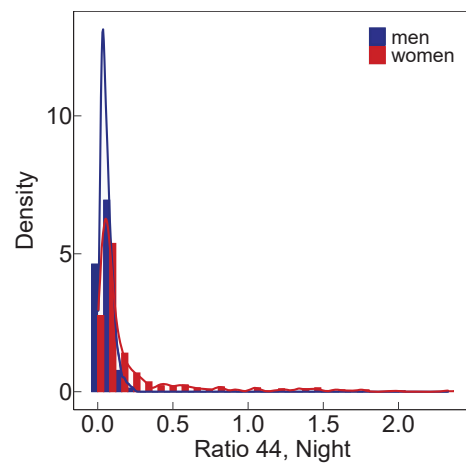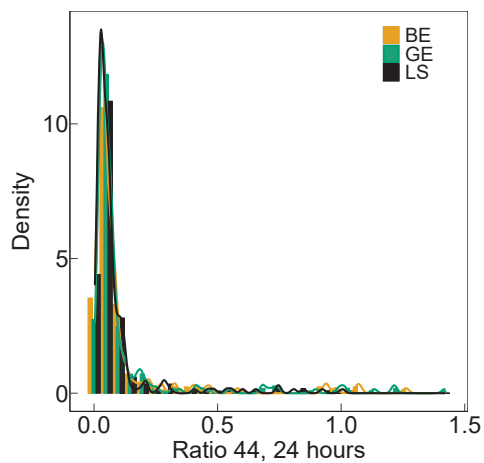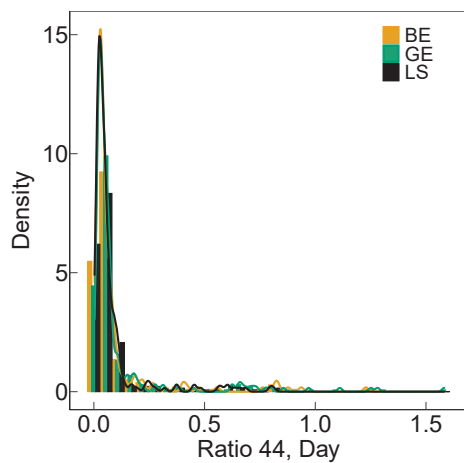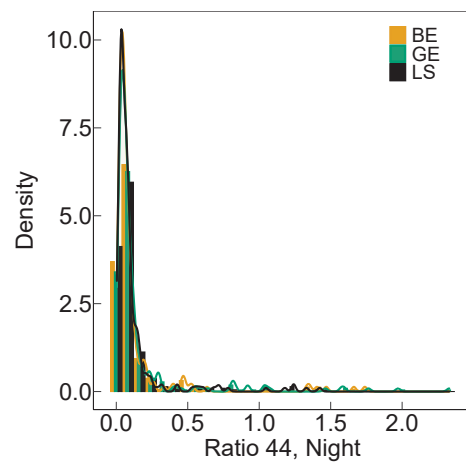

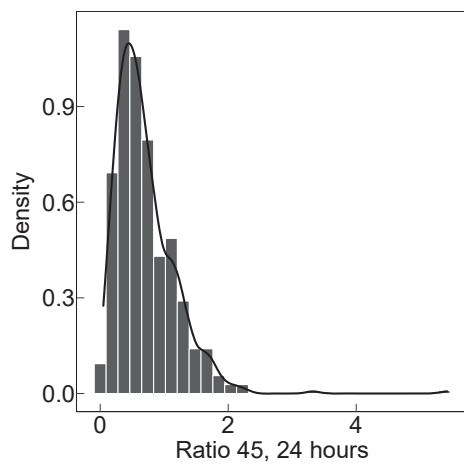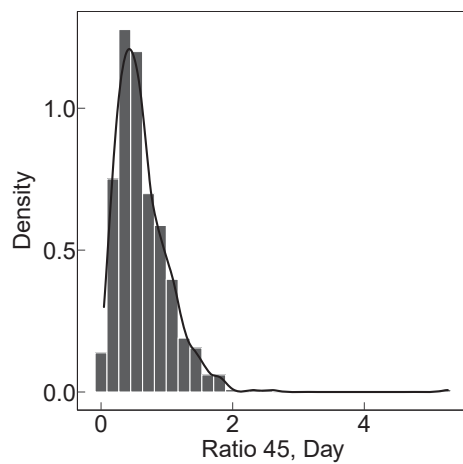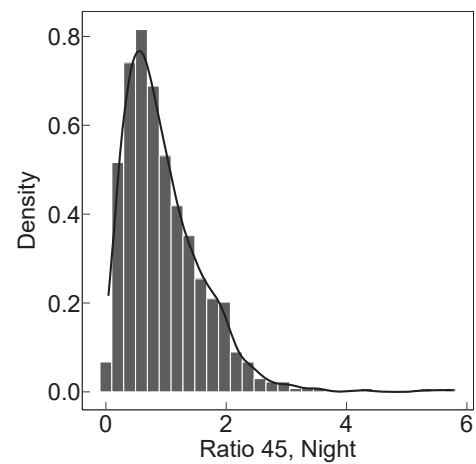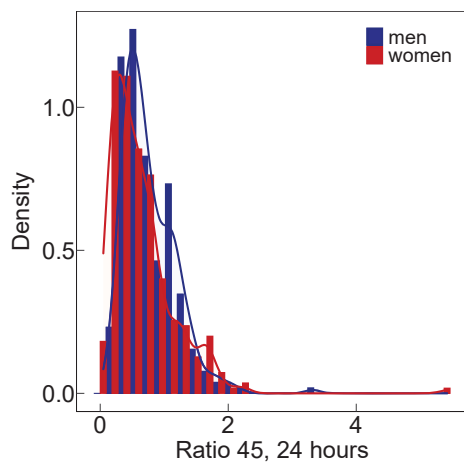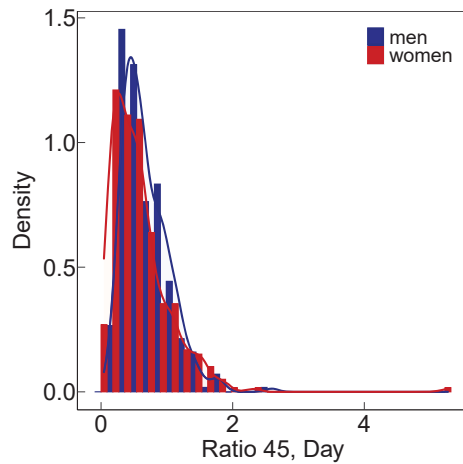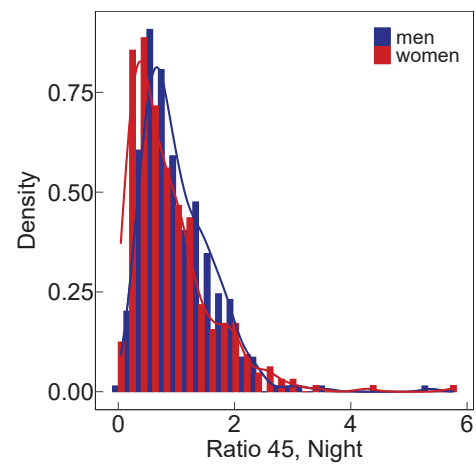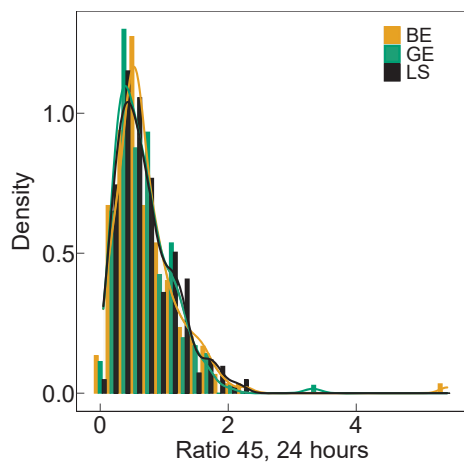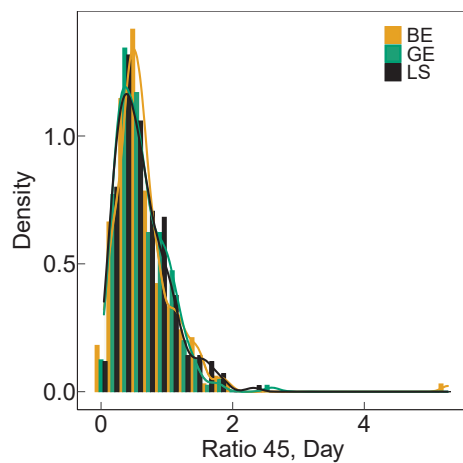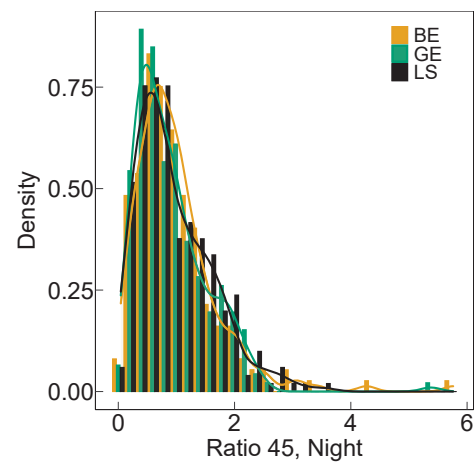

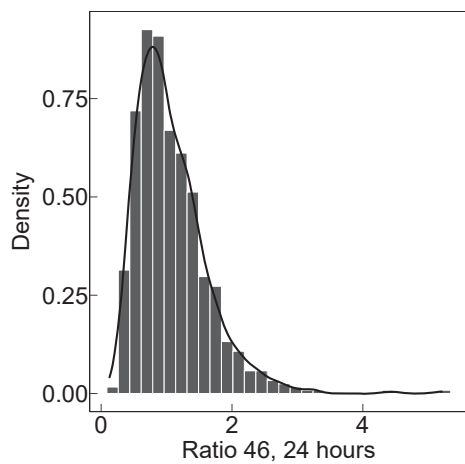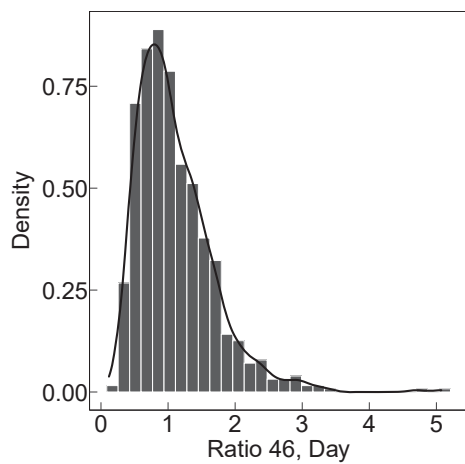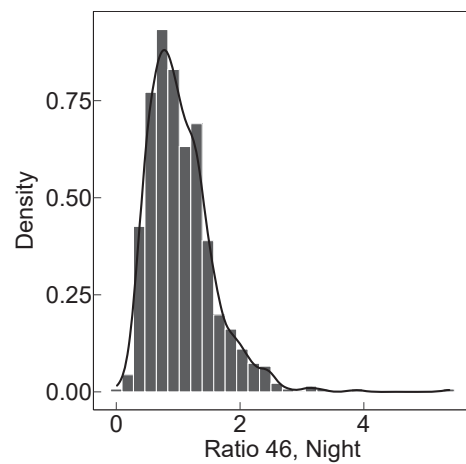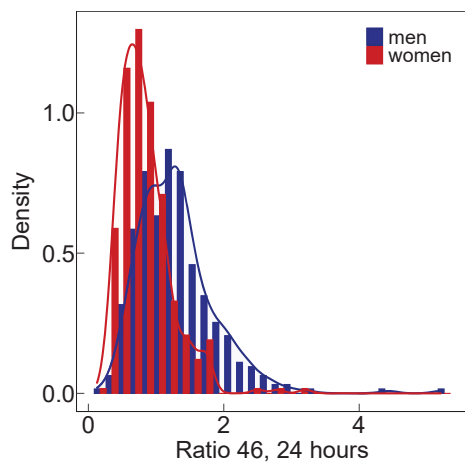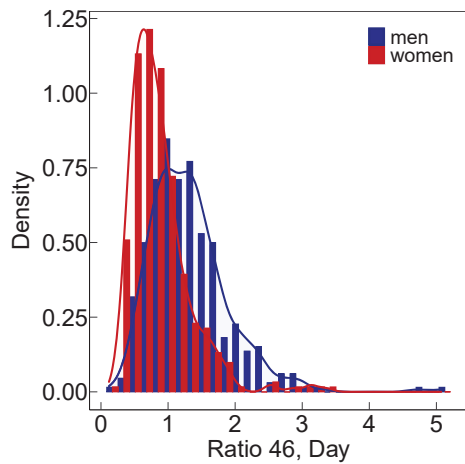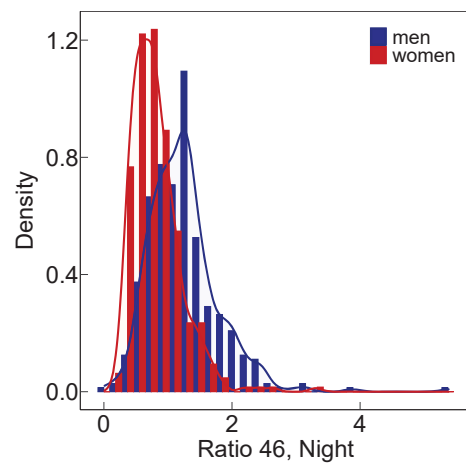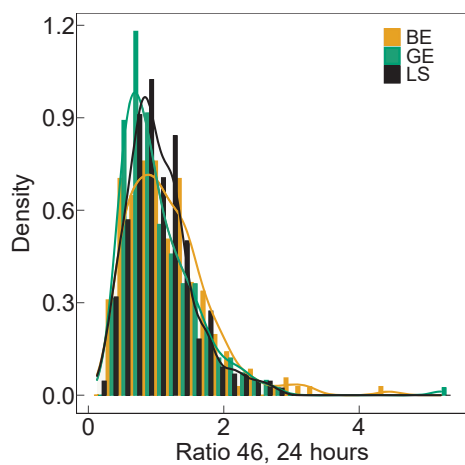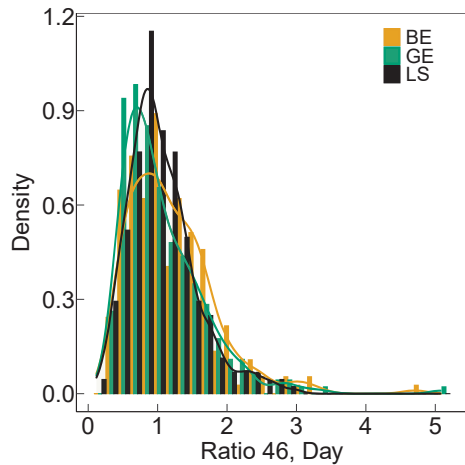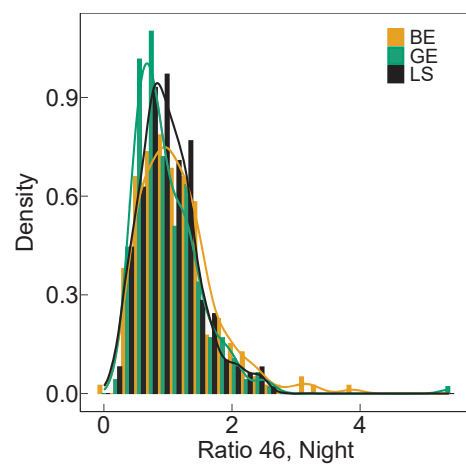

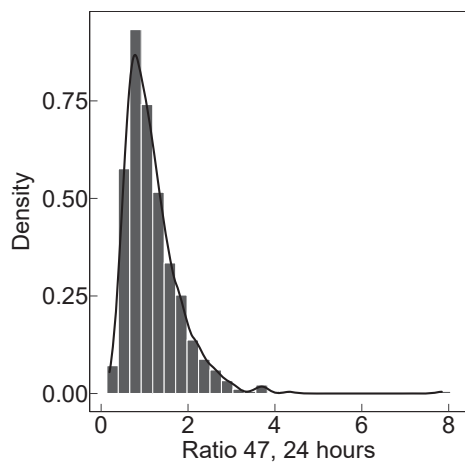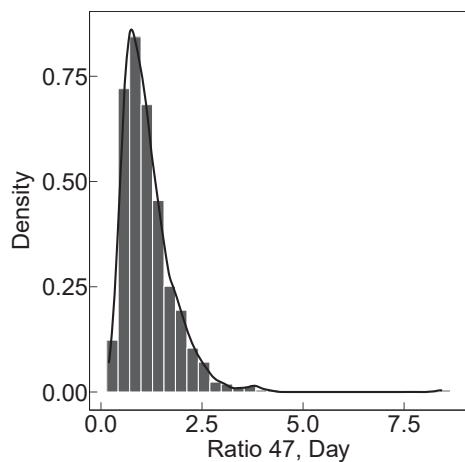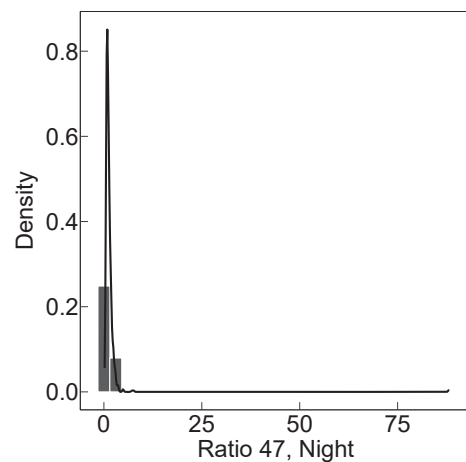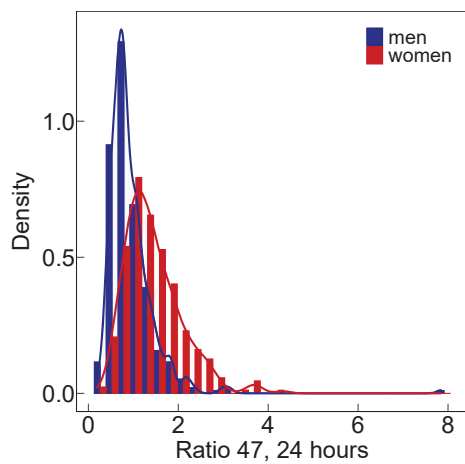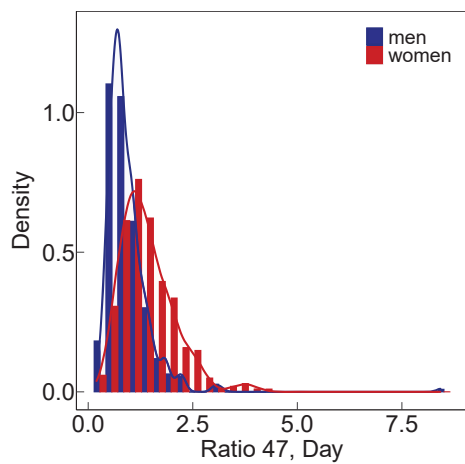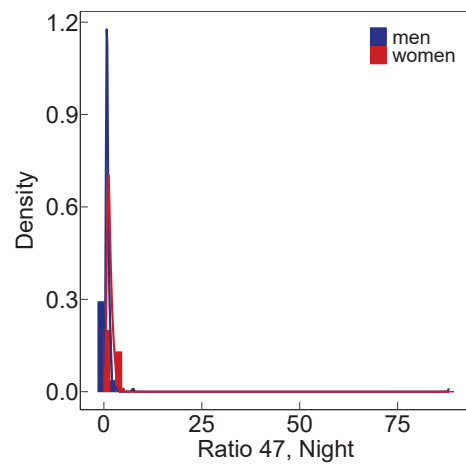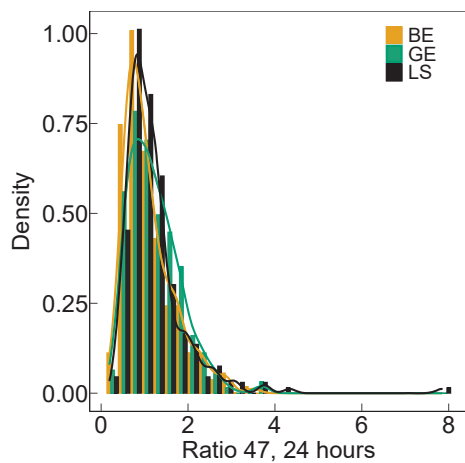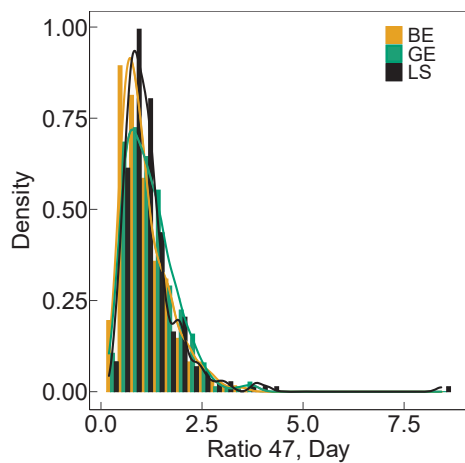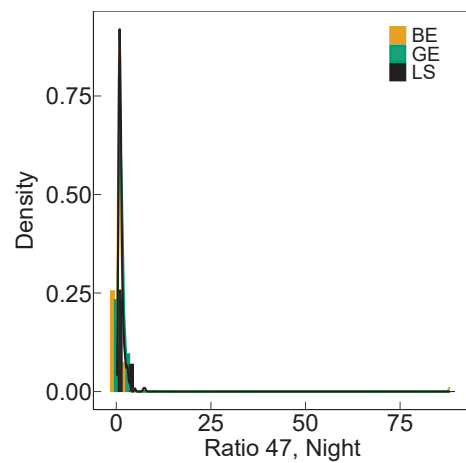

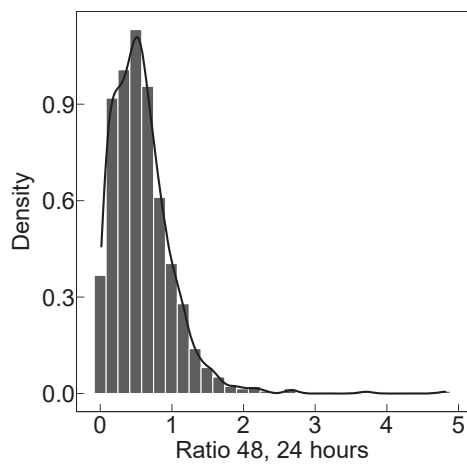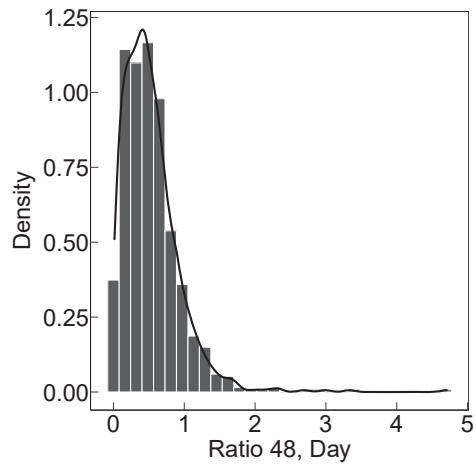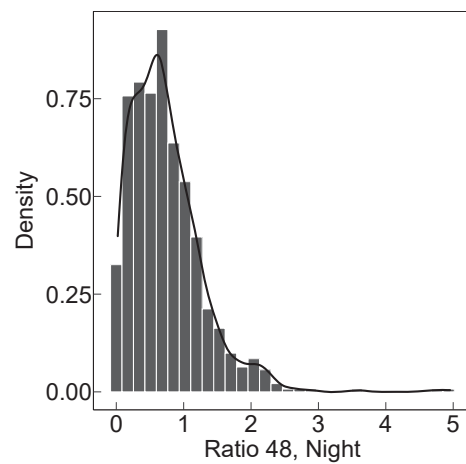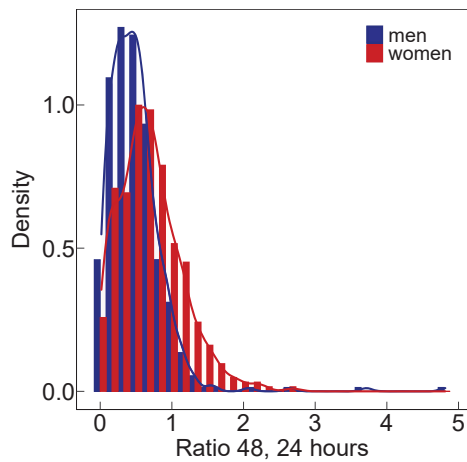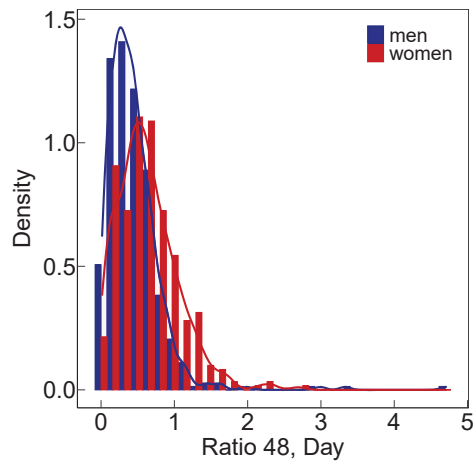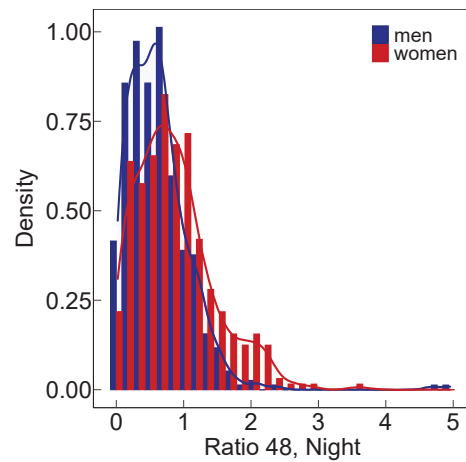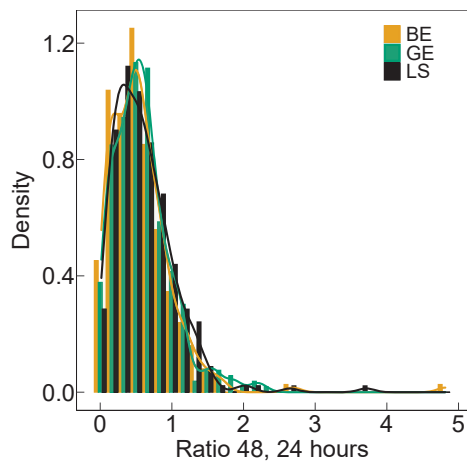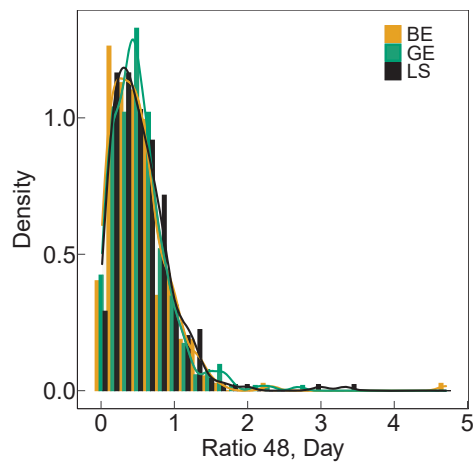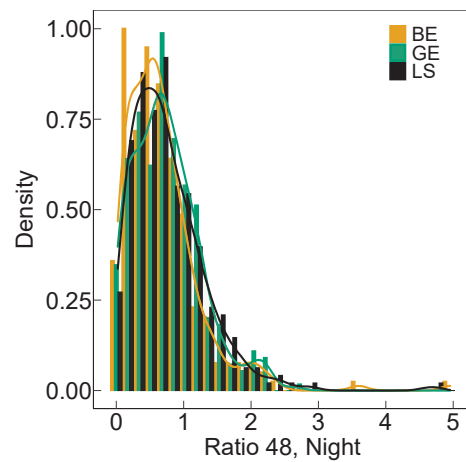

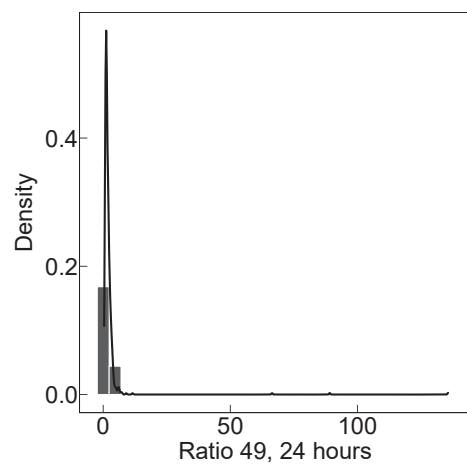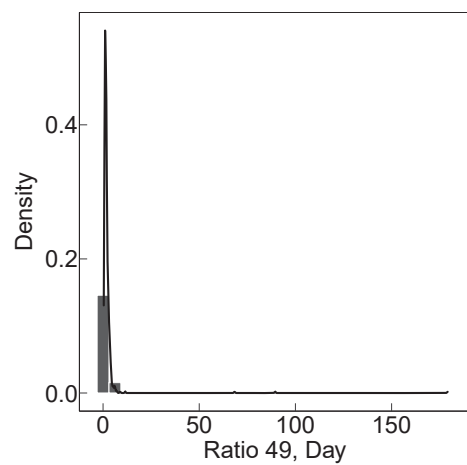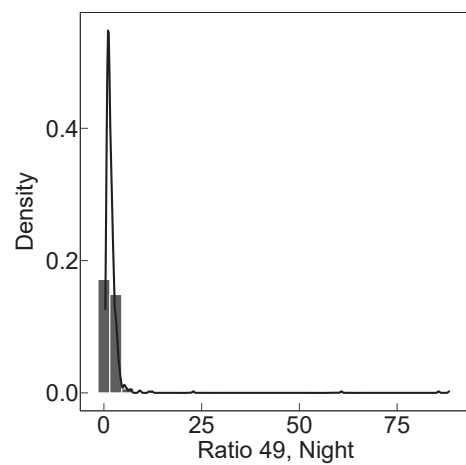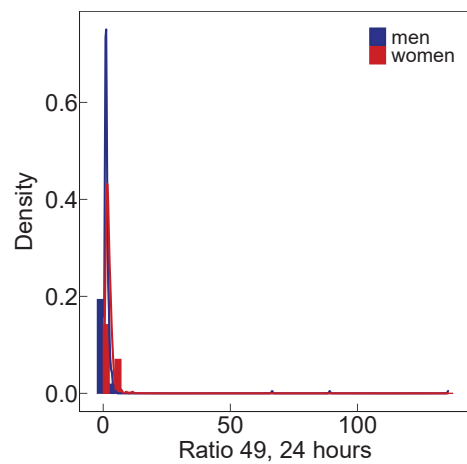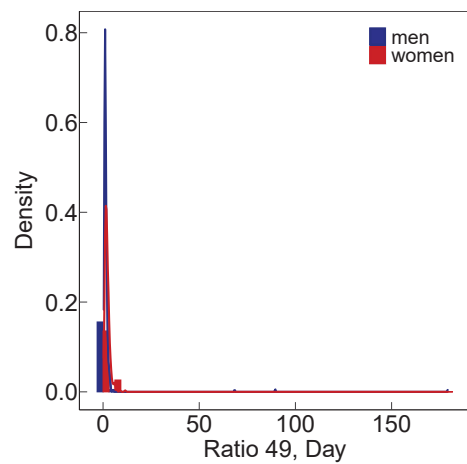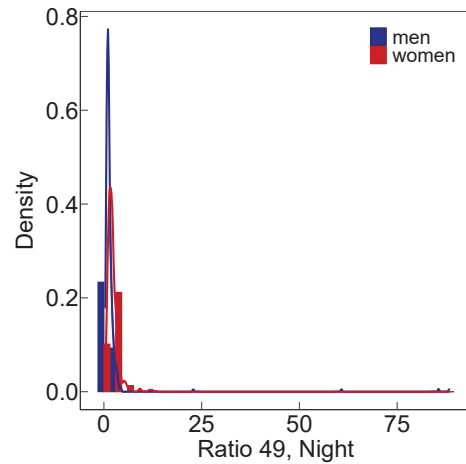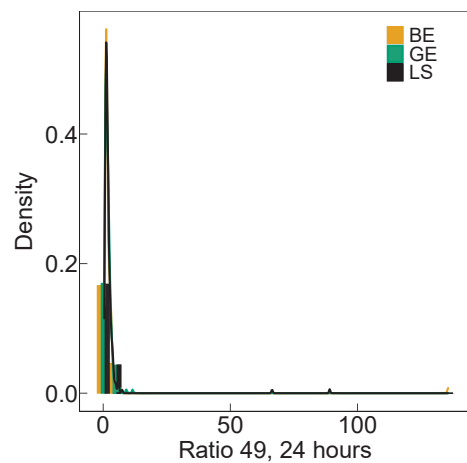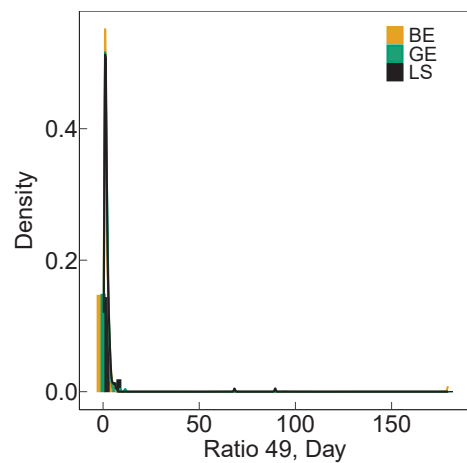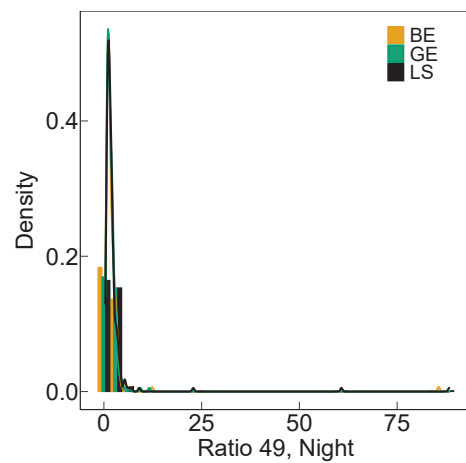

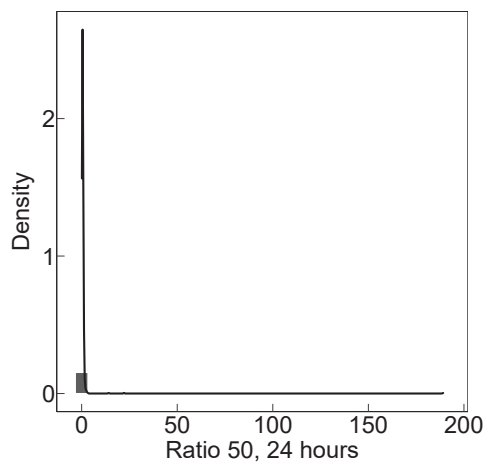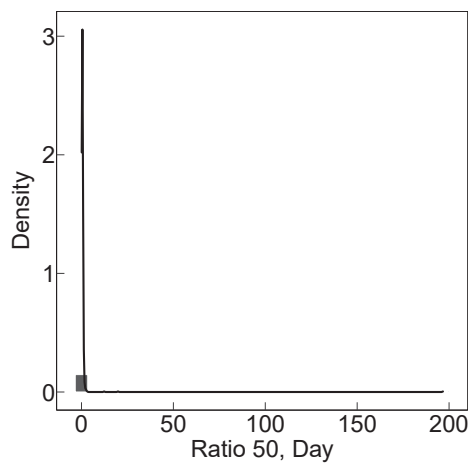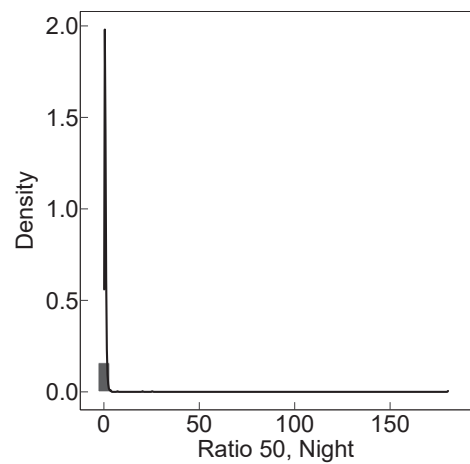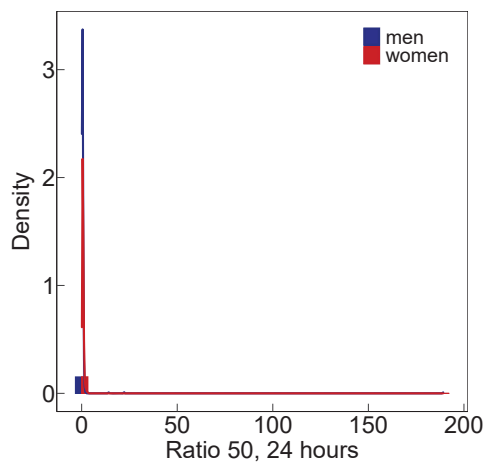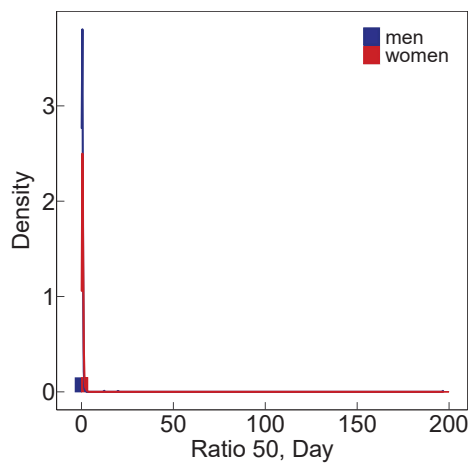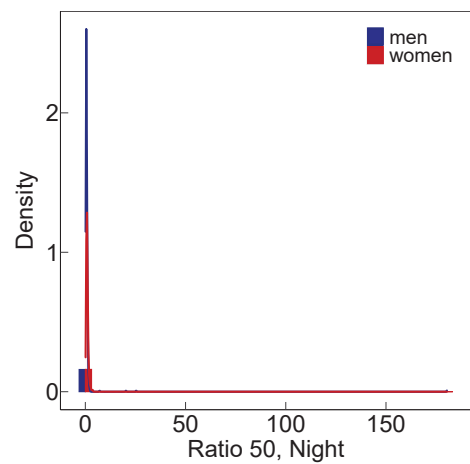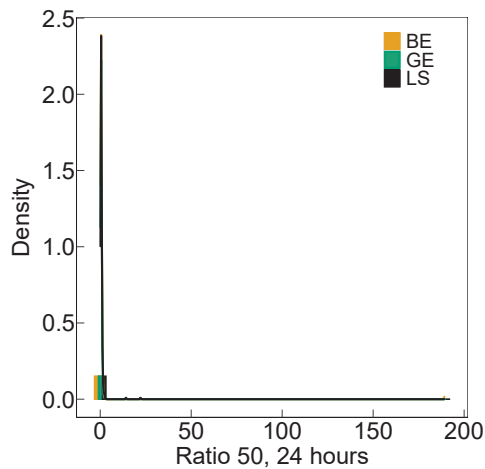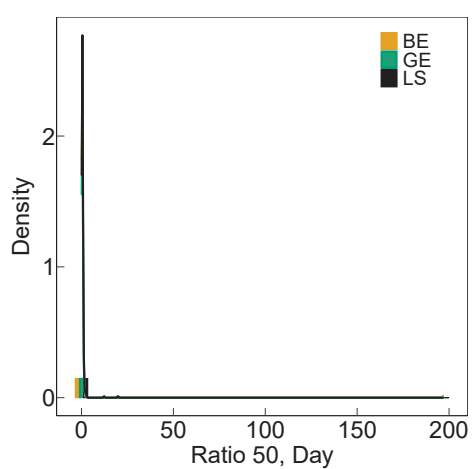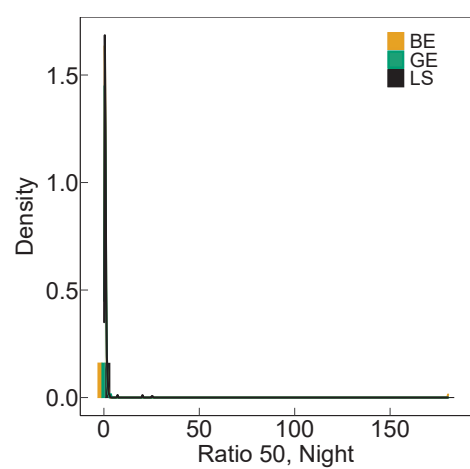

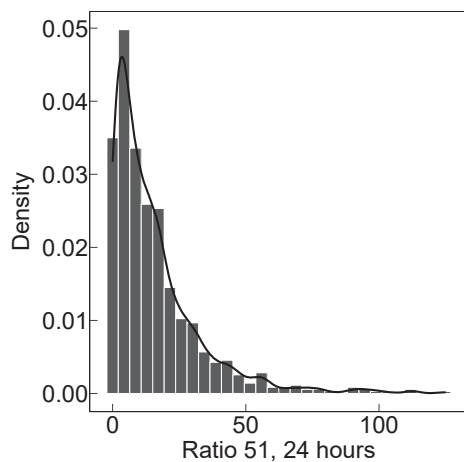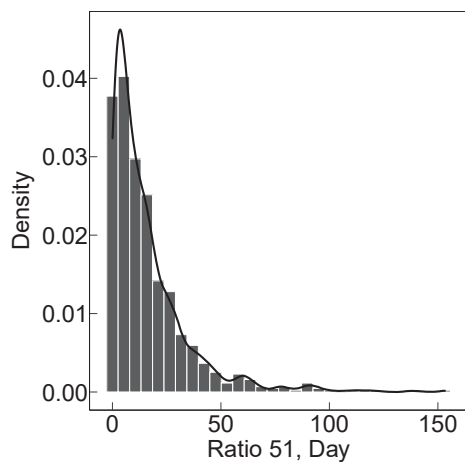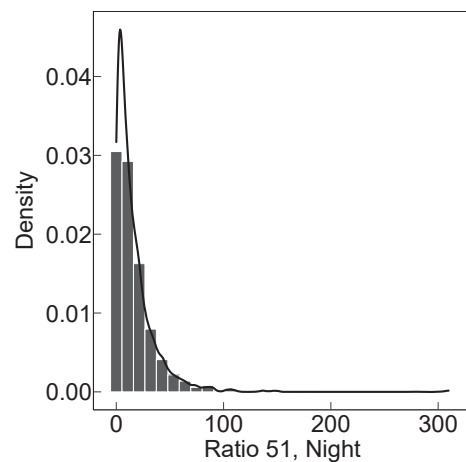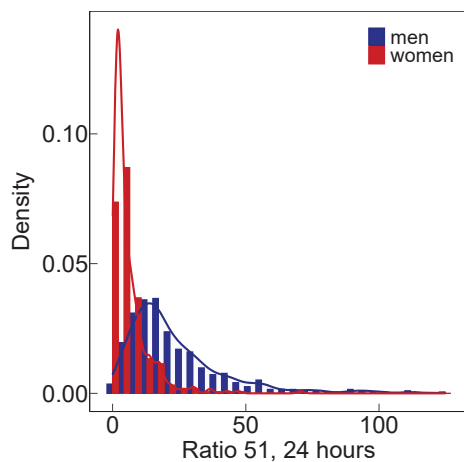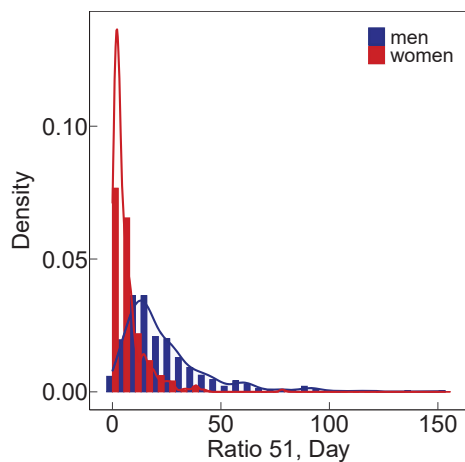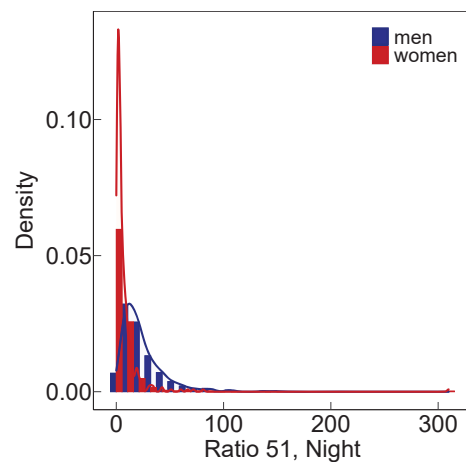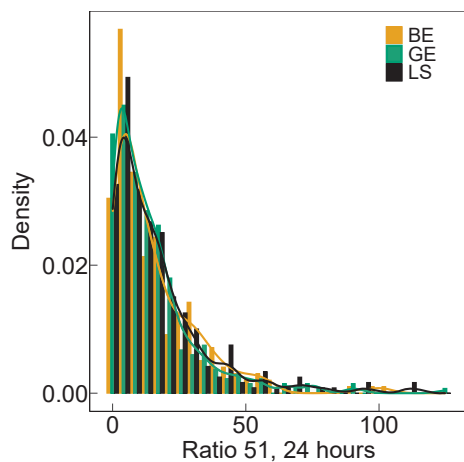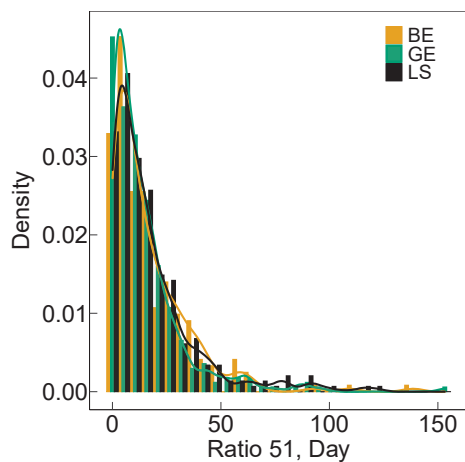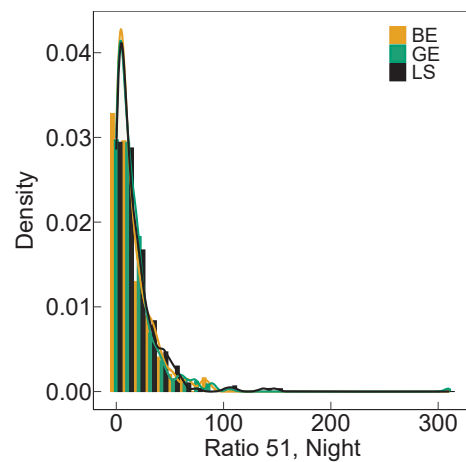

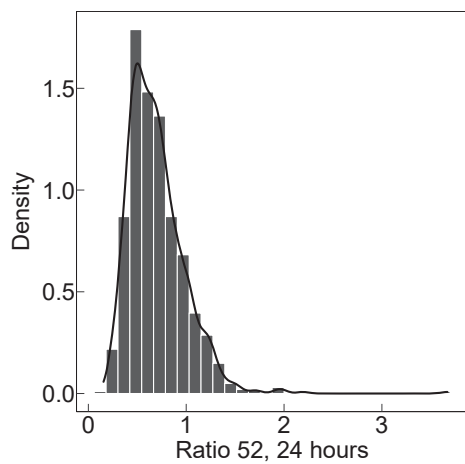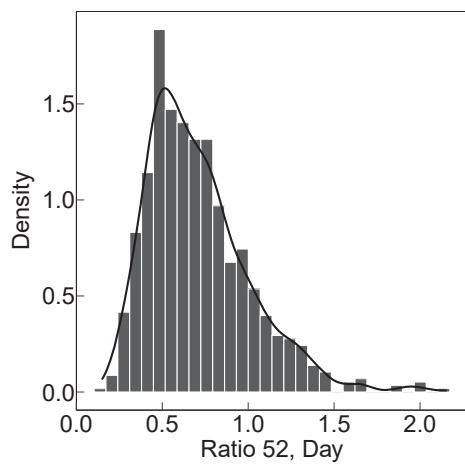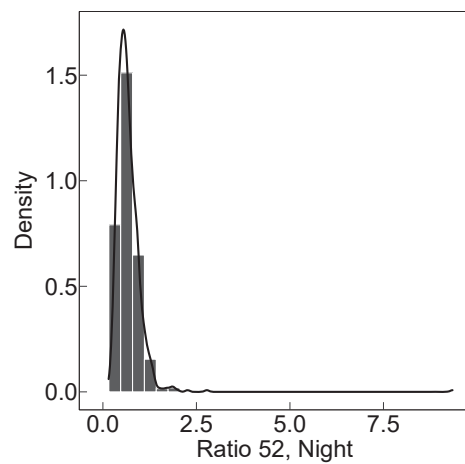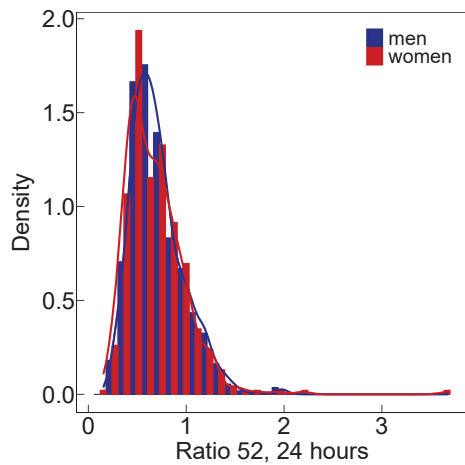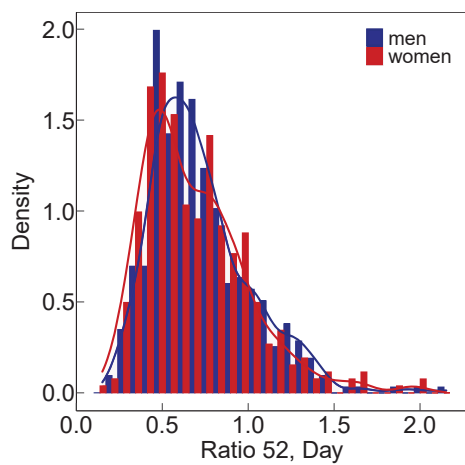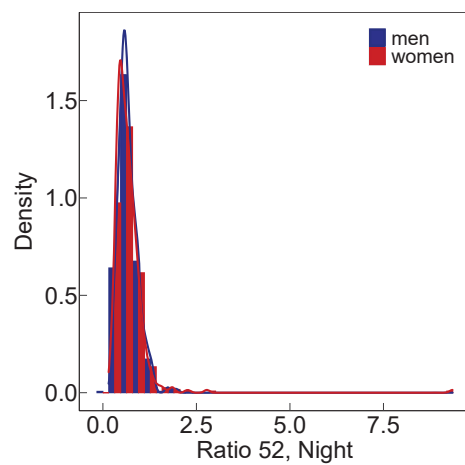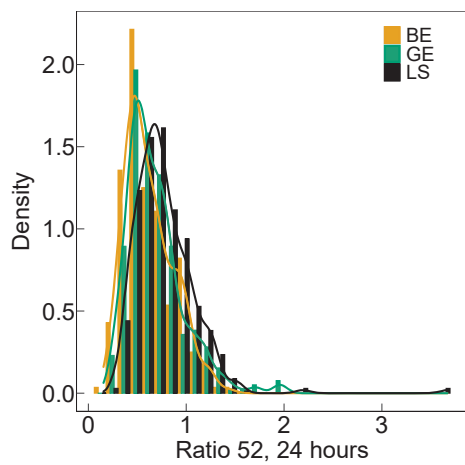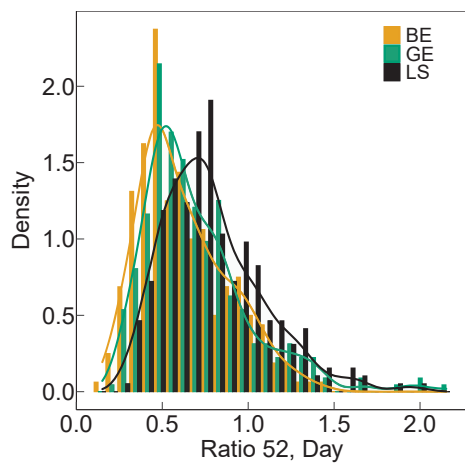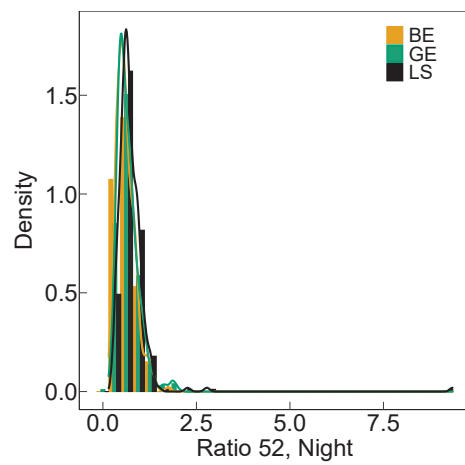

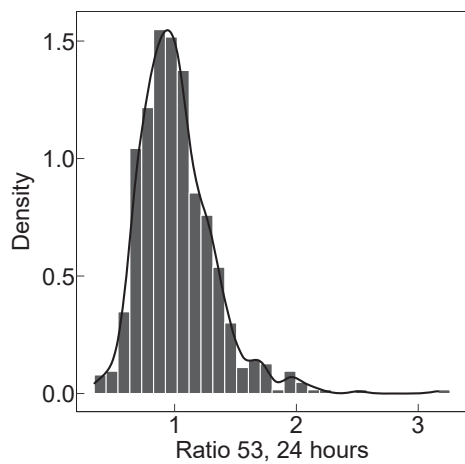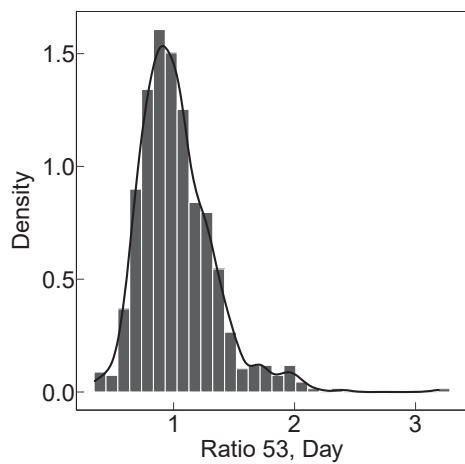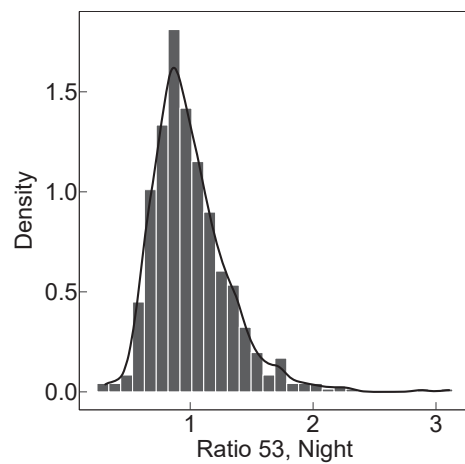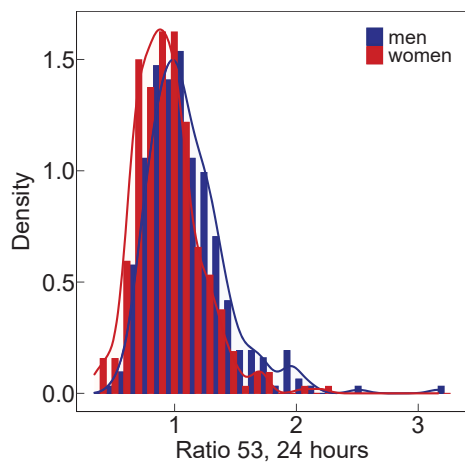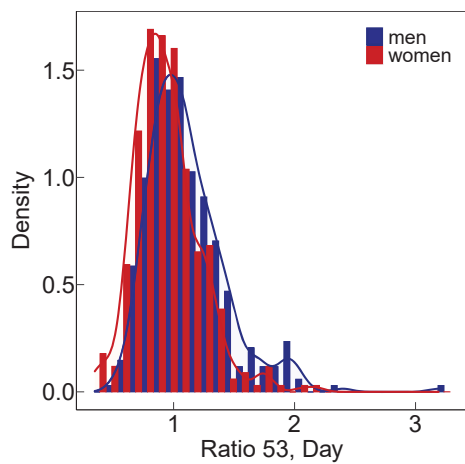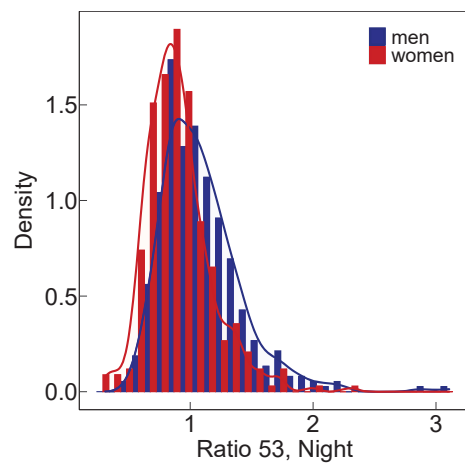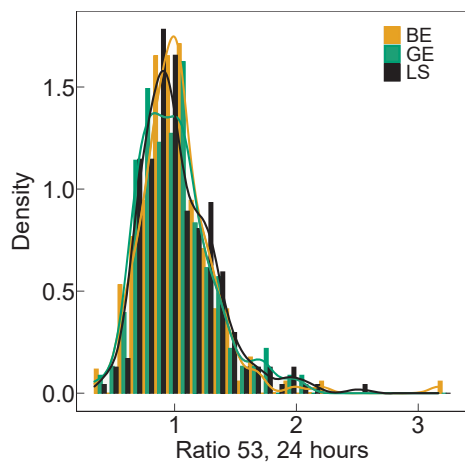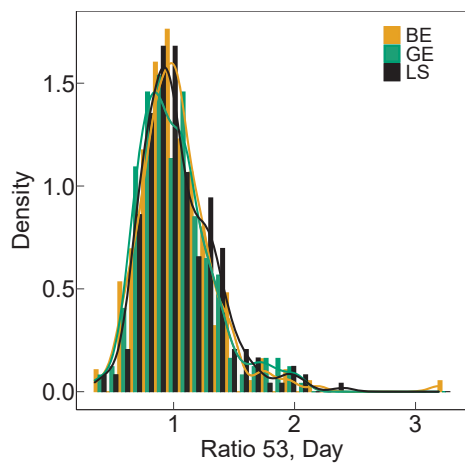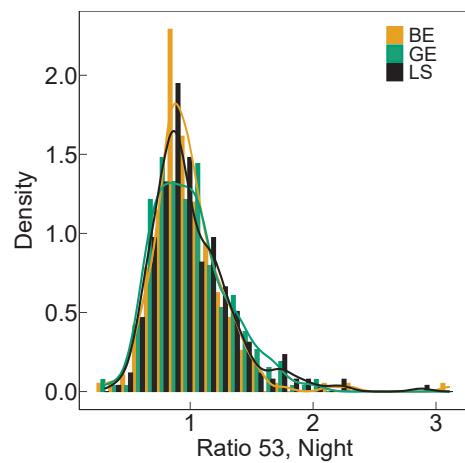

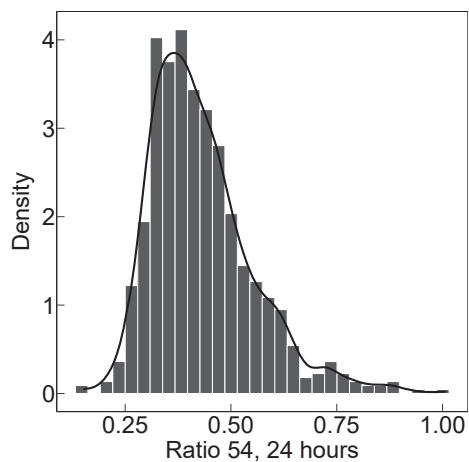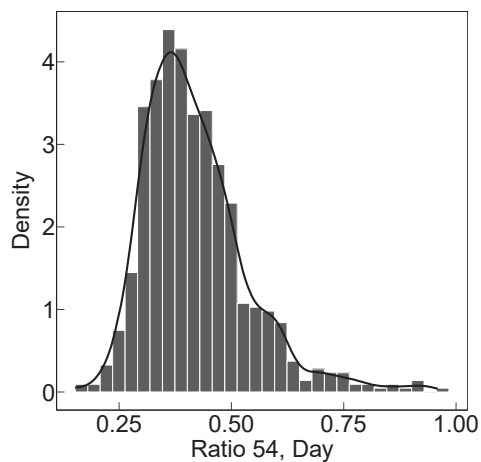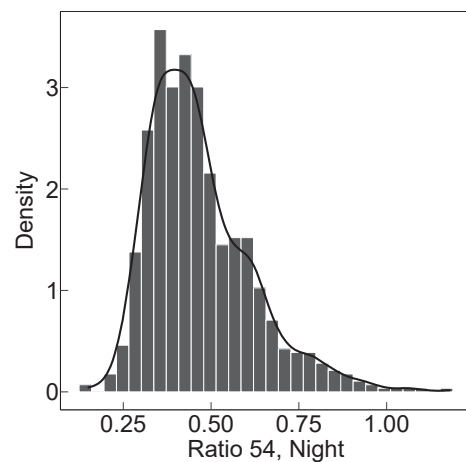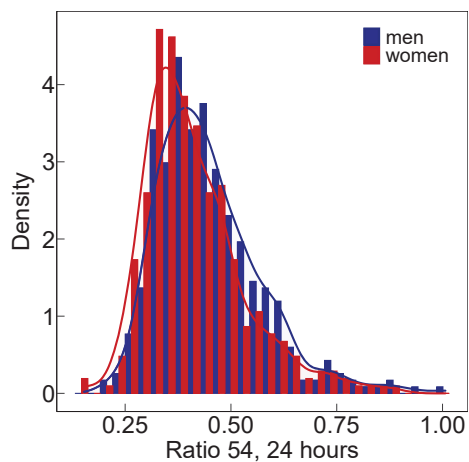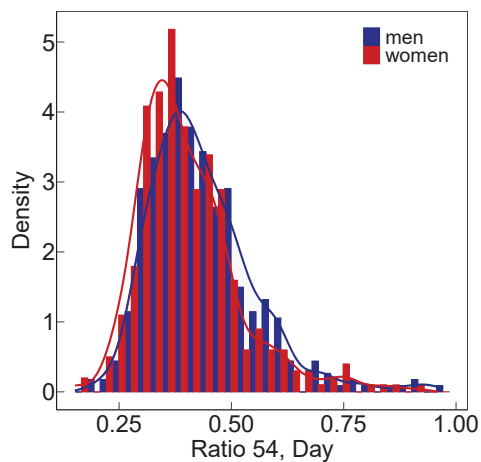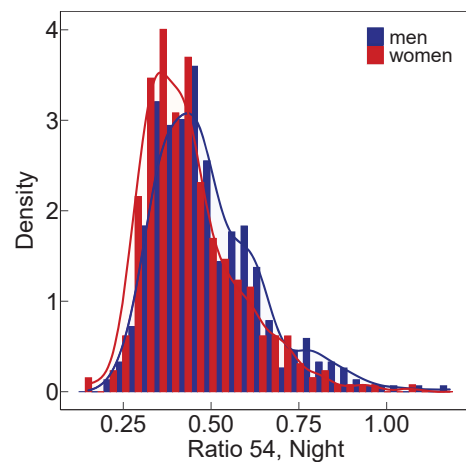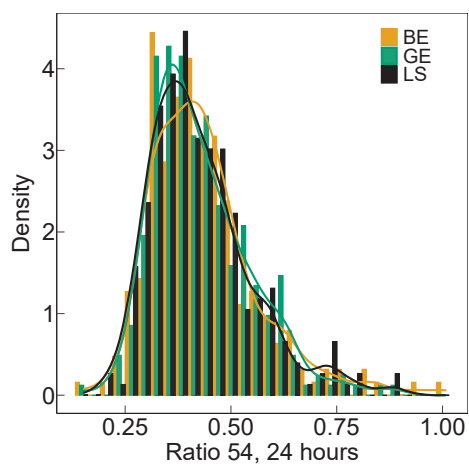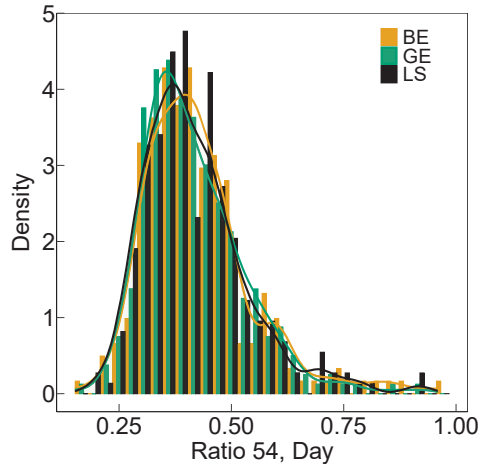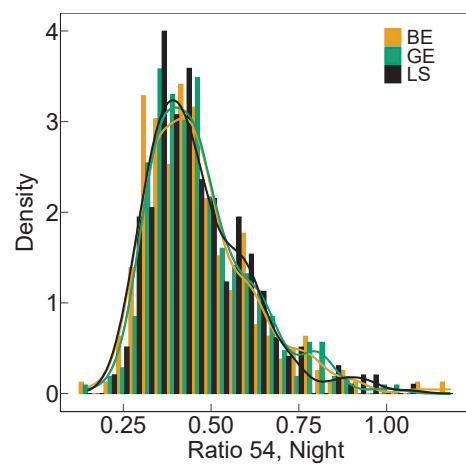

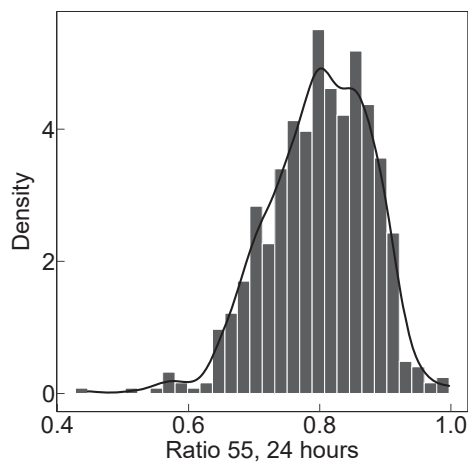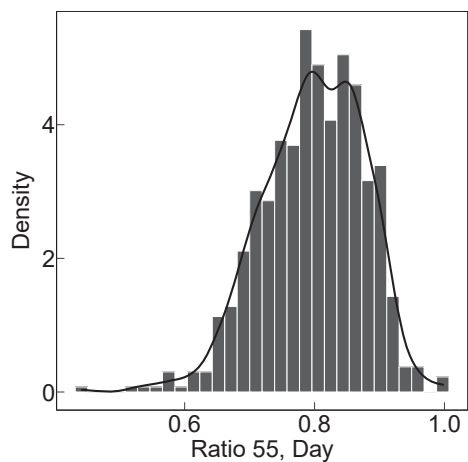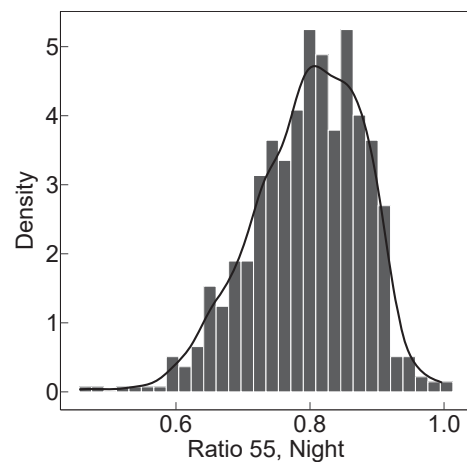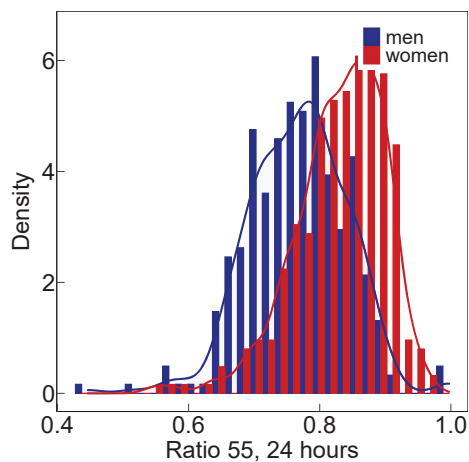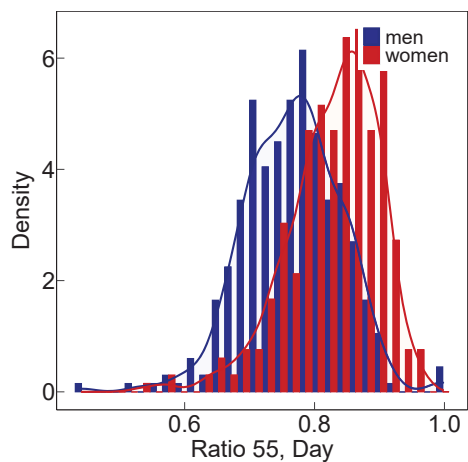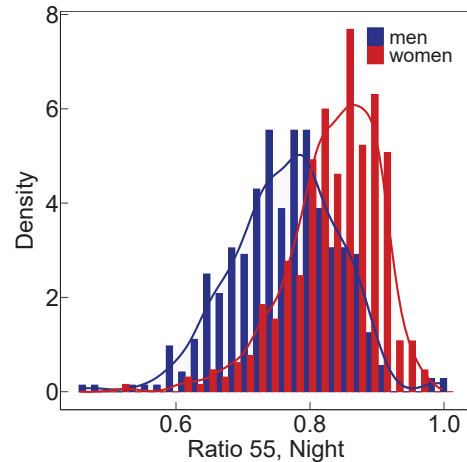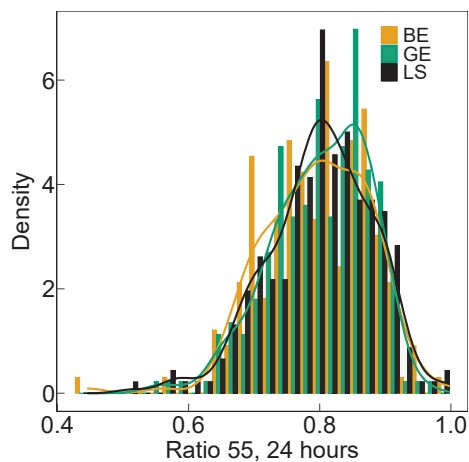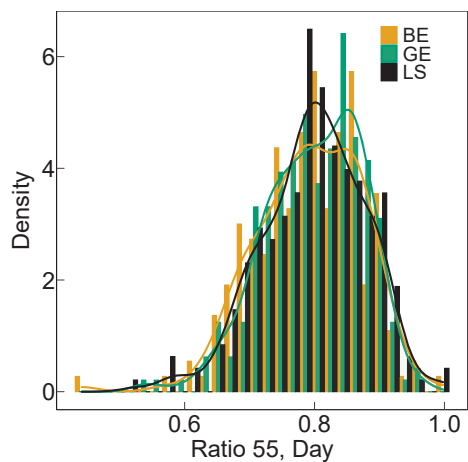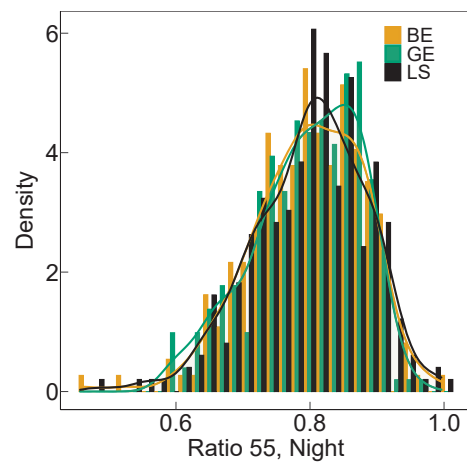

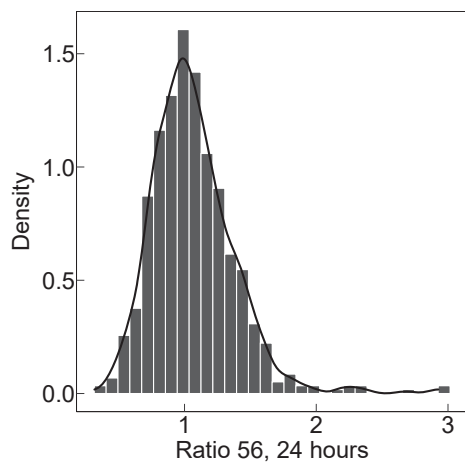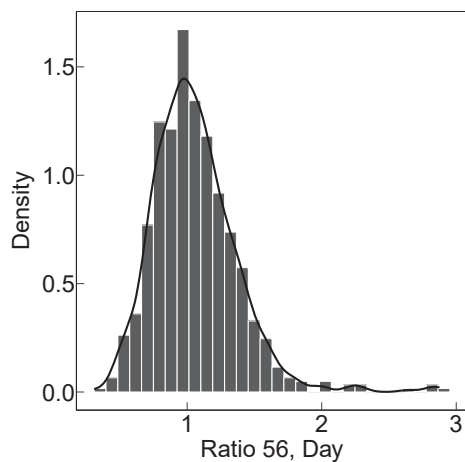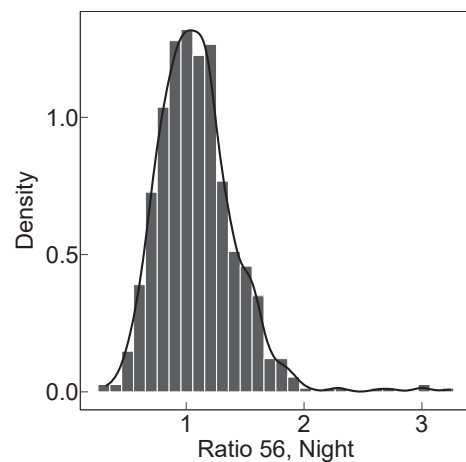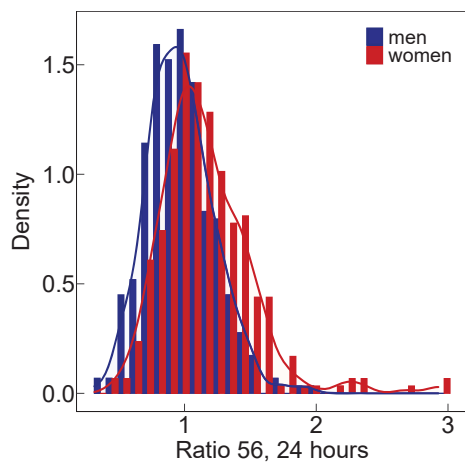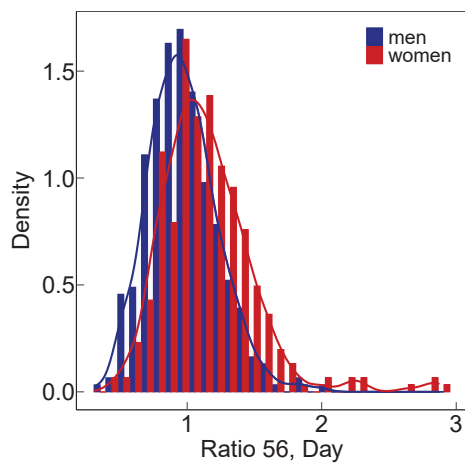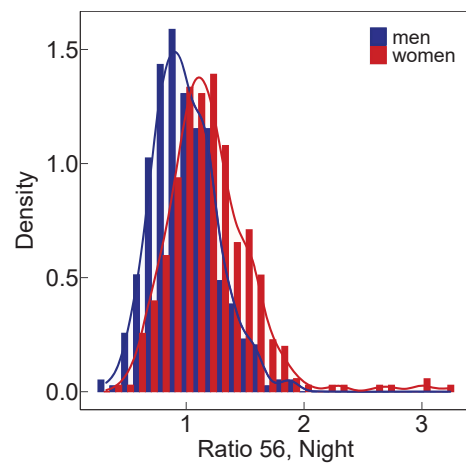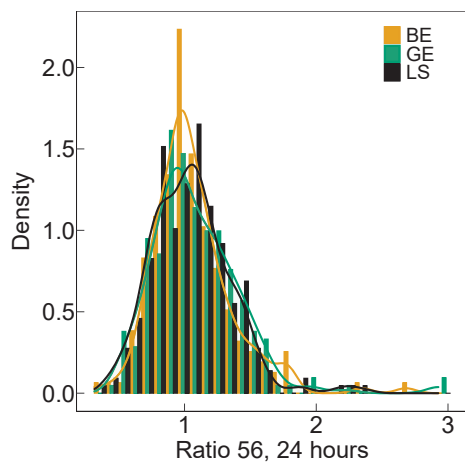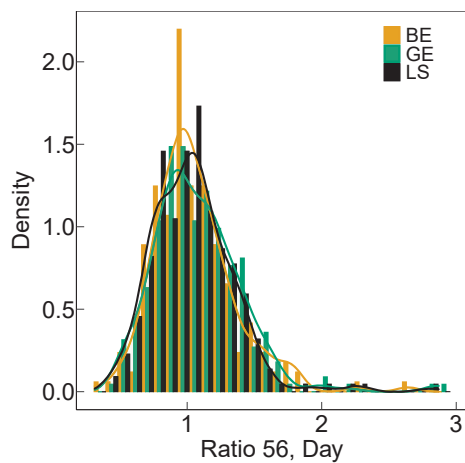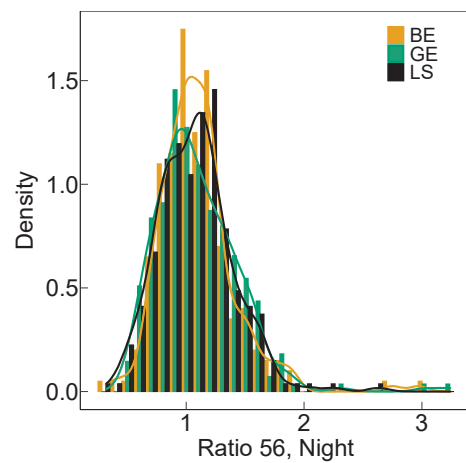

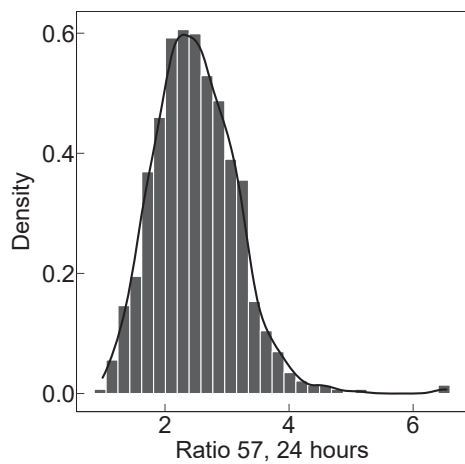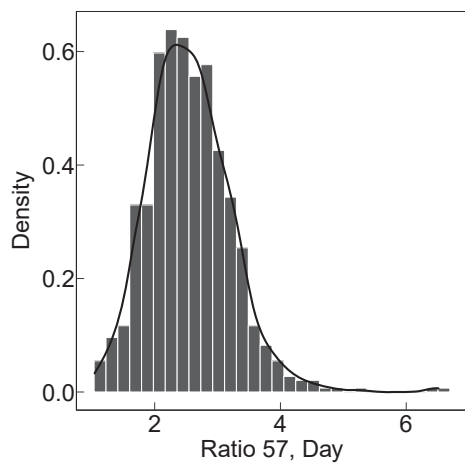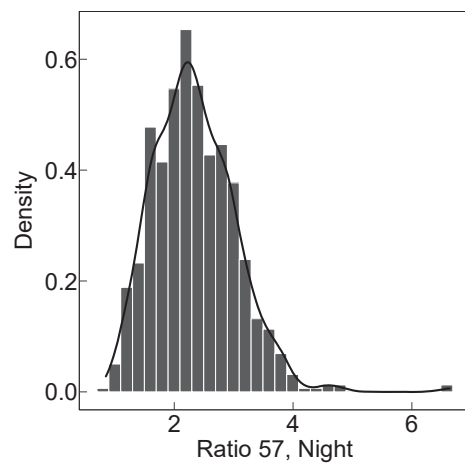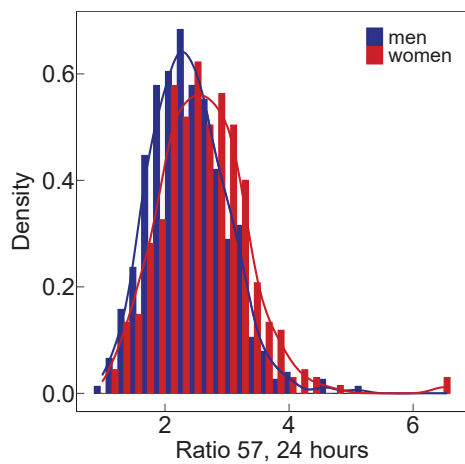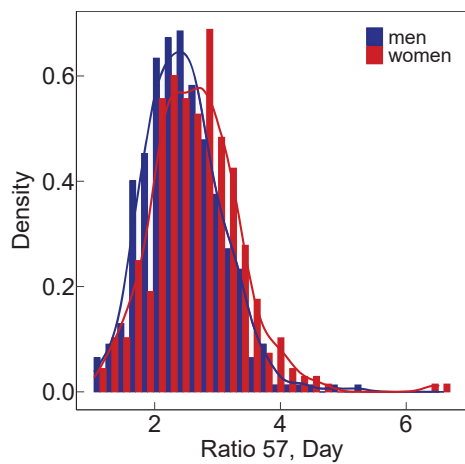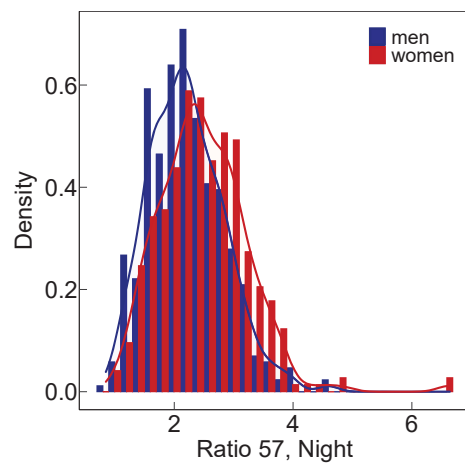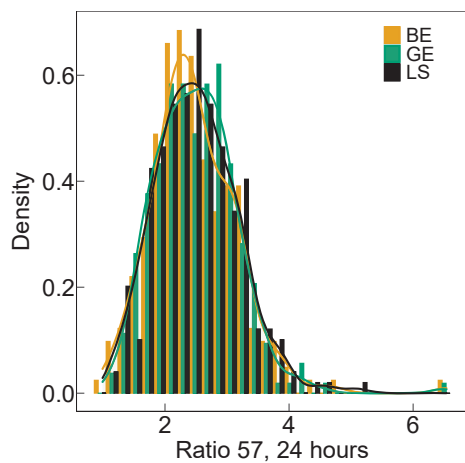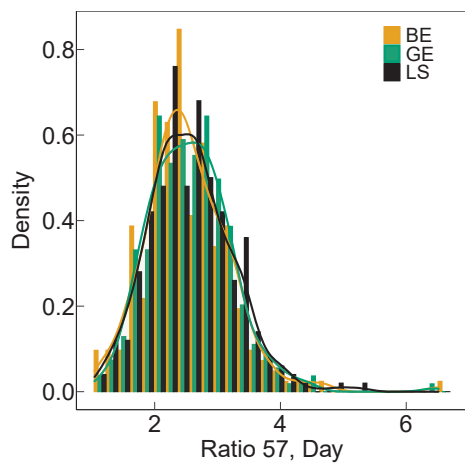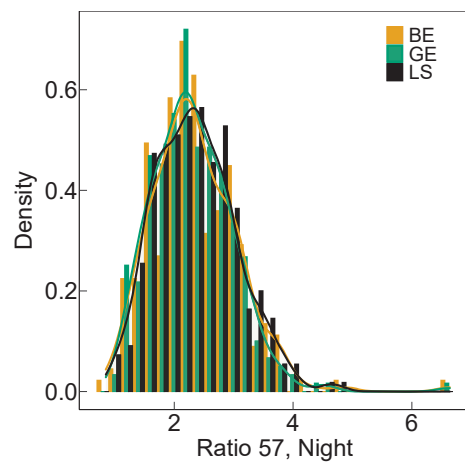

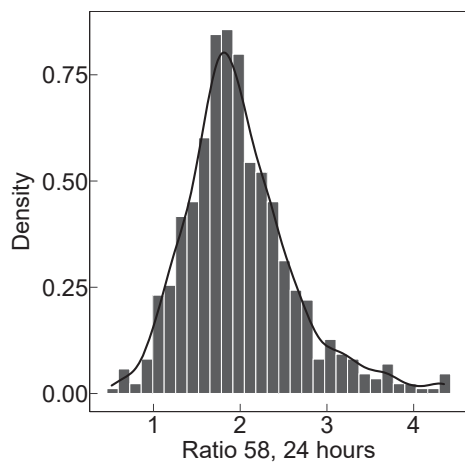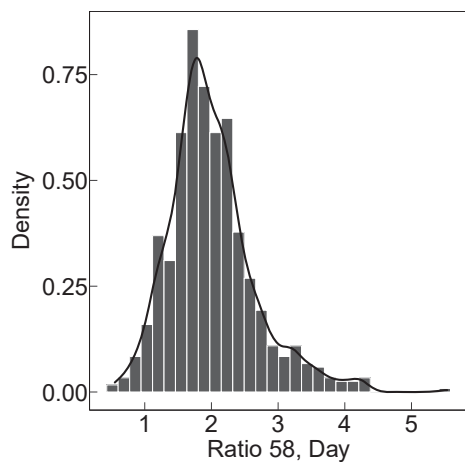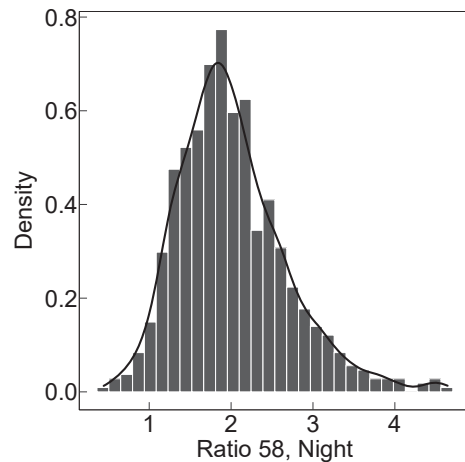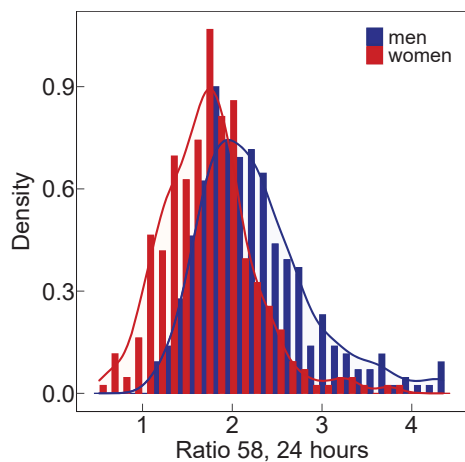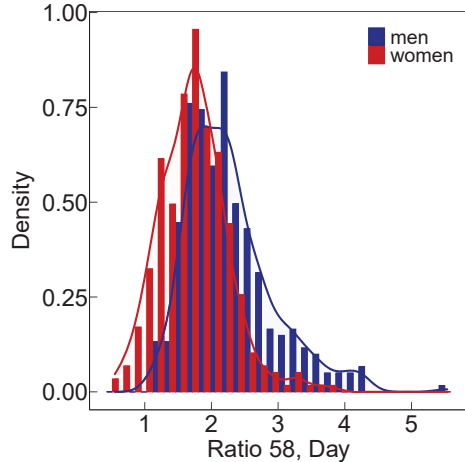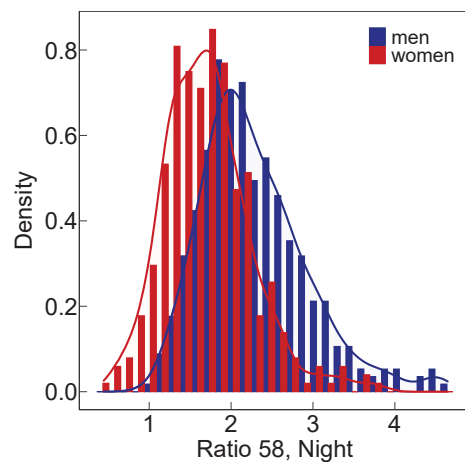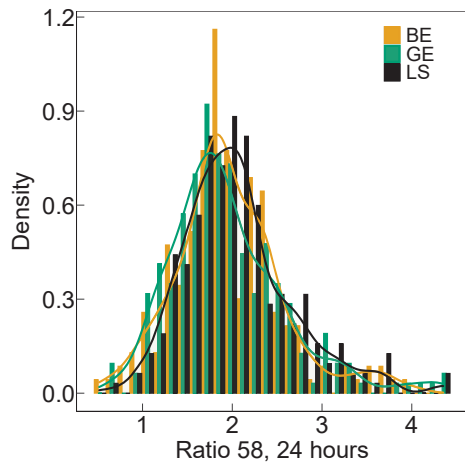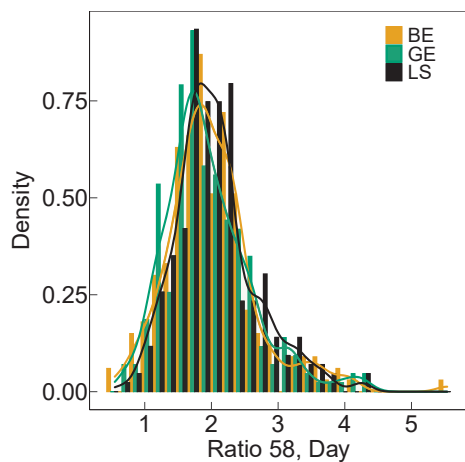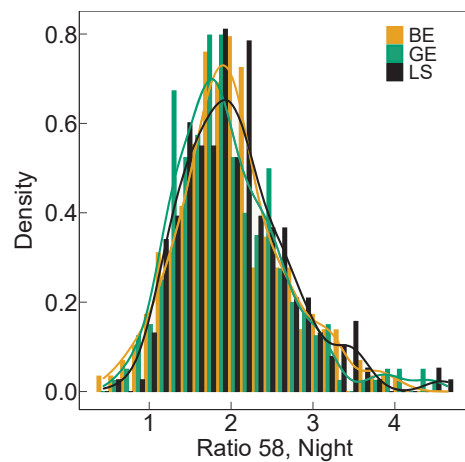

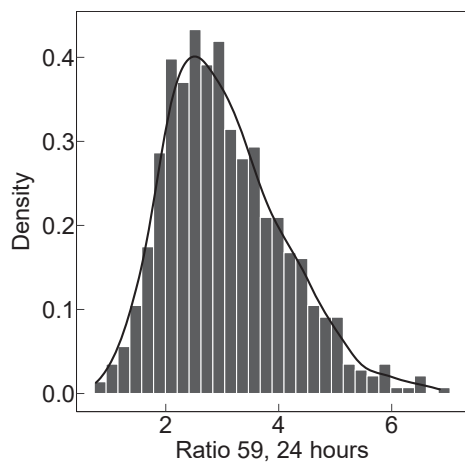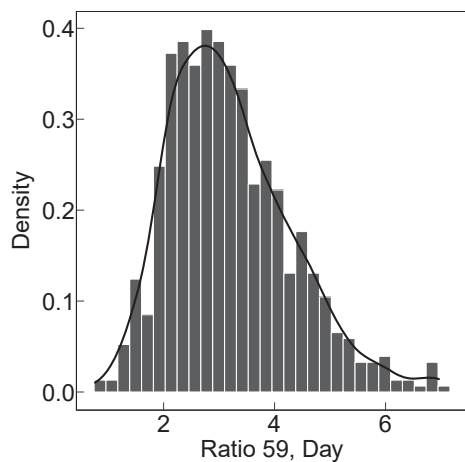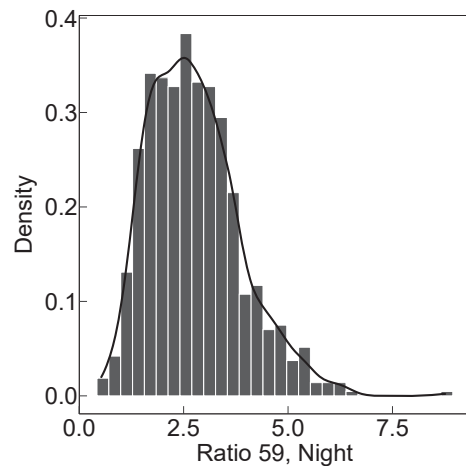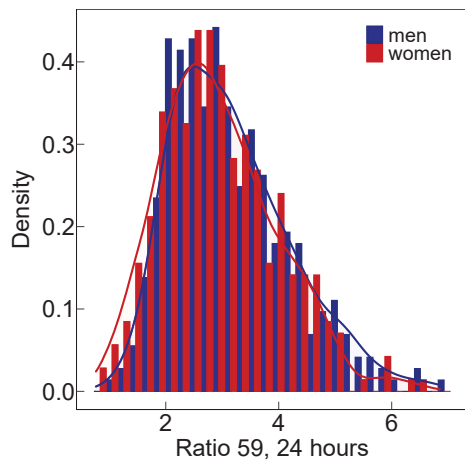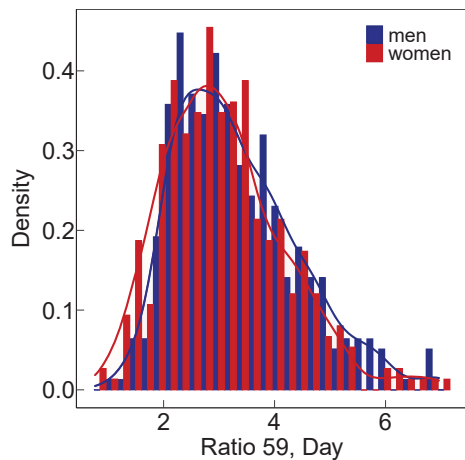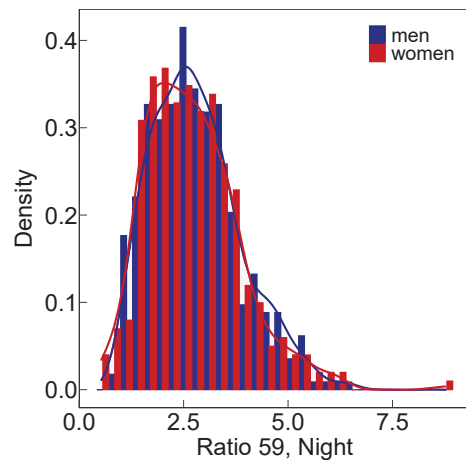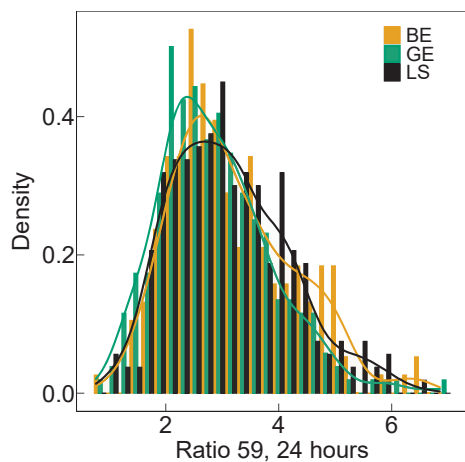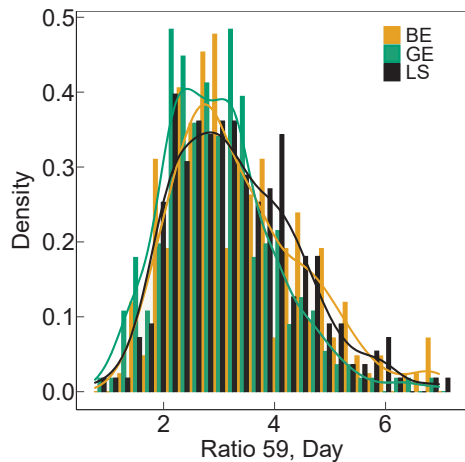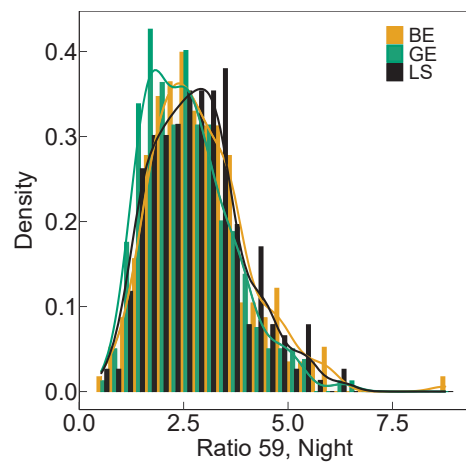

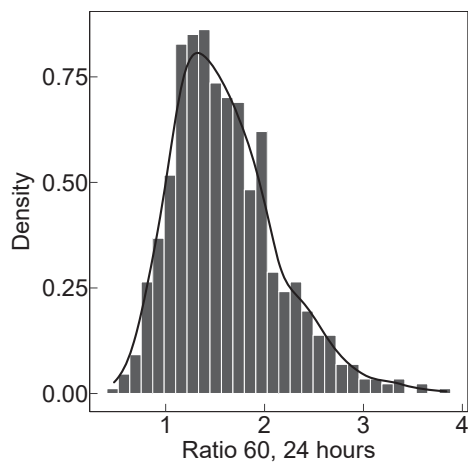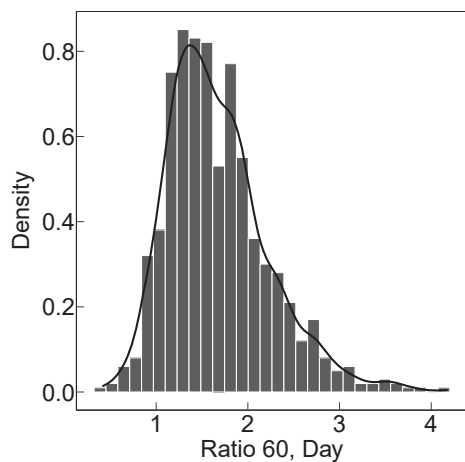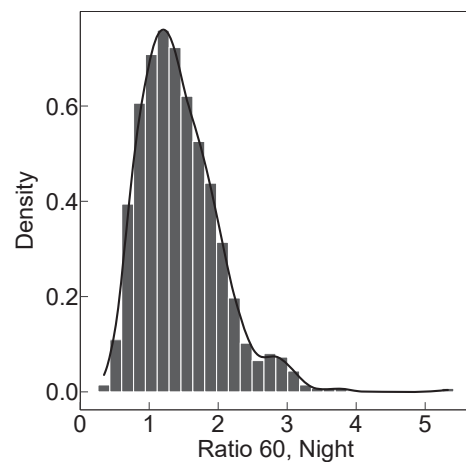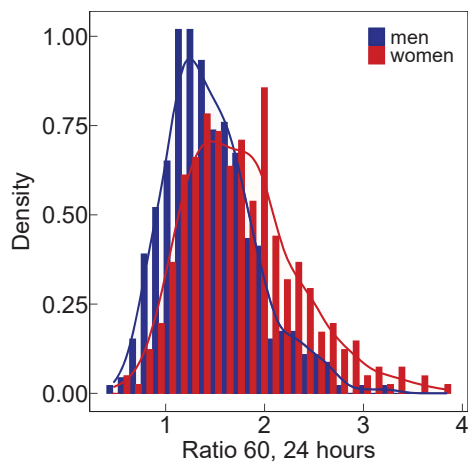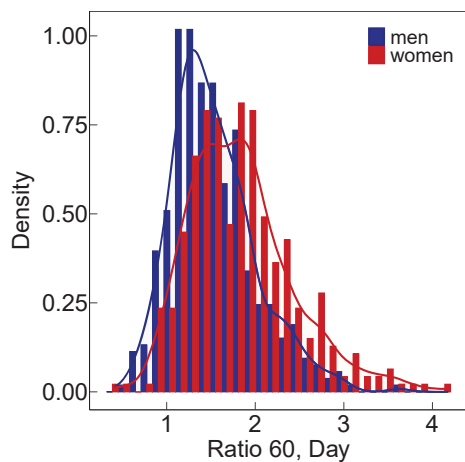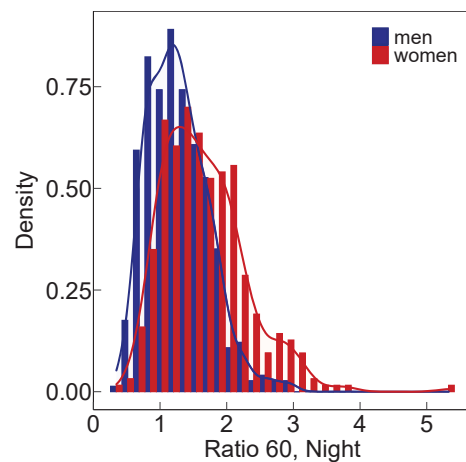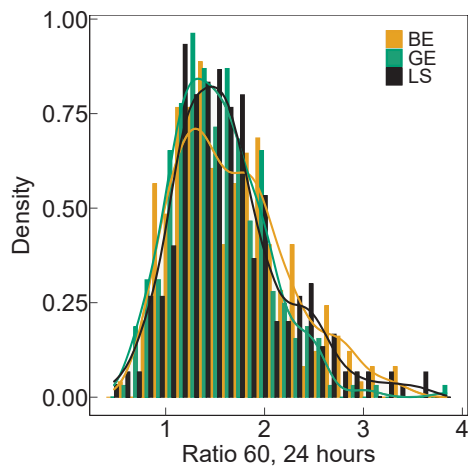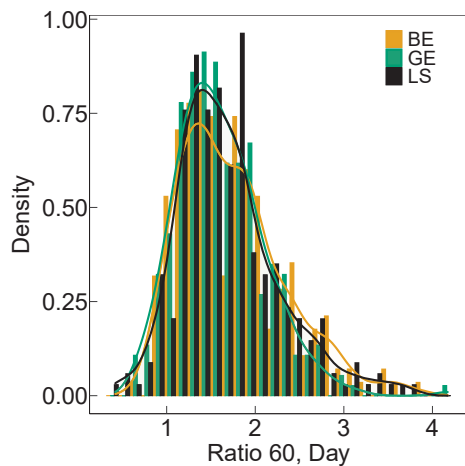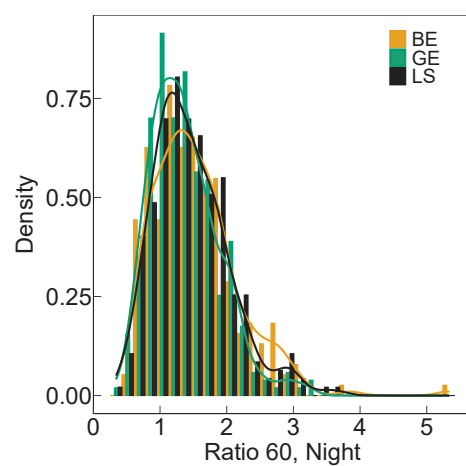

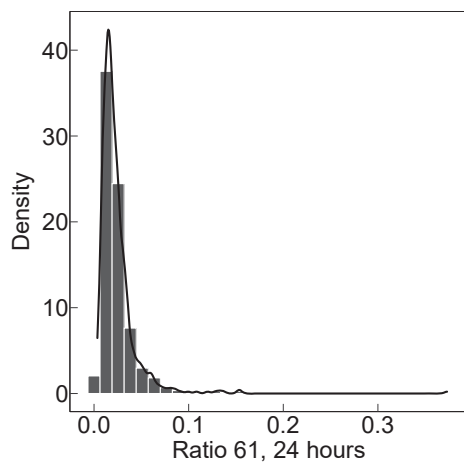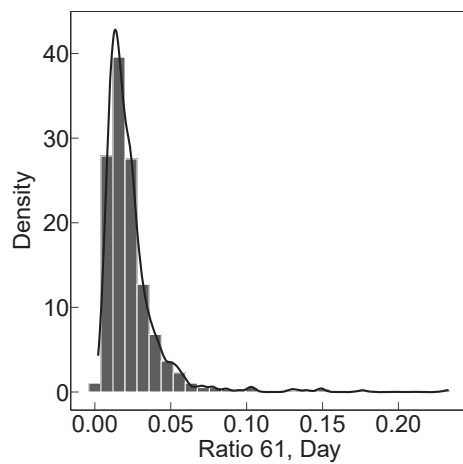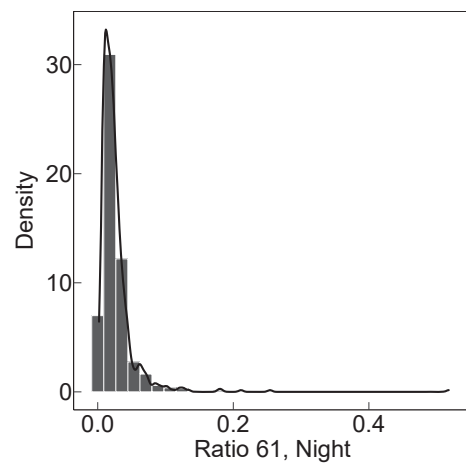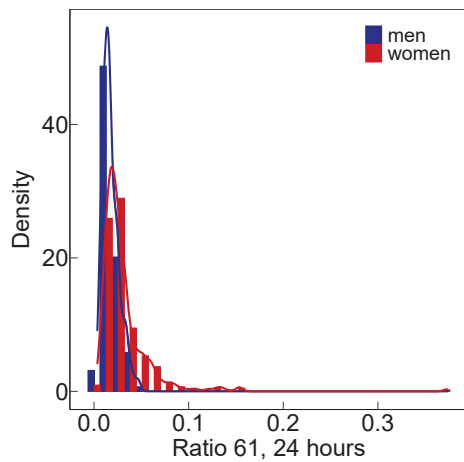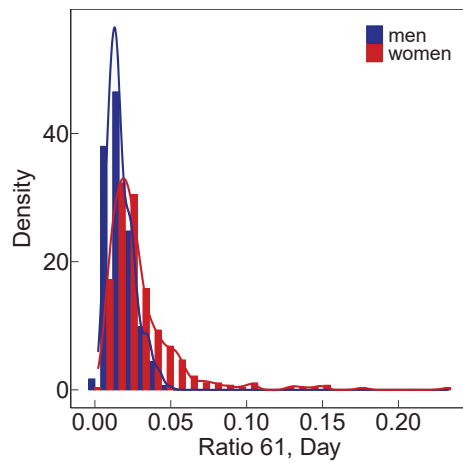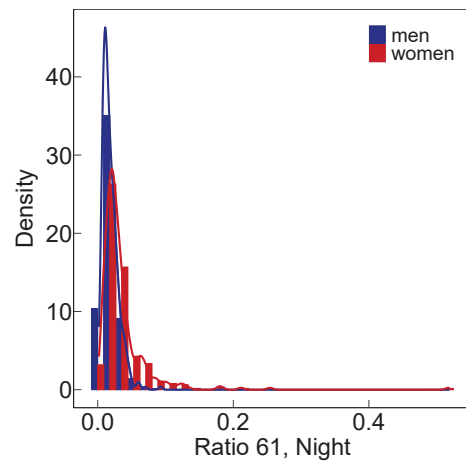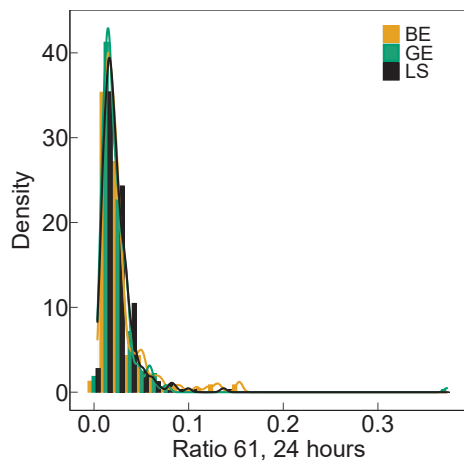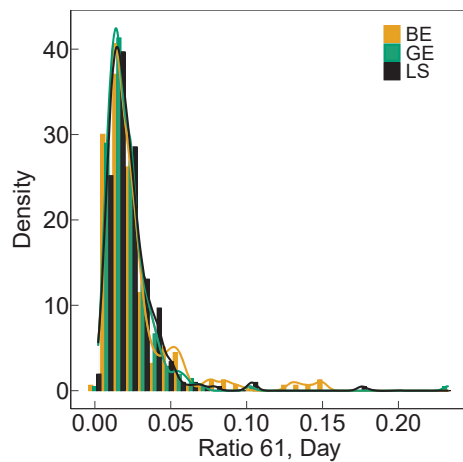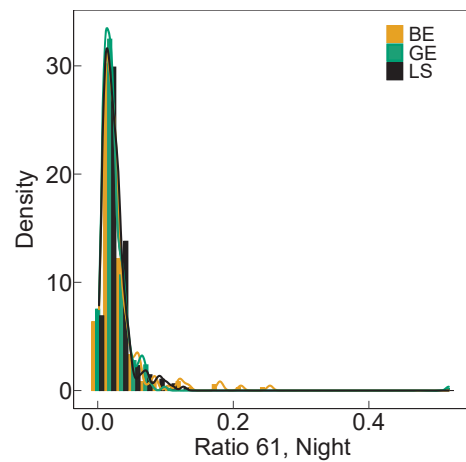

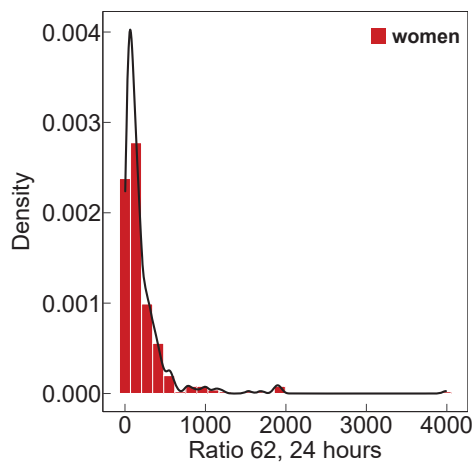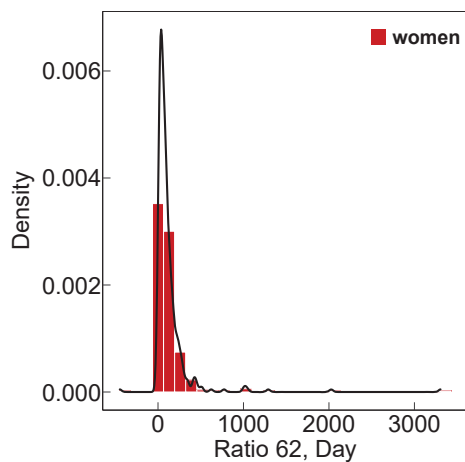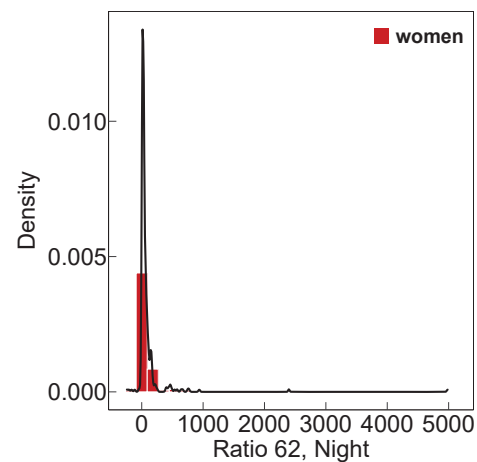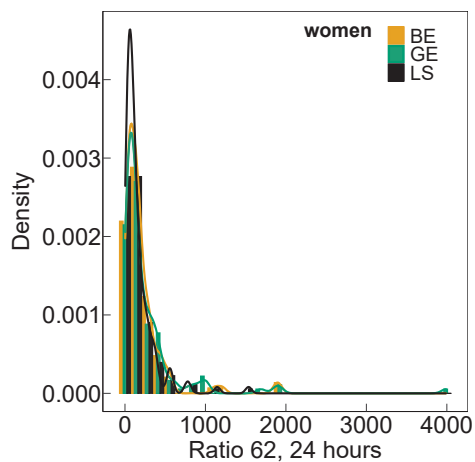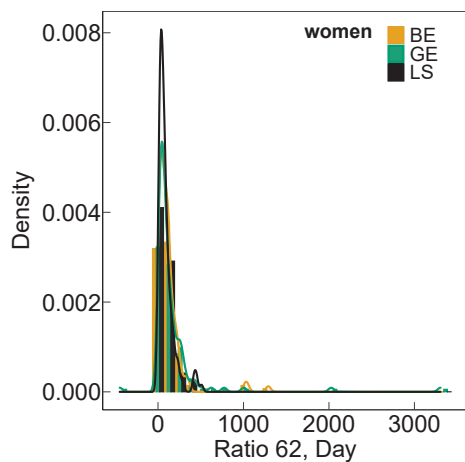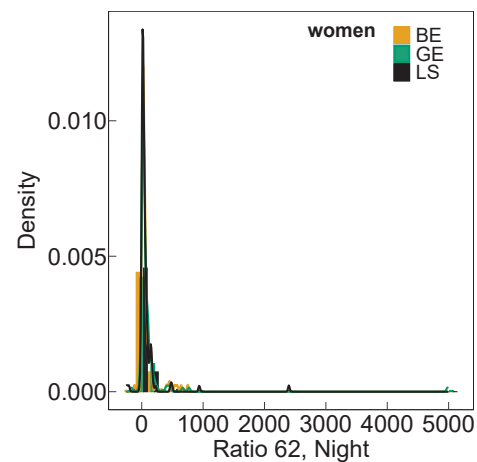

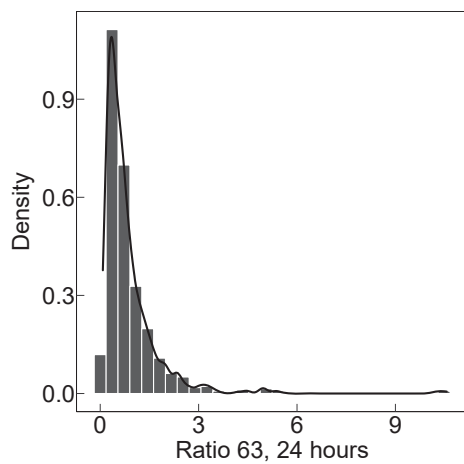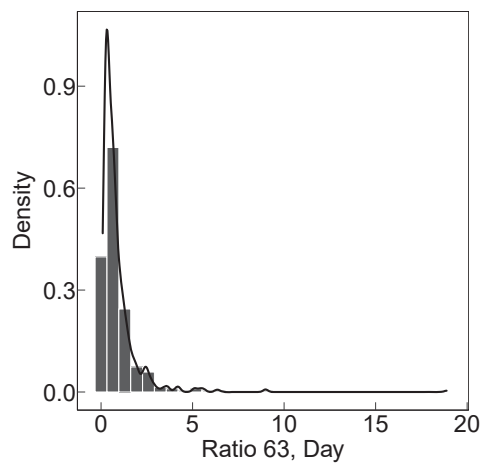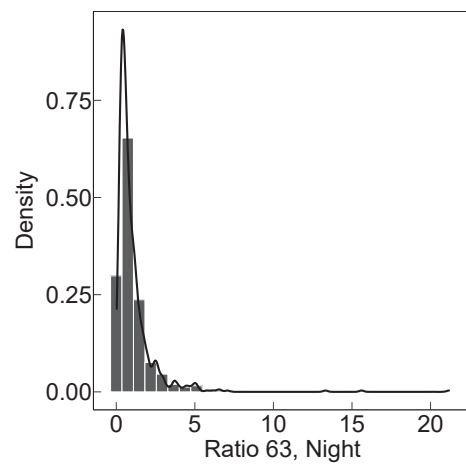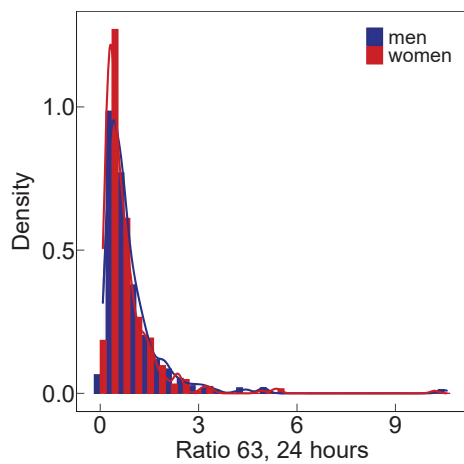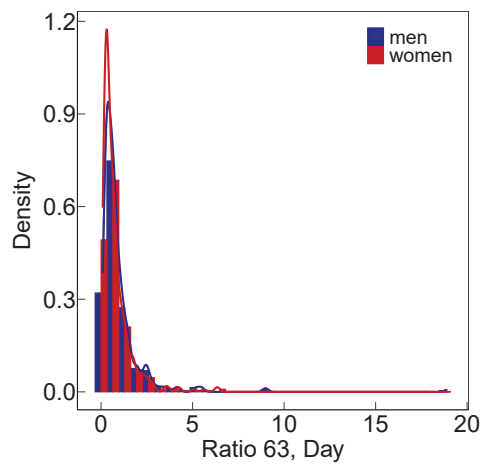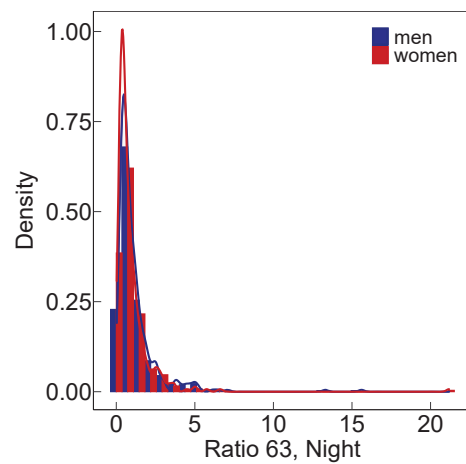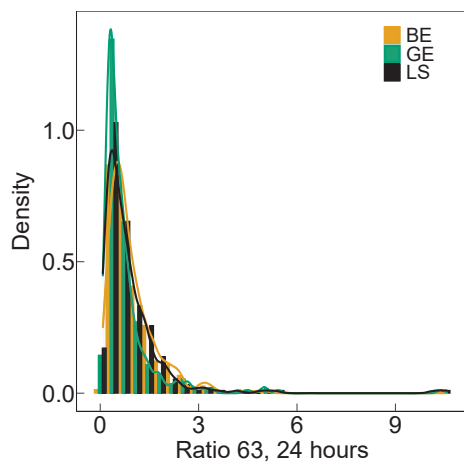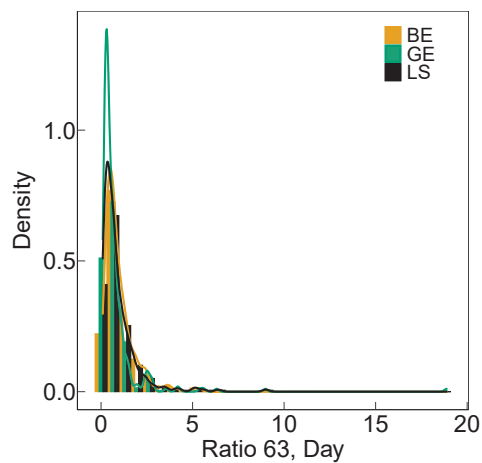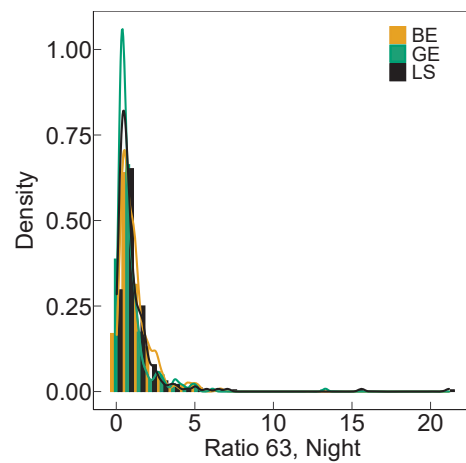

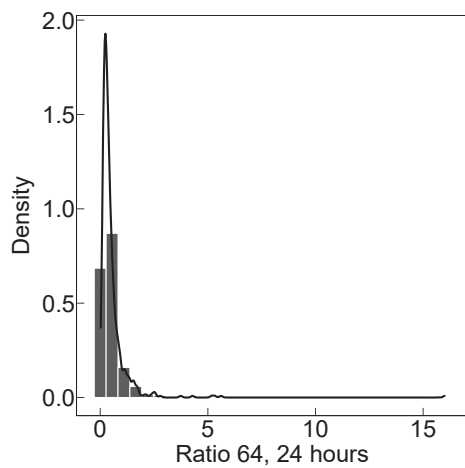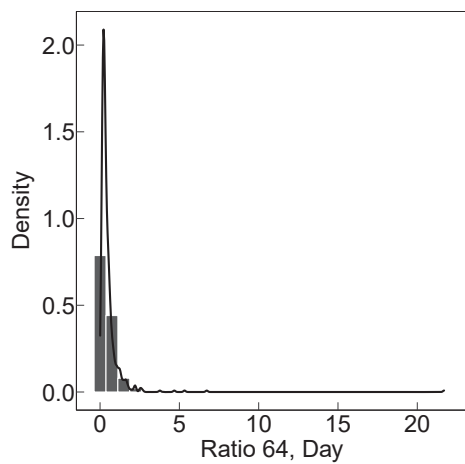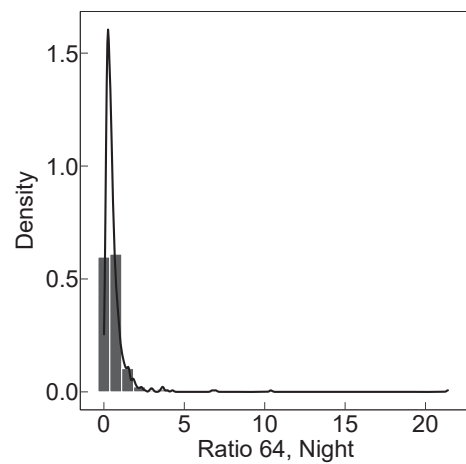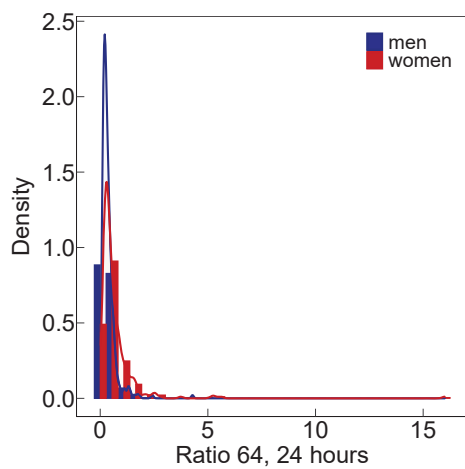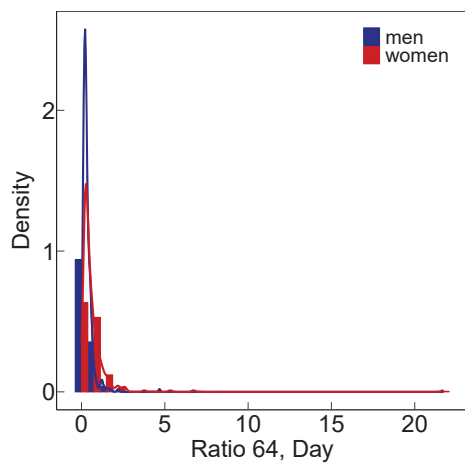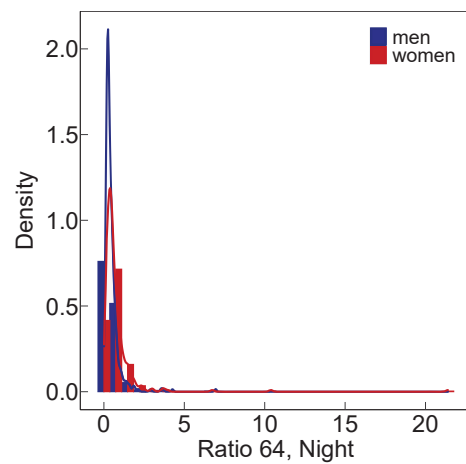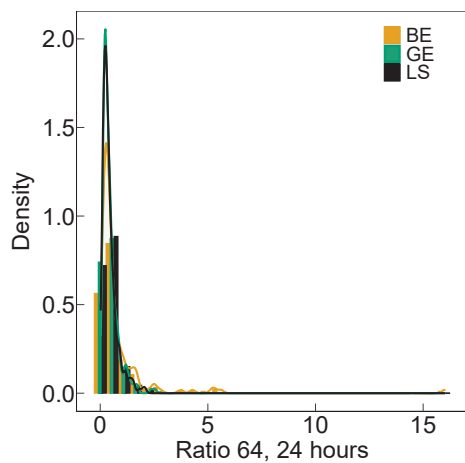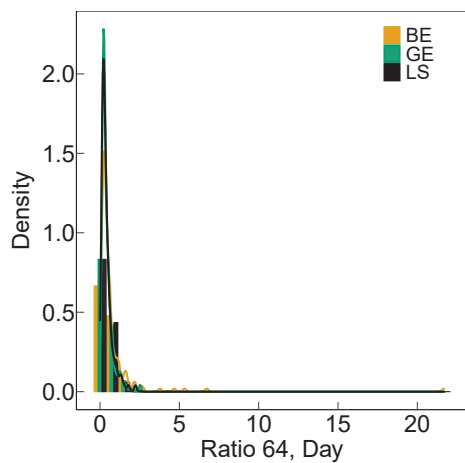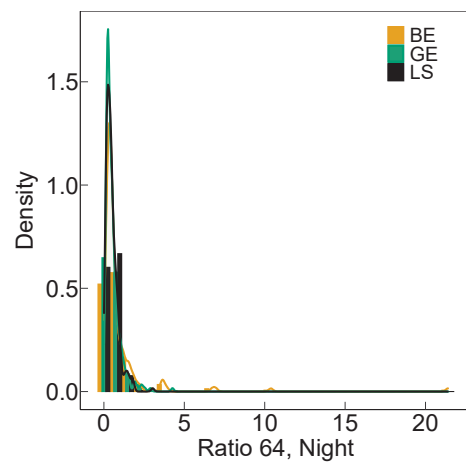

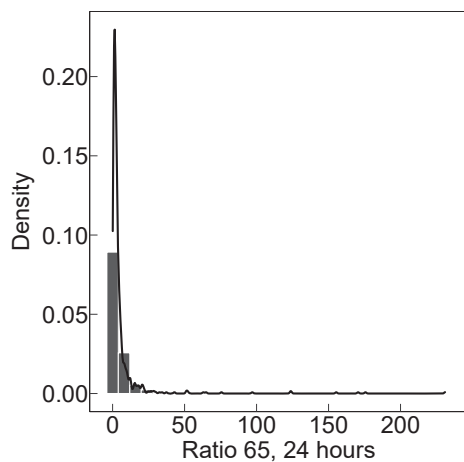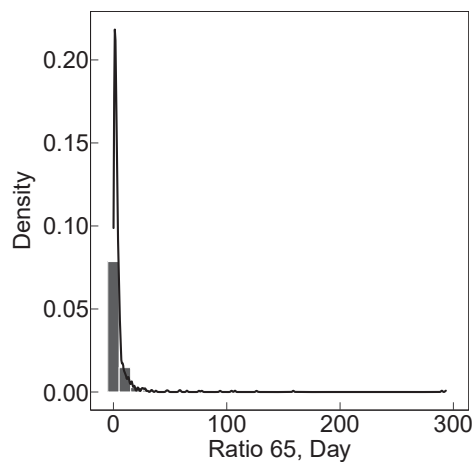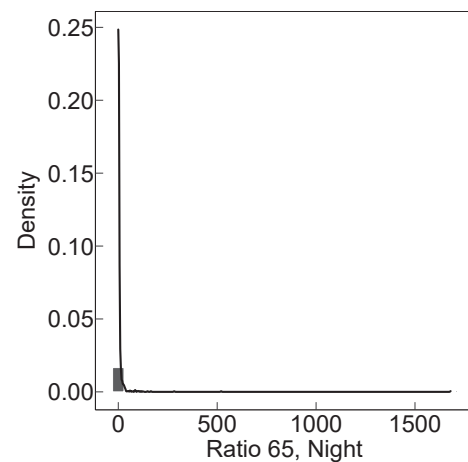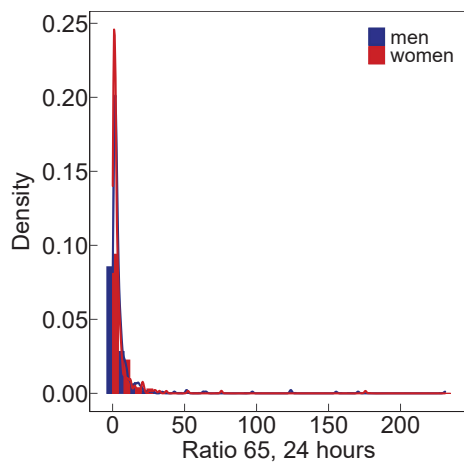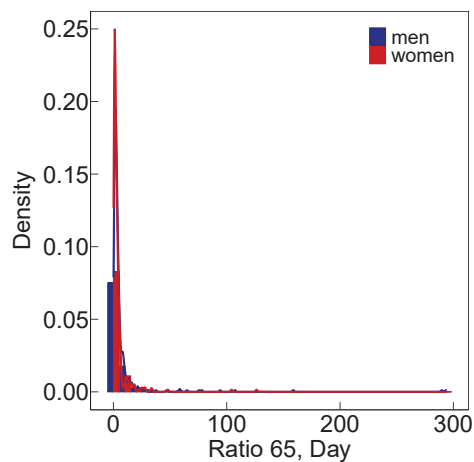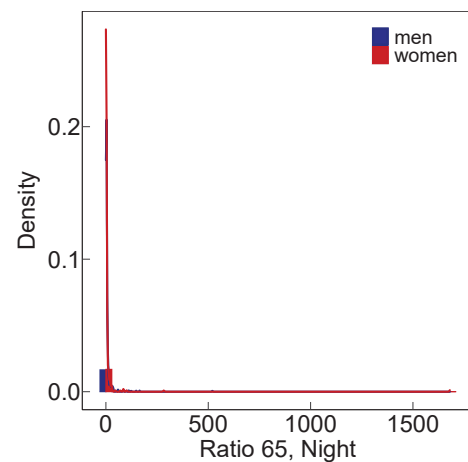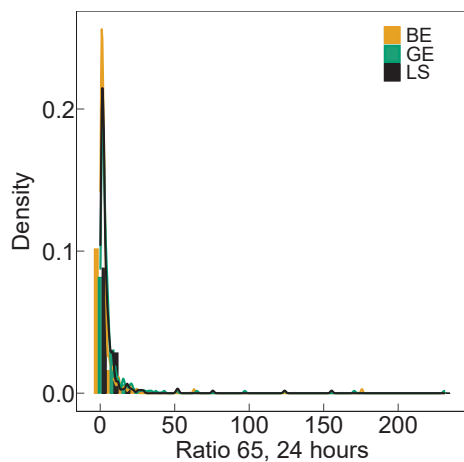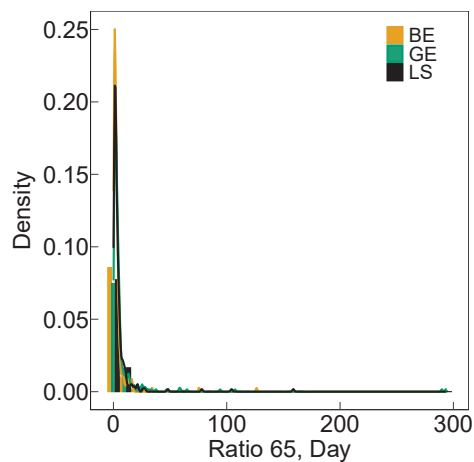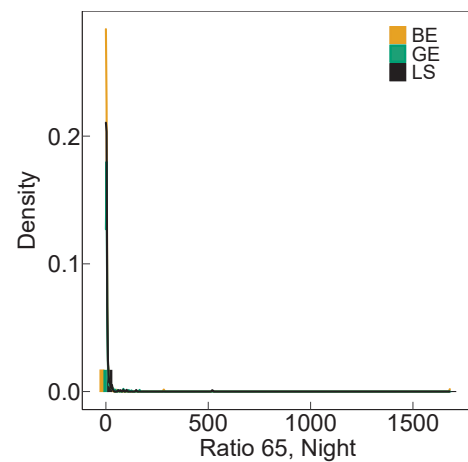

Supplement: S1 Fig — Kernel density plot histograms of diagnostic ratios are shown from left to right for 24-hour urine collections (left panels), daytime urine collections (center panels), and night-time urine collections (right panels) and from top to the bottom for the entire cohort (upper panels), separated for women and men (center panels), and separated for the study centers in Bern (BE), Geneva (GE), and Lausanne (LS). (PDF) [file pone.0253975.s001.pdf]

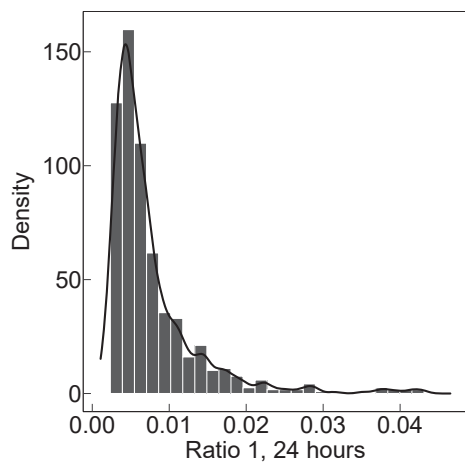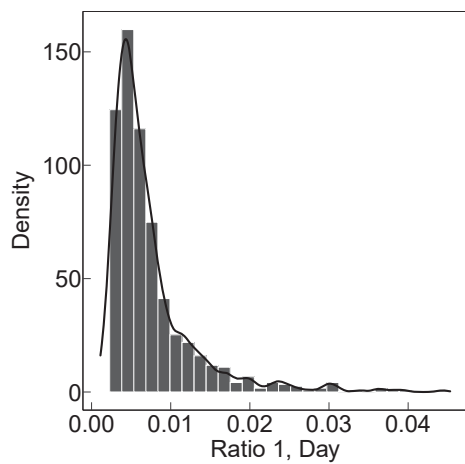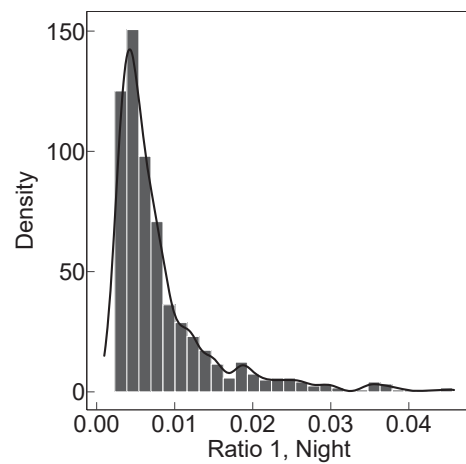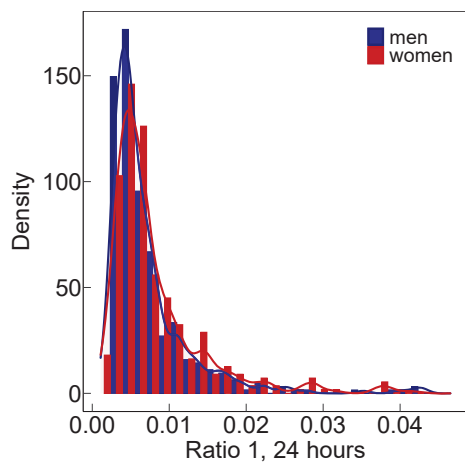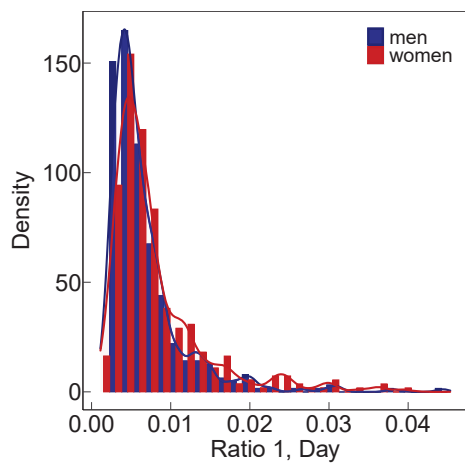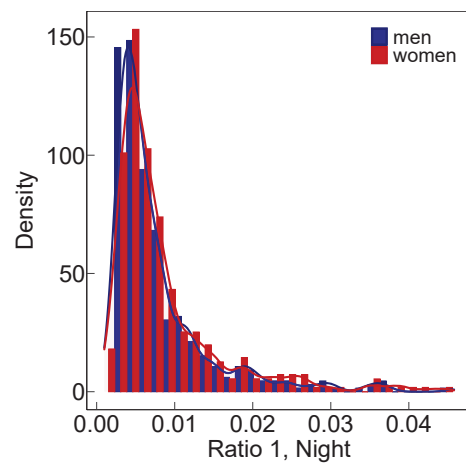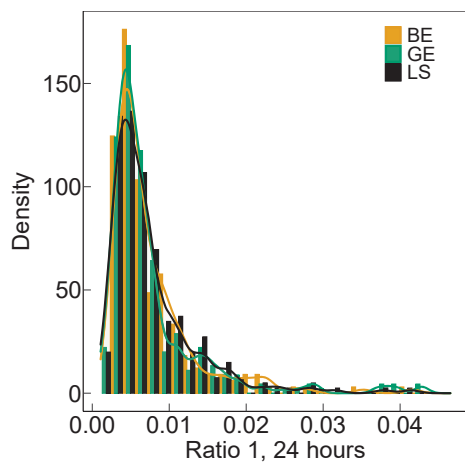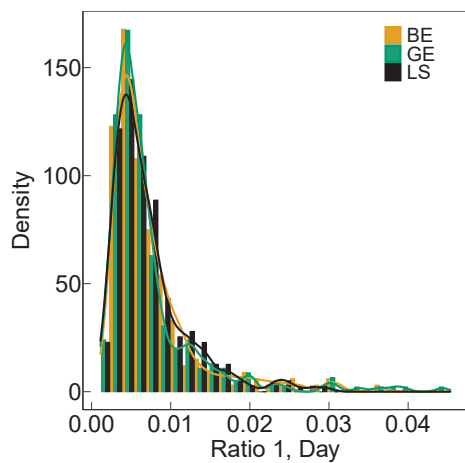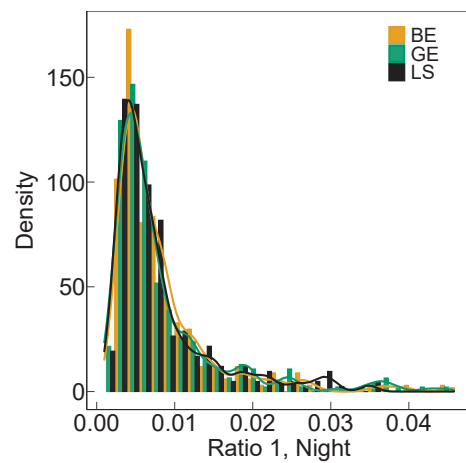

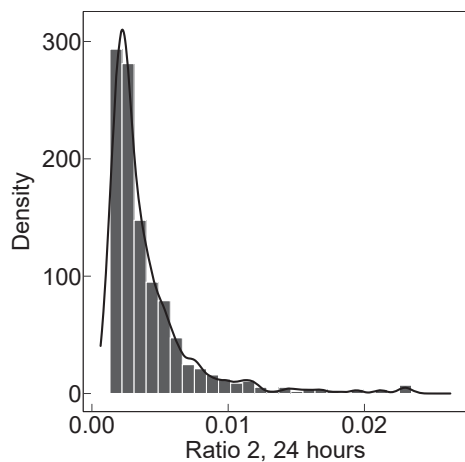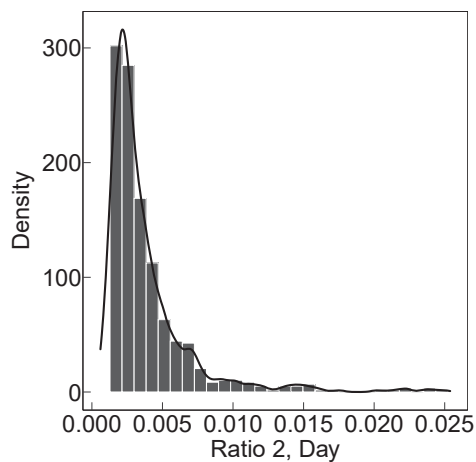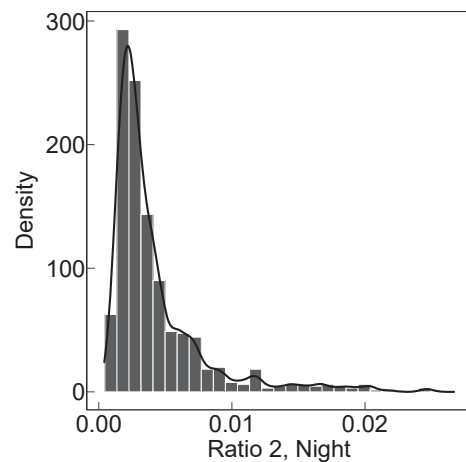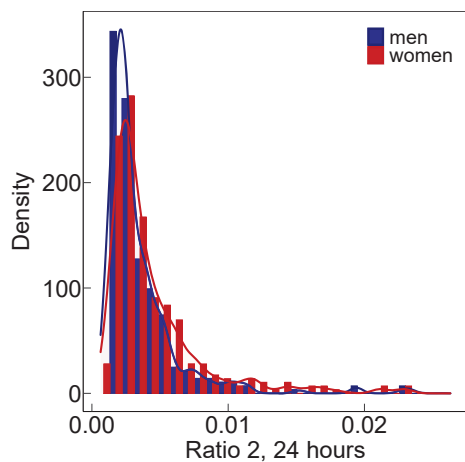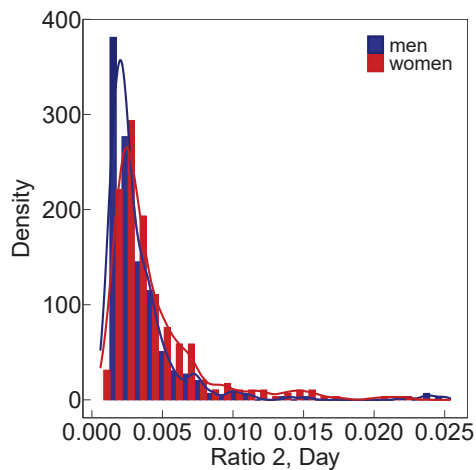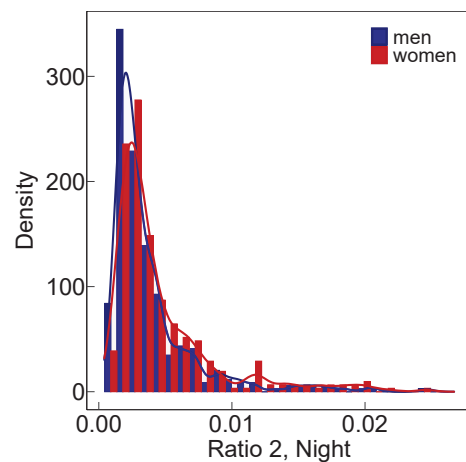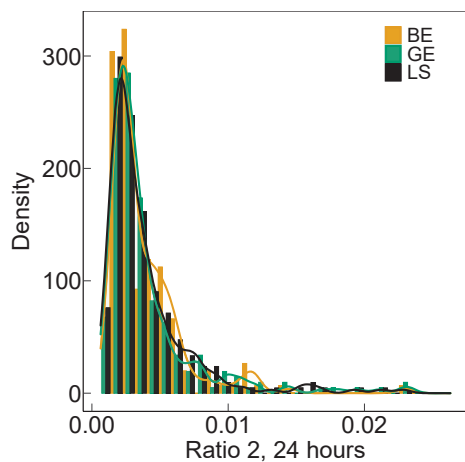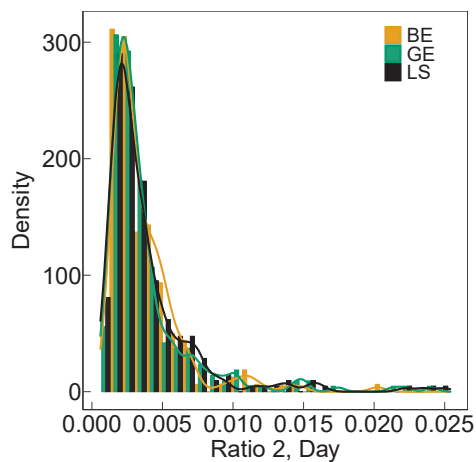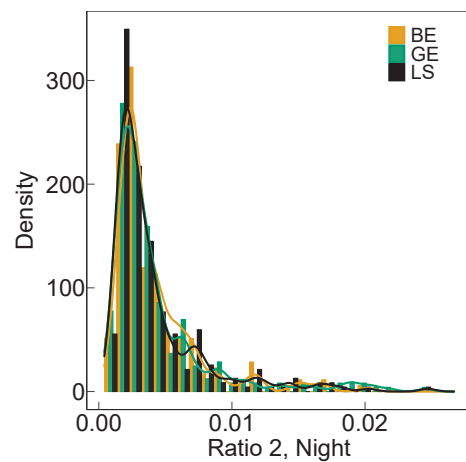

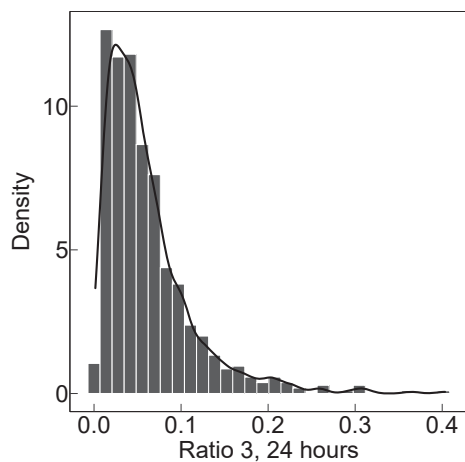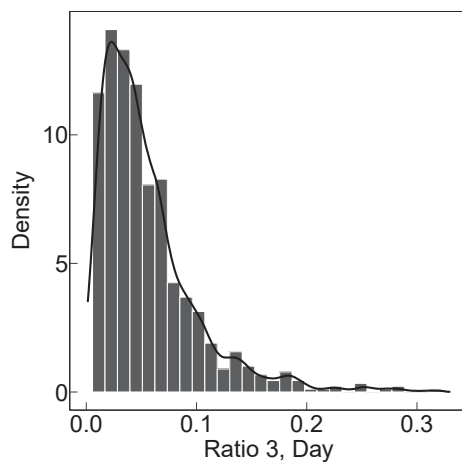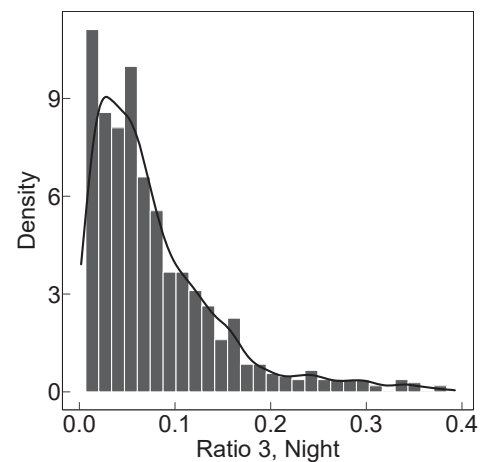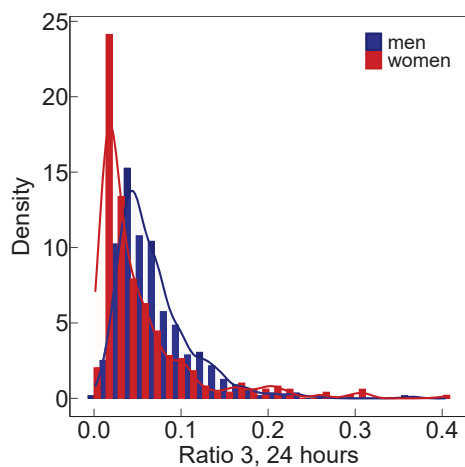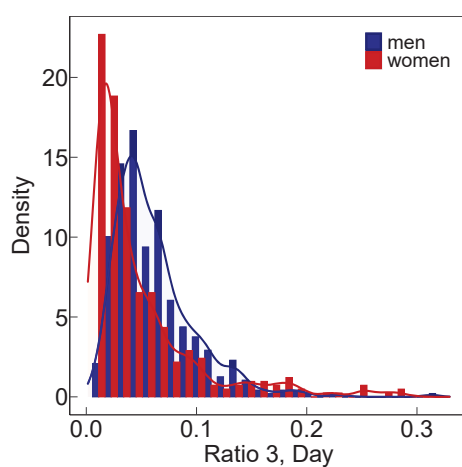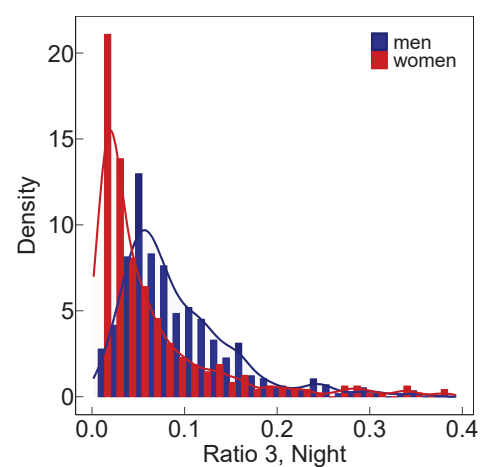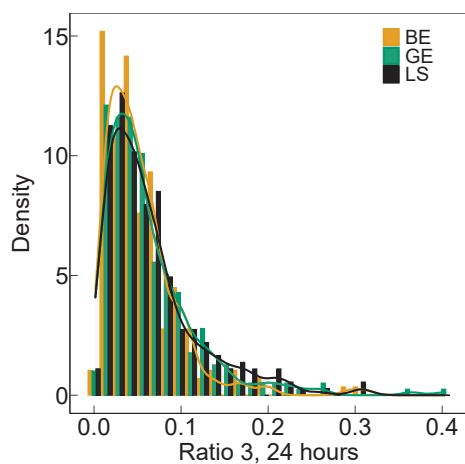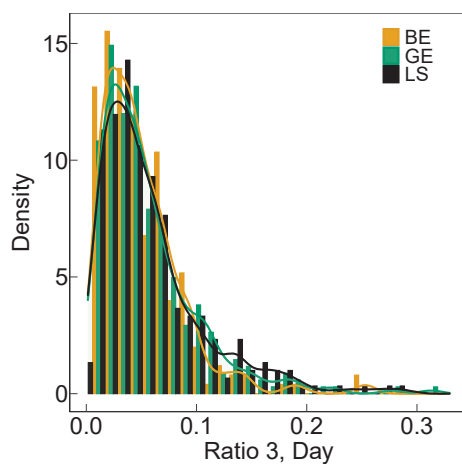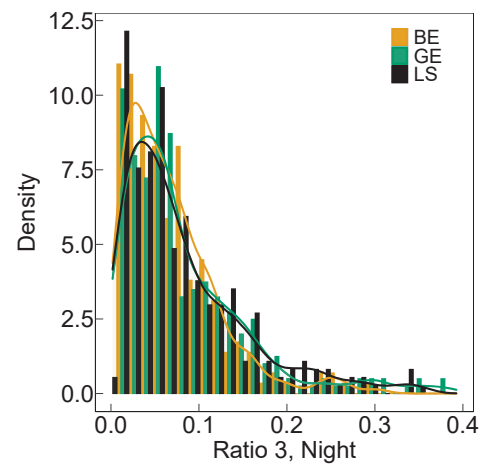

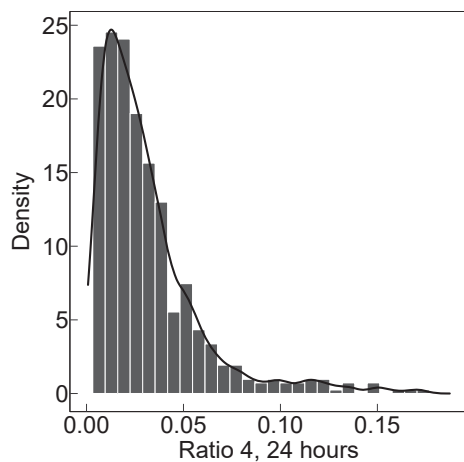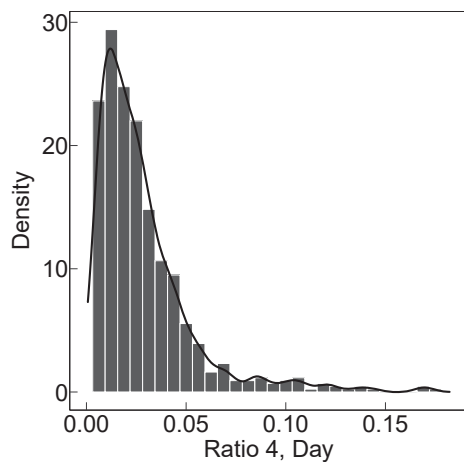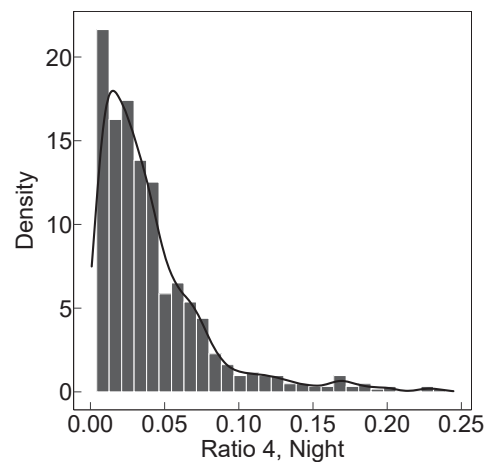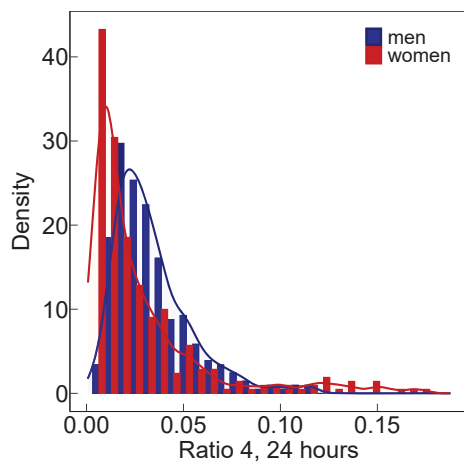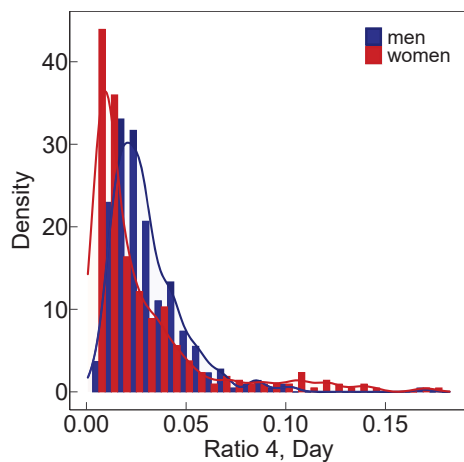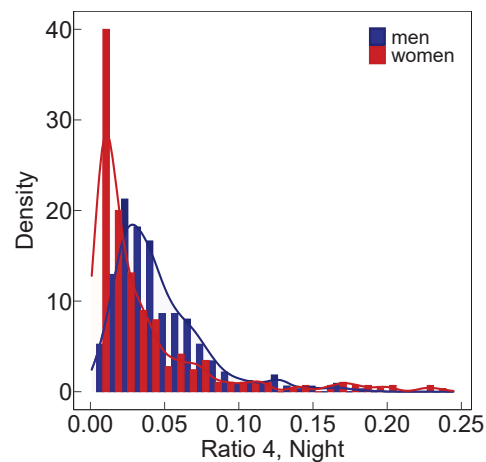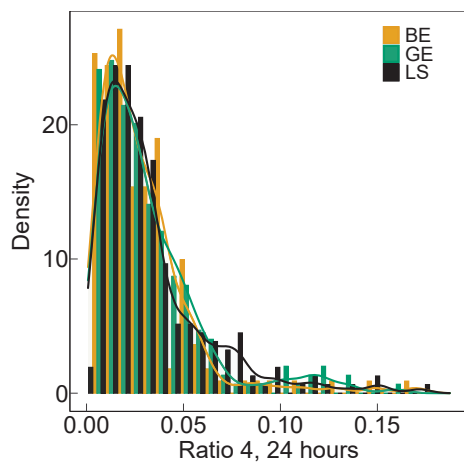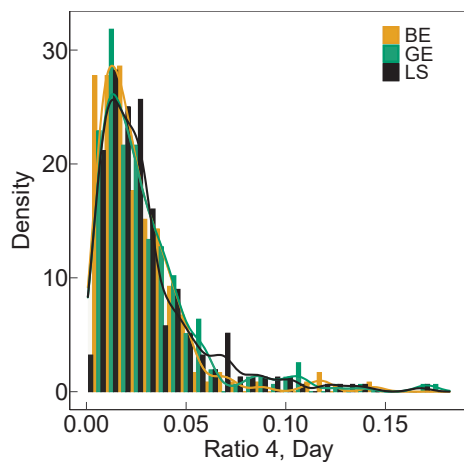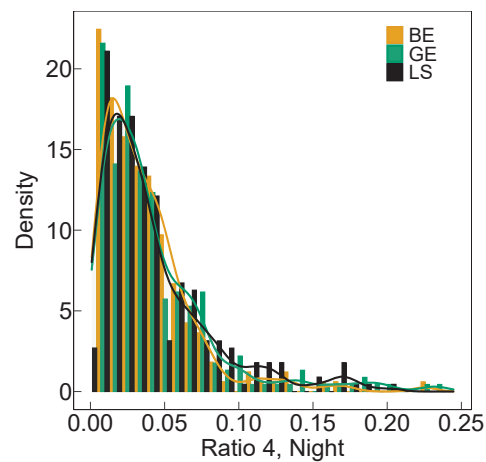

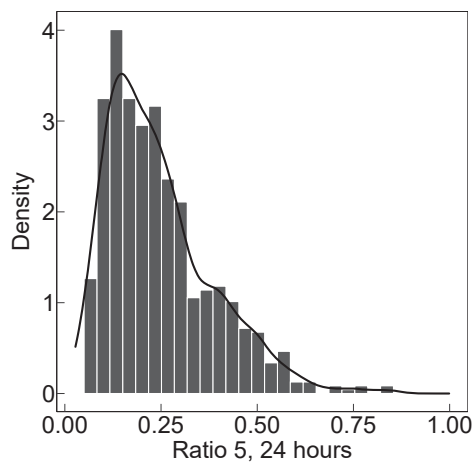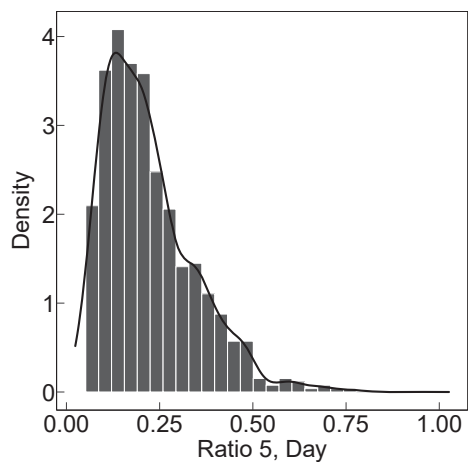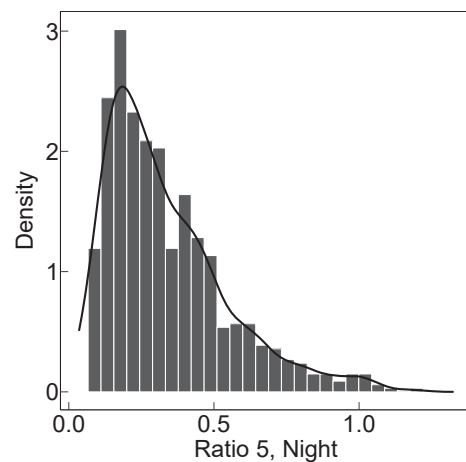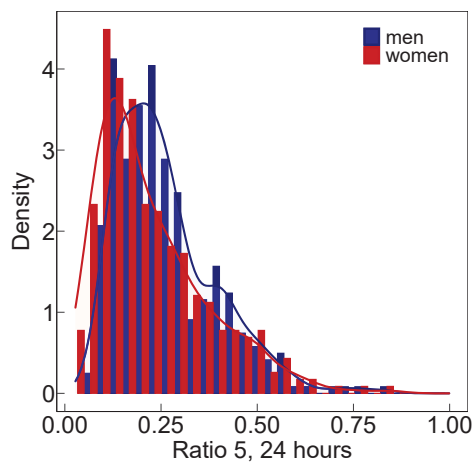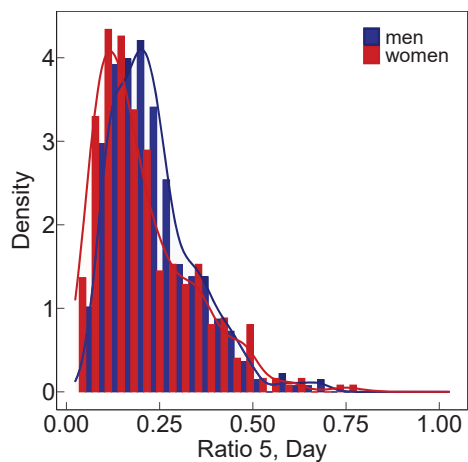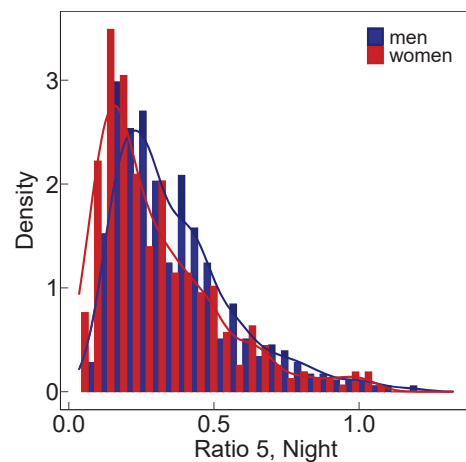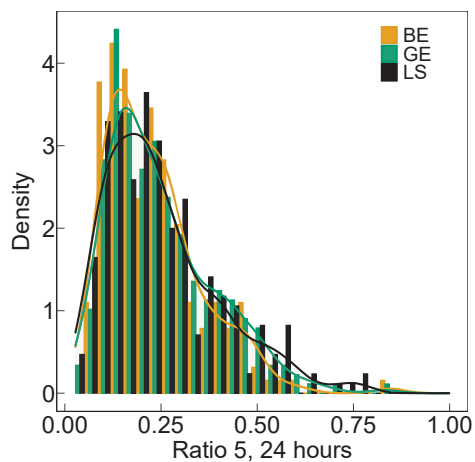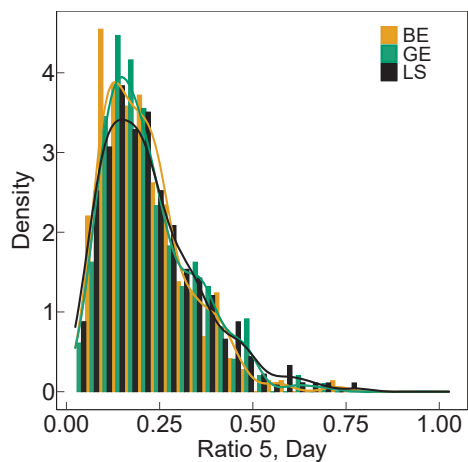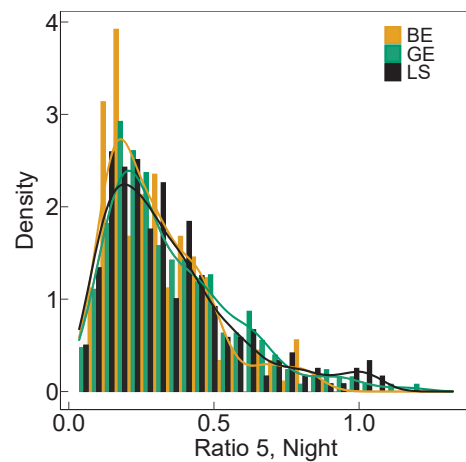

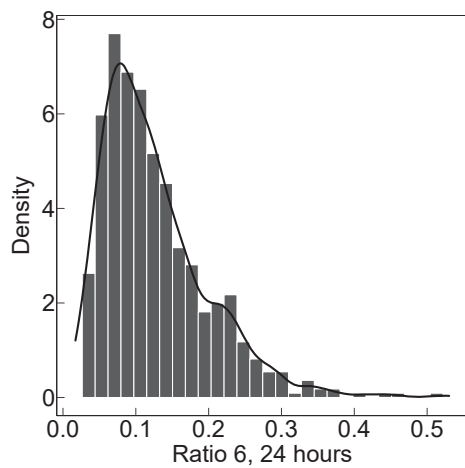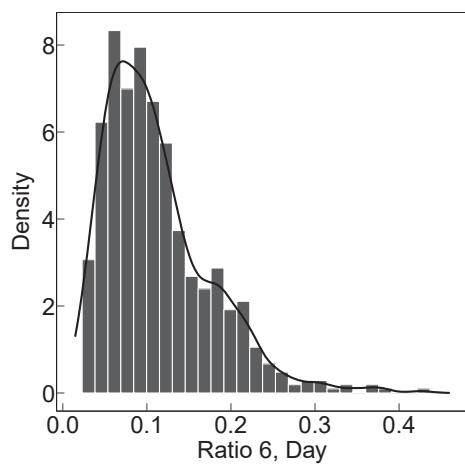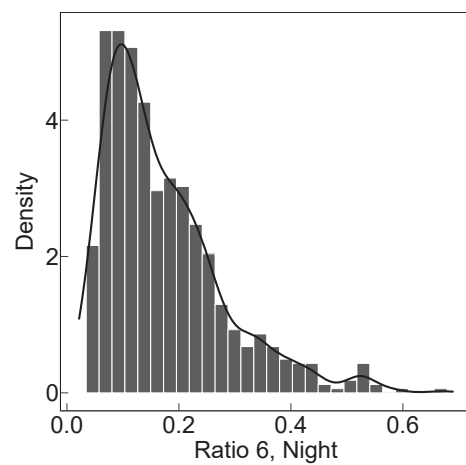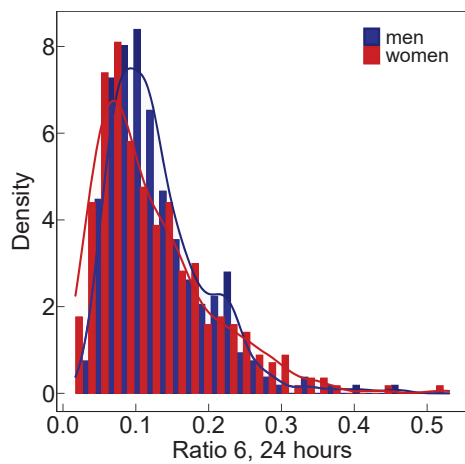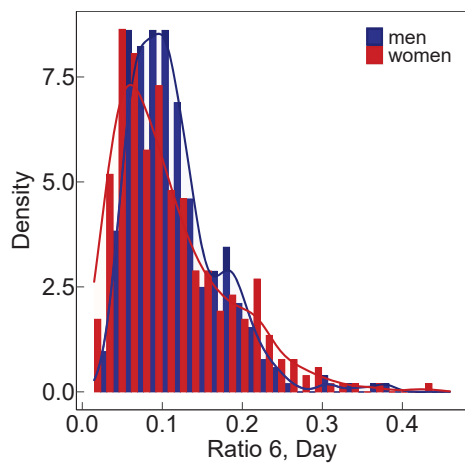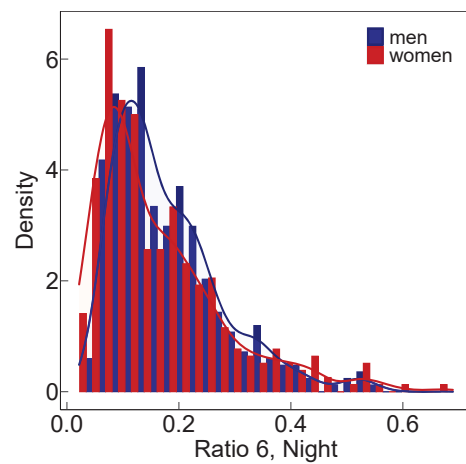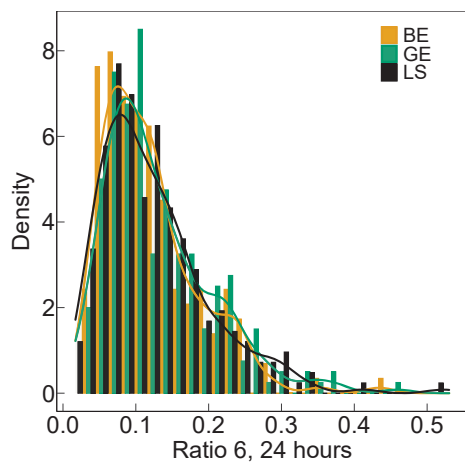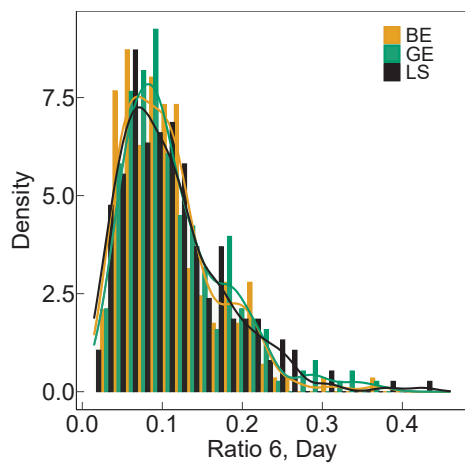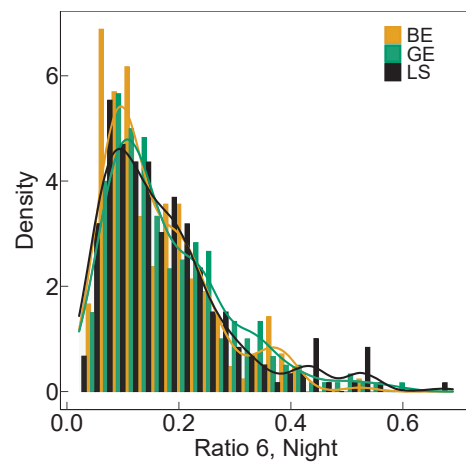

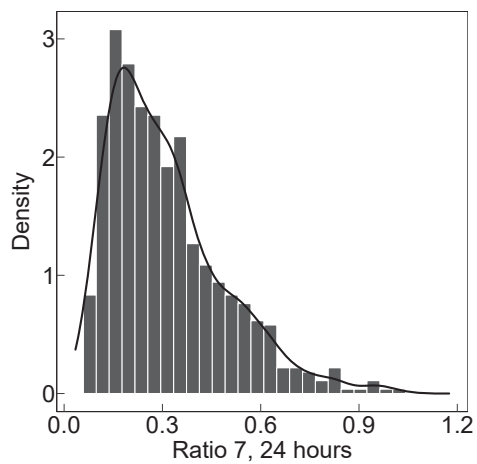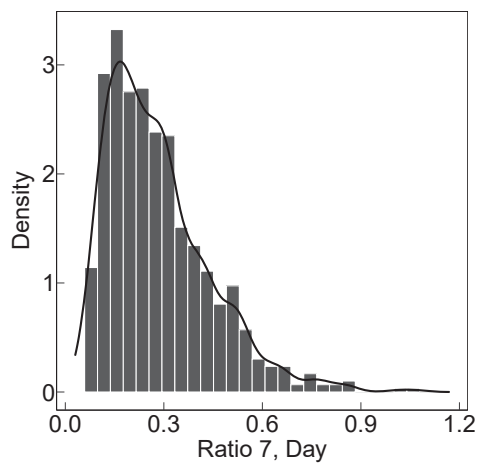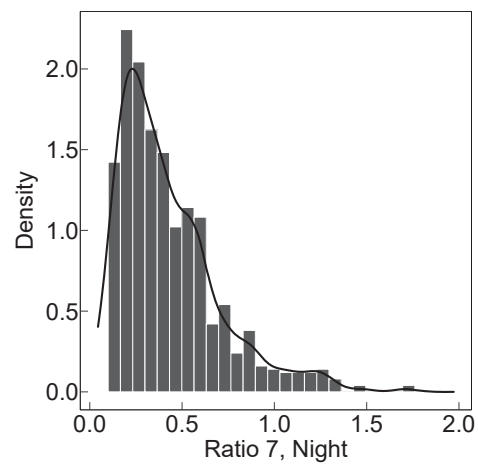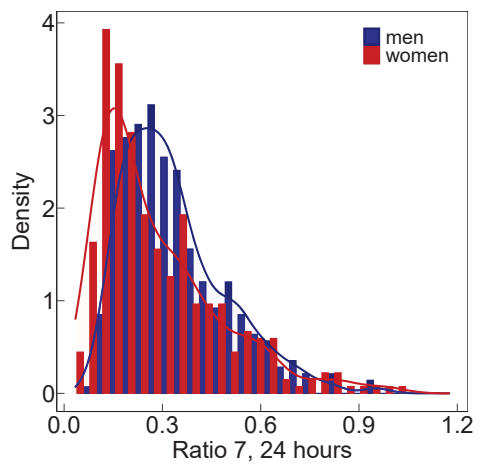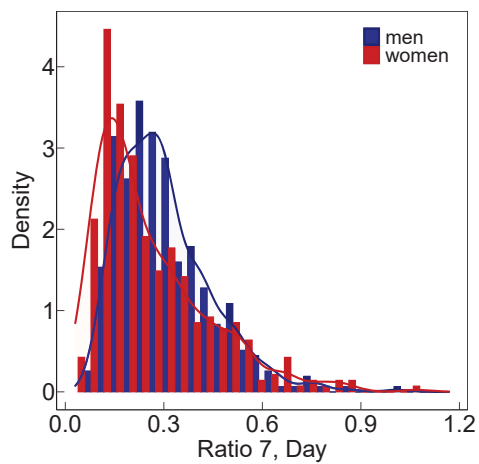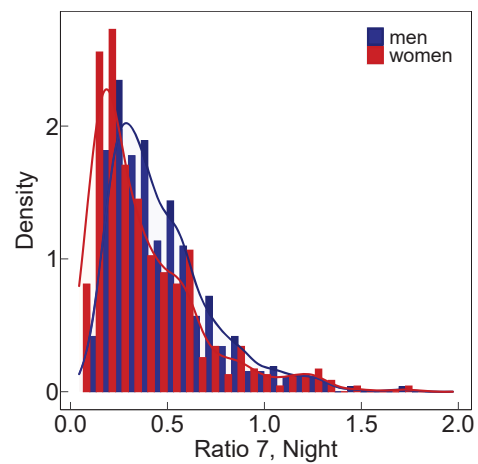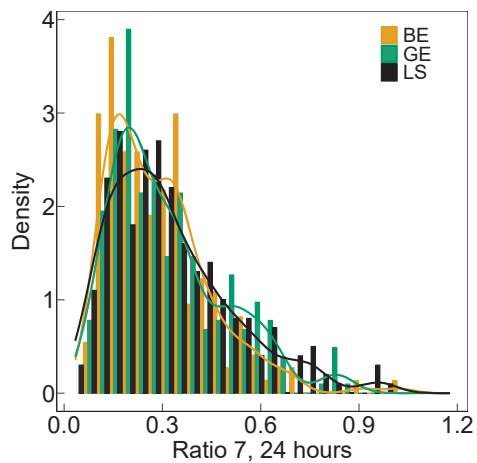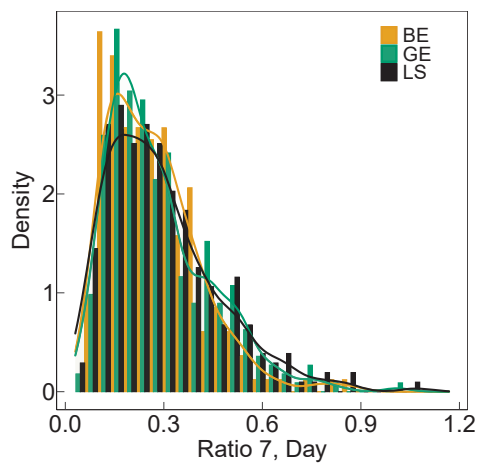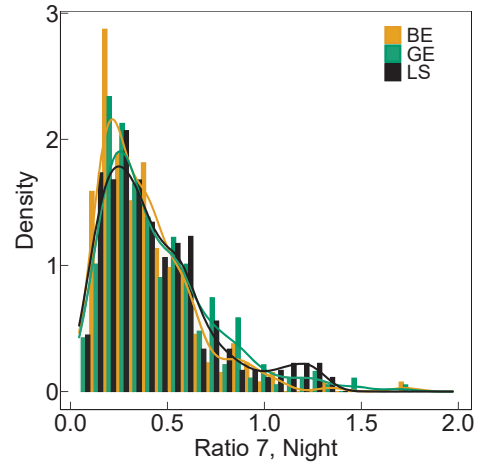

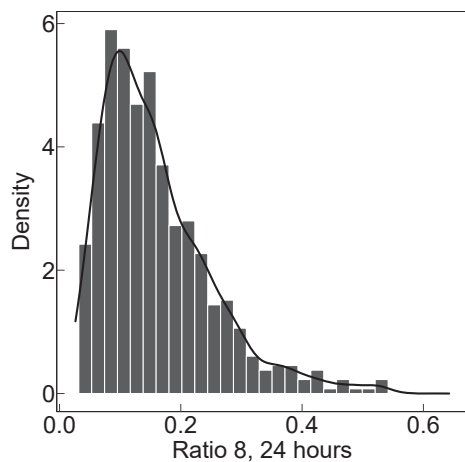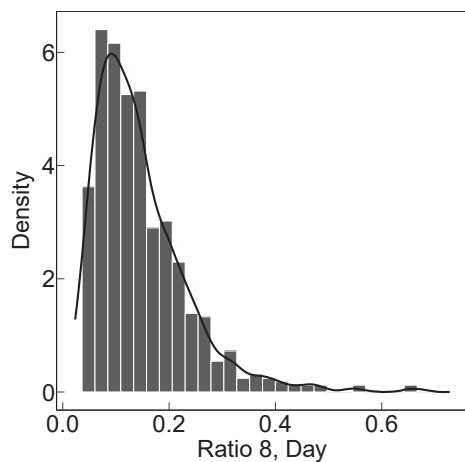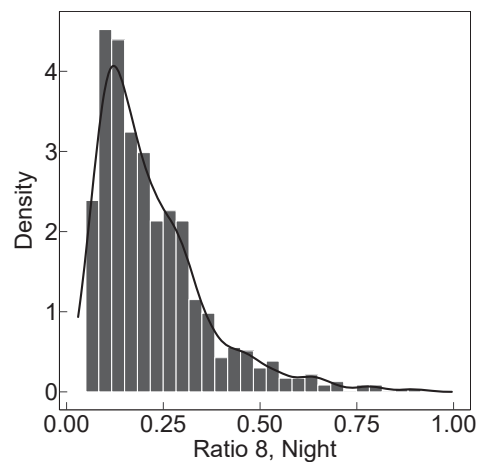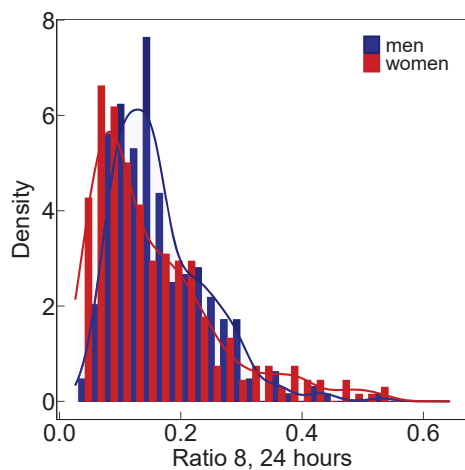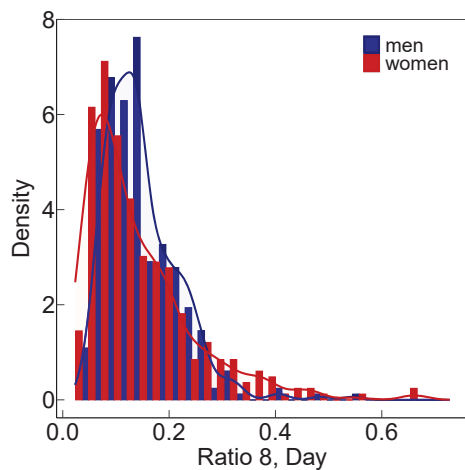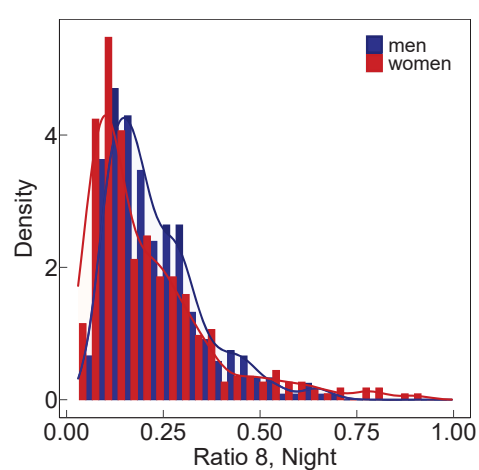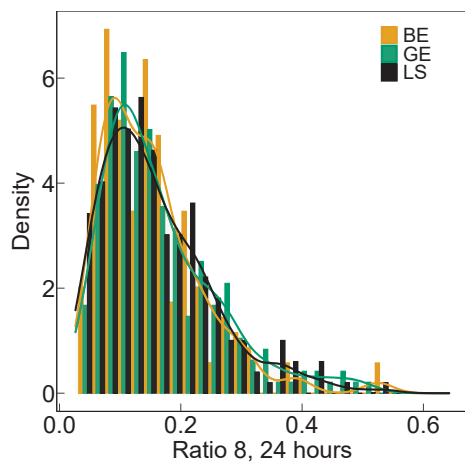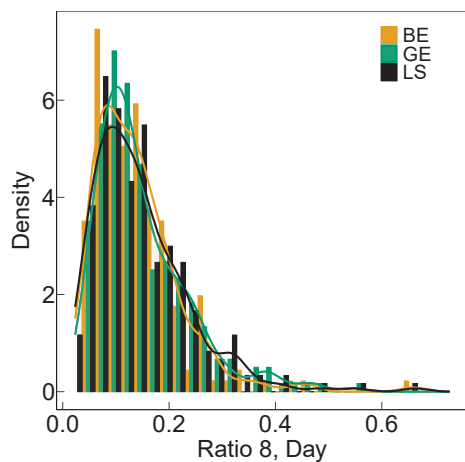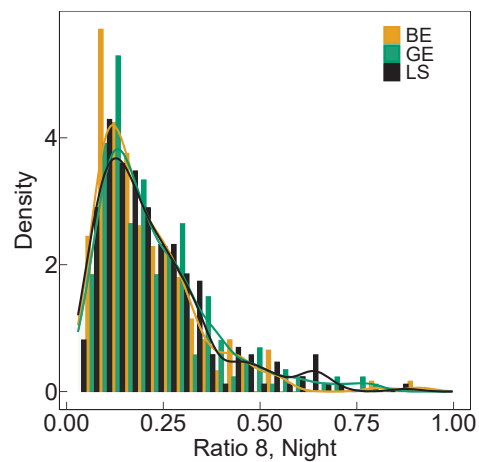

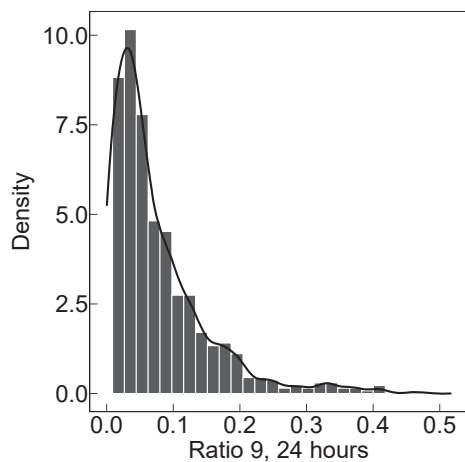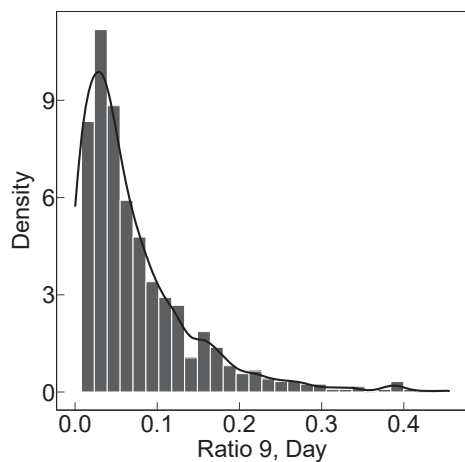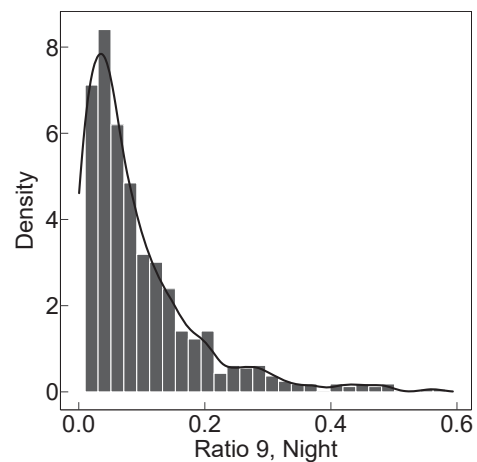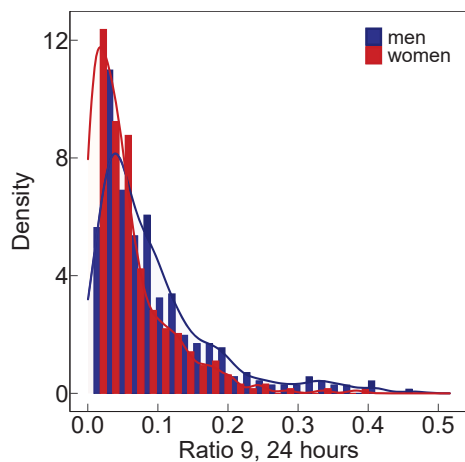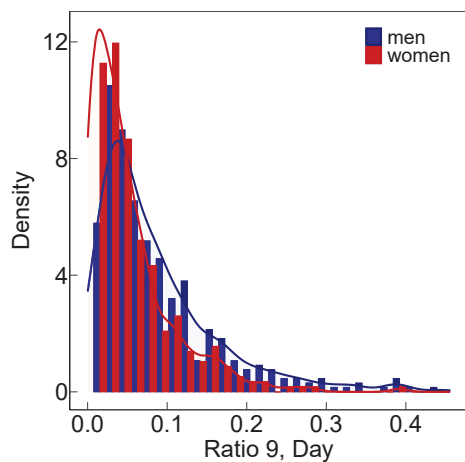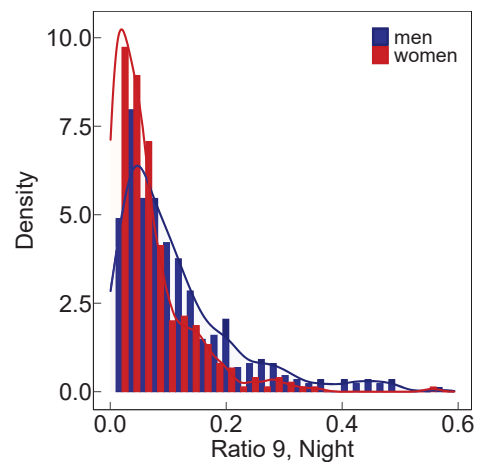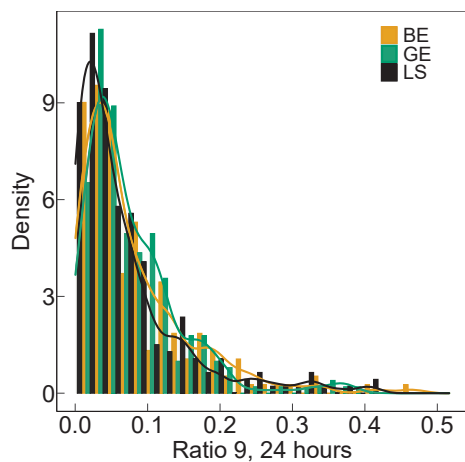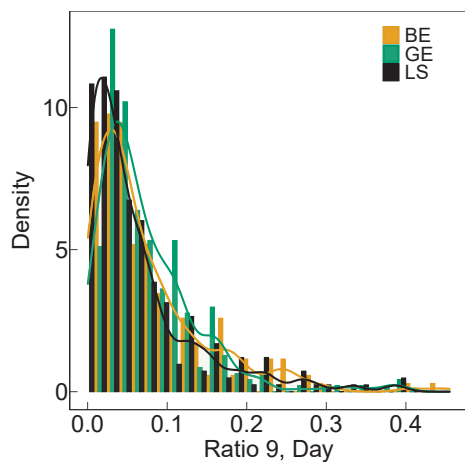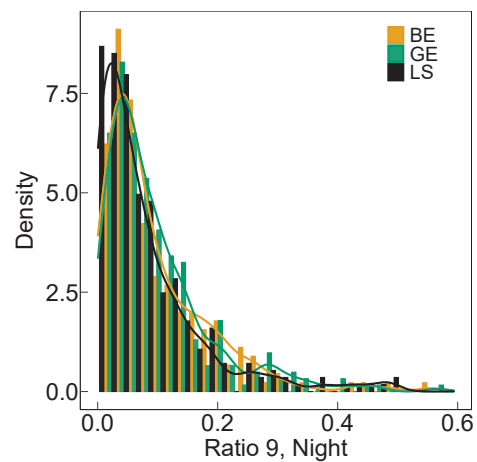

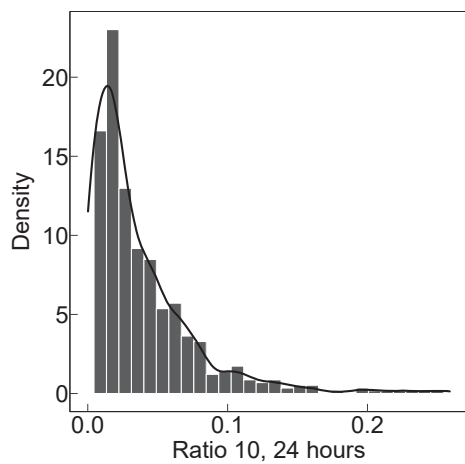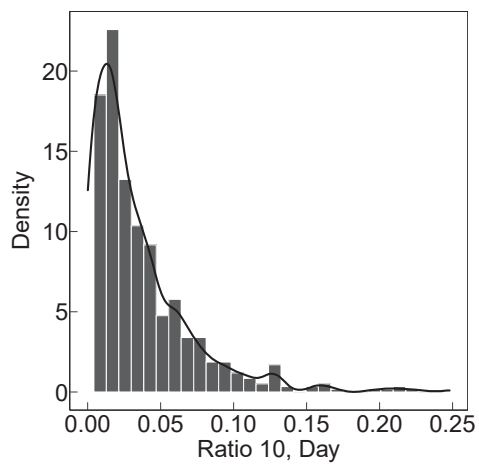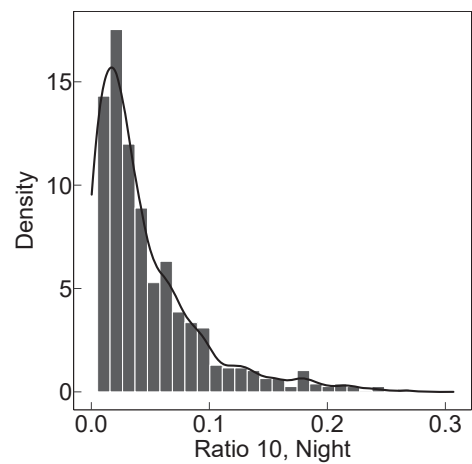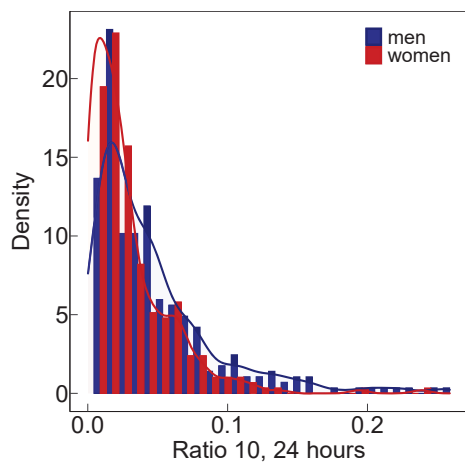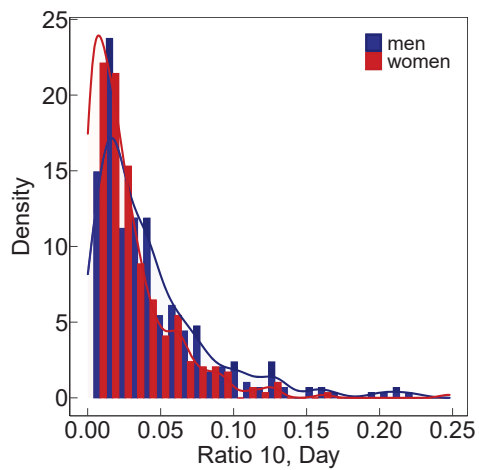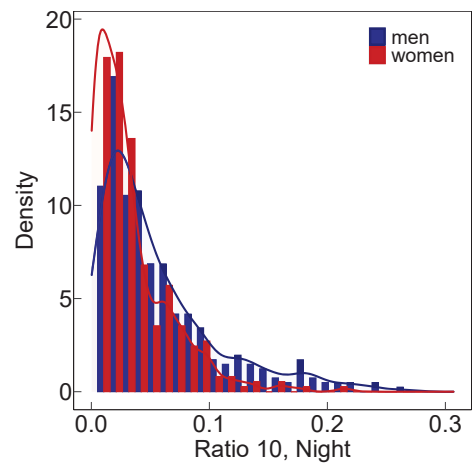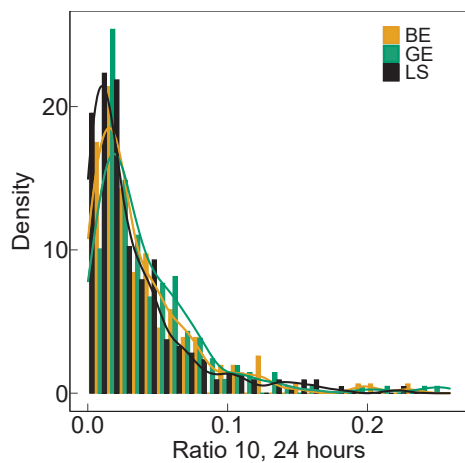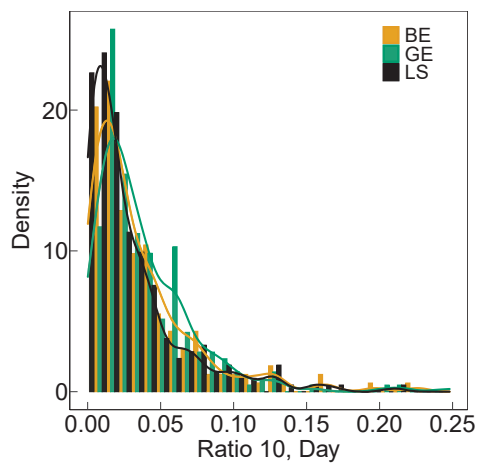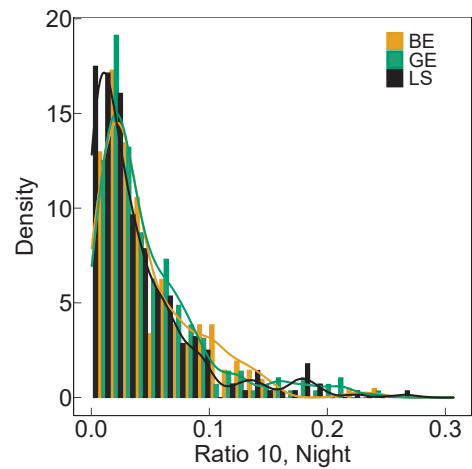

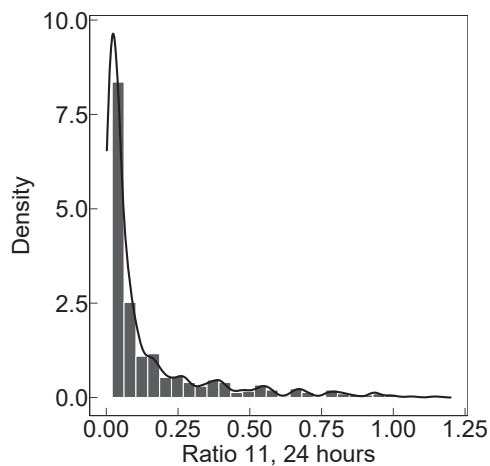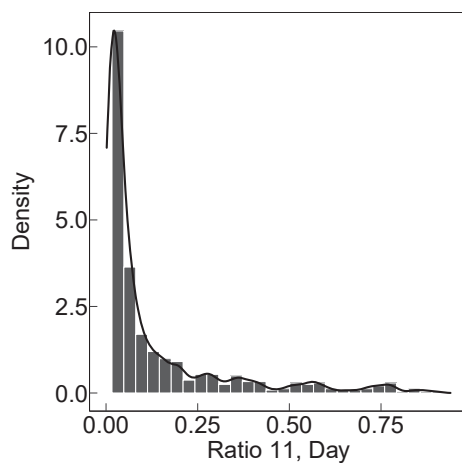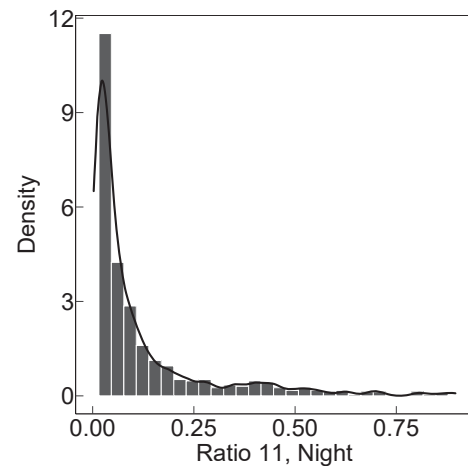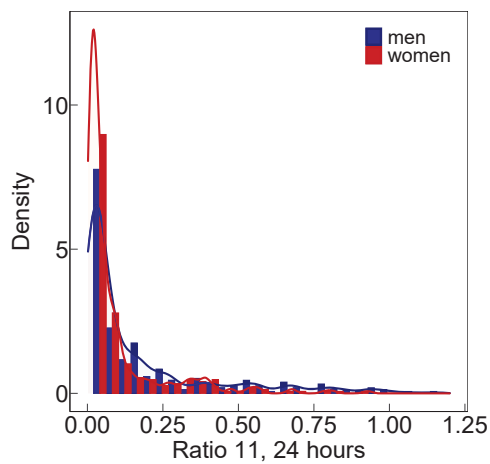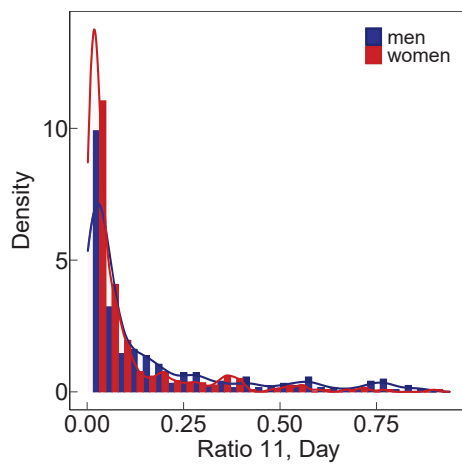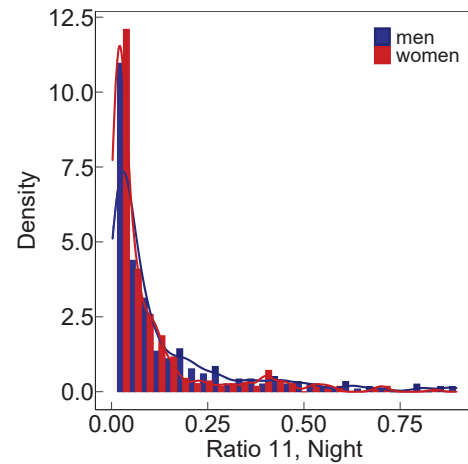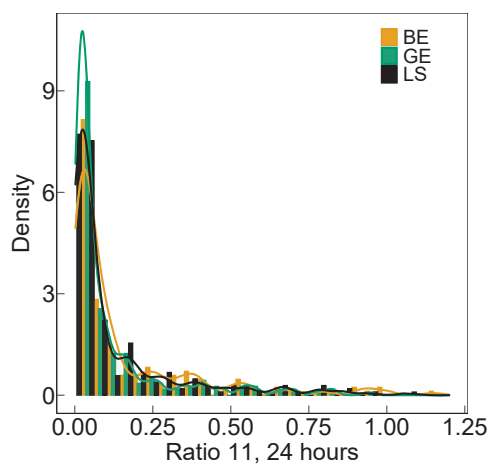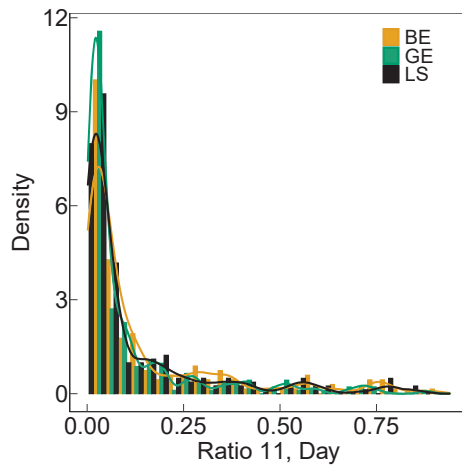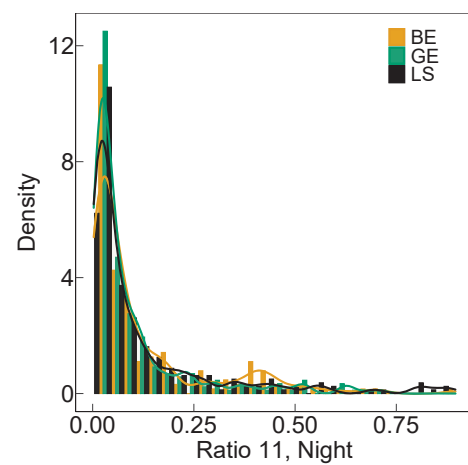

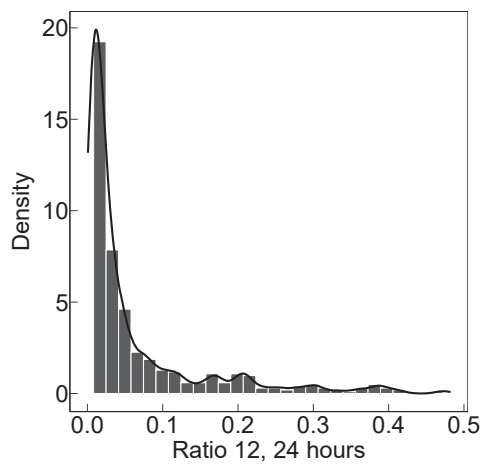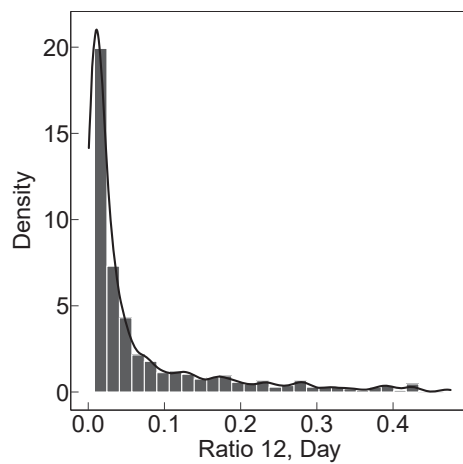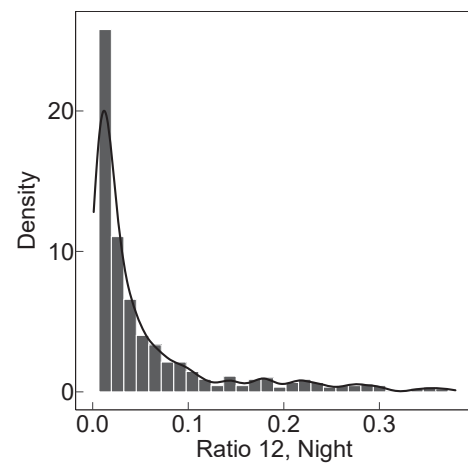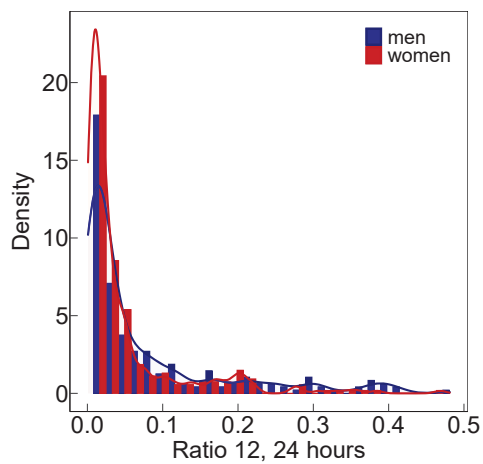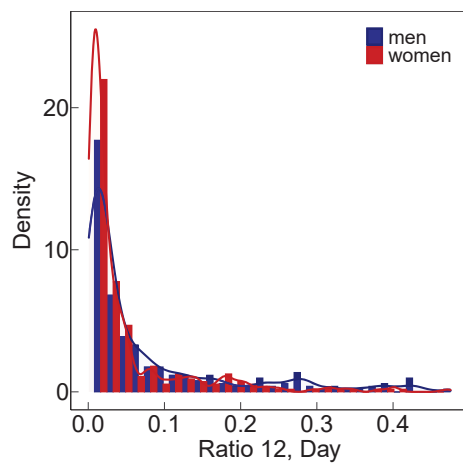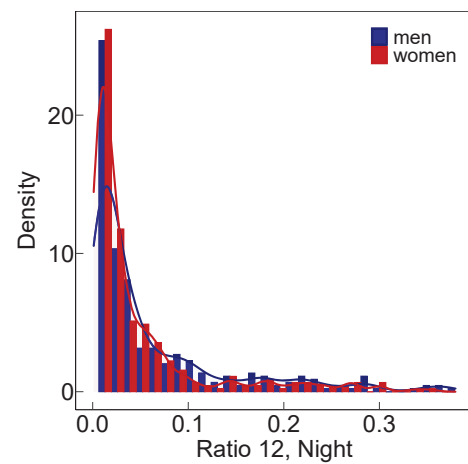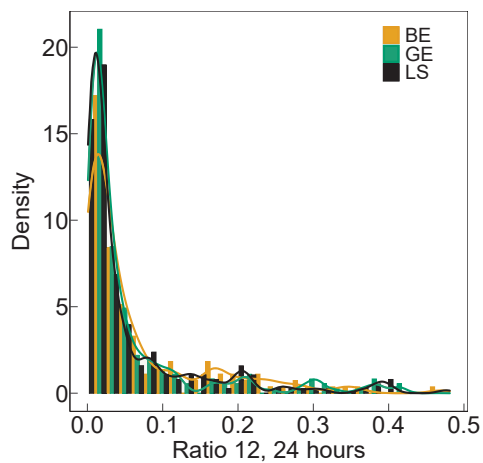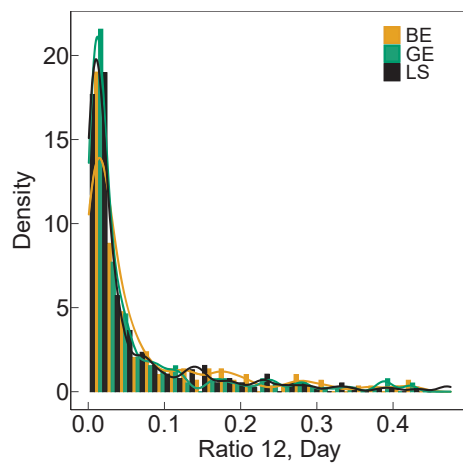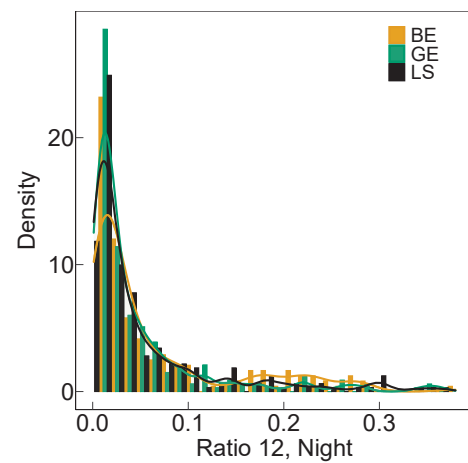

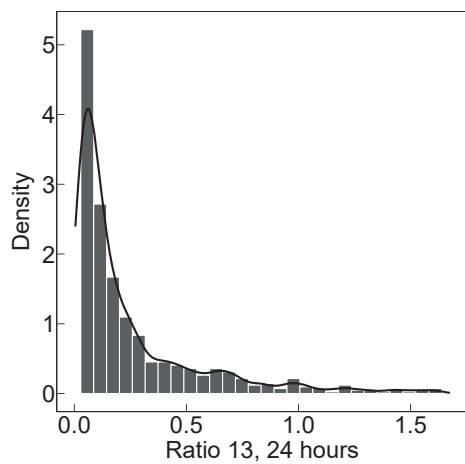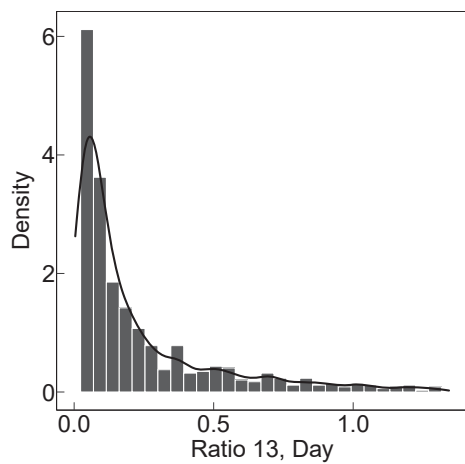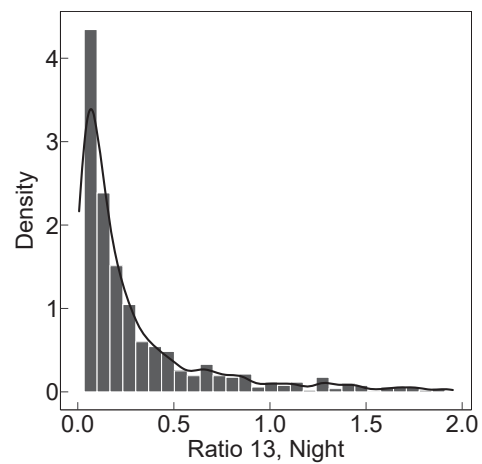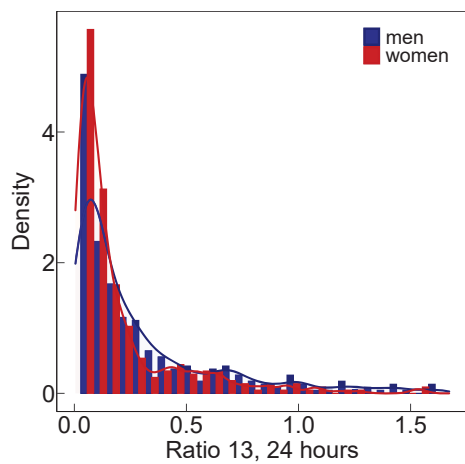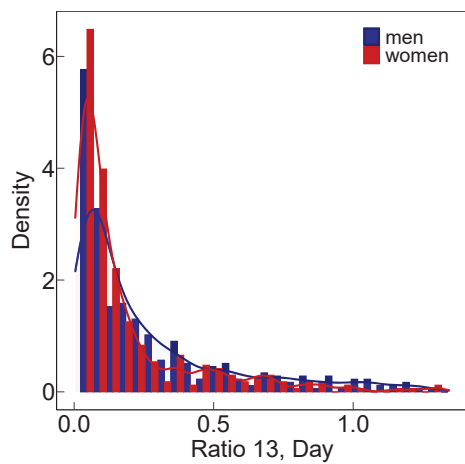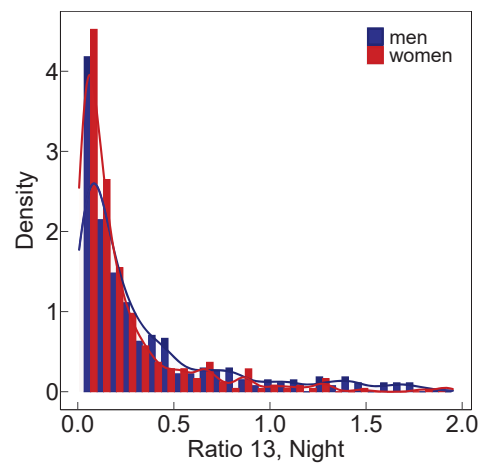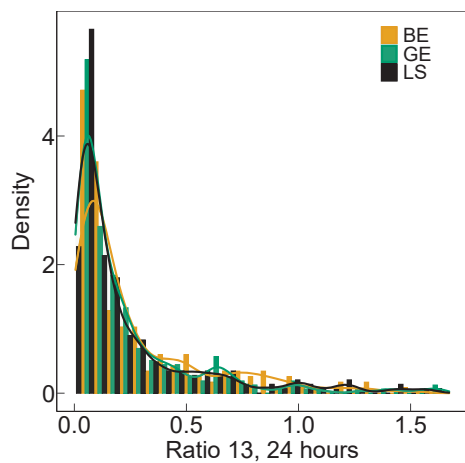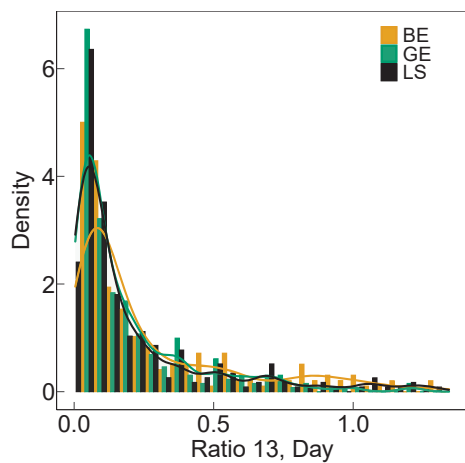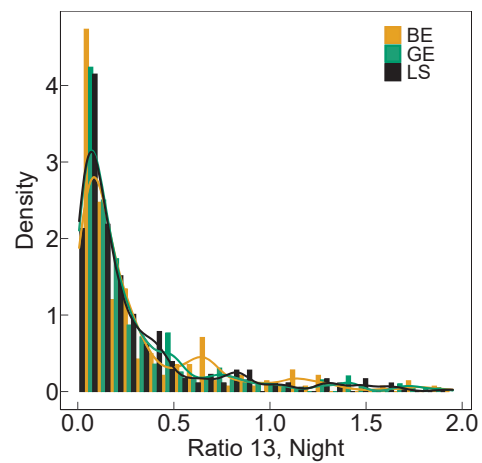

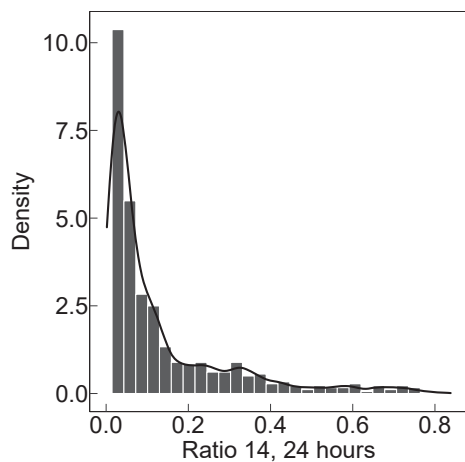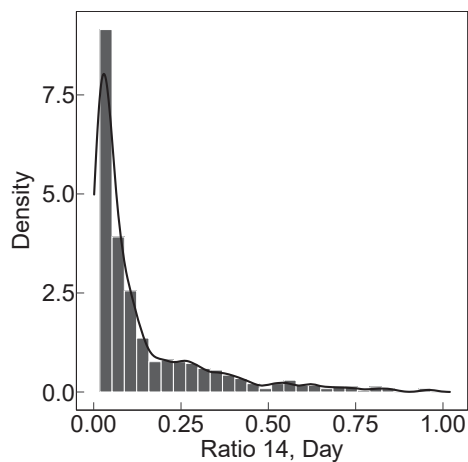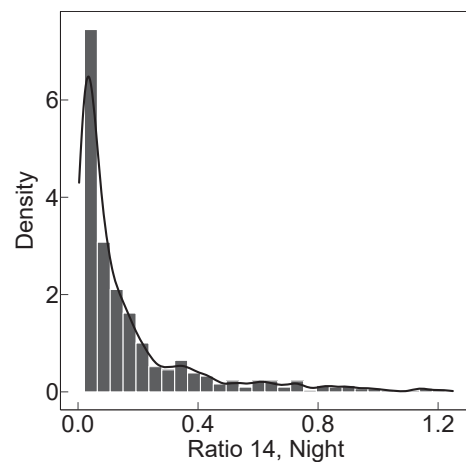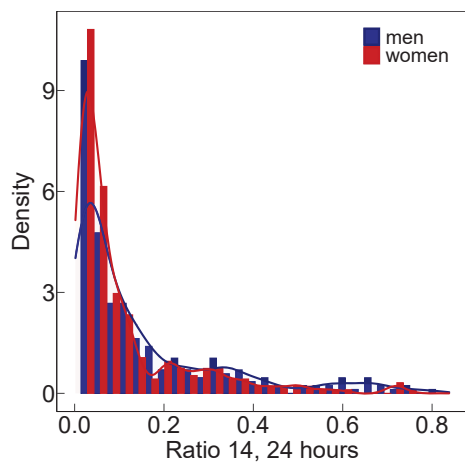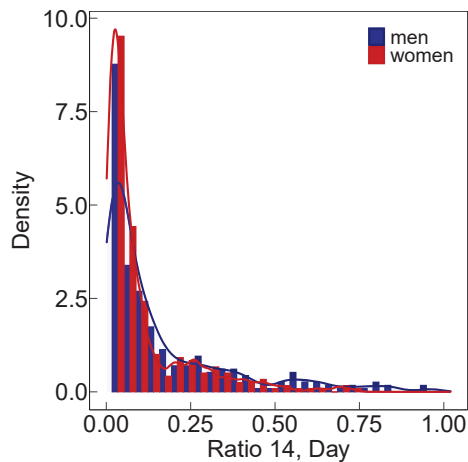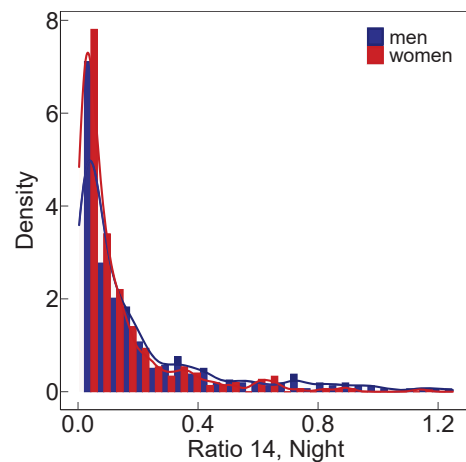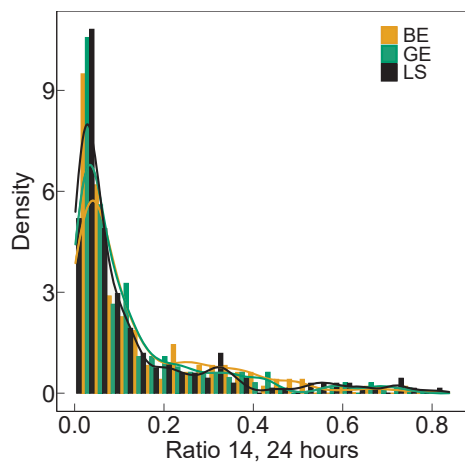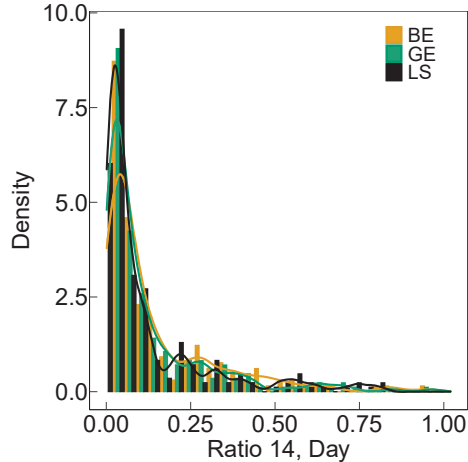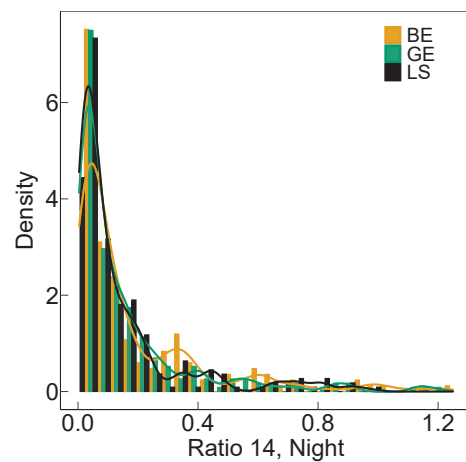

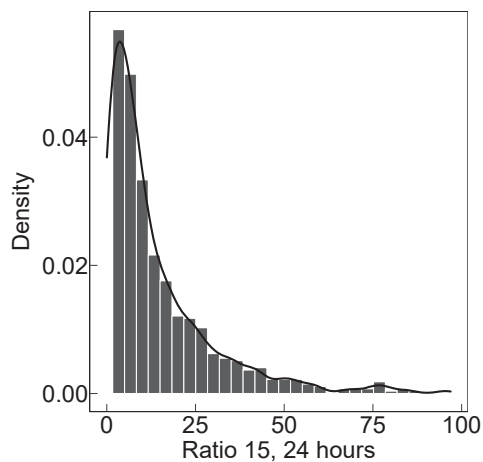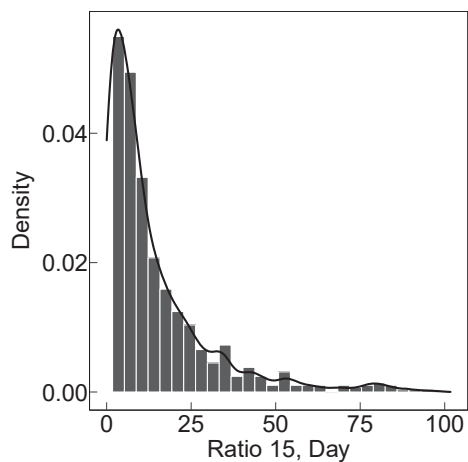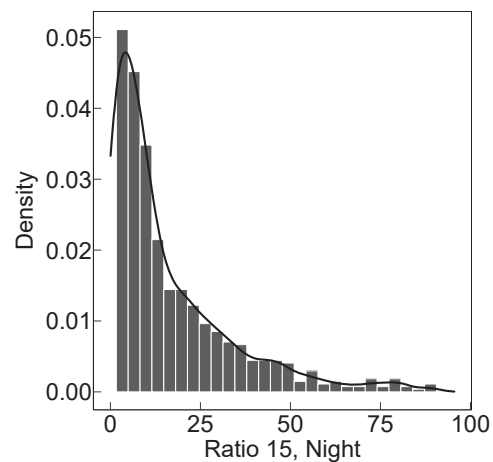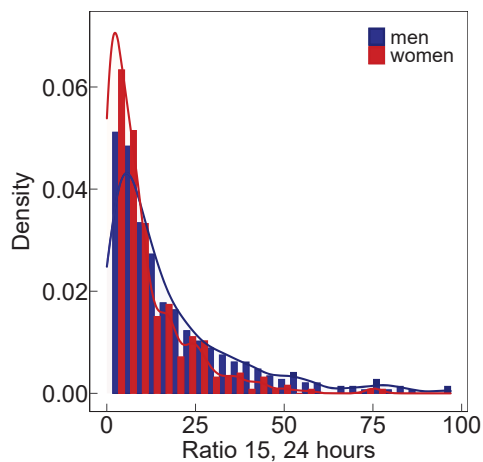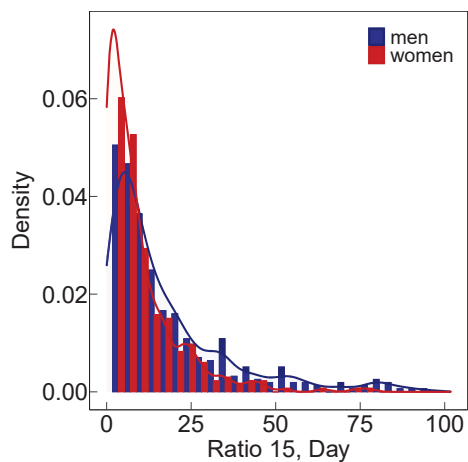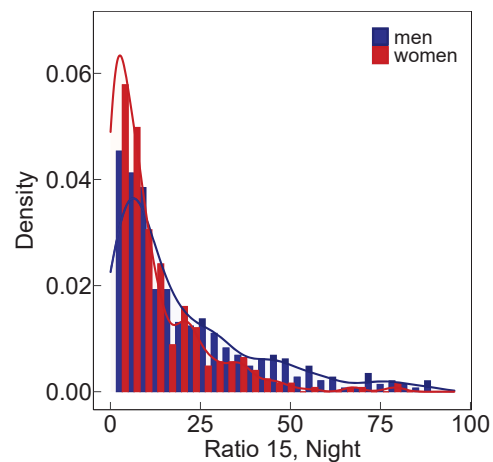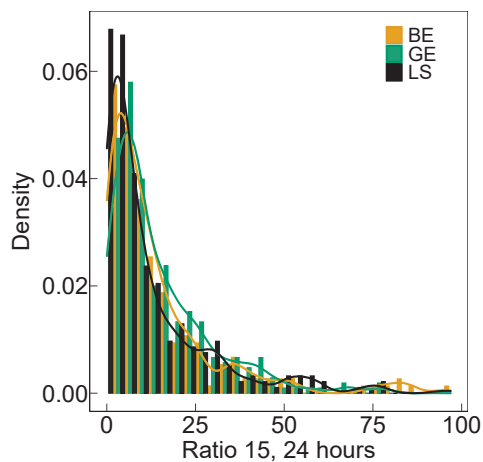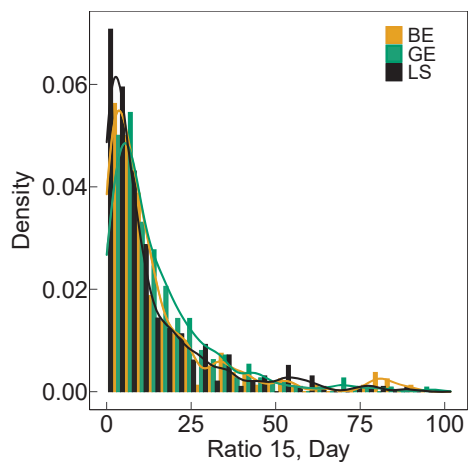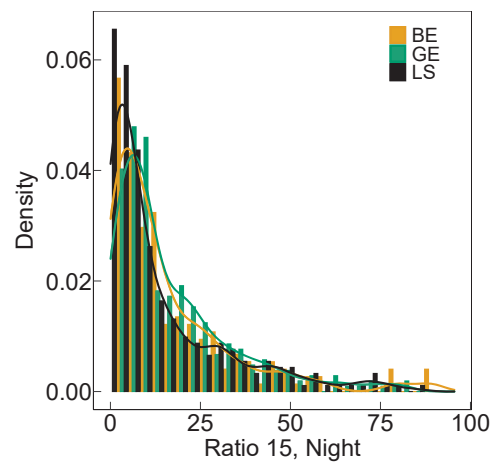

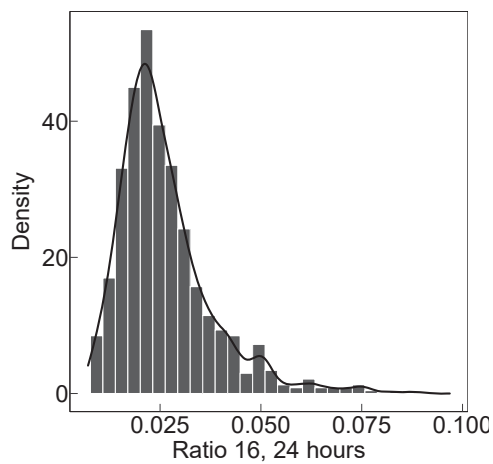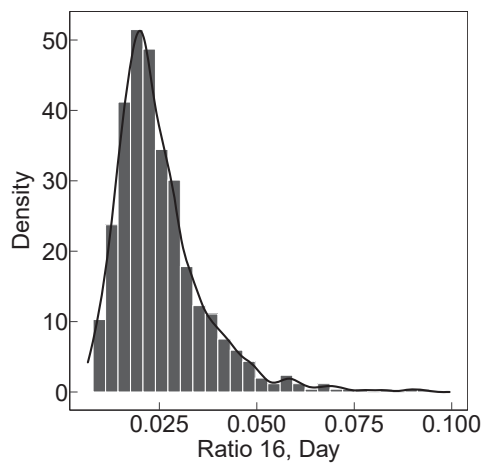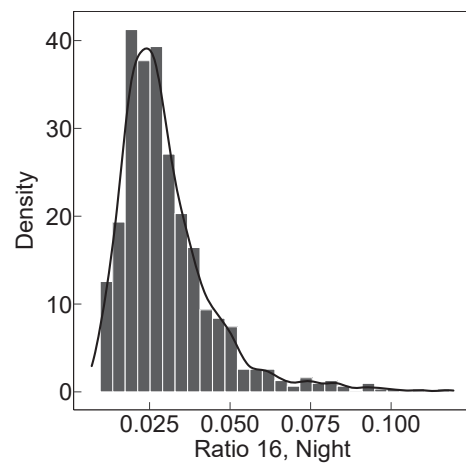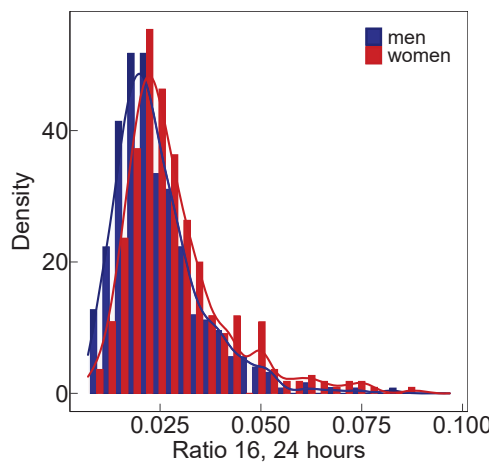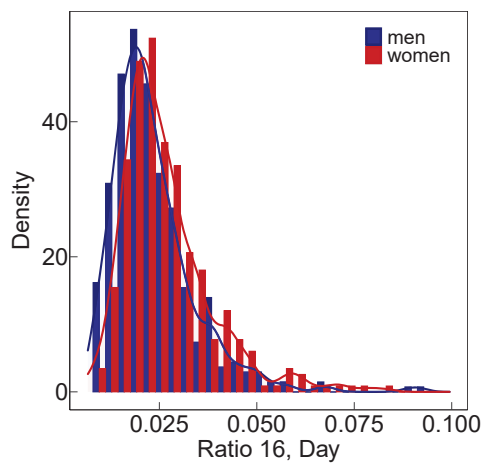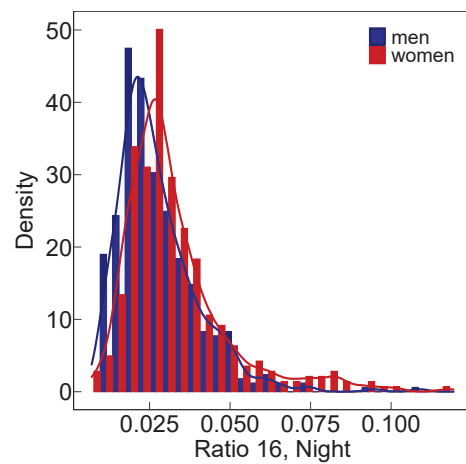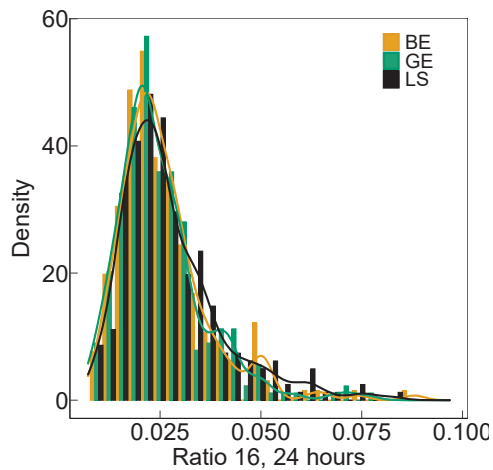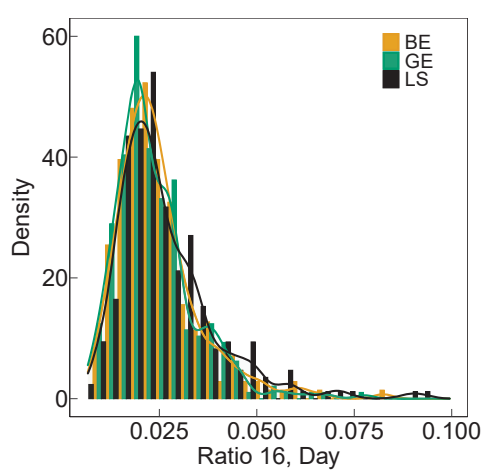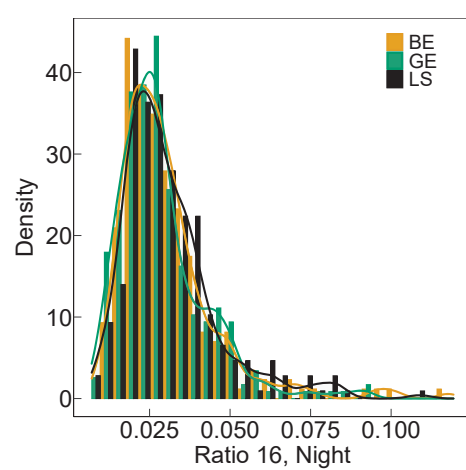

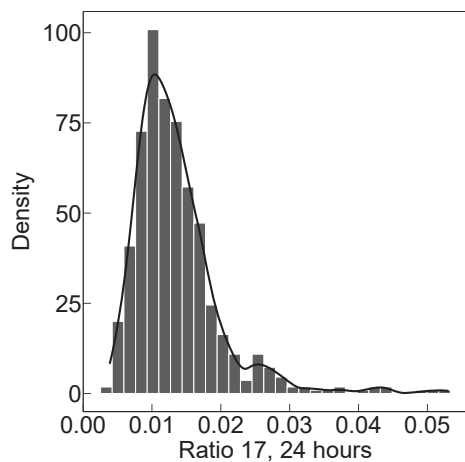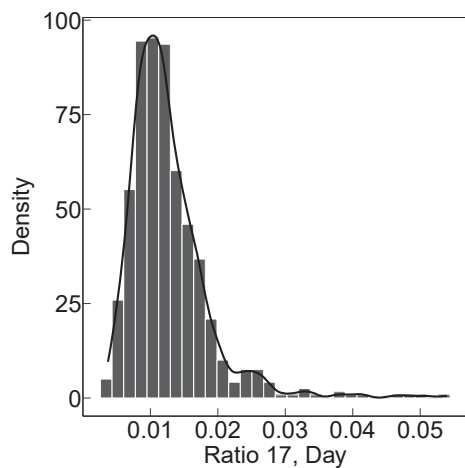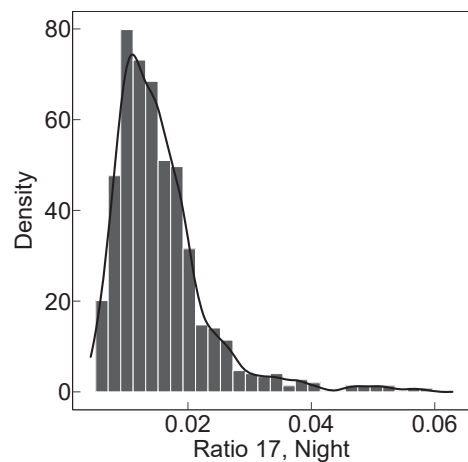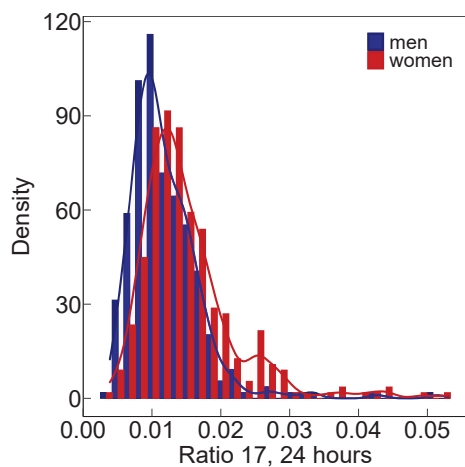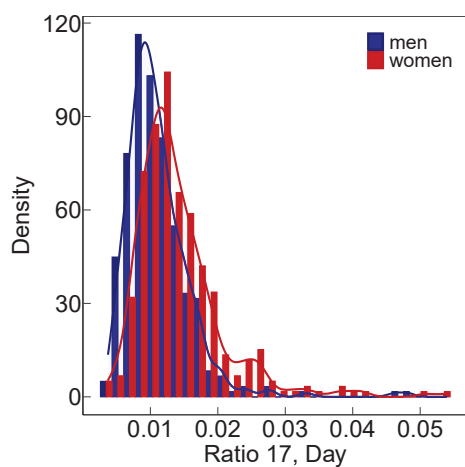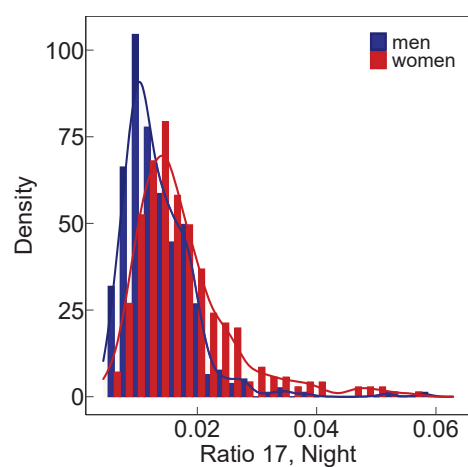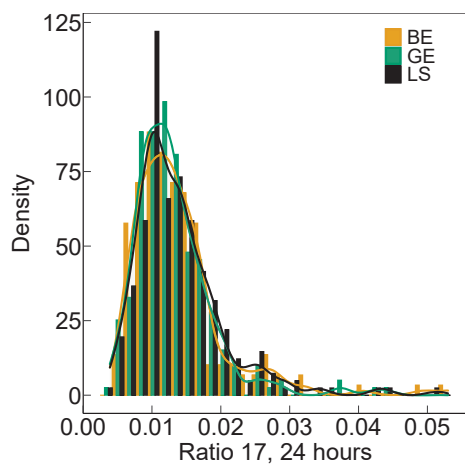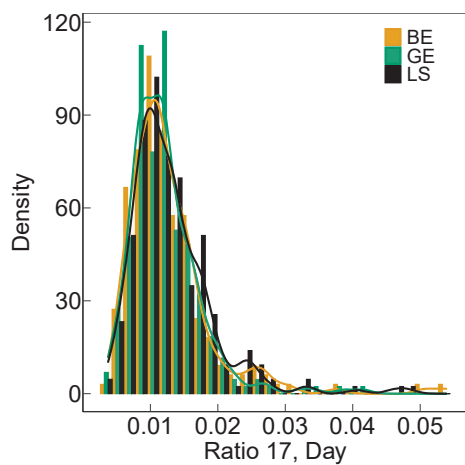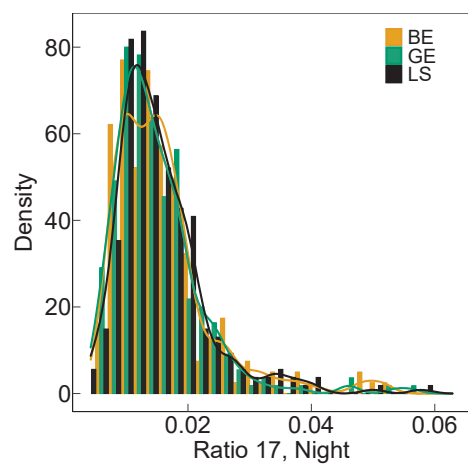

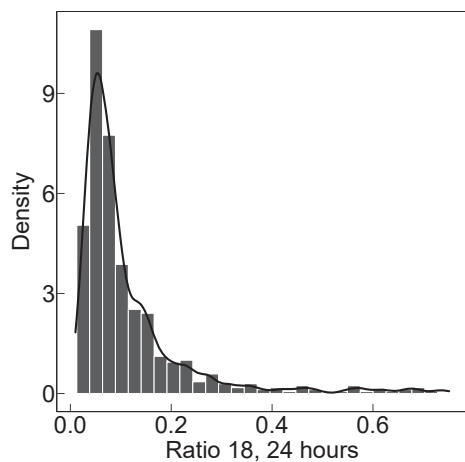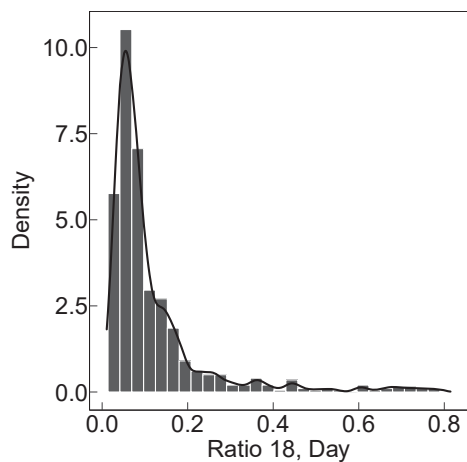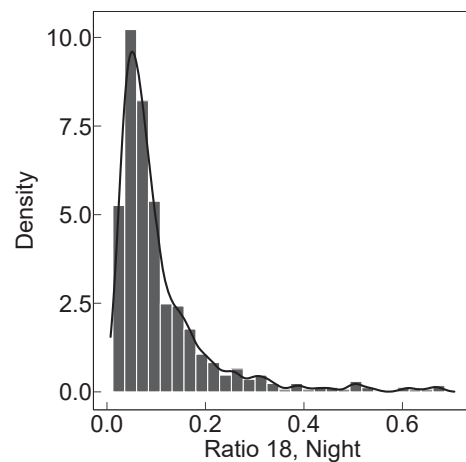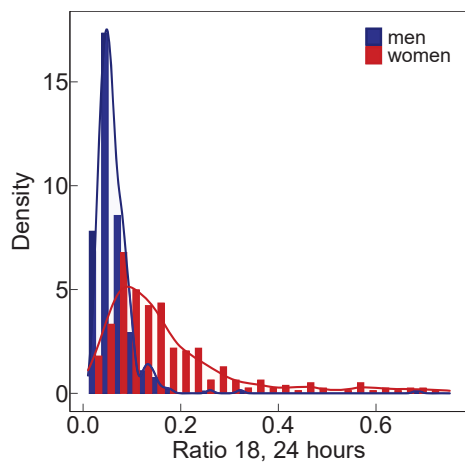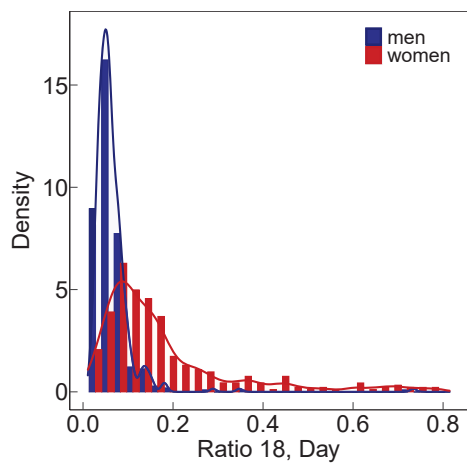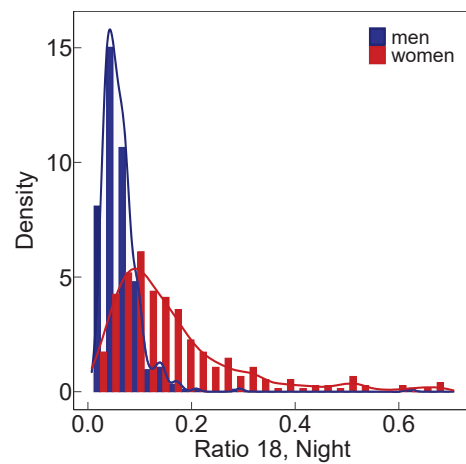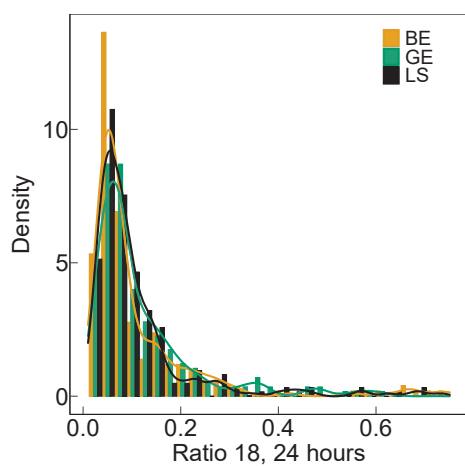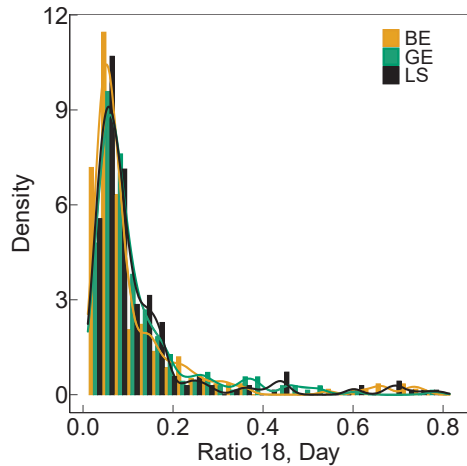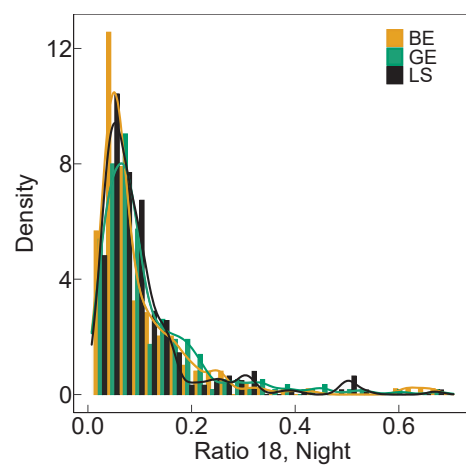

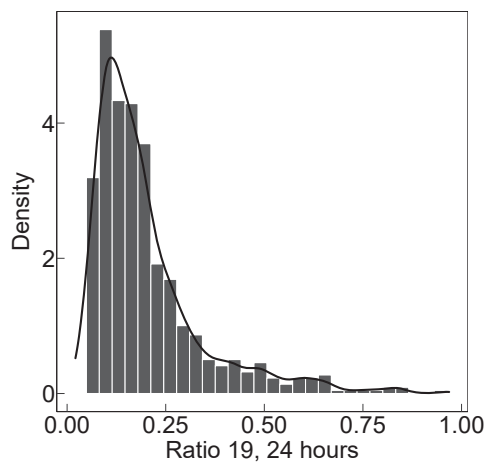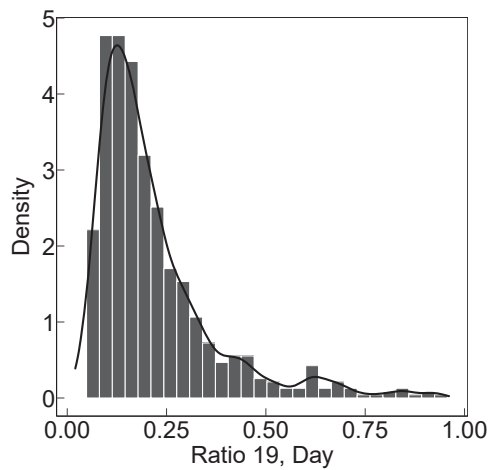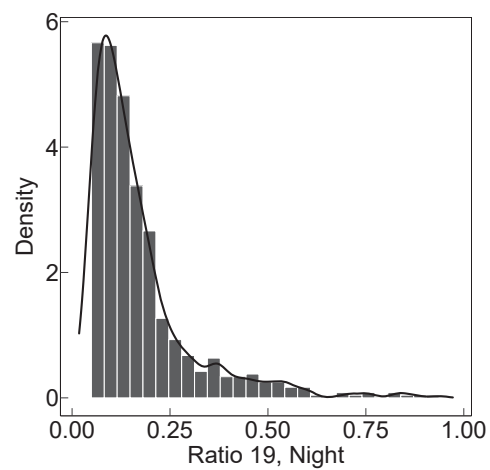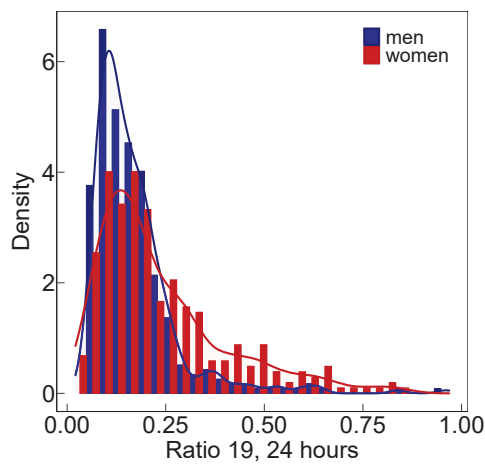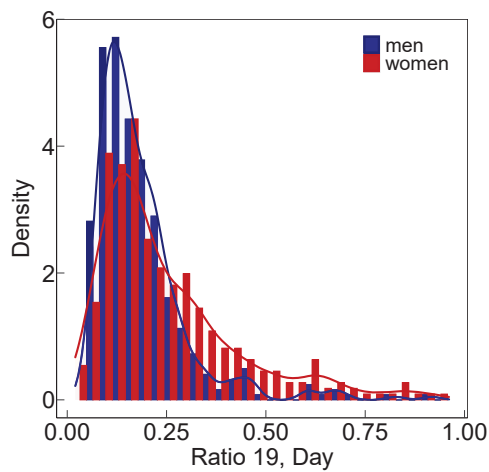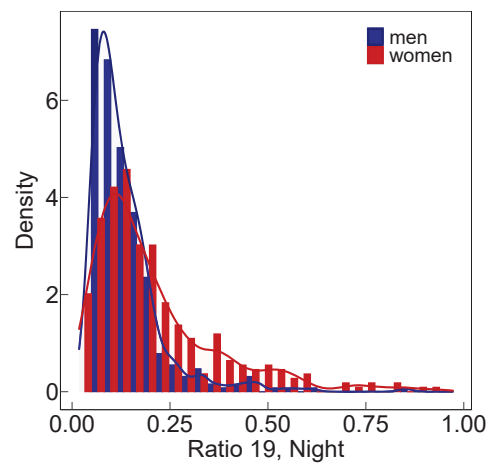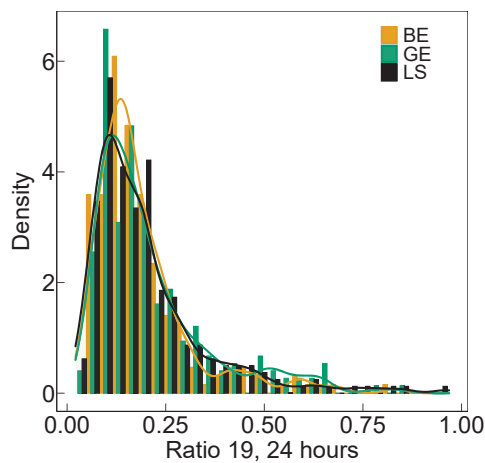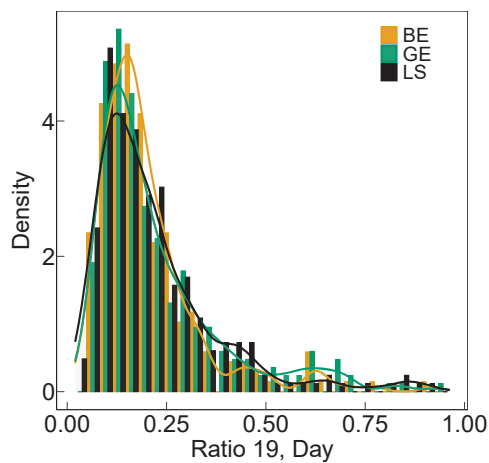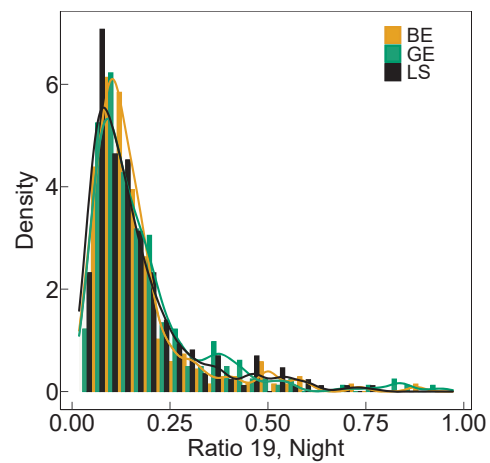

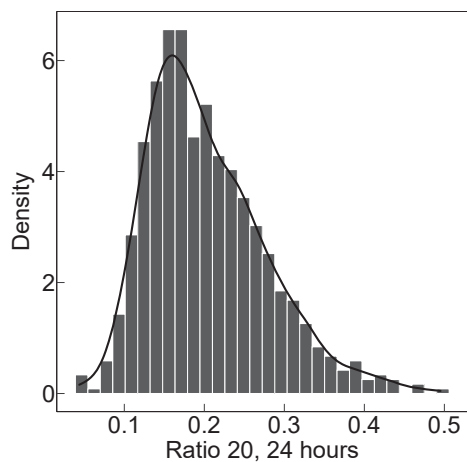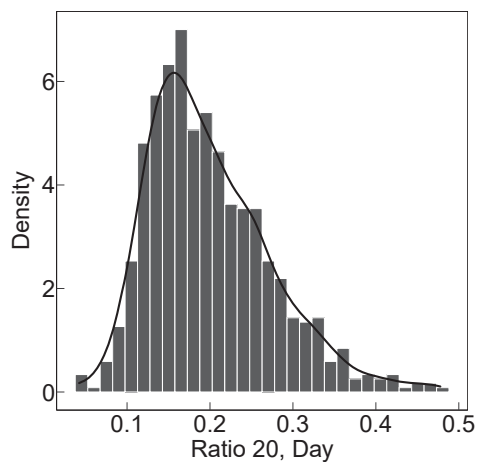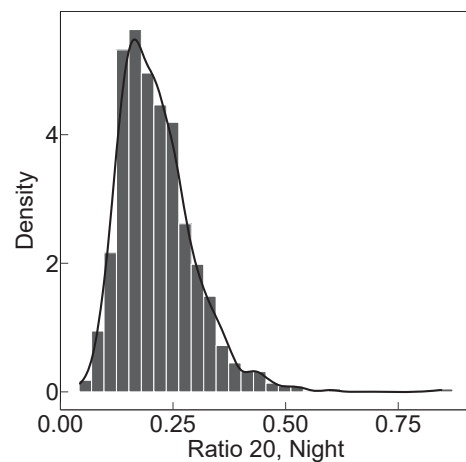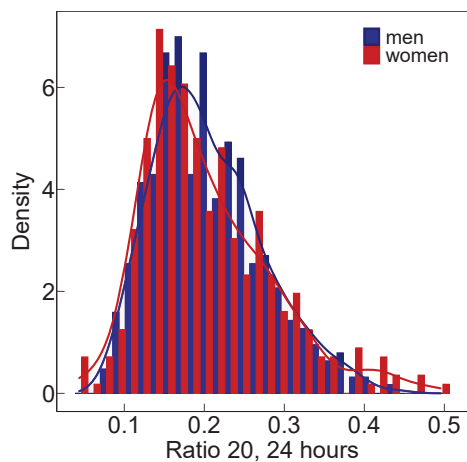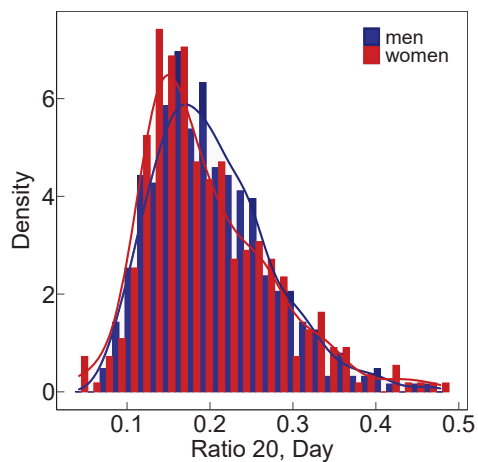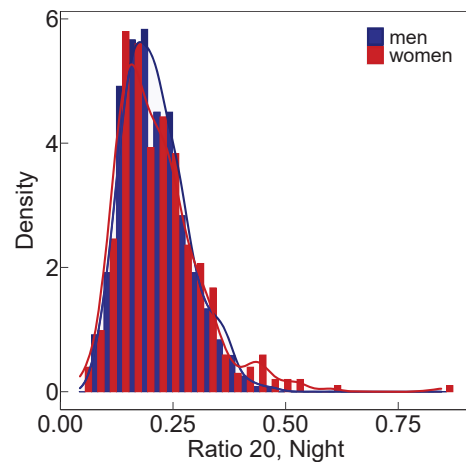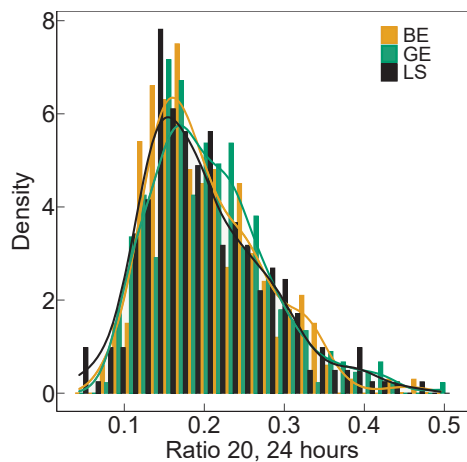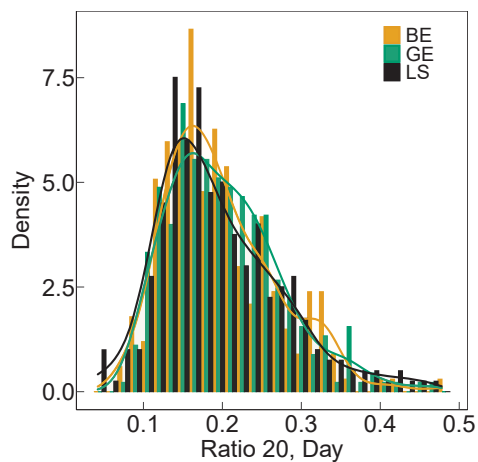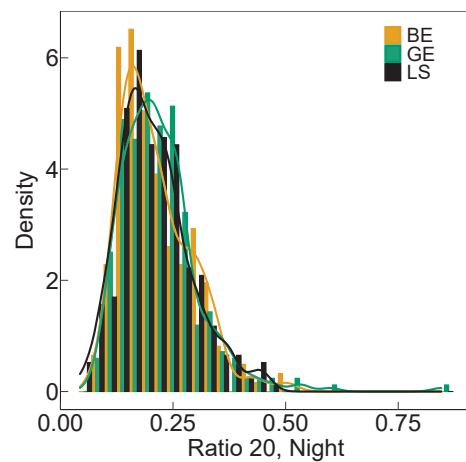

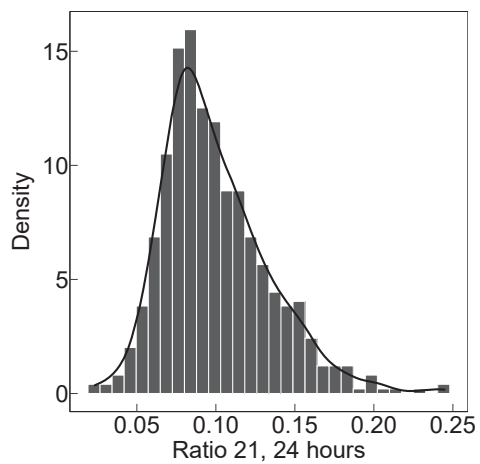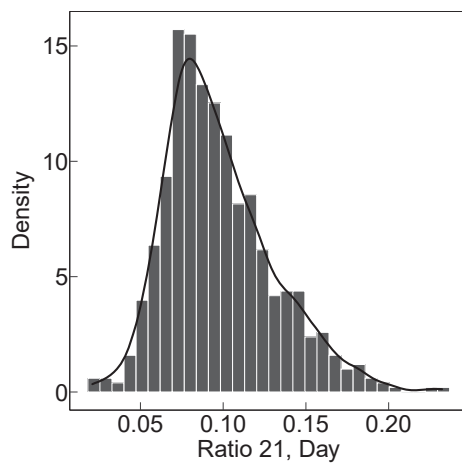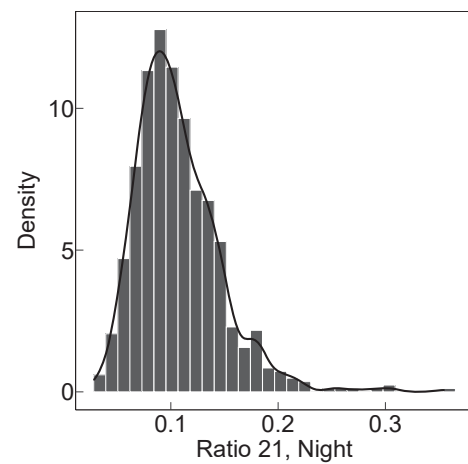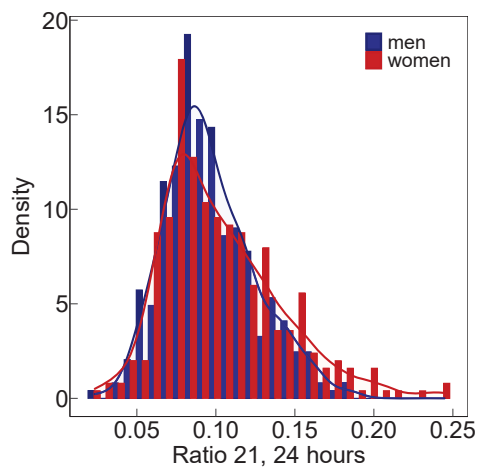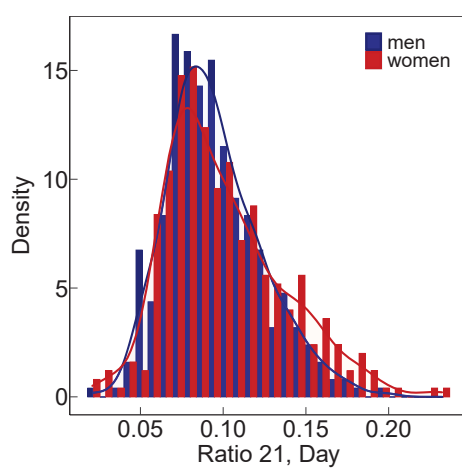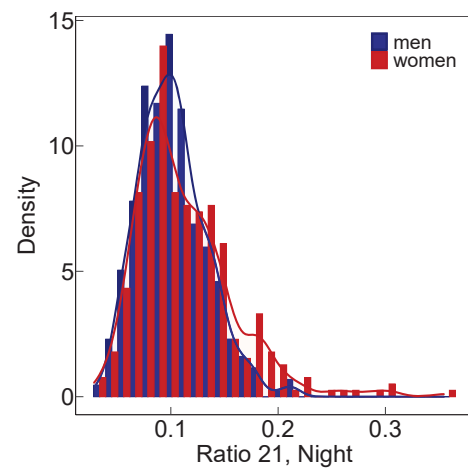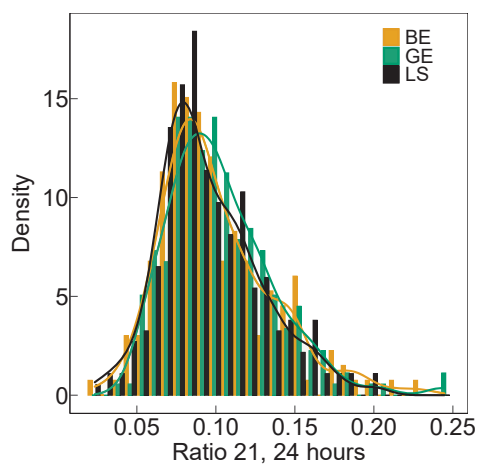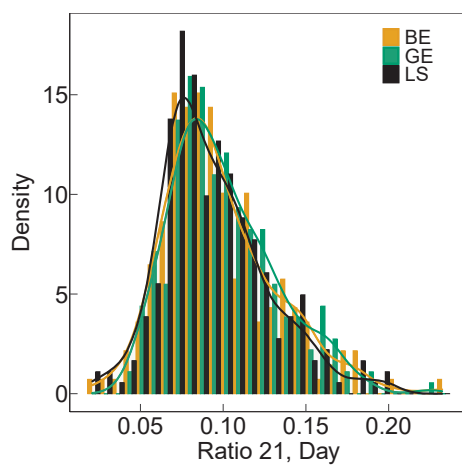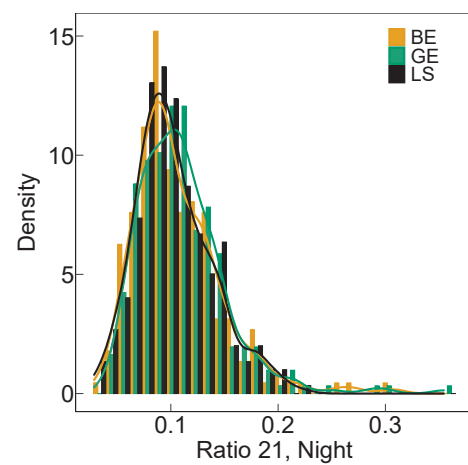

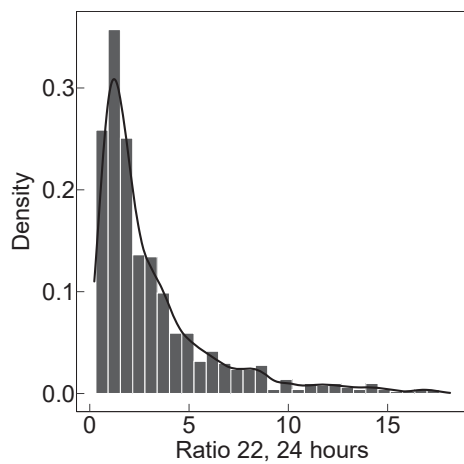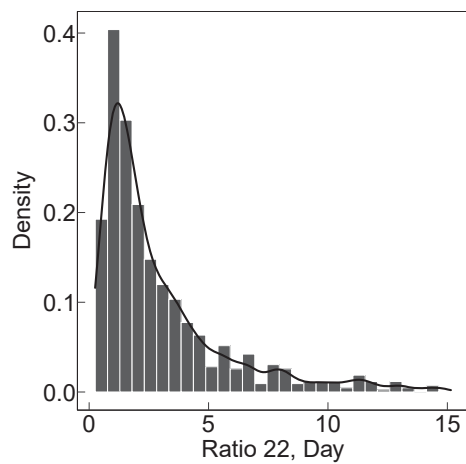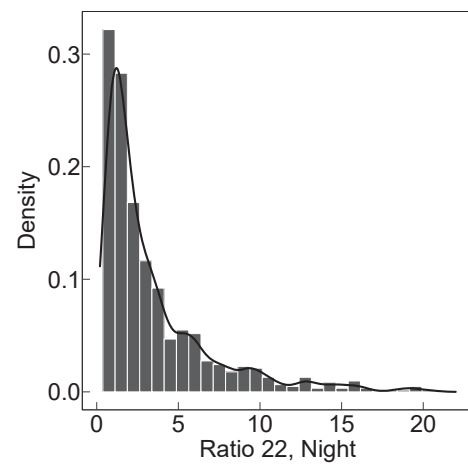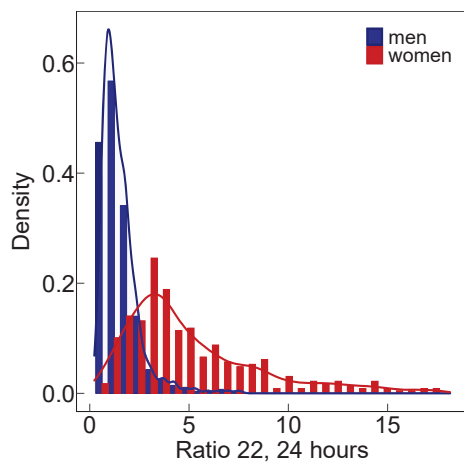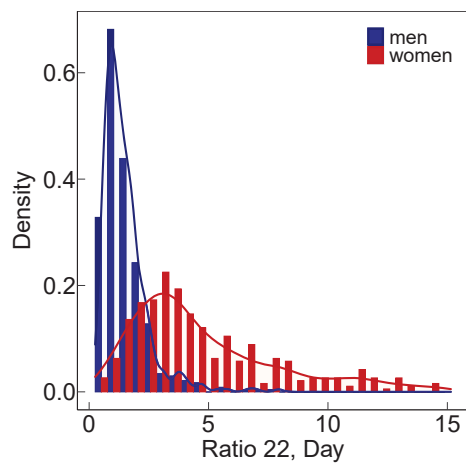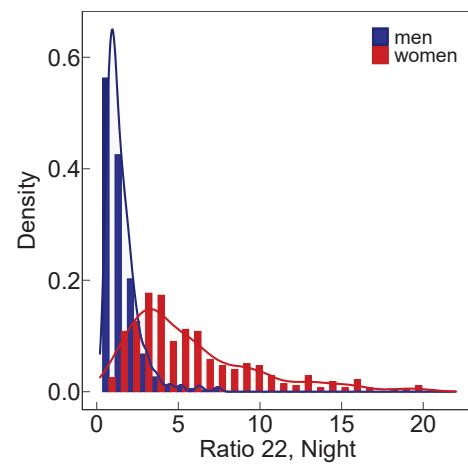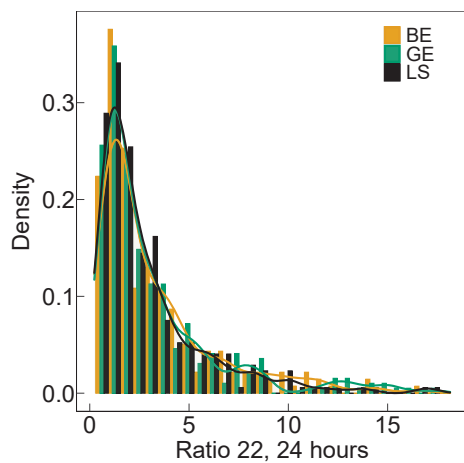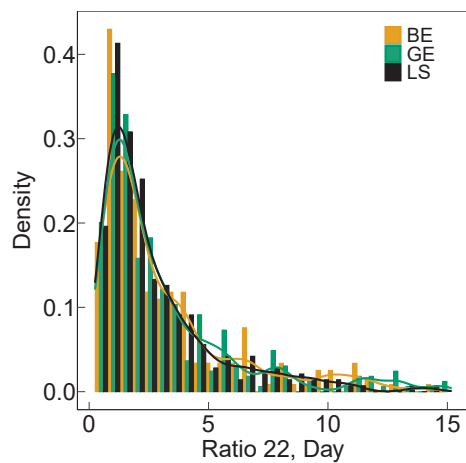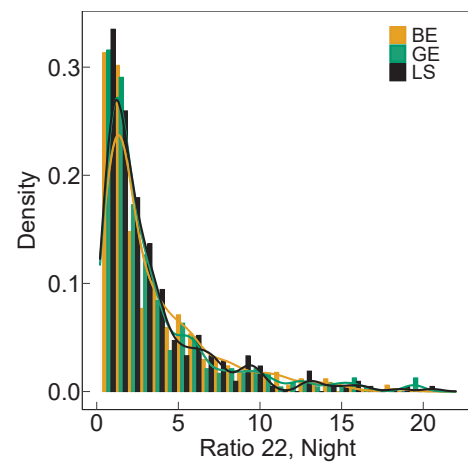

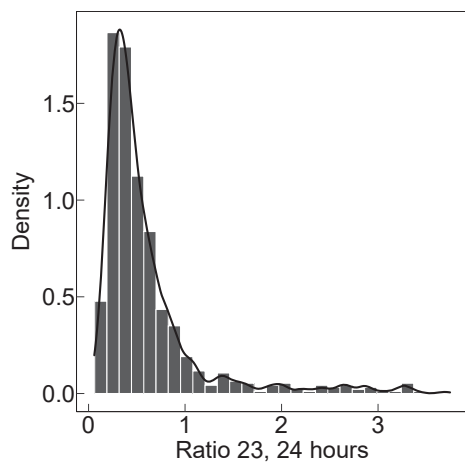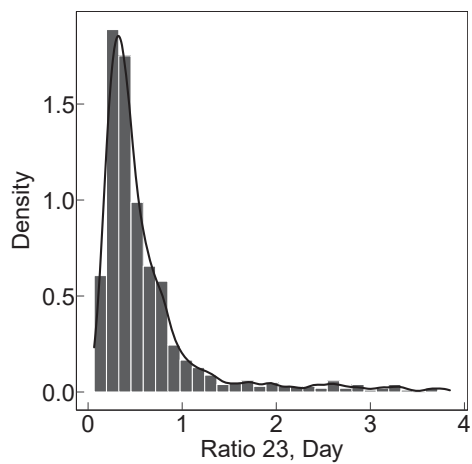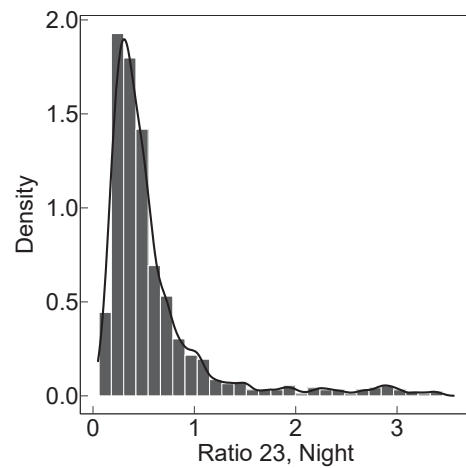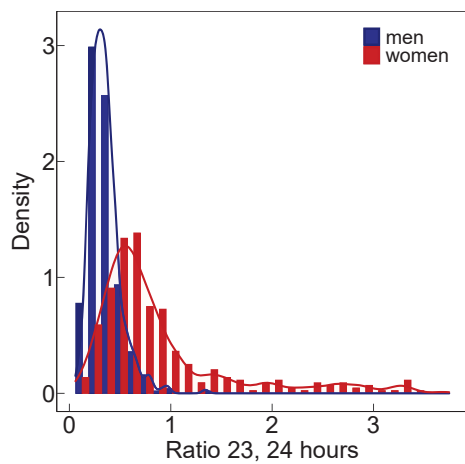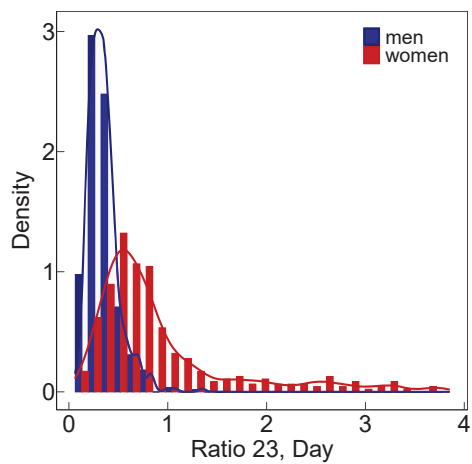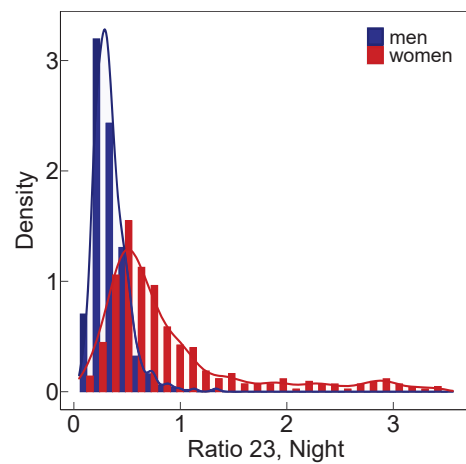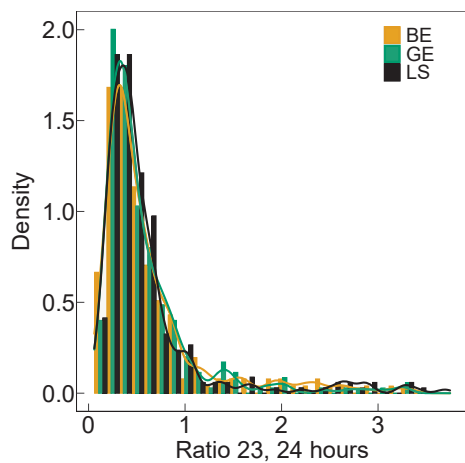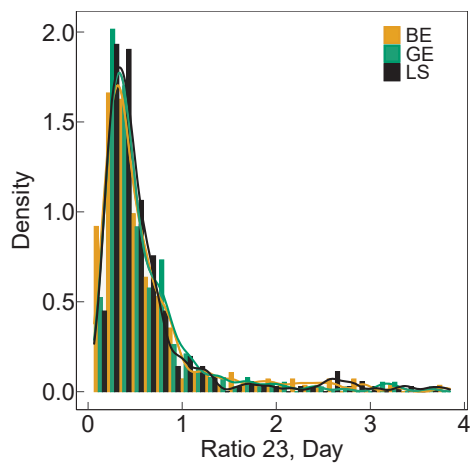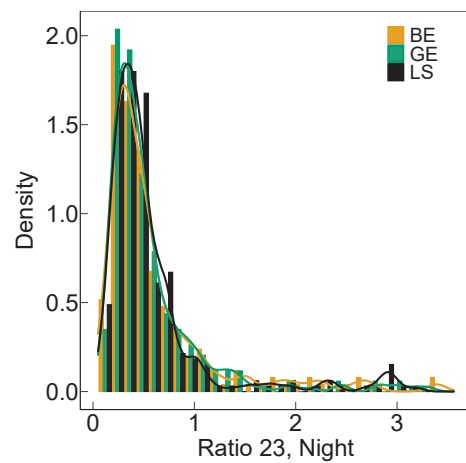

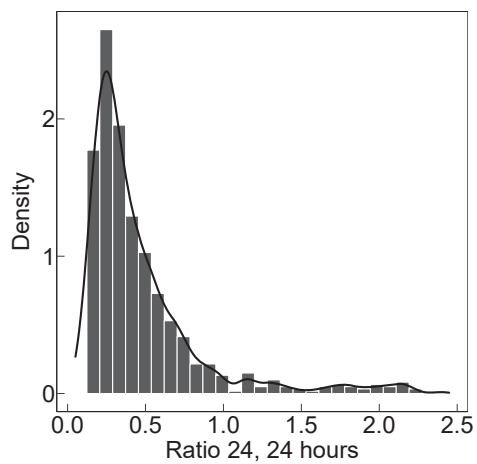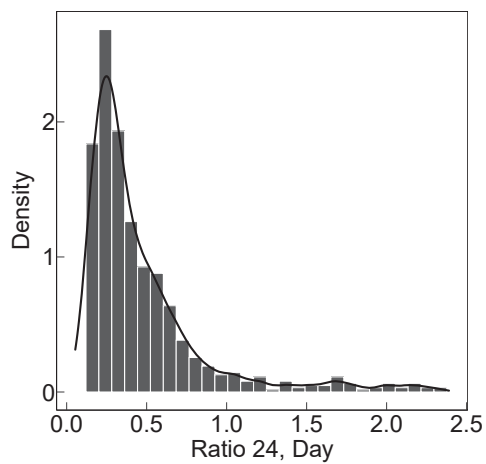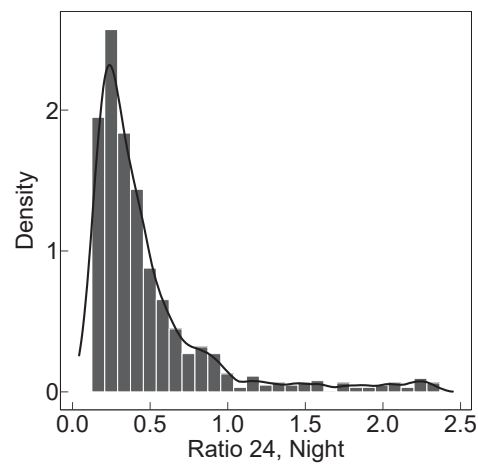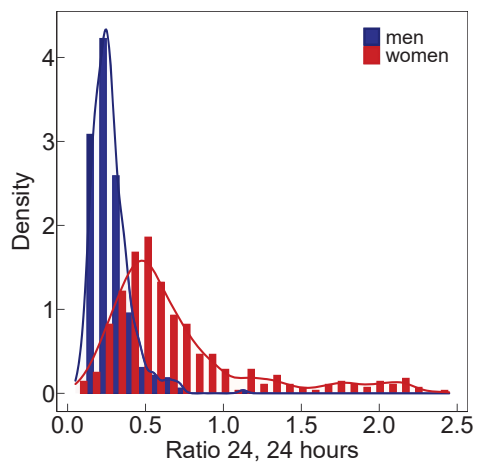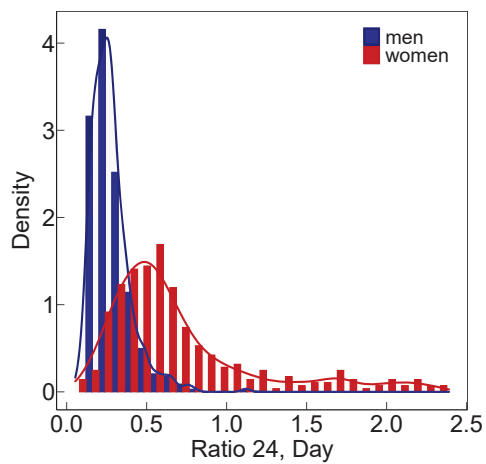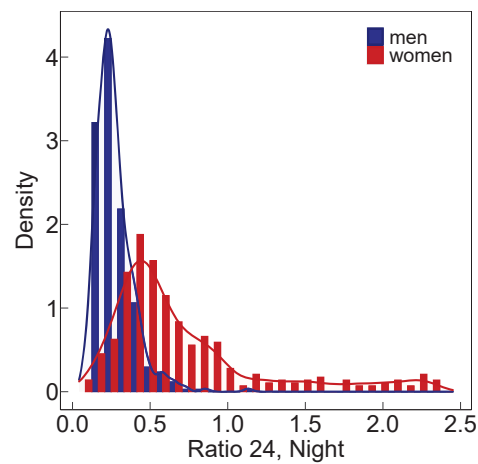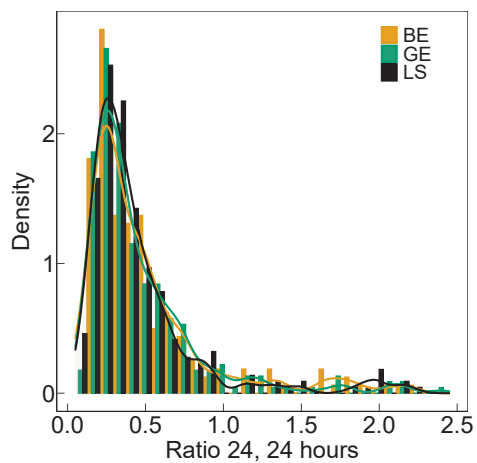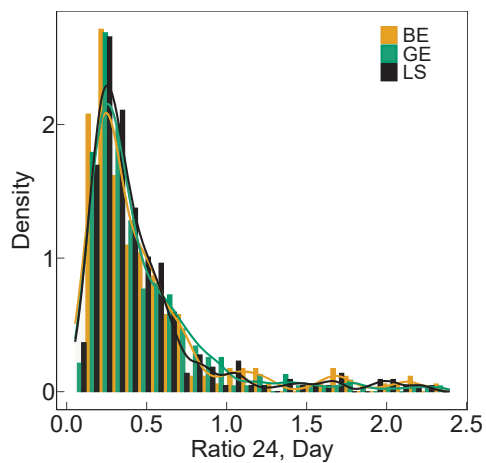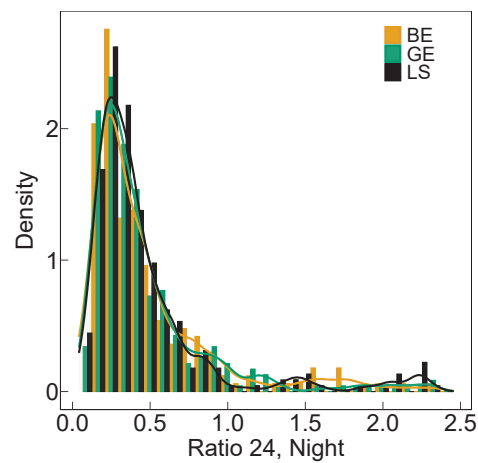

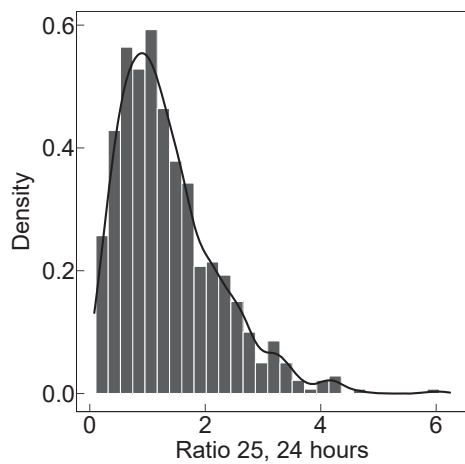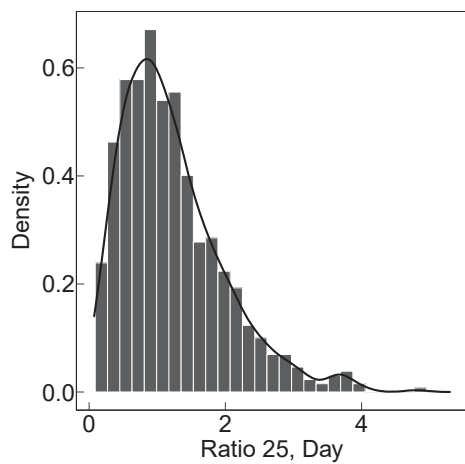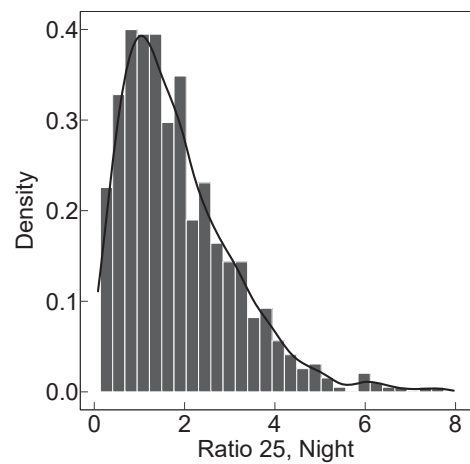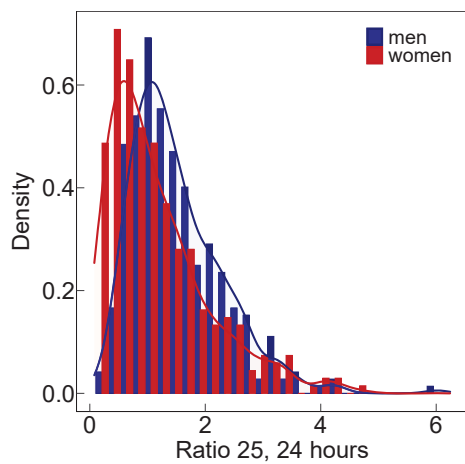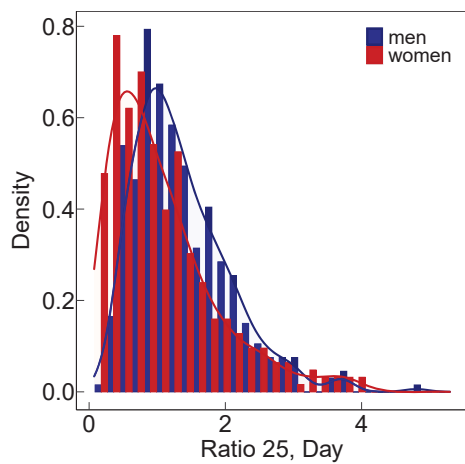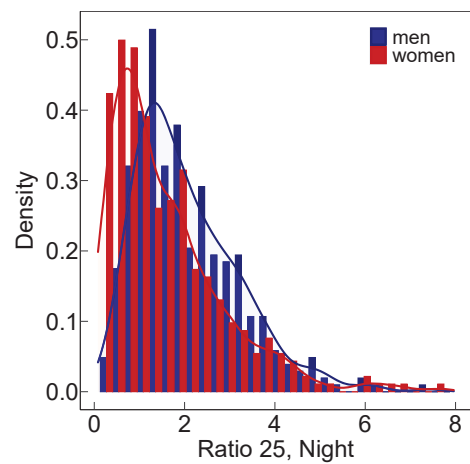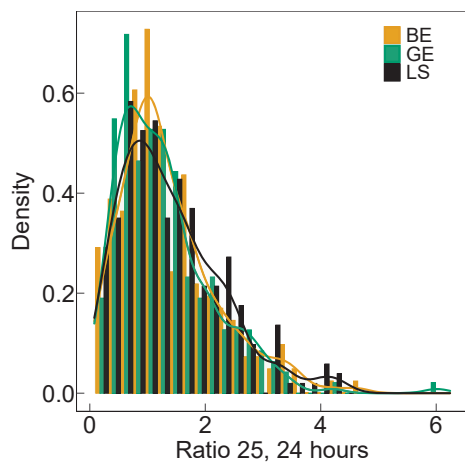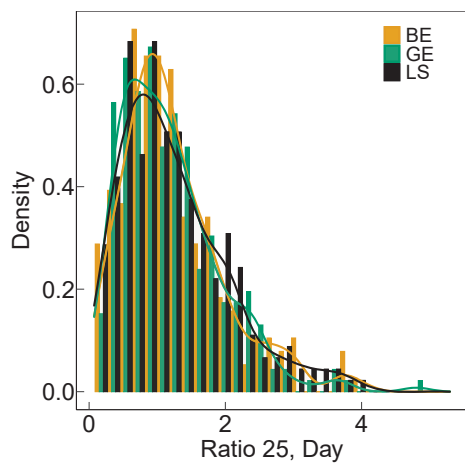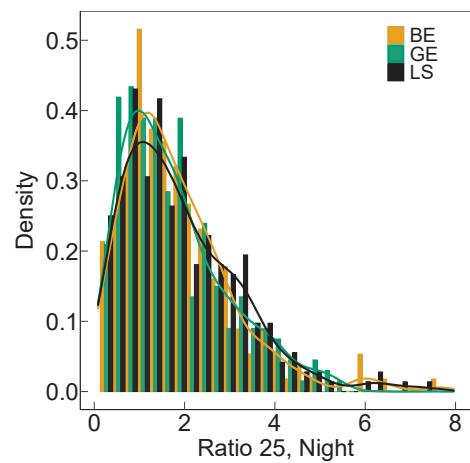

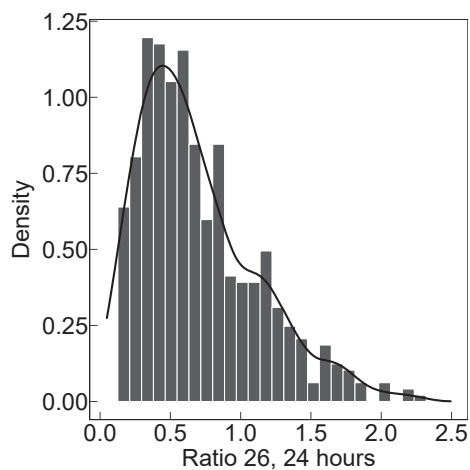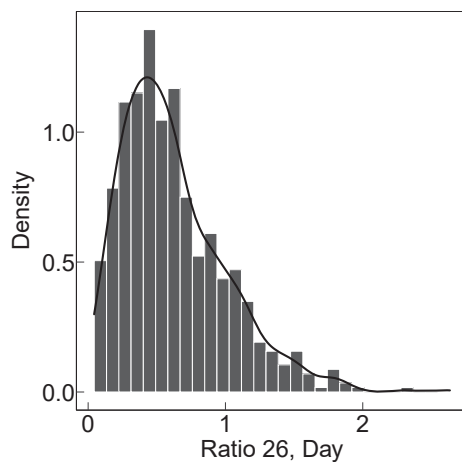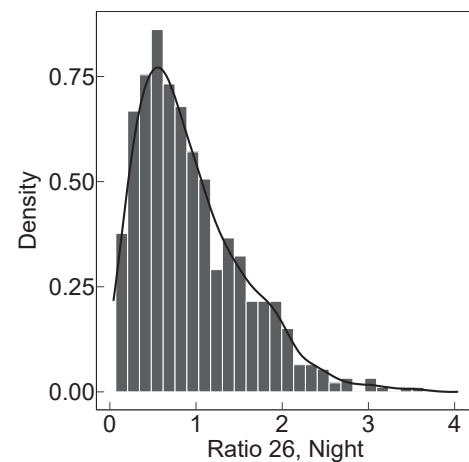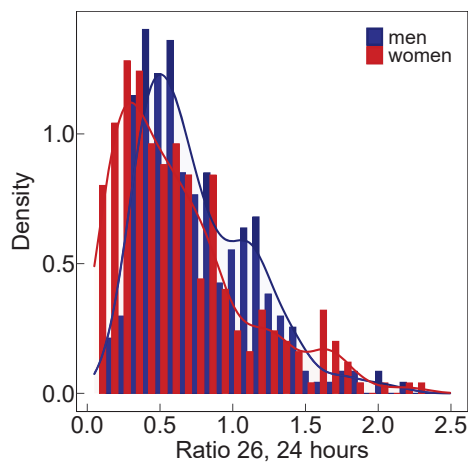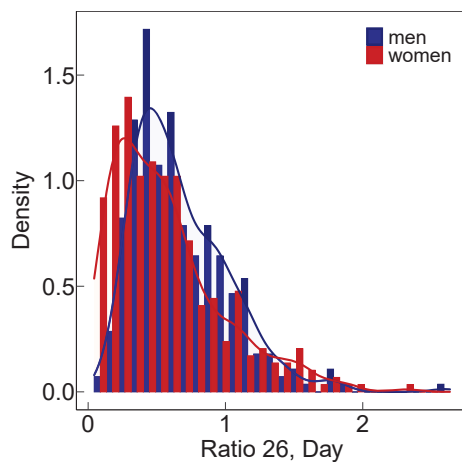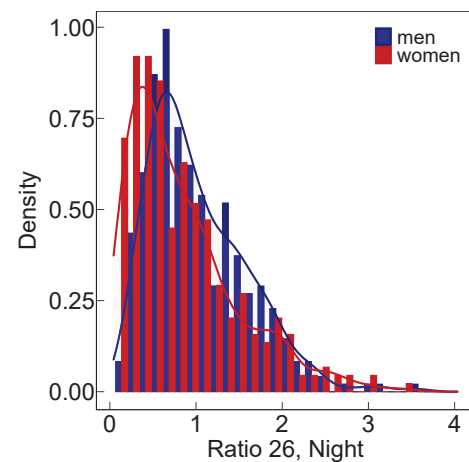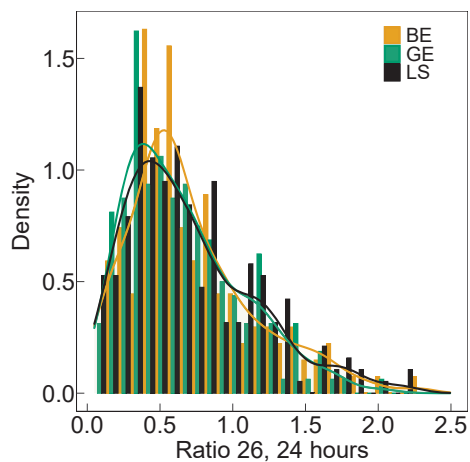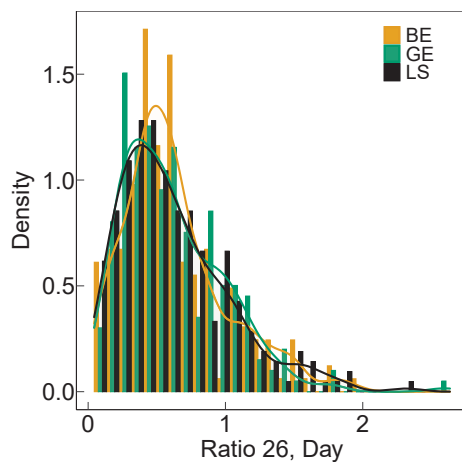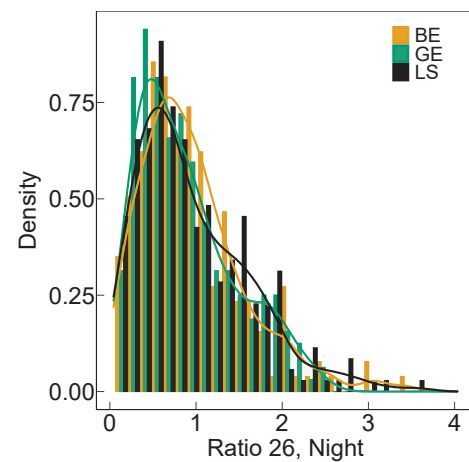

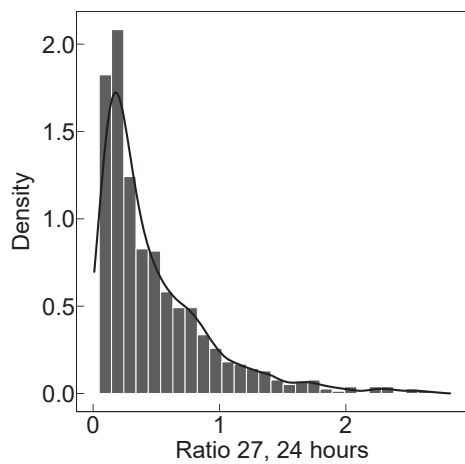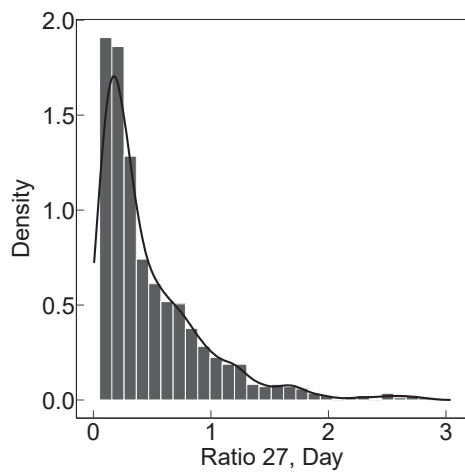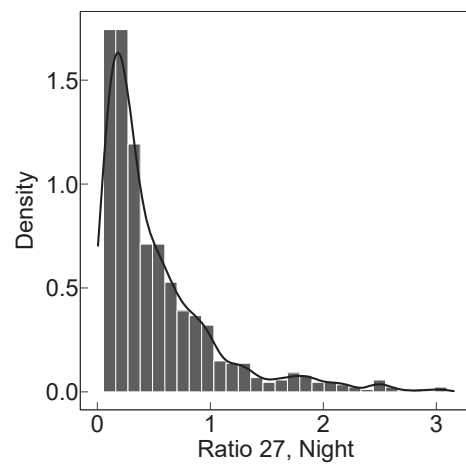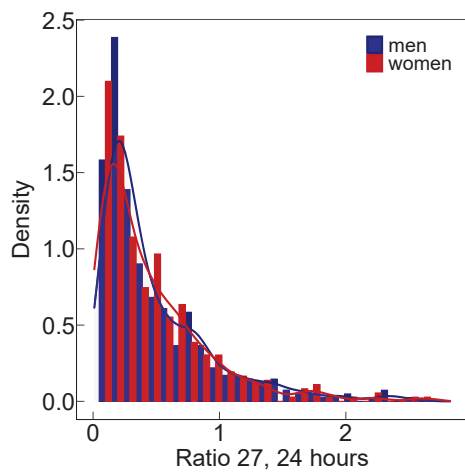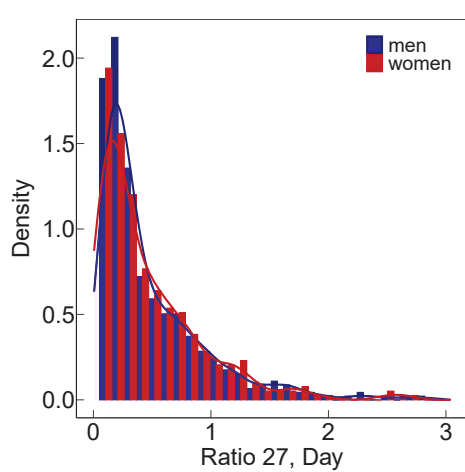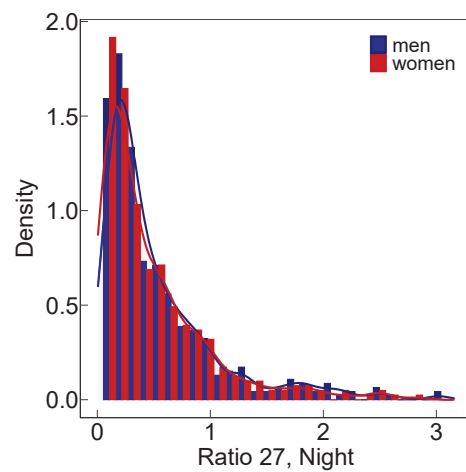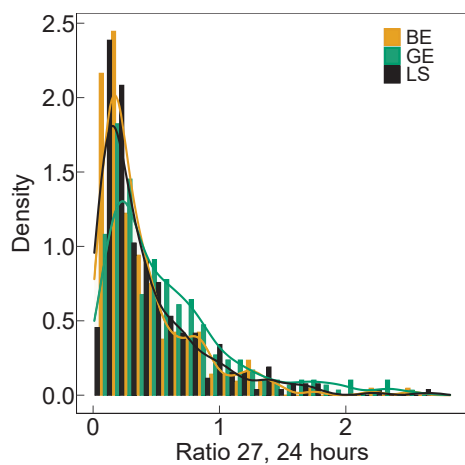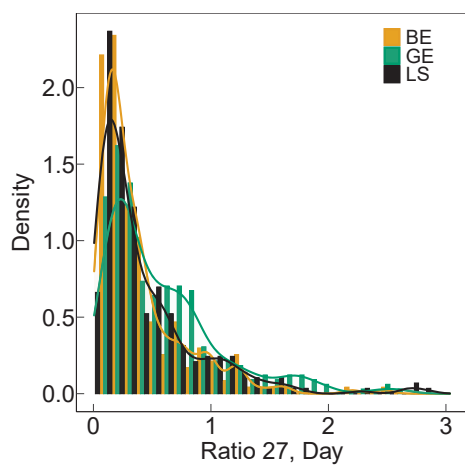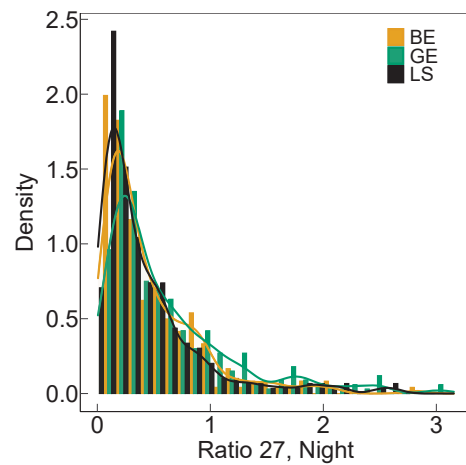

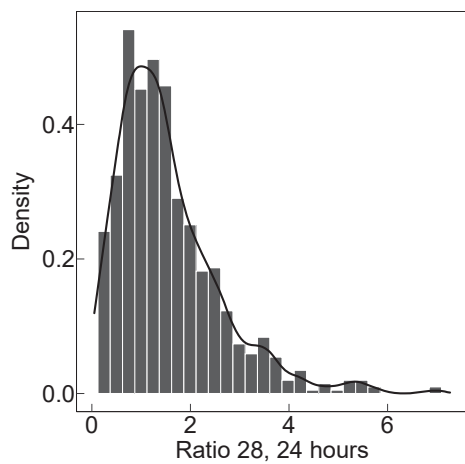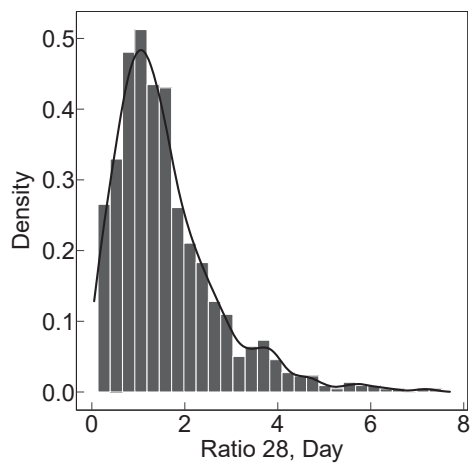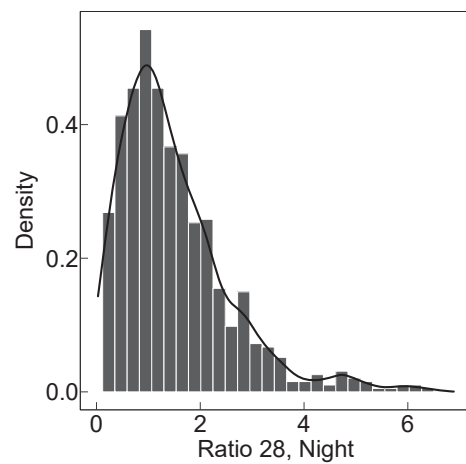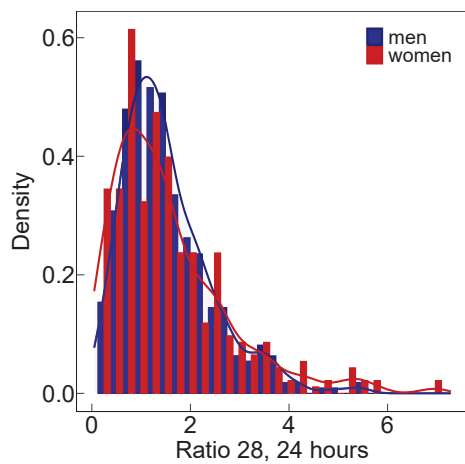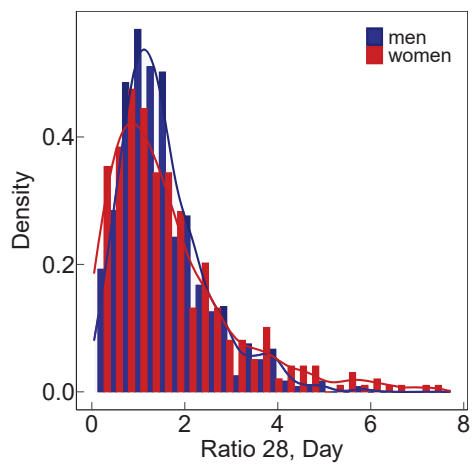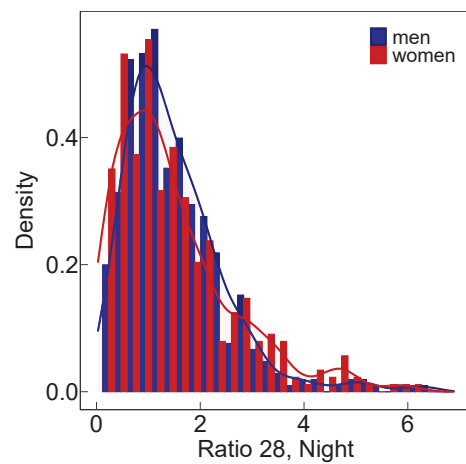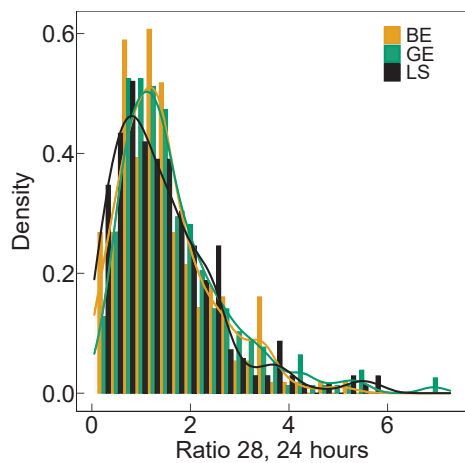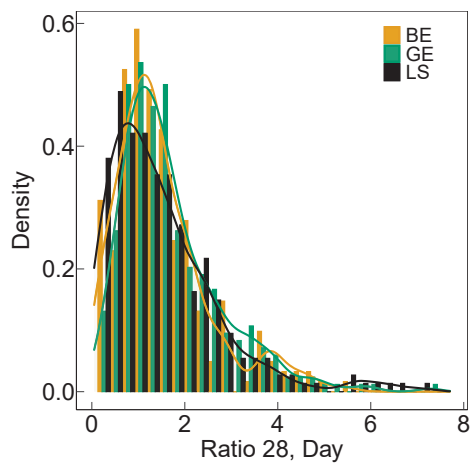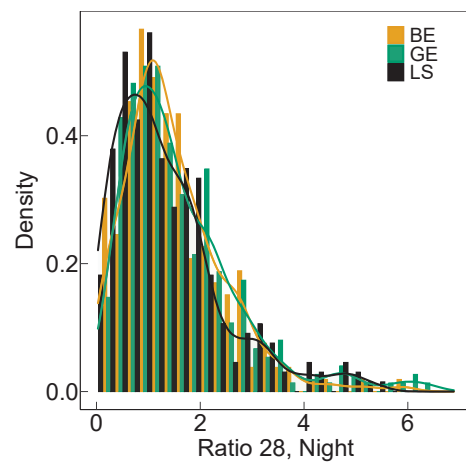

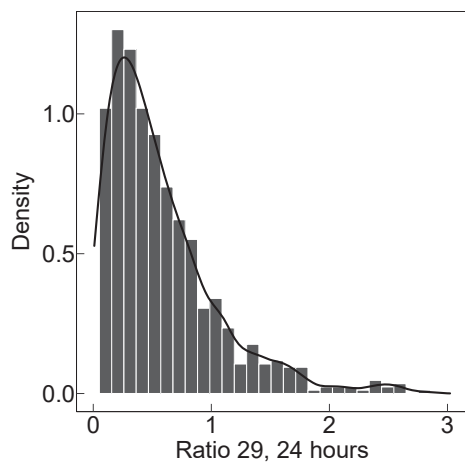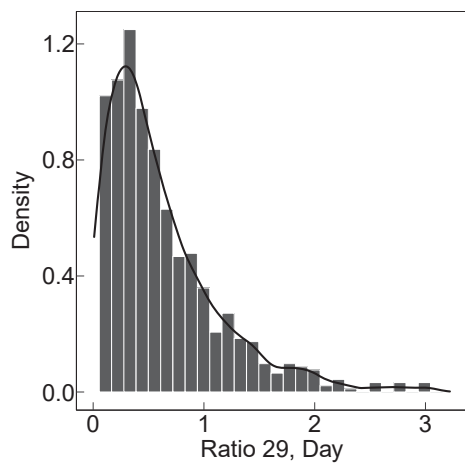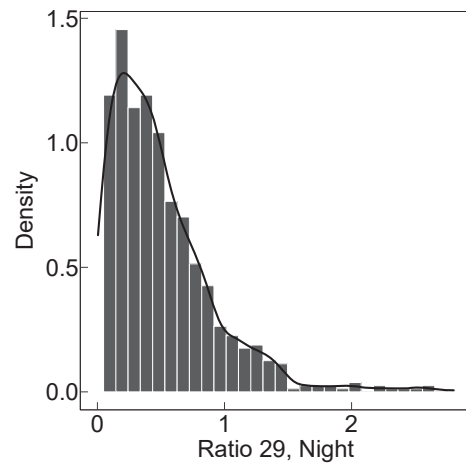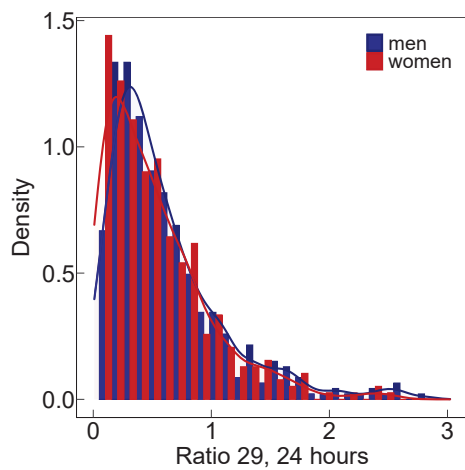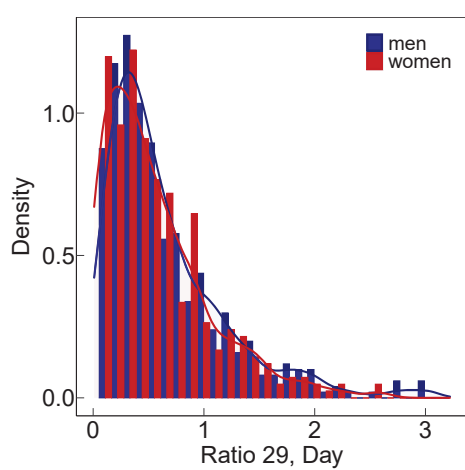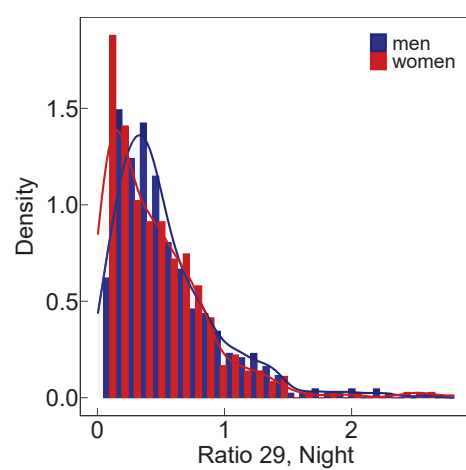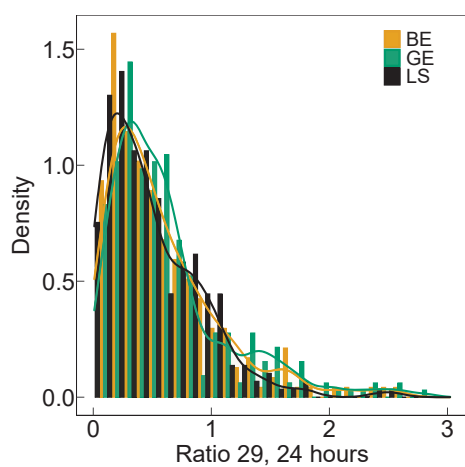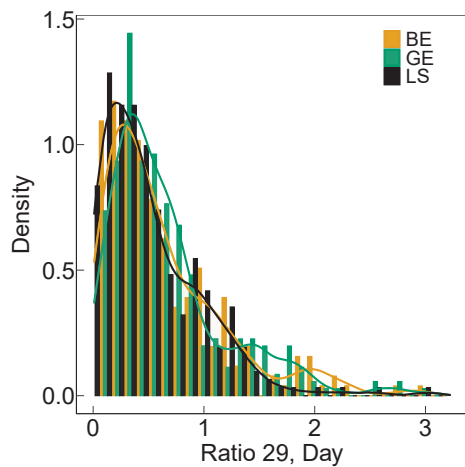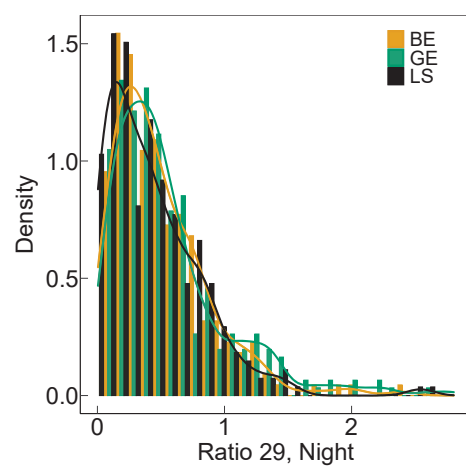

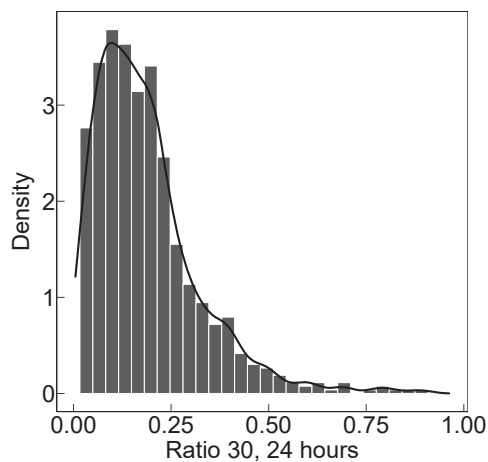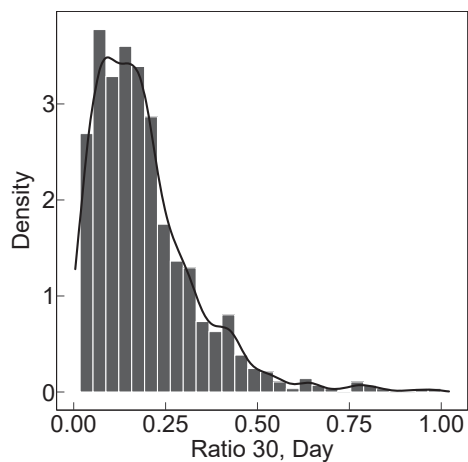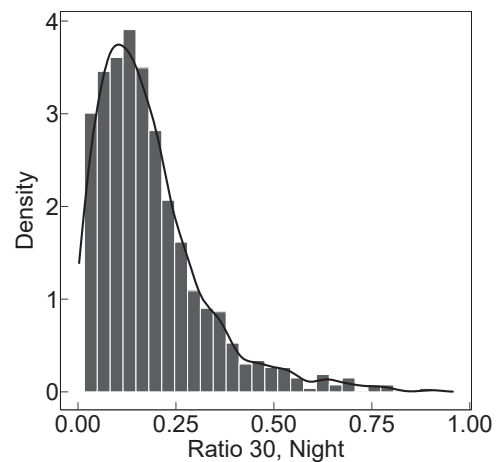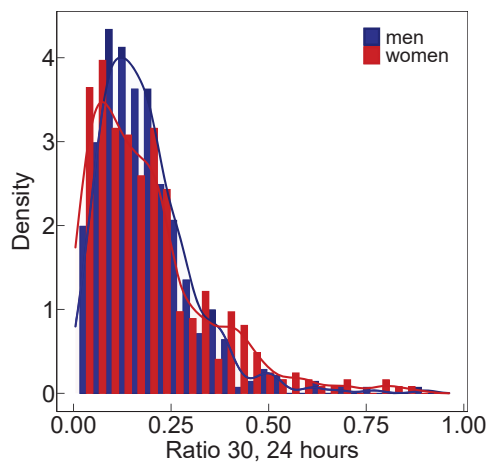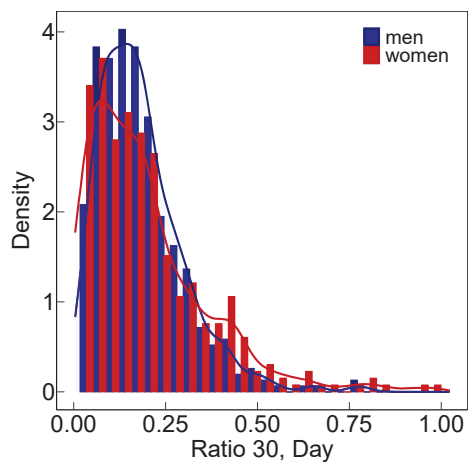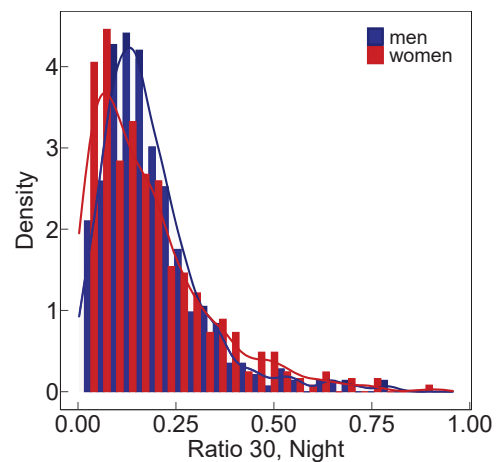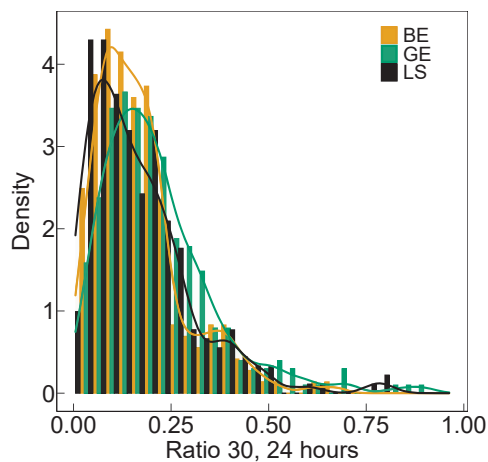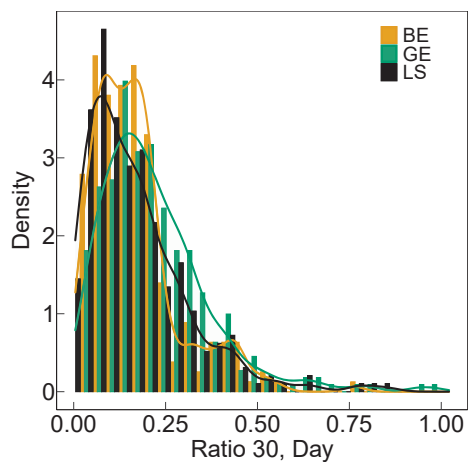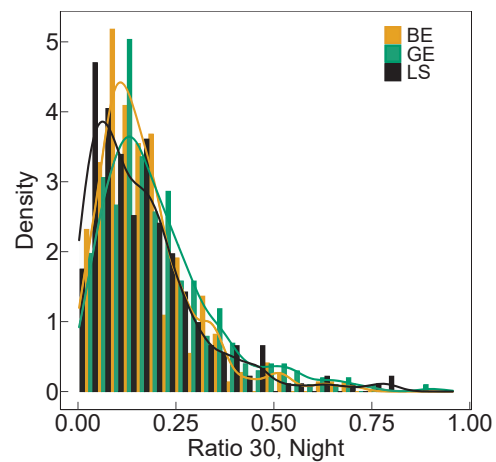

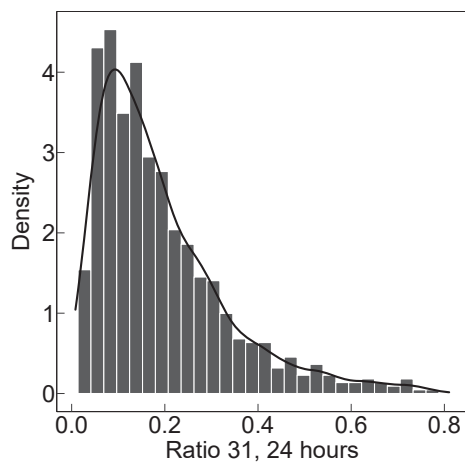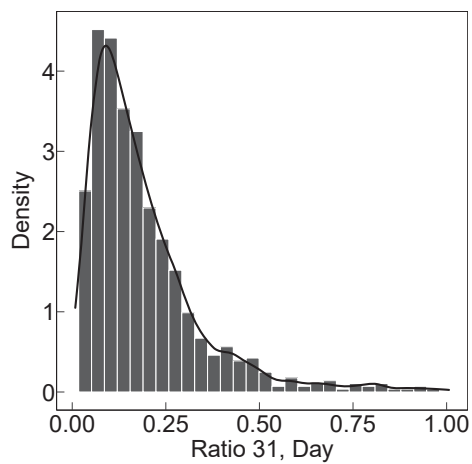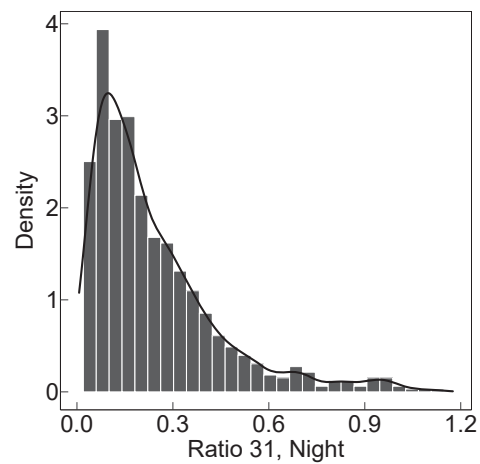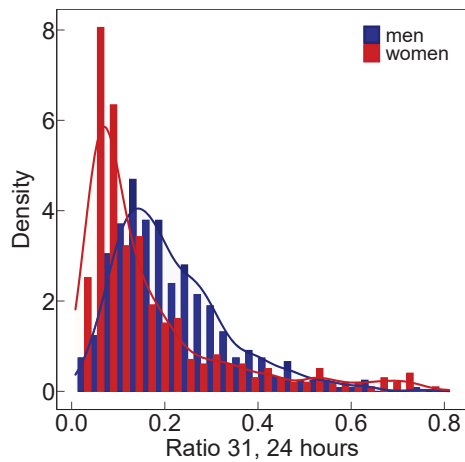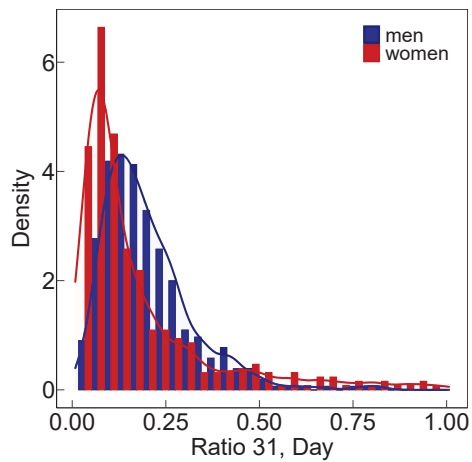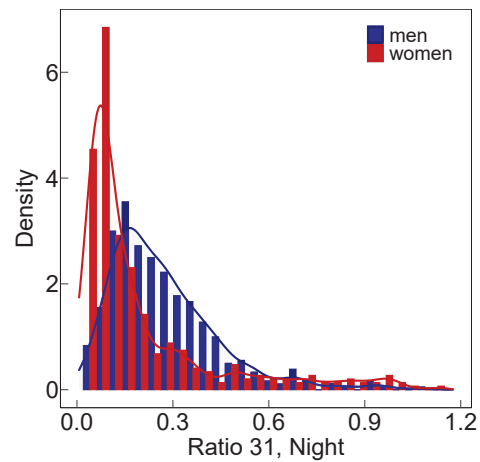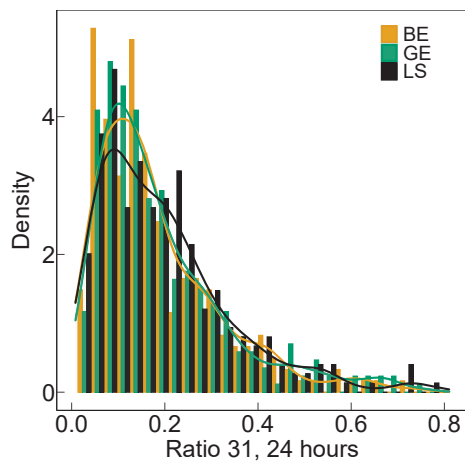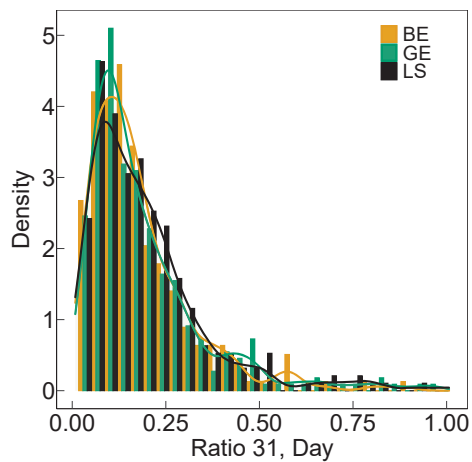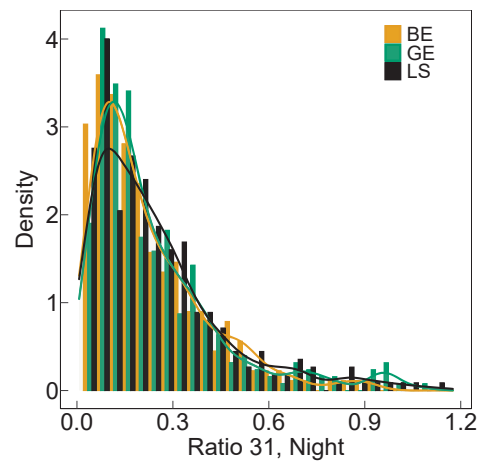

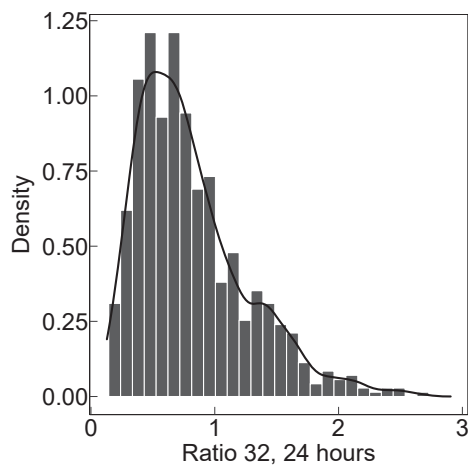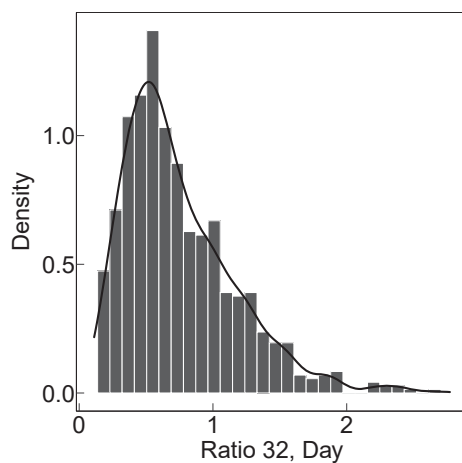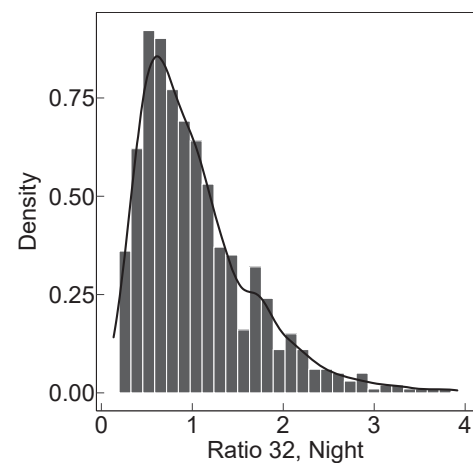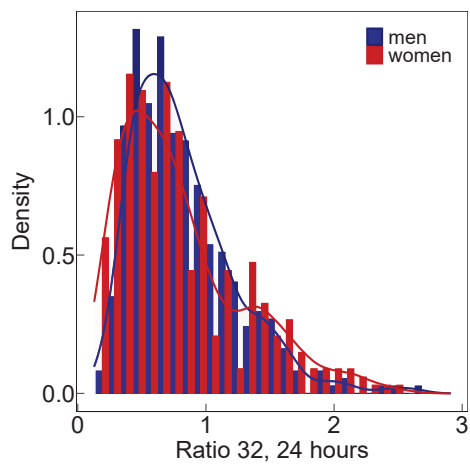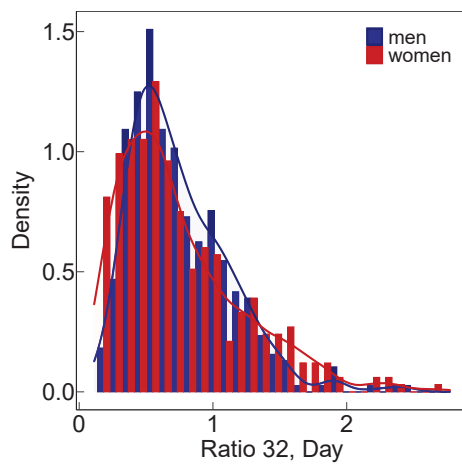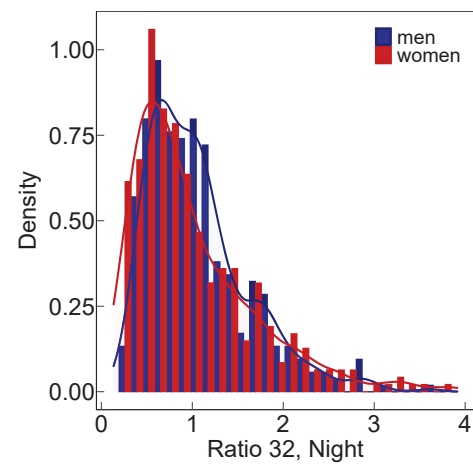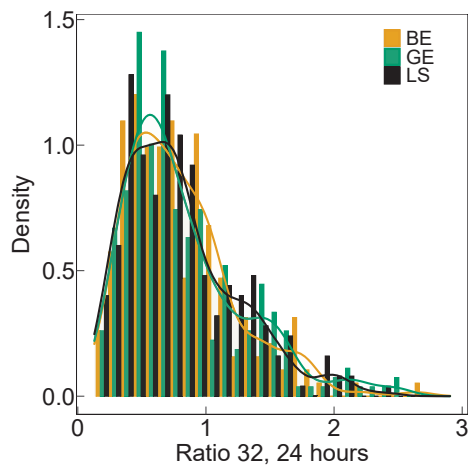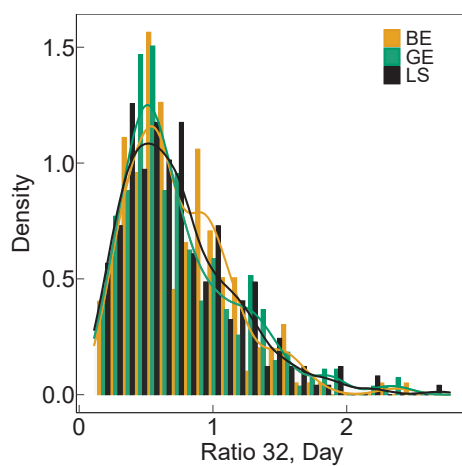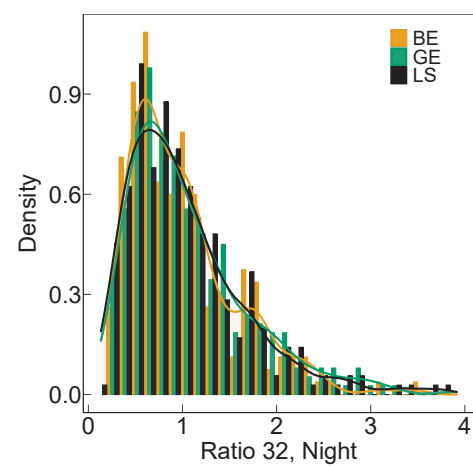

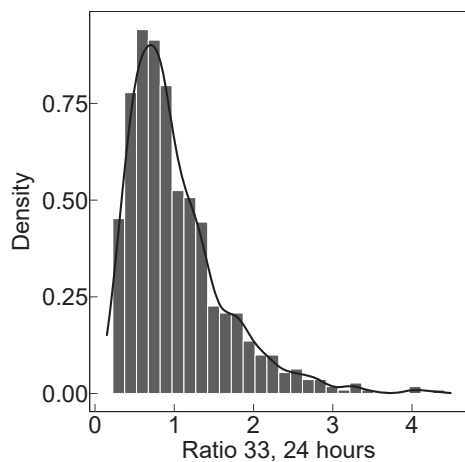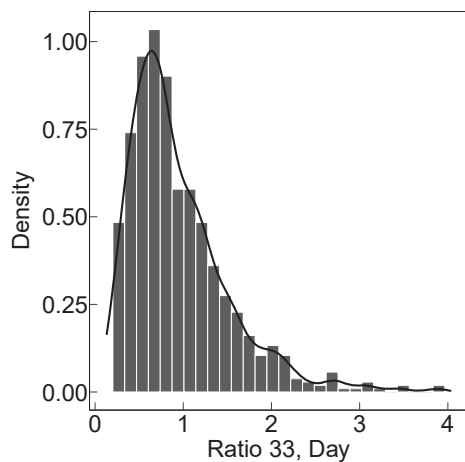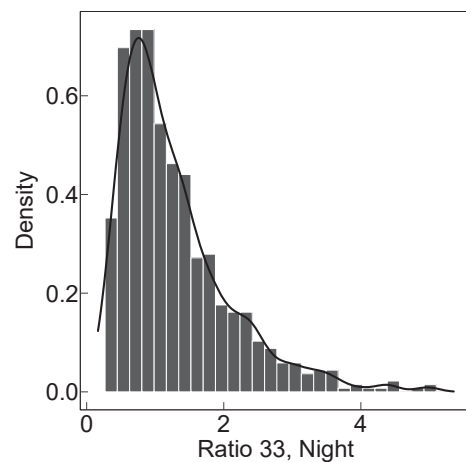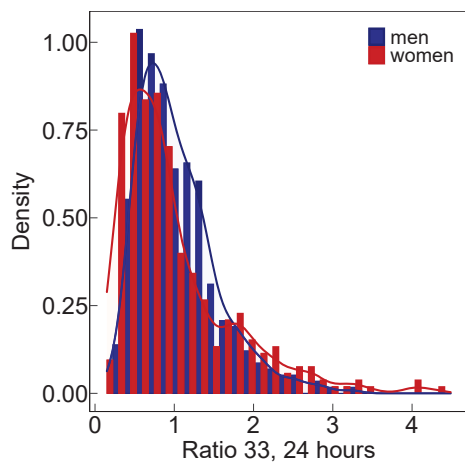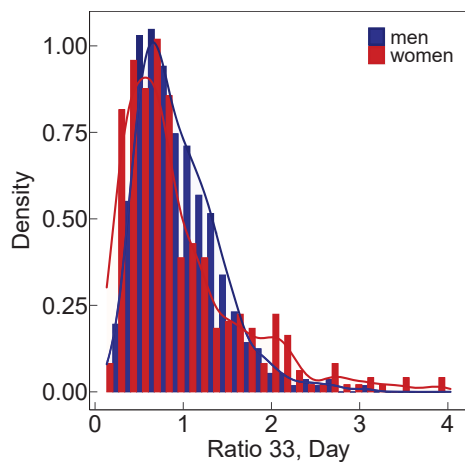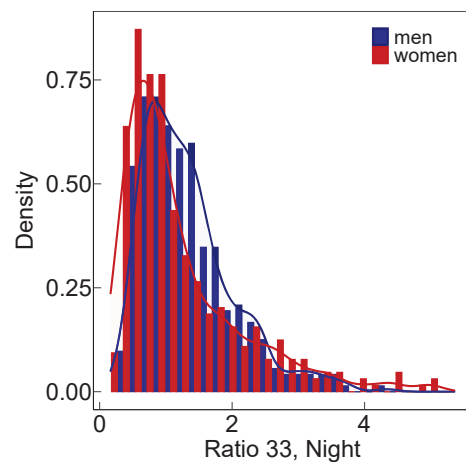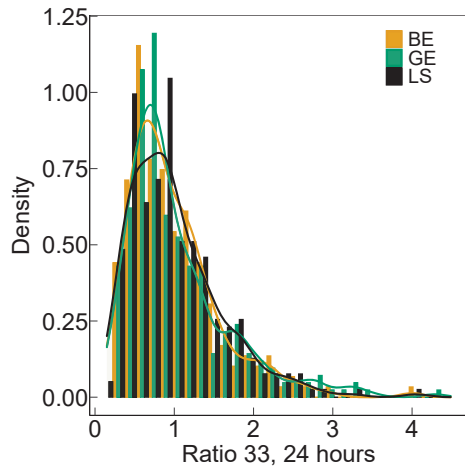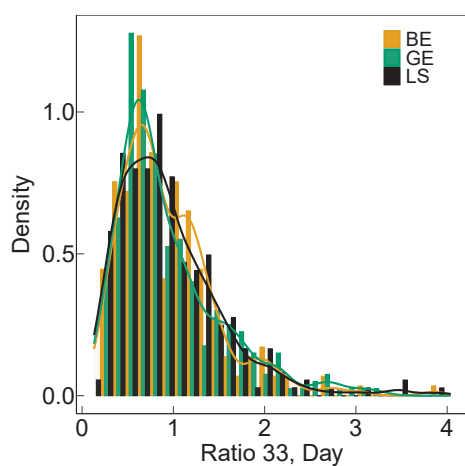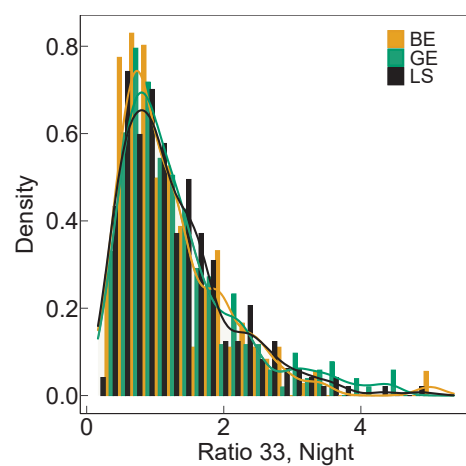

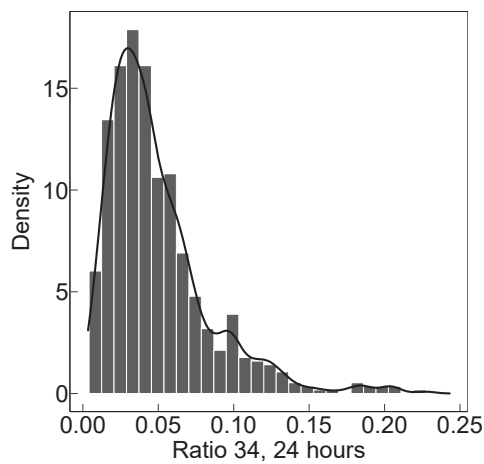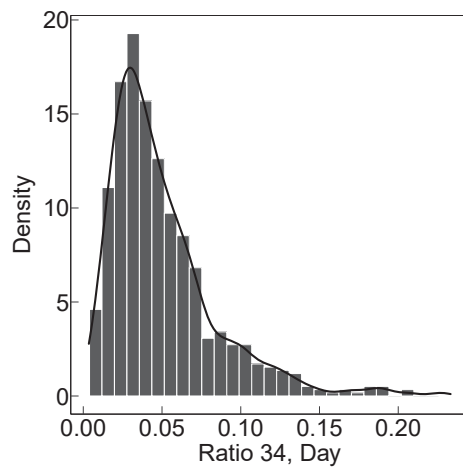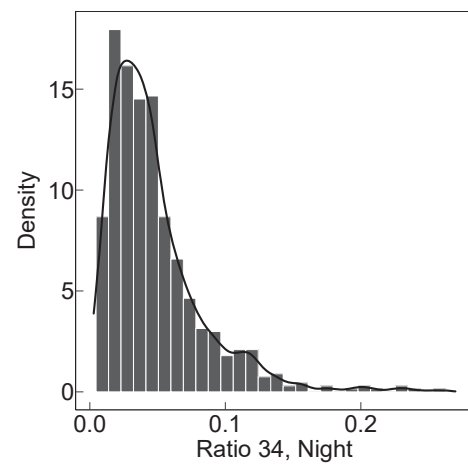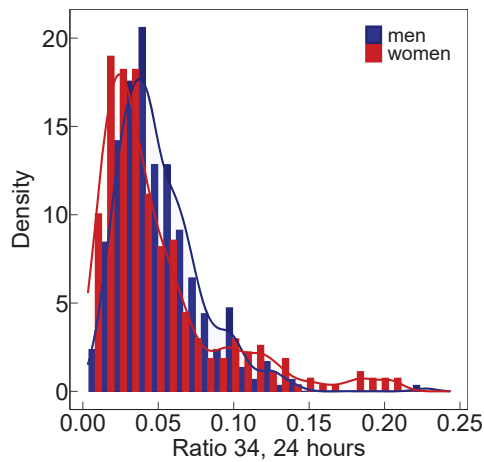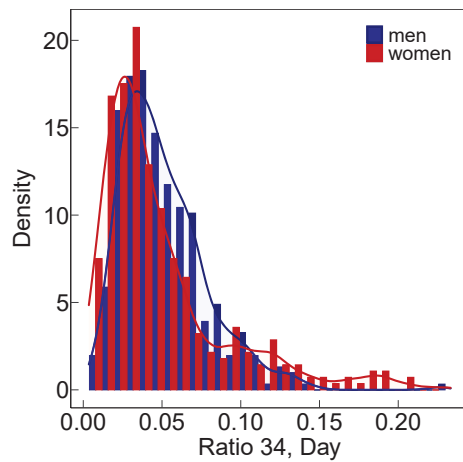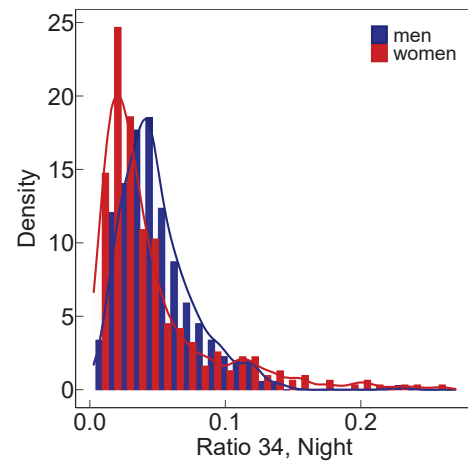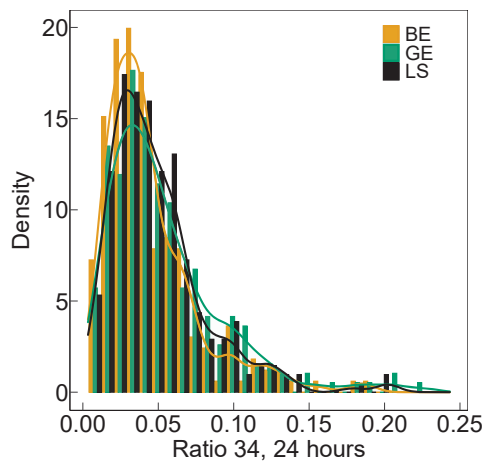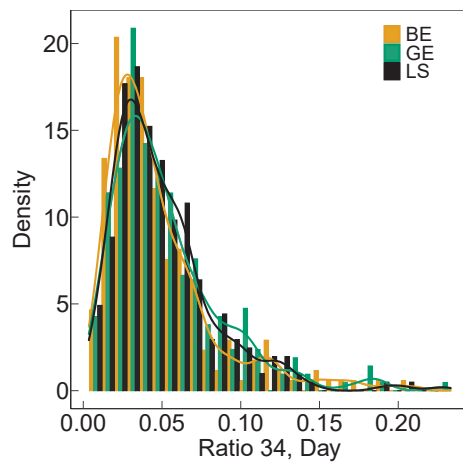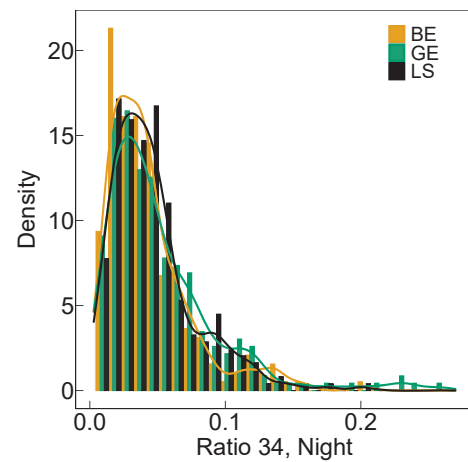

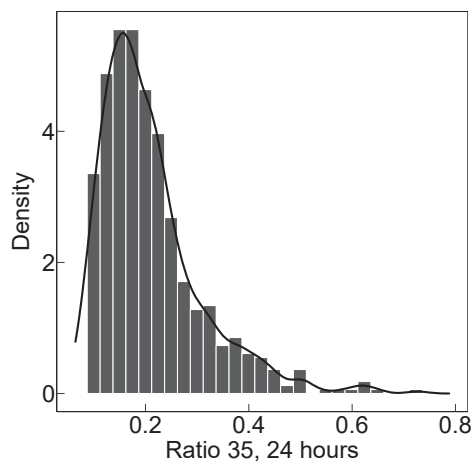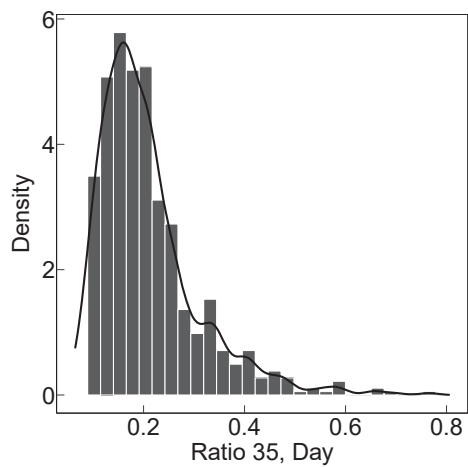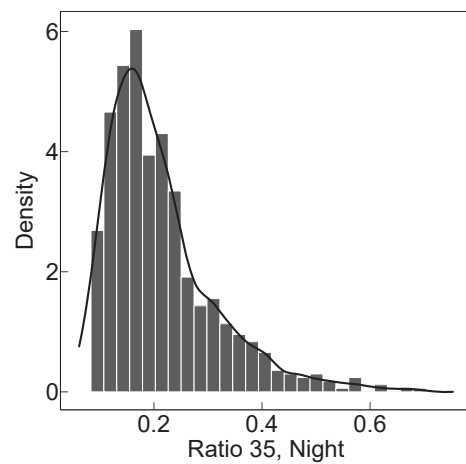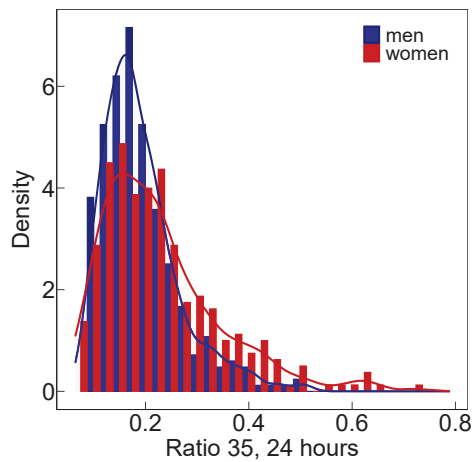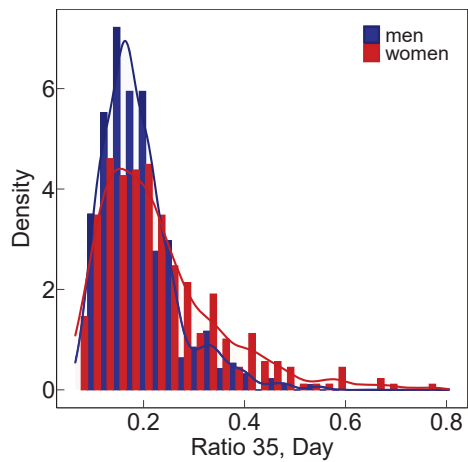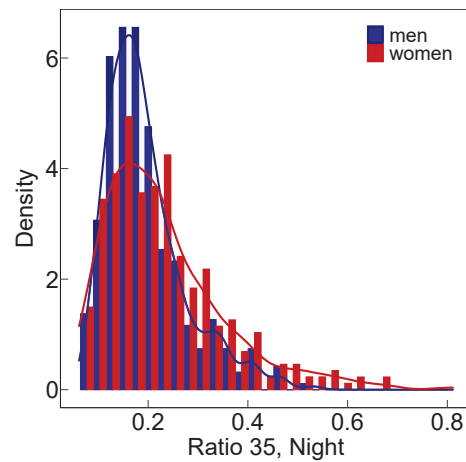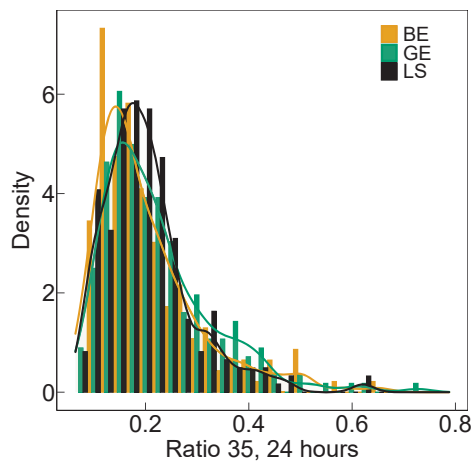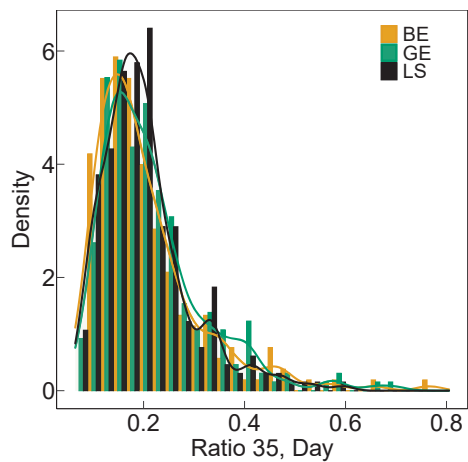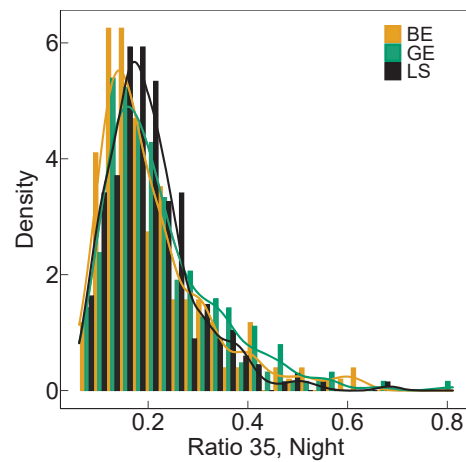

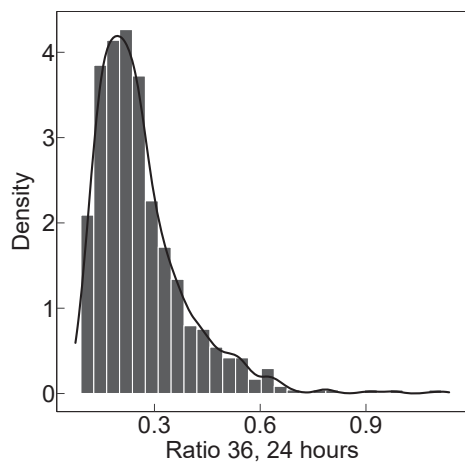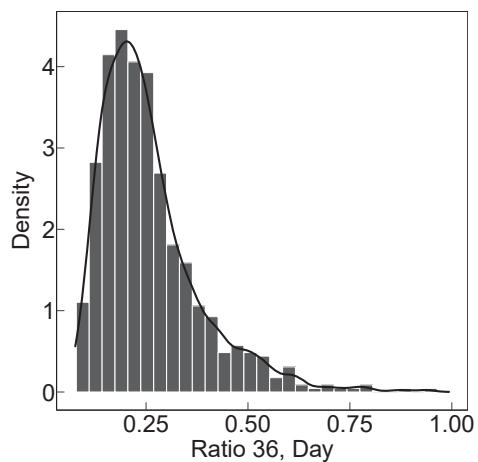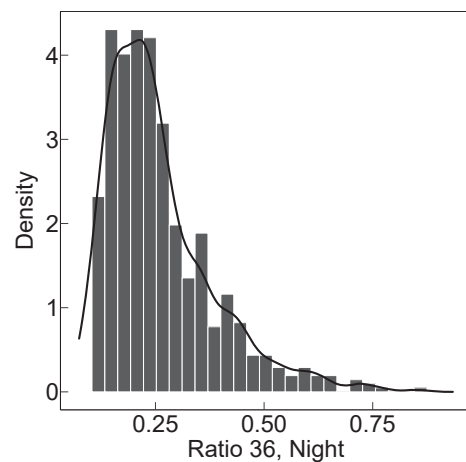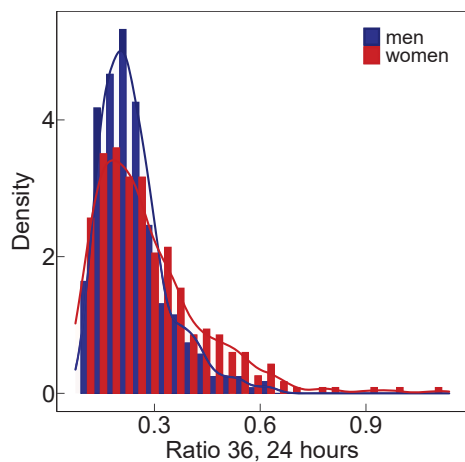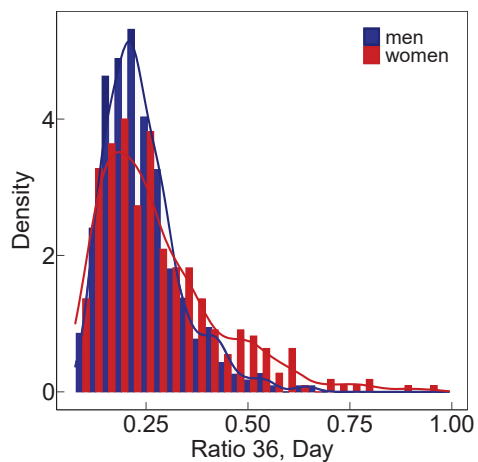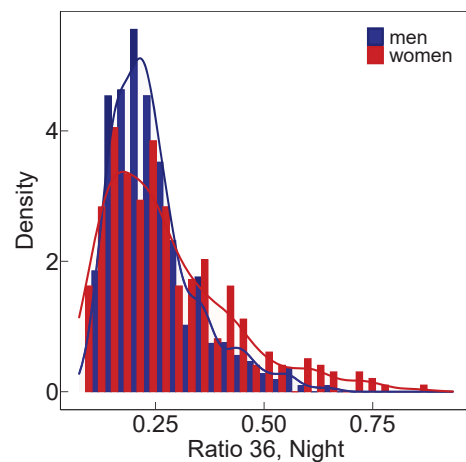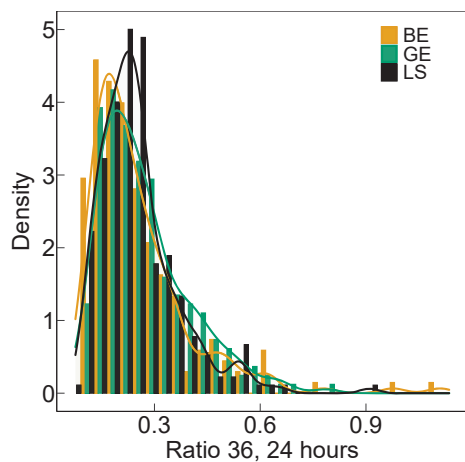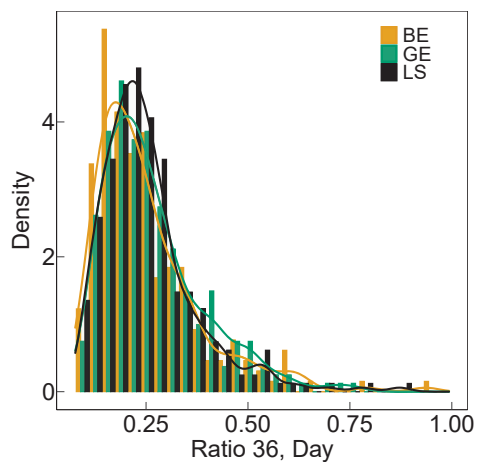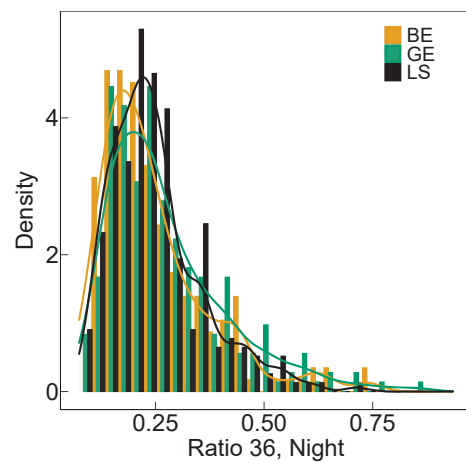

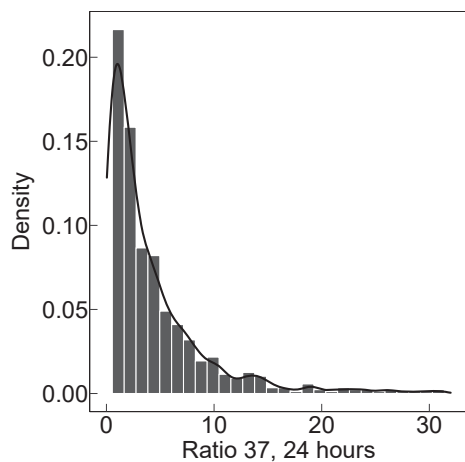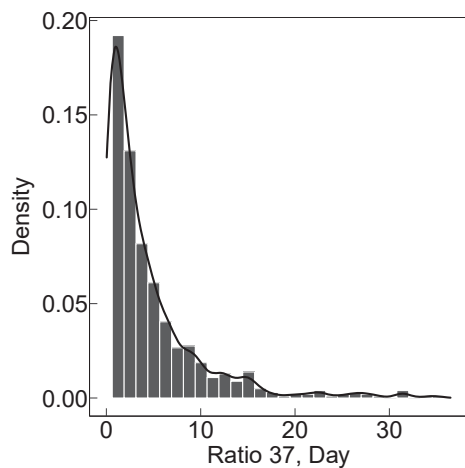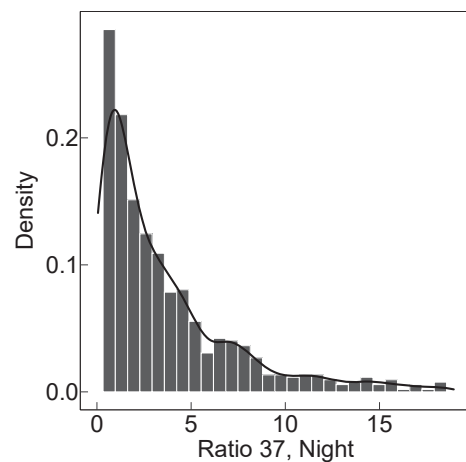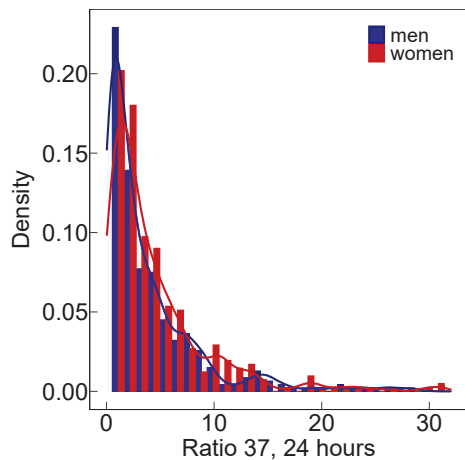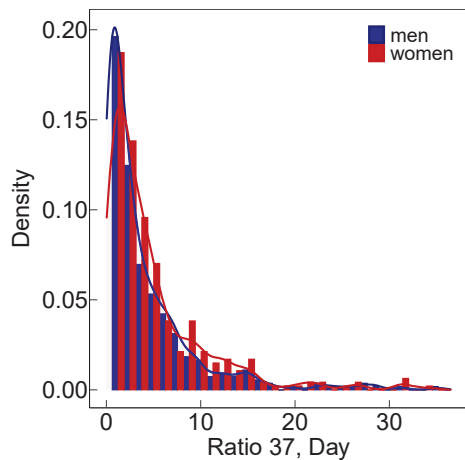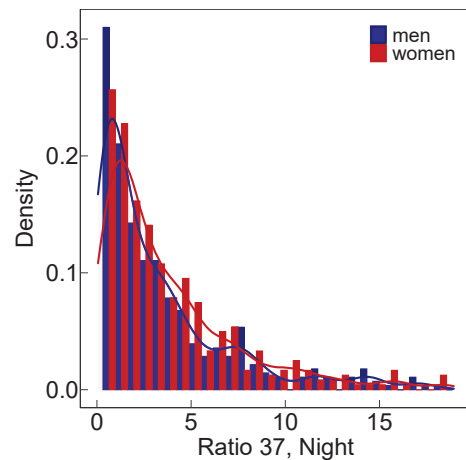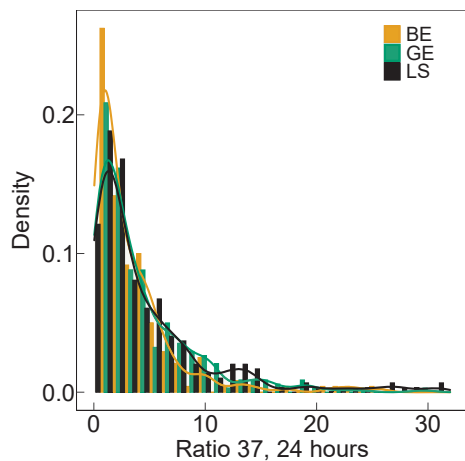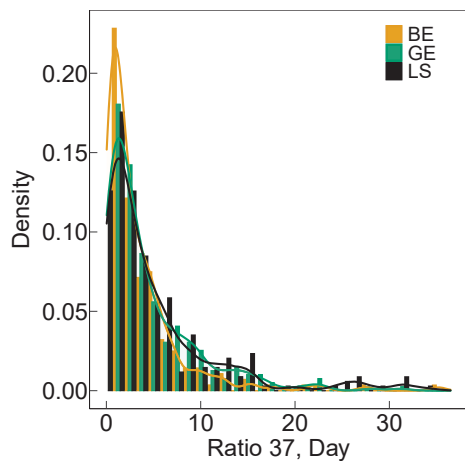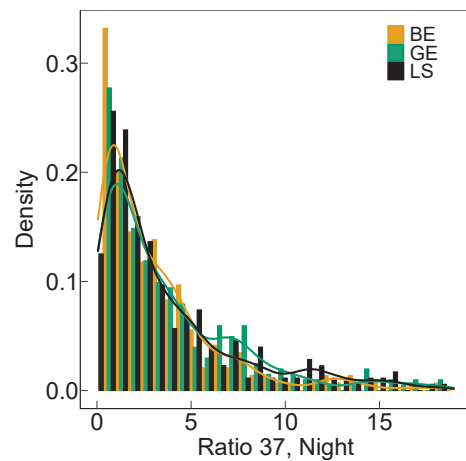

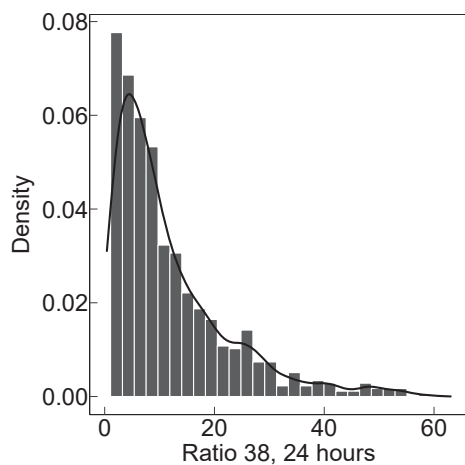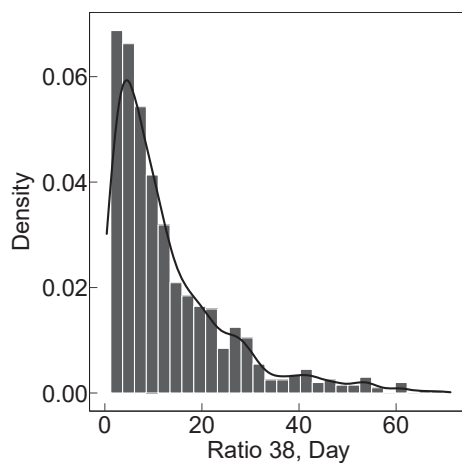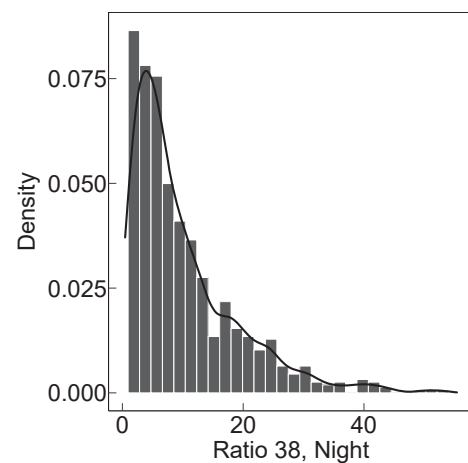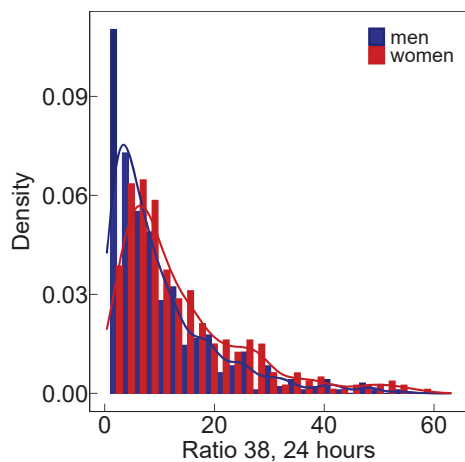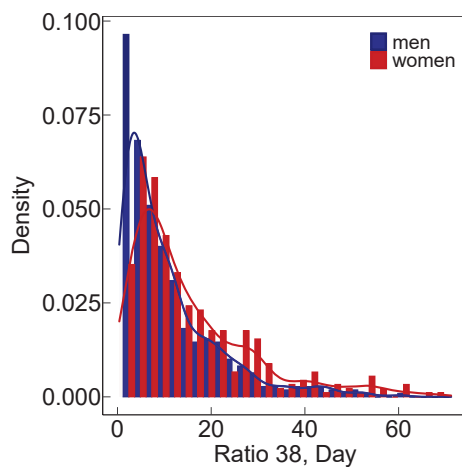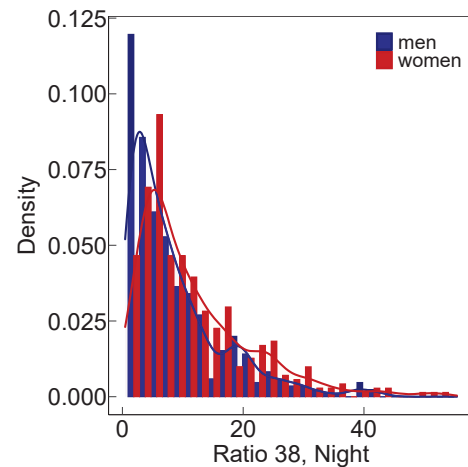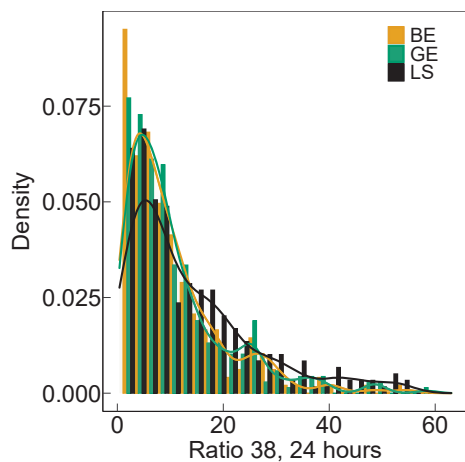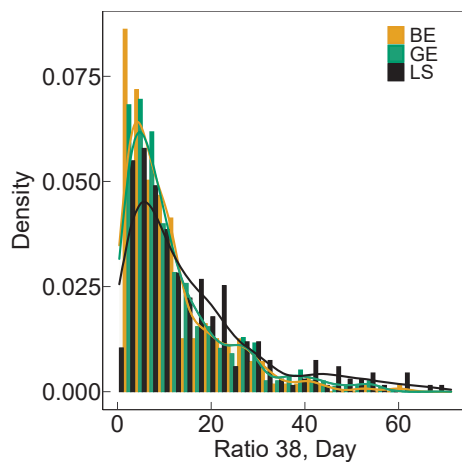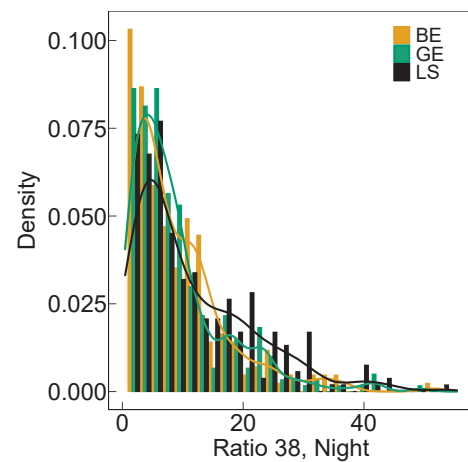

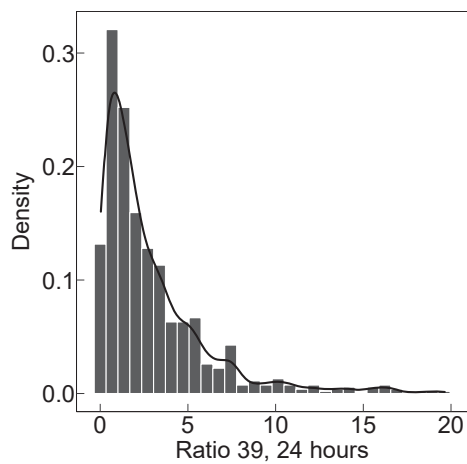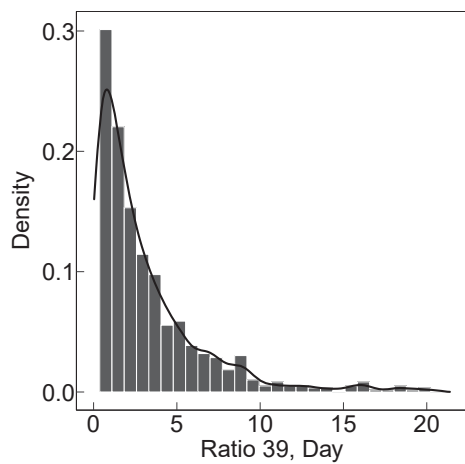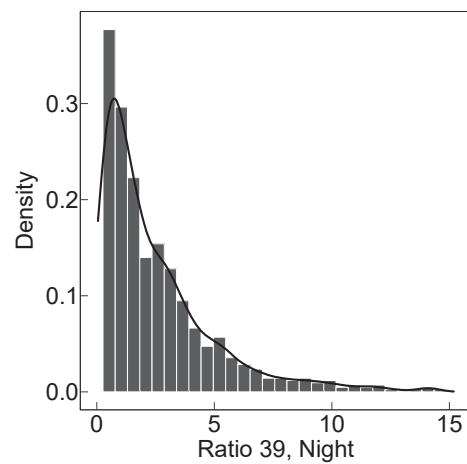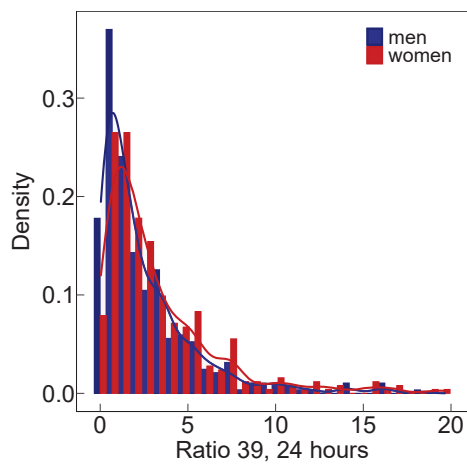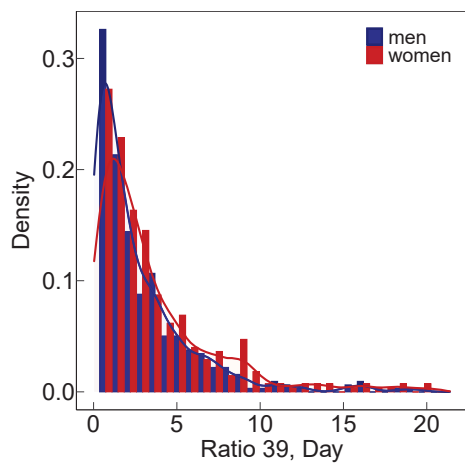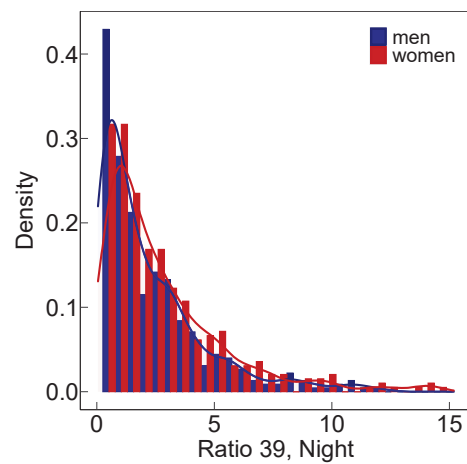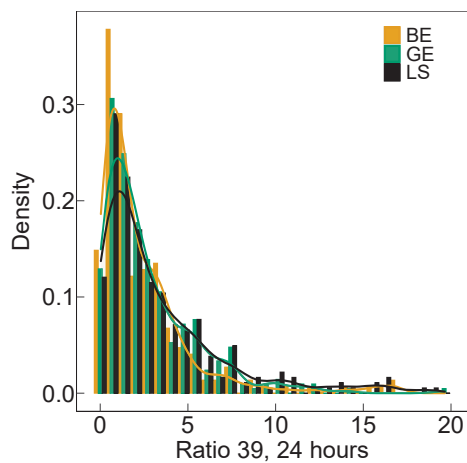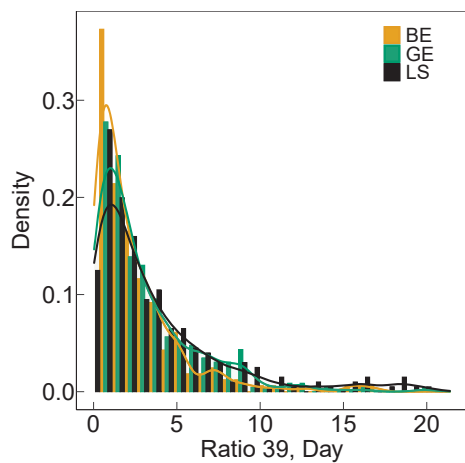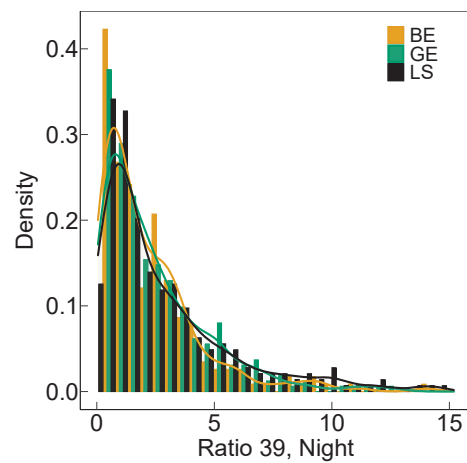

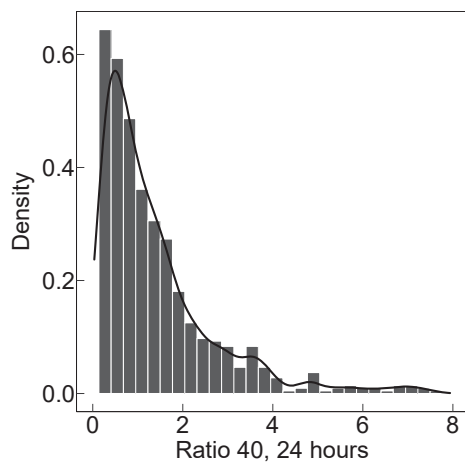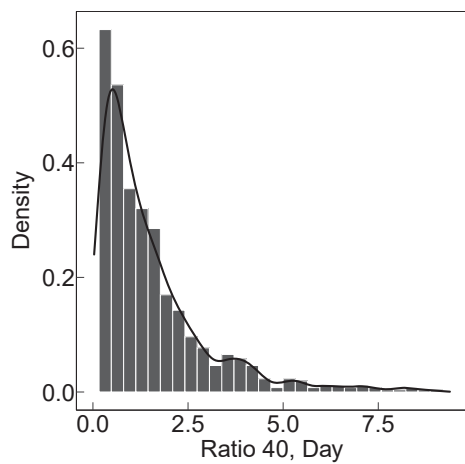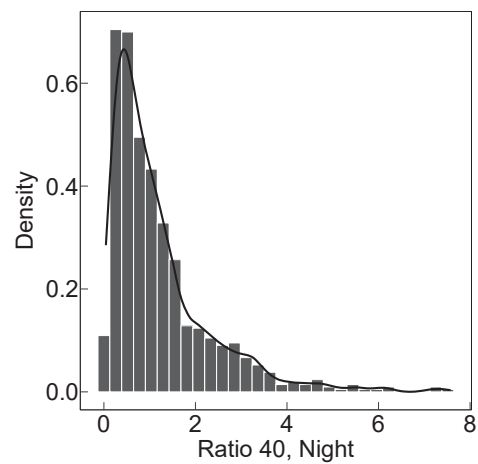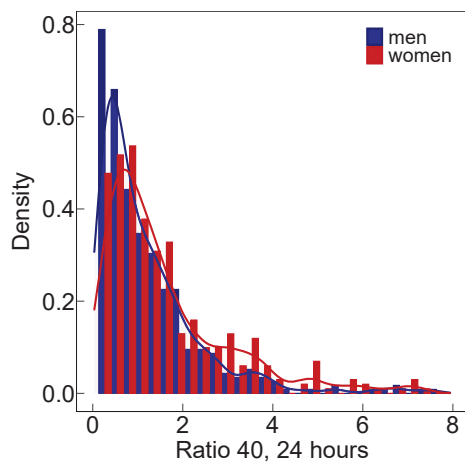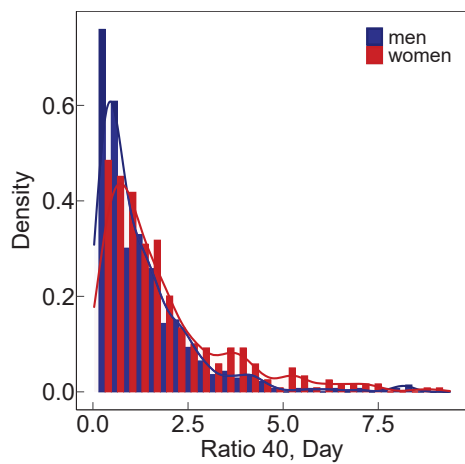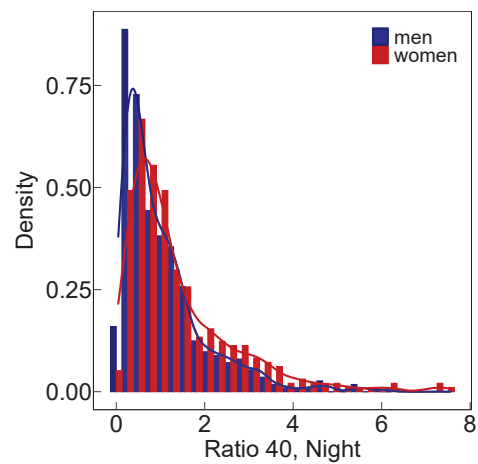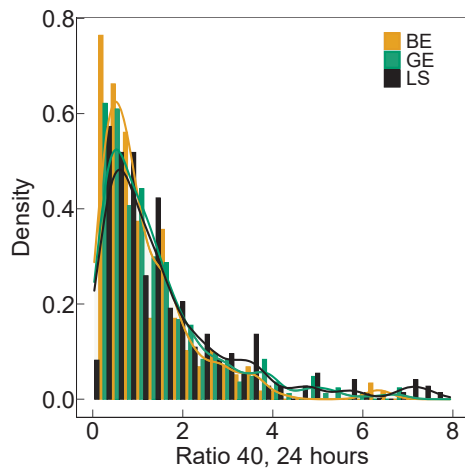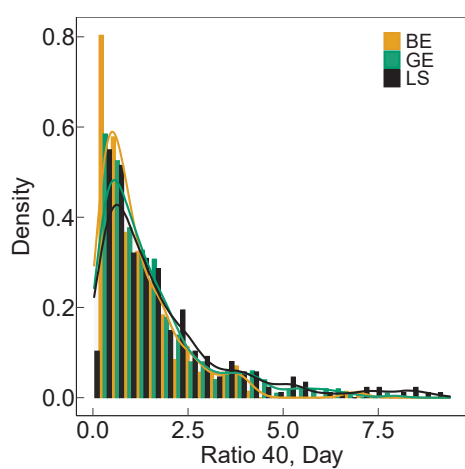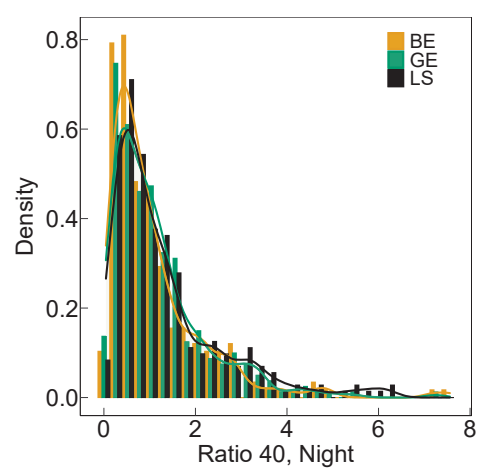

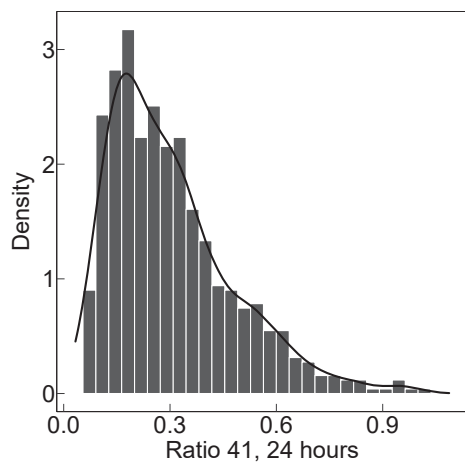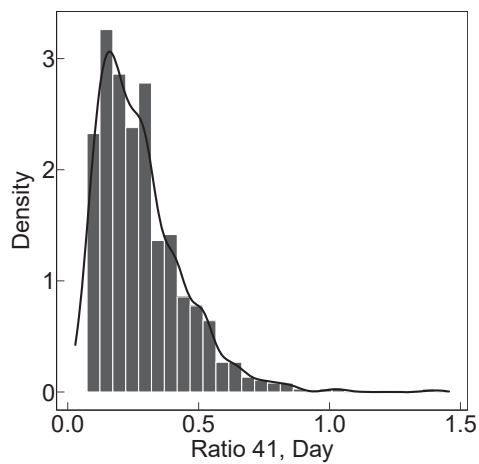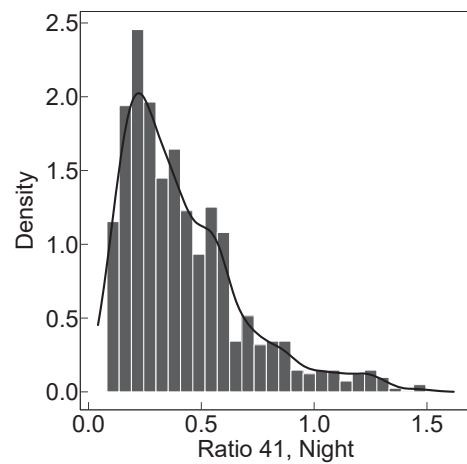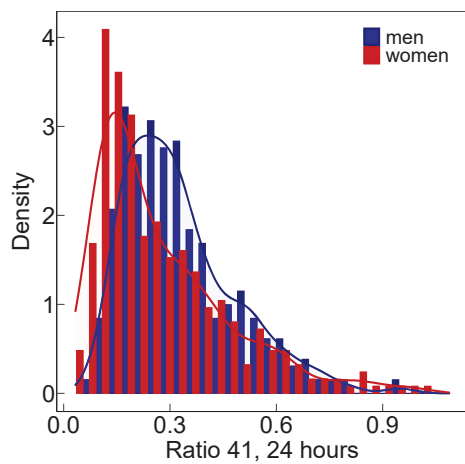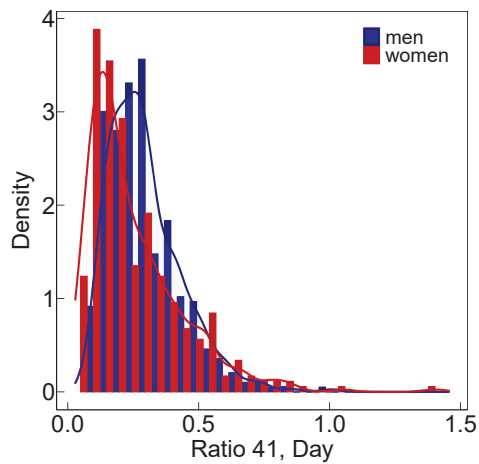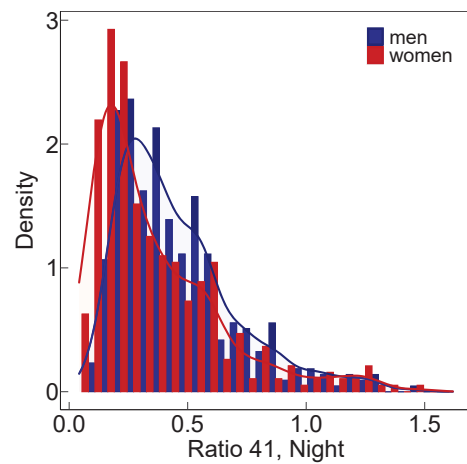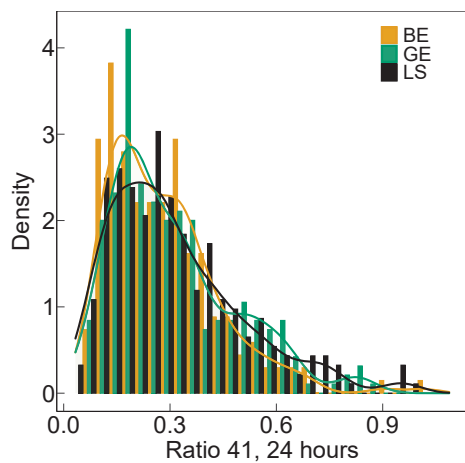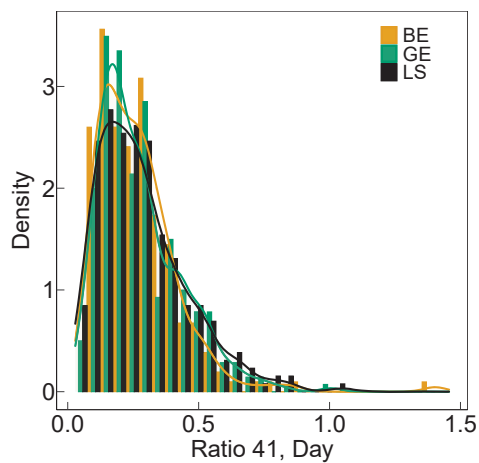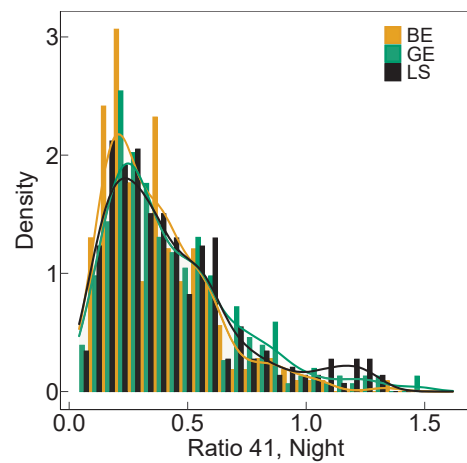

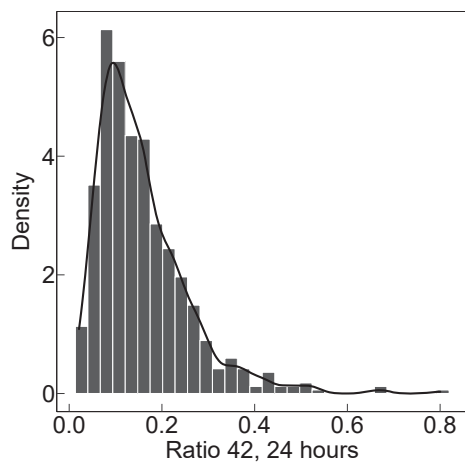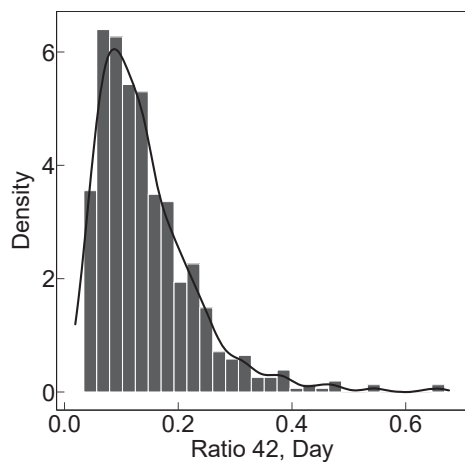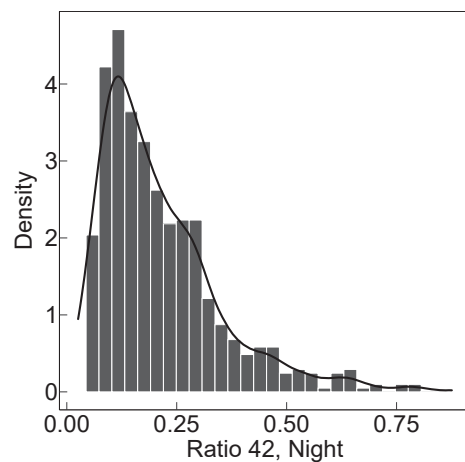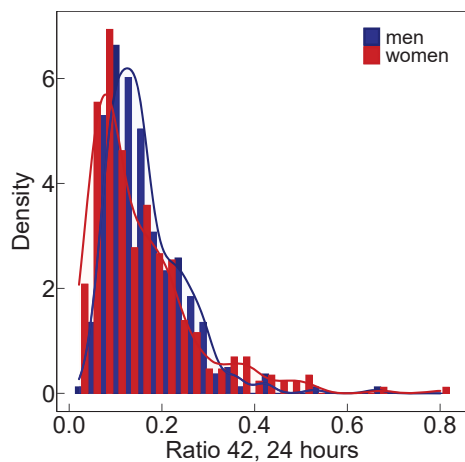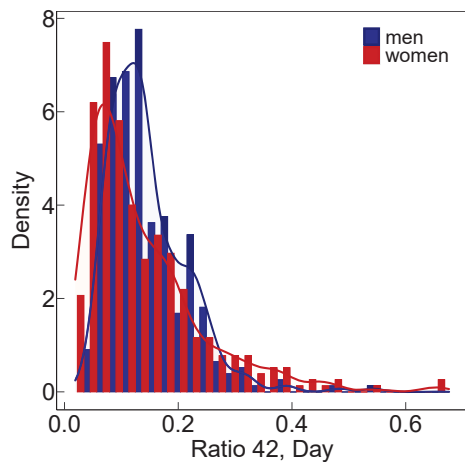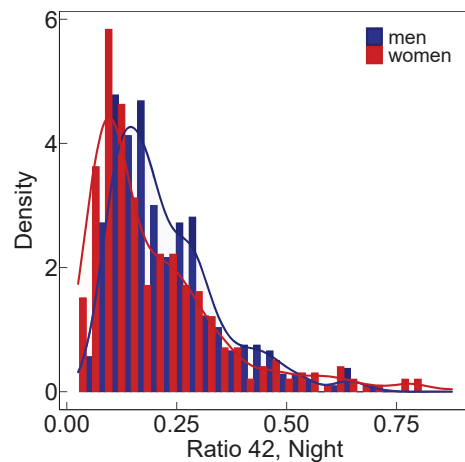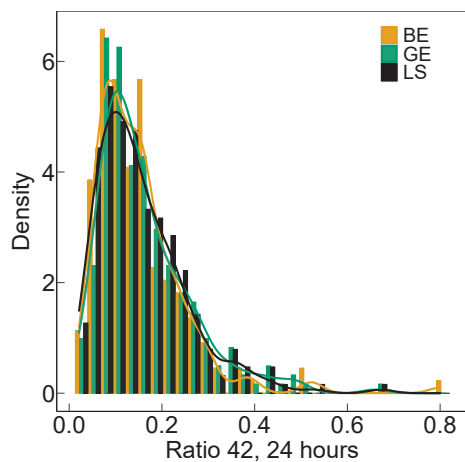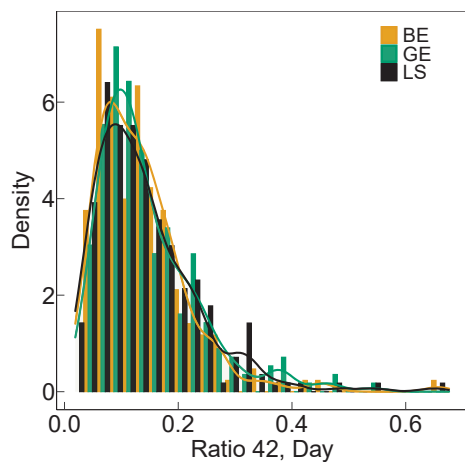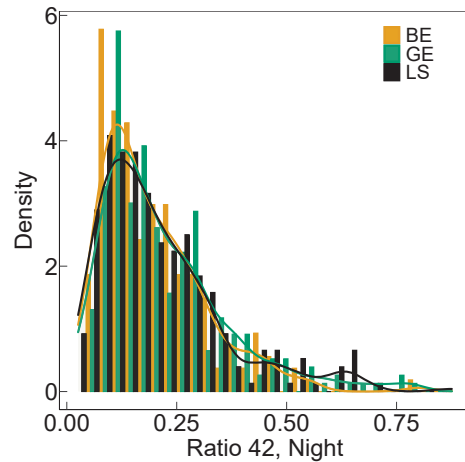

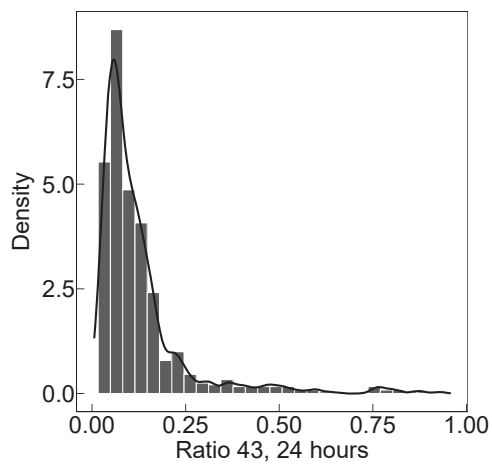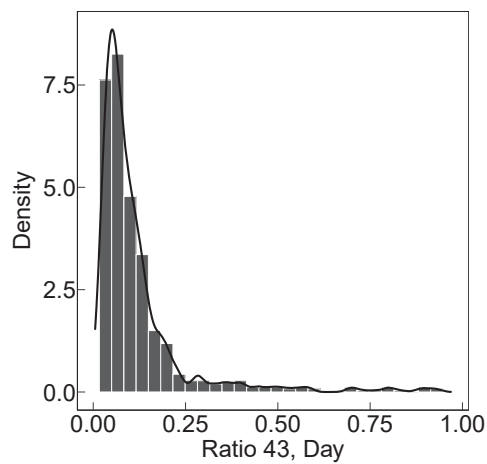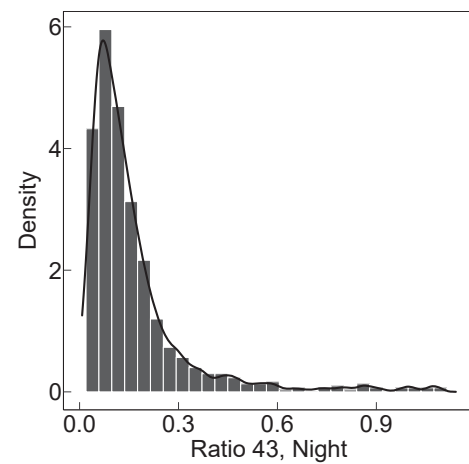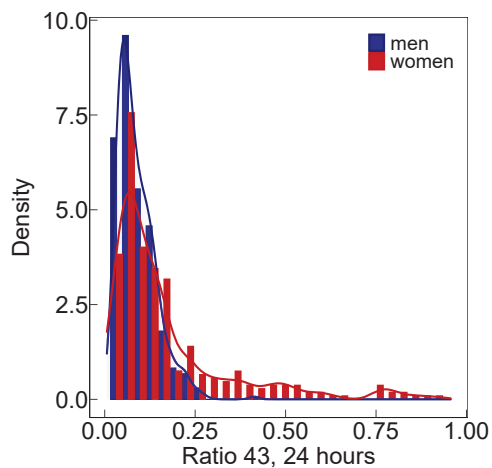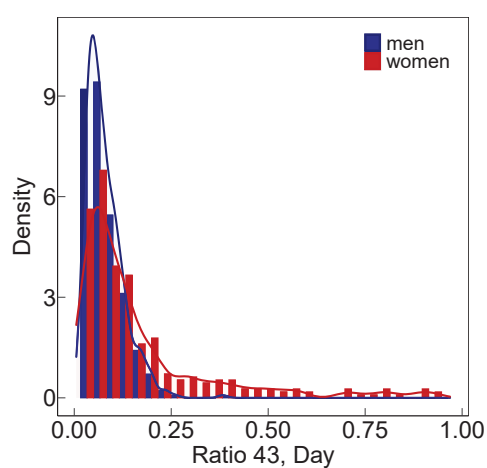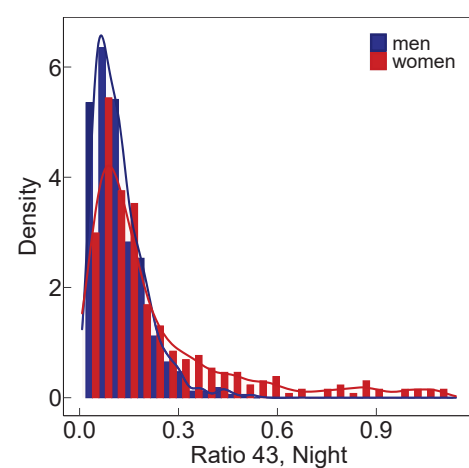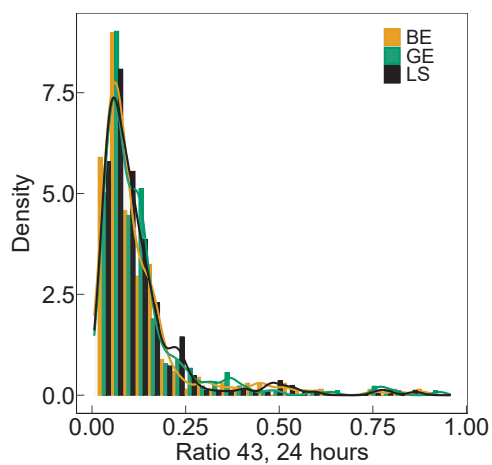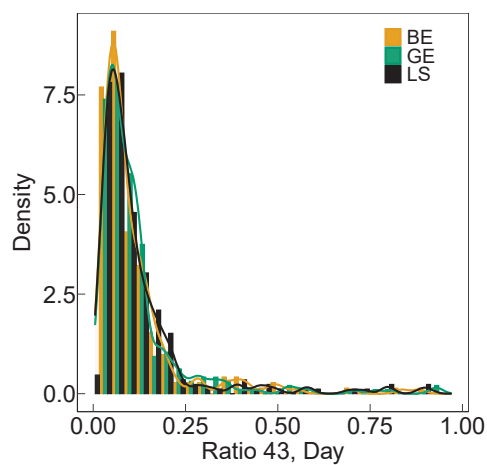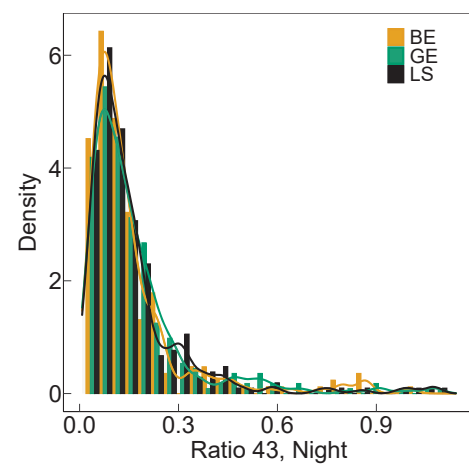

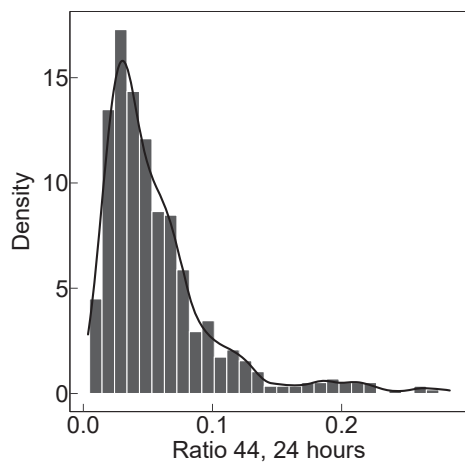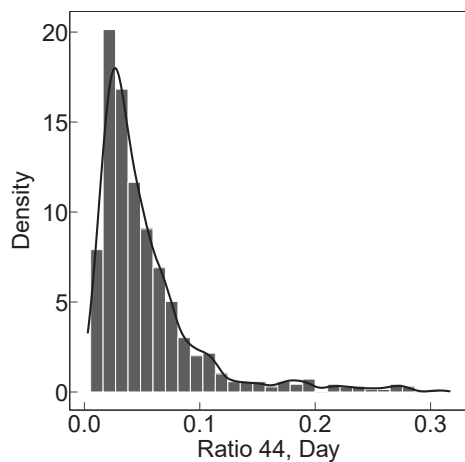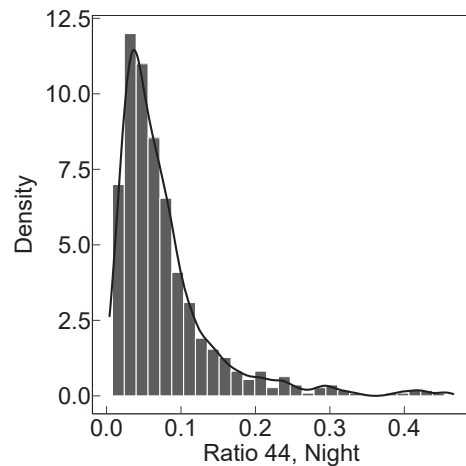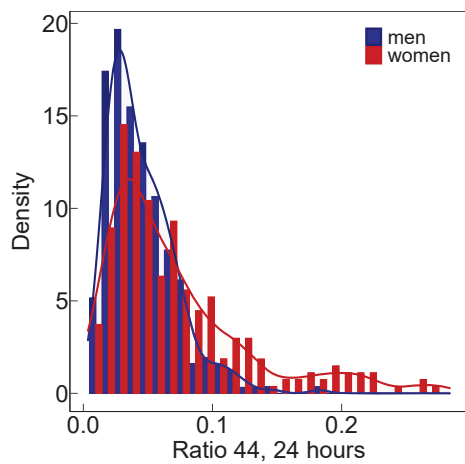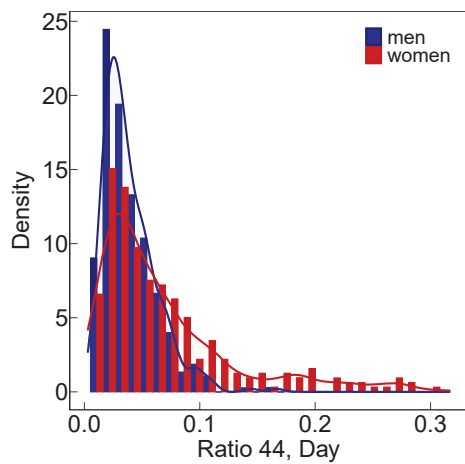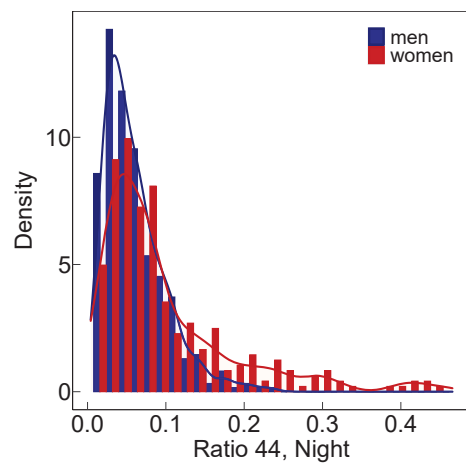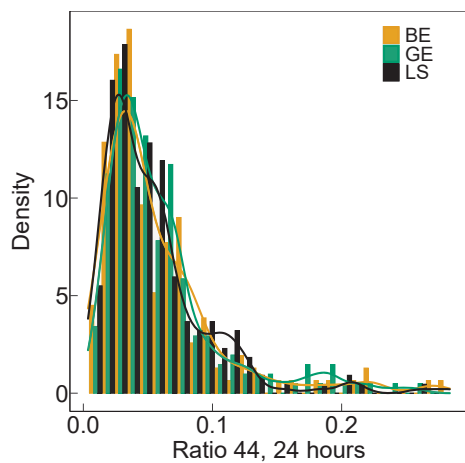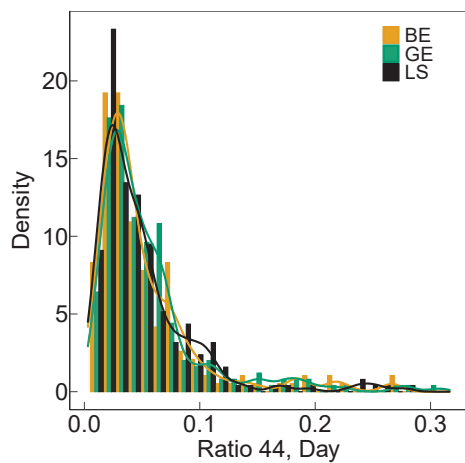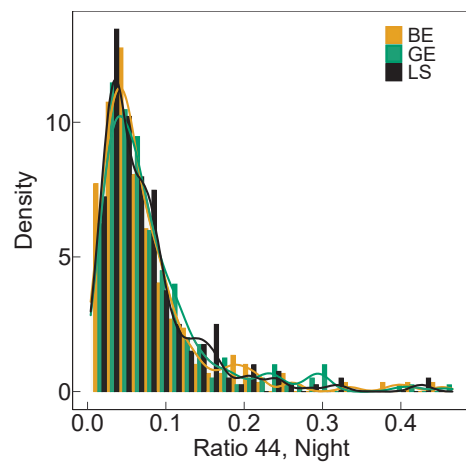

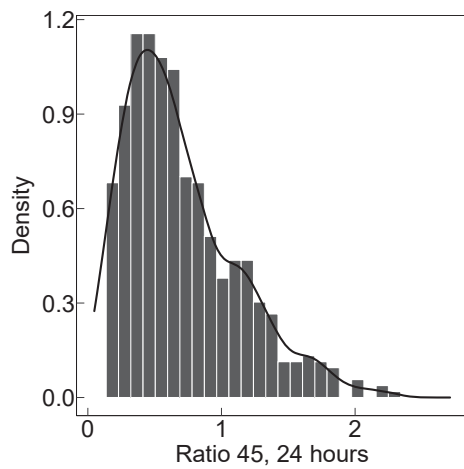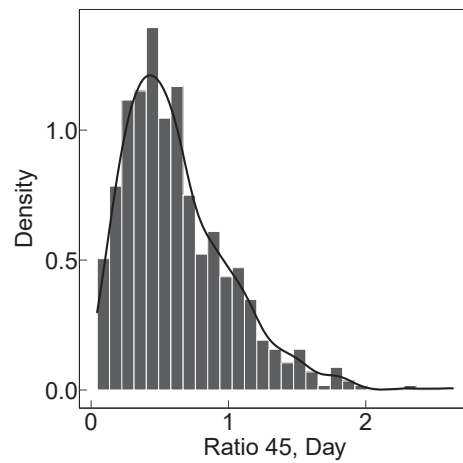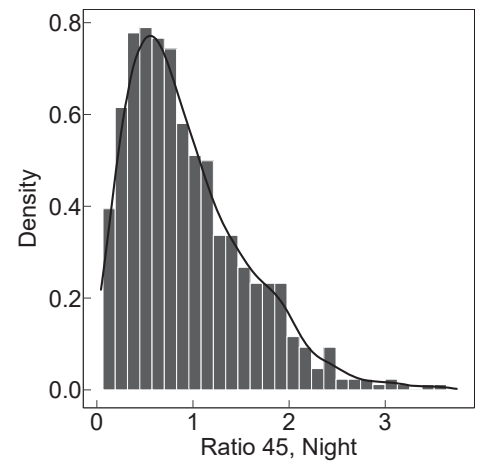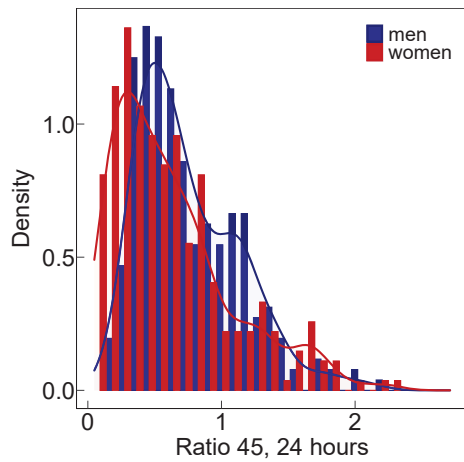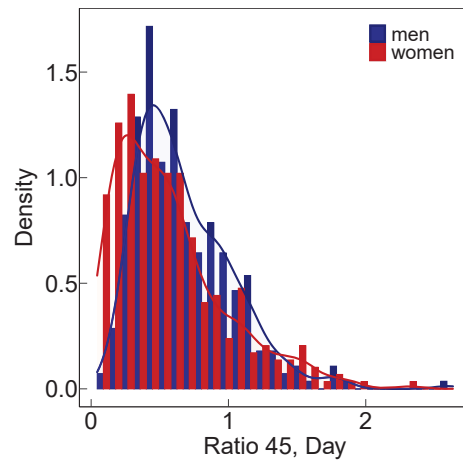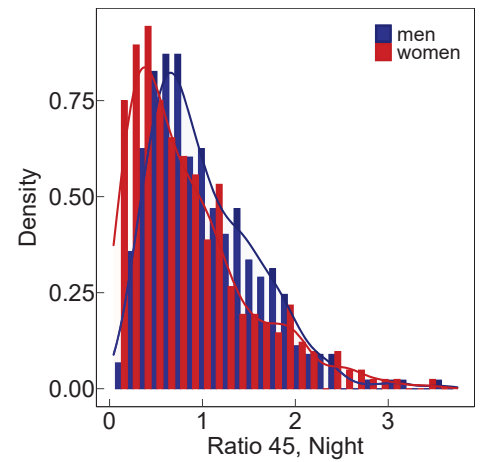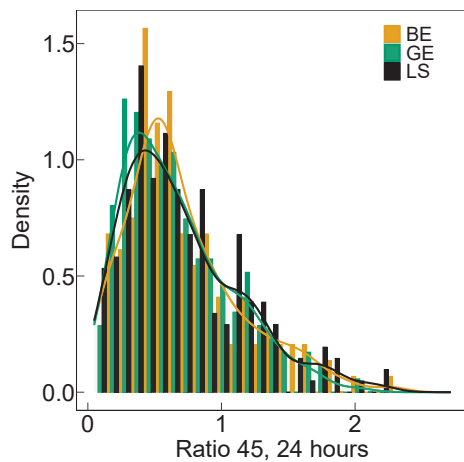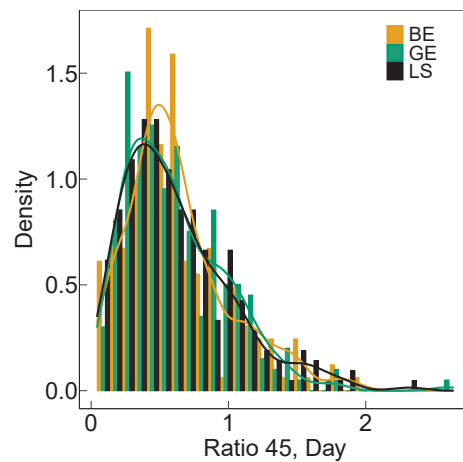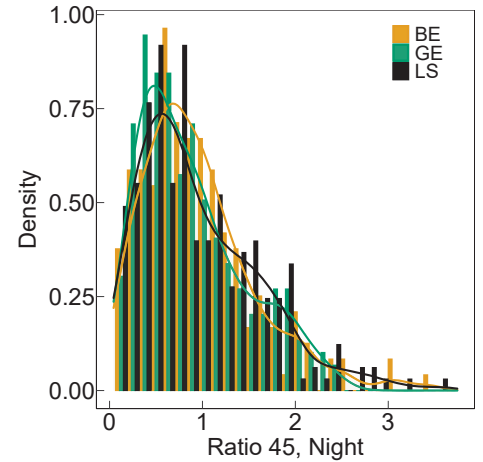

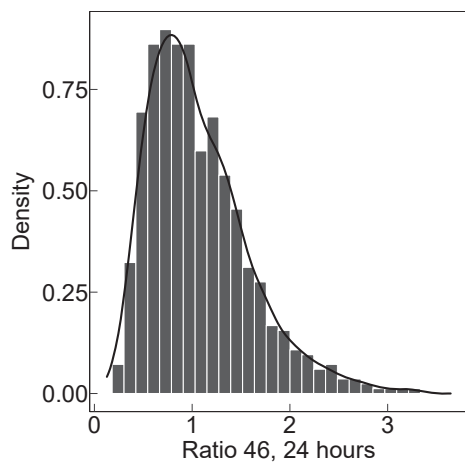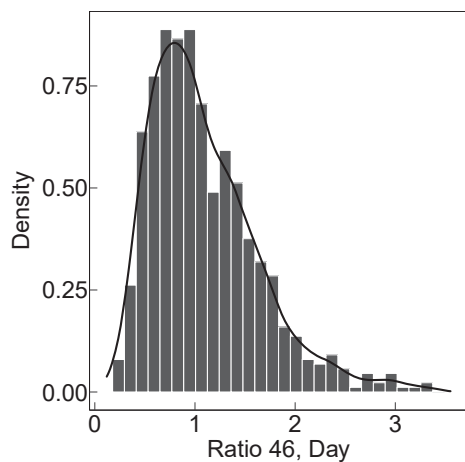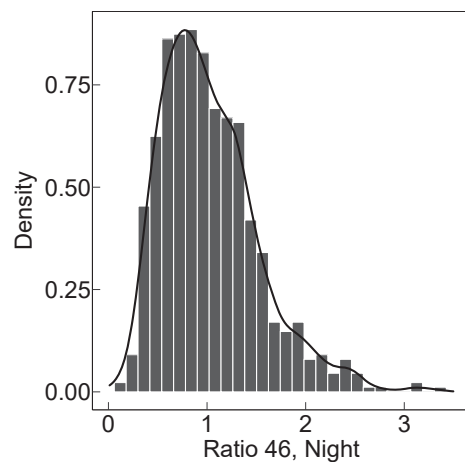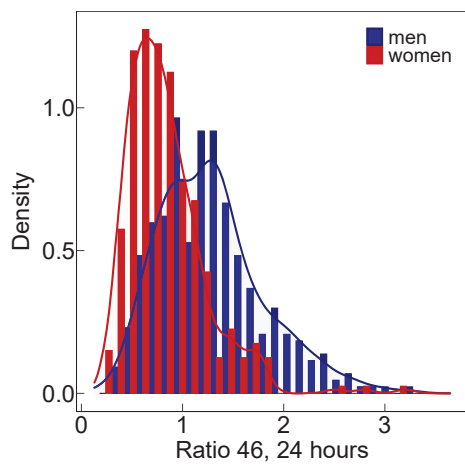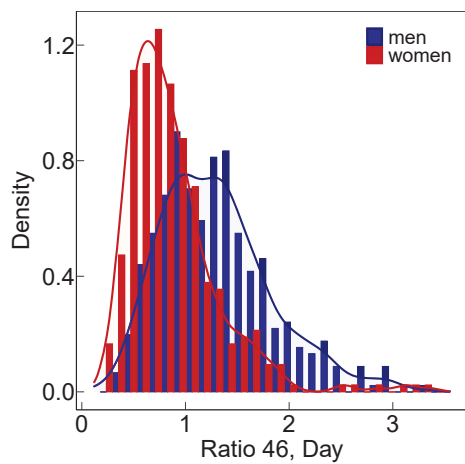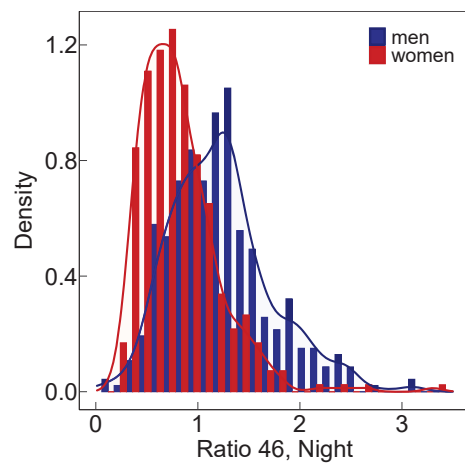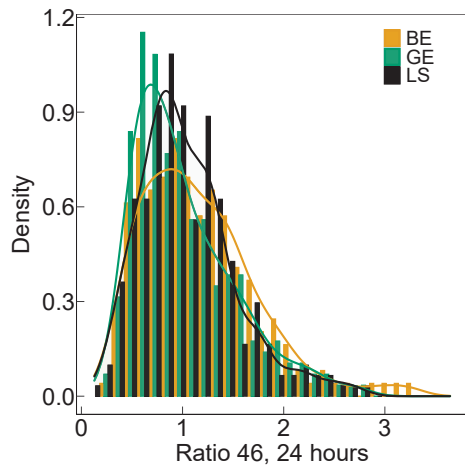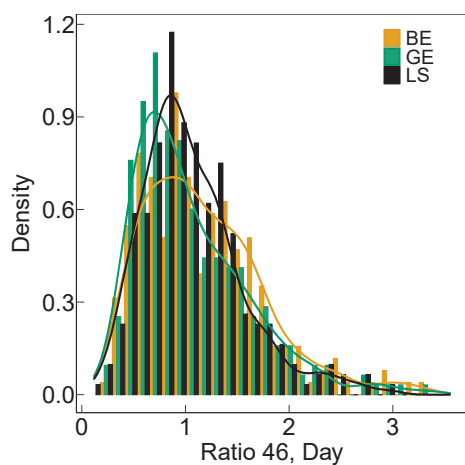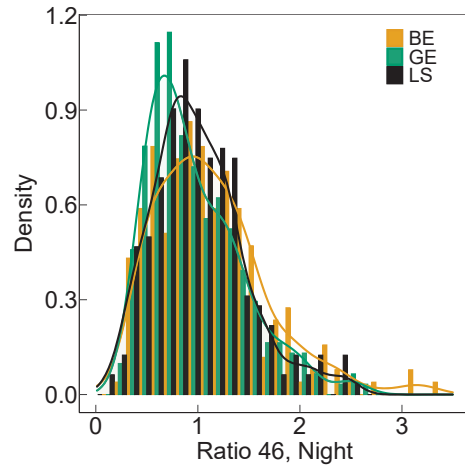

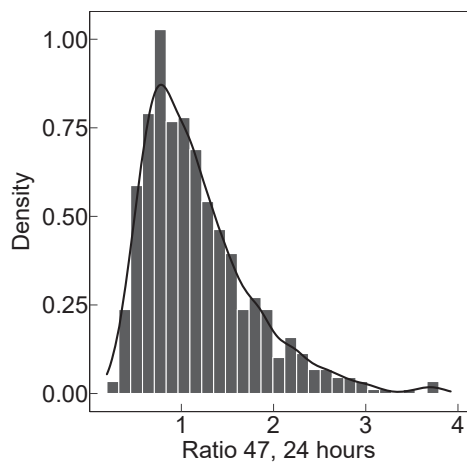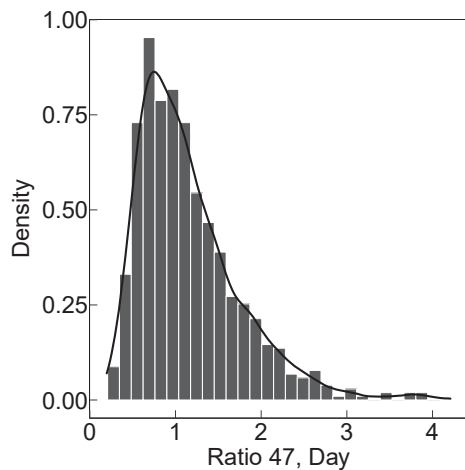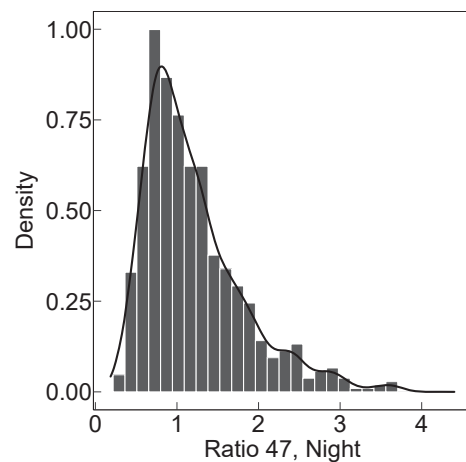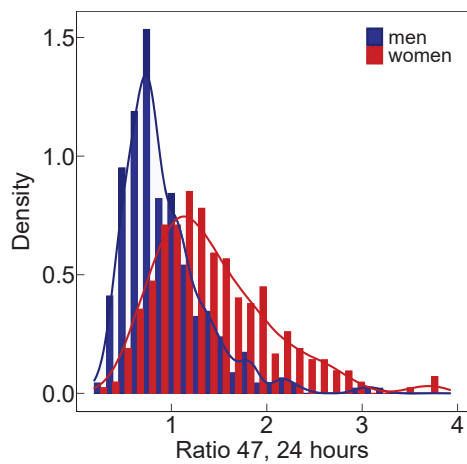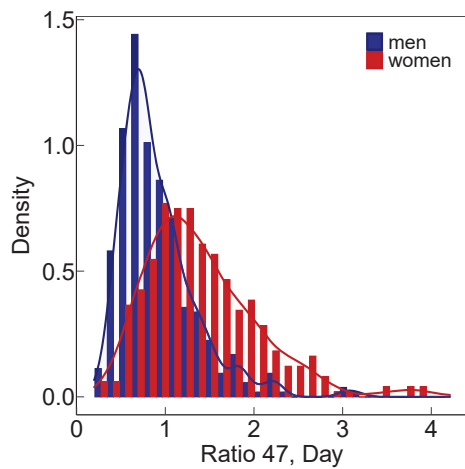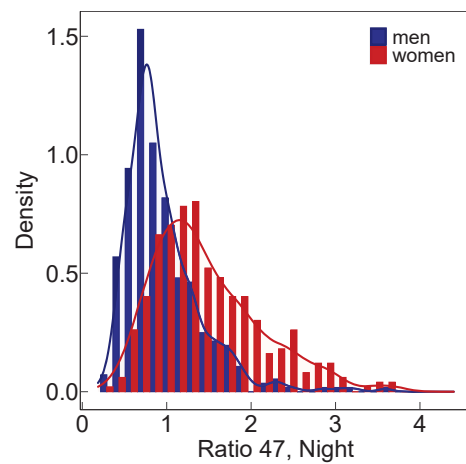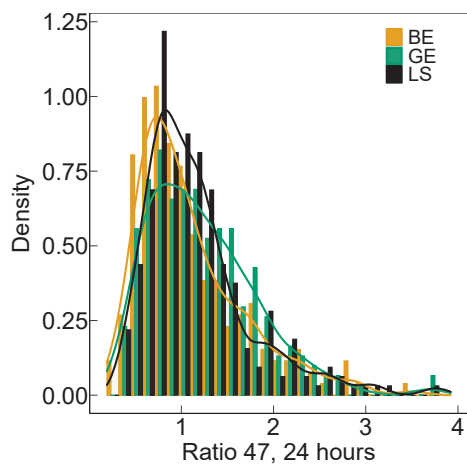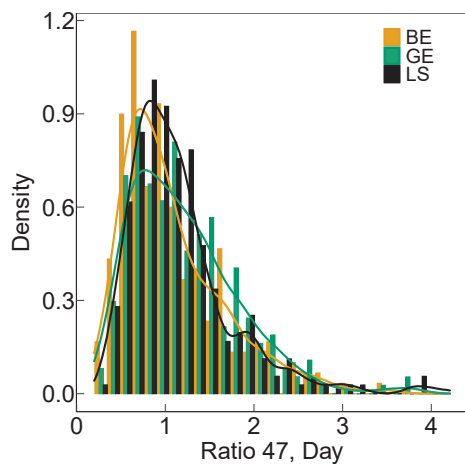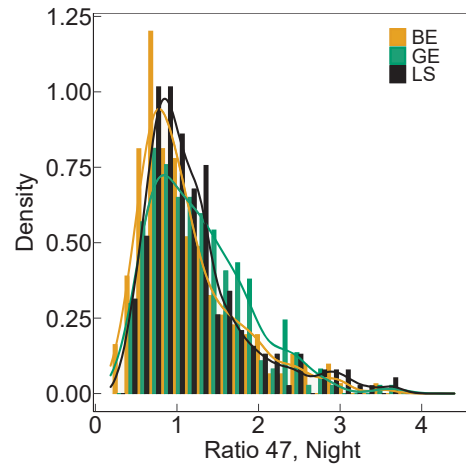

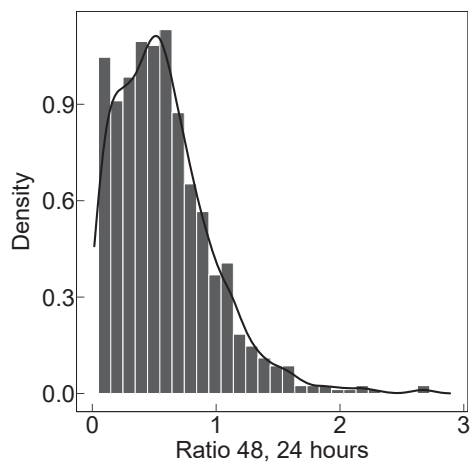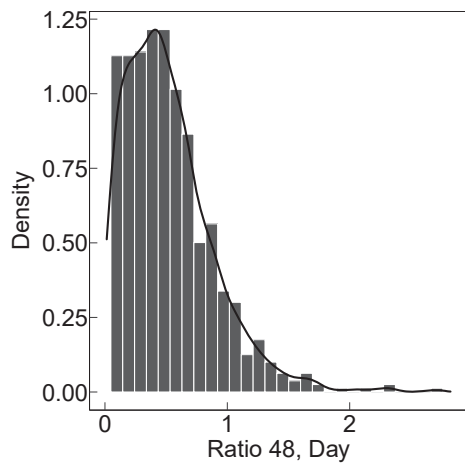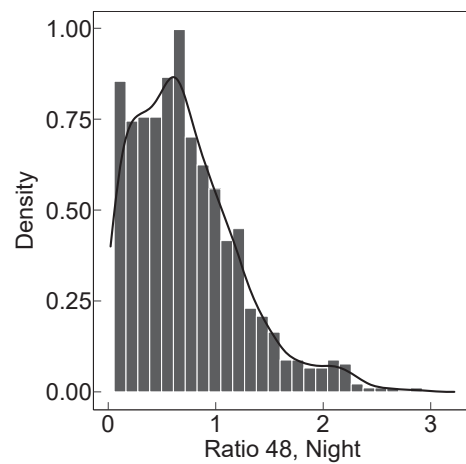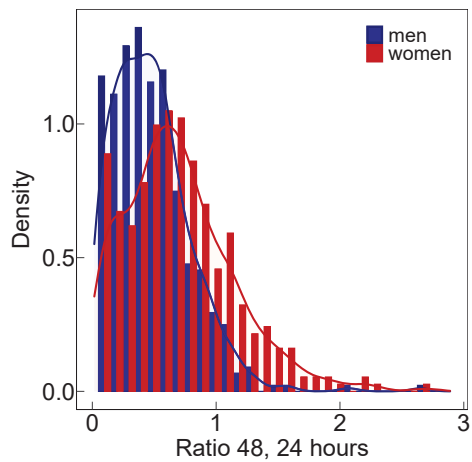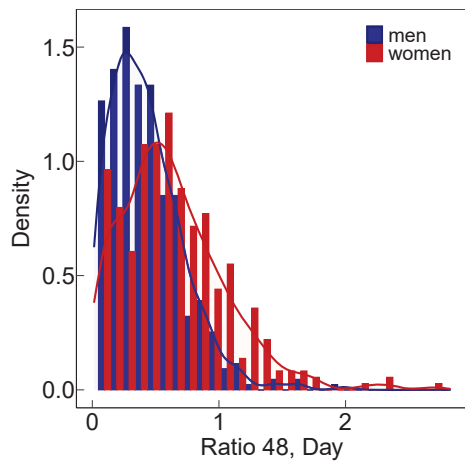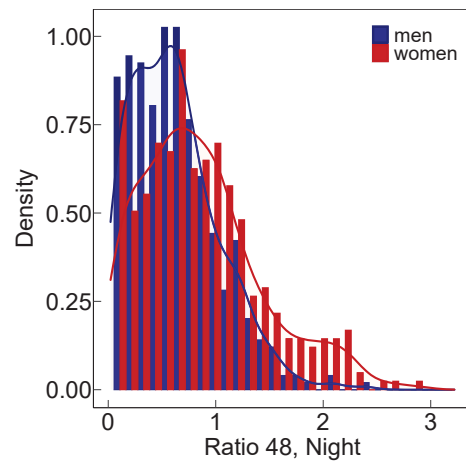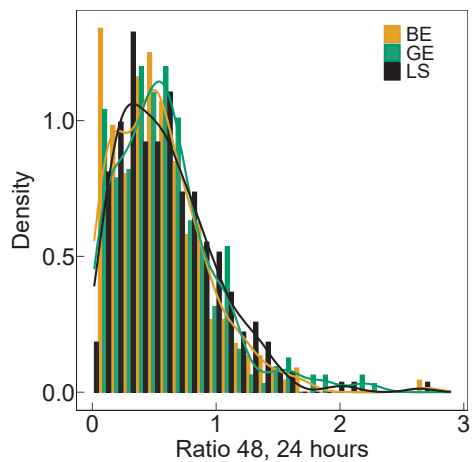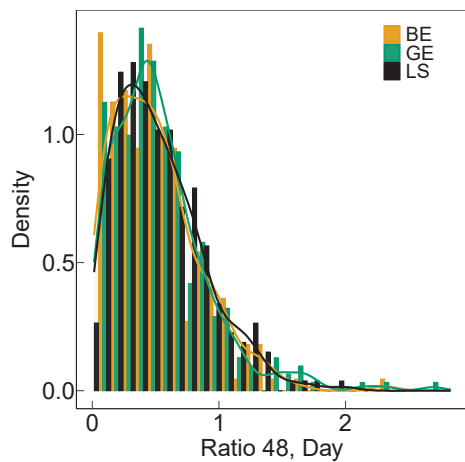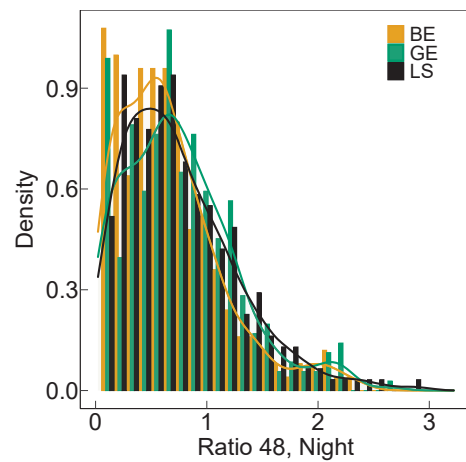

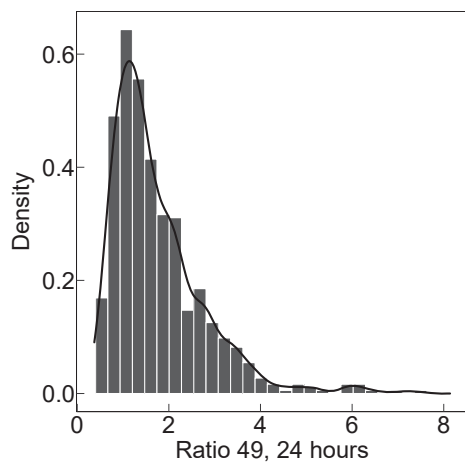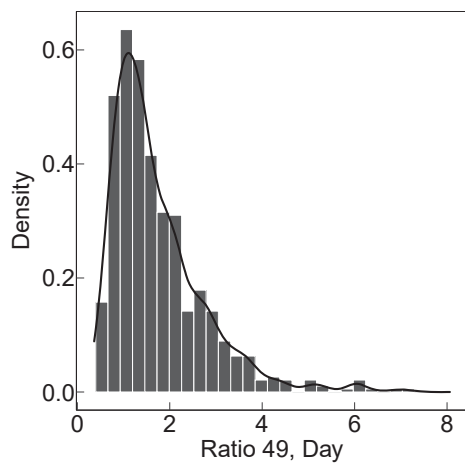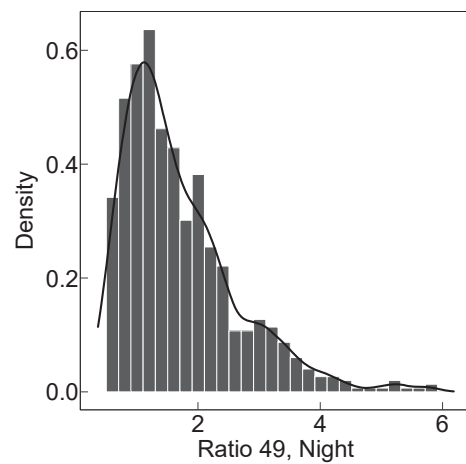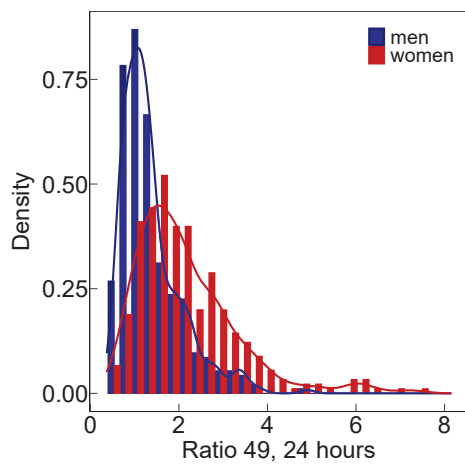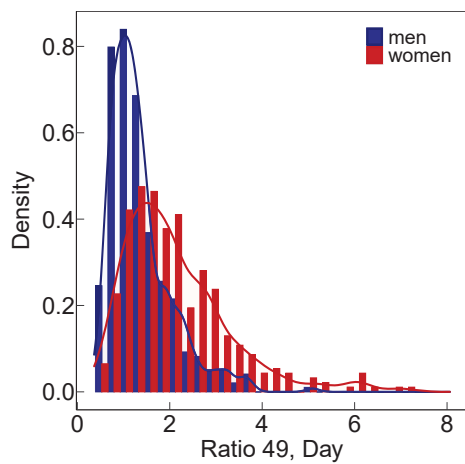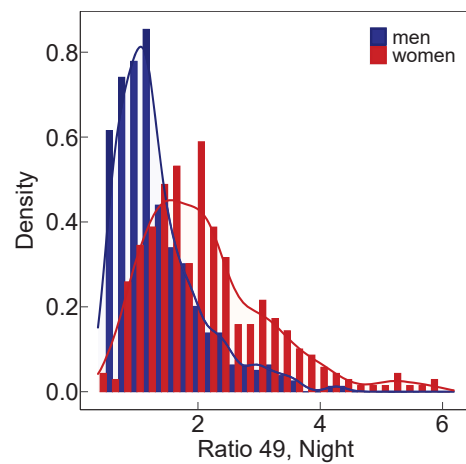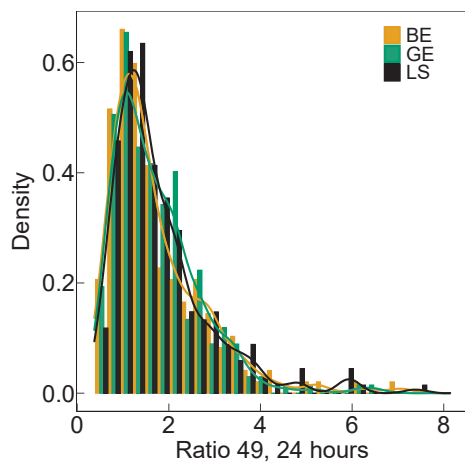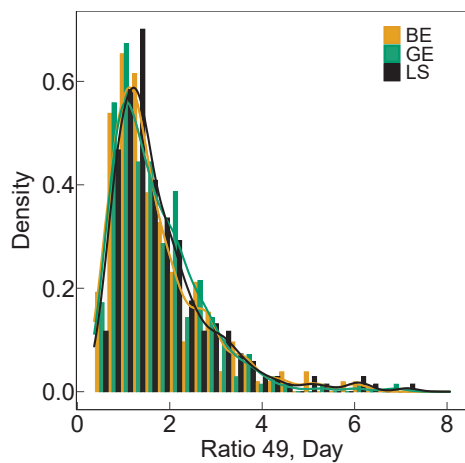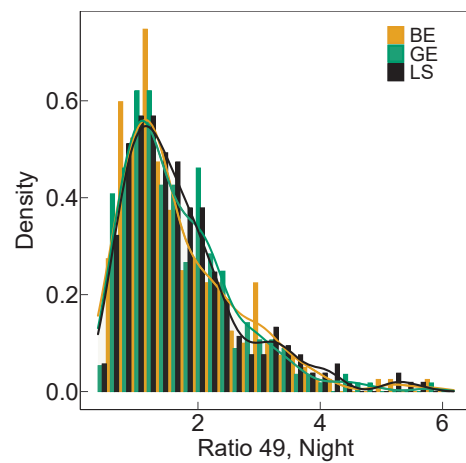

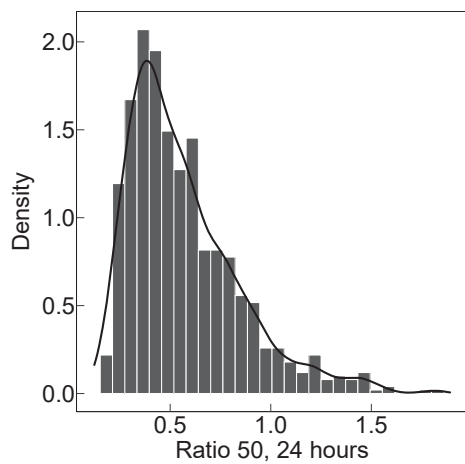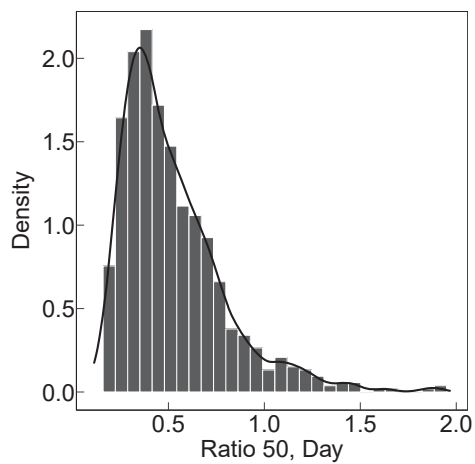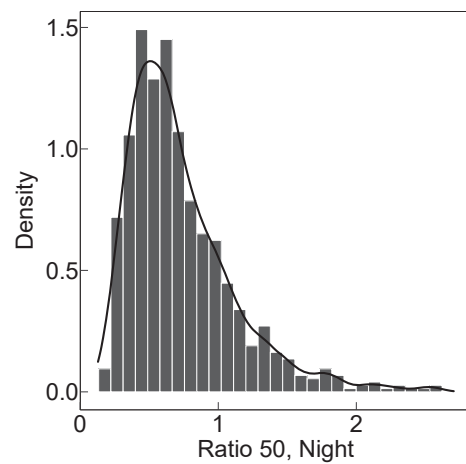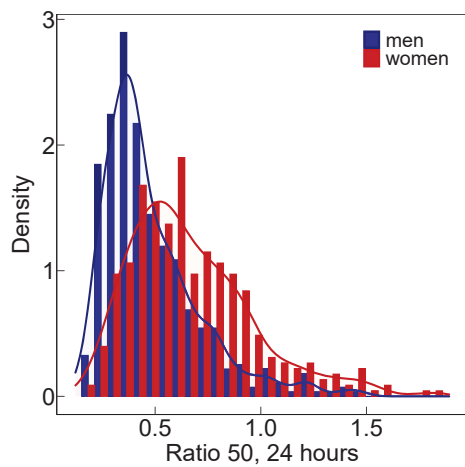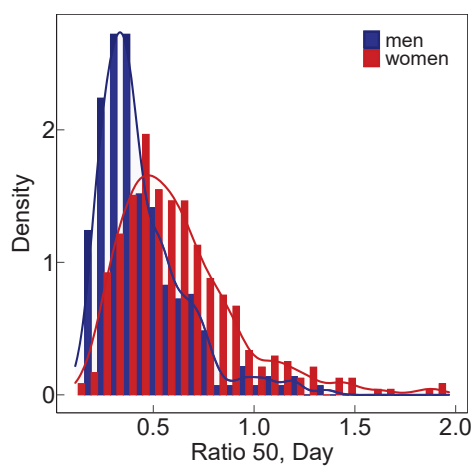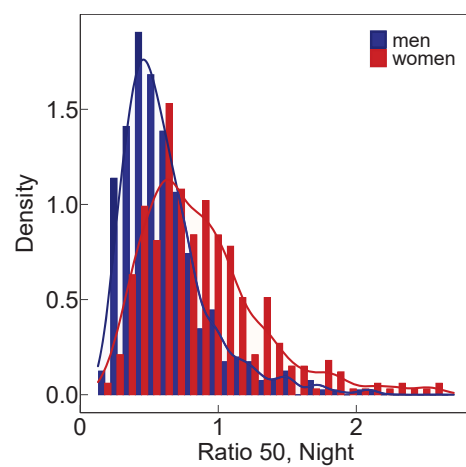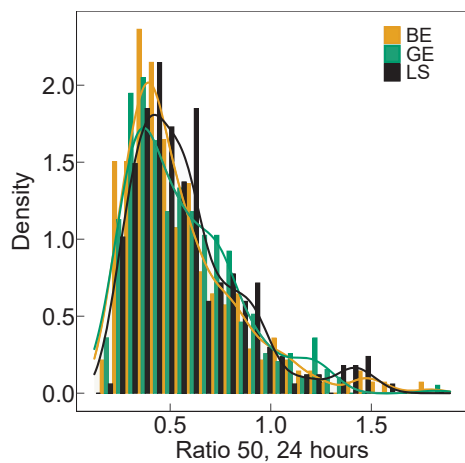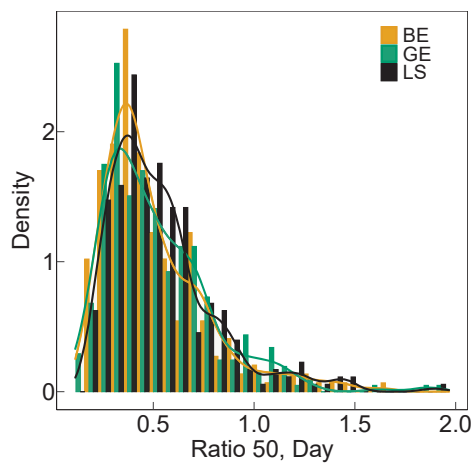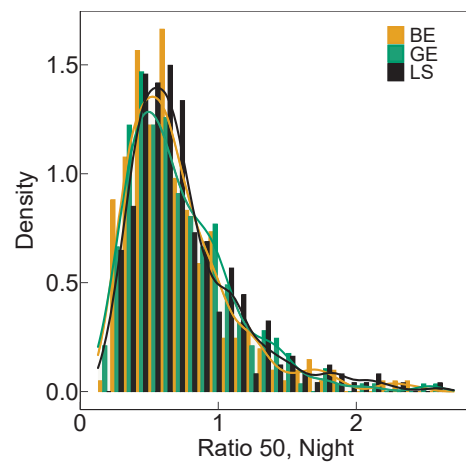

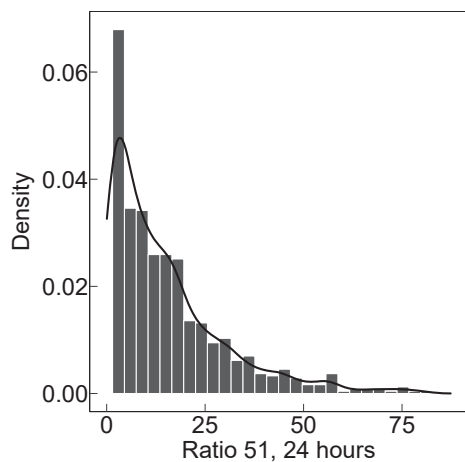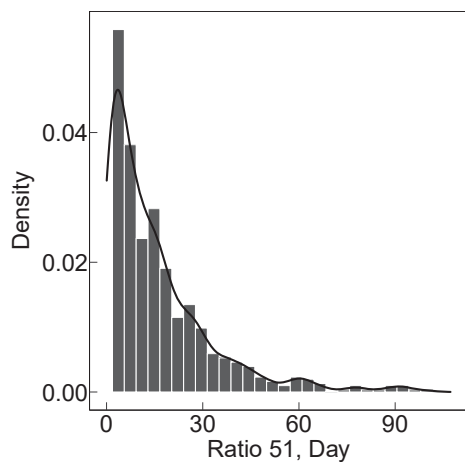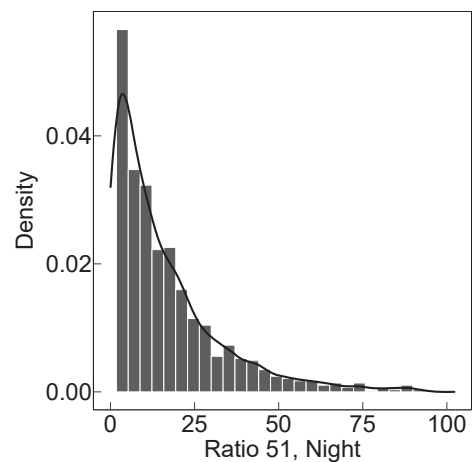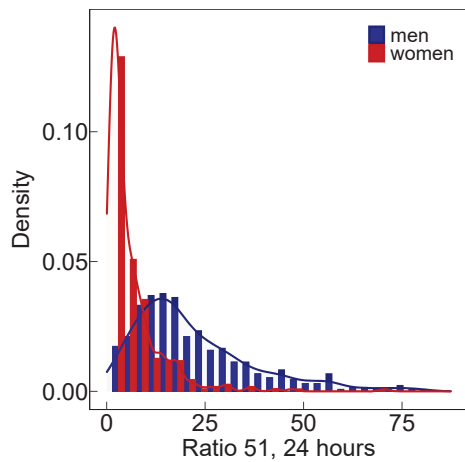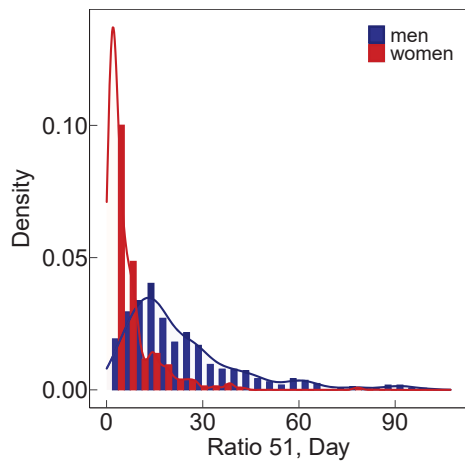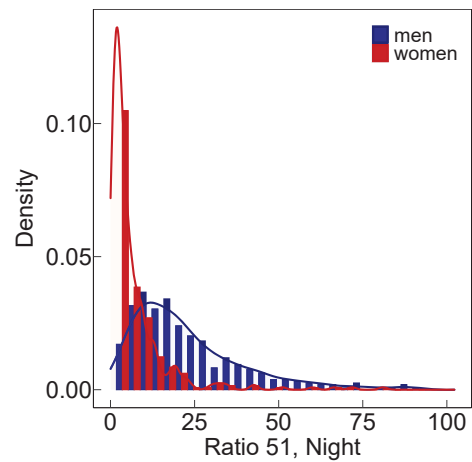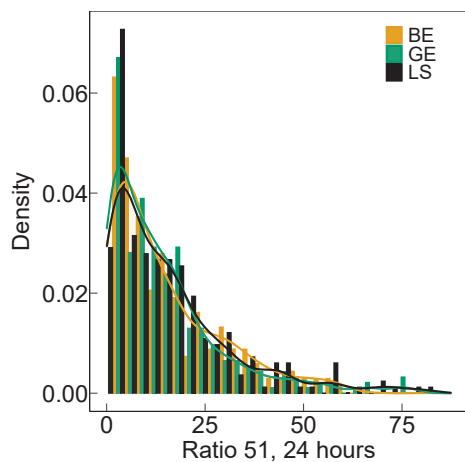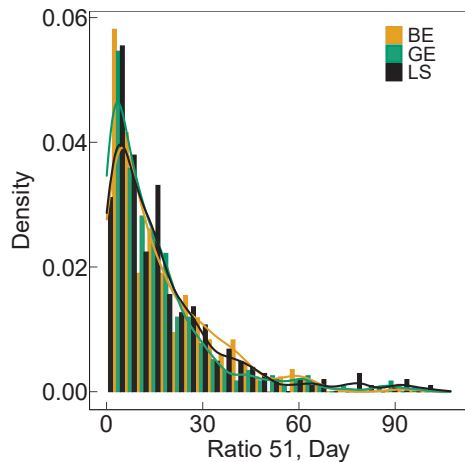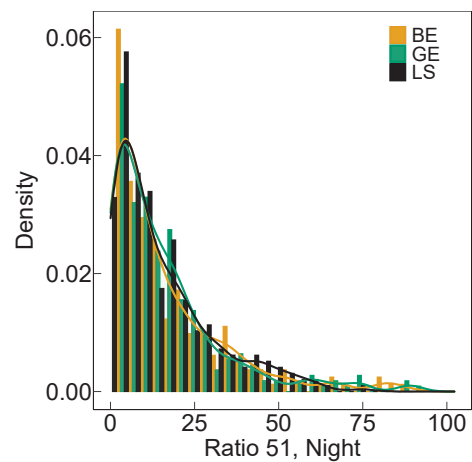

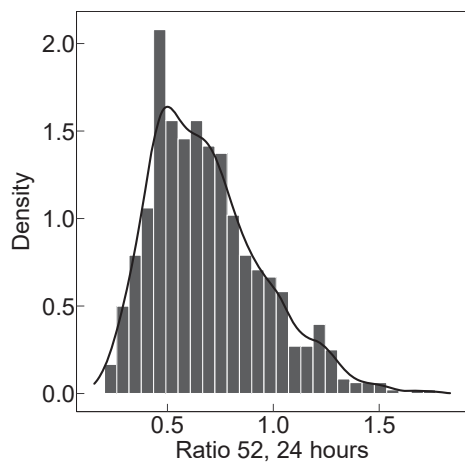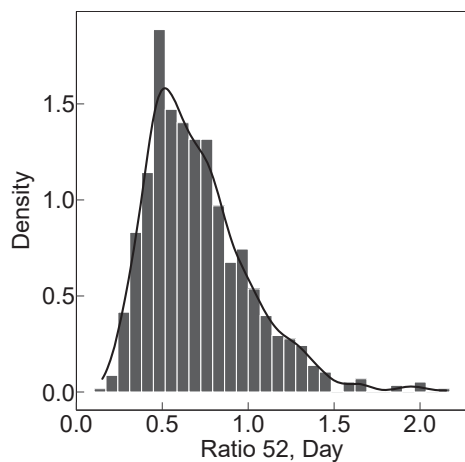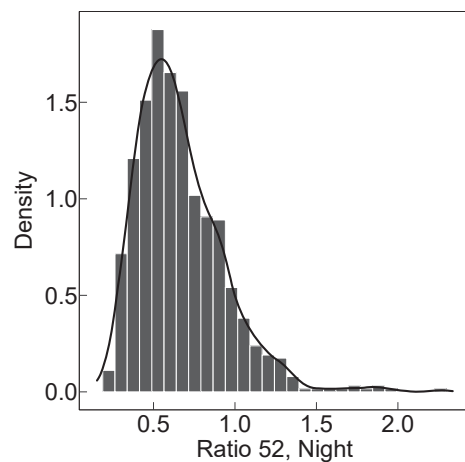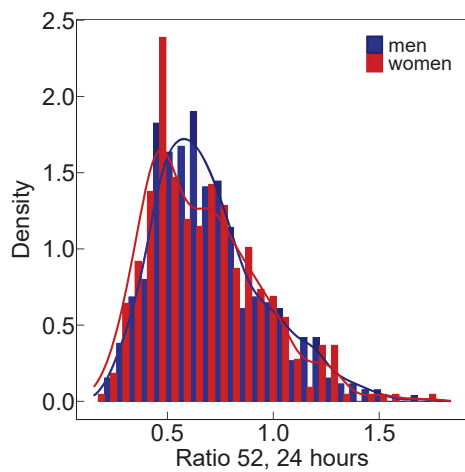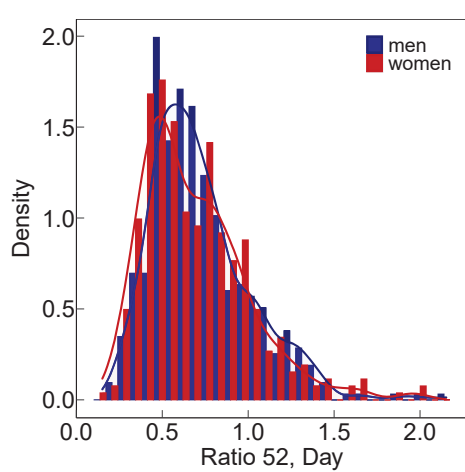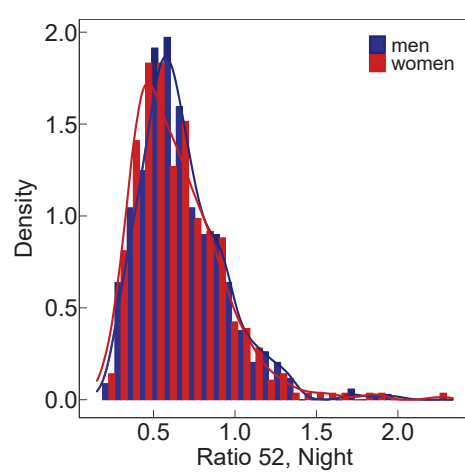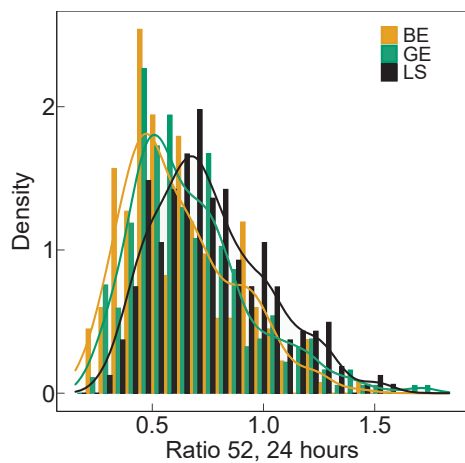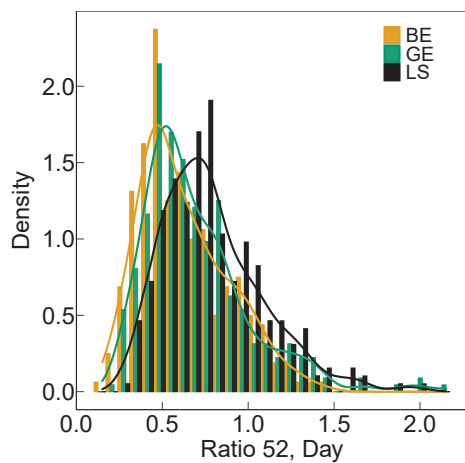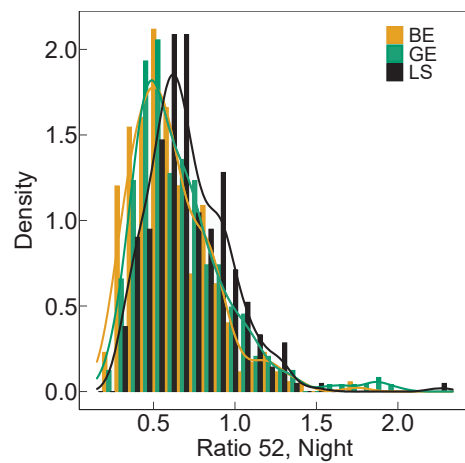

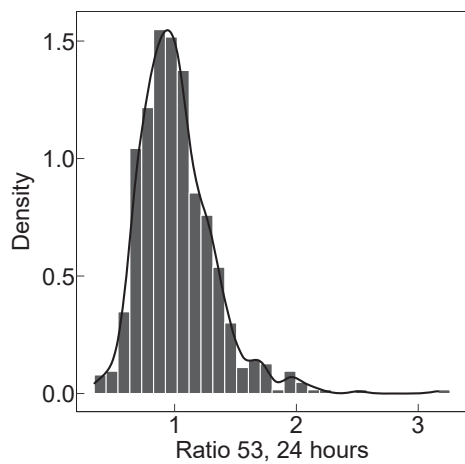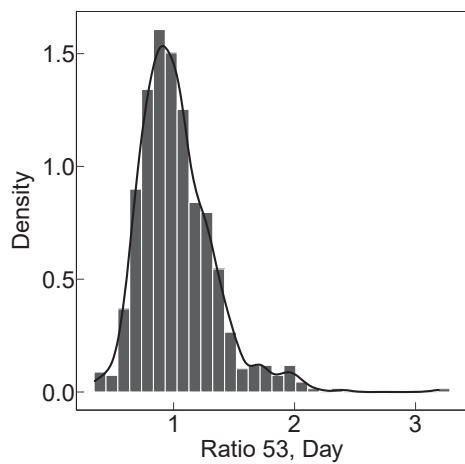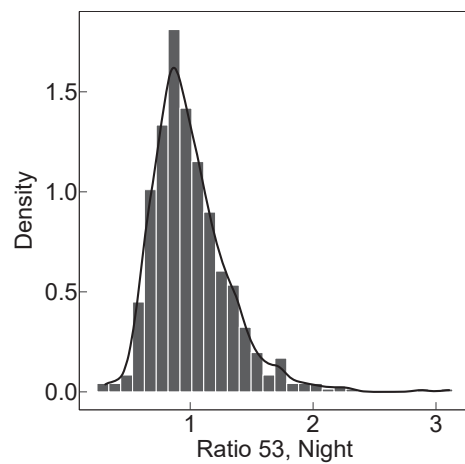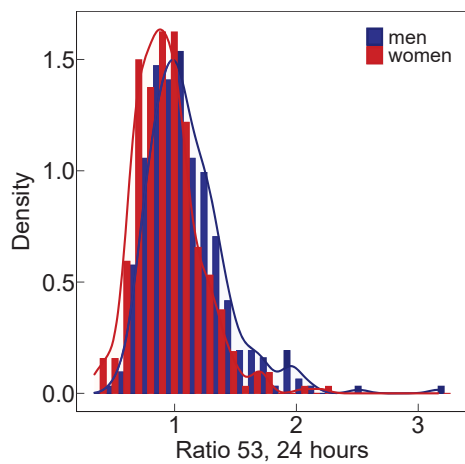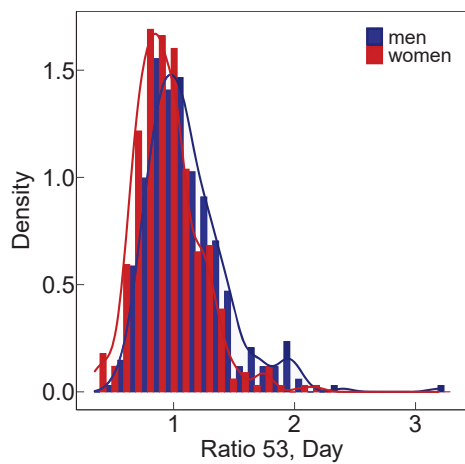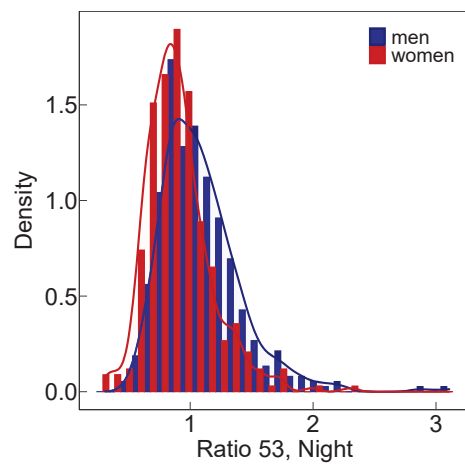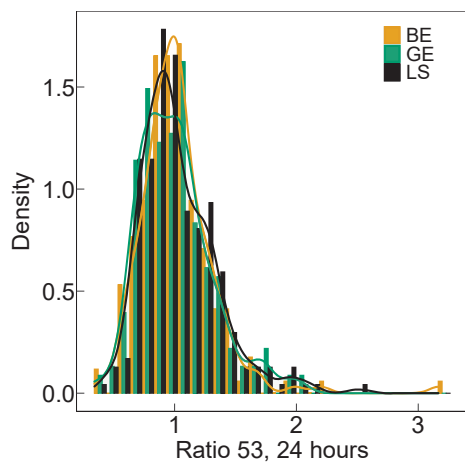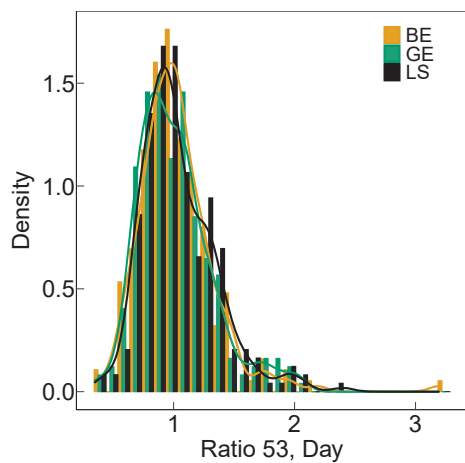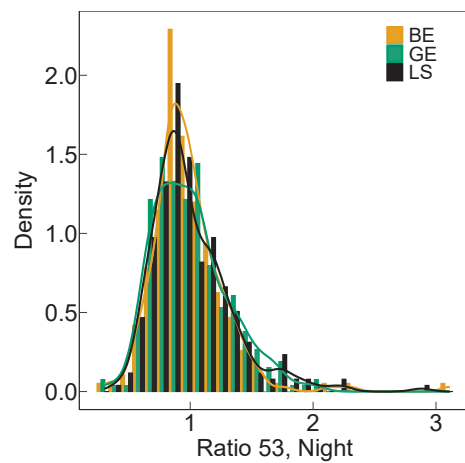

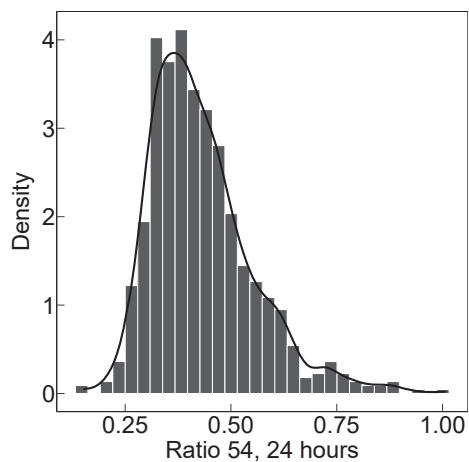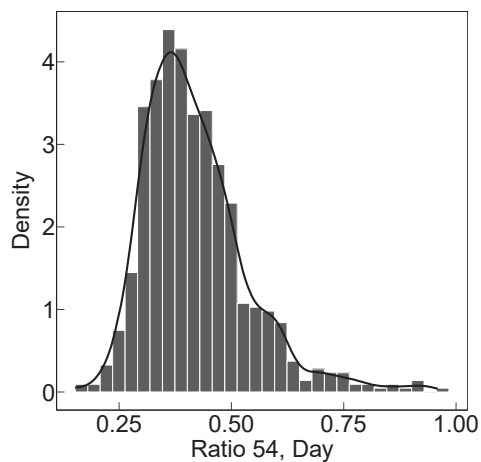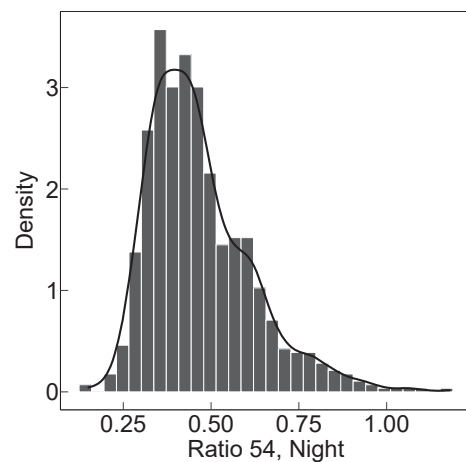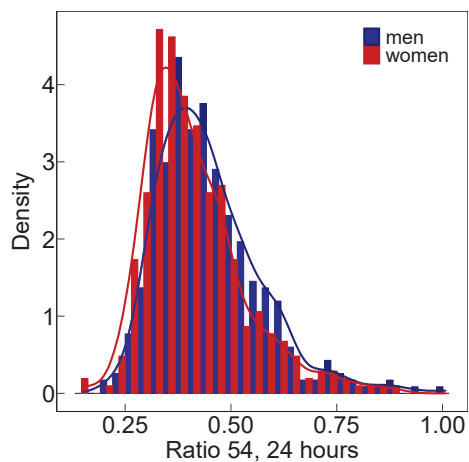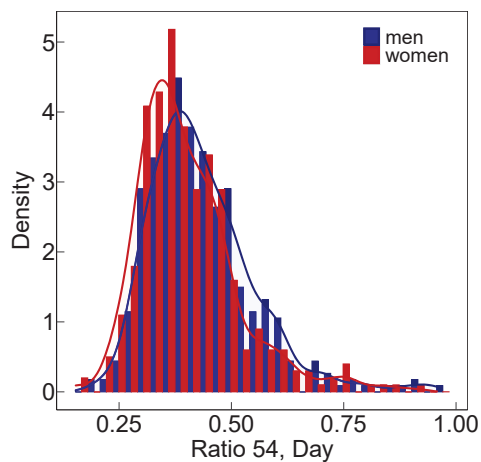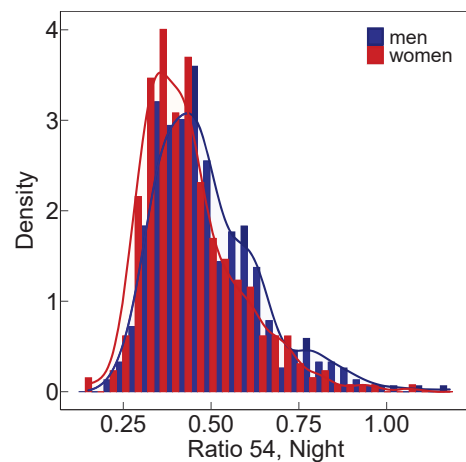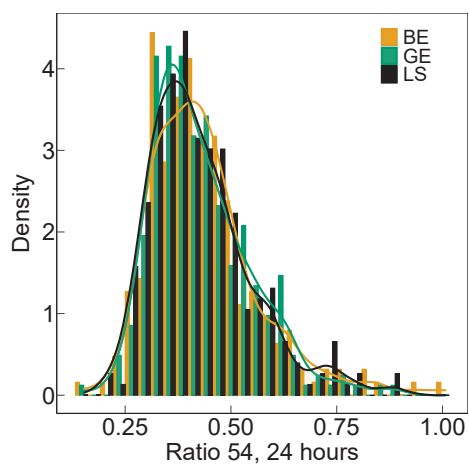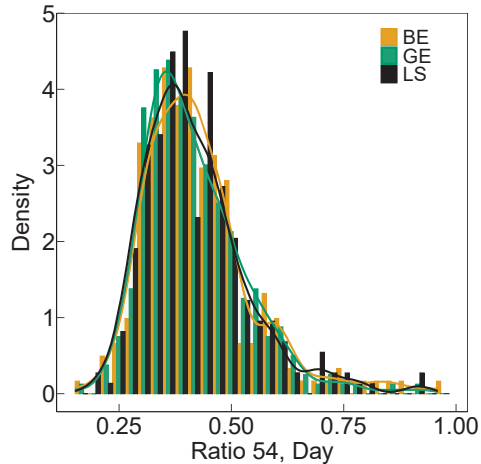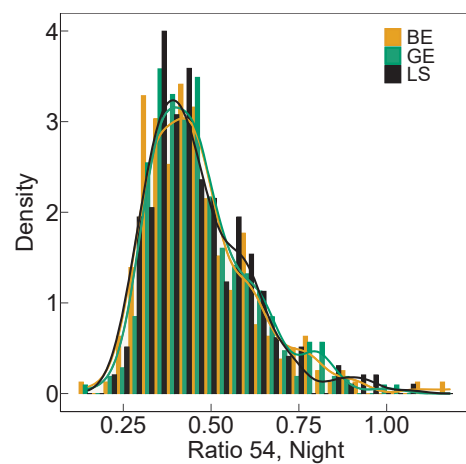

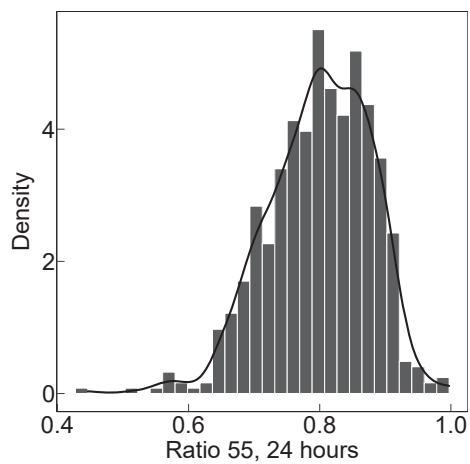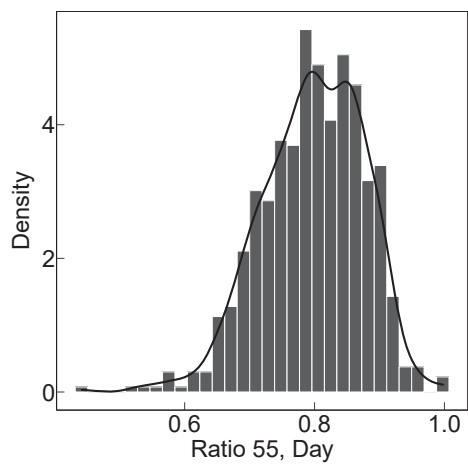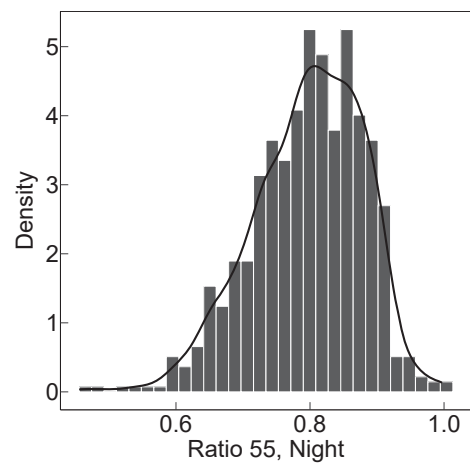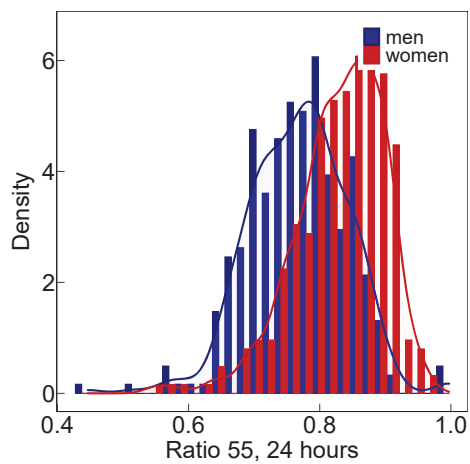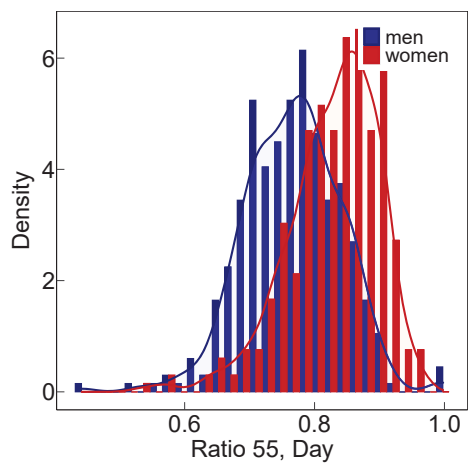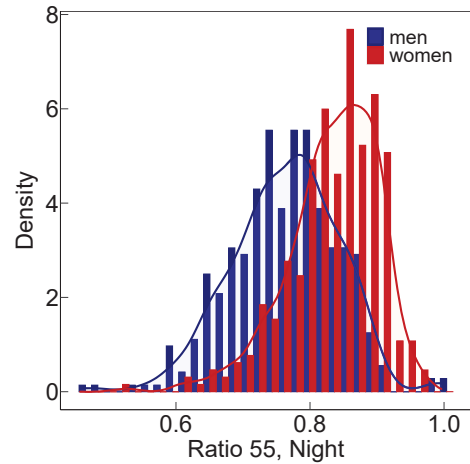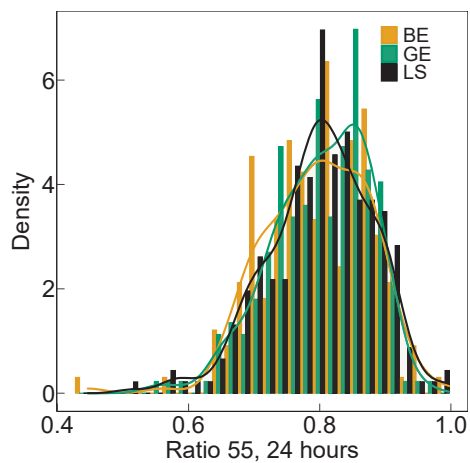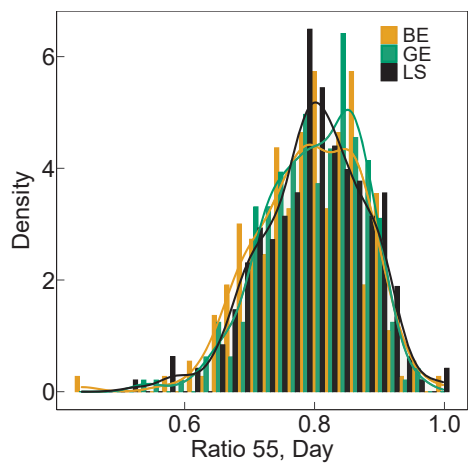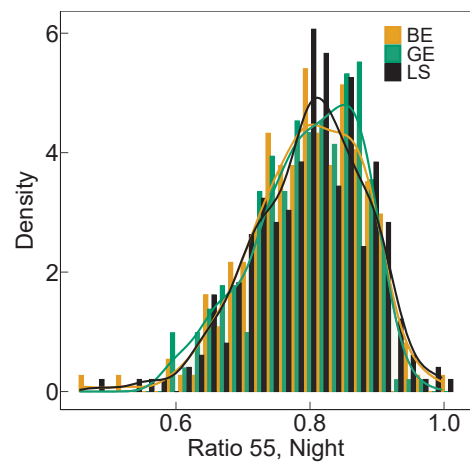

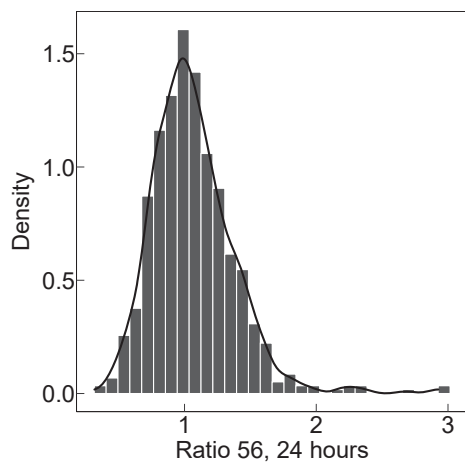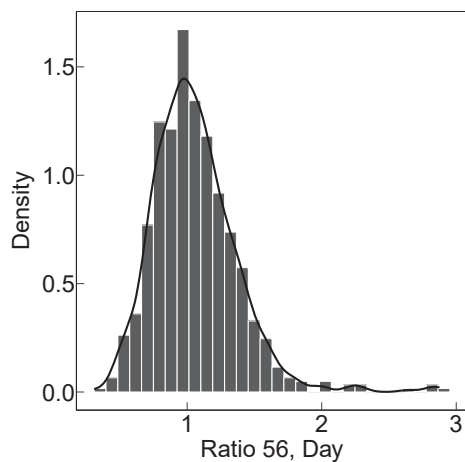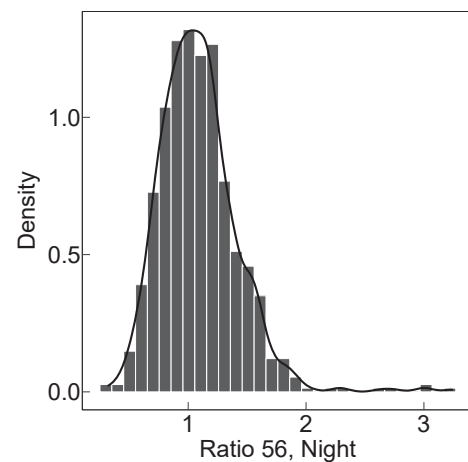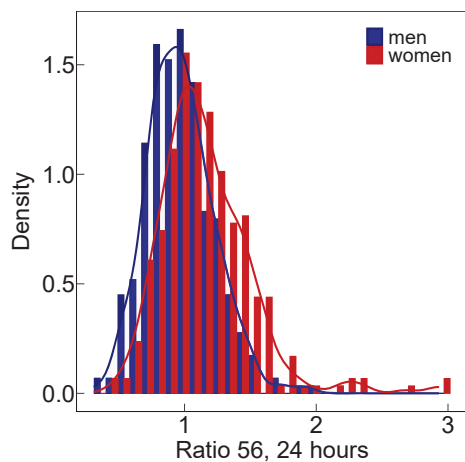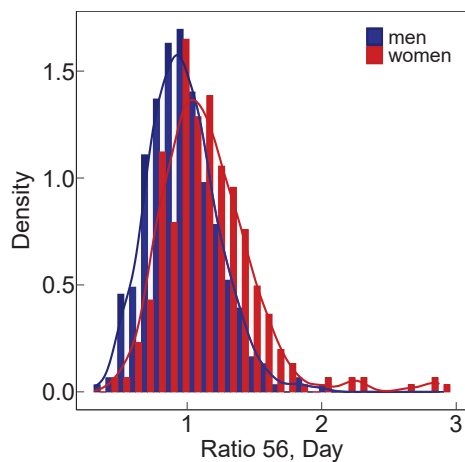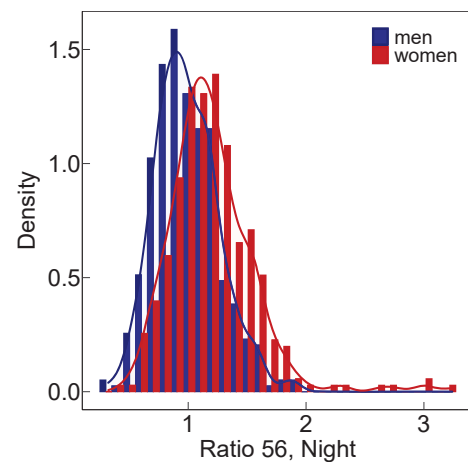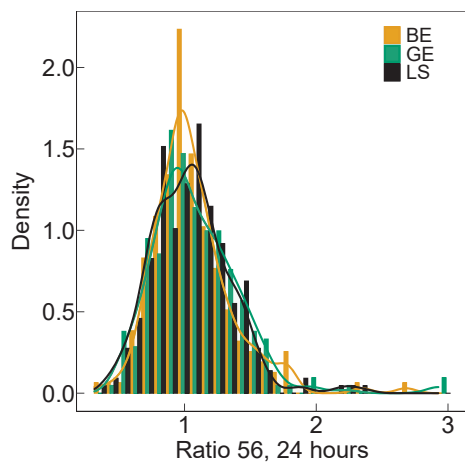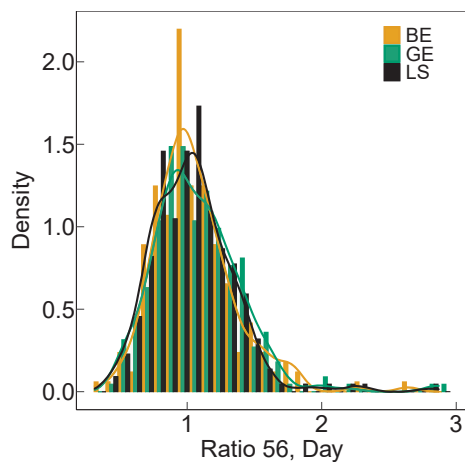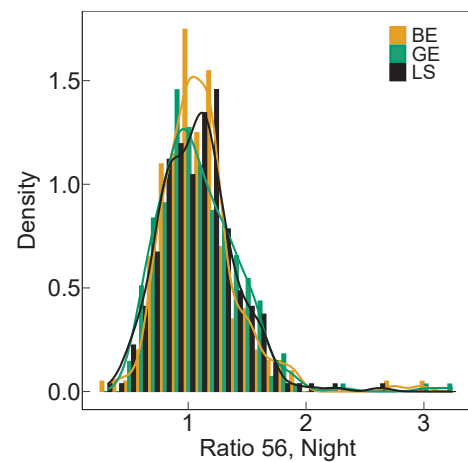

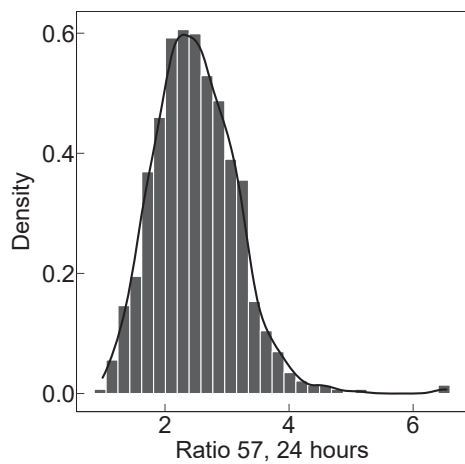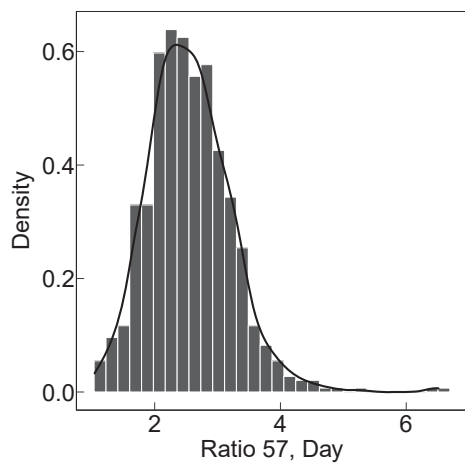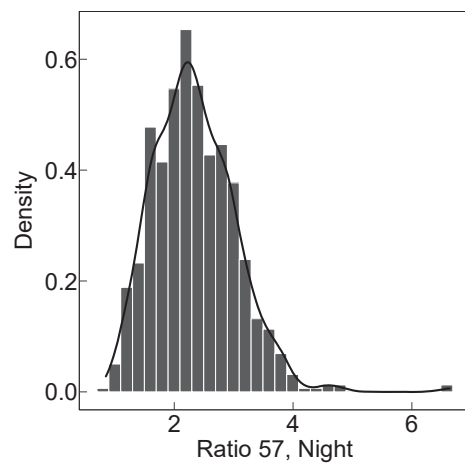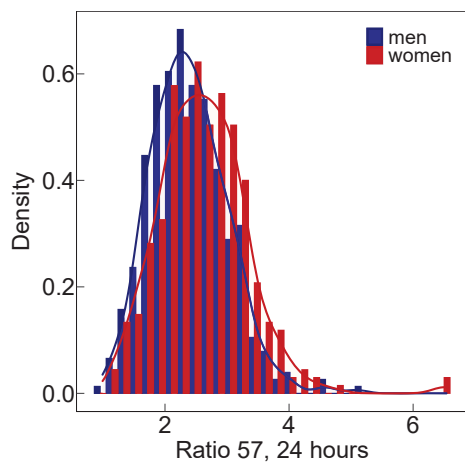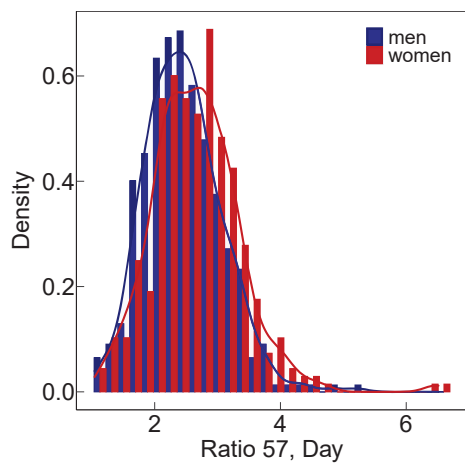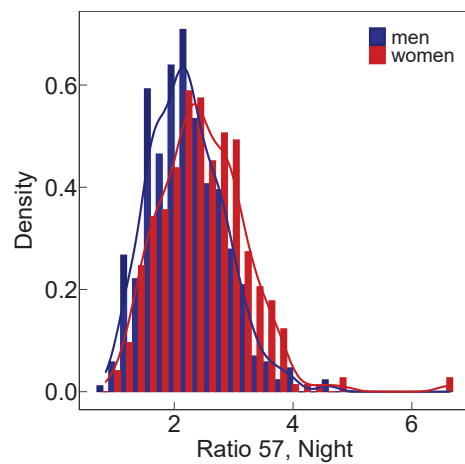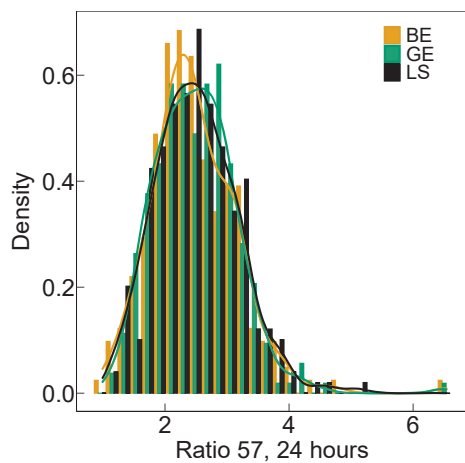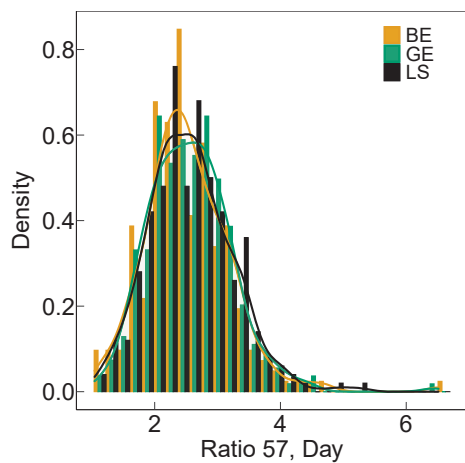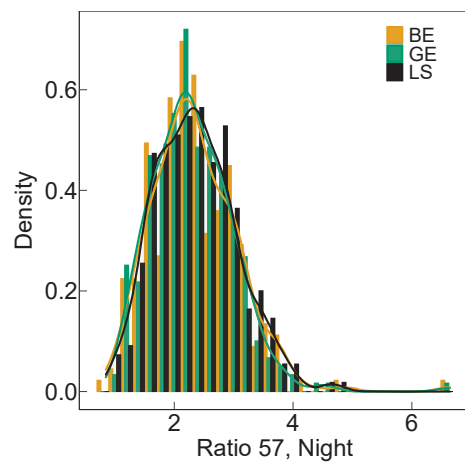

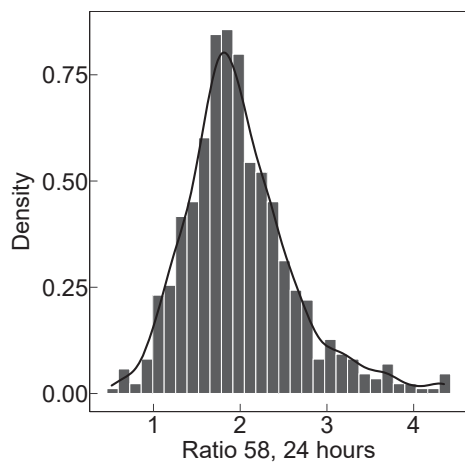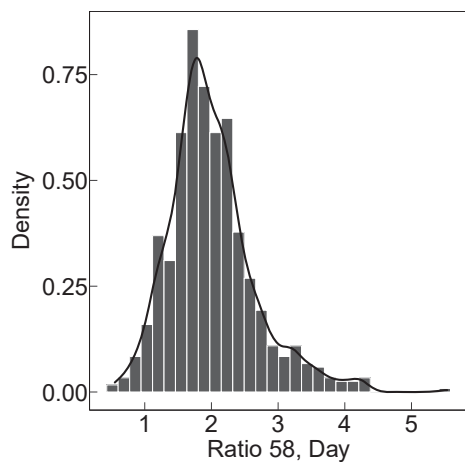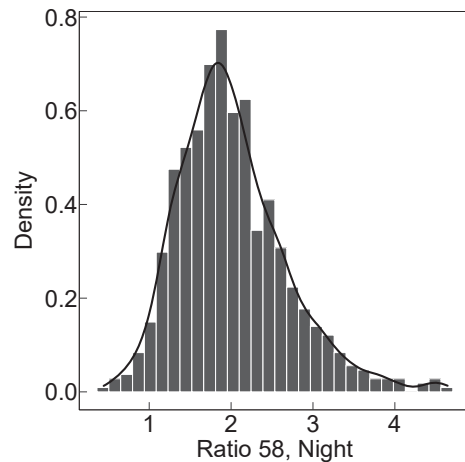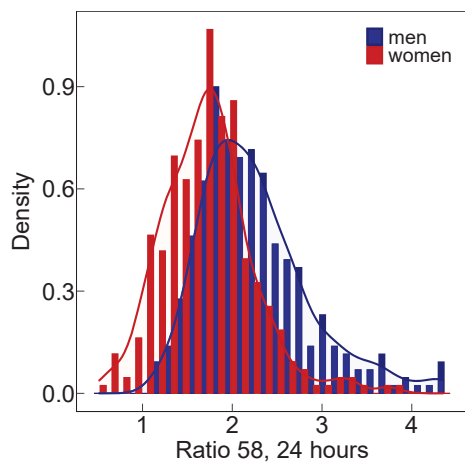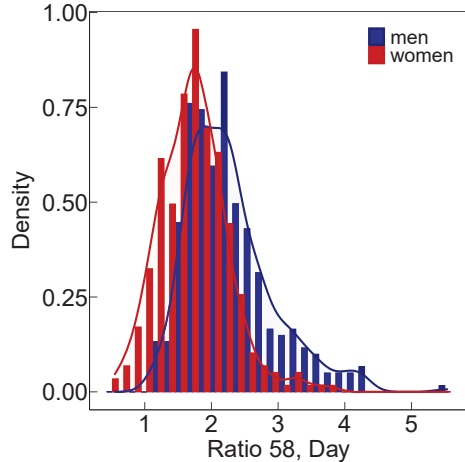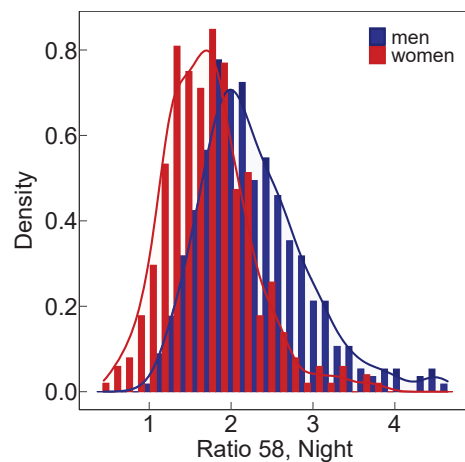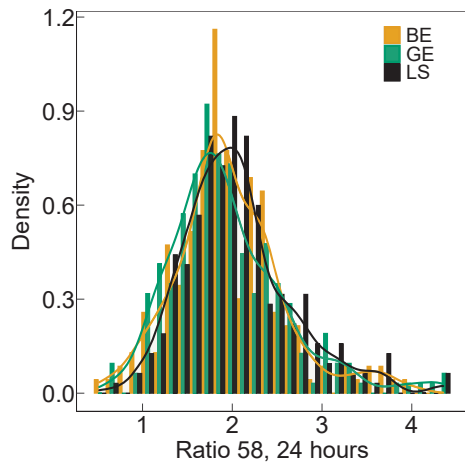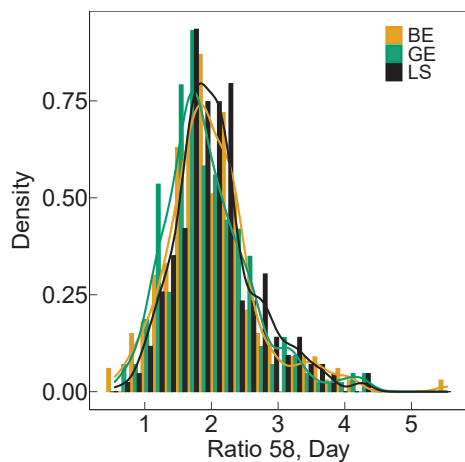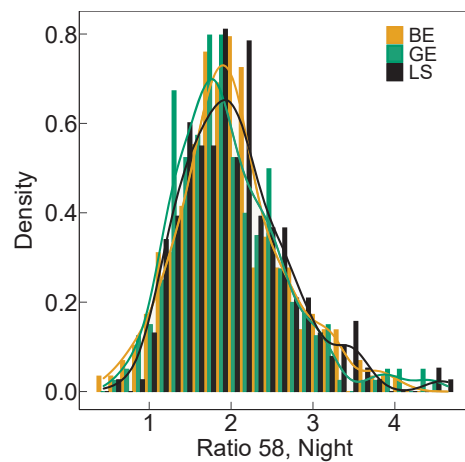

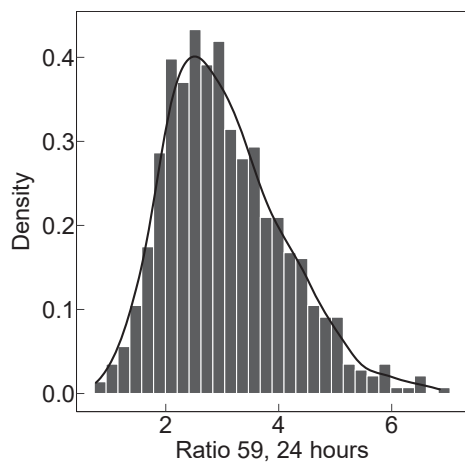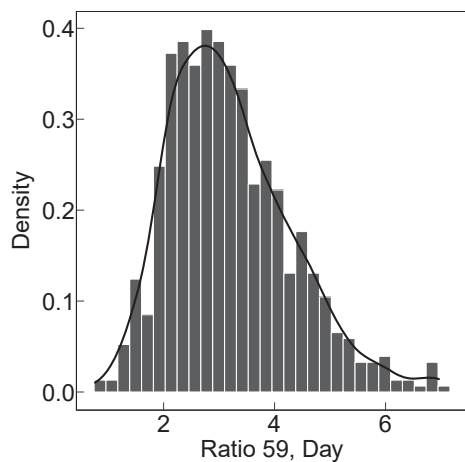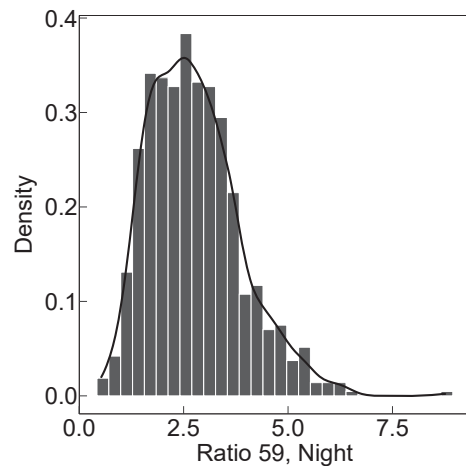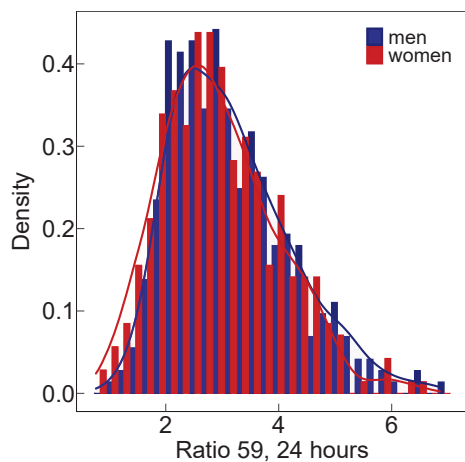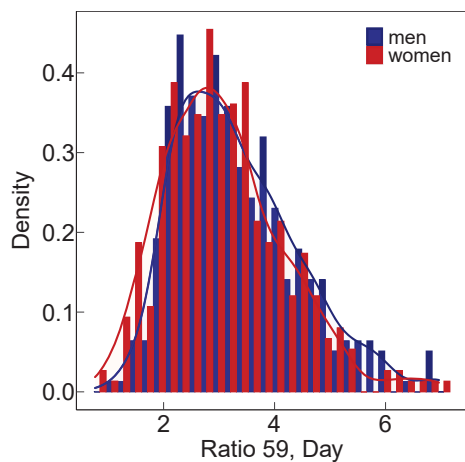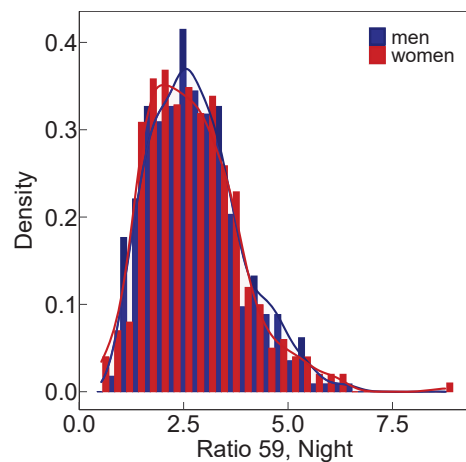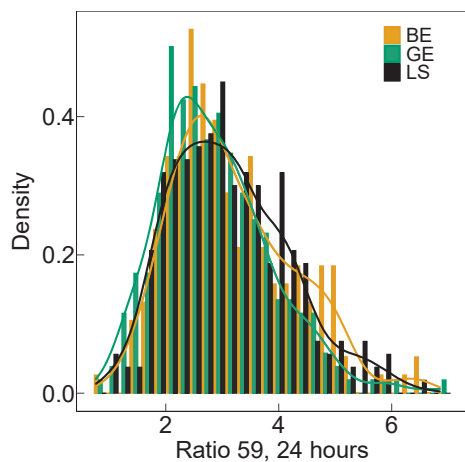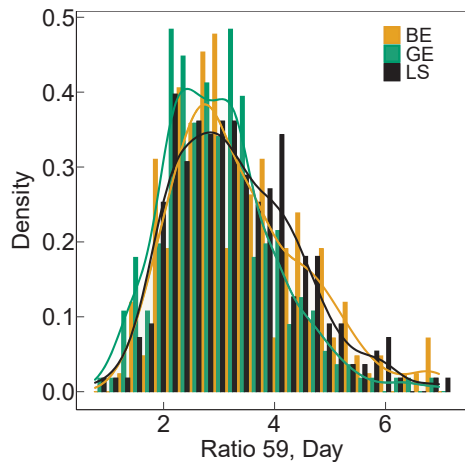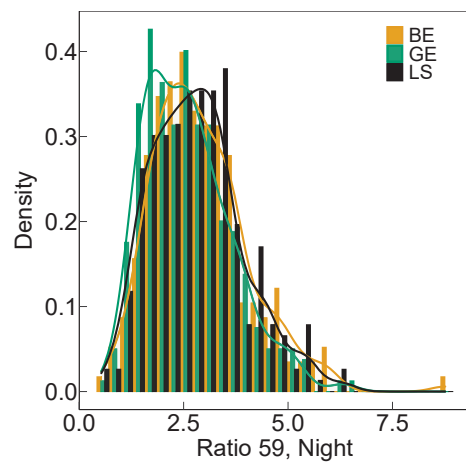

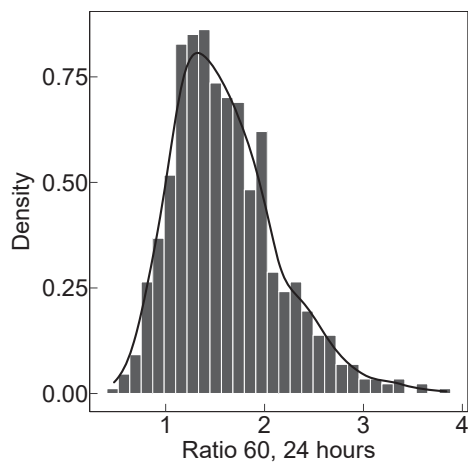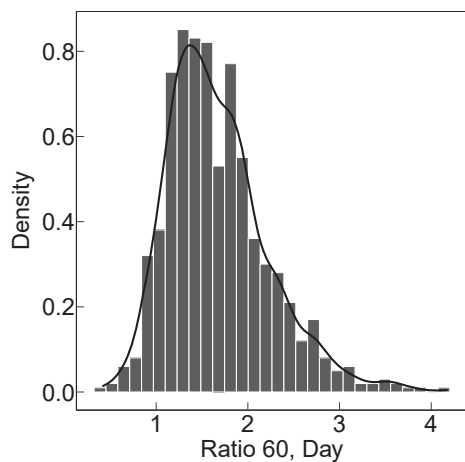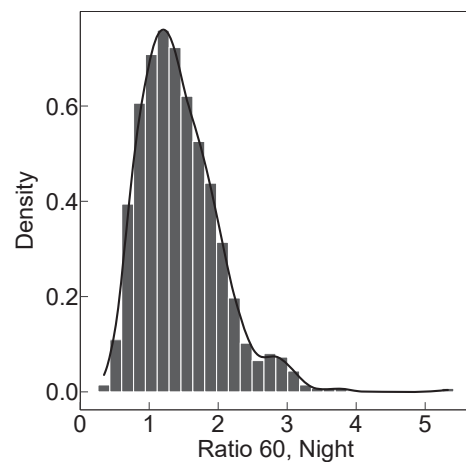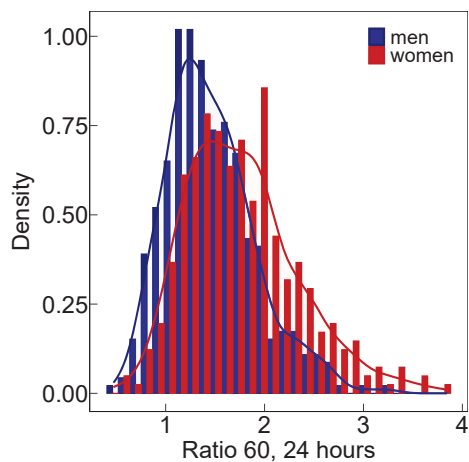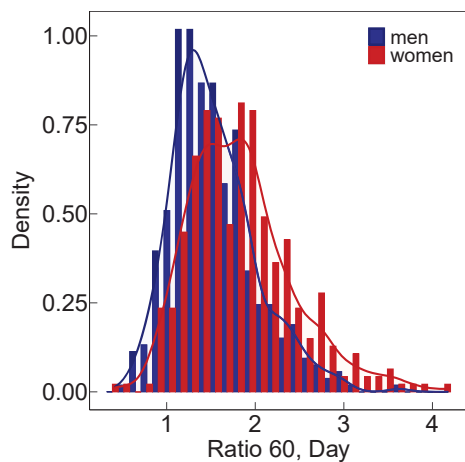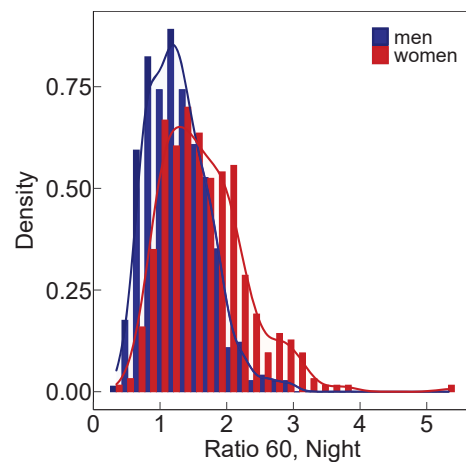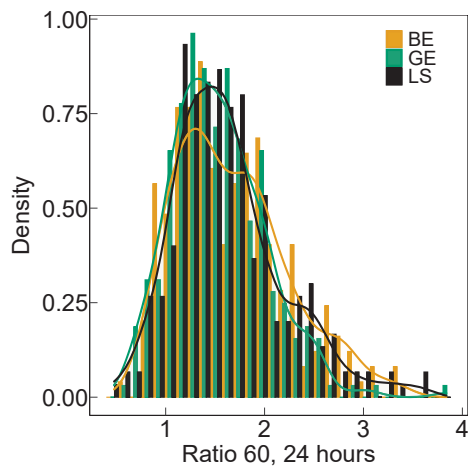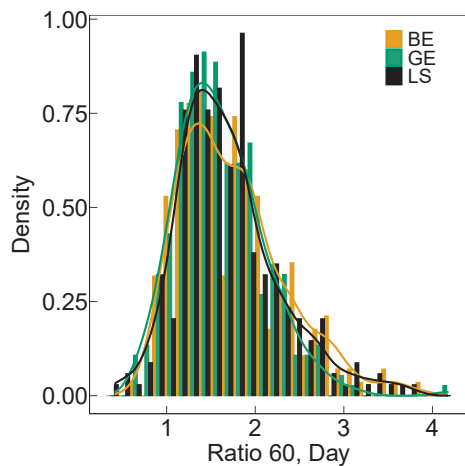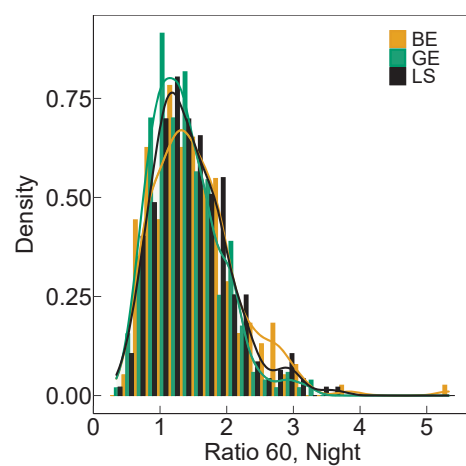

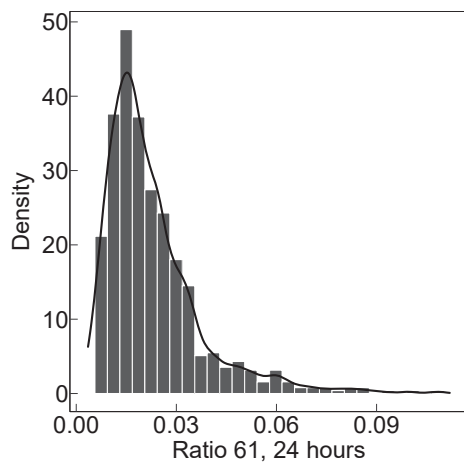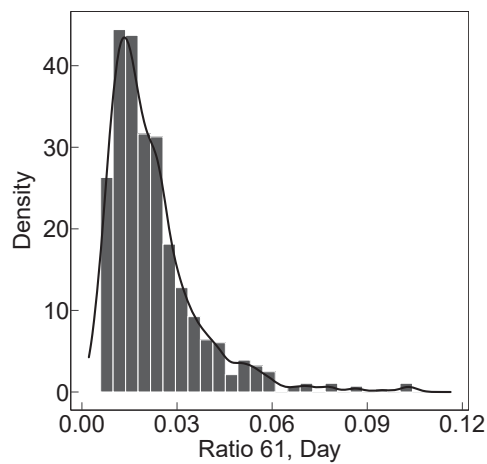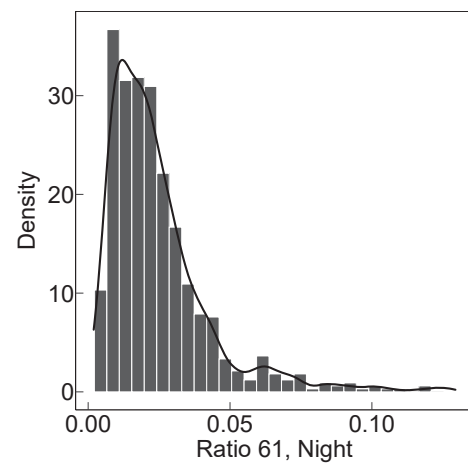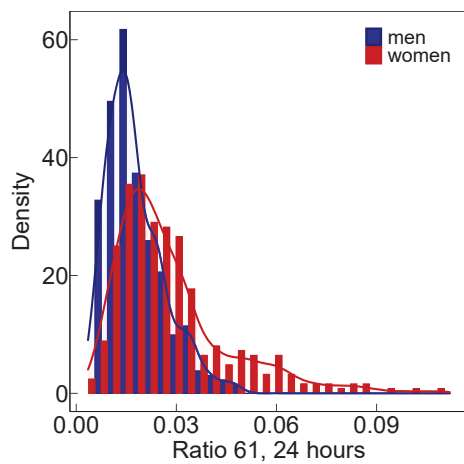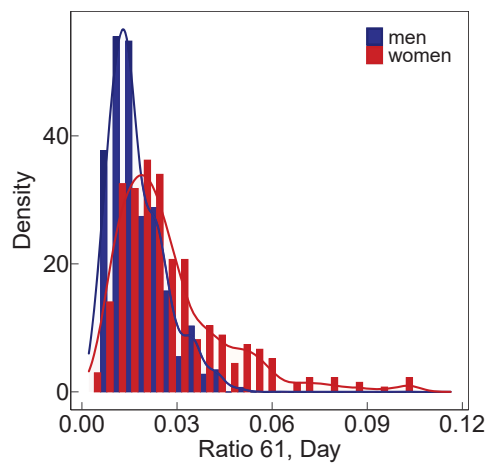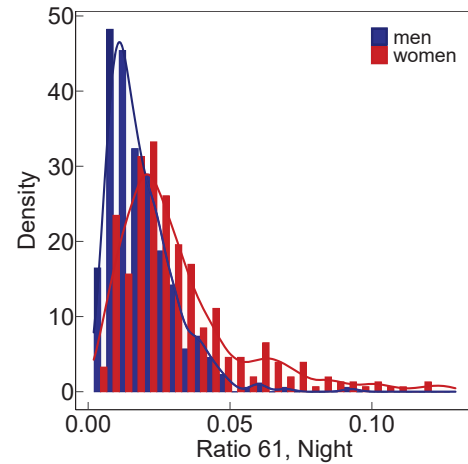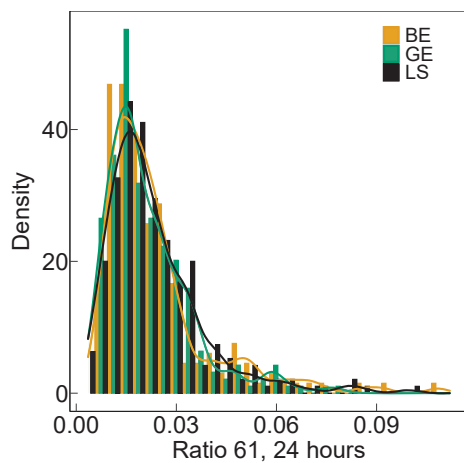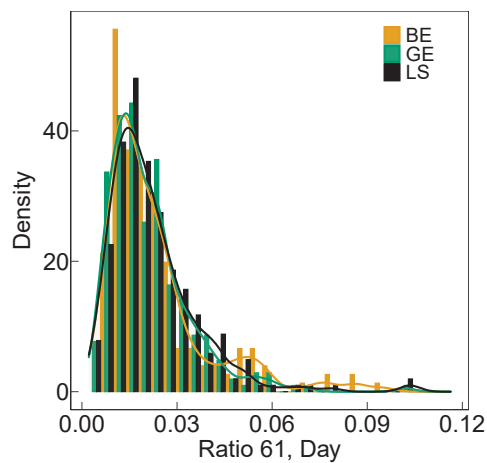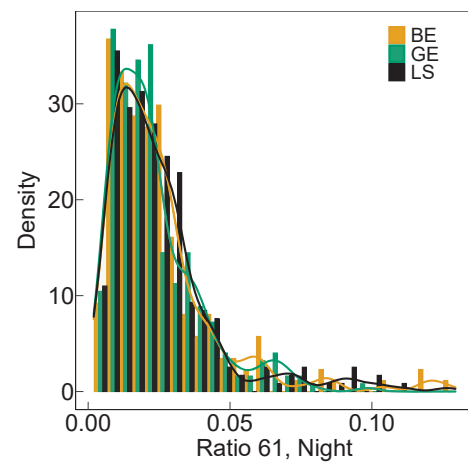

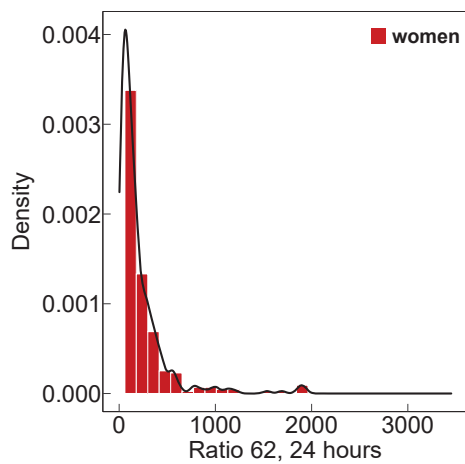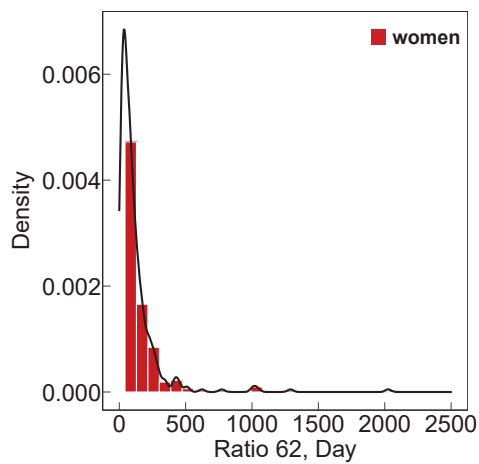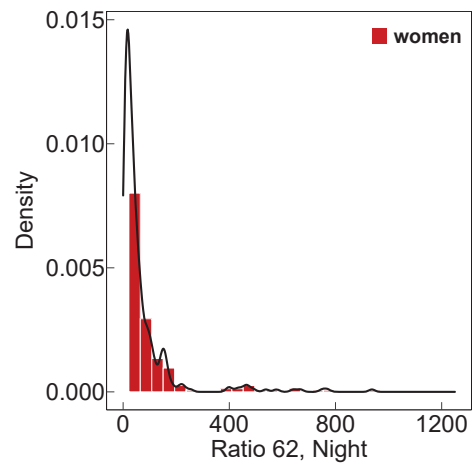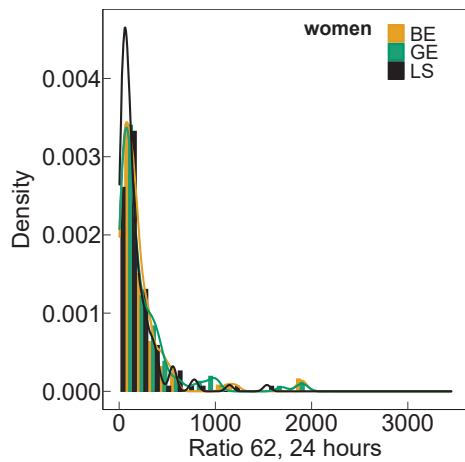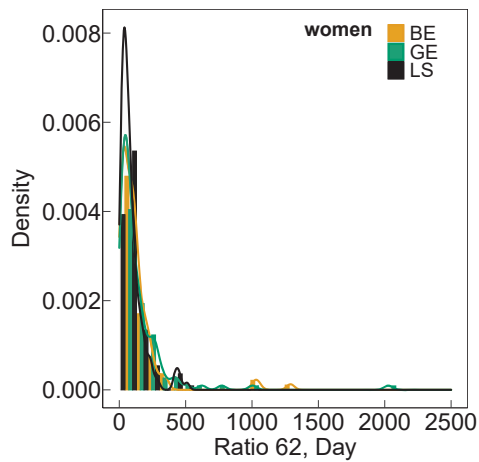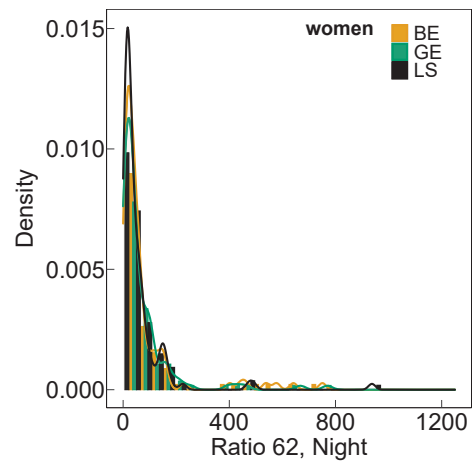

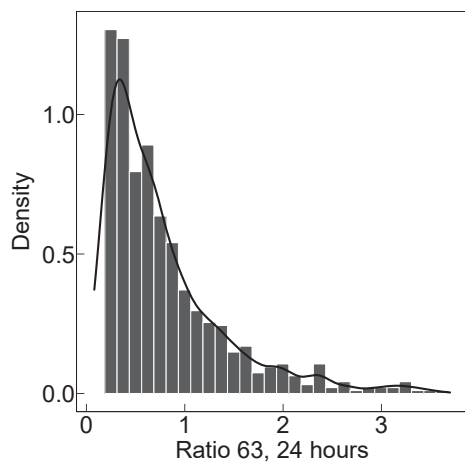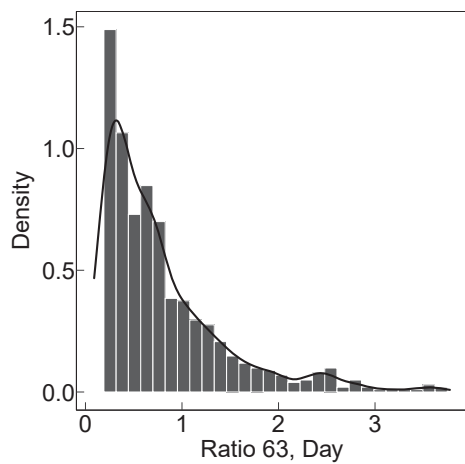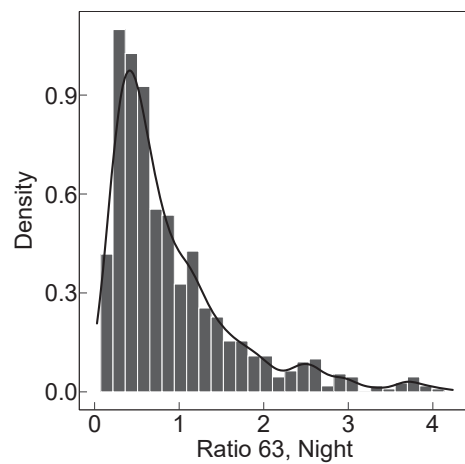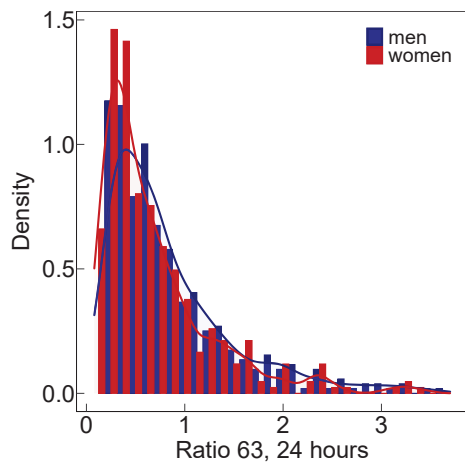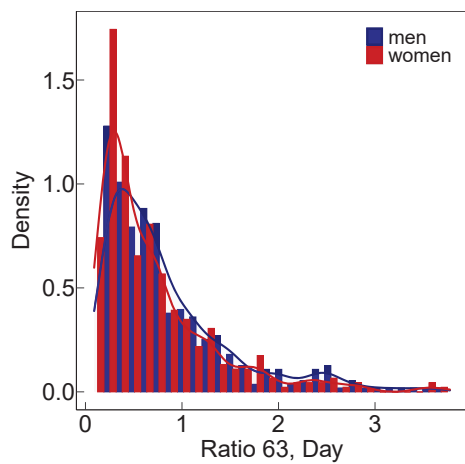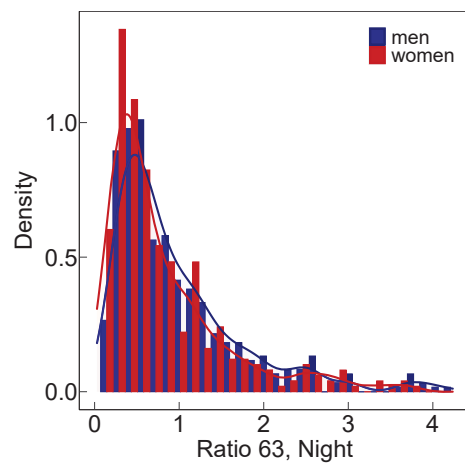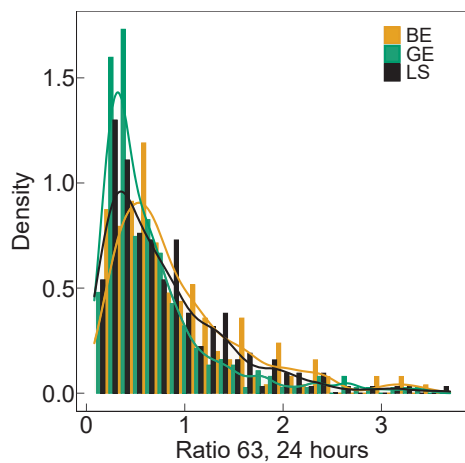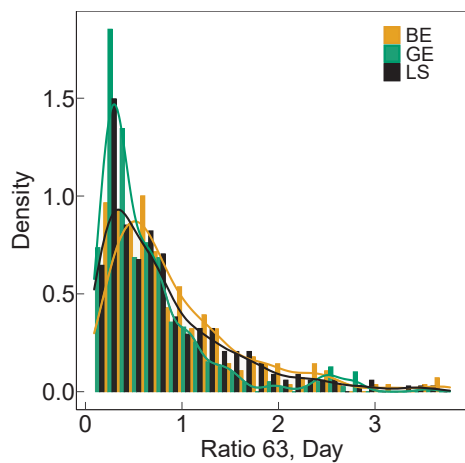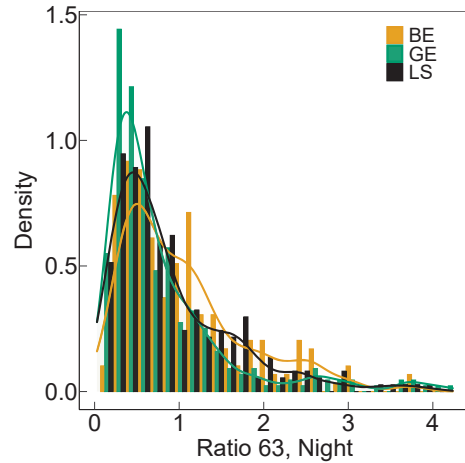

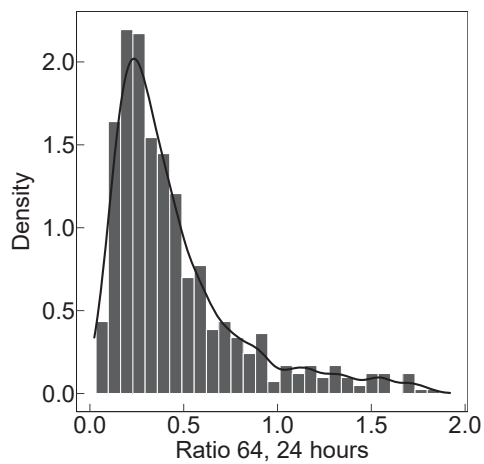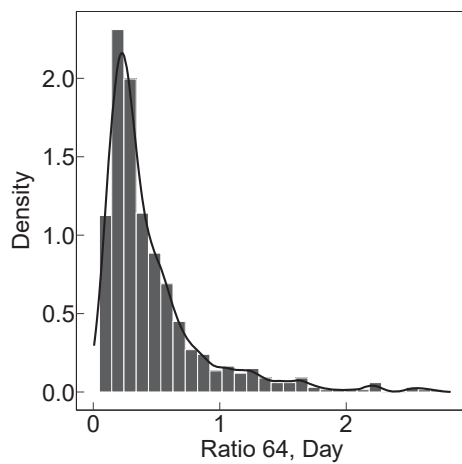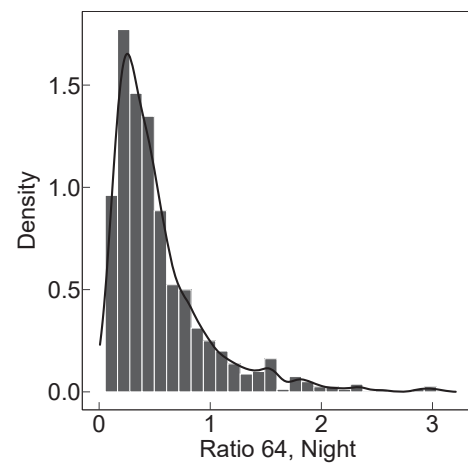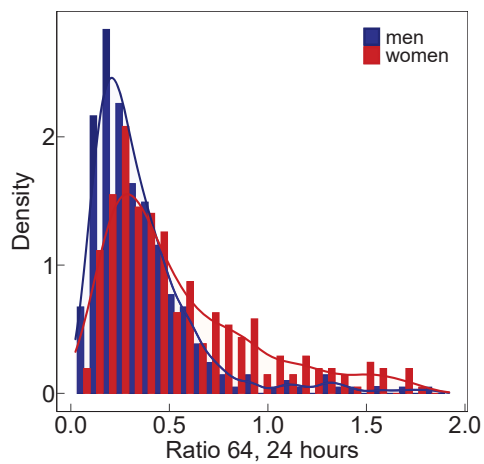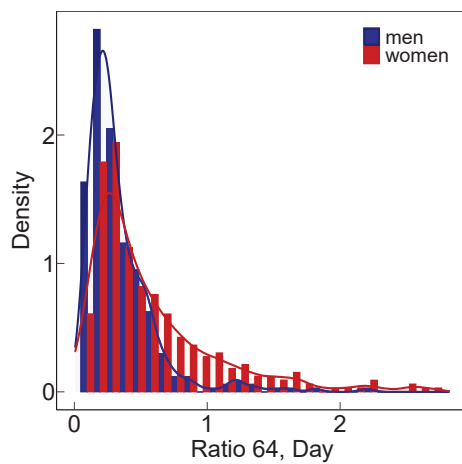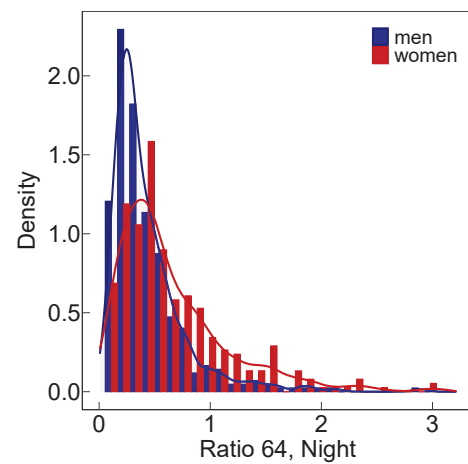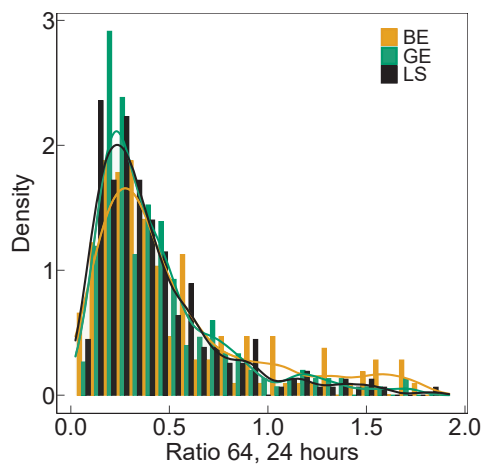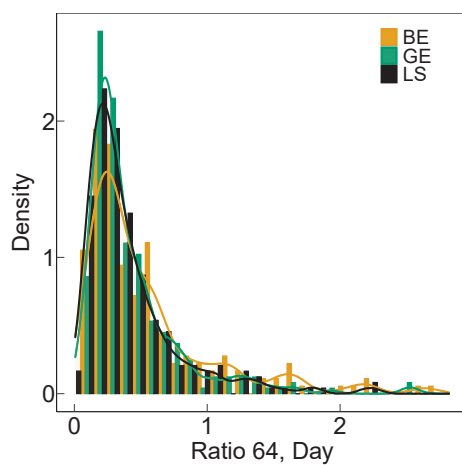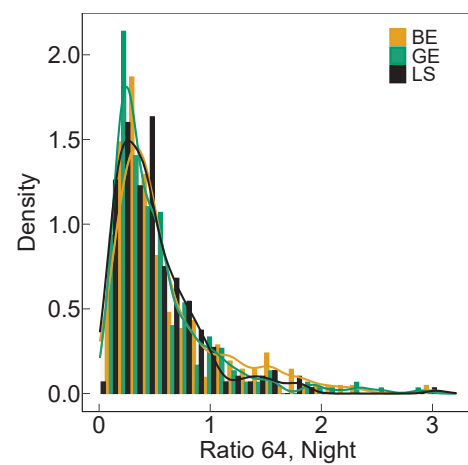

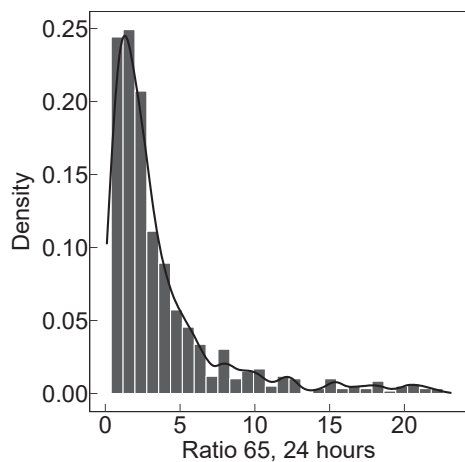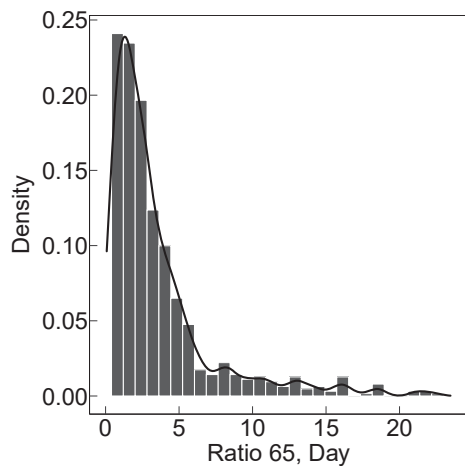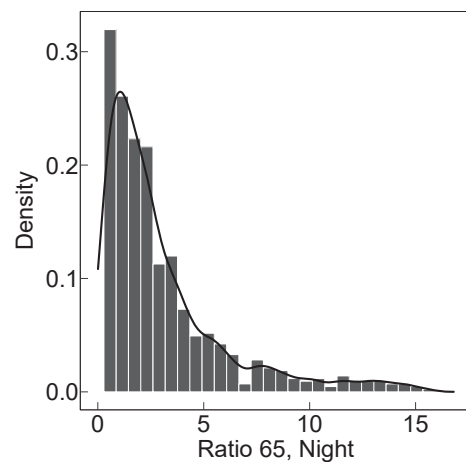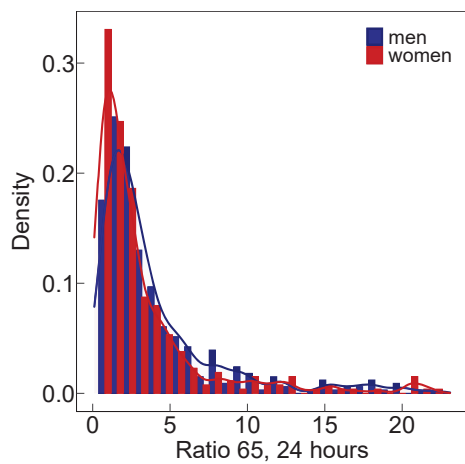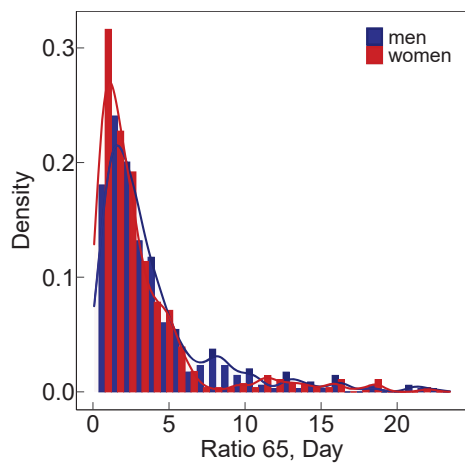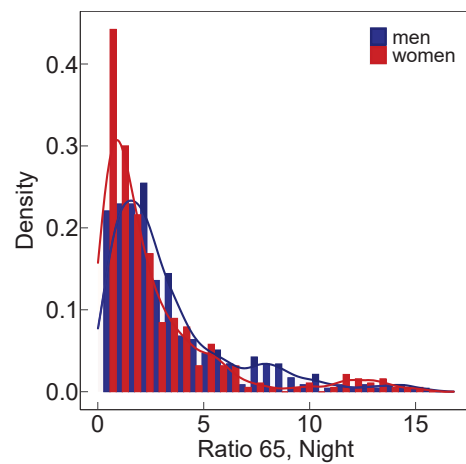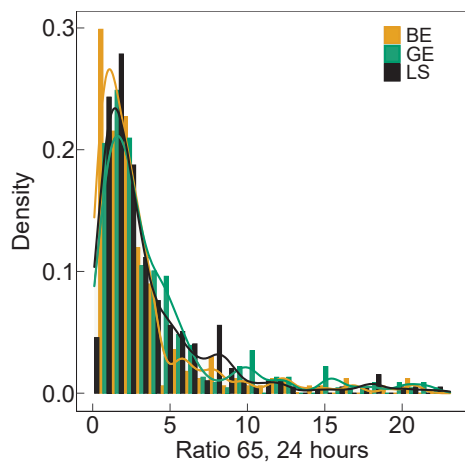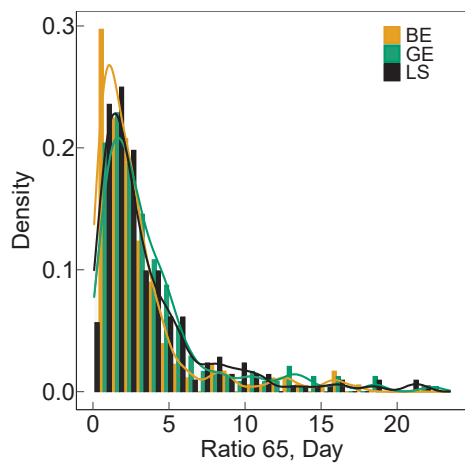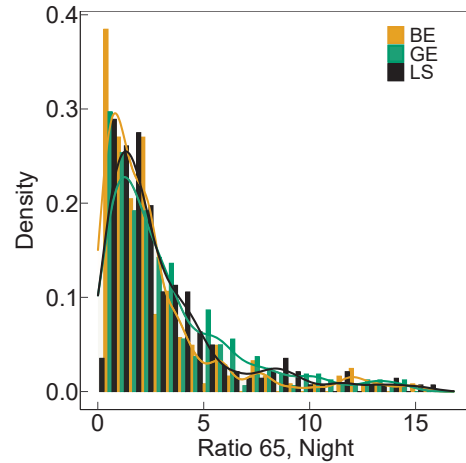

Supplement: S2 Fig — Figures from S1 Fig were remade and the tail from extremely right-skewed distributions were truncated after visual inspection for improved visibility. (PDF) [file pone.0253975.s002.pdf]
