# Supplementary material for: Sex- and age-specific reference intervals for diagnostic ratios reflecting relative activity of steroidogenic enzymes and pathways in adults
Source: PLoS One. 2021 Jul 8;16(7):e0253975. doi: 10.1371/journal.pone.0253975 (PMC8266106; doi:10.1371/journal.pone.0253975)
Supplement: S3 Fig — A descriptive analysis of 65 calculated ratios is shown including one ratio per page. Panel (a): boxplots, ratio (log-scale) by sex; Panel (b): Gasser-Müller nonparametric fit and scatter plot, ratio (log-scale) by age and by sex; Panel (c) boxplot, transformed ratio for men according to optimal power transformation, outliers are plotted as black dots; Panel (d) boxplot, transformed ratio for women according to optimal power transformation, outliers are plotted as black dots; Panel (e): ratio (log-scale) by day and night time for men; Panel (f): ratio (log-scale) by day and night time for women; Panel (g): Spearman rank correlation of transformed ratio night vs day for men; Panel (h): Spearman rank correlation of transformed steroid night vs day for women. Abbreviations used: M = men; W = women; D = day; N = night; TR = optimal power transformation; nout = number of outliers; ku = kurtosis; sk = skewness; delta = robust estimate of mean difference expressed in standard deviations; rho = Spearman rank correlation coefficient; n = sample size. (PDF) [file pone.0253975.s003.pdf]

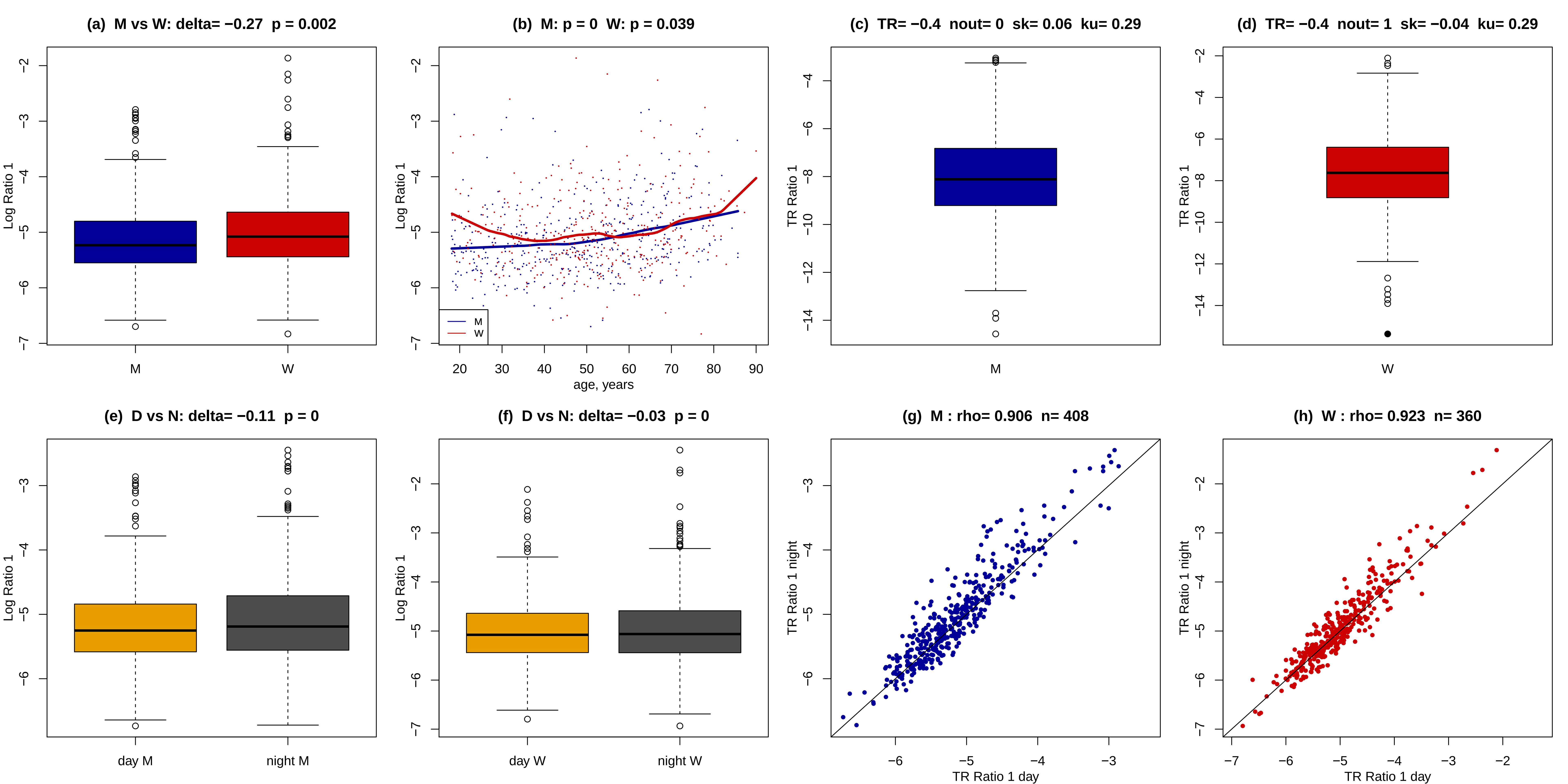
(a) M vs W: delta= −0.27 p = 0.002
(b) M: p = 0 W: p = 0.039
(c) TR= −0.4 nout= 0 sk= 0.06 ku= 0.29
(d) TR= −0.4 nout= 1 sk= −0.04 ku= 0.29
Log Ratio 1
M
W
age, years
TR Ratio 1
(e) D vs N: delta= −0.11 p = 0
(f) D vs N: delta= −0.03 p = 0
(g) M : rho= 0.906 n= 408
(h) W : rho= 0.923 n= 360
day M
night M
day W
night W
TR Ratio 1 night
TR Ratio 1 day

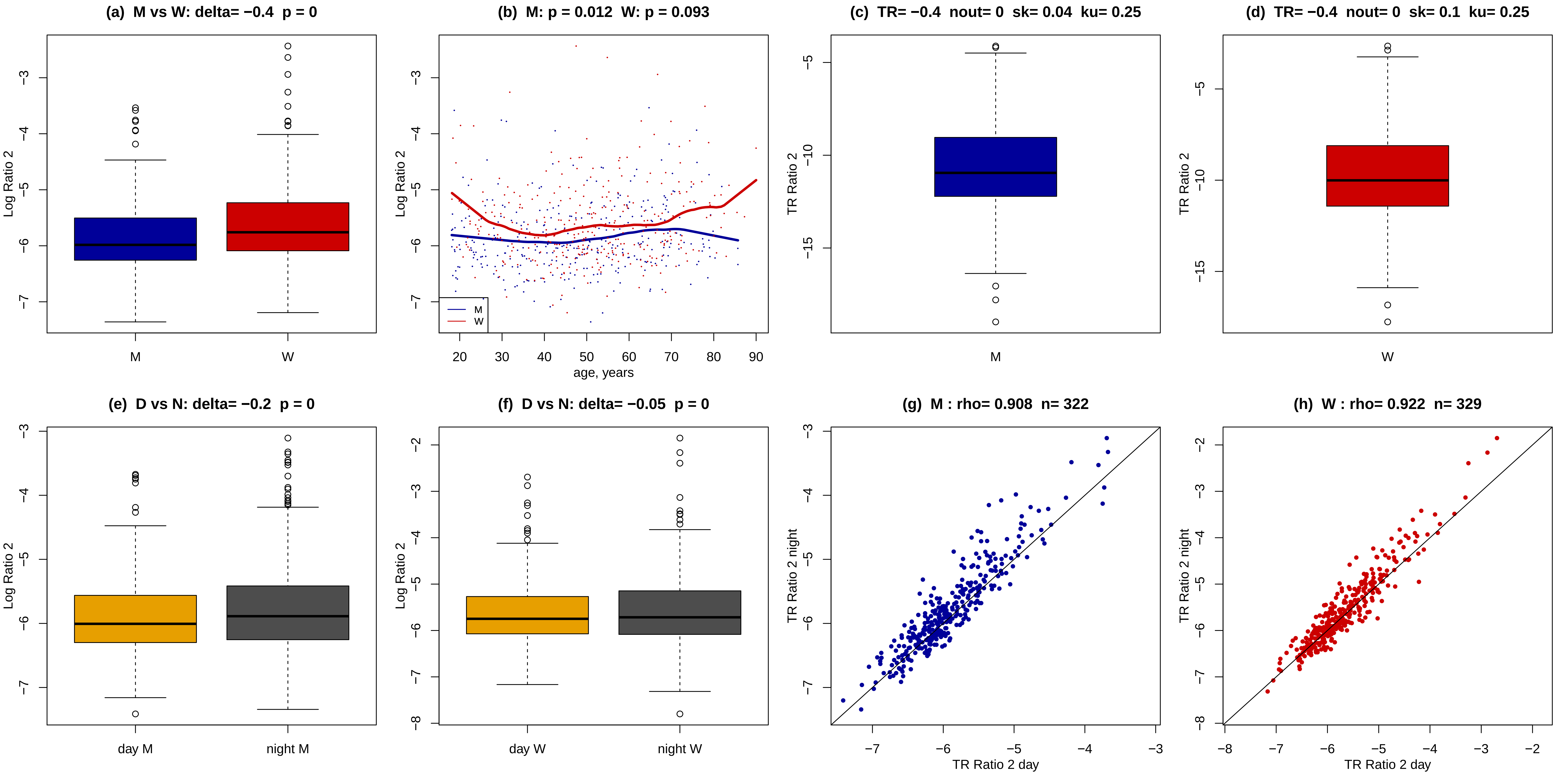

(a) M vs W: delta= −0.4 p = 0
(b) M: p = 0.012 W: p = 0.093
(c) TR= −0.4 nout= 0 sk= 0.04 ku= 0.25
(d) TR= −0.4 nout= 0 sk= 0.1 ku= 0.25
(e) D vs N: delta= −0.2 p = 0
(f) D vs N: delta= −0.05 p = 0
(g) M : rho= 0.908 n= 322
(h) W : rho= 0.922 n= 329
Log Ratio 2
TR Ratio 2
TR Ratio 2 night
TR Ratio 2 day
age, years
M
W
day M
night M
day W
night W

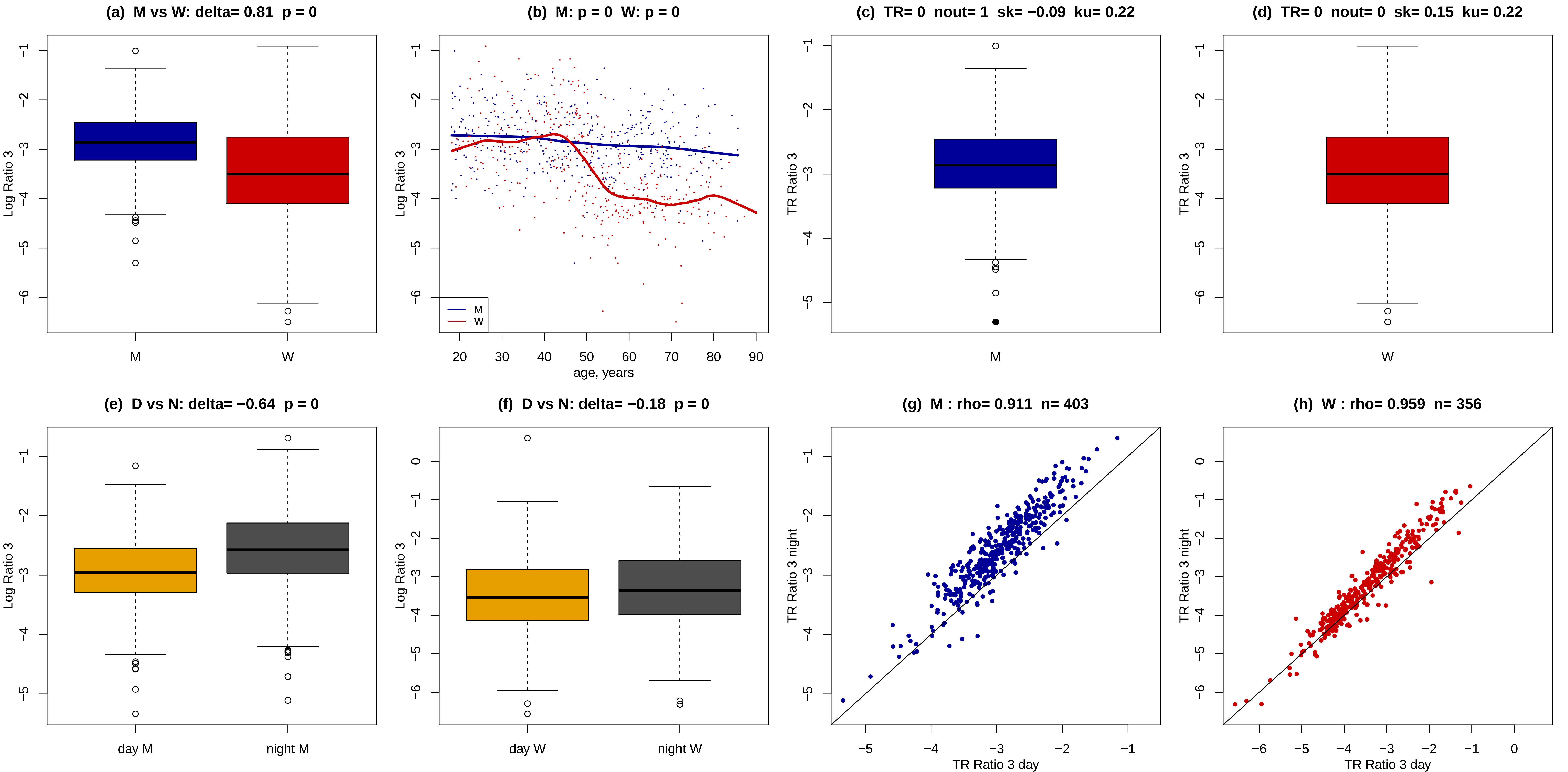

(a) M vs W: delta= 0.81 p = 0
(b) M: p = 0 W: p = 0
(c) TR= 0 nout= 1 sk= −0.09 ku= 0.22
(d) TR= 0 nout= 0 sk= 0.15 ku= 0.22
(e) D vs N: delta= −0.64 p = 0
(f) D vs N: delta= −0.18 p = 0
(g) M : rho= 0.911 n= 403
(h) W : rho= 0.959 n= 356
Log Ratio 3
TR Ratio 3
age, years
M
W
day M
night M
day W
night W
TR Ratio 3 night
TR Ratio 3 day

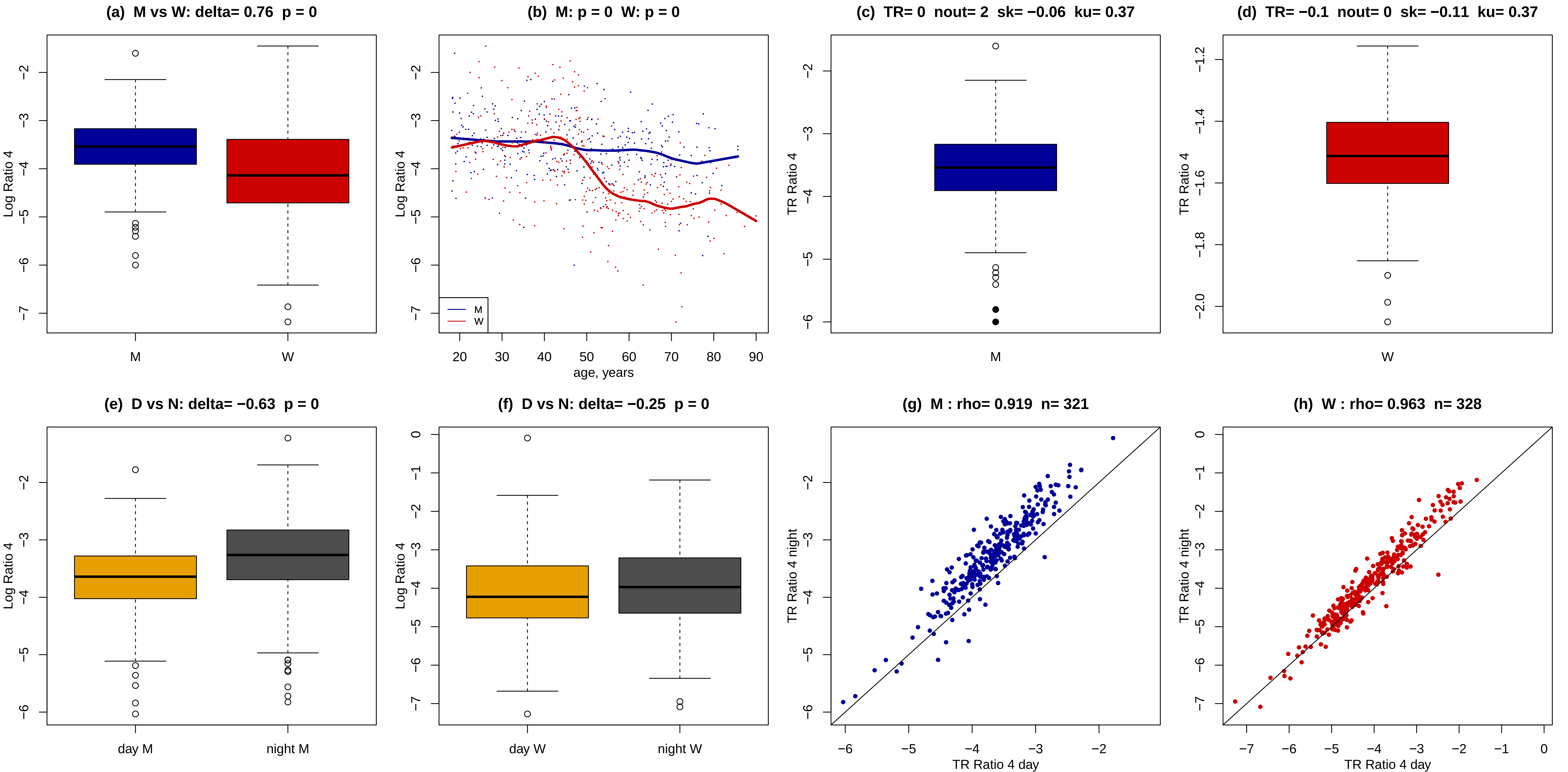
(a) M vs W: delta= 0.76 p = 0
Log Ratio 4
M
W
(b) M: p = 0 W: p = 0
Log Ratio 4
age, years
M
W
(c) TR= 0 nout= 2 sk= −0.06 ku= 0.37
TR Ratio 4
M
(d) TR= −0.1 nout= 0 sk= −0.11 ku= 0.37
TR Ratio 4
W
(e) D vs N: delta= −0.63 p = 0
Log Ratio 4
day M
night M
(f) D vs N: delta= −0.25 p = 0
Log Ratio 4
day W
night W
(g) M : rho= 0.919 n= 321
TR Ratio 4 night
TR Ratio 4 day
(h) W : rho= 0.963 n= 328
TR Ratio 4 night
TR Ratio 4 day

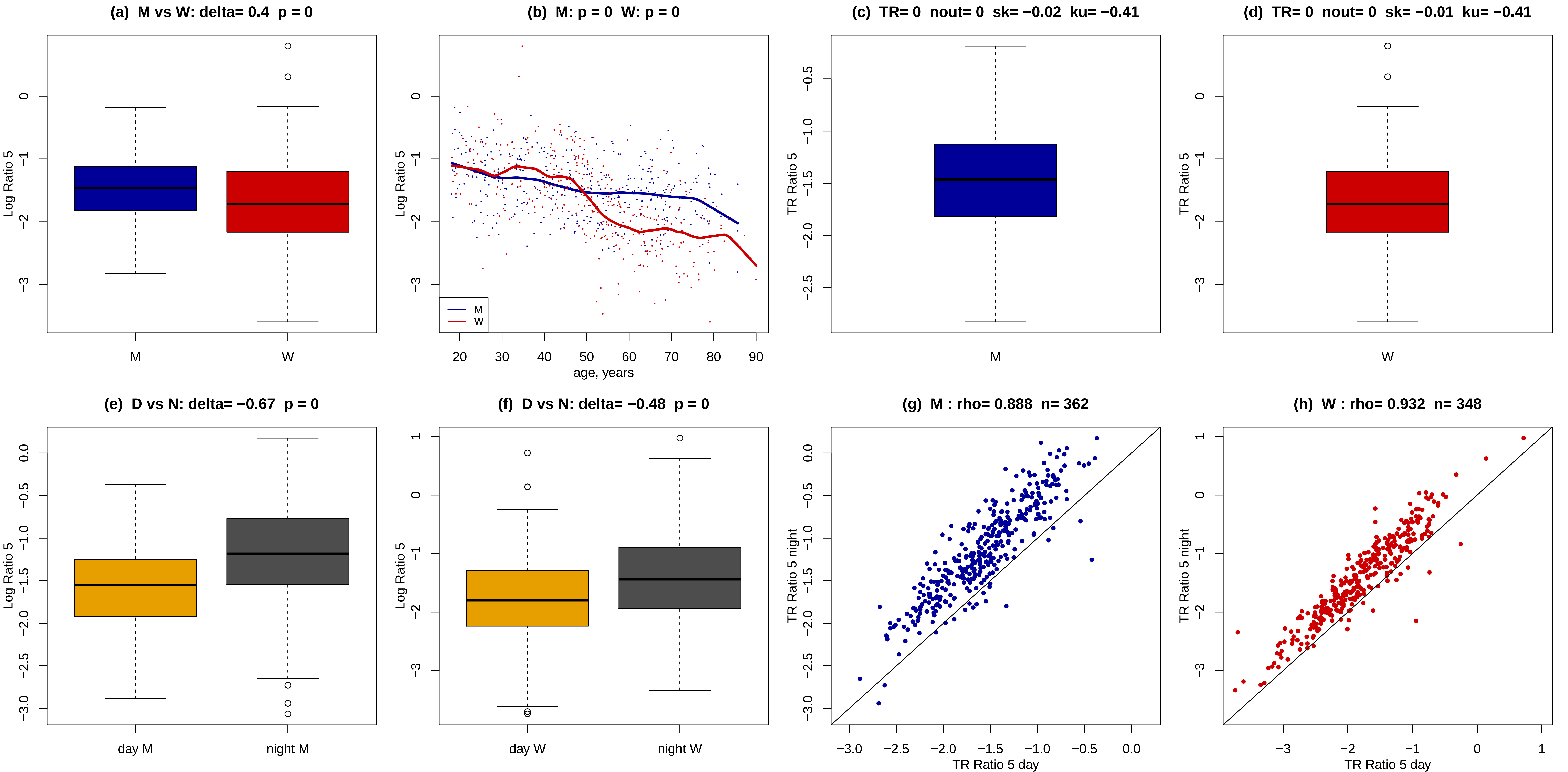

(a) M vs W: delta= 0.4 p = 0
(b) M: p = 0 W: p = 0
(c) TR= 0 nout= 0 sk= −0.02 ku= −0.41
(d) TR= 0 nout= 0 sk= −0.01 ku= −0.41
(e) D vs N: delta= −0.67 p = 0
(f) D vs N: delta= −0.48 p = 0
(g) M : rho= 0.888 n= 362
(h) W : rho= 0.932 n= 348
Log Ratio 5
TR Ratio 5
TR Ratio 5 night
TR Ratio 5 day
age, years
M
W
day M
night M
day W
night W

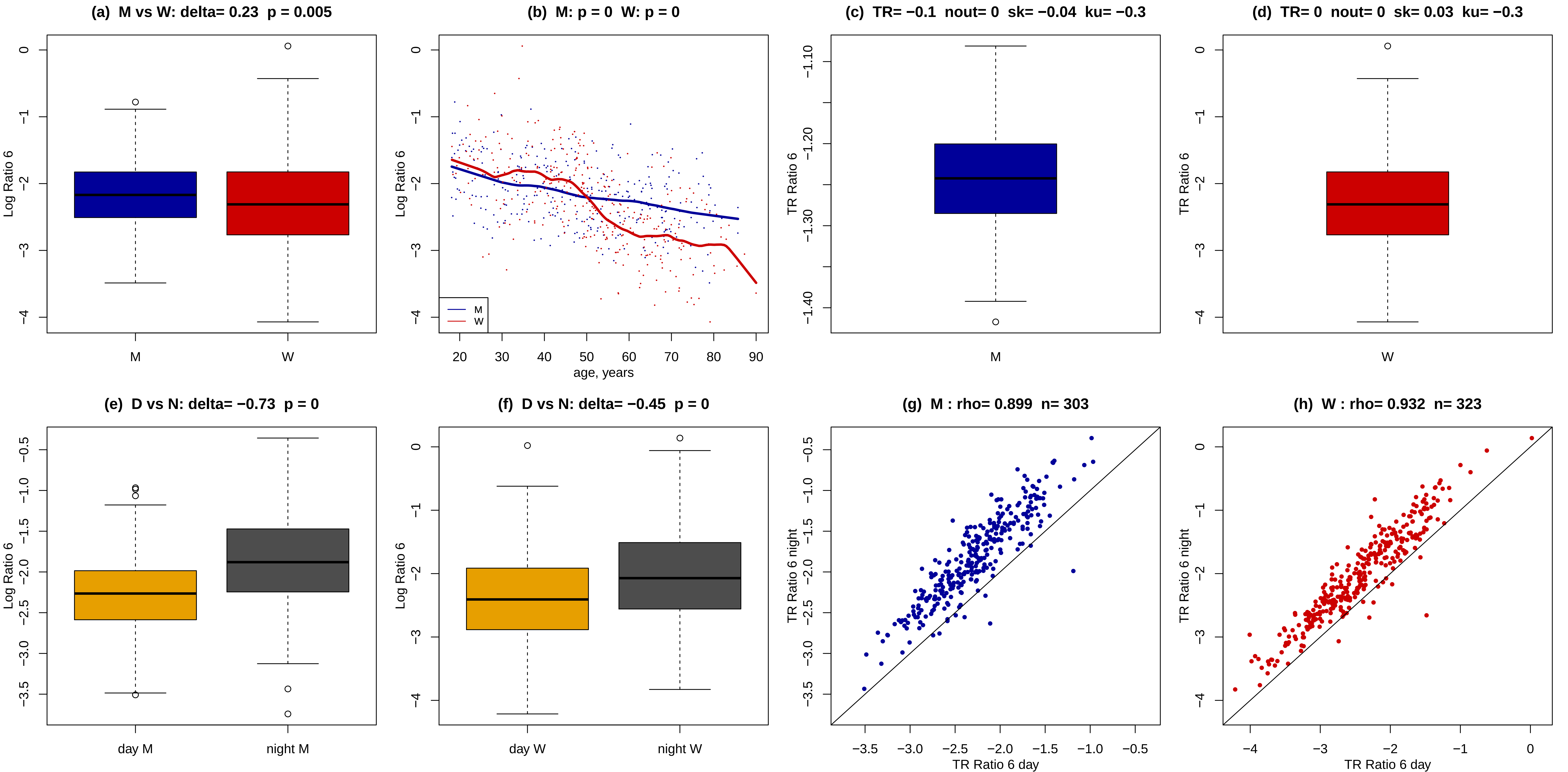

(a) M vs W: delta= 0.23 p = 0.005
(b) M: p = 0 W: p = 0
(c) TR= −0.1 nout= 0 sk= −0.04 ku= −0.3
(d) TR= 0 nout= 0 sk= 0.03 ku= −0.3
(e) D vs N: delta= −0.73 p = 0
(f) D vs N: delta= −0.45 p = 0
(g) M : rho= 0.899 n= 303
(h) W : rho= 0.932 n= 323
Log Ratio 6
TR Ratio 6
TR Ratio 6 night
TR Ratio 6 day
age, years
M
W
day M
night M
day W
night W

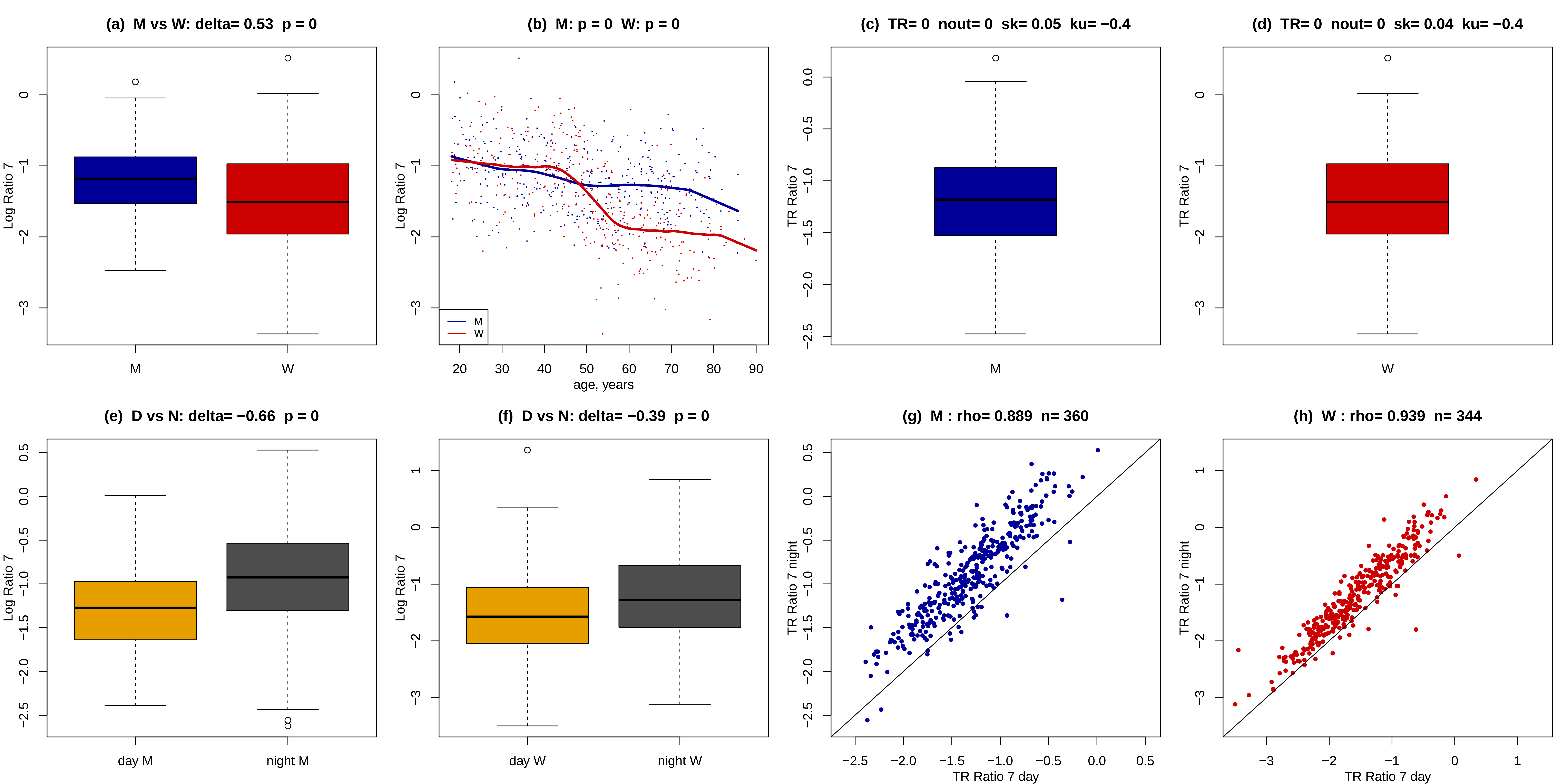

(a) M vs W: delta= 0.53 p = 0
(b) M: p = 0 W: p = 0
(c) TR= 0 nout= 0 sk= 0.05 ku= −0.4
(d) TR= 0 nout= 0 sk= 0.04 ku= −0.4
Log Ratio 7
TR Ratio 7
M
W
age, years
(e) D vs N: delta= −0.66 p = 0
(f) D vs N: delta= −0.39 p = 0
(g) M : rho= 0.889 n= 360
(h) W : rho= 0.939 n= 344
day M
night M
day W
night W
TR Ratio 7 night
TR Ratio 7 day

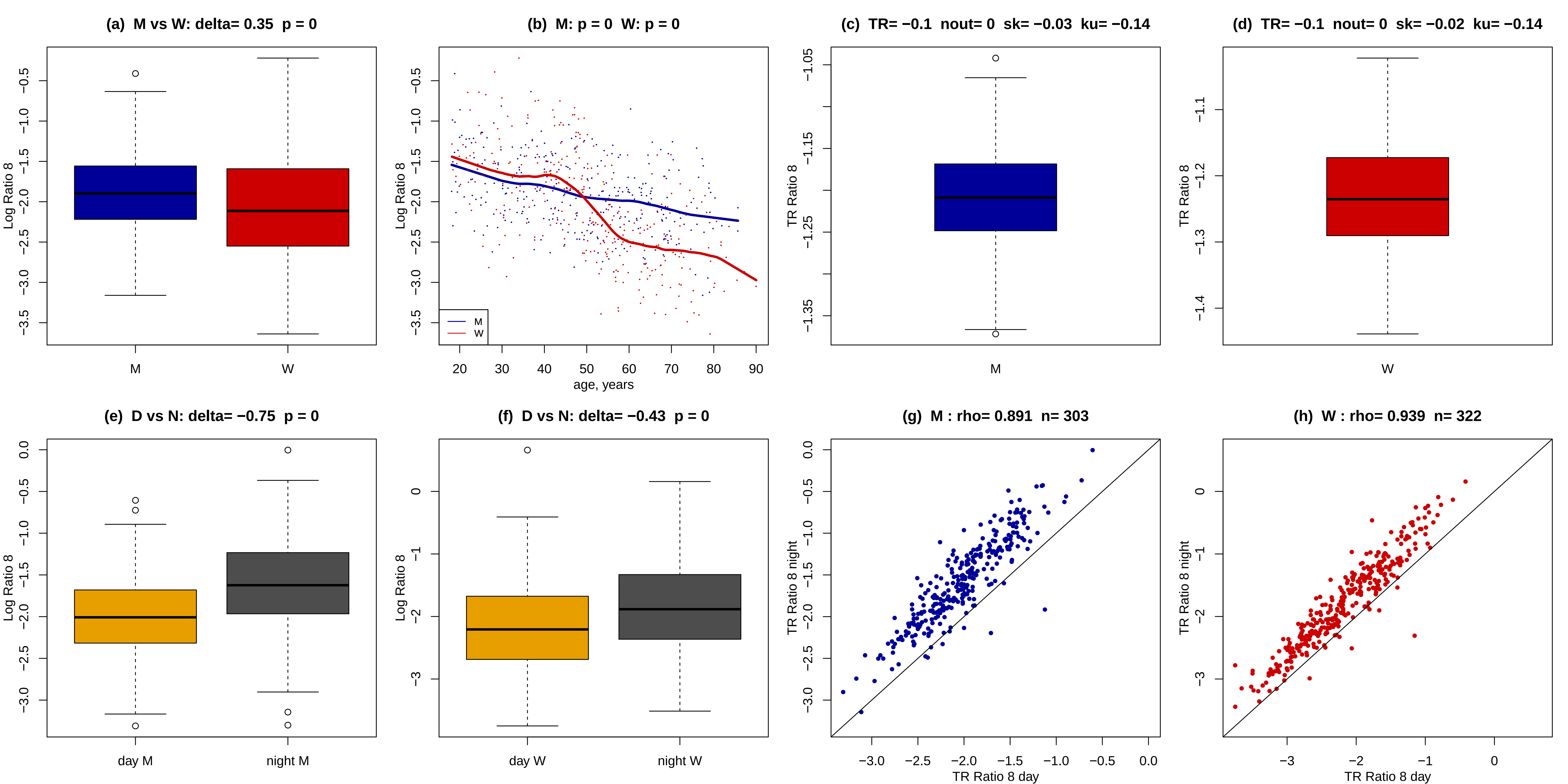

(a) M vs W: delta= 0.35 p = 0
(b) M: p = 0 W: p = 0
(c) TR= −0.1 nout= 0 sk= −0.03 ku= −0.14
(d) TR= −0.1 nout= 0 sk= −0.02 ku= −0.14
(e) D vs N: delta= −0.75 p = 0
(f) D vs N: delta= −0.43 p = 0
(g) M : rho= 0.891 n= 303
(h) W : rho= 0.939 n= 322
Log Ratio 8
TR Ratio 8
TR Ratio 8 night
TR Ratio 8 day
age, years
M
W
day M
night M
day W
night W

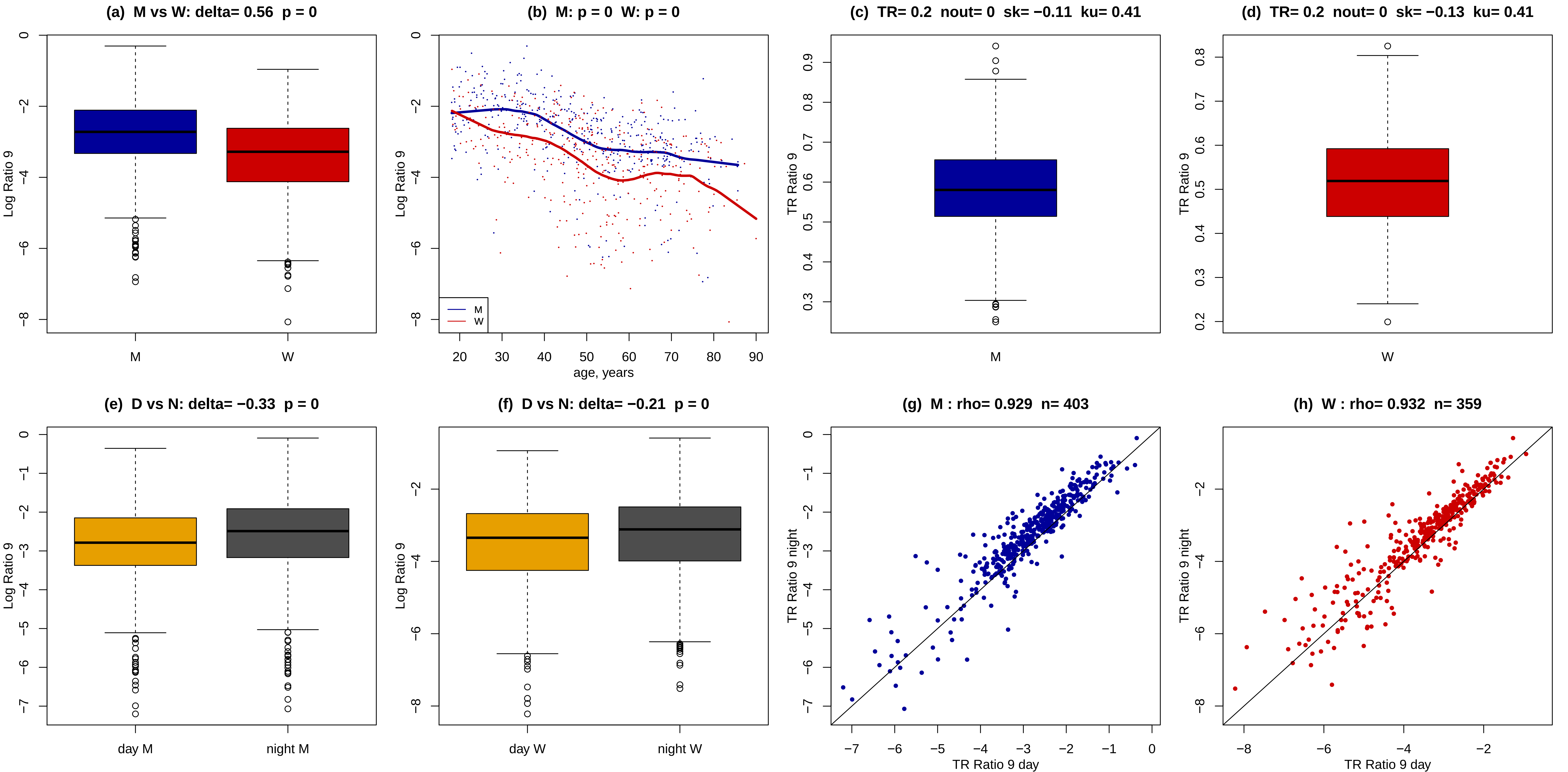

(a) M vs W: delta= 0.56 p = 0
Log Ratio 9
M
W
(b) M: p = 0 W: p = 0
Log Ratio 9
age, years
M
W
(c) TR= 0.2 nout= 0 sk= −0.11 ku= 0.41
TR Ratio 9
M
(d) TR= 0.2 nout= 0 sk= −0.13 ku= 0.41
TR Ratio 9
W
(e) D vs N: delta= −0.33 p = 0
Log Ratio 9
day M
night M
(f) D vs N: delta= −0.21 p = 0
Log Ratio 9
day W
night W
(g) M : rho= 0.929 n= 403
TR Ratio 9 night
TR Ratio 9 day
(h) W : rho= 0.932 n= 359
TR Ratio 9 night
TR Ratio 9 day

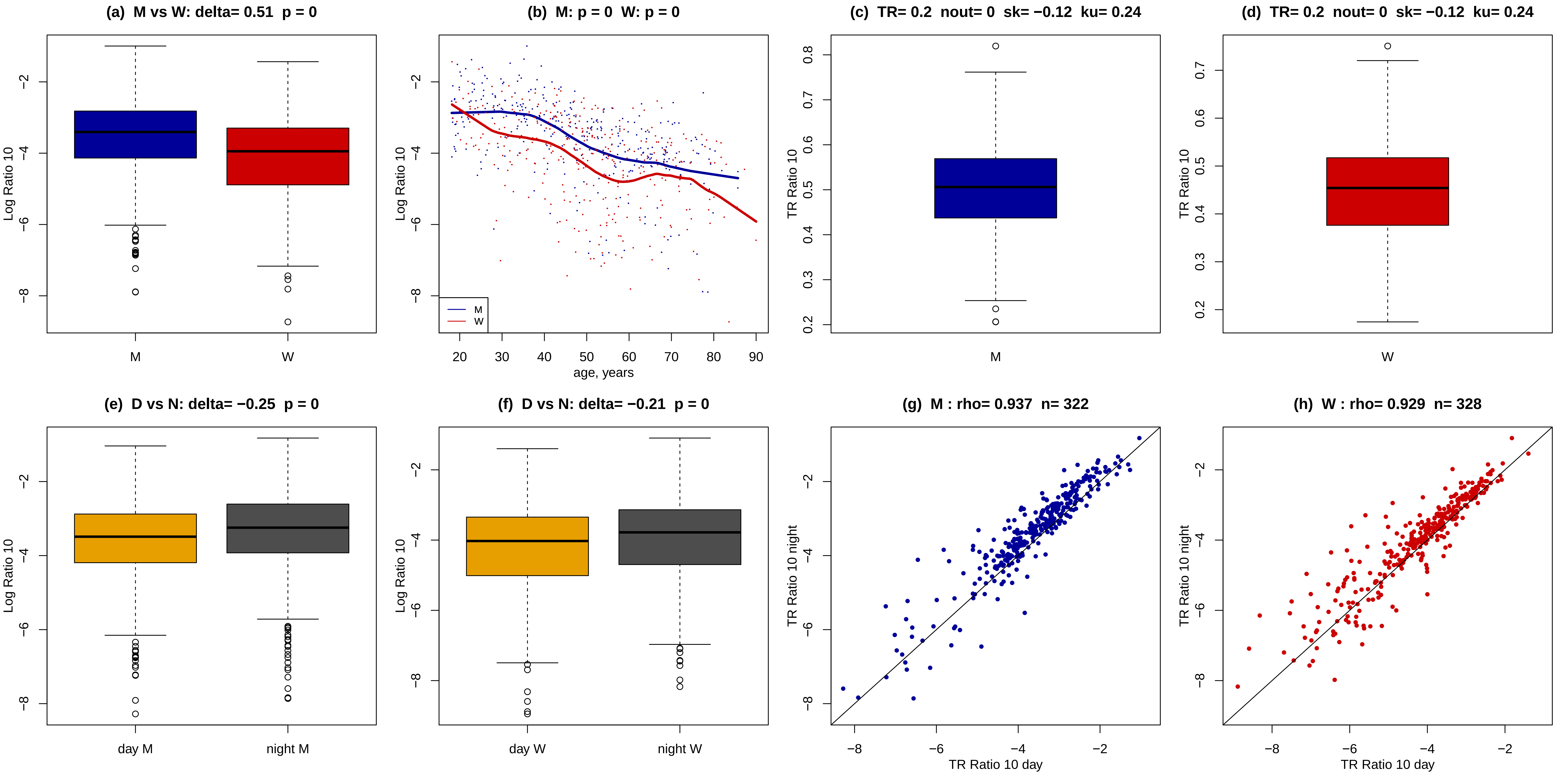

(a) M vs W: delta= 0.51 p = 0
Log Ratio 10
M
W
(b) M: p = 0 W: p = 0
Log Ratio 10
age, years
M
W
(c) TR= 0.2 nout= 0 sk= −0.12 ku= 0.24
TR Ratio 10
M
(d) TR= 0.2 nout= 0 sk= −0.12 ku= 0.24
TR Ratio 10
W
(e) D vs N: delta= −0.25 p = 0
Log Ratio 10
day M
night M
(f) D vs N: delta= −0.21 p = 0
Log Ratio 10
day W
night W
(g) M : rho= 0.937 n= 322
TR Ratio 10 night
TR Ratio 10 day
(h) W : rho= 0.929 n= 328
TR Ratio 10 night
TR Ratio 10 day

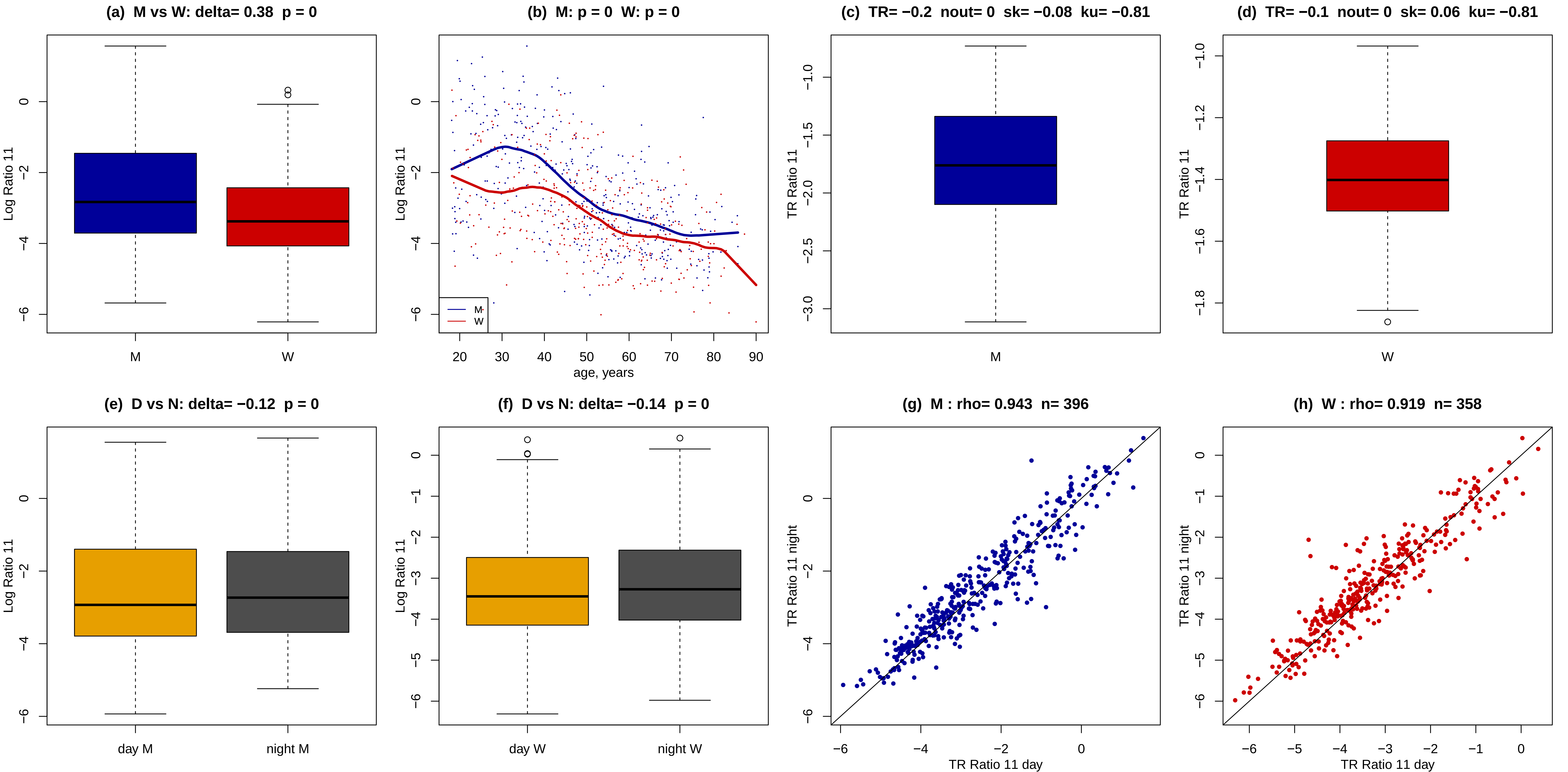
(a) M vs W: delta= 0.38 p = 0
(b) M: p = 0 W: p = 0
(c) TR= −0.2 nout= 0 sk= −0.08 ku= −0.81
(d) TR= −0.1 nout= 0 sk= 0.06 ku= −0.81
(e) D vs N: delta= −0.12 p = 0
(f) D vs N: delta= −0.14 p = 0
(g) M : rho= 0.943 n= 396
(h) W : rho= 0.919 n= 358
Log Ratio 11
TR Ratio 11
age, years
M
W
day M
night M
day W
night W
TR Ratio 11 night
TR Ratio 11 day

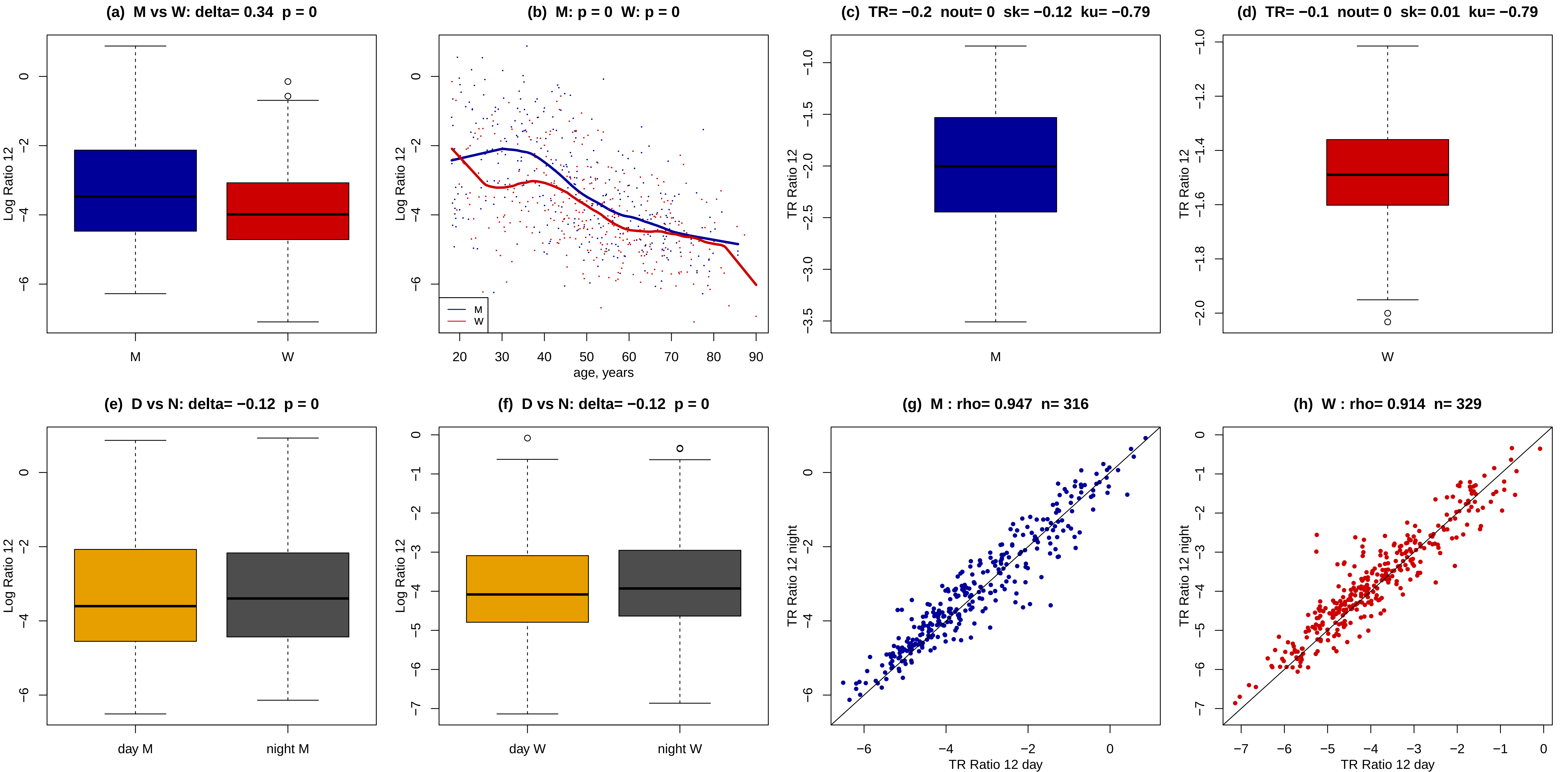
(a) M vs W: delta= 0.34 p = 0
(b) M: p = 0 W: p = 0
(c) TR= −0.2 nout= 0 sk= −0.12 ku= −0.79
(d) TR= −0.1 nout= 0 sk= 0.01 ku= −0.79
(e) D vs N: delta= −0.12 p = 0
(f) D vs N: delta= −0.12 p = 0
(g) M : rho= 0.947 n= 316
(h) W : rho= 0.914 n= 329
Log Ratio 12
TR Ratio 12
age, years
M
W
day M
night M
day W
night W
TR Ratio 12 night
TR Ratio 12 day

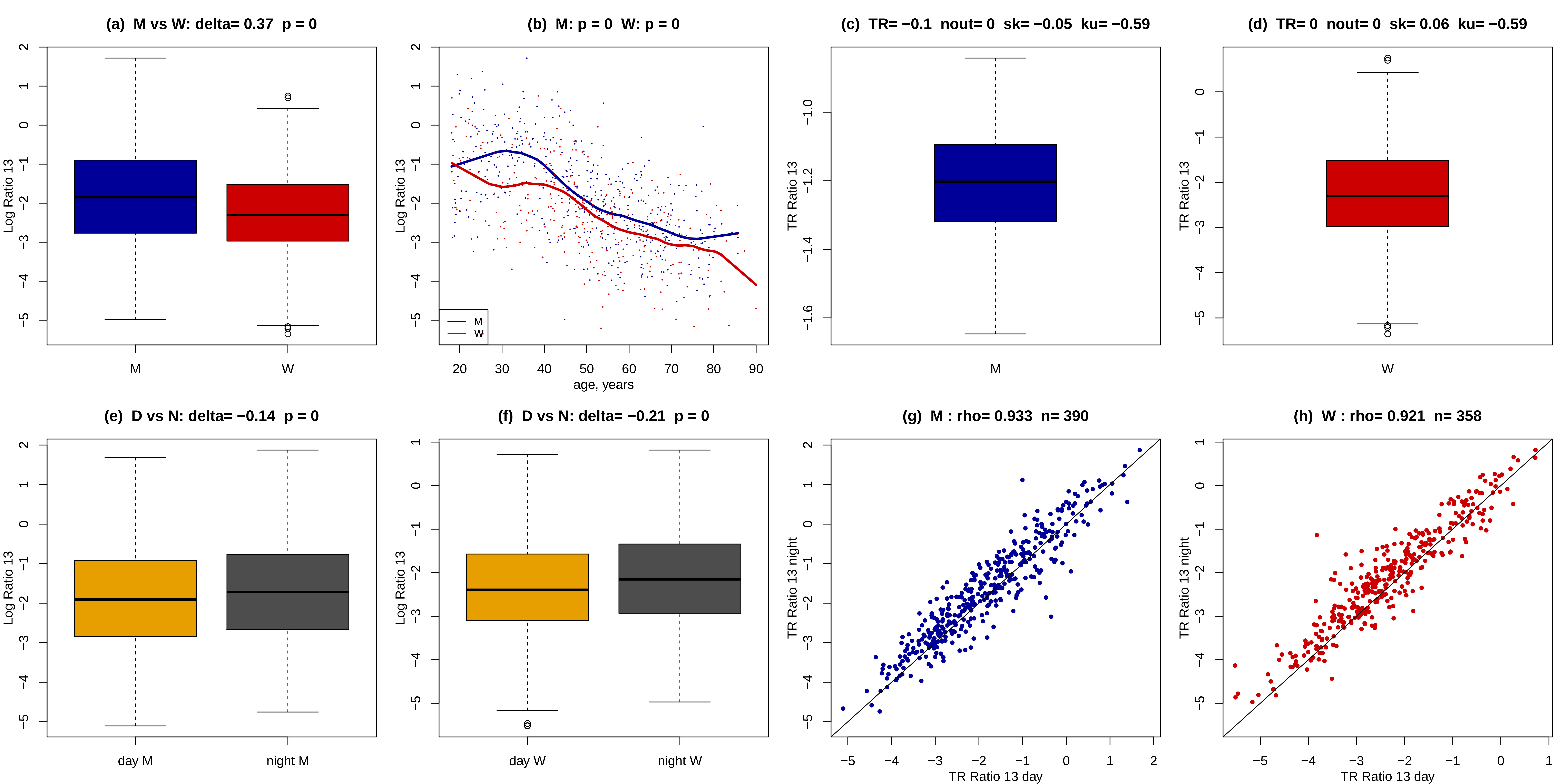

(a) M vs W: delta= 0.37 p = 0
Log Ratio 13
M
W
(b) M: p = 0 W: p = 0
Log Ratio 13
age, years
M
W
(c) TR= −0.1 nout= 0 sk= −0.05 ku= −0.59
TR Ratio 13
M
(d) TR= 0 nout= 0 sk= 0.06 ku= −0.59
TR Ratio 13
W
(e) D vs N: delta= −0.14 p = 0
Log Ratio 13
day M
night M
(f) D vs N: delta= −0.21 p = 0
Log Ratio 13
day W
night W
(g) M : rho= 0.933 n= 390
TR Ratio 13 night
TR Ratio 13 day
(h) W : rho= 0.921 n= 358
TR Ratio 13 night
TR Ratio 13 day

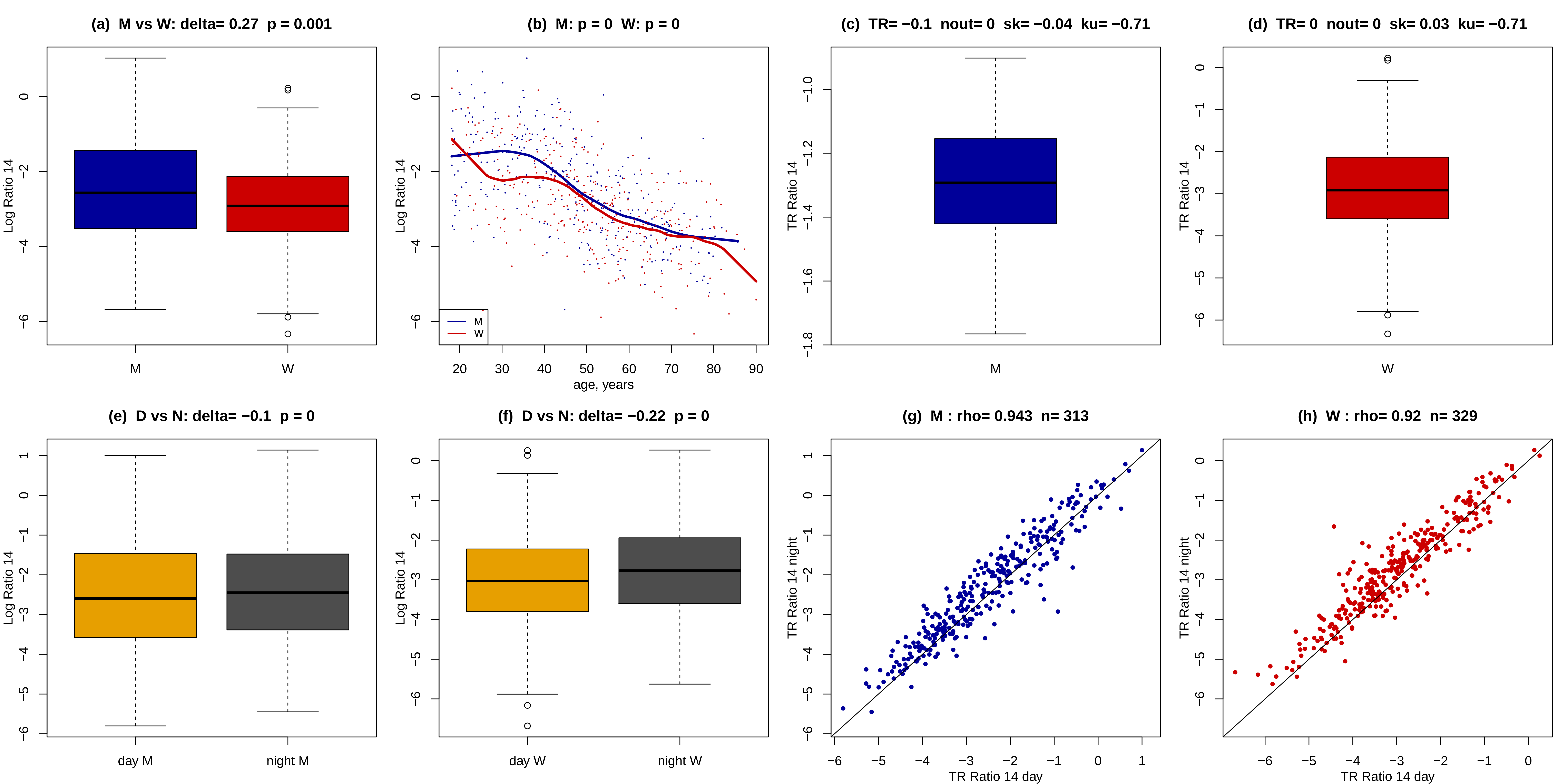
(a) M vs W: delta= 0.27 p = 0.001
Log Ratio 14
M
W
(b) M: p = 0 W: p = 0
Log Ratio 14
age, years
M
W
(c) TR= −0.1 nout= 0 sk= −0.04 ku= −0.71
TR Ratio 14
M
(d) TR= 0 nout= 0 sk= 0.03 ku= −0.71
TR Ratio 14
W
(e) D vs N: delta= −0.1 p = 0
Log Ratio 14
day M
night M
(f) D vs N: delta= −0.22 p = 0
Log Ratio 14
day W
night W
(g) M : rho= 0.943 n= 313
TR Ratio 14 night
TR Ratio 14 day
(h) W : rho= 0.92 n= 329
TR Ratio 14 night
TR Ratio 14 day

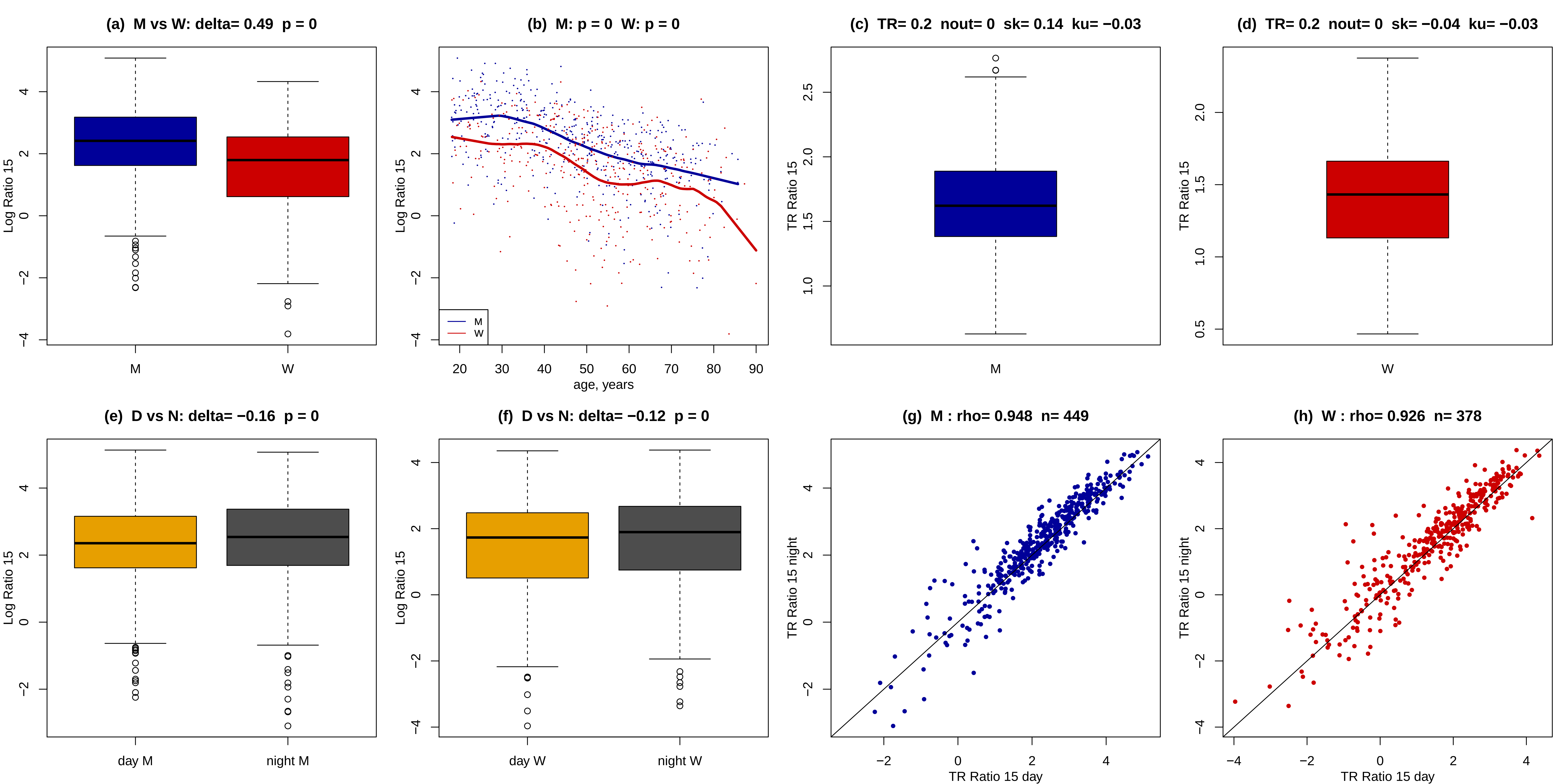
(a) M vs W: delta= 0.49 p = 0
Log Ratio 15
M
W
(b) M: p = 0 W: p = 0
Log Ratio 15
age, years
M
W
(c) TR= 0.2 nout= 0 sk= 0.14 ku= −0.03
TR Ratio 15
M
(d) TR= 0.2 nout= 0 sk= −0.04 ku= −0.03
TR Ratio 15
W
(e) D vs N: delta= −0.16 p = 0
Log Ratio 15
day M
night M
(f) D vs N: delta= −0.12 p = 0
Log Ratio 15
day W
night W
(g) M : rho= 0.948 n= 449
TR Ratio 15 night
TR Ratio 15 day
(h) W : rho= 0.926 n= 378
TR Ratio 15 night
TR Ratio 15 day

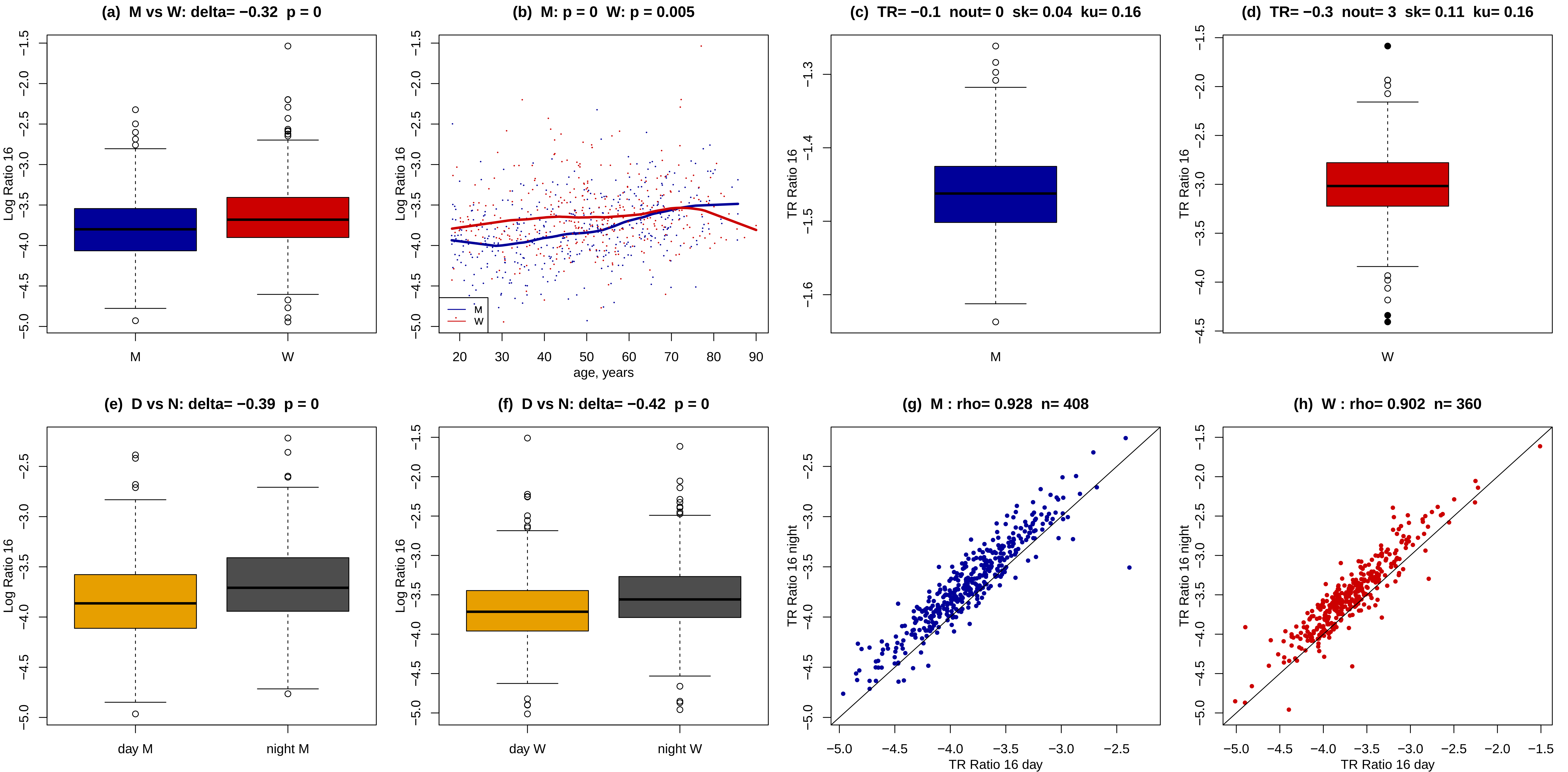
(a) M vs W: delta= −0.32 p = 0
Log Ratio 16
M
W
(b) M: p = 0 W: p = 0.005
Log Ratio 16
age, years
M
W
(c) TR= −0.1 nout= 0 sk= 0.04 ku= 0.16
TR Ratio 16
M
(d) TR= −0.3 nout= 3 sk= 0.11 ku= 0.16
TR Ratio 16
W
(e) D vs N: delta= −0.39 p = 0
Log Ratio 16
day M
night M
(f) D vs N: delta= −0.42 p = 0
Log Ratio 16
day W
night W
(g) M : rho= 0.928 n= 408
TR Ratio 16 night
TR Ratio 16 day
(h) W : rho= 0.902 n= 360
TR Ratio 16 night
TR Ratio 16 day

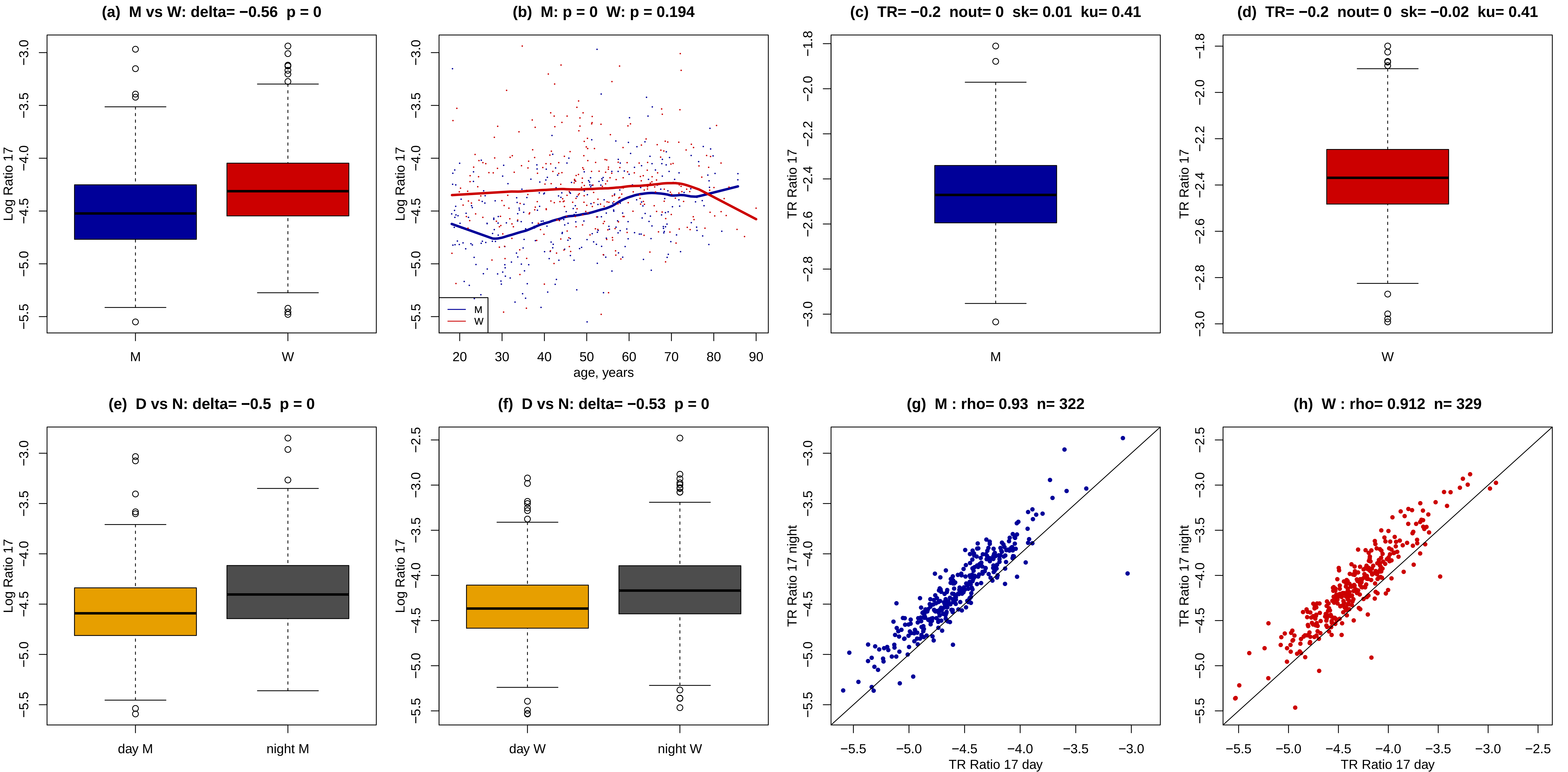

(a) M vs W: delta= −0.56 p = 0
Log Ratio 17
M
W
(b) M: p = 0 W: p = 0.194
Log Ratio 17
age, years
M
W
(c) TR= −0.2 nout= 0 sk= 0.01 ku= 0.41
TR Ratio 17
M
(d) TR= −0.2 nout= 0 sk= −0.02 ku= 0.41
TR Ratio 17
W
(e) D vs N: delta= −0.5 p = 0
Log Ratio 17
day M
night M
(f) D vs N: delta= −0.53 p = 0
Log Ratio 17
day W
night W
(g) M : rho= 0.93 n= 322
TR Ratio 17 night
TR Ratio 17 day
(h) W : rho= 0.912 n= 329
TR Ratio 17 night
TR Ratio 17 day

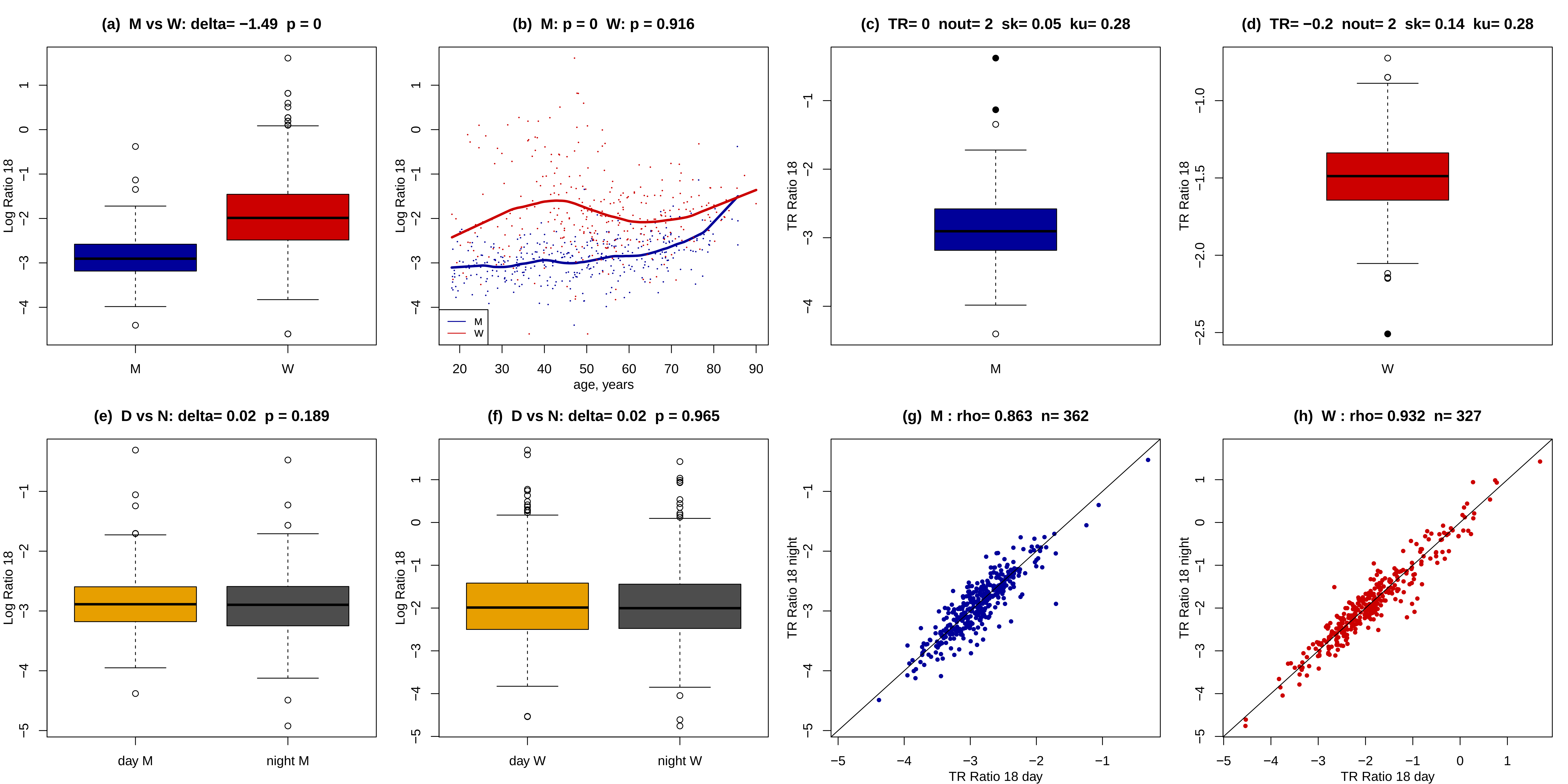

(a) M vs W: delta= −1.49 p = 0
(b) M: p = 0 W: p = 0.916
(c) TR= 0 nout= 2 sk= 0.05 ku= 0.28
(d) TR= −0.2 nout= 2 sk= 0.14 ku= 0.28
Log Ratio 18
TR Ratio 18
M
W
age, years
(e) D vs N: delta= 0.02 p = 0.189
(f) D vs N: delta= 0.02 p = 0.965
(g) M : rho= 0.863 n= 362
(h) W : rho= 0.932 n= 327
day M
night M
day W
night W
TR Ratio 18 night
TR Ratio 18 day

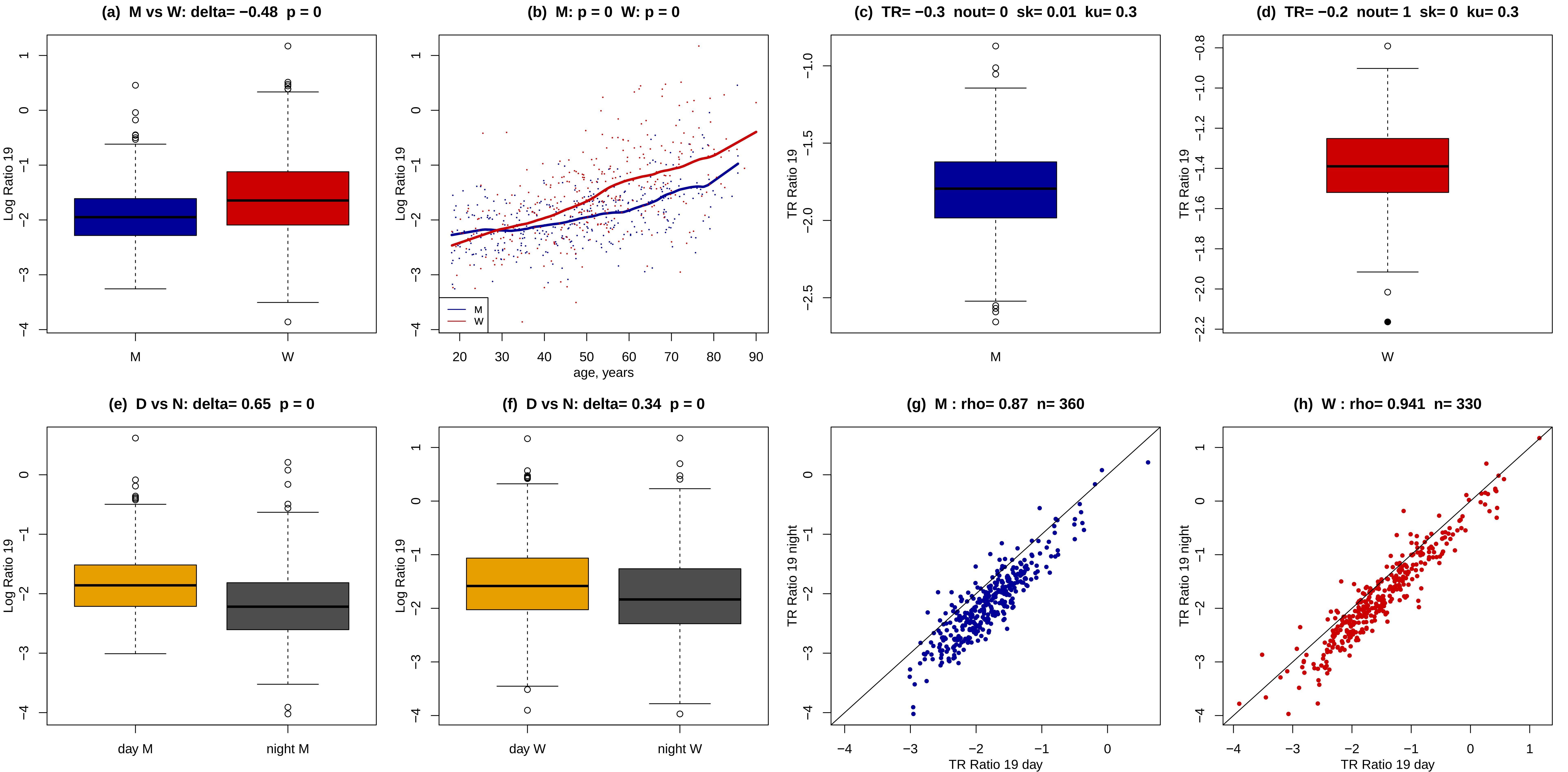

(a) M vs W: delta= −0.48 p = 0
Log Ratio 19
M
W
(b) M: p = 0 W: p = 0
Log Ratio 19
age, years
M
W
(c) TR= −0.3 nout= 0 sk= 0.01 ku= 0.3
TR Ratio 19
M
(d) TR= −0.2 nout= 1 sk= 0 ku= 0.3
TR Ratio 19
W
(e) D vs N: delta= 0.65 p = 0
Log Ratio 19
day M
night M
(f) D vs N: delta= 0.34 p = 0
Log Ratio 19
day W
night W
(g) M : rho= 0.87 n= 360
TR Ratio 19 night
TR Ratio 19 day
(h) W : rho= 0.941 n= 330
TR Ratio 19 night
TR Ratio 19 day

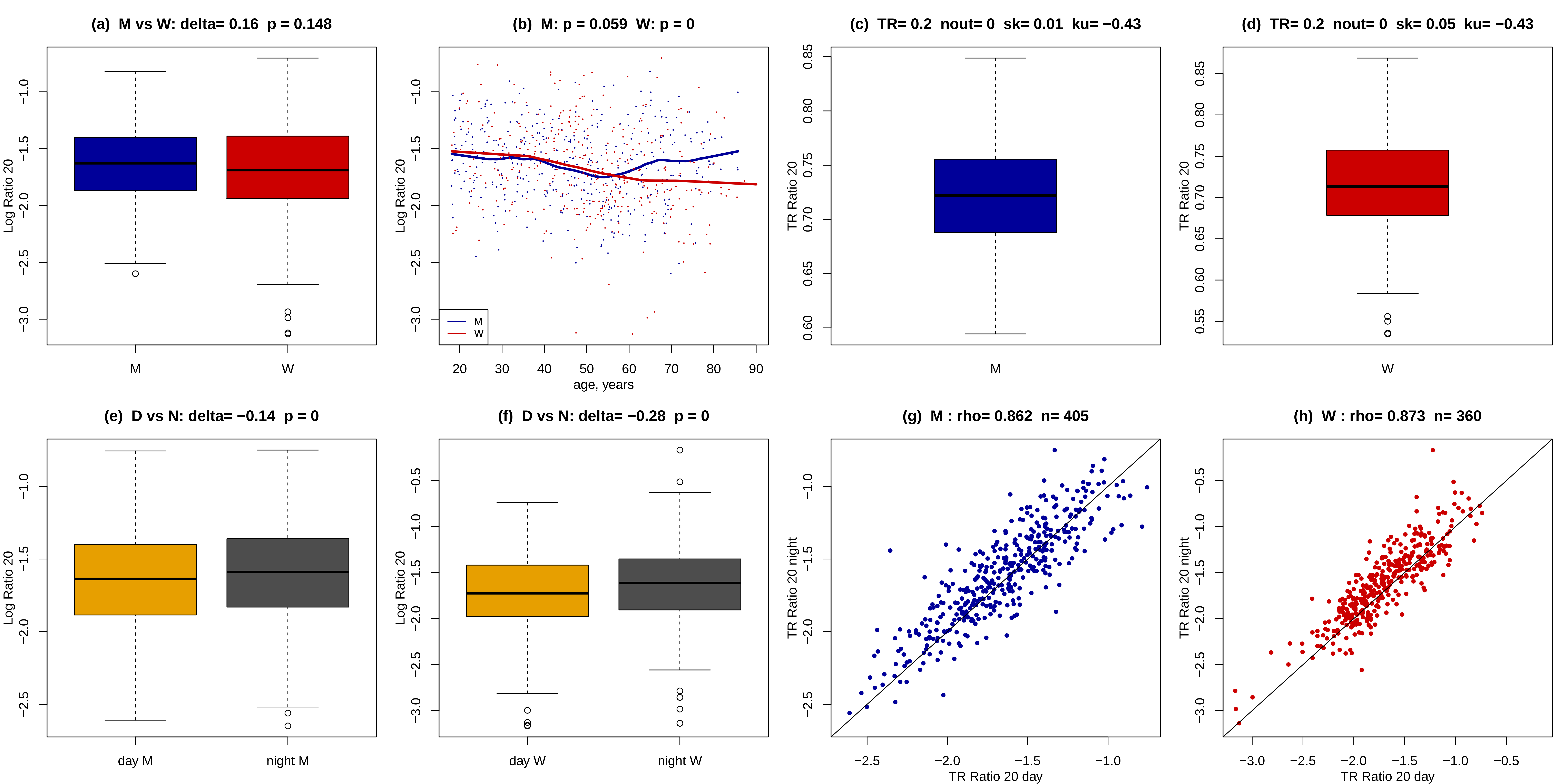

(a) M vs W: delta= 0.16 p = 0.148
Log Ratio 20
M
W
(b) M: p = 0.059 W: p = 0
Log Ratio 20
M
W
age, years
(c) TR= 0.2 nout= 0 sk= 0.01 ku= −0.43
TR Ratio 20
M
(d) TR= 0.2 nout= 0 sk= 0.05 ku= −0.43
TR Ratio 20
W
(e) D vs N: delta= −0.14 p = 0
Log Ratio 20
day M
night M
(f) D vs N: delta= −0.28 p = 0
Log Ratio 20
day W
night W
(g) M : rho= 0.862 n= 405
TR Ratio 20 night
TR Ratio 20 day
(h) W : rho= 0.873 n= 360
TR Ratio 20 night
TR Ratio 20 day

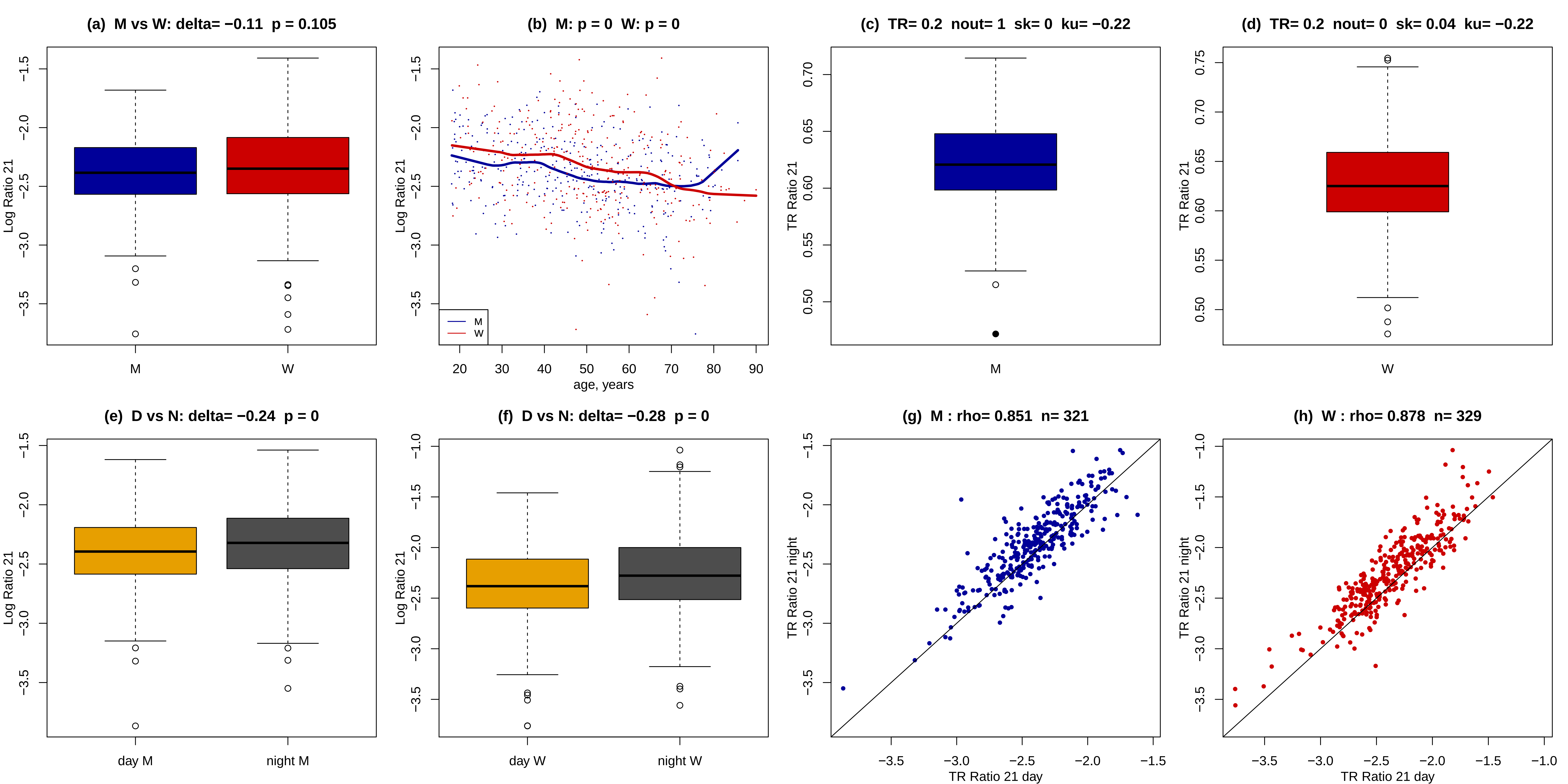

(a) M vs W: delta= −0.11 p = 0.105
(b) M: p = 0 W: p = 0
(c) TR= 0.2 nout= 1 sk= 0 ku= −0.22
(d) TR= 0.2 nout= 0 sk= 0.04 ku= −0.22
(e) D vs N: delta= −0.24 p = 0
(f) D vs N: delta= −0.28 p = 0
(g) M : rho= 0.851 n= 321
(h) W : rho= 0.878 n= 329
Log Ratio 21
TR Ratio 21
TR Ratio 21 night
TR Ratio 21 day
age, years
M
W
day M
night M
day W
night W

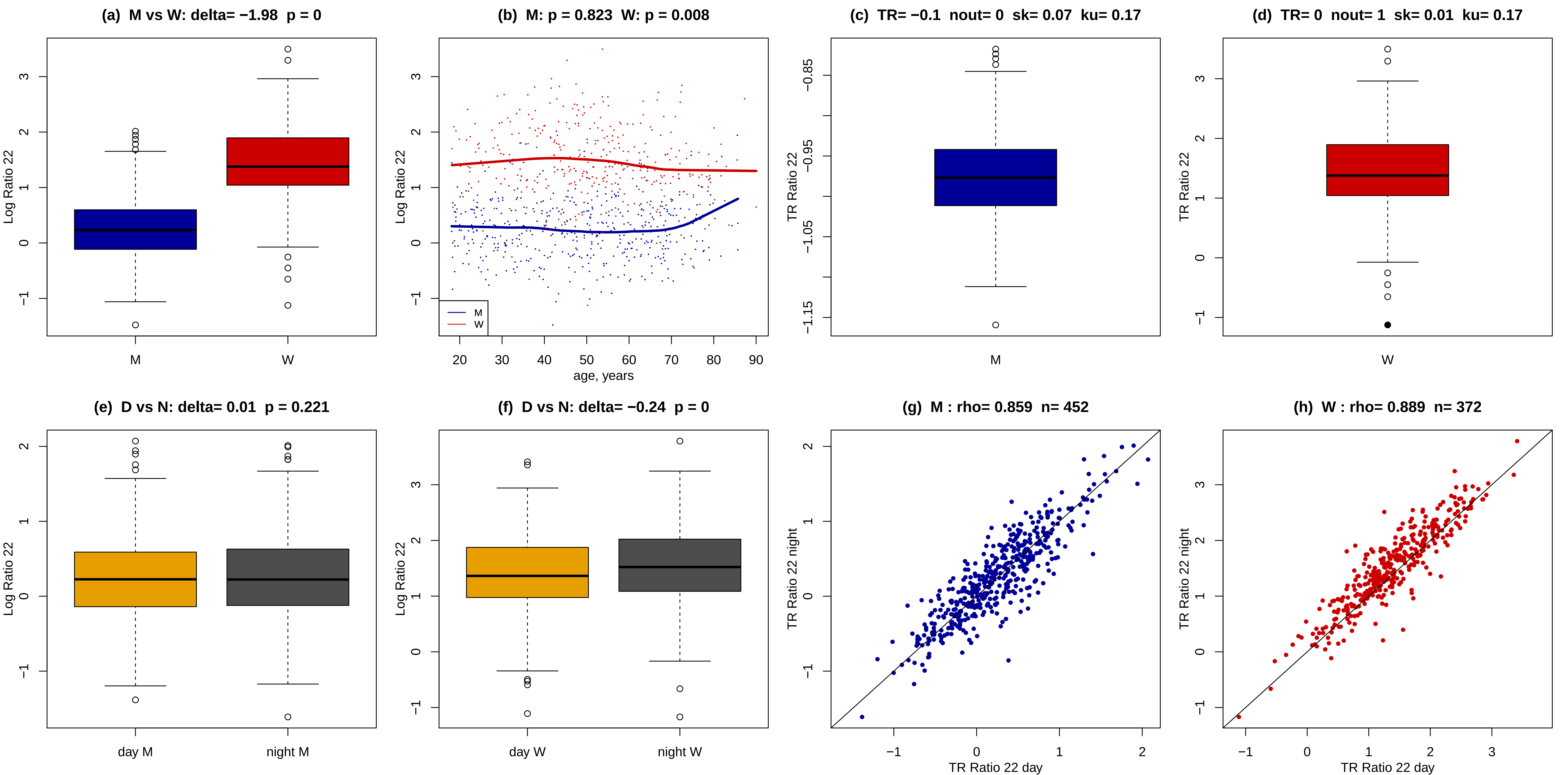
(a) M vs W: delta= −1.98 p = 0
Log Ratio 22
M
W
(b) M: p = 0.823 W: p = 0.008
Log Ratio 22
age, years
M
W
(c) TR= −0.1 nout= 0 sk= 0.07 ku= 0.17
TR Ratio 22
M
(d) TR= 0 nout= 1 sk= 0.01 ku= 0.17
TR Ratio 22
W
(e) D vs N: delta= 0.01 p = 0.221
Log Ratio 22
day M
night M
(f) D vs N: delta= −0.24 p = 0
Log Ratio 22
day W
night W
(g) M : rho= 0.859 n= 452
TR Ratio 22 night
TR Ratio 22 day
(h) W : rho= 0.889 n= 372
TR Ratio 22 night
TR Ratio 22 day

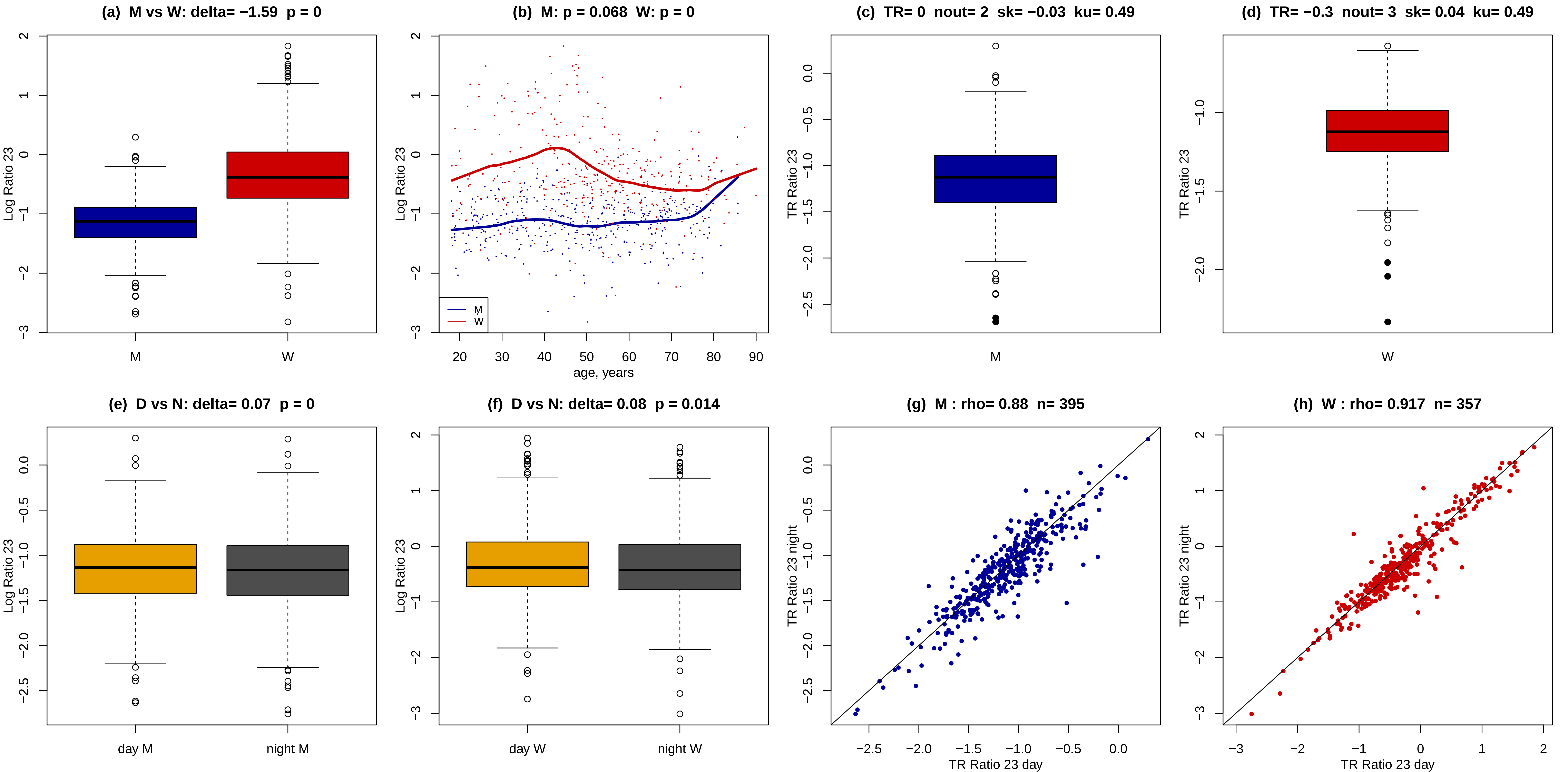
(a) M vs W: delta= −1.59 p = 0
(b) M: p = 0.068 W: p = 0
(c) TR= 0 nout= 2 sk= −0.03 ku= 0.49
(d) TR= −0.3 nout= 3 sk= 0.04 ku= 0.49
(e) D vs N: delta= 0.07 p = 0
(f) D vs N: delta= 0.08 p = 0.014
(g) M : rho= 0.88 n= 395
(h) W : rho= 0.917 n= 357
Log Ratio 23
TR Ratio 23
age, years
M
W
day M
night M
day W
night W
TR Ratio 23 night
TR Ratio 23 day

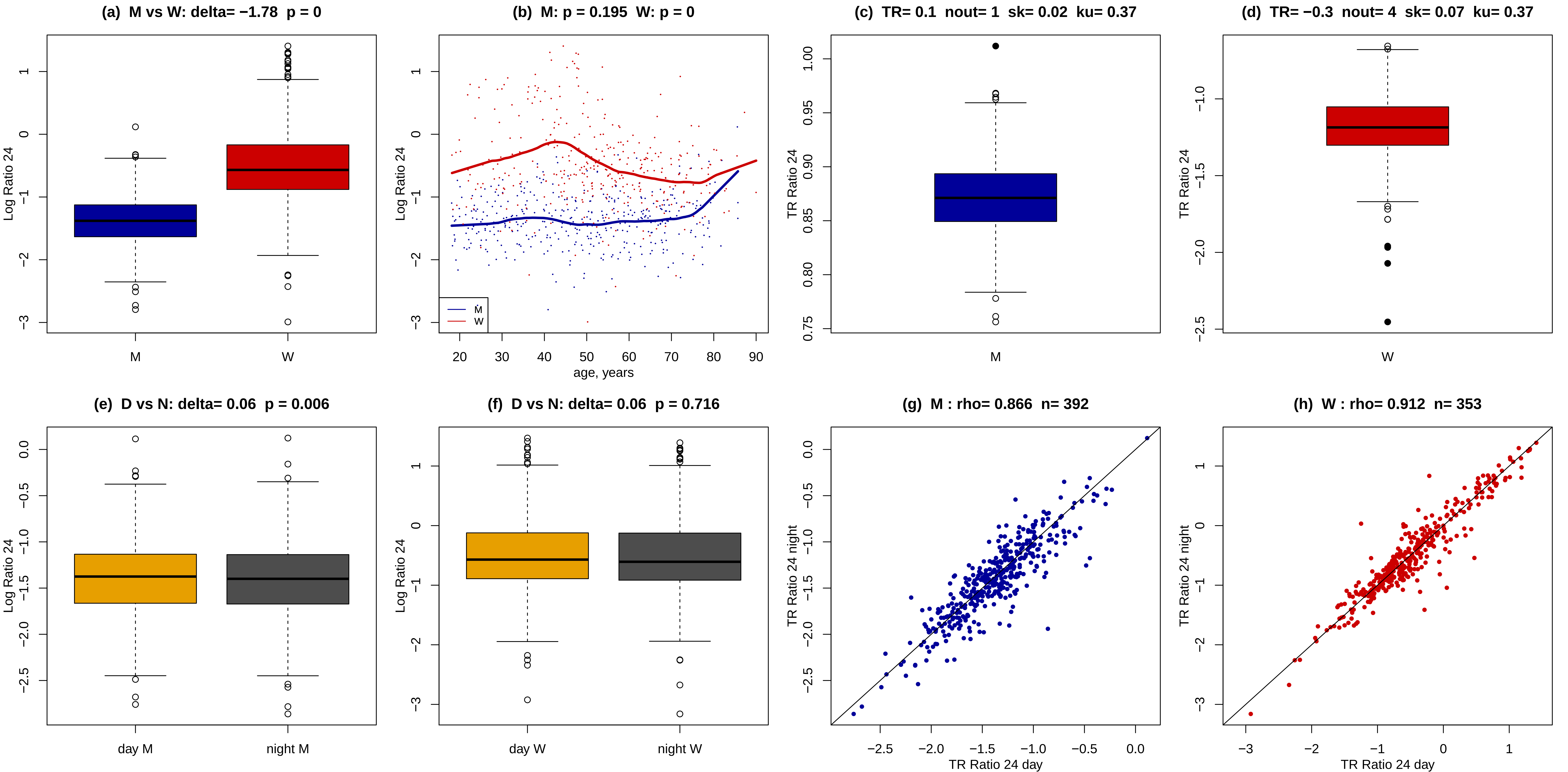

(a) M vs W: delta= −1.78 p = 0
Log Ratio 24
M
W
(b) M: p = 0.195 W: p = 0
Log Ratio 24
age, years
M
W
(c) TR= 0.1 nout= 1 sk= 0.02 ku= 0.37
TR Ratio 24
M
(d) TR= −0.3 nout= 4 sk= 0.07 ku= 0.37
TR Ratio 24
W
(e) D vs N: delta= 0.06 p = 0.006
Log Ratio 24
day M
night M
(f) D vs N: delta= 0.06 p = 0.716
Log Ratio 24
day W
night W
(g) M : rho= 0.866 n= 392
TR Ratio 24 night
TR Ratio 24 day
(h) W : rho= 0.912 n= 353
TR Ratio 24 night
TR Ratio 24 day

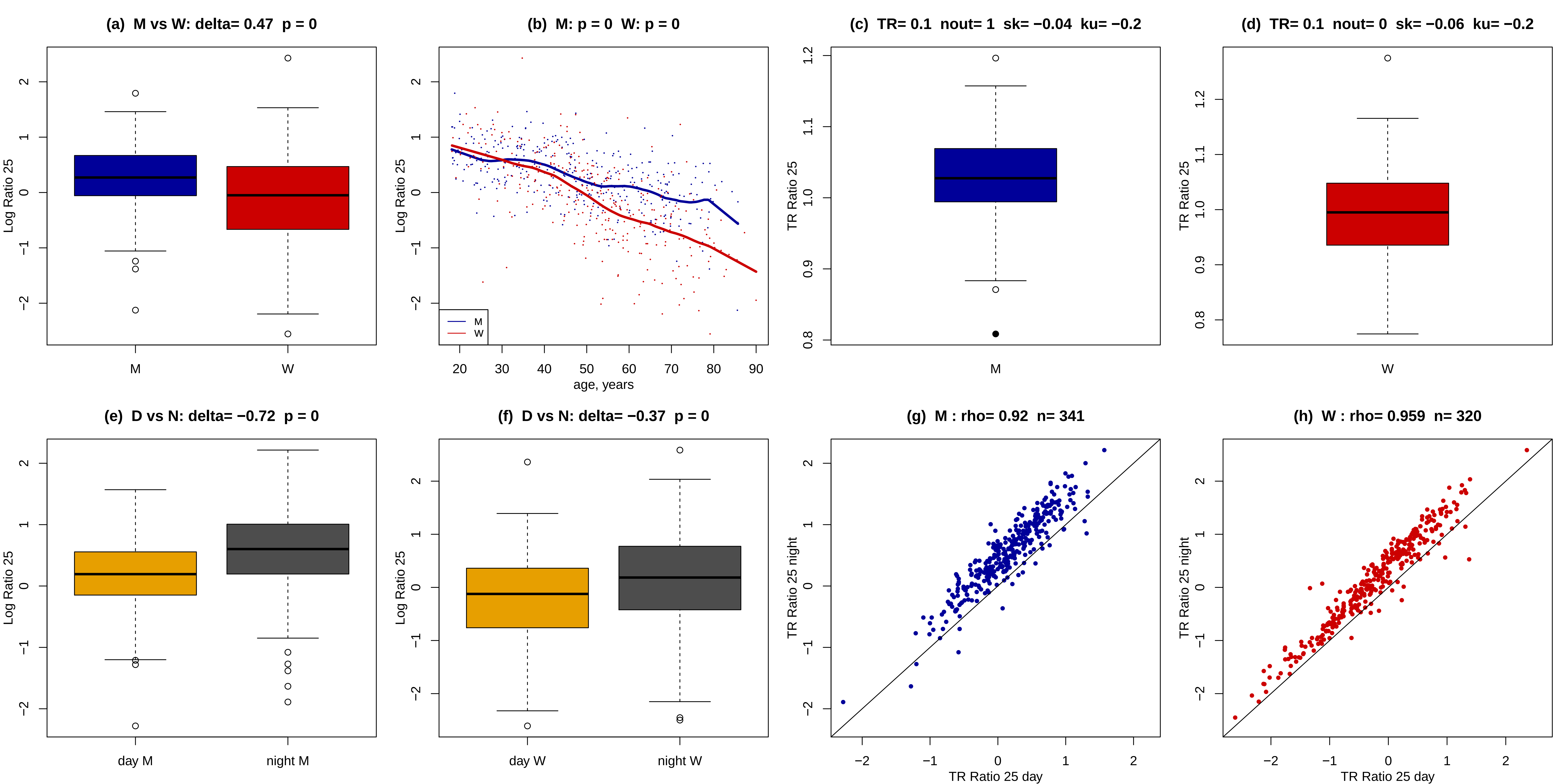
(a) M vs W: delta= 0.47 p = 0
Log Ratio 25
M
W
(b) M: p = 0 W: p = 0
Log Ratio 25
age, years
M
W
(c) TR= 0.1 nout= 1 sk= −0.04 ku= −0.2
TR Ratio 25
M
(d) TR= 0.1 nout= 0 sk= −0.06 ku= −0.2
TR Ratio 25
W
(e) D vs N: delta= −0.72 p = 0
Log Ratio 25
day M
night M
(f) D vs N: delta= −0.37 p = 0
Log Ratio 25
day W
night W
(g) M : rho= 0.92 n= 341
TR Ratio 25 night
TR Ratio 25 day
(h) W : rho= 0.959 n= 320
TR Ratio 25 night
TR Ratio 25 day

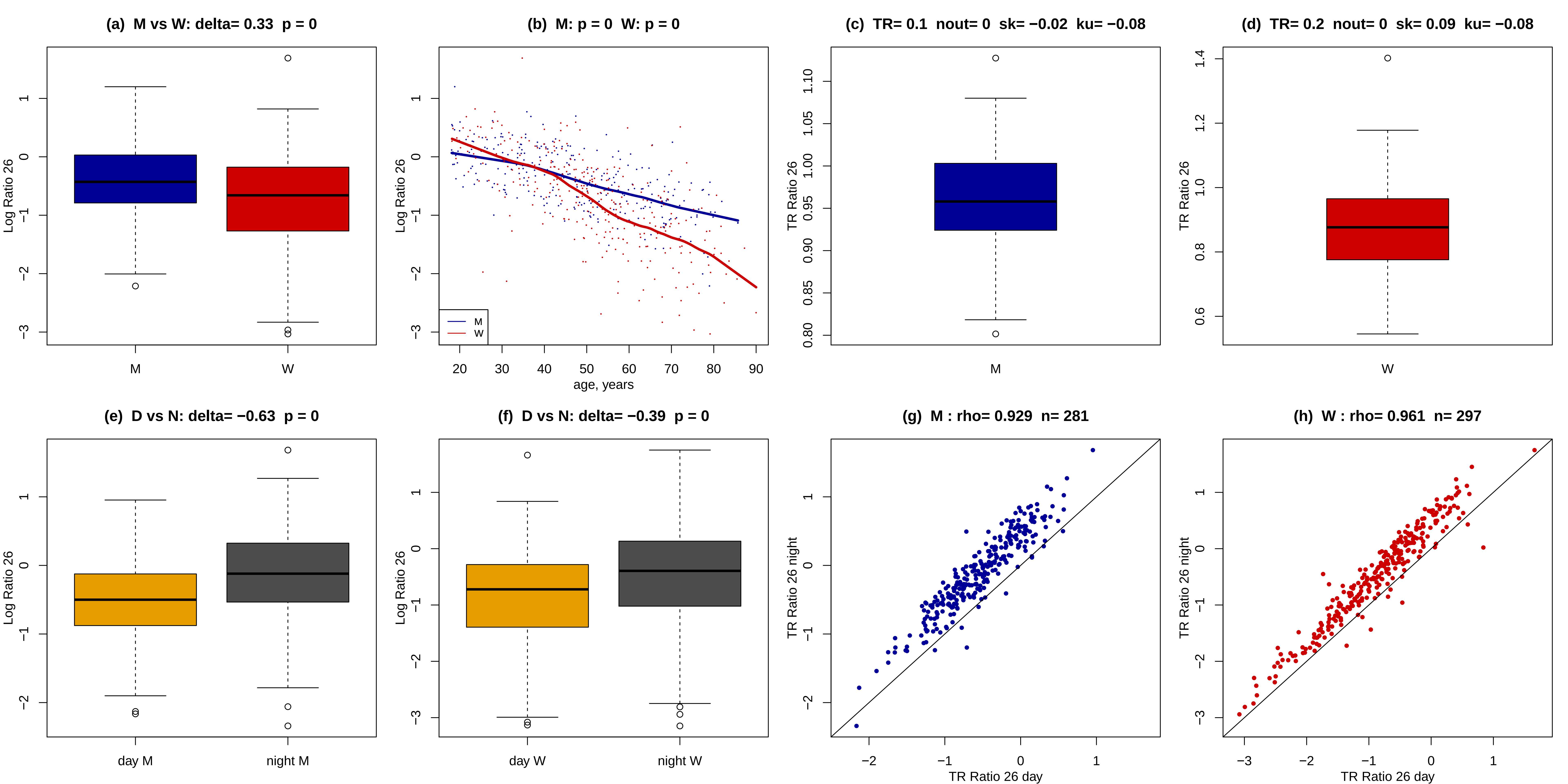
(a) M vs W: delta= 0.33 p = 0
(b) M: p = 0 W: p = 0
(c) TR= 0.1 nout= 0 sk= −0.02 ku= −0.08
(d) TR= 0.2 nout= 0 sk= 0.09 ku= −0.08
(e) D vs N: delta= −0.63 p = 0
(f) D vs N: delta= −0.39 p = 0
(g) M : rho= 0.929 n= 281
(h) W : rho= 0.961 n= 297
Log Ratio 26
TR Ratio 26
age, years
TR Ratio 26 night
TR Ratio 26 day
M
W
day M
night M
day W
night W

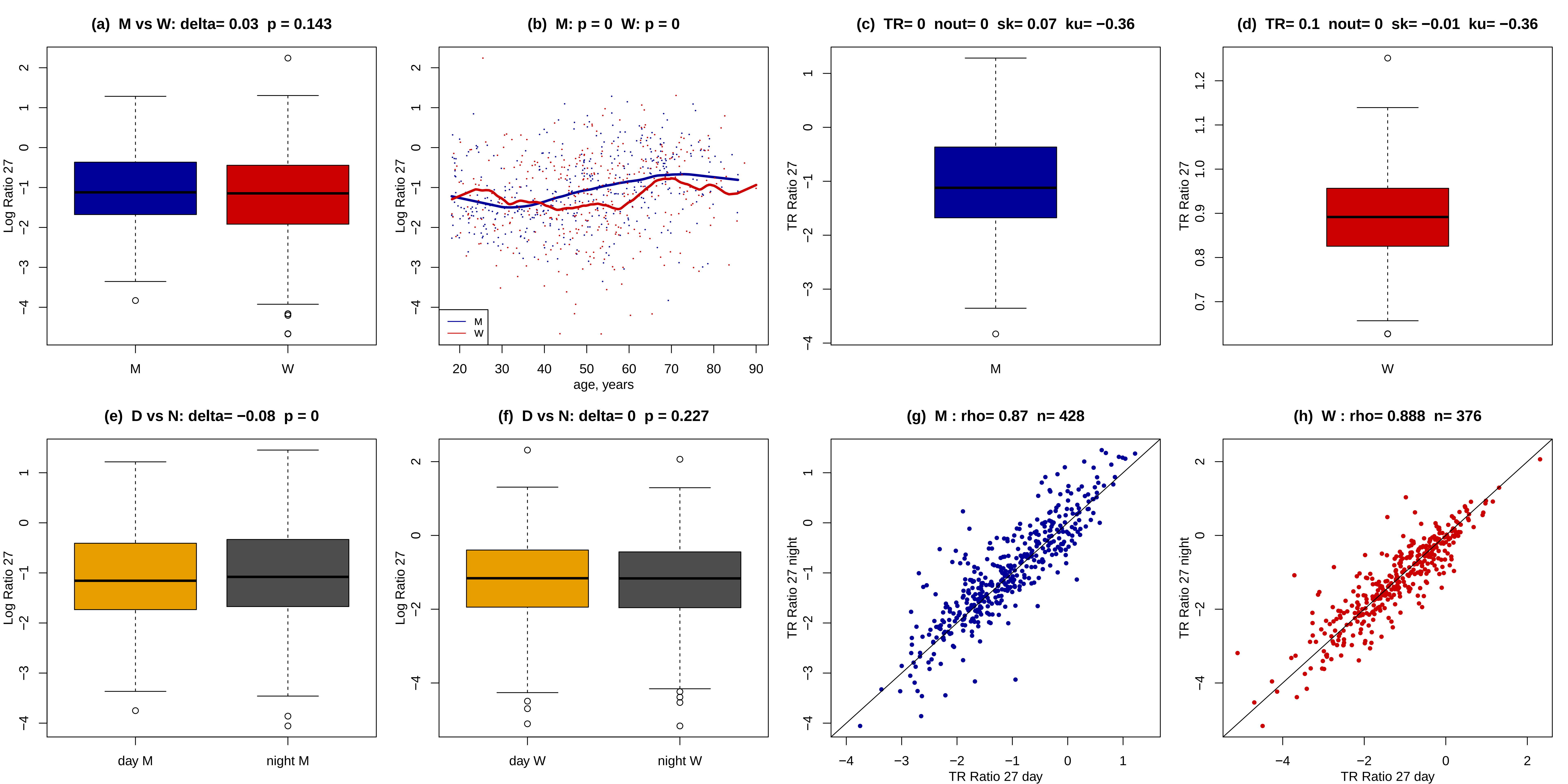
(a) M vs W: delta= 0.03 p = 0.143
Log Ratio 27
M
W
(b) M: p = 0 W: p = 0
Log Ratio 27
age, years
M
W
(c) TR= 0 nout= 0 sk= 0.07 ku= −0.36
TR Ratio 27
M
(d) TR= 0.1 nout= 0 sk= −0.01 ku= −0.36
TR Ratio 27
W
(e) D vs N: delta= −0.08 p = 0
Log Ratio 27
day M
night M
(f) D vs N: delta= 0 p = 0.227
Log Ratio 27
day W
night W
(g) M : rho= 0.87 n= 428
TR Ratio 27 night
TR Ratio 27 day
(h) W : rho= 0.888 n= 376
TR Ratio 27 night
TR Ratio 27 day

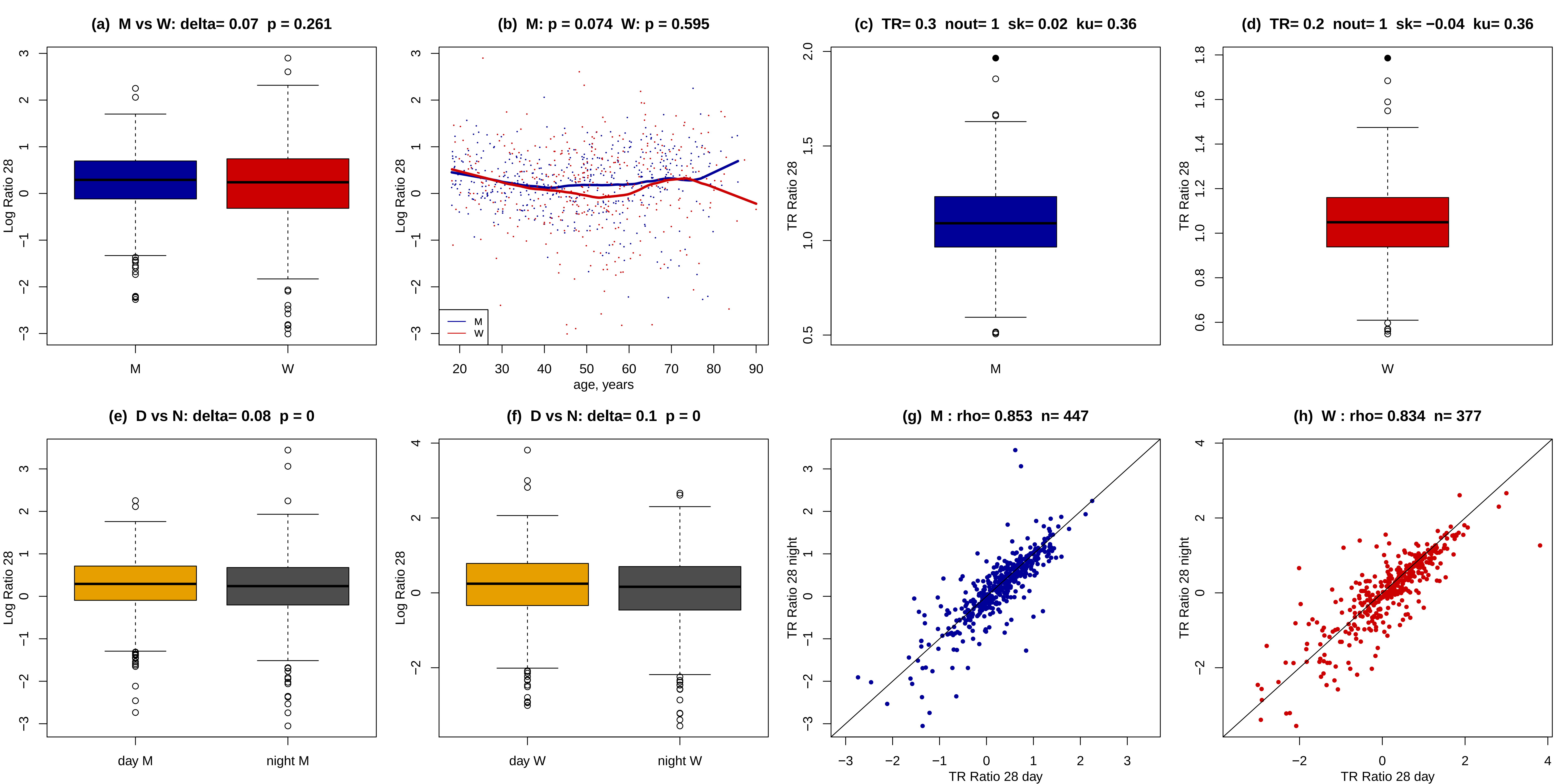

(a) M vs W: delta= 0.07 p = 0.261
Log Ratio 28
M
W
(b) M: p = 0.074 W: p = 0.595
Log Ratio 28
M
W
age, years
(c) TR= 0.3 nout= 1 sk= 0.02 ku= 0.36
TR Ratio 28
M
(d) TR= 0.2 nout= 1 sk= −0.04 ku= 0.36
TR Ratio 28
W
(e) D vs N: delta= 0.08 p = 0
Log Ratio 28
day M
night M
(f) D vs N: delta= 0.1 p = 0
Log Ratio 28
day W
night W
(g) M : rho= 0.853 n= 447
TR Ratio 28 night
TR Ratio 28 day
(h) W : rho= 0.834 n= 377
TR Ratio 28 night
TR Ratio 28 day

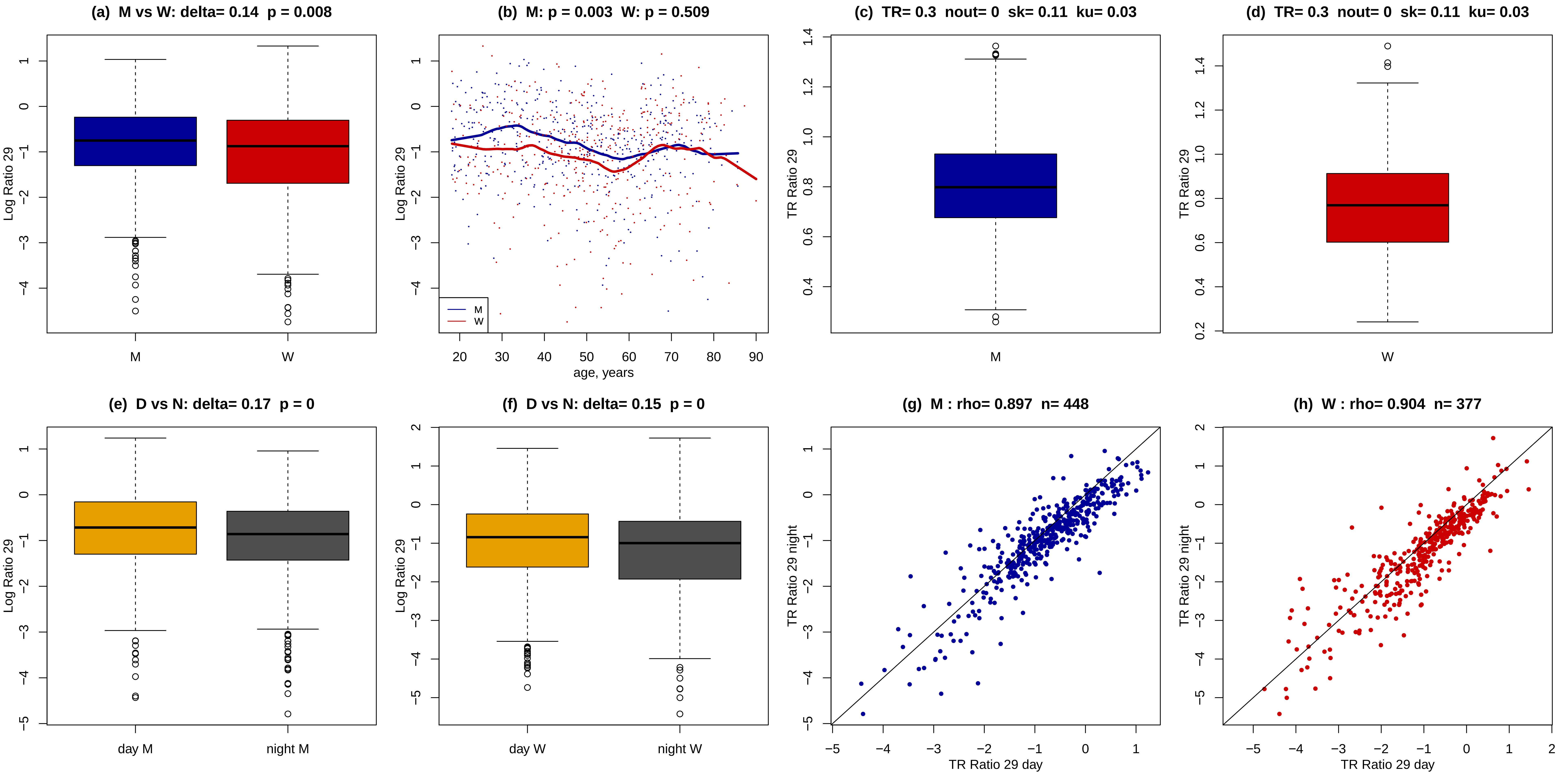
(a) M vs W: delta= 0.14 p = 0.008
Log Ratio 29
M
W
(b) M: p = 0.003 W: p = 0.509
Log Ratio 29
age, years
M
W
(c) TR= 0.3 nout= 0 sk= 0.11 ku= 0.03
TR Ratio 29
M
(d) TR= 0.3 nout= 0 sk= 0.11 ku= 0.03
TR Ratio 29
W
(e) D vs N: delta= 0.17 p = 0
Log Ratio 29
day M
night M
(f) D vs N: delta= 0.15 p = 0
Log Ratio 29
day W
night W
(g) M : rho= 0.897 n= 448
TR Ratio 29 night
TR Ratio 29 day
(h) W : rho= 0.904 n= 377
TR Ratio 29 night
TR Ratio 29 day

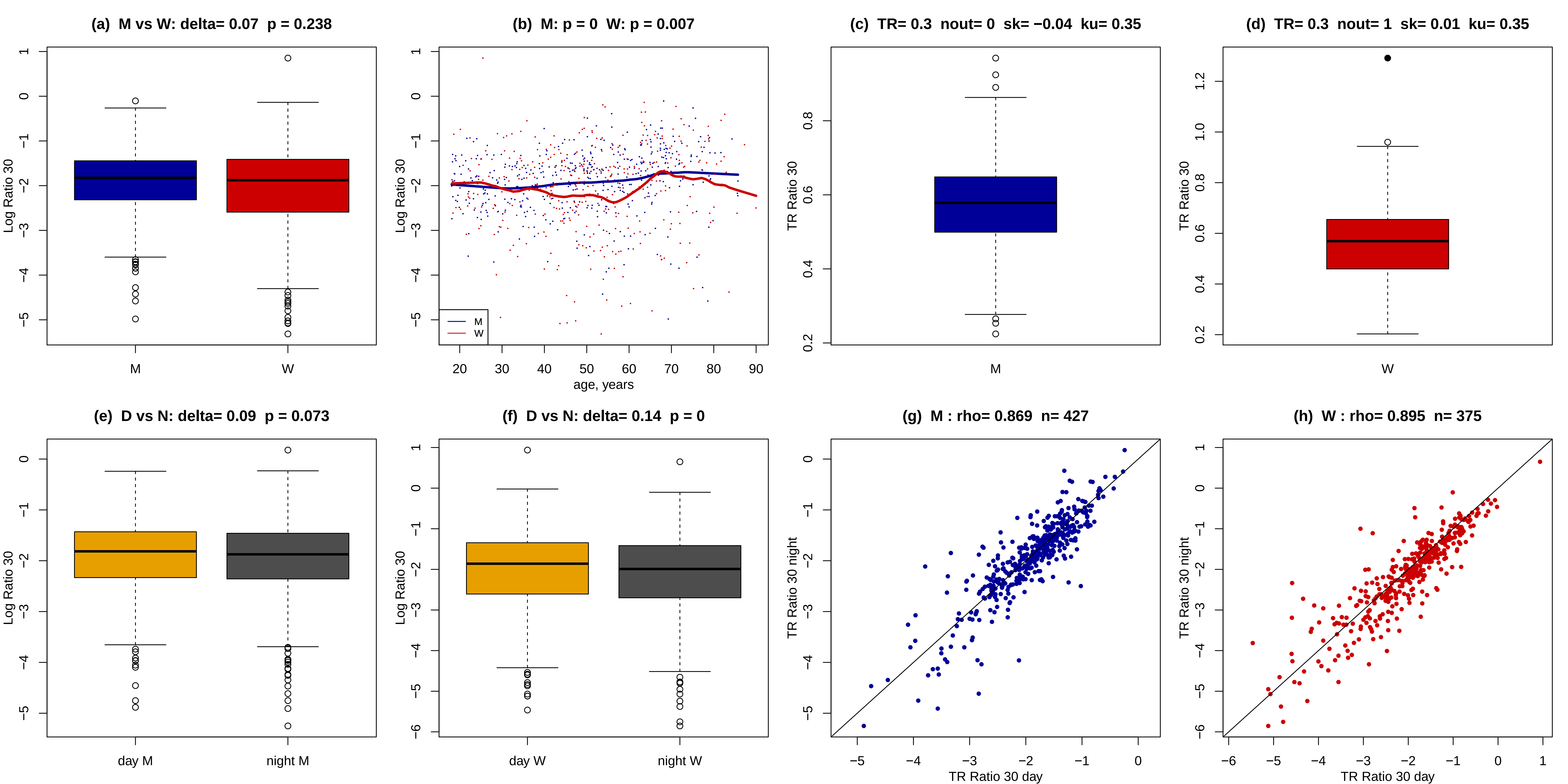
(a) M vs W: delta= 0.07 p = 0.238
Log Ratio 30
M
W
(b) M: p = 0 W: p = 0.007
Log Ratio 30
age, years
M
W
(c) TR= 0.3 nout= 0 sk= −0.04 ku= 0.35
TR Ratio 30
M
(d) TR= 0.3 nout= 1 sk= 0.01 ku= 0.35
TR Ratio 30
W
(e) D vs N: delta= 0.09 p = 0.073
Log Ratio 30
day M
night M
(f) D vs N: delta= 0.14 p = 0
Log Ratio 30
day W
night W
(g) M : rho= 0.869 n= 427
TR Ratio 30 night
TR Ratio 30 day
(h) W : rho= 0.895 n= 375
TR Ratio 30 night
TR Ratio 30 day

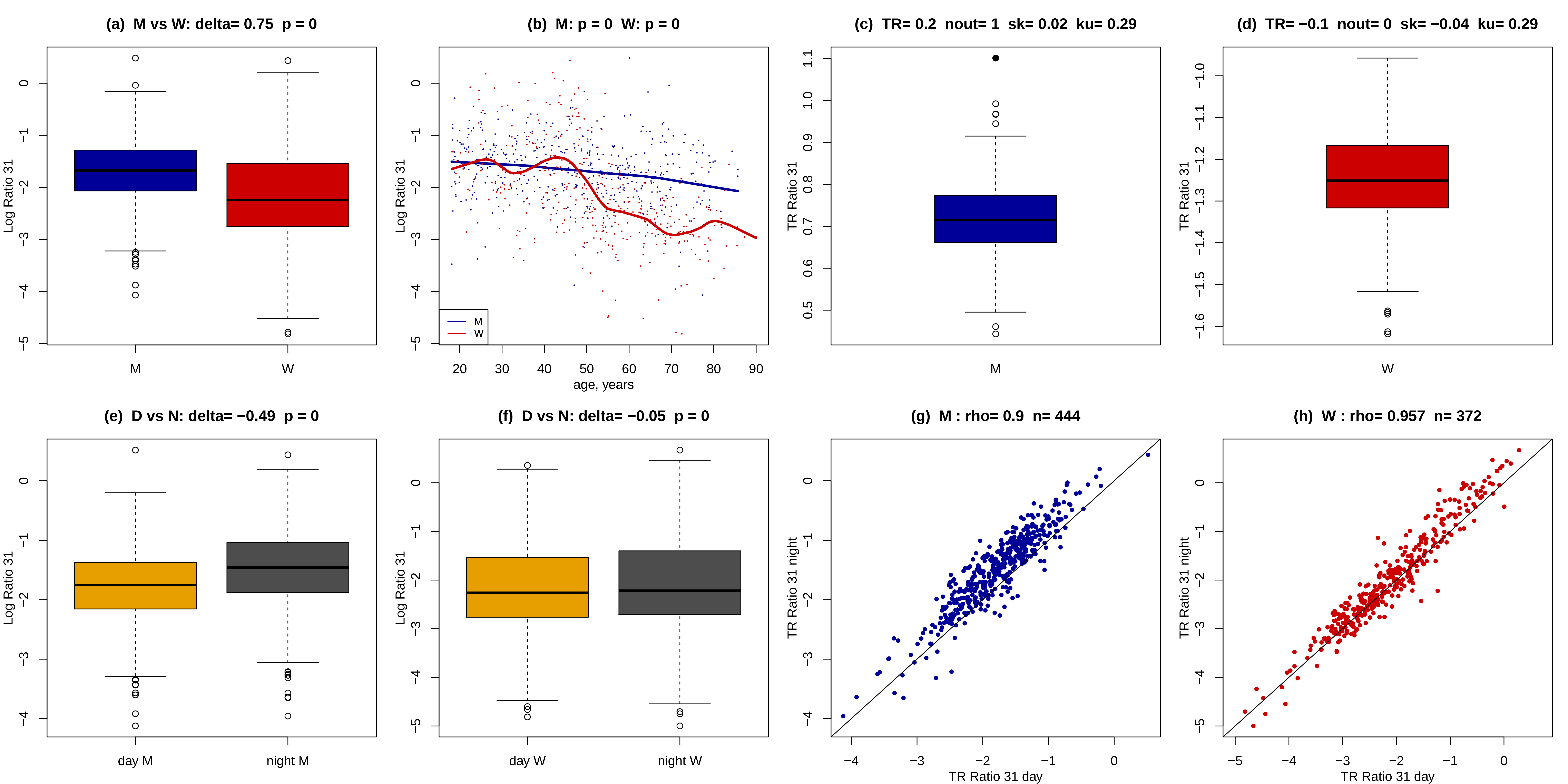
(a) M vs W: delta= 0.75 p = 0
(b) M: p = 0 W: p = 0
(c) TR= 0.2 nout= 1 sk= 0.02 ku= 0.29
(d) TR= −0.1 nout= 0 sk= −0.04 ku= 0.29
(e) D vs N: delta= −0.49 p = 0
(f) D vs N: delta= −0.05 p = 0
(g) M : rho= 0.9 n= 444
(h) W : rho= 0.957 n= 372
Log Ratio 31
TR Ratio 31
age, years
TR Ratio 31 night
TR Ratio 31 day
M
W
day M
night M
day W
night W

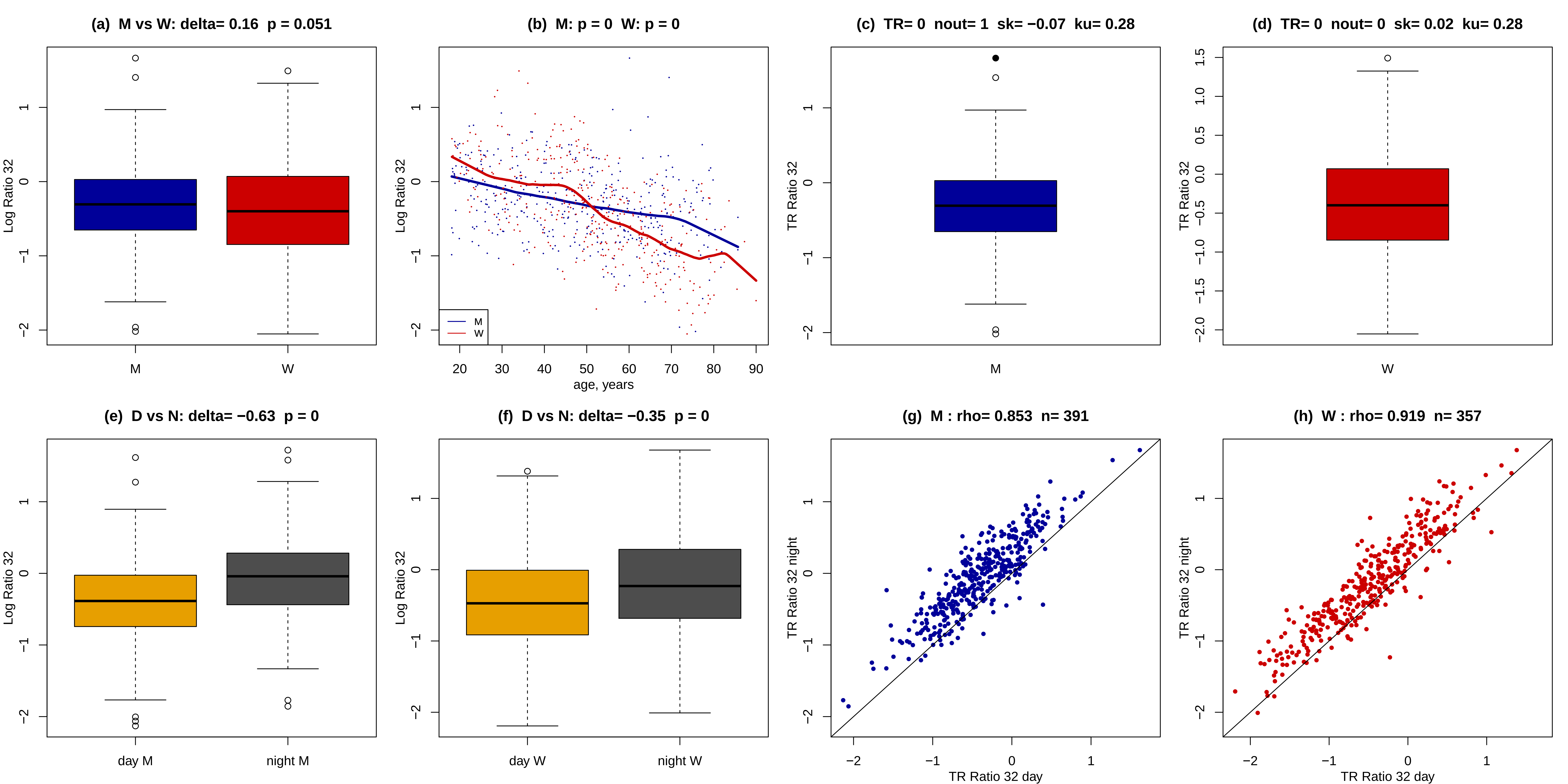
(a) M vs W: delta= 0.16 p = 0.051
Log Ratio 32
M
W
(b) M: p = 0 W: p = 0
Log Ratio 32
age, years
M
W
(c) TR= 0 nout= 1 sk= −0.07 ku= 0.28
TR Ratio 32
M
(d) TR= 0 nout= 0 sk= 0.02 ku= 0.28
TR Ratio 32
W
(e) D vs N: delta= −0.63 p = 0
Log Ratio 32
day M
night M
(f) D vs N: delta= −0.35 p = 0
Log Ratio 32
day W
night W
(g) M : rho= 0.853 n= 391
TR Ratio 32 night
TR Ratio 32 day
(h) W : rho= 0.919 n= 357
TR Ratio 32 night
TR Ratio 32 day

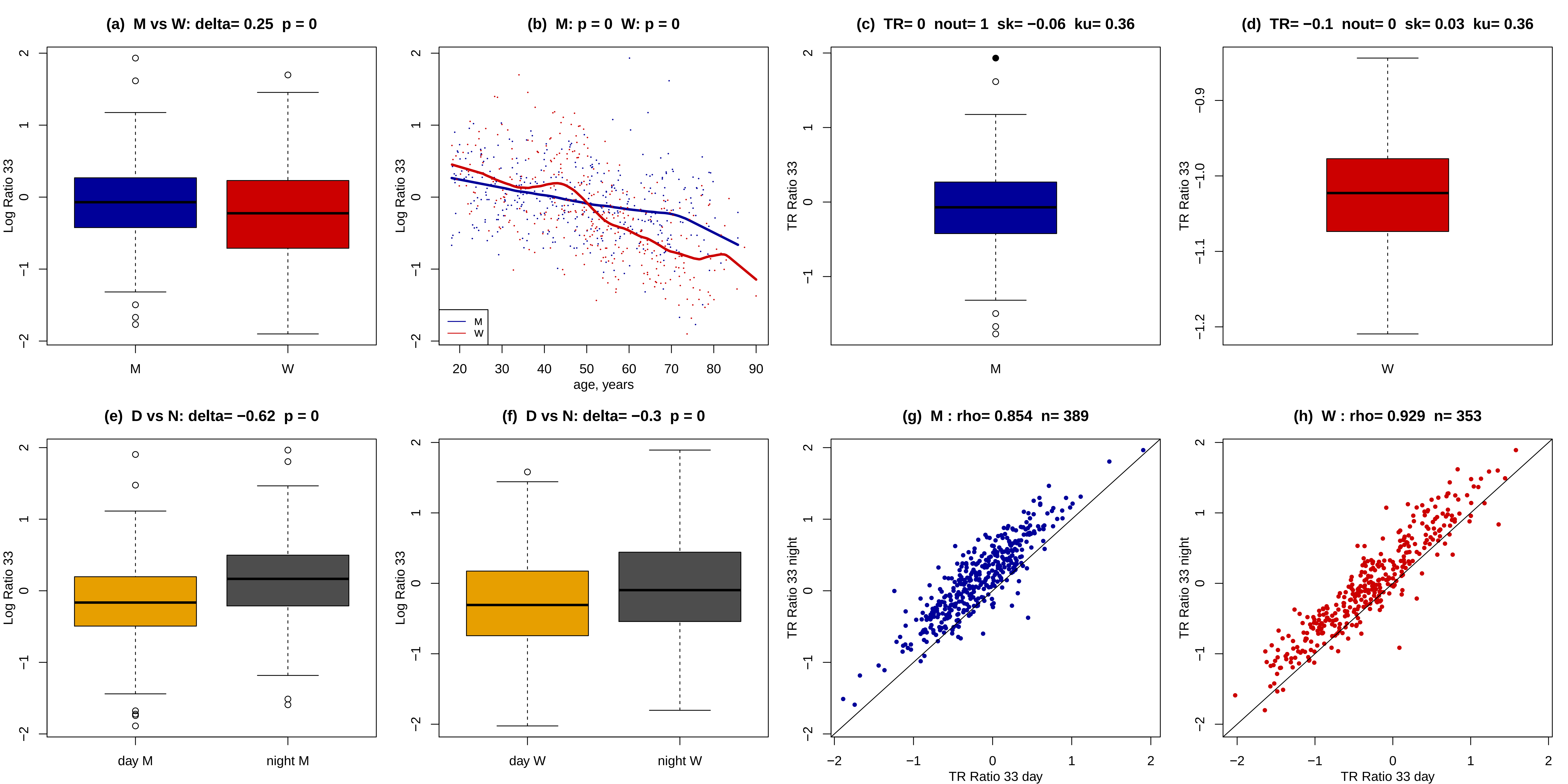

(a) M vs W: delta= 0.25 p = 0
(b) M: p = 0 W: p = 0
(c) TR= 0 nout= 1 sk= −0.06 ku= 0.36
(d) TR= −0.1 nout= 0 sk= 0.03 ku= 0.36
Log Ratio 33
TR Ratio 33
M
W
age, years
(e) D vs N: delta= −0.62 p = 0
(f) D vs N: delta= −0.3 p = 0
(g) M : rho= 0.854 n= 389
(h) W : rho= 0.929 n= 353
day M
night M
day W
night W
TR Ratio 33 night
TR Ratio 33 day

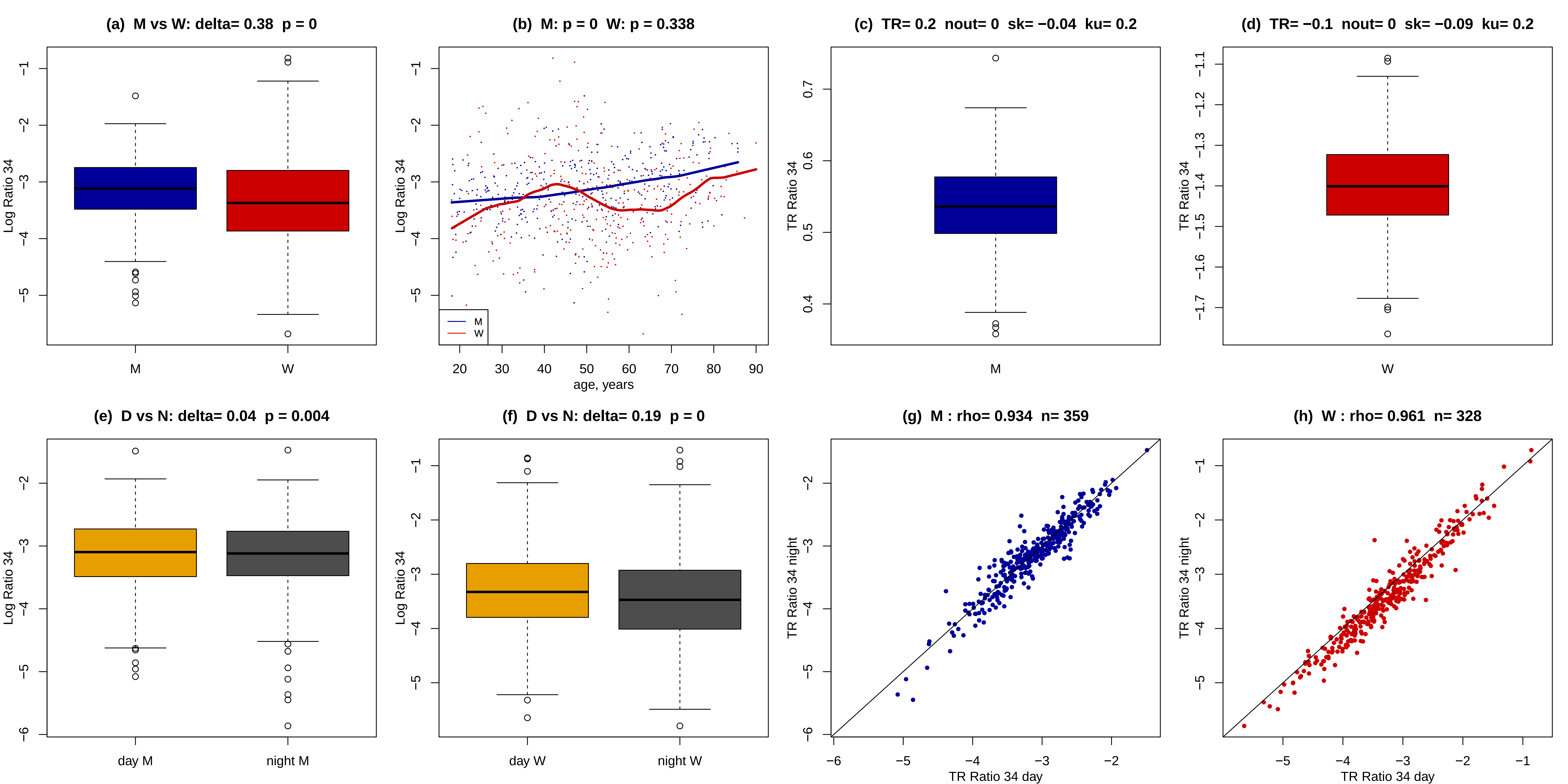
(a) M vs W: delta= 0.38 p = 0
Log Ratio 34
M
W
(b) M: p = 0 W: p = 0.338
Log Ratio 34
M
W
age, years
(c) TR= 0.2 nout= 0 sk= −0.04 ku= 0.2
TR Ratio 34
M
(d) TR= −0.1 nout= 0 sk= −0.09 ku= 0.2
TR Ratio 34
W
(e) D vs N: delta= 0.04 p = 0.004
Log Ratio 34
day M
night M
(f) D vs N: delta= 0.19 p = 0
Log Ratio 34
day W
night W
(g) M : rho= 0.934 n= 359
TR Ratio 34 night
TR Ratio 34 day
(h) W : rho= 0.961 n= 328
TR Ratio 34 night
TR Ratio 34 day

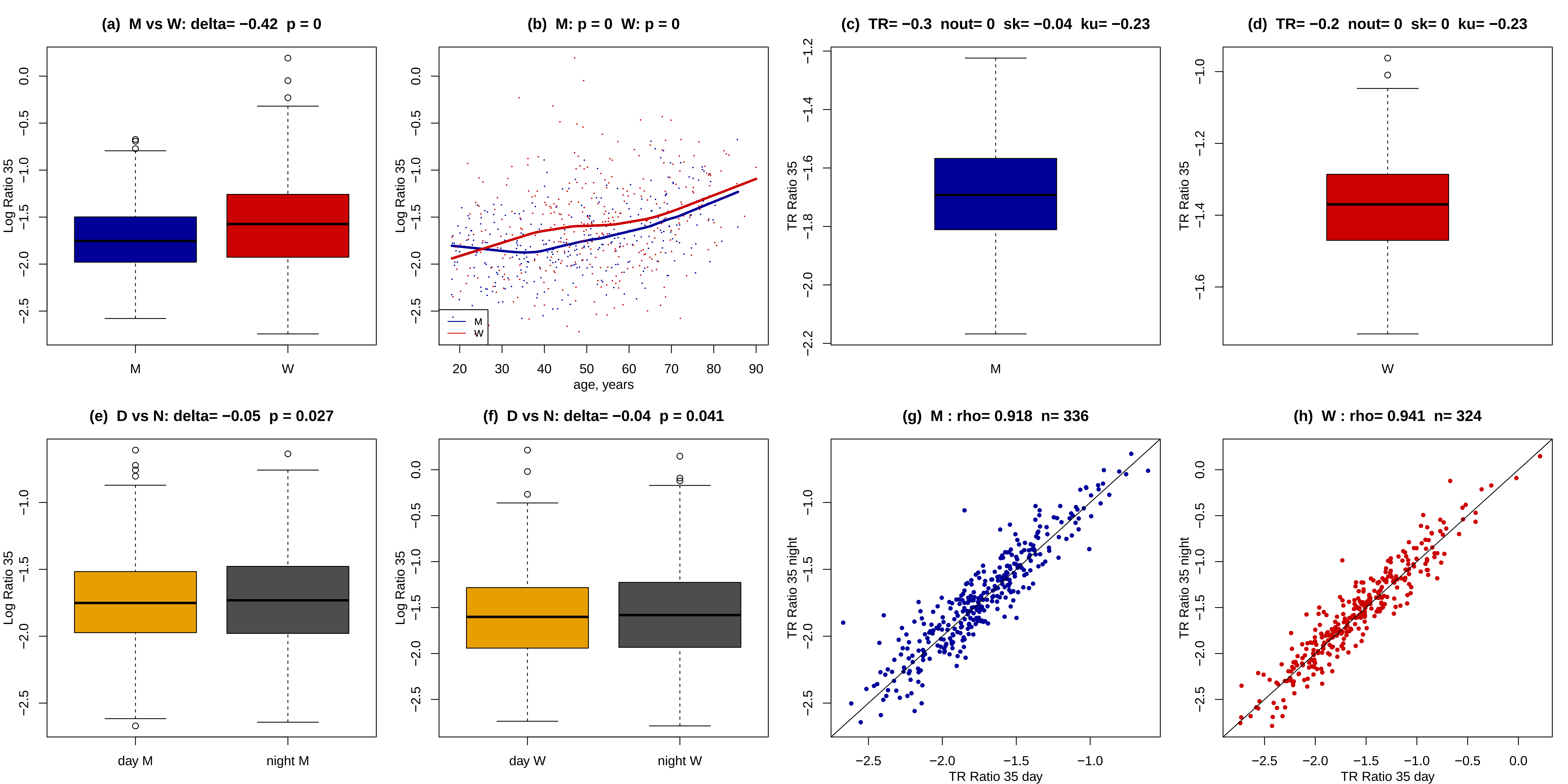

(a) M vs W: delta= −0.42 p = 0
(b) M: p = 0 W: p = 0
(c) TR= −0.3 nout= 0 sk= −0.04 ku= −0.23
(d) TR= −0.2 nout= 0 sk= 0 ku= −0.23
Log Ratio 35
TR Ratio 35
M
W
age, years
(e) D vs N: delta= −0.05 p = 0.027
(f) D vs N: delta= −0.04 p = 0.041
(g) M : rho= 0.918 n= 336
(h) W : rho= 0.941 n= 324
day M
night M
day W
night W
TR Ratio 35 night
TR Ratio 35 day

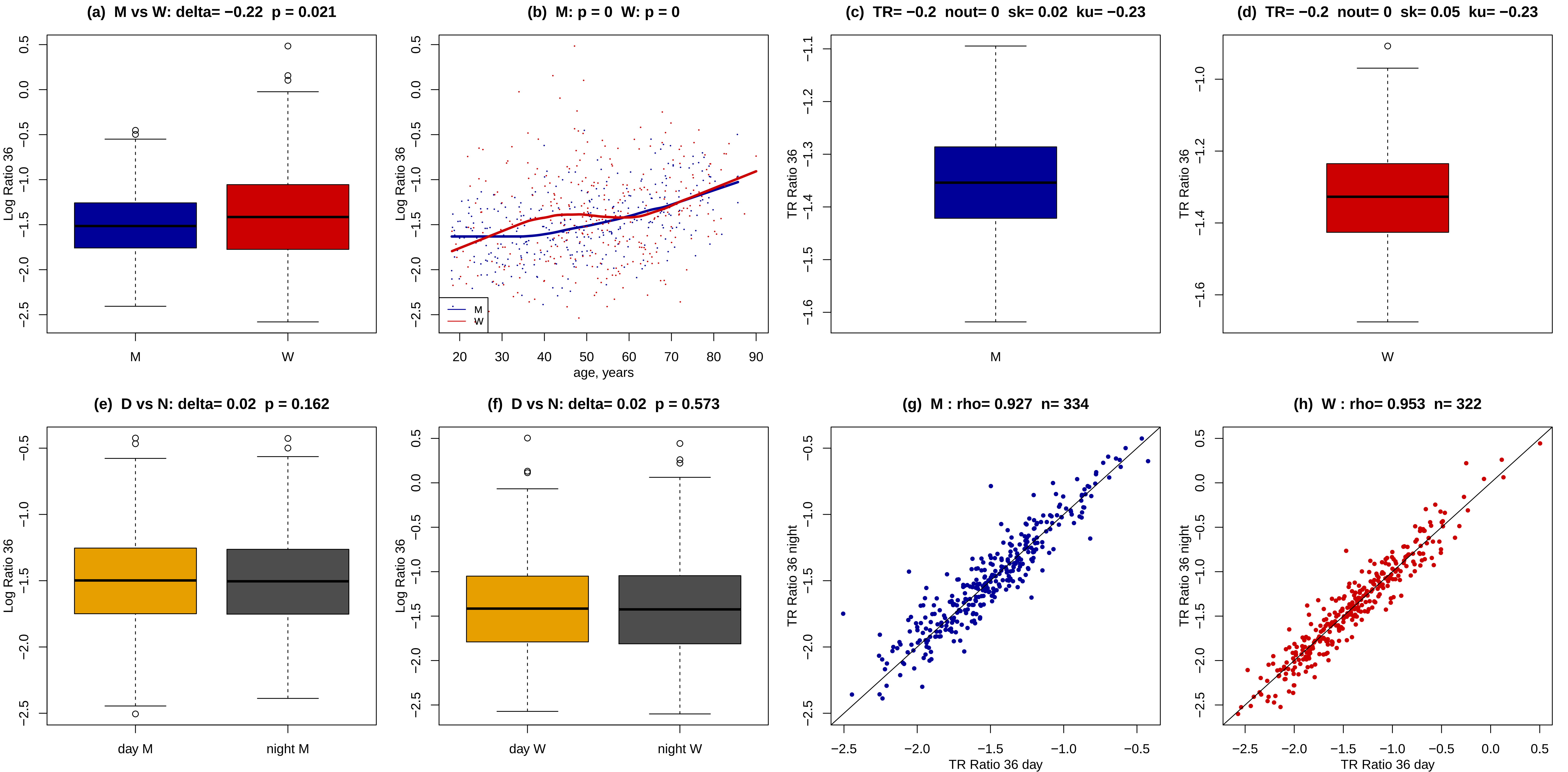

(a) M vs W: delta= −0.22 p = 0.021
(b) M: p = 0 W: p = 0
(c) TR= −0.2 nout= 0 sk= 0.02 ku= −0.23
(d) TR= −0.2 nout= 0 sk= 0.05 ku= −0.23
(e) D vs N: delta= 0.02 p = 0.162
(f) D vs N: delta= 0.02 p = 0.573
(g) M : rho= 0.927 n= 334
(h) W : rho= 0.953 n= 322
Log Ratio 36
TR Ratio 36
TR Ratio 36 night
TR Ratio 36 day
age, years
M
W
day M
night M
day W
night W

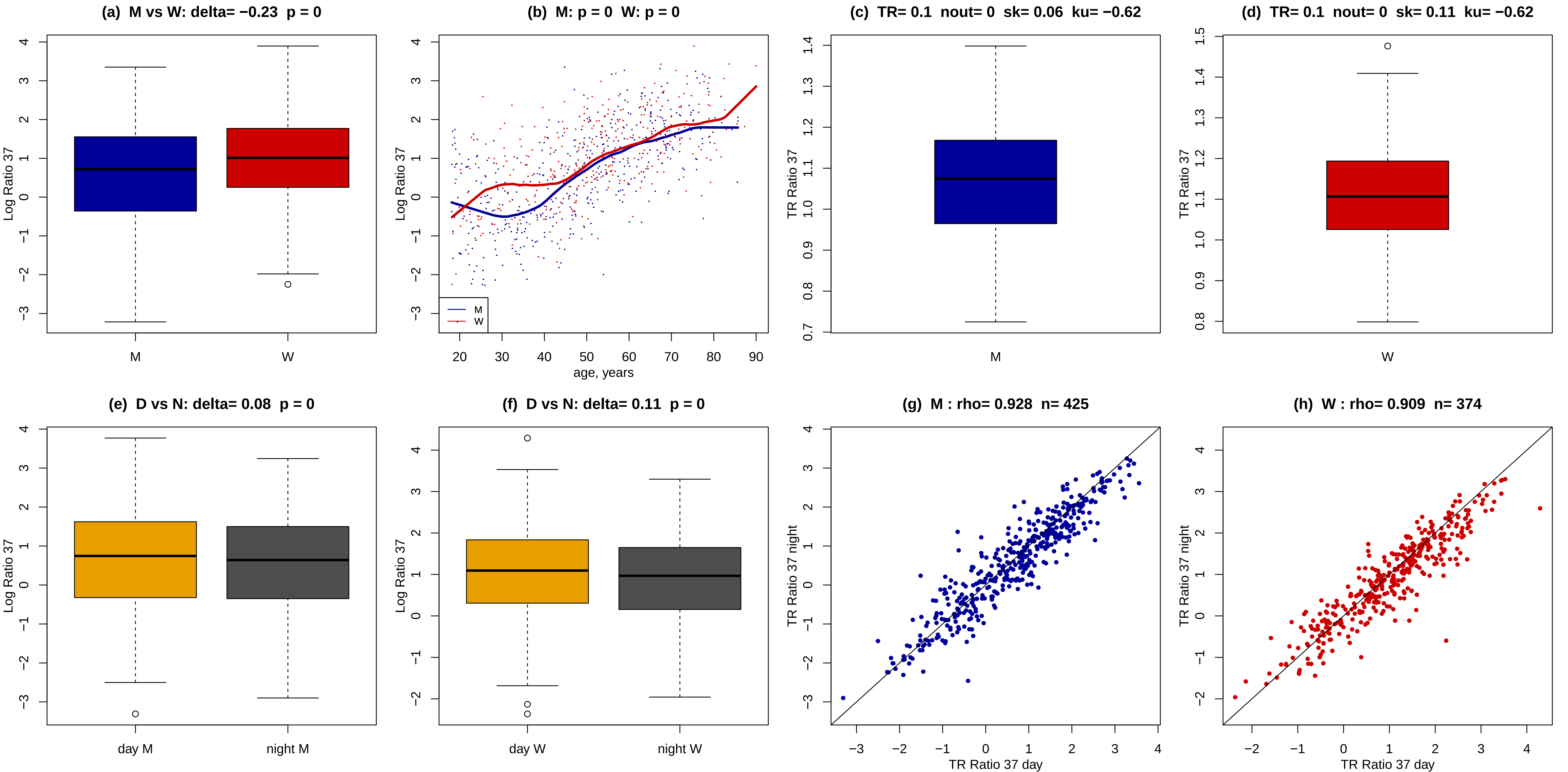
(a) M vs W: delta= −0.23 p = 0
(b) M: p = 0 W: p = 0
(c) TR= 0.1 nout= 0 sk= 0.06 ku= −0.62
(d) TR= 0.1 nout= 0 sk= 0.11 ku= −0.62
(e) D vs N: delta= 0.08 p = 0
(f) D vs N: delta= 0.11 p = 0
(g) M : rho= 0.928 n= 425
(h) W : rho= 0.909 n= 374
Log Ratio 37
TR Ratio 37
age, years
TR Ratio 37 night
TR Ratio 37 day
M
W
day M
night M
day W
night W

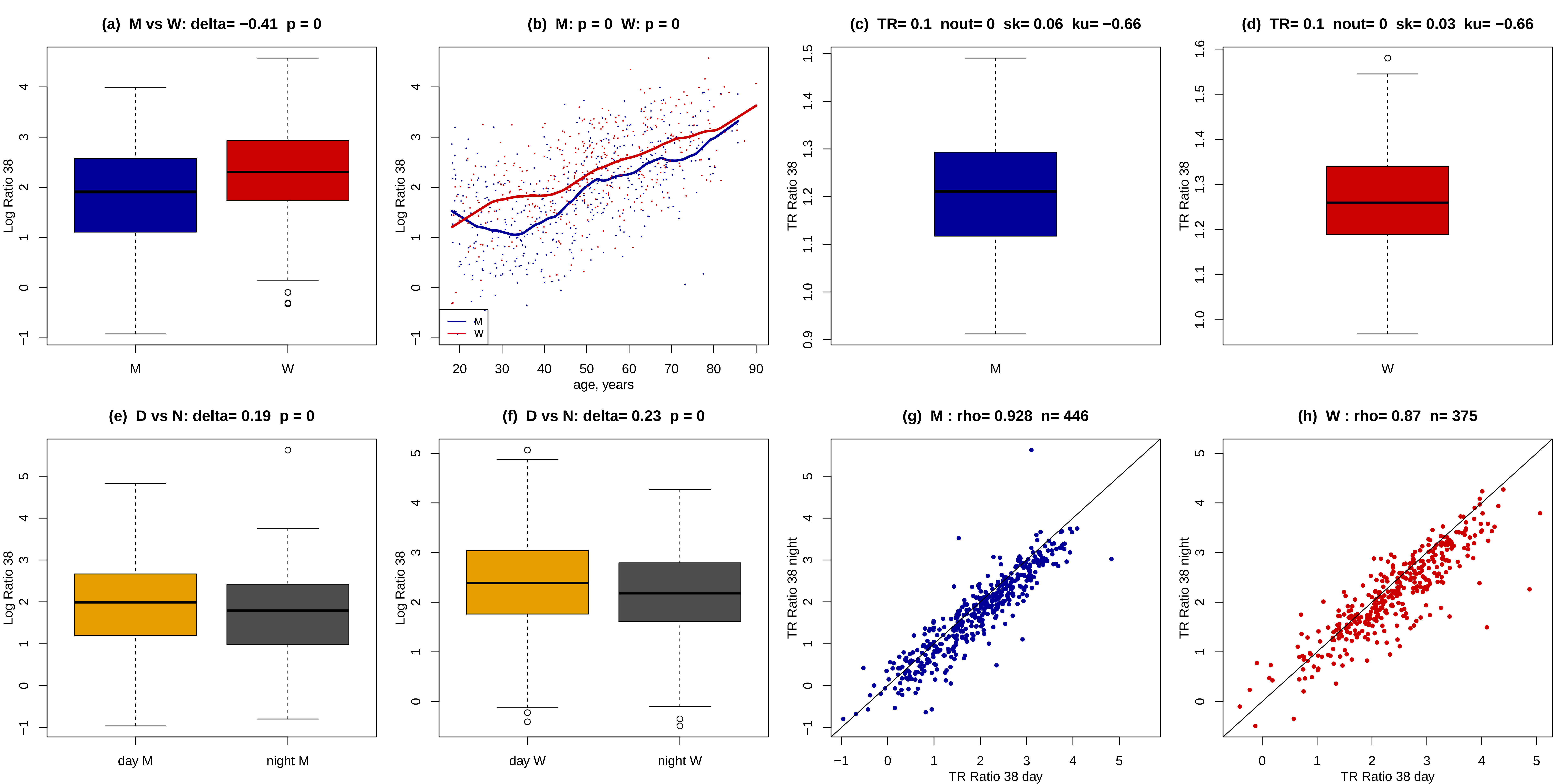
(a) M vs W: delta= −0.41 p = 0
Log Ratio 38
M
W
(b) M: p = 0 W: p = 0
Log Ratio 38
age, years
M
W
(c) TR= 0.1 nout= 0 sk= 0.06 ku= −0.66
TR Ratio 38
M
(d) TR= 0.1 nout= 0 sk= 0.03 ku= −0.66
TR Ratio 38
W
(e) D vs N: delta= 0.19 p = 0
Log Ratio 38
day M
night M
(f) D vs N: delta= 0.23 p = 0
Log Ratio 38
day W
night W
(g) M : rho= 0.928 n= 446
TR Ratio 38 night
TR Ratio 38 day
(h) W : rho= 0.87 n= 375
TR Ratio 38 night
TR Ratio 38 day

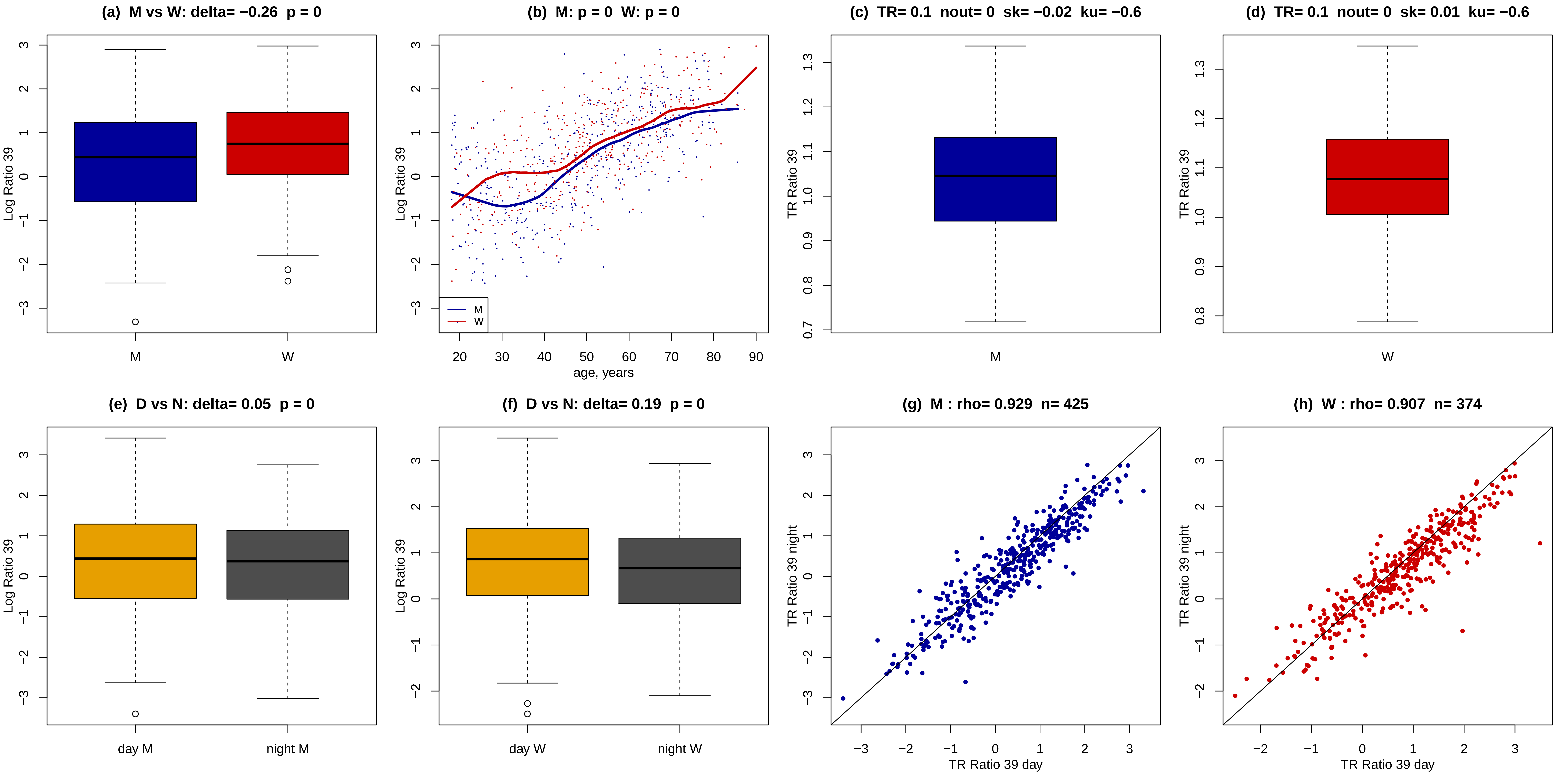
(a) M vs W: delta= −0.26 p = 0
(b) M: p = 0 W: p = 0
(c) TR= 0.1 nout= 0 sk= −0.02 ku= −0.6
(d) TR= 0.1 nout= 0 sk= 0.01 ku= −0.6
Log Ratio 39
TR Ratio 39
M
W
age, years
(e) D vs N: delta= 0.05 p = 0
(f) D vs N: delta= 0.19 p = 0
(g) M : rho= 0.929 n= 425
(h) W : rho= 0.907 n= 374
day M
night M
day W
night W
TR Ratio 39 night
TR Ratio 39 day

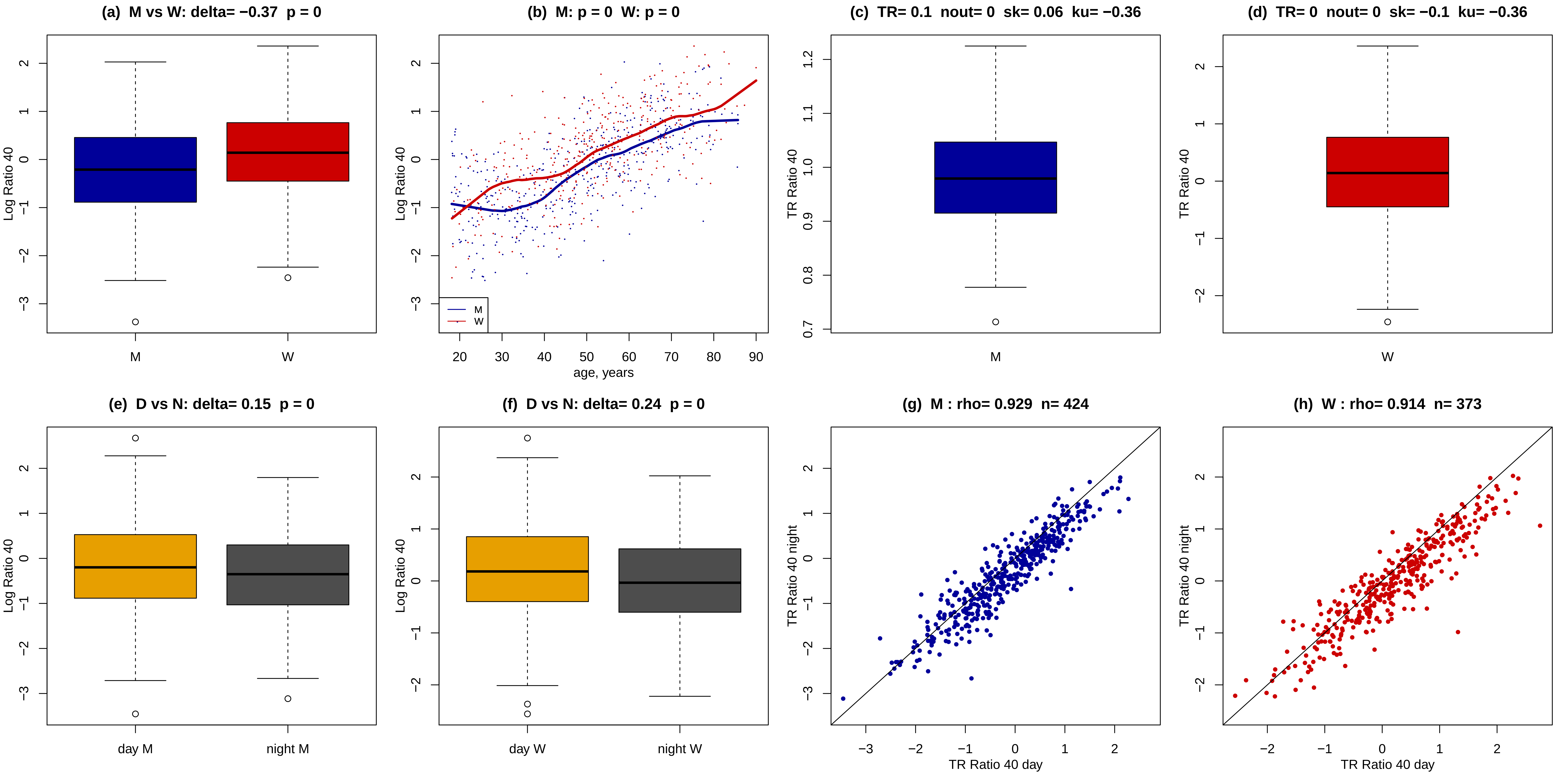
(a) M vs W: delta= −0.37 p = 0
Log Ratio 40
M
W
(b) M: p = 0 W: p = 0
Log Ratio 40
age, years
M
W
(c) TR= 0.1 nout= 0 sk= 0.06 ku= −0.36
TR Ratio 40
M
(d) TR= 0 nout= 0 sk= −0.1 ku= −0.36
TR Ratio 40
W
(e) D vs N: delta= 0.15 p = 0
Log Ratio 40
day M
night M
(f) D vs N: delta= 0.24 p = 0
Log Ratio 40
day W
night W
(g) M : rho= 0.929 n= 424
TR Ratio 40 night
TR Ratio 40 day
(h) W : rho= 0.914 n= 373
TR Ratio 40 night
TR Ratio 40 day

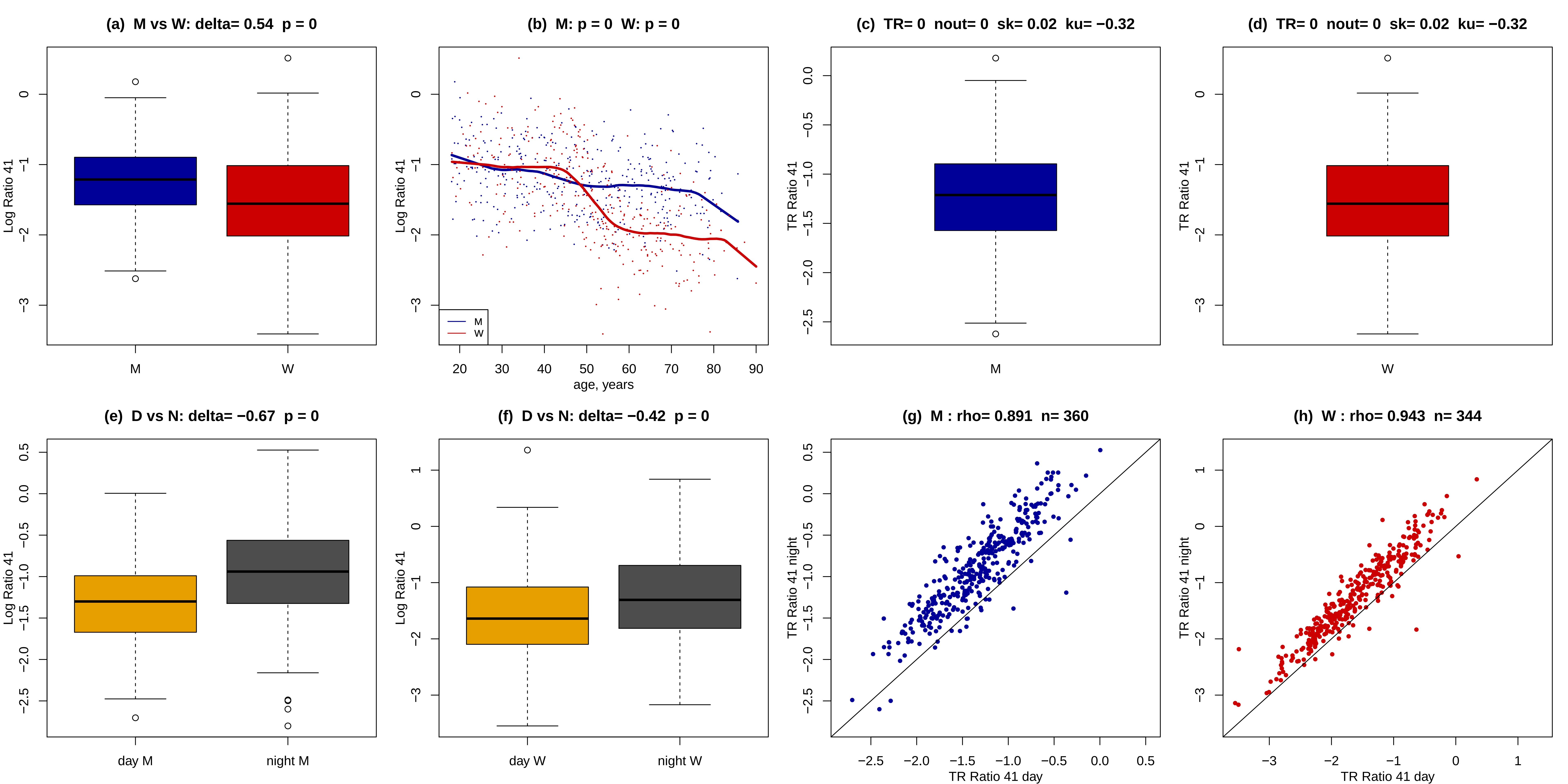

(a) M vs W: delta= 0.54 p = 0
(b) M: p = 0 W: p = 0
(c) TR= 0 nout= 0 sk= 0.02 ku= −0.32
(d) TR= 0 nout= 0 sk= 0.02 ku= −0.32
Log Ratio 41
TR Ratio 41
M
W
age, years
(e) D vs N: delta= −0.67 p = 0
(f) D vs N: delta= −0.42 p = 0
(g) M : rho= 0.891 n= 360
(h) W : rho= 0.943 n= 344
day M
night M
day W
night W
TR Ratio 41 night
TR Ratio 41 day

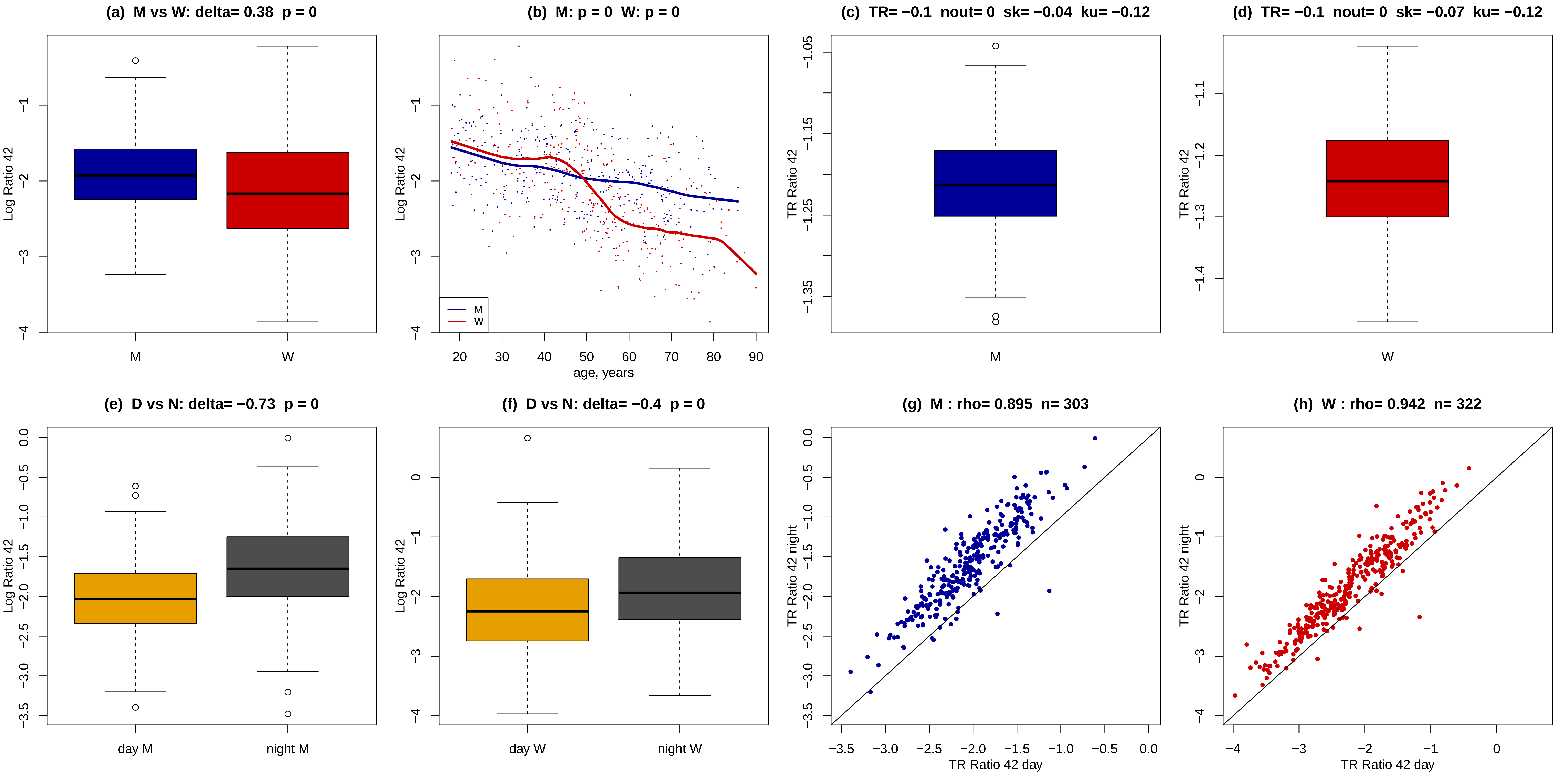
(a) M vs W: delta= 0.38 p = 0
Log Ratio 42
M
W
(b) M: p = 0 W: p = 0
Log Ratio 42
age, years
M
W
(c) TR= −0.1 nout= 0 sk= −0.04 ku= −0.12
TR Ratio 42
M
(d) TR= −0.1 nout= 0 sk= −0.07 ku= −0.12
TR Ratio 42
W
(e) D vs N: delta= −0.73 p = 0
Log Ratio 42
day M
night M
(f) D vs N: delta= −0.4 p = 0
Log Ratio 42
day W
night W
(g) M : rho= 0.895 n= 303
TR Ratio 42 night
TR Ratio 42 day
(h) W : rho= 0.942 n= 322
TR Ratio 42 night
TR Ratio 42 day

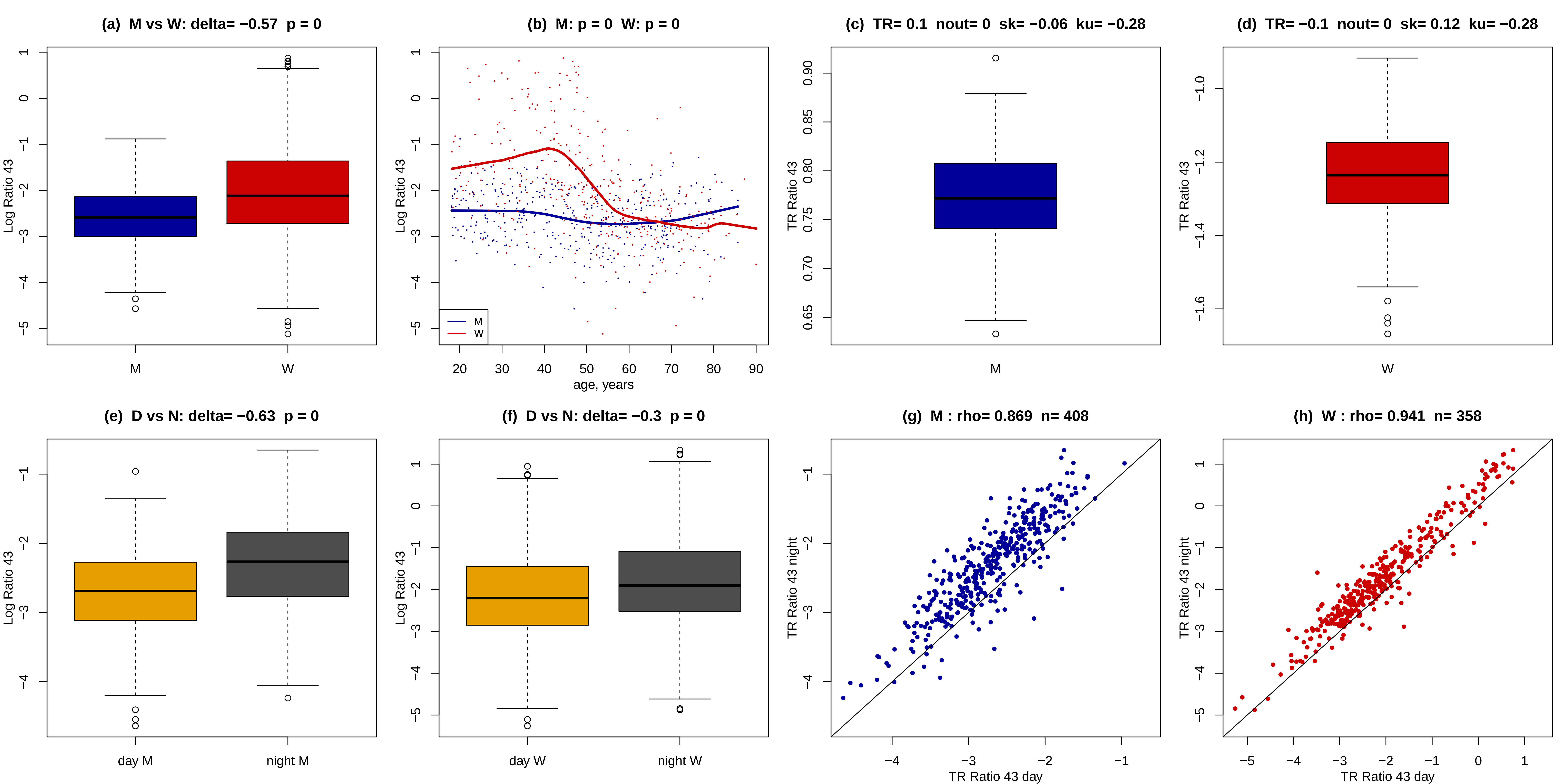

(a) M vs W: delta= −0.57 p = 0
(b) M: p = 0 W: p = 0
(c) TR= 0.1 nout= 0 sk= −0.06 ku= −0.28
(d) TR= −0.1 nout= 0 sk= 0.12 ku= −0.28
(e) D vs N: delta= −0.63 p = 0
(f) D vs N: delta= −0.3 p = 0
(g) M : rho= 0.869 n= 408
(h) W : rho= 0.941 n= 358
Log Ratio 43
TR Ratio 43
TR Ratio 43 night
TR Ratio 43 day
age, years
M
W
day M
night M
day W
night W

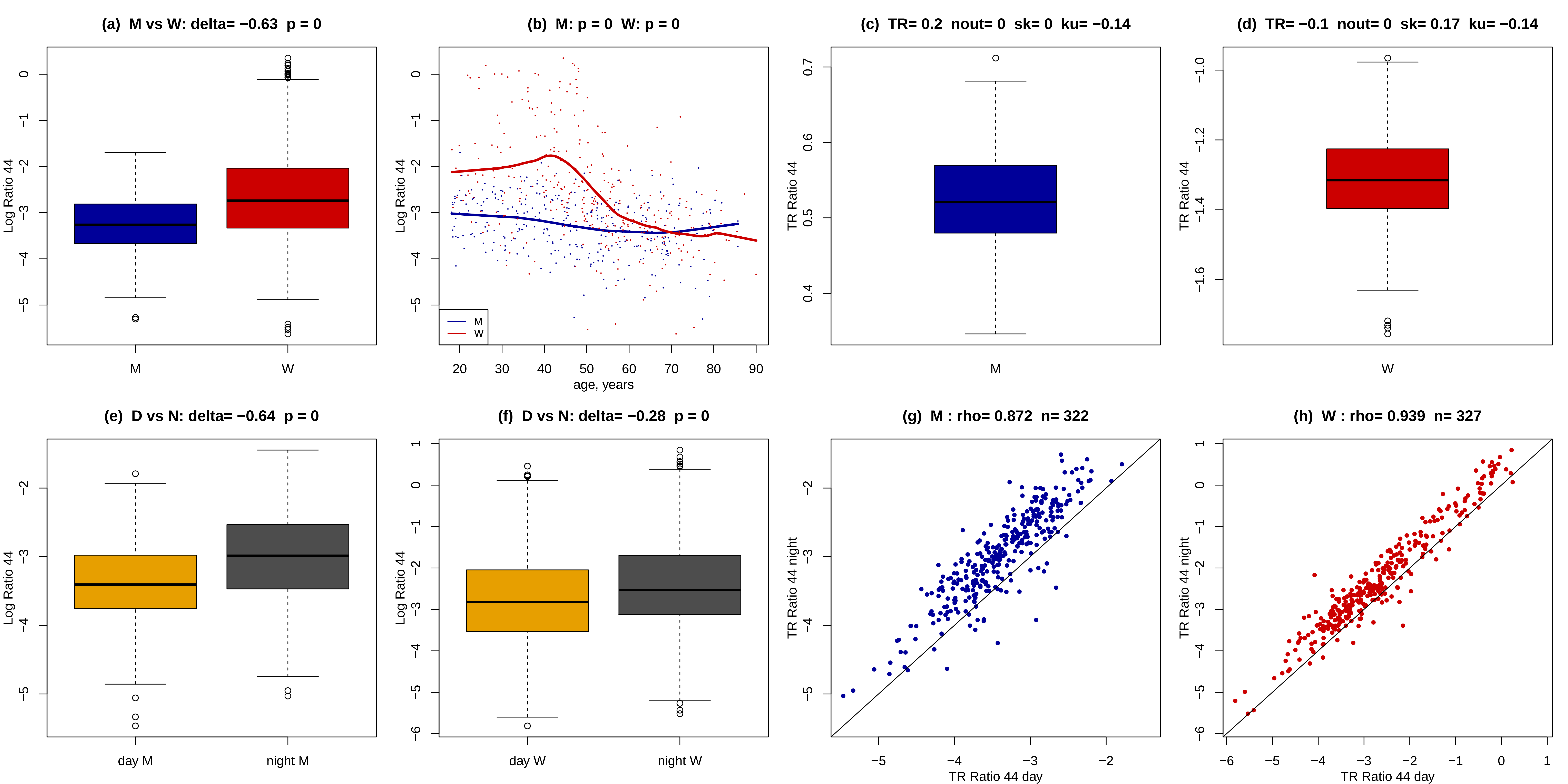

(a) M vs W: delta= −0.63 p = 0
(b) M: p = 0 W: p = 0
(c) TR= 0.2 nout= 0 sk= 0 ku= −0.14
(d) TR= −0.1 nout= 0 sk= 0.17 ku= −0.14
(e) D vs N: delta= −0.64 p = 0
(f) D vs N: delta= −0.28 p = 0
(g) M : rho= 0.872 n= 322
(h) W : rho= 0.939 n= 327
Log Ratio 44
TR Ratio 44
age, years
TR Ratio 44 night
TR Ratio 44 day
M
W
day M
night M
day W
night W

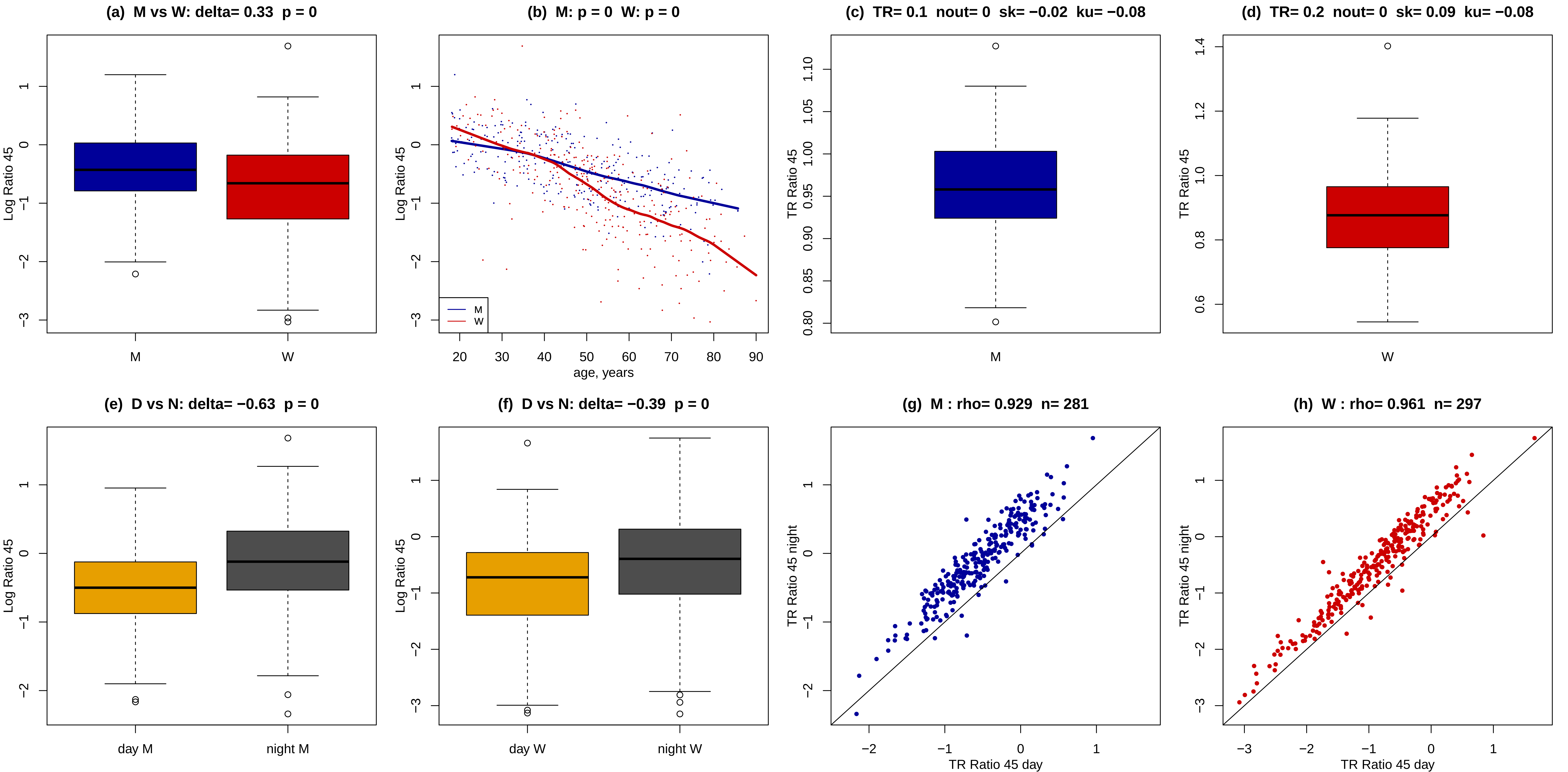
(a) M vs W: delta= 0.33 p = 0
Log Ratio 45
M
W
(b) M: p = 0 W: p = 0
Log Ratio 45
age, years
M
W
(c) TR= 0.1 nout= 0 sk= −0.02 ku= −0.08
TR Ratio 45
M
(d) TR= 0.2 nout= 0 sk= 0.09 ku= −0.08
TR Ratio 45
W
(e) D vs N: delta= −0.63 p = 0
Log Ratio 45
day M
night M
(f) D vs N: delta= −0.39 p = 0
Log Ratio 45
day W
night W
(g) M : rho= 0.929 n= 281
TR Ratio 45 night
TR Ratio 45 day
(h) W : rho= 0.961 n= 297
TR Ratio 45 night
TR Ratio 45 day

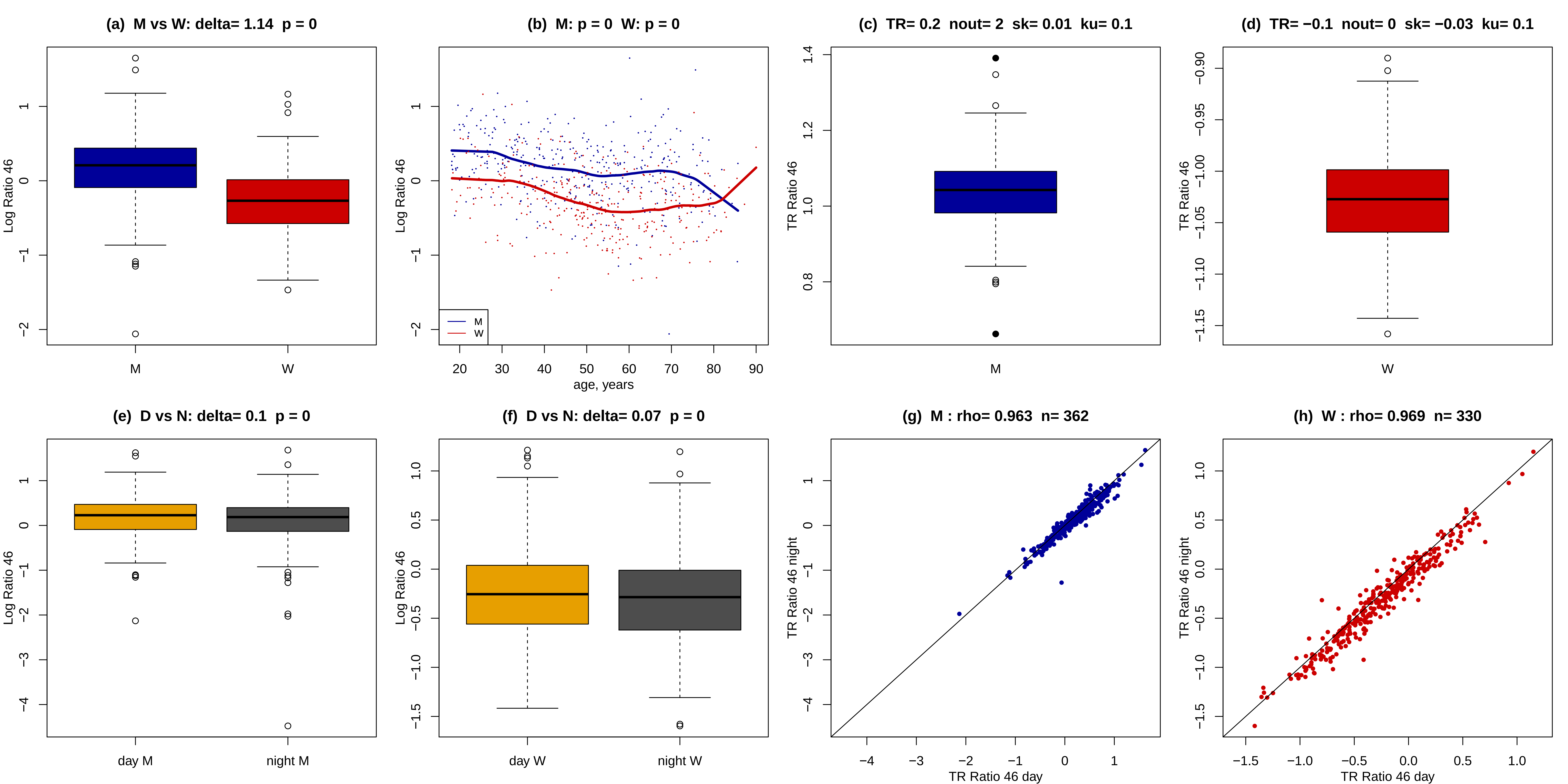

(a) M vs W: delta= 1.14 p = 0
Log Ratio 46
M
W
(b) M: p = 0 W: p = 0
Log Ratio 46
age, years
M
W
(c) TR= 0.2 nout= 2 sk= 0.01 ku= 0.1
TR Ratio 46
M
(d) TR= −0.1 nout= 0 sk= −0.03 ku= 0.1
TR Ratio 46
W
(e) D vs N: delta= 0.1 p = 0
Log Ratio 46
day M
night M
(f) D vs N: delta= 0.07 p = 0
Log Ratio 46
day W
night W
(g) M : rho= 0.963 n= 362
TR Ratio 46 night
TR Ratio 46 day
(h) W : rho= 0.969 n= 330
TR Ratio 46 night
TR Ratio 46 day

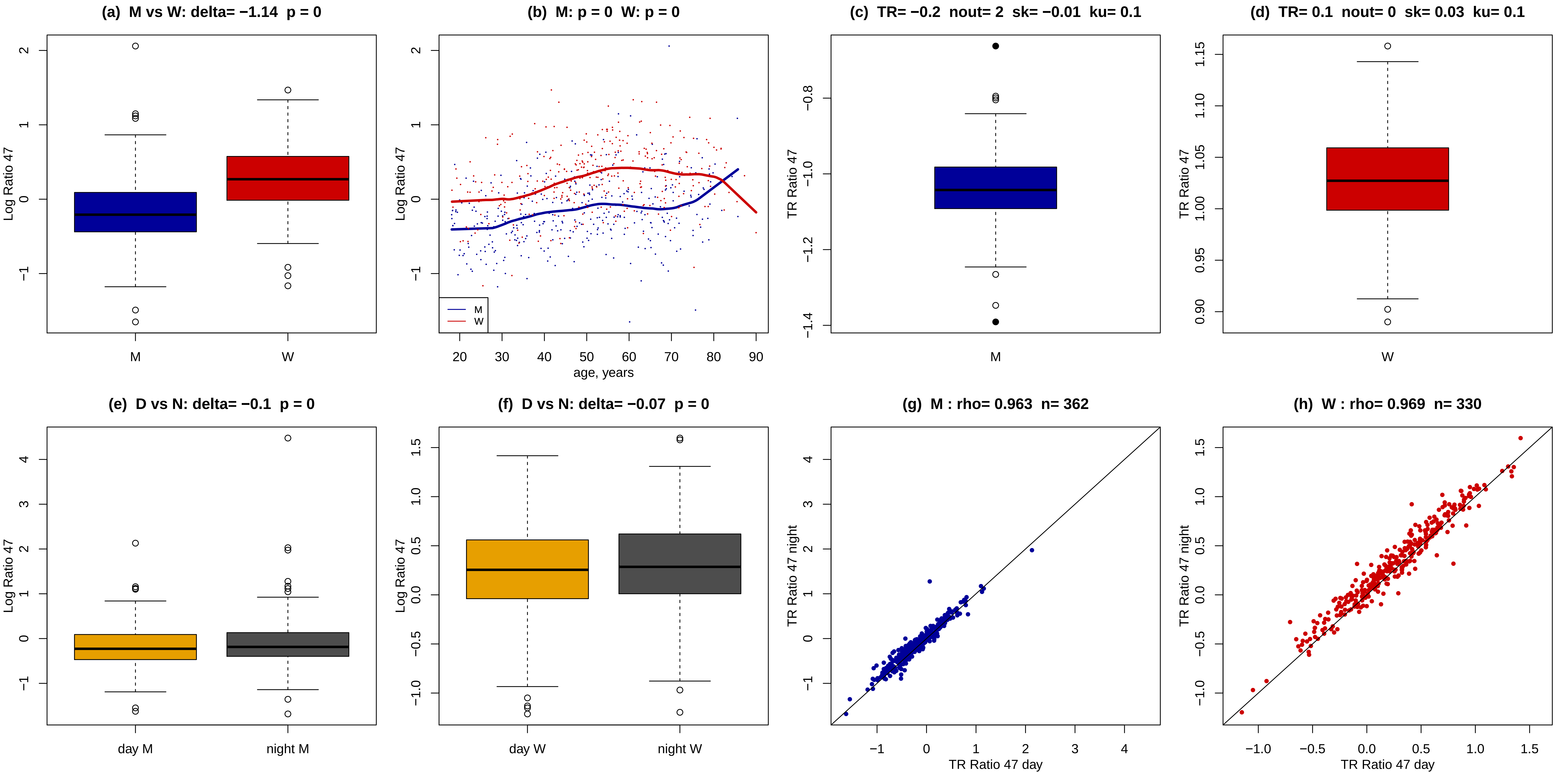
(a) M vs W: delta= −1.14 p = 0
Log Ratio 47
M
W
(b) M: p = 0 W: p = 0
Log Ratio 47
age, years
M
W
(c) TR= −0.2 nout= 2 sk= −0.01 ku= 0.1
TR Ratio 47
M
(d) TR= 0.1 nout= 0 sk= 0.03 ku= 0.1
TR Ratio 47
W
(e) D vs N: delta= −0.1 p = 0
Log Ratio 47
day M
night M
(f) D vs N: delta= −0.07 p = 0
Log Ratio 47
day W
night W
(g) M : rho= 0.963 n= 362
TR Ratio 47 night
TR Ratio 47 day
(h) W : rho= 0.969 n= 330
TR Ratio 47 night
TR Ratio 47 day

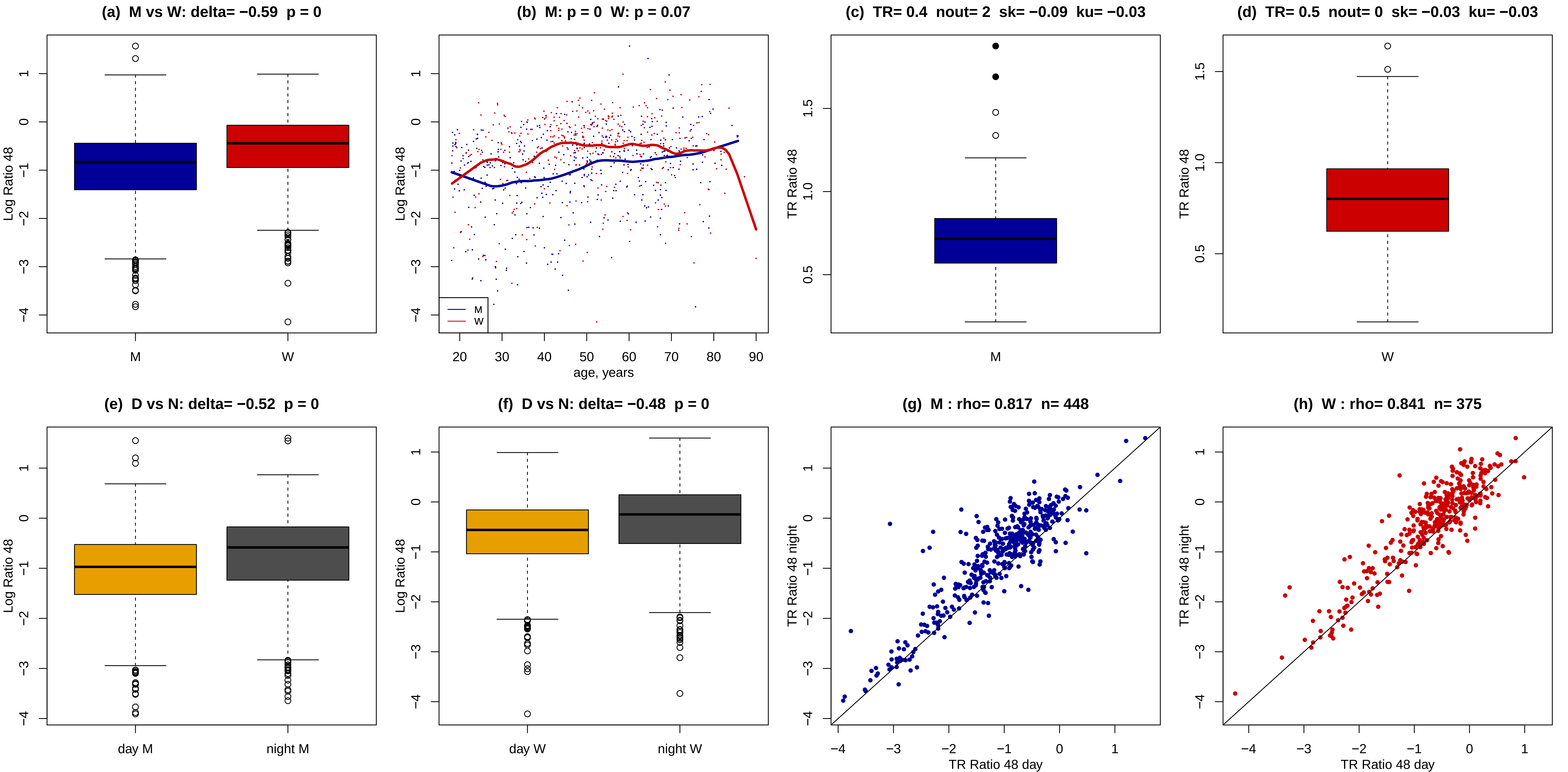
(a) M vs W: delta= −0.59 p = 0
(b) M: p = 0 W: p = 0.07
(c) TR= 0.4 nout= 2 sk= −0.09 ku= −0.03
(d) TR= 0.5 nout= 0 sk= −0.03 ku= −0.03
(e) D vs N: delta= −0.52 p = 0
(f) D vs N: delta= −0.48 p = 0
(g) M : rho= 0.817 n= 448
(h) W : rho= 0.841 n= 375
Log Ratio 48
TR Ratio 48
TR Ratio 48 night
TR Ratio 48 day
age, years
M
W
day M
night M
day W
night W

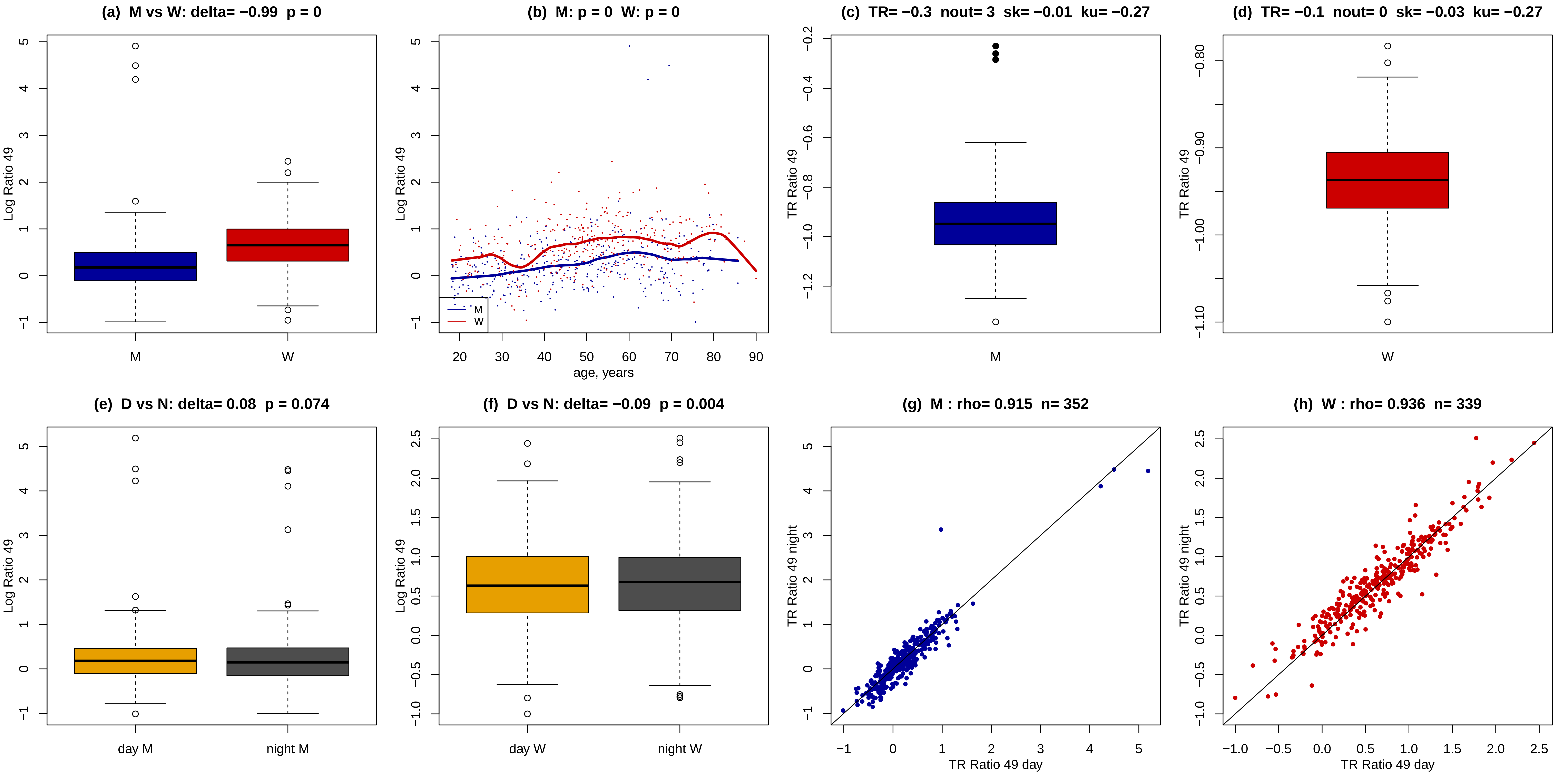

(a) M vs W: delta= −0.99 p = 0
Log Ratio 49
M
W
(b) M: p = 0 W: p = 0
Log Ratio 49
age, years
M
W
(c) TR= −0.3 nout= 3 sk= −0.01 ku= −0.27
TR Ratio 49
M
(d) TR= −0.1 nout= 0 sk= −0.03 ku= −0.27
TR Ratio 49
W
(e) D vs N: delta= 0.08 p = 0.074
Log Ratio 49
day M
night M
(f) D vs N: delta= −0.09 p = 0.004
Log Ratio 49
day W
night W
(g) M : rho= 0.915 n= 352
TR Ratio 49 night
TR Ratio 49 day
(h) W : rho= 0.936 n= 339
TR Ratio 49 night
TR Ratio 49 day

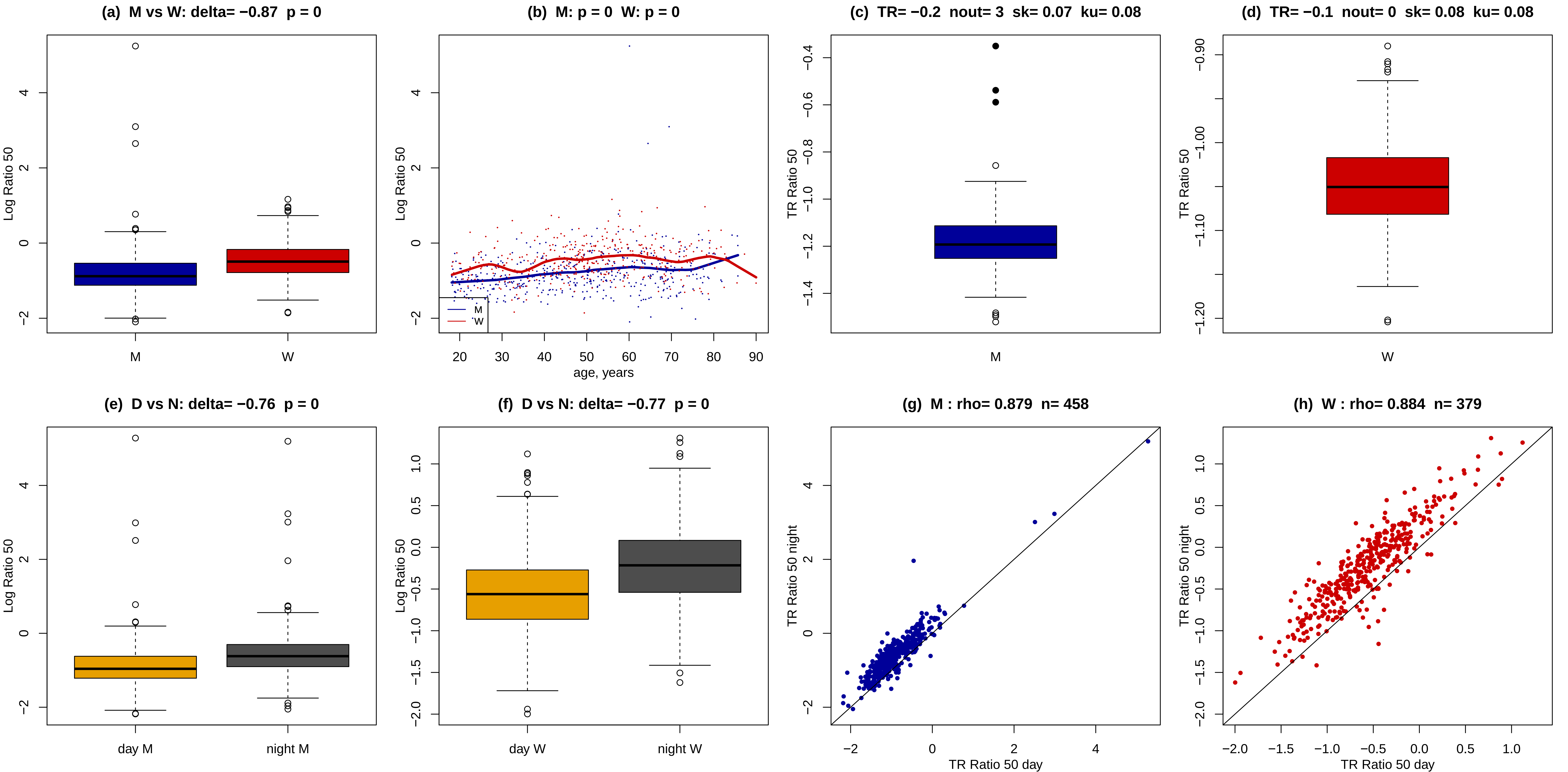
(a) M vs W: delta= −0.87 p = 0
(b) M: p = 0 W: p = 0
(c) TR= −0.2 nout= 3 sk= 0.07 ku= 0.08
(d) TR= −0.1 nout= 0 sk= 0.08 ku= 0.08
Log Ratio 50
TR Ratio 50
M
W
age, years
(e) D vs N: delta= −0.76 p = 0
(f) D vs N: delta= −0.77 p = 0
(g) M : rho= 0.879 n= 458
(h) W : rho= 0.884 n= 379
day M
night M
day W
night W
TR Ratio 50 night
TR Ratio 50 day

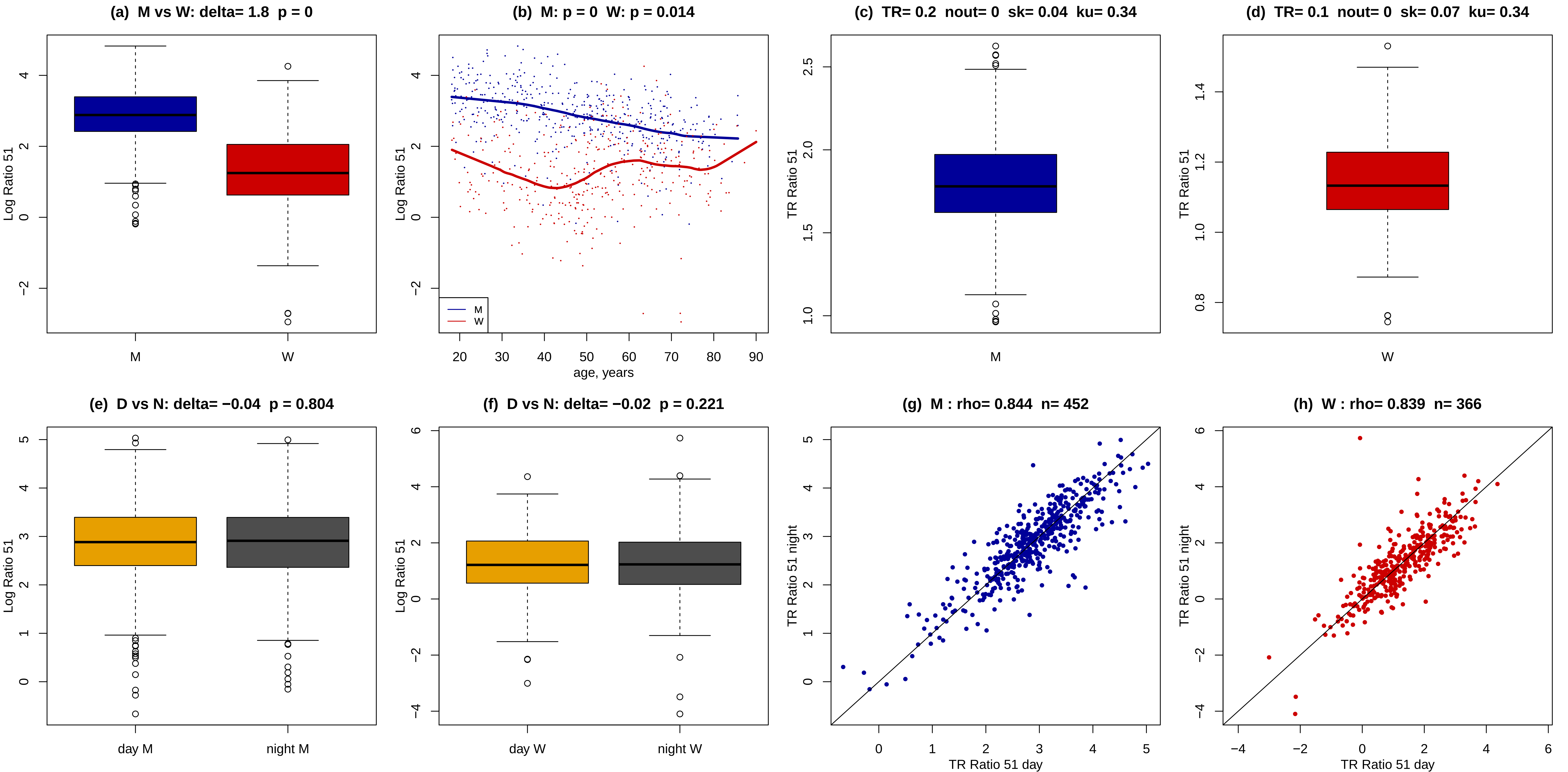
(a) M vs W: delta= 1.8 p = 0
Log Ratio 51
M
W
(b) M: p = 0 W: p = 0.014
Log Ratio 51
age, years
M
W
(c) TR= 0.2 nout= 0 sk= 0.04 ku= 0.34
TR Ratio 51
M
(d) TR= 0.1 nout= 0 sk= 0.07 ku= 0.34
TR Ratio 51
W
(e) D vs N: delta= −0.04 p = 0.804
Log Ratio 51
day M
night M
(f) D vs N: delta= −0.02 p = 0.221
Log Ratio 51
day W
night W
(g) M : rho= 0.844 n= 452
TR Ratio 51 night
TR Ratio 51 day
(h) W : rho= 0.839 n= 366
TR Ratio 51 night
TR Ratio 51 day

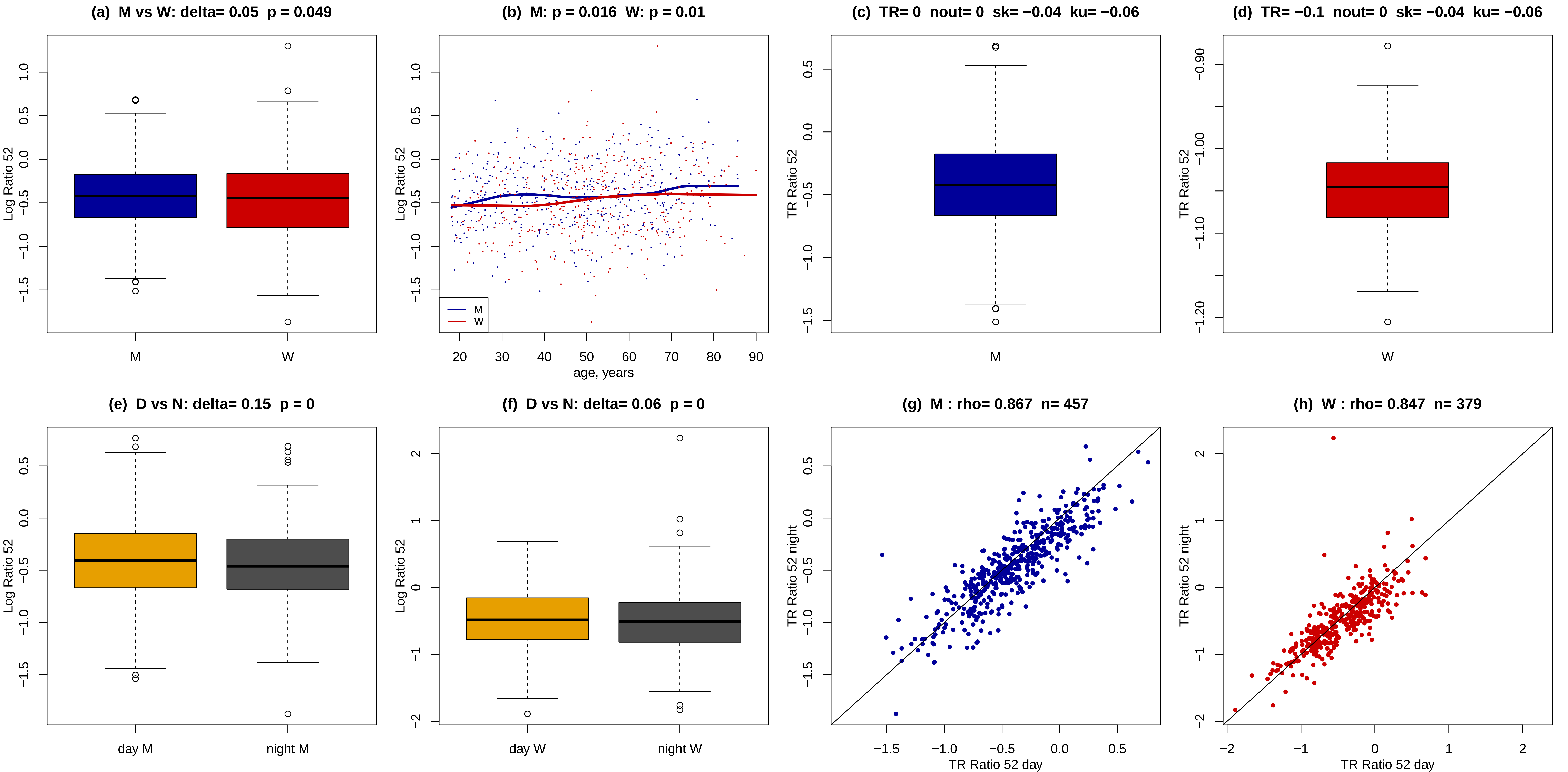
(a) M vs W: delta= 0.05 p = 0.049
Log Ratio 52
M
W
(b) M: p = 0.016 W: p = 0.01
Log Ratio 52
age, years
M
W
(c) TR= 0 nout= 0 sk= −0.04 ku= −0.06
TR Ratio 52
M
(d) TR= −0.1 nout= 0 sk= −0.04 ku= −0.06
TR Ratio 52
W
(e) D vs N: delta= 0.15 p = 0
Log Ratio 52
day M
night M
(f) D vs N: delta= 0.06 p = 0
Log Ratio 52
day W
night W
(g) M : rho= 0.867 n= 457
TR Ratio 52 night
TR Ratio 52 day
(h) W : rho= 0.847 n= 379
TR Ratio 52 night
TR Ratio 52 day

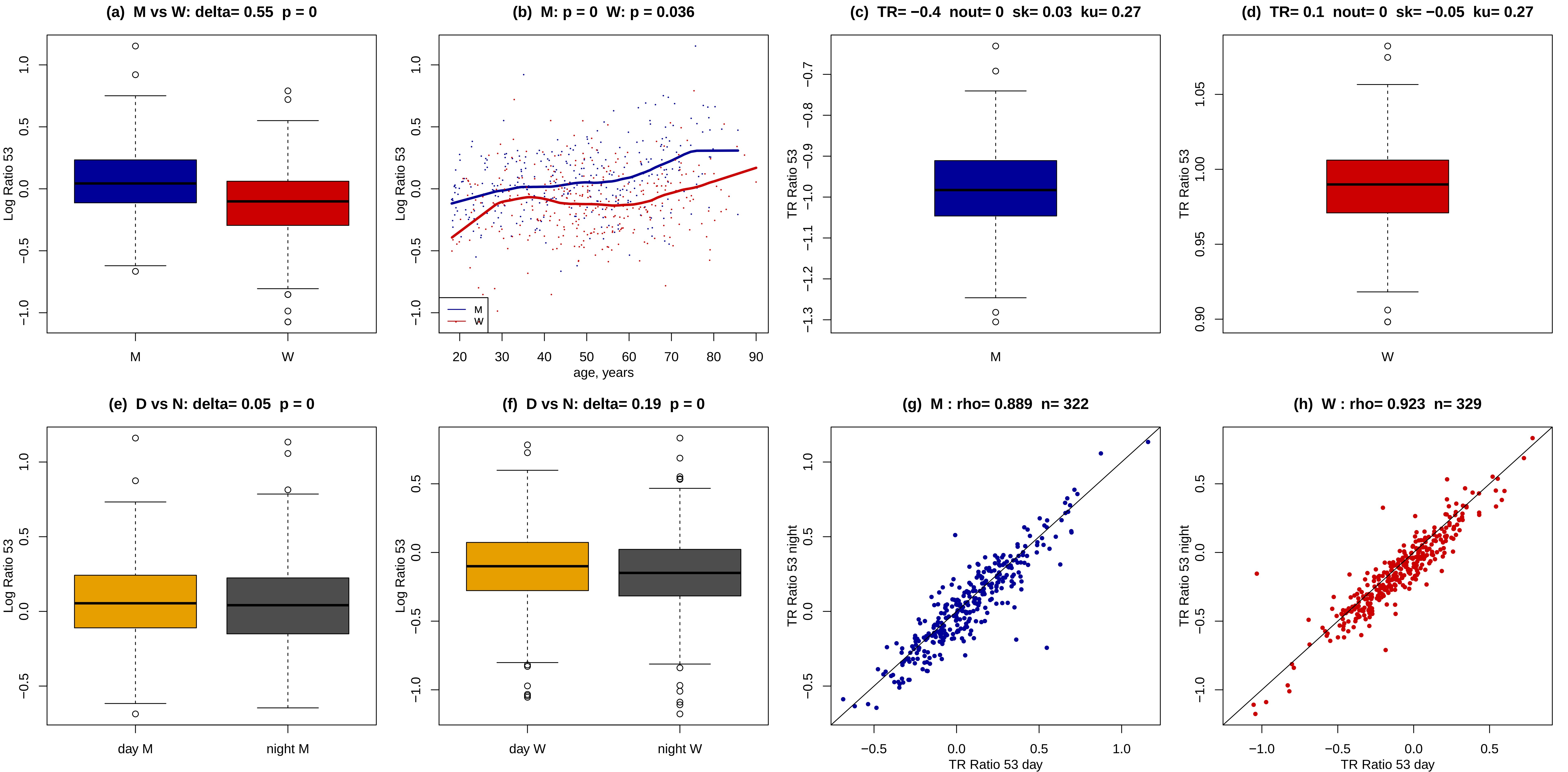
(a) M vs W: delta= 0.55 p = 0
(b) M: p = 0 W: p = 0.036
(c) TR= −0.4 nout= 0 sk= 0.03 ku= 0.27
(d) TR= 0.1 nout= 0 sk= −0.05 ku= 0.27
Log Ratio 53
M
W
age, years
TR Ratio 53
(e) D vs N: delta= 0.05 p = 0
(f) D vs N: delta= 0.19 p = 0
(g) M : rho= 0.889 n= 322
(h) W : rho= 0.923 n= 329
day M
night M
day W
night W
TR Ratio 53 night
TR Ratio 53 day

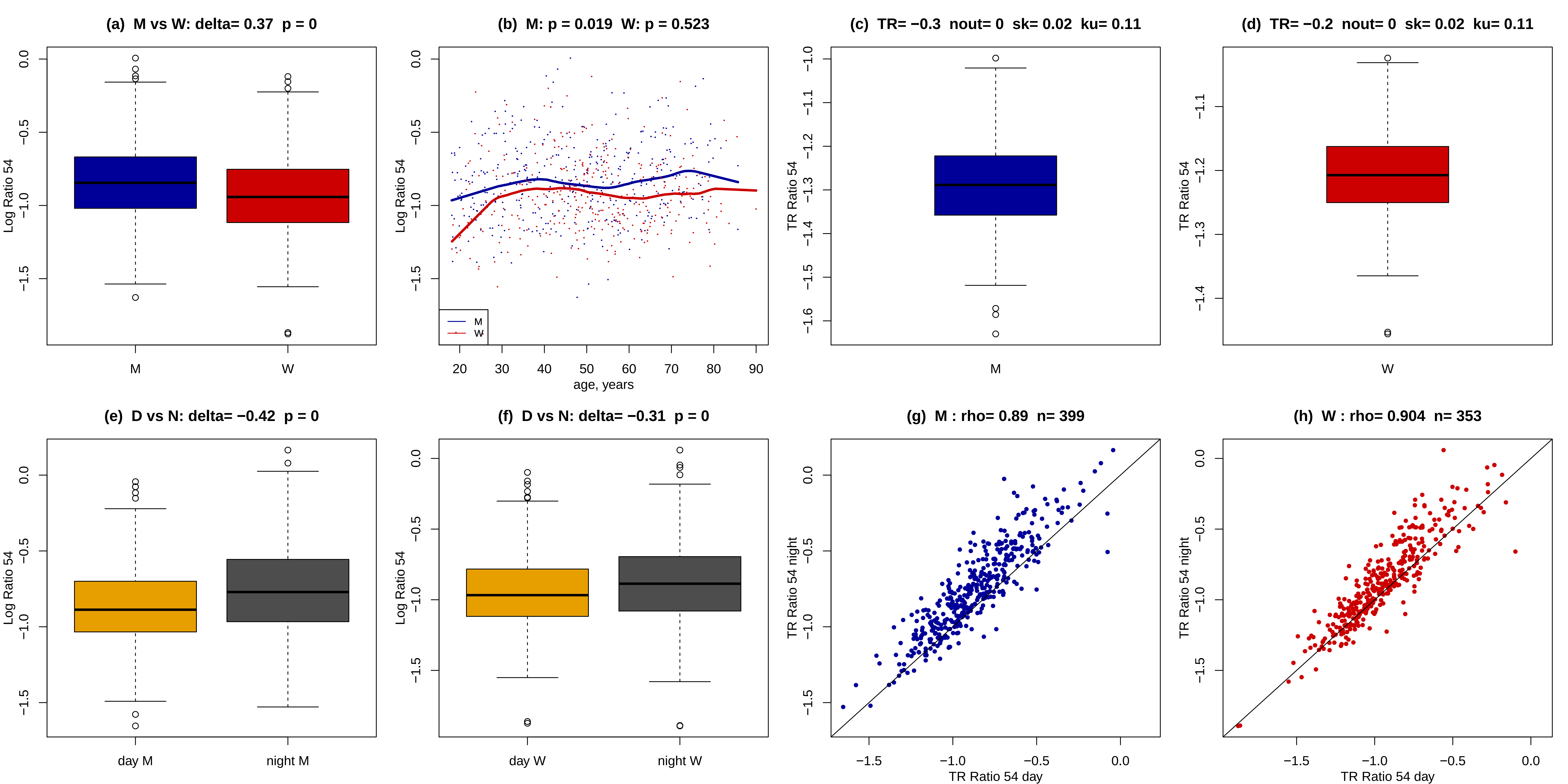

(a) M vs W: delta= 0.37 p = 0
(b) M: p = 0.019 W: p = 0.523
(c) TR= −0.3 nout= 0 sk= 0.02 ku= 0.11
(d) TR= −0.2 nout= 0 sk= 0.02 ku= 0.11
Log Ratio 54
TR Ratio 54
M
W
age, years
(e) D vs N: delta= −0.42 p = 0
(f) D vs N: delta= −0.31 p = 0
(g) M : rho= 0.89 n= 399
(h) W : rho= 0.904 n= 353
day M
night M
day W
night W
TR Ratio 54 night
TR Ratio 54 day

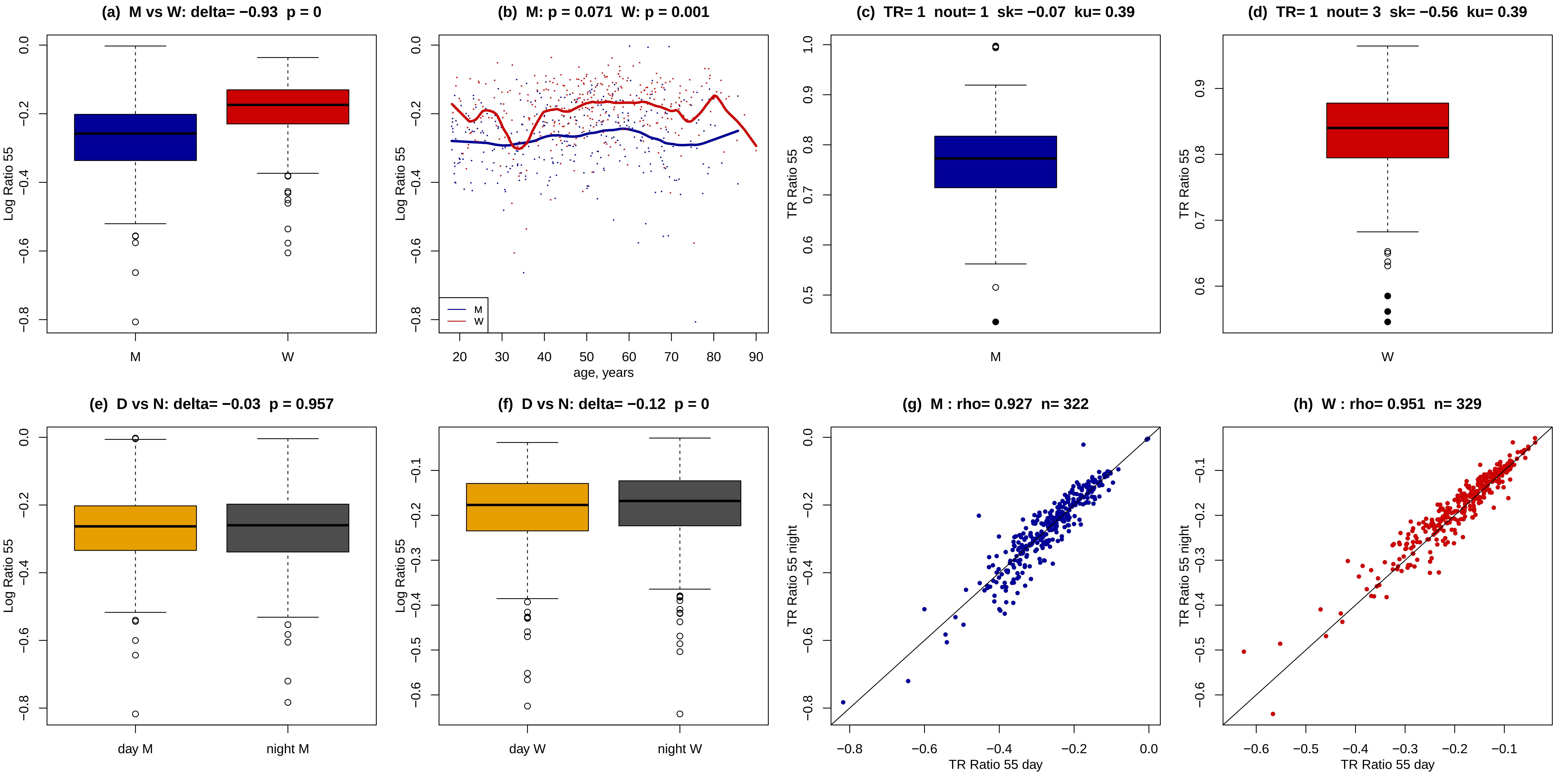
(a) M vs W: delta= −0.93 p = 0
Log Ratio 55
M
W
(b) M: p = 0.071 W: p = 0.001
Log Ratio 55
age, years
M
W
(c) TR= 1 nout= 1 sk= −0.07 ku= 0.39
TR Ratio 55
M
(d) TR= 1 nout= 3 sk= −0.56 ku= 0.39
TR Ratio 55
W
(e) D vs N: delta= −0.03 p = 0.957
Log Ratio 55
day M
night M
(f) D vs N: delta= −0.12 p = 0
Log Ratio 55
day W
night W
(g) M : rho= 0.927 n= 322
TR Ratio 55 night
TR Ratio 55 day
(h) W : rho= 0.951 n= 329
TR Ratio 55 night
TR Ratio 55 day

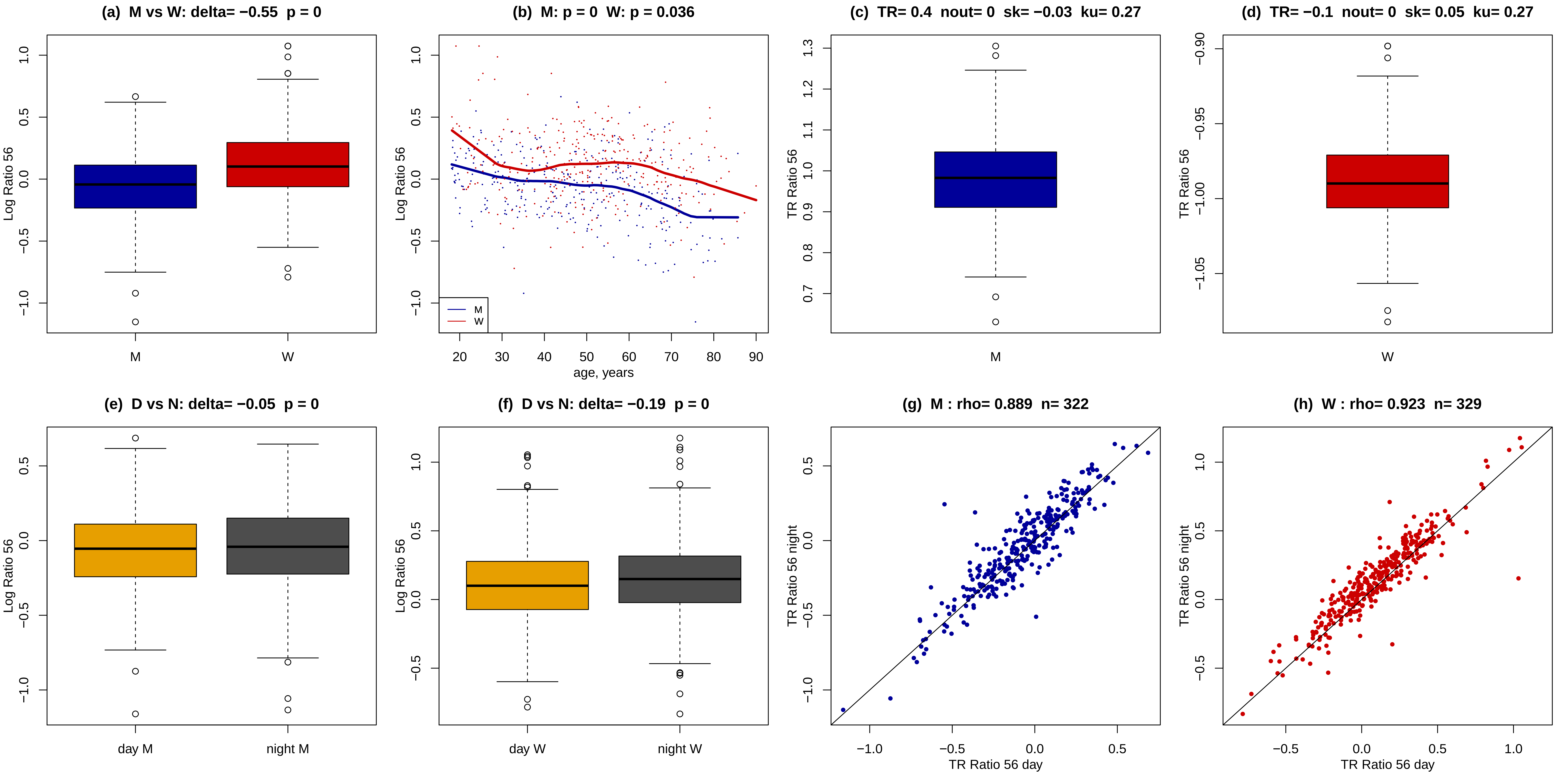

(a) M vs W: delta= −0.55 p = 0
Log Ratio 56
M
W
(b) M: p = 0 W: p = 0.036
Log Ratio 56
age, years
M
W
(c) TR= 0.4 nout= 0 sk= −0.03 ku= 0.27
TR Ratio 56
M
(d) TR= −0.1 nout= 0 sk= 0.05 ku= 0.27
TR Ratio 56
W
(e) D vs N: delta= −0.05 p = 0
Log Ratio 56
day M
night M
(f) D vs N: delta= −0.19 p = 0
Log Ratio 56
day W
night W
(g) M : rho= 0.889 n= 322
TR Ratio 56 night
TR Ratio 56 day
(h) W : rho= 0.923 n= 329
TR Ratio 56 night
TR Ratio 56 day

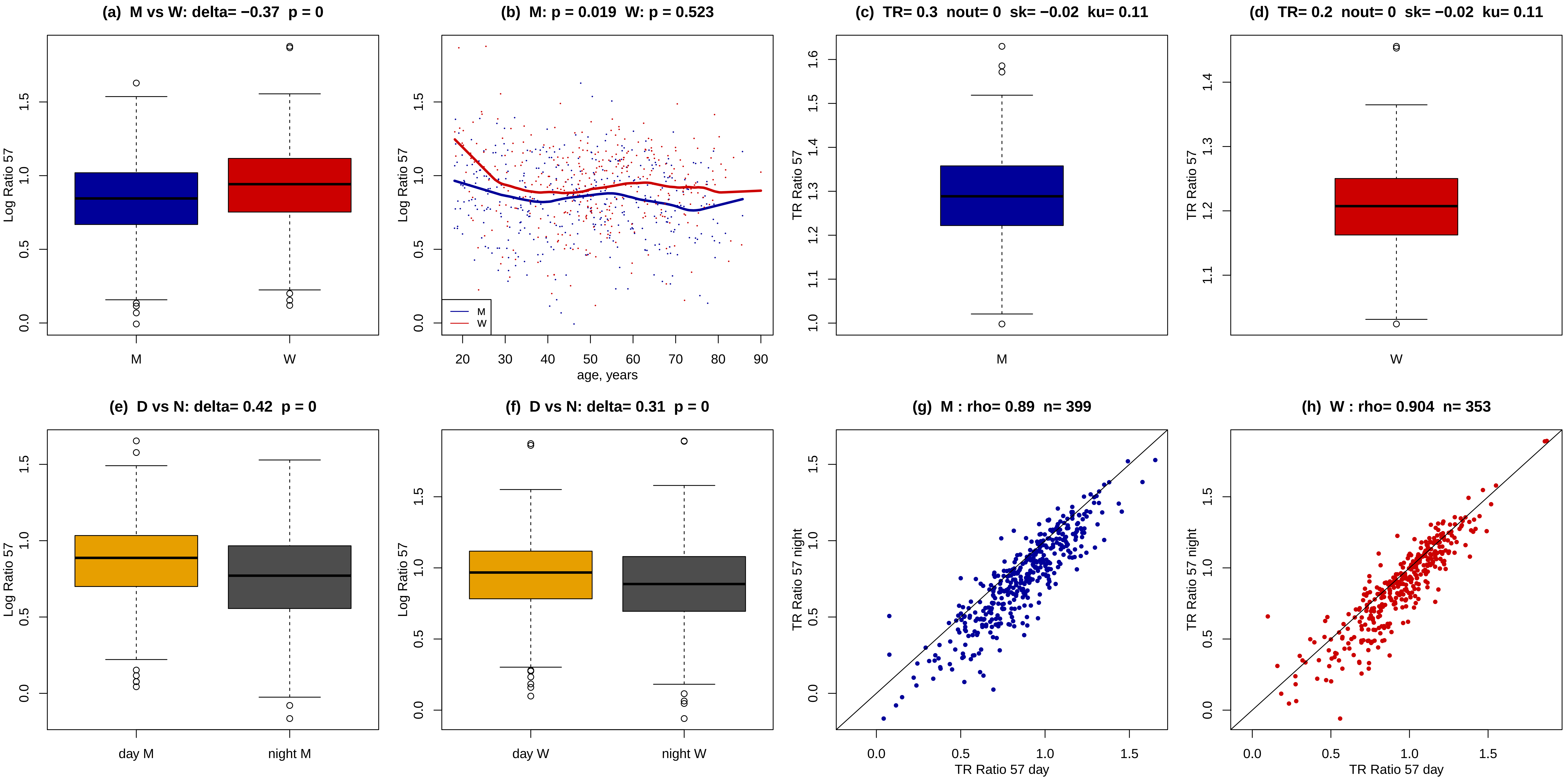

(a) M vs W: delta= −0.37 p = 0
Log Ratio 57
M
W
(b) M: p = 0.019 W: p = 0.523
Log Ratio 57
age, years
M
W
(c) TR= 0.3 nout= 0 sk= −0.02 ku= 0.11
TR Ratio 57
M
(d) TR= 0.2 nout= 0 sk= −0.02 ku= 0.11
TR Ratio 57
W
(e) D vs N: delta= 0.42 p = 0
Log Ratio 57
day M
night M
(f) D vs N: delta= 0.31 p = 0
Log Ratio 57
day W
night W
(g) M : rho= 0.89 n= 399
TR Ratio 57 night
TR Ratio 57 day
(h) W : rho= 0.904 n= 353
TR Ratio 57 night
TR Ratio 57 day

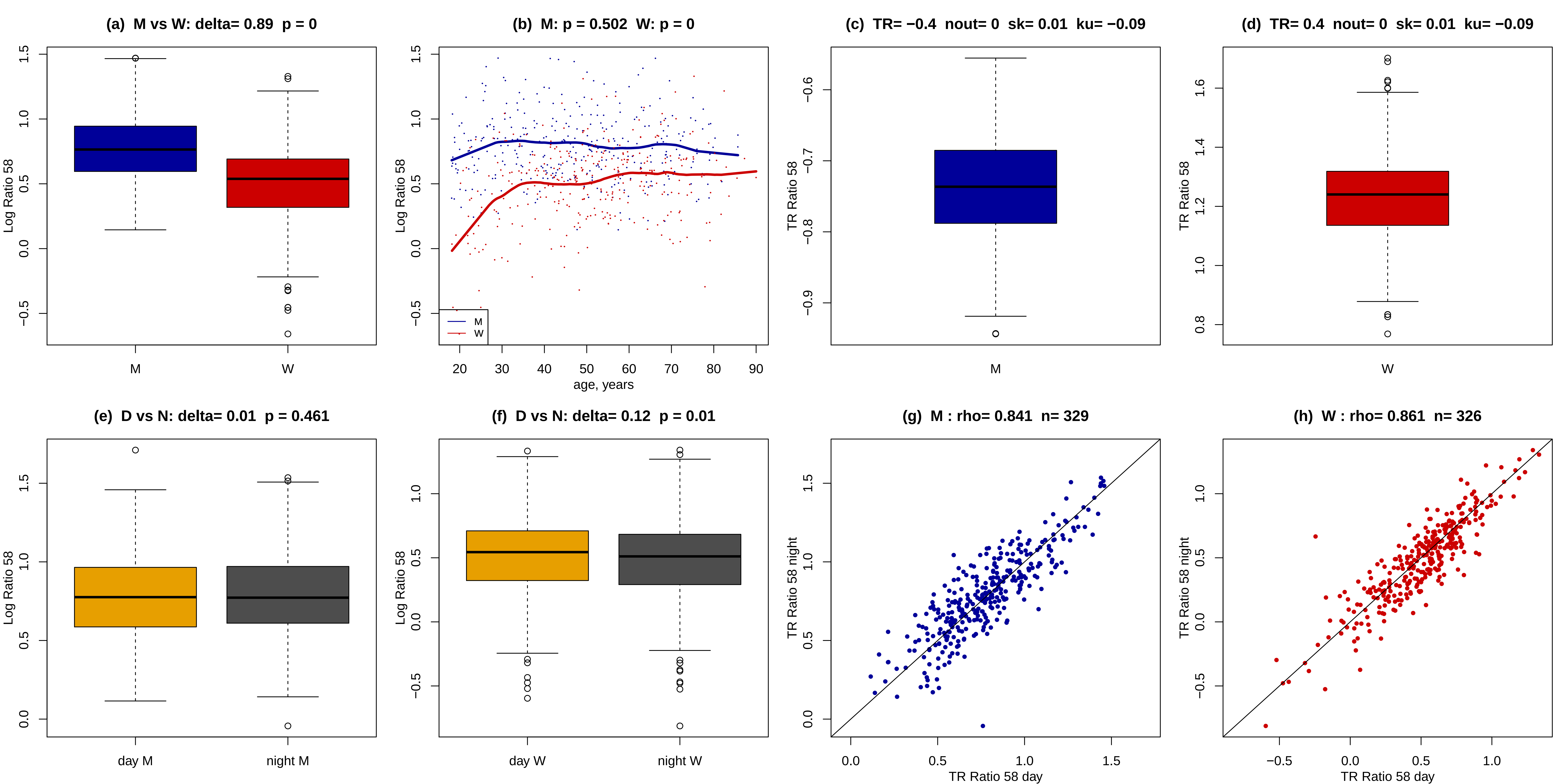
(a) M vs W: delta= 0.89 p = 0
Log Ratio 58
M
W
(b) M: p = 0.502 W: p = 0
Log Ratio 58
age, years
M
W
(c) TR= −0.4 nout= 0 sk= 0.01 ku= −0.09
TR Ratio 58
M
(d) TR= 0.4 nout= 0 sk= 0.01 ku= −0.09
TR Ratio 58
W
(e) D vs N: delta= 0.01 p = 0.461
Log Ratio 58
day M
night M
(f) D vs N: delta= 0.12 p = 0.01
Log Ratio 58
day W
night W
(g) M : rho= 0.841 n= 329
TR Ratio 58 night
TR Ratio 58 day
(h) W : rho= 0.861 n= 326
TR Ratio 58 night
TR Ratio 58 day

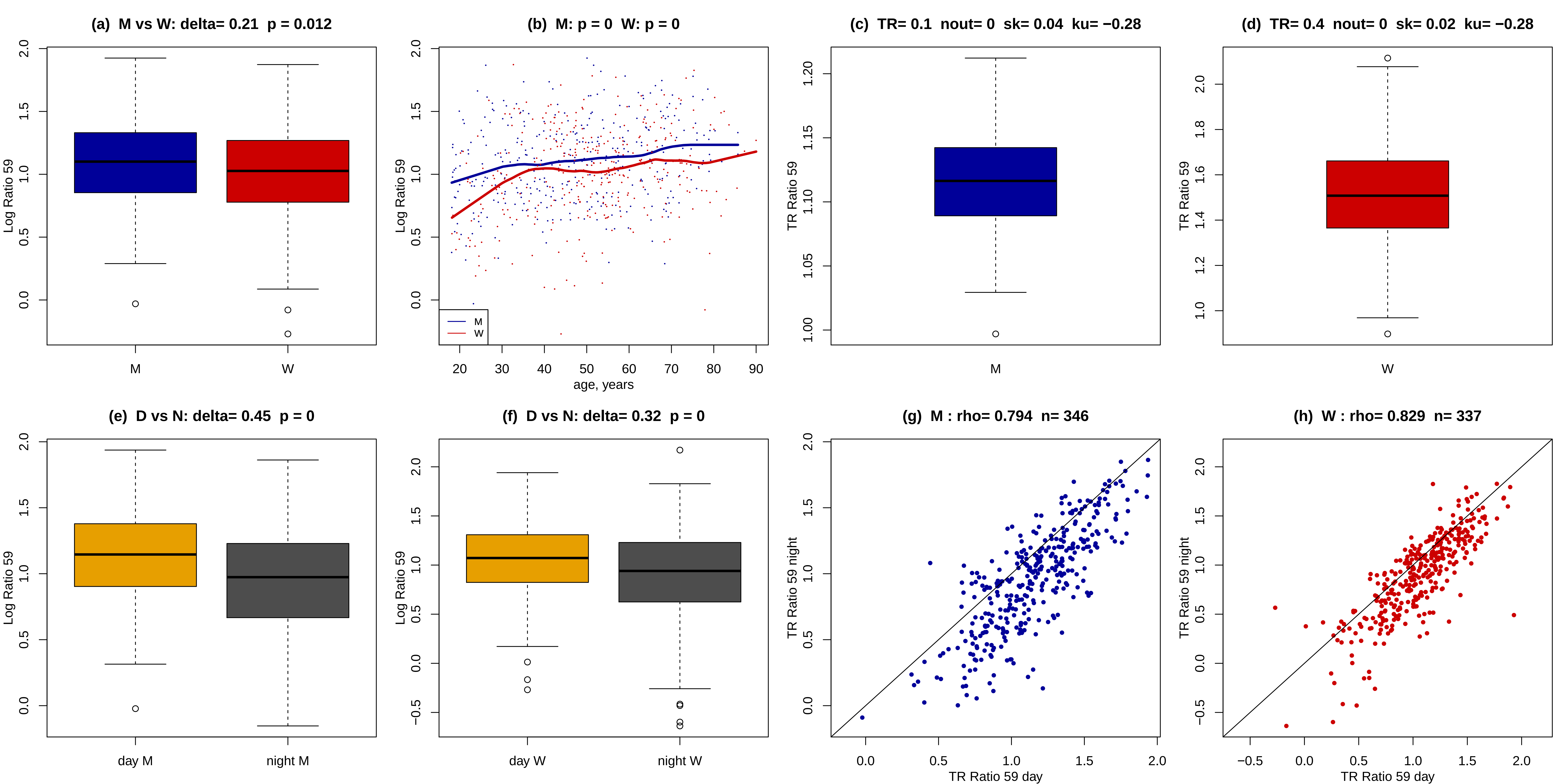
(a) M vs W: delta= 0.21 p = 0.012
Log Ratio 59
M
W
(b) M: p = 0 W: p = 0
Log Ratio 59
age, years
M
W
(c) TR= 0.1 nout= 0 sk= 0.04 ku= −0.28
TR Ratio 59
M
(d) TR= 0.4 nout= 0 sk= 0.02 ku= −0.28
TR Ratio 59
W
(e) D vs N: delta= 0.45 p = 0
Log Ratio 59
day M
night M
(f) D vs N: delta= 0.32 p = 0
Log Ratio 59
day W
night W
(g) M : rho= 0.794 n= 346
TR Ratio 59 night
TR Ratio 59 day
(h) W : rho= 0.829 n= 337
TR Ratio 59 night
TR Ratio 59 day

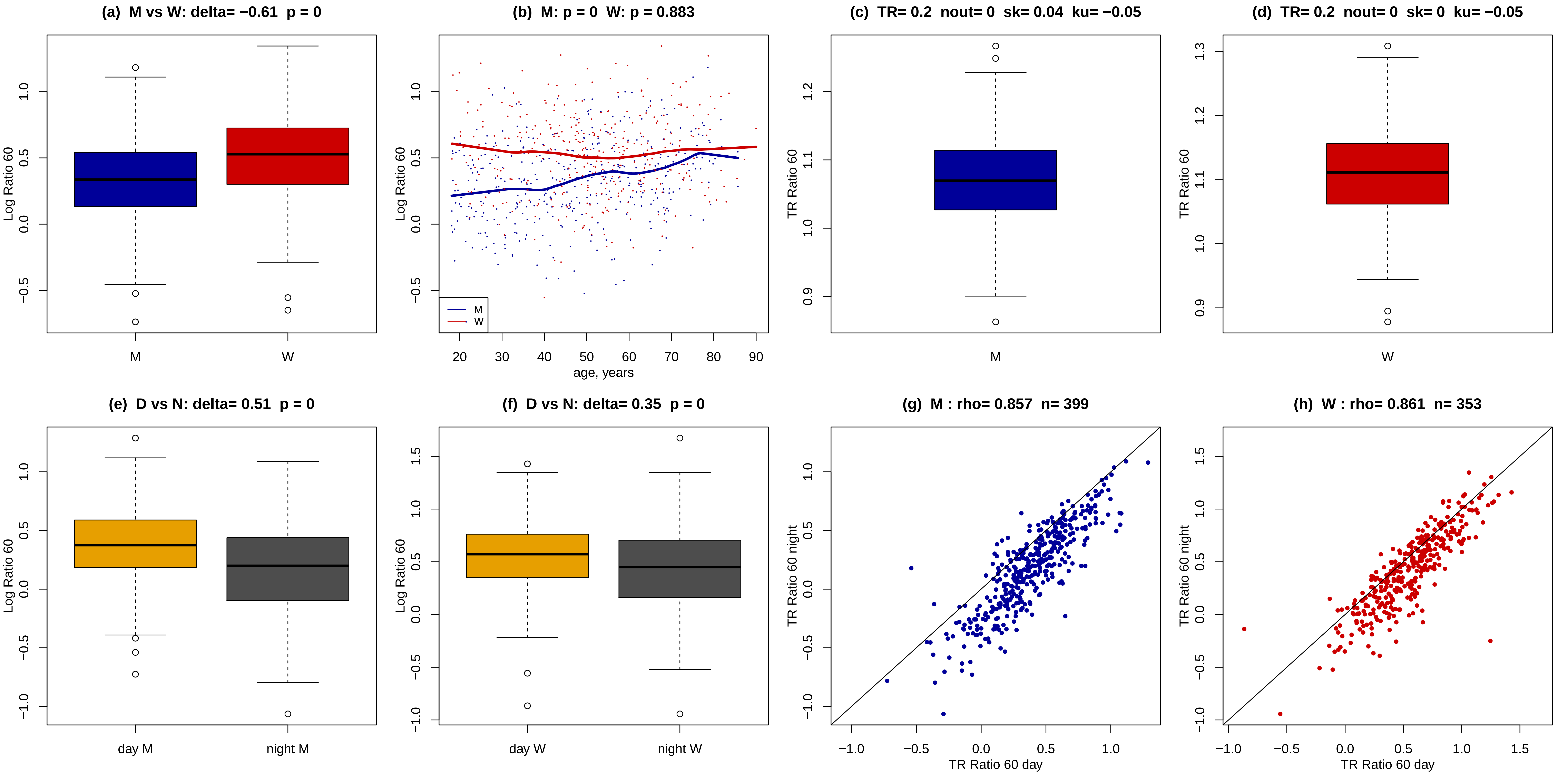

(a) M vs W: delta= −0.61 p = 0
Log Ratio 60
M
W
(b) M: p = 0 W: p = 0.883
Log Ratio 60
age, years
M
W
(c) TR= 0.2 nout= 0 sk= 0.04 ku= −0.05
TR Ratio 60
M
(d) TR= 0.2 nout= 0 sk= 0 ku= −0.05
TR Ratio 60
W
(e) D vs N: delta= 0.51 p = 0
Log Ratio 60
day M
night M
(f) D vs N: delta= 0.35 p = 0
Log Ratio 60
day W
night W
(g) M : rho= 0.857 n= 399
TR Ratio 60 night
TR Ratio 60 day
(h) W : rho= 0.861 n= 353
TR Ratio 60 night
TR Ratio 60 day

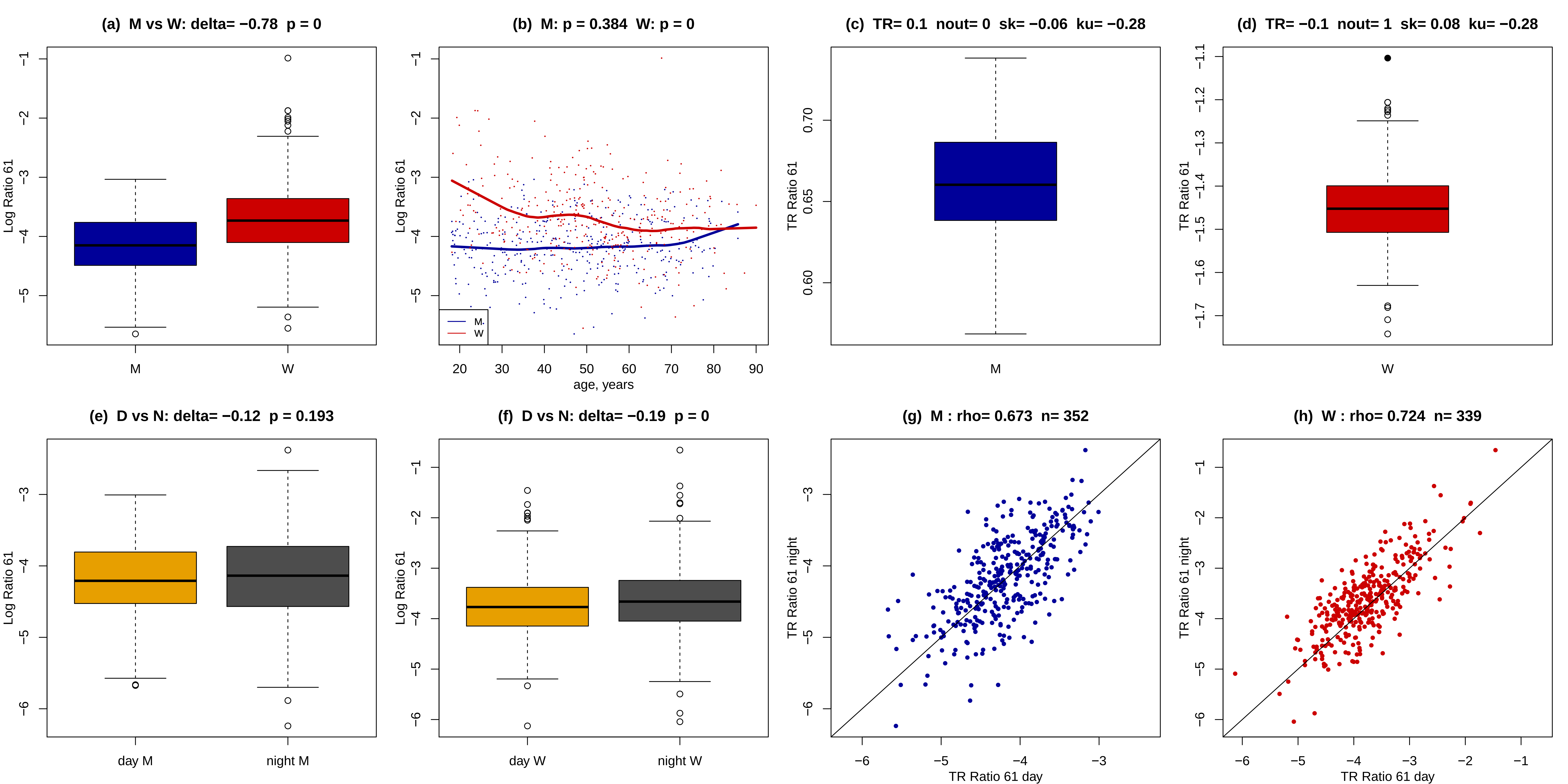

(a) M vs W: delta= −0.78 p = 0
Log Ratio 61
M
W
(b) M: p = 0.384 W: p = 0
Log Ratio 61
M
W
age, years
(c) TR= 0.1 nout= 0 sk= −0.06 ku= −0.28
TR Ratio 61
M
(d) TR= −0.1 nout= 1 sk= 0.08 ku= −0.28
TR Ratio 61
W
(e) D vs N: delta= −0.12 p = 0.193
Log Ratio 61
day M
night M
(f) D vs N: delta= −0.19 p = 0
Log Ratio 61
day W
night W
(g) M : rho= 0.673 n= 352
TR Ratio 61 night
TR Ratio 61 day
(h) W : rho= 0.724 n= 339
TR Ratio 61 night
TR Ratio 61 day

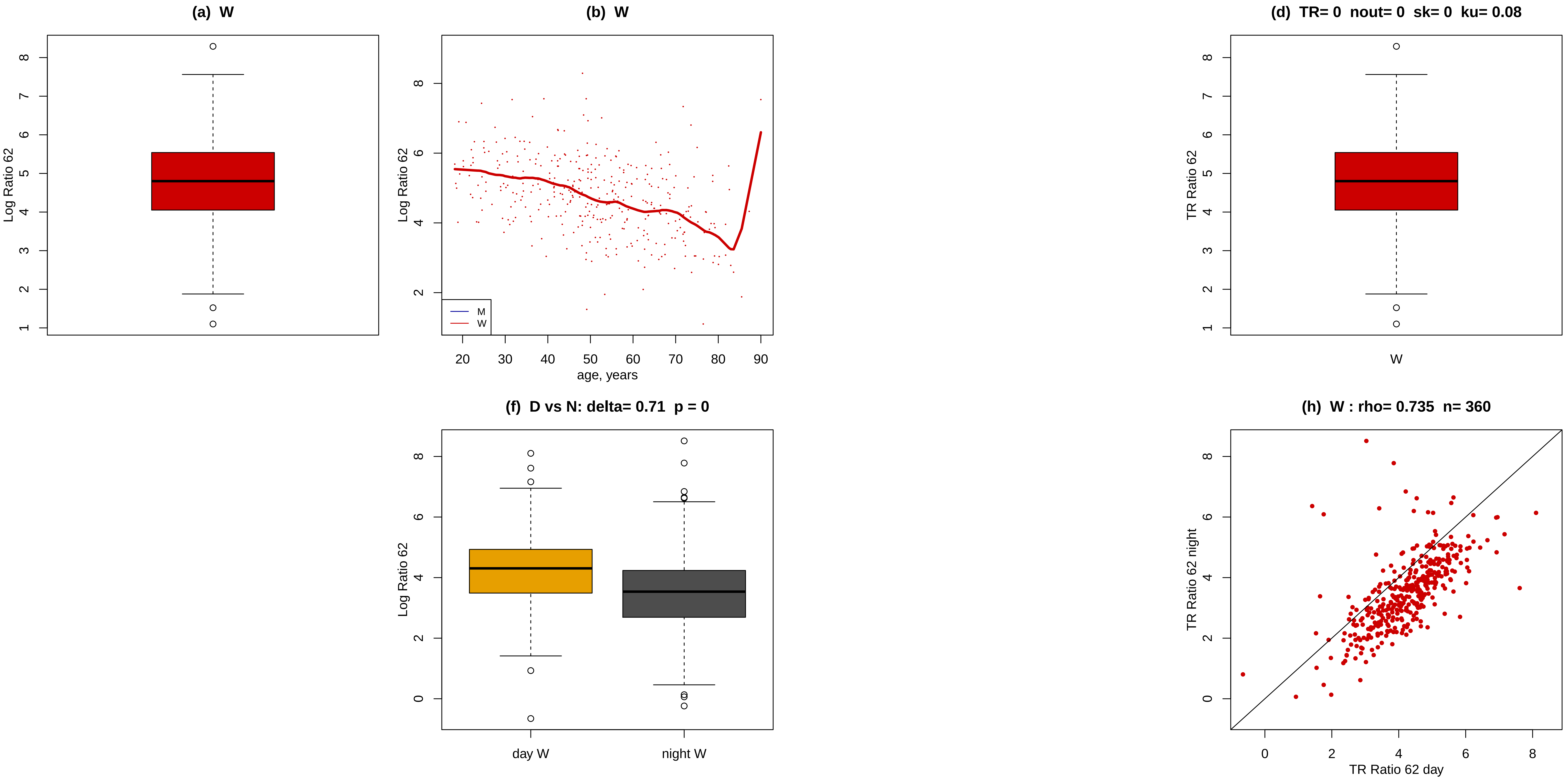

(a) W
Log Ratio 62
(b) W
Log Ratio 62
M
W
age, years
(d) TR= 0 nout= 0 sk= 0 ku= 0.08
TR Ratio 62
W
(f) D vs N: delta= 0.71 p = 0
Log Ratio 62
day W
night W
(h) W : rho= 0.735 n= 360
TR Ratio 62 night
TR Ratio 62 day

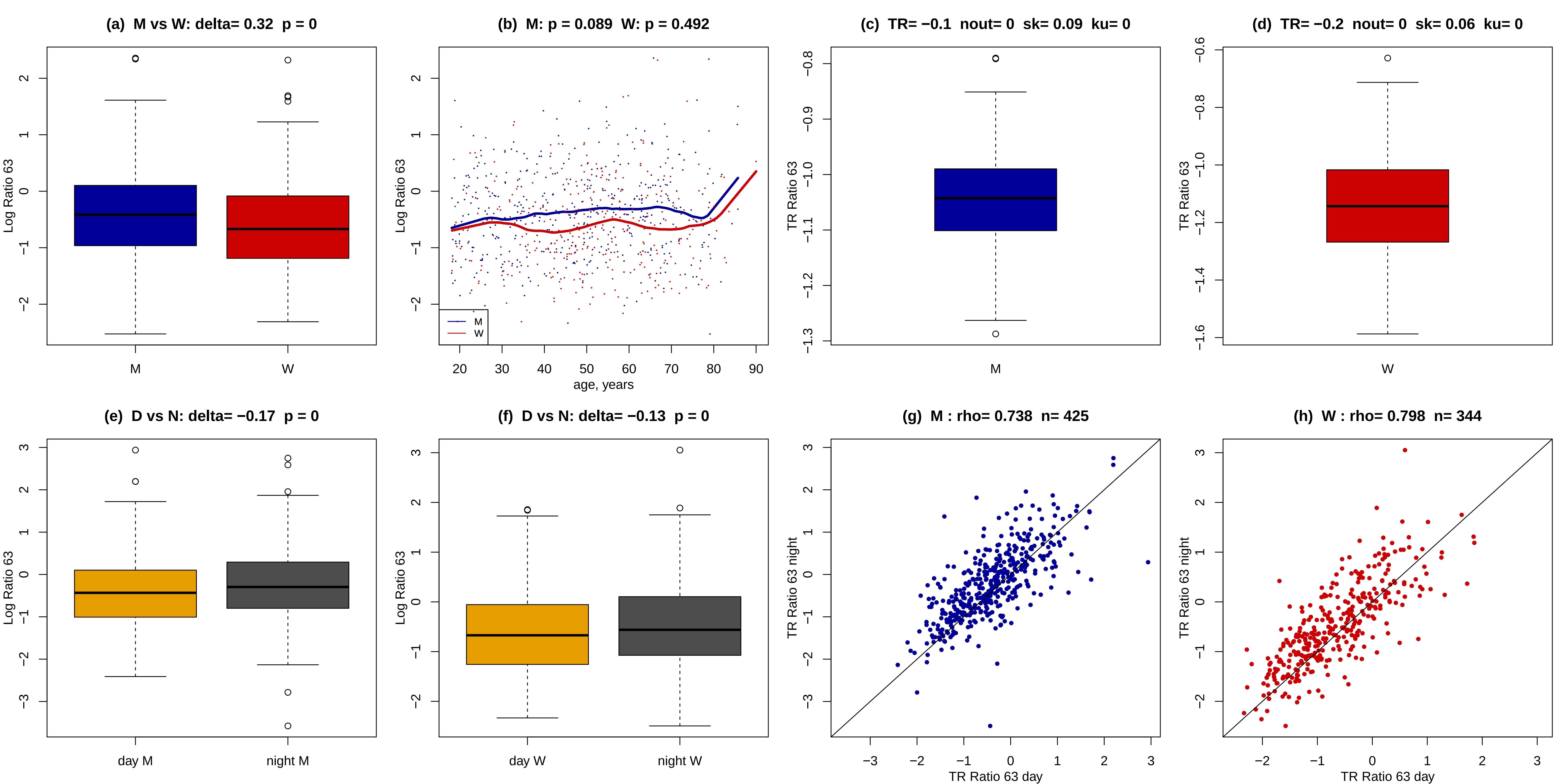

(a) M vs W: delta= 0.32 p = 0
(b) M: p = 0.089 W: p = 0.492
(c) TR= −0.1 nout= 0 sk= 0.09 ku= 0
(d) TR= −0.2 nout= 0 sk= 0.06 ku= 0
(e) D vs N: delta= −0.17 p = 0
(f) D vs N: delta= −0.13 p = 0
(g) M : rho= 0.738 n= 425
(h) W : rho= 0.798 n= 344
Log Ratio 63
TR Ratio 63
age, years
M
W
day M
night M
day W
night W
TR Ratio 63 night
TR Ratio 63 day

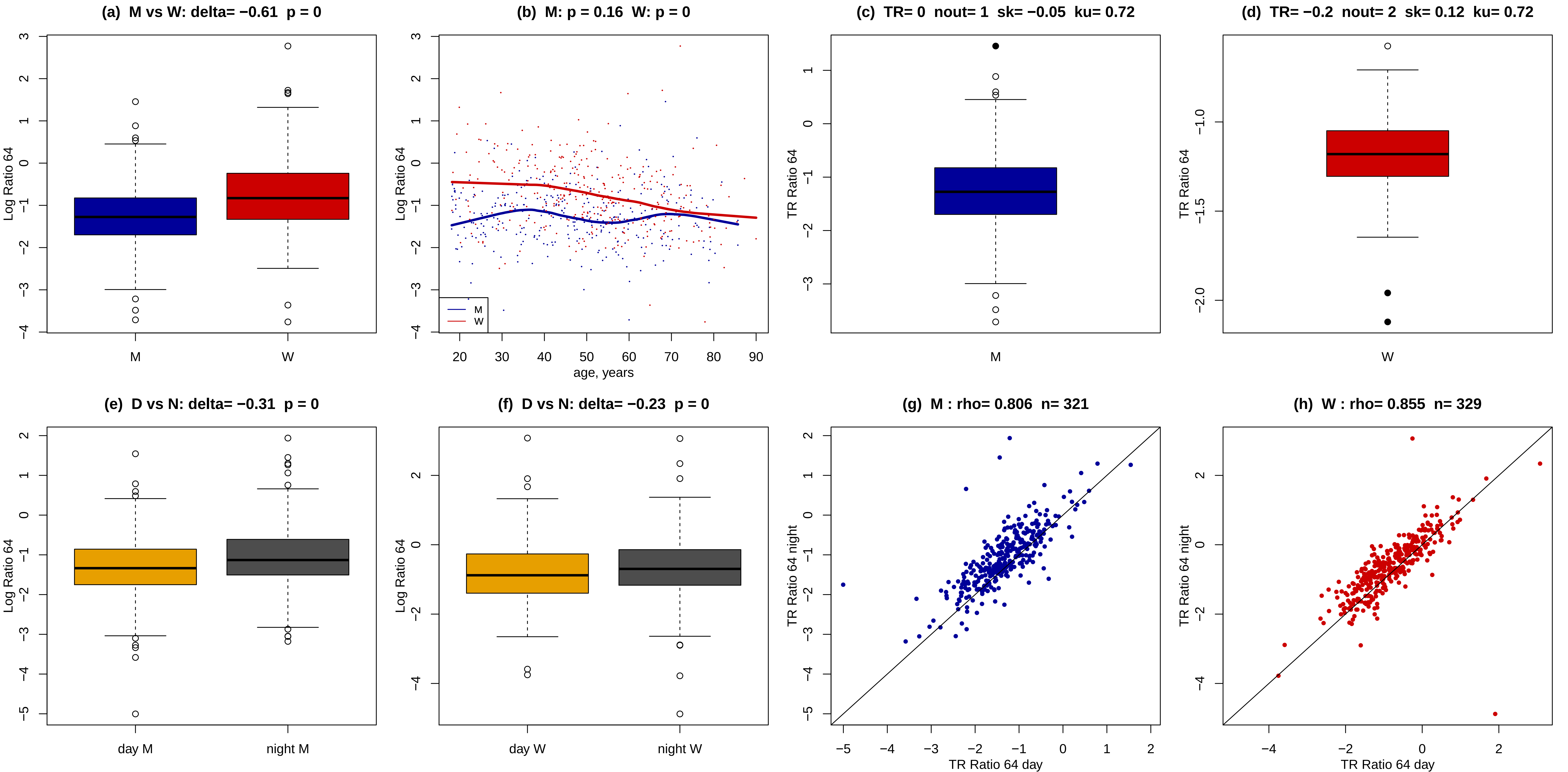
(a) M vs W: delta= −0.61 p = 0
Log Ratio 64
M
W
(b) M: p = 0.16 W: p = 0
Log Ratio 64
age, years
M
W
(c) TR= 0 nout= 1 sk= −0.05 ku= 0.72
TR Ratio 64
M
(d) TR= −0.2 nout= 2 sk= 0.12 ku= 0.72
TR Ratio 64
W
(e) D vs N: delta= −0.31 p = 0
Log Ratio 64
day M
night M
(f) D vs N: delta= −0.23 p = 0
Log Ratio 64
day W
night W
(g) M : rho= 0.806 n= 321
TR Ratio 64 night
TR Ratio 64 day
(h) W : rho= 0.855 n= 329
TR Ratio 64 night
TR Ratio 64 day

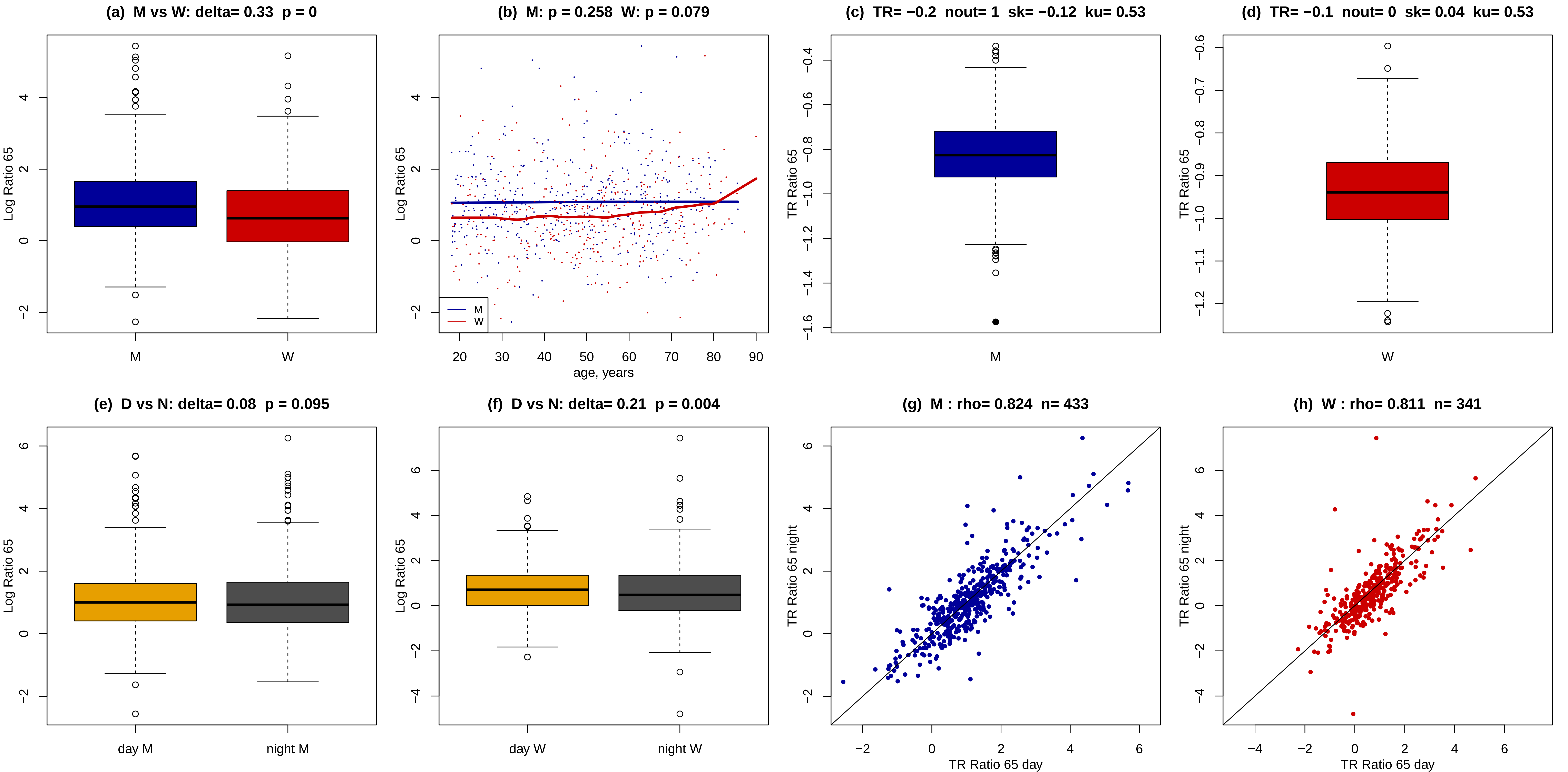
(a) M vs W: delta= 0.33 p = 0
(b) M: p = 0.258 W: p = 0.079
(c) TR= −0.2 nout= 1 sk= −0.12 ku= 0.53
(d) TR= −0.1 nout= 0 sk= 0.04 ku= 0.53
(e) D vs N: delta= 0.08 p = 0.095
(f) D vs N: delta= 0.21 p = 0.004
(g) M : rho= 0.824 n= 433
(h) W : rho= 0.811 n= 341
Log Ratio 65
TR Ratio 65
age, years
M
W
day M
night M
day W
night W
TR Ratio 65 day
TR Ratio 65 night
