# Supplementary material for: Sex- and age-specific reference intervals for diagnostic ratios reflecting relative activity of steroidogenic enzymes and pathways in adults
Source: PLoS One. 2021 Jul 8;16(7):e0253975. doi: 10.1371/journal.pone.0253975 (PMC8266106; doi:10.1371/journal.pone.0253975)
Supplement: S4 Fig — Reference curves of 65 calculated ratios are shown including one ratio per page. The percentiles 2.5, 10, 25, 50, 75, 90 and 97.5 of the ratios in function of age and sex are shown on a log-scale. To facilitate comparison the same scale has been used for men and women. (PDF) [file pone.0253975.s004.pdf]

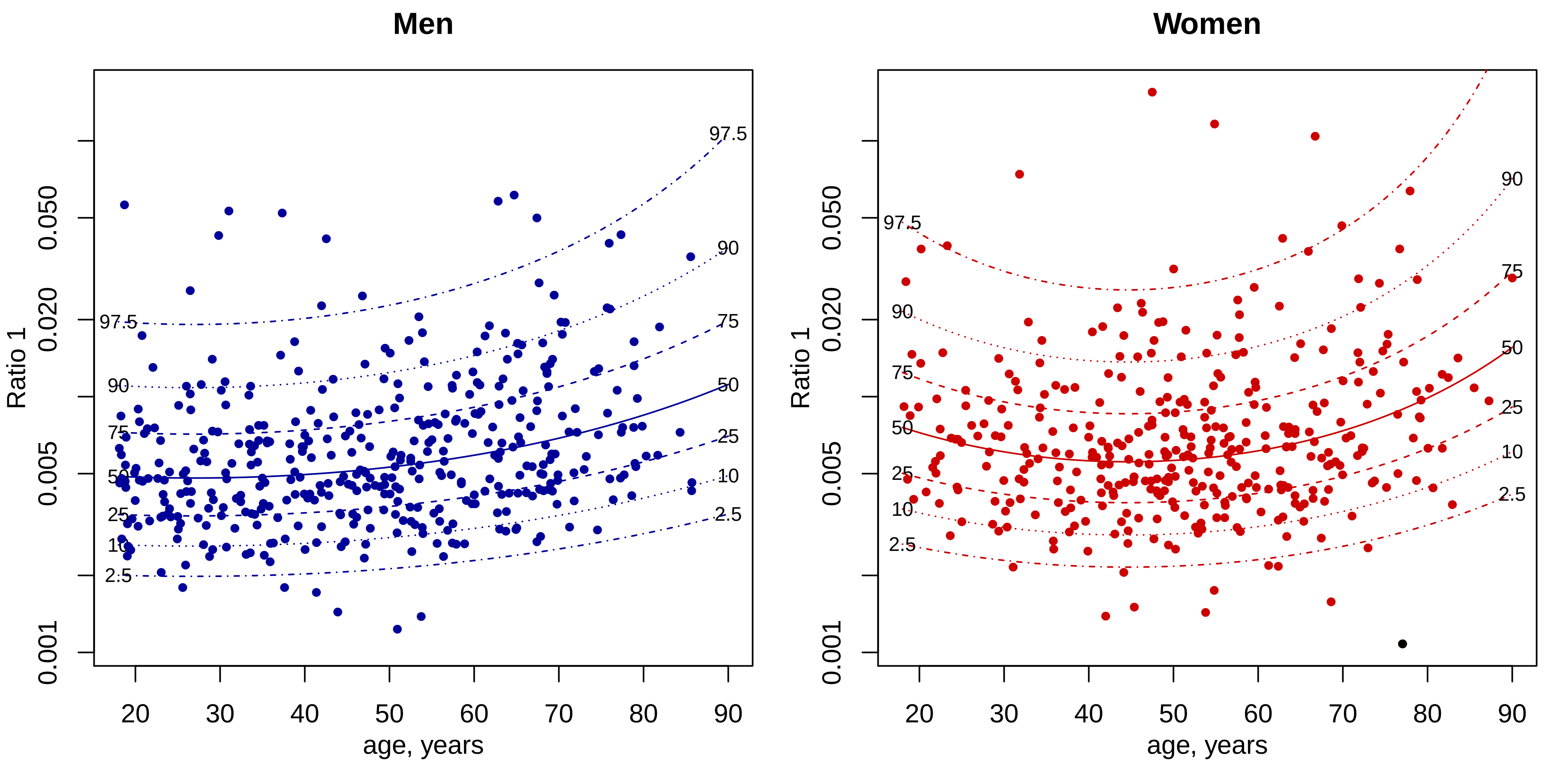

Men
Women
Ratio 1
0.001
0.005
0.020
0.050
20
30
40
50
60
70
80
90
age, years
97.5
90
75
50
25
10
2.5

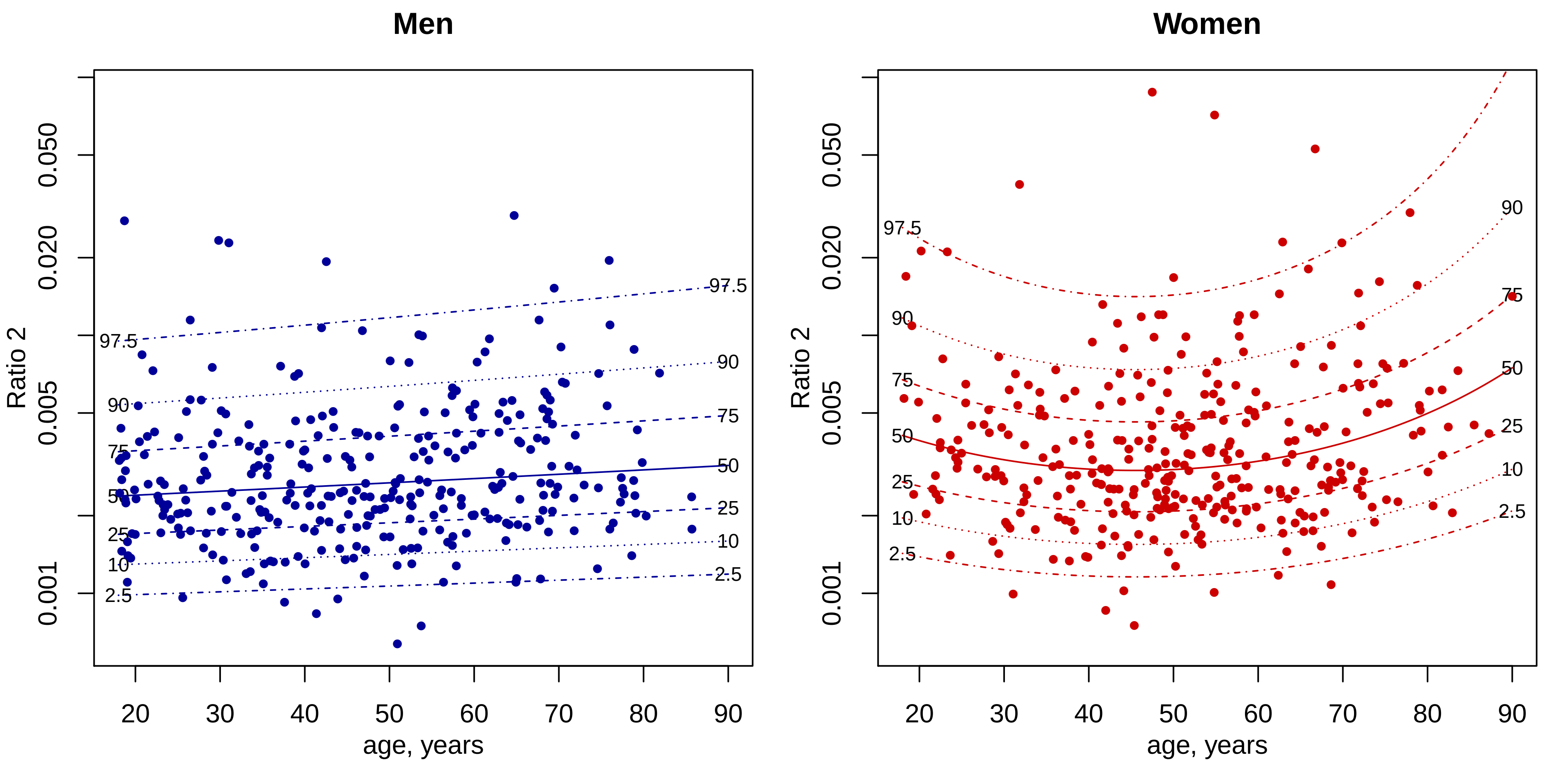
Men
Women
Ratio 2
0.001
0.005
0.020
0.050
age, years
20
30
40
50
60
70
80
90
97.5
90
75
50
25
10
2.5

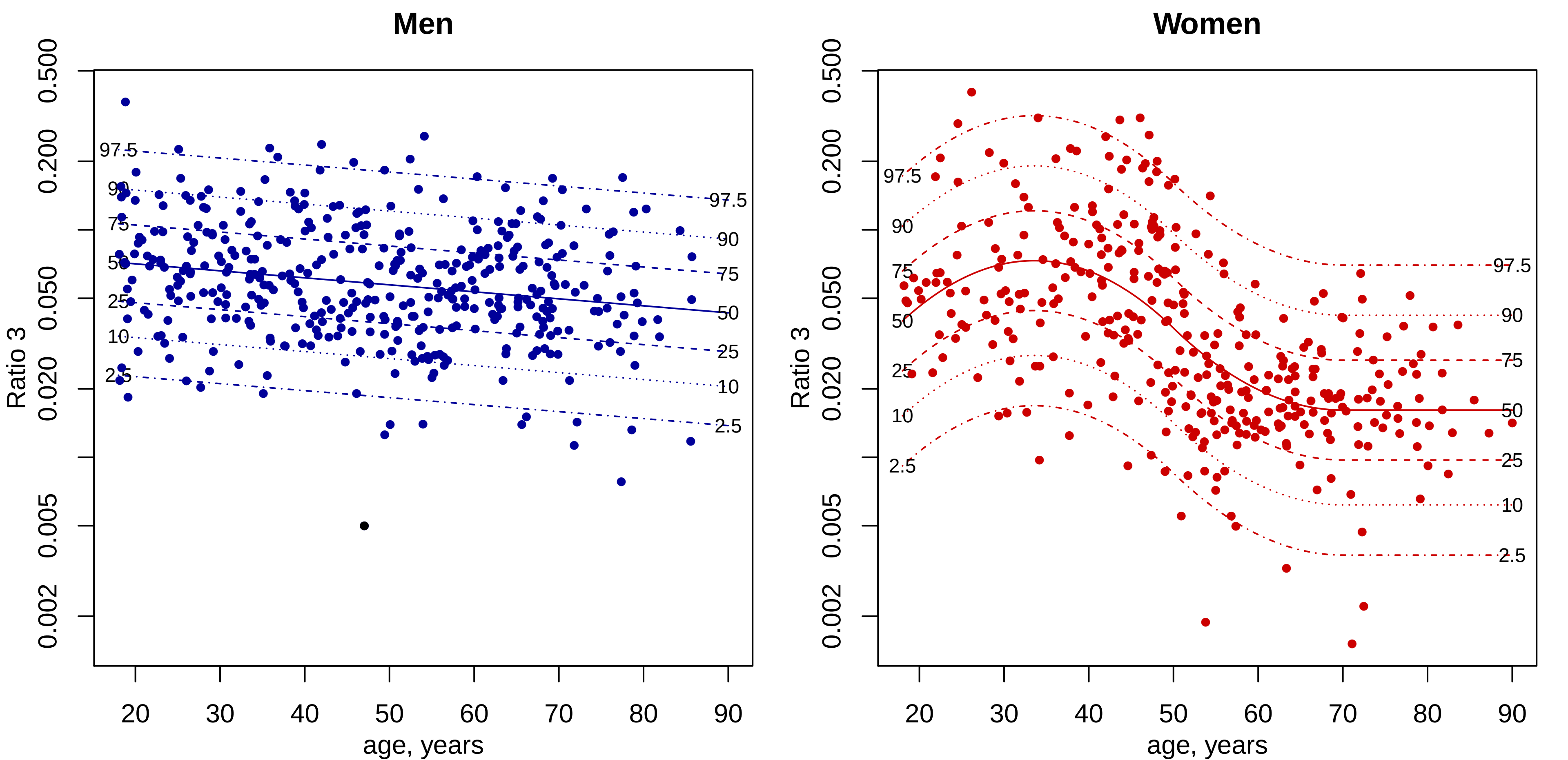

Men
Women
Ratio 3
0.500
0.200
0.050
0.020
0.005
0.002
age, years
20
30
40
50
60
70
80
90
97.5
90
75
50
25
10
2.5

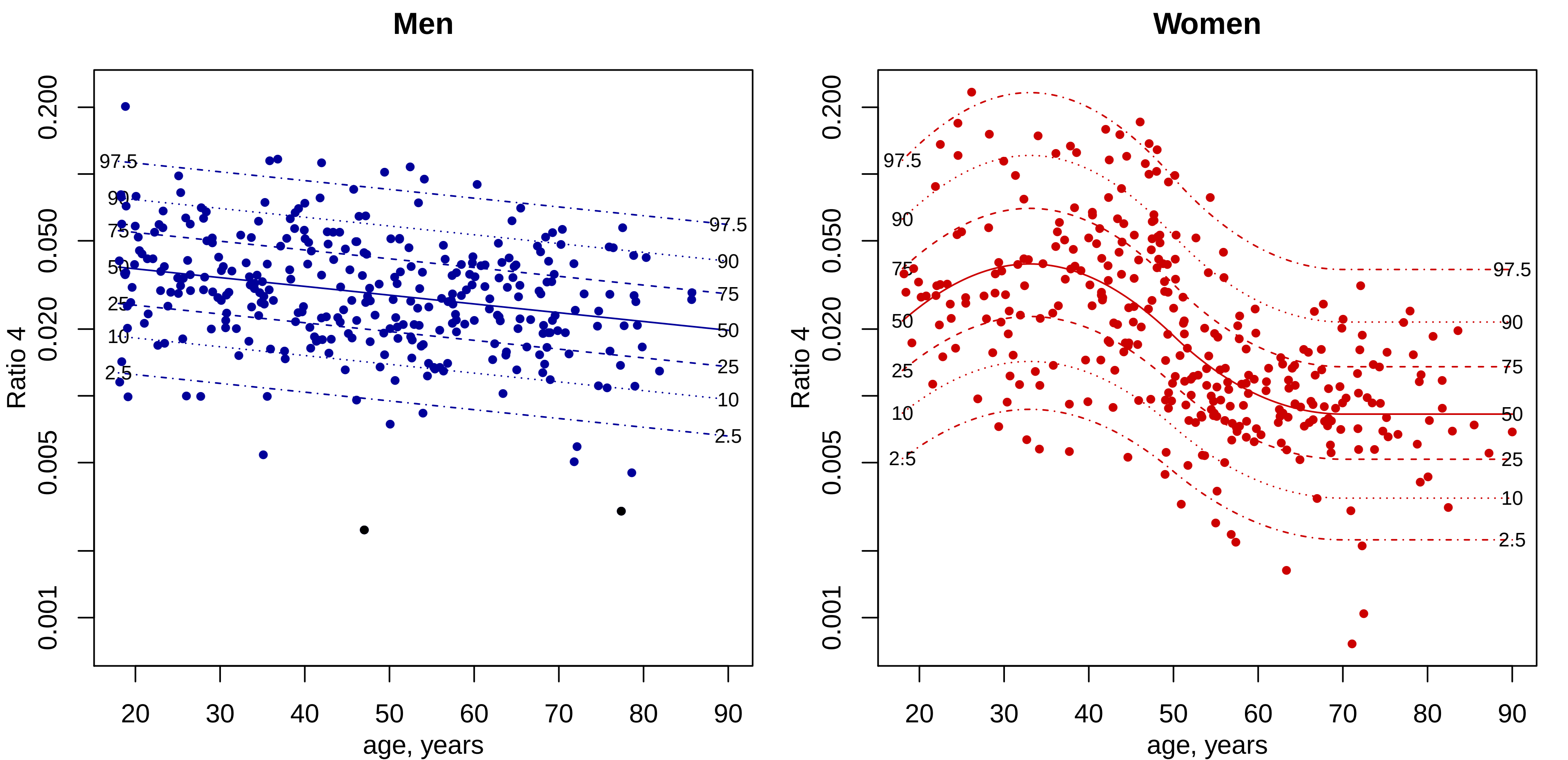

Men
Women
Ratio 4
0.200
0.050
0.020
0.005
0.001
age, years
20
30
40
50
60
70
80
90
97.5
90
75
50
25
10
2.5

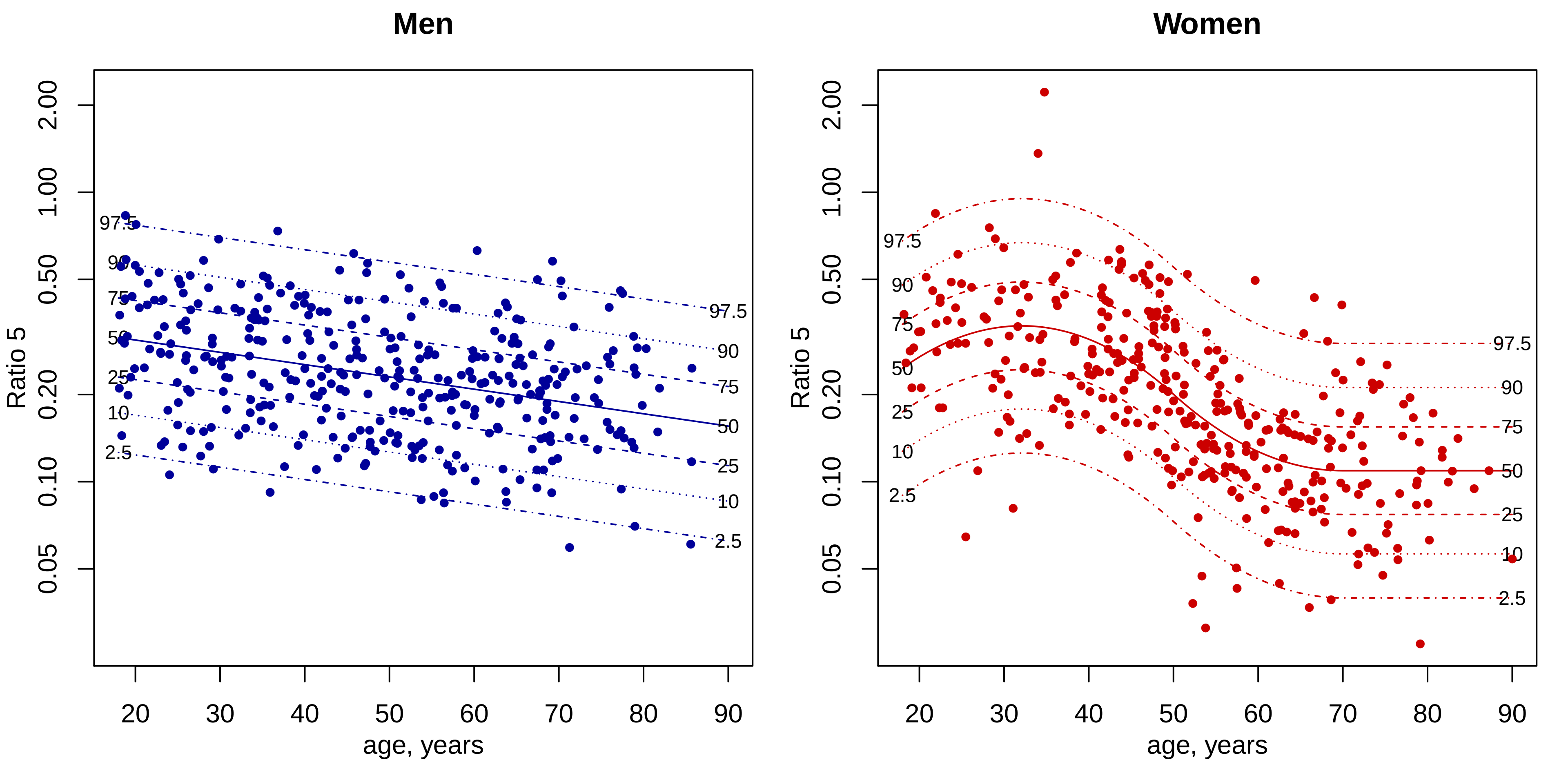

Men
Women
Ratio 5
2.00
1.00
0.50
0.20
0.10
0.05
age, years
20
30
40
50
60
70
80
90
97.5
90
75
50
25
10
2.5

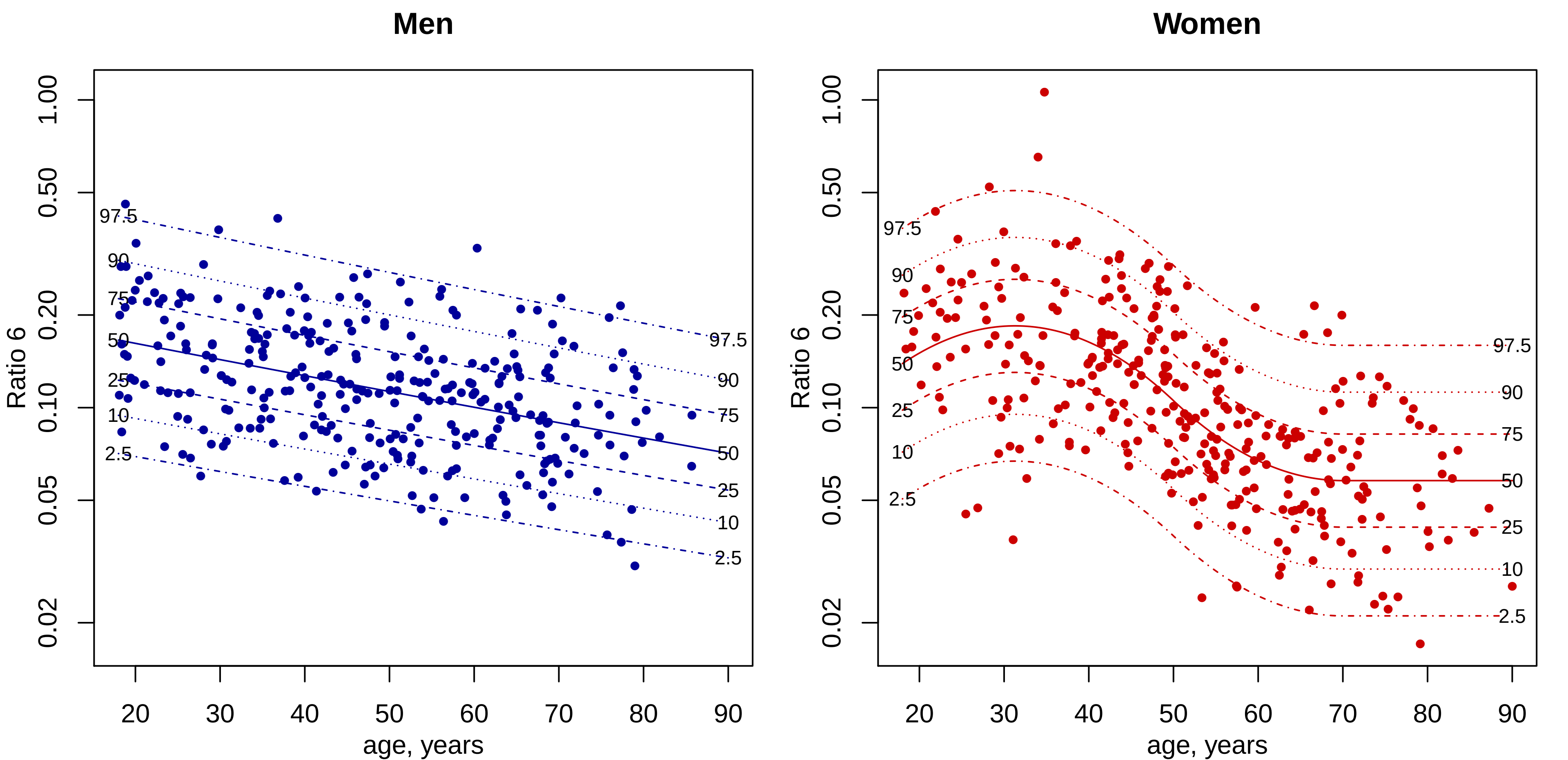

Men
Women
Ratio 6
1.00
0.50
0.20
0.10
0.05
0.02
20
30
40
50
60
70
80
90
age, years
97.5
90
75
50
25
10
2.5

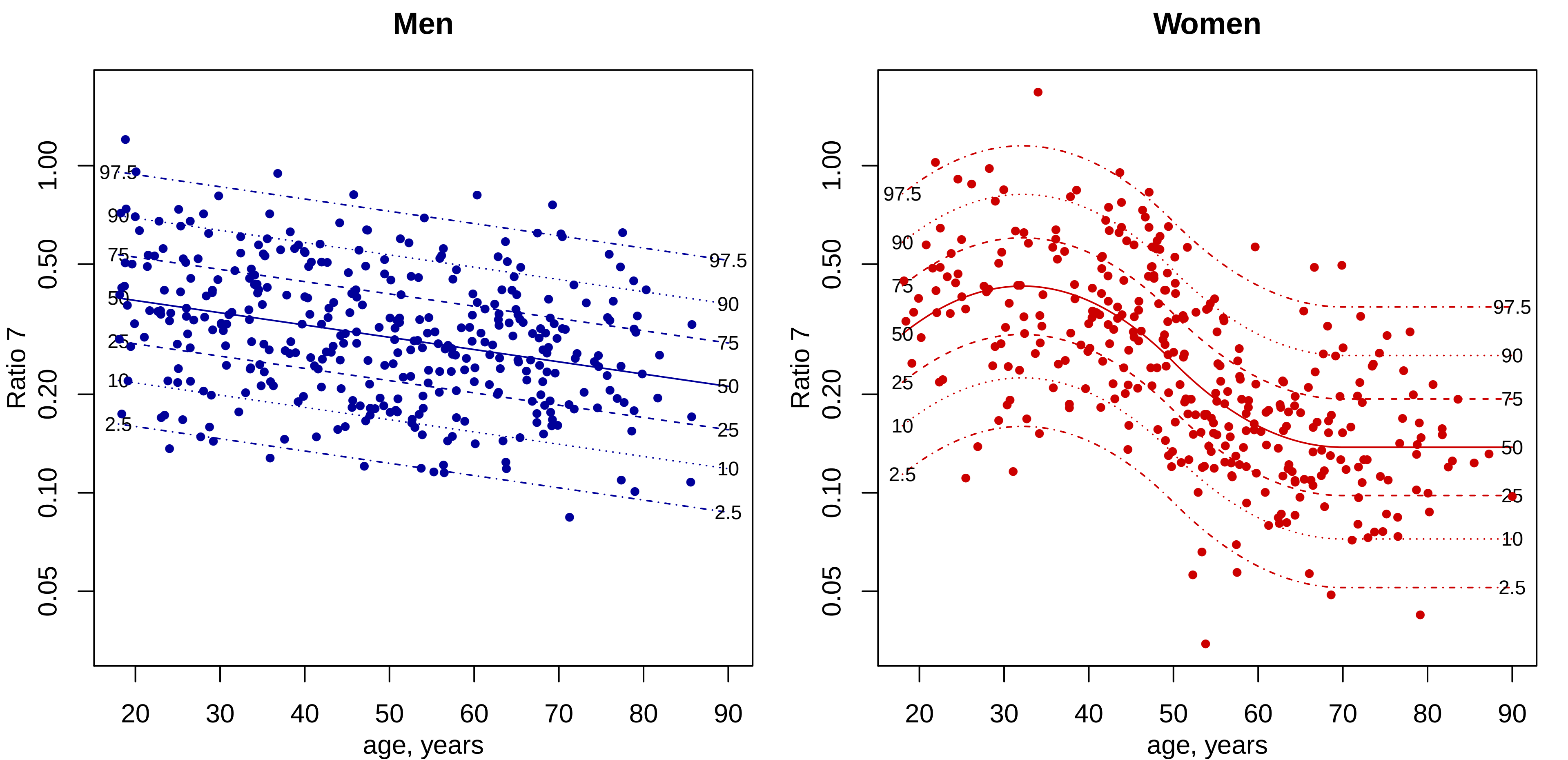

Men
Women
Ratio 7
age, years
1.00
0.50
0.20
0.10
0.05
20
30
40
50
60
70
80
90
97.5
90
75
50
25
10
2.5

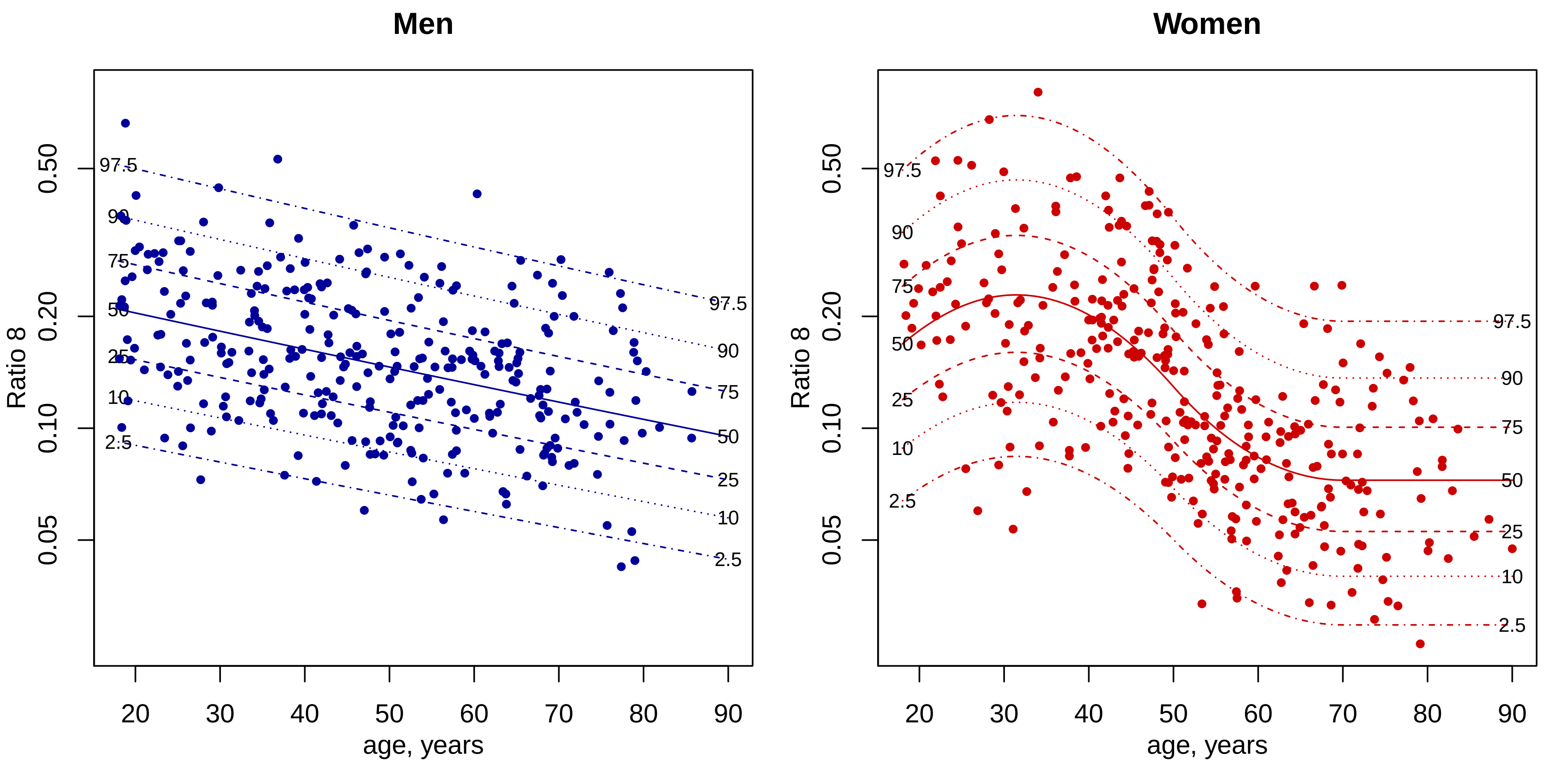
Men
Women
Ratio 8
0.05
0.10
0.20
0.50
age, years
20
30
40
50
60
70
80
90
97.5
90
75
50
25
10
2.5

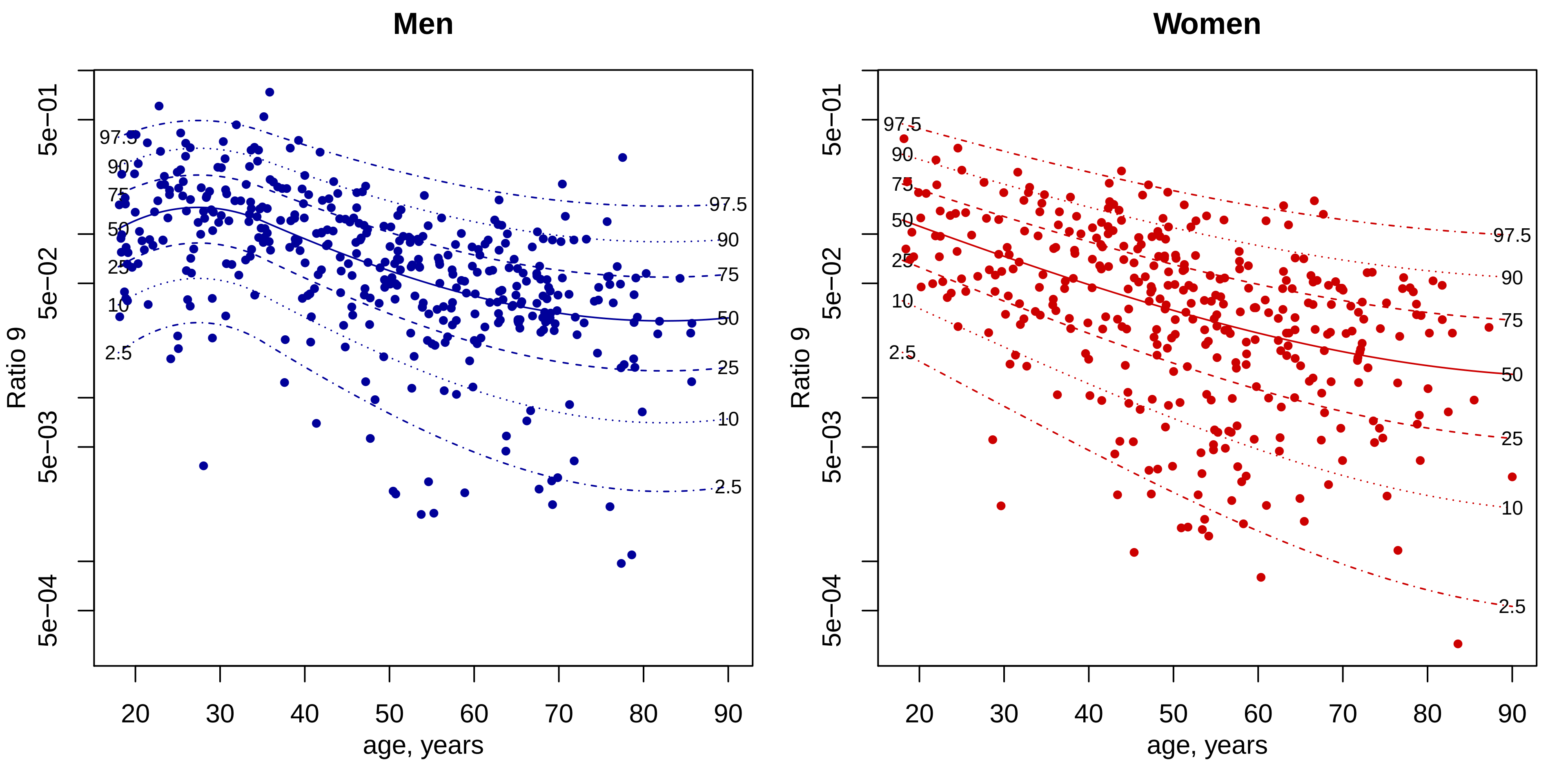

Men
Women
Ratio 9
5e−01
5e−02
5e−03
5e−04
age, years
20
30
40
50
60
70
80
90
97.5
90
75
50
25
10
2.5

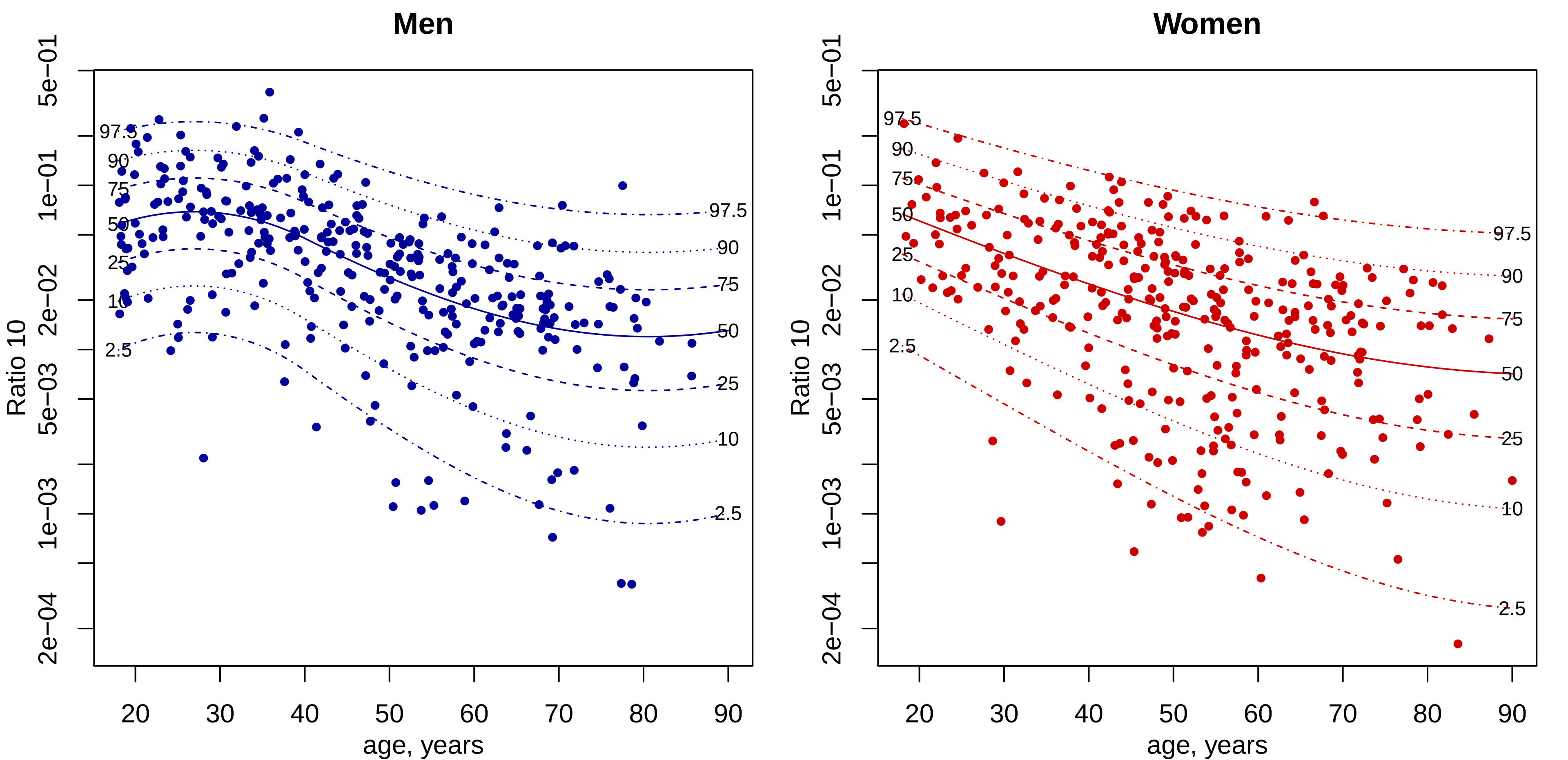

Men
Women
Ratio 10
age, years
5e−01
1e−01
2e−02
5e−03
1e−03
2e−04
20
30
40
50
60
70
80
90
97.5
90
75
50
25
10
2.5

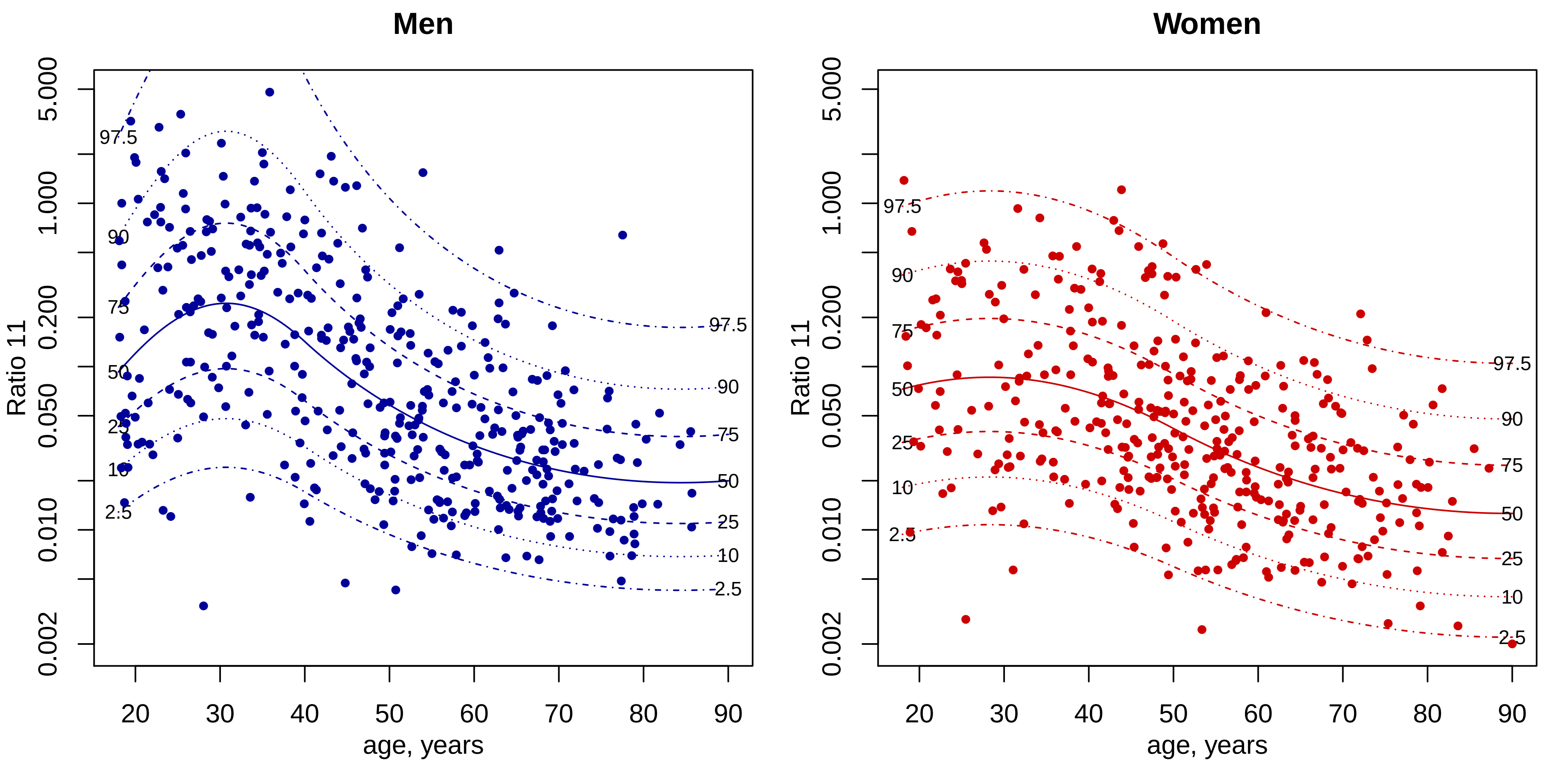

Men
Women
Ratio 11
5.000
1.000
0.200
0.050
0.010
0.002
age, years
20
30
40
50
60
70
80
90
97.5
90
75
50
25
10
2.5

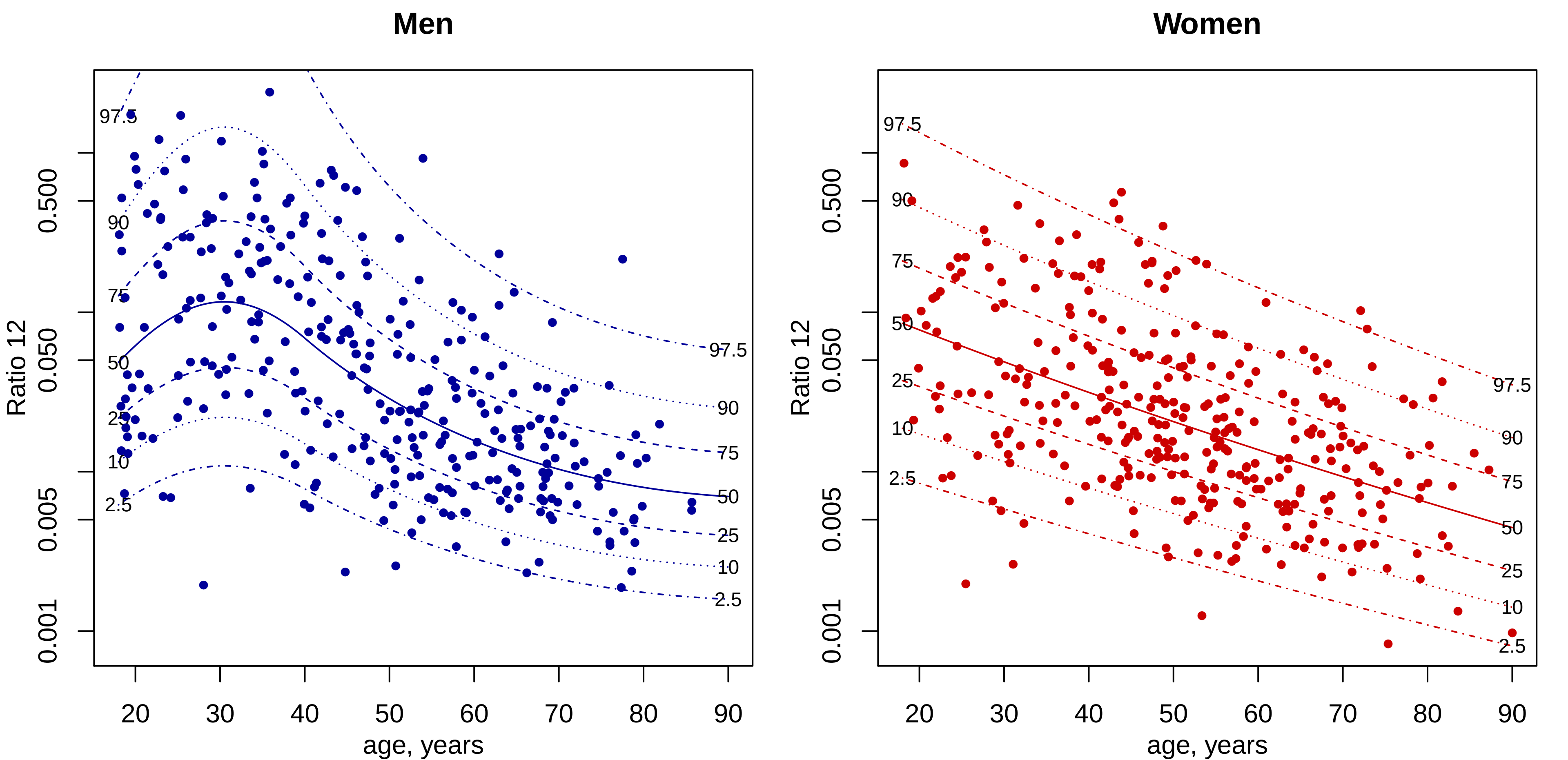

Men
Women
Ratio 12
0.500
0.050
0.005
0.001
97.5
90
75
50
25
10
2.5
20
30
40
50
60
70
80
90
age, years

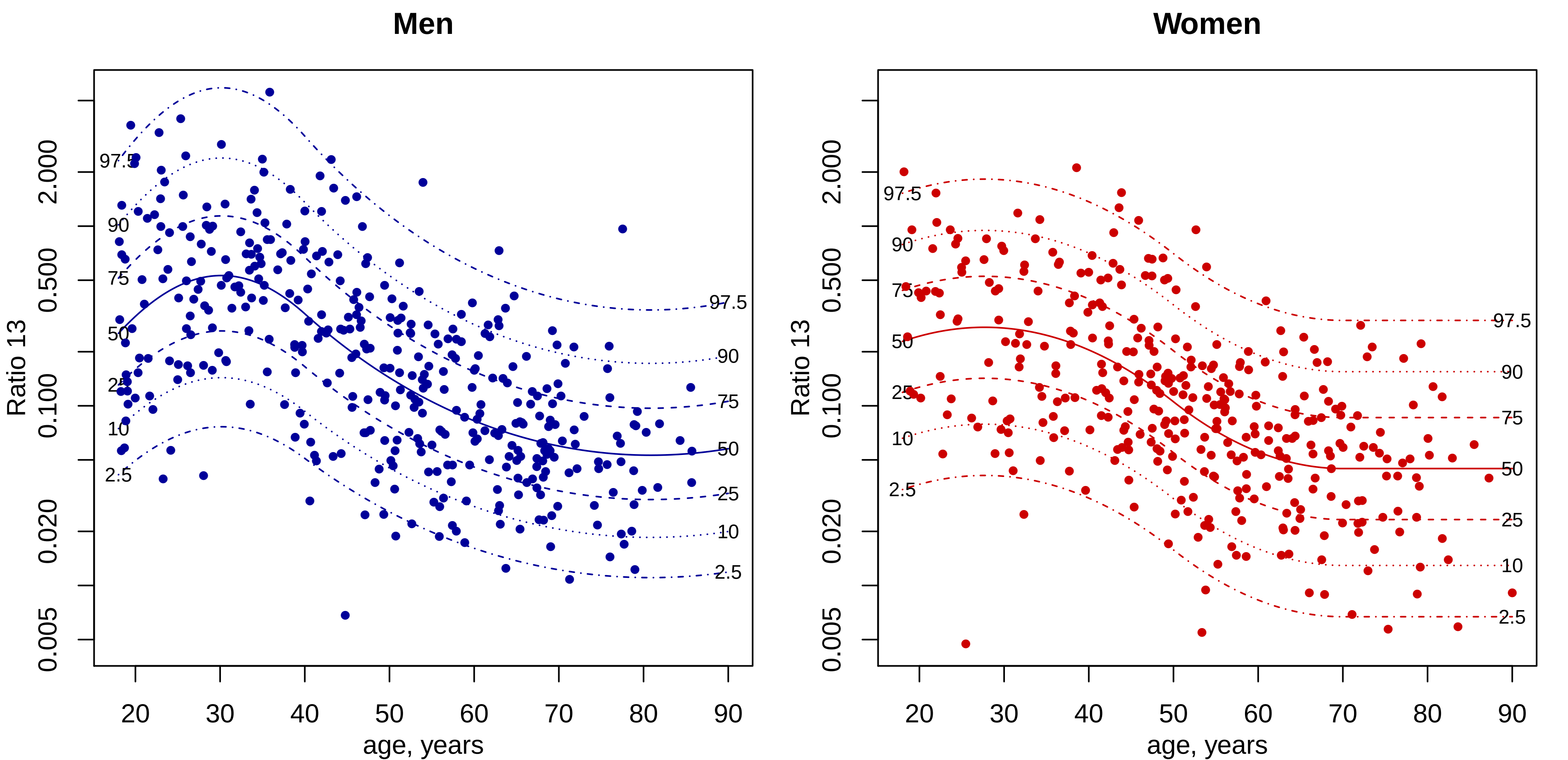

Men
Women
Ratio 13
0.005
0.020
0.100
0.500
2.000
age, years
20
30
40
50
60
70
80
90
97.5
90
75
50
25
10
2.5

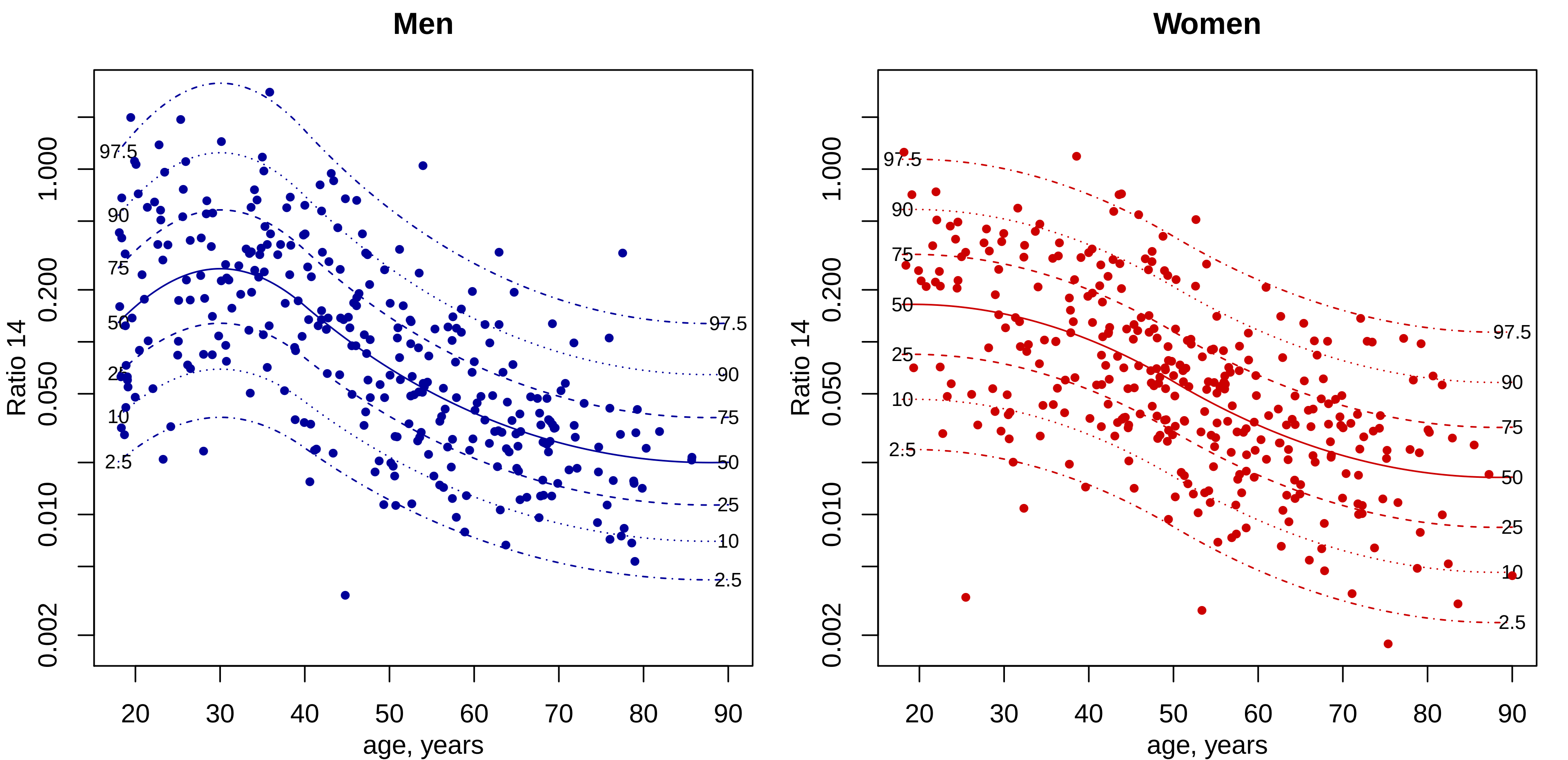

Men
Women
Ratio 14
age, years
1.000
0.200
0.050
0.010
0.002
20
30
40
50
60
70
80
90
97.5
90
75
50
25
10
2.5

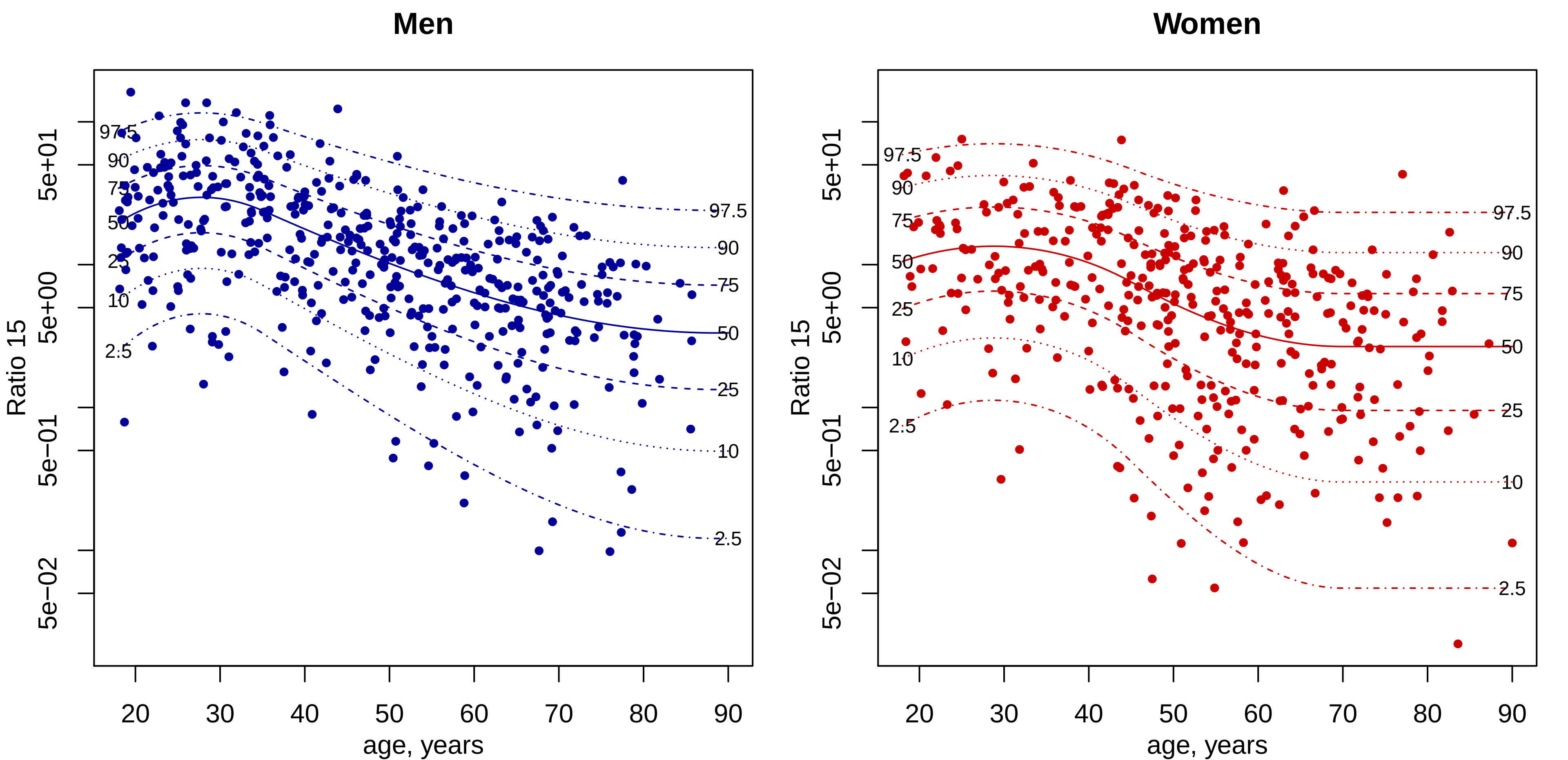

Men
Women
Ratio 15
5e+01
5e+00
5e−01
5e−02
97.5
90
75
50
25
10
2.5
20
30
40
50
60
70
80
90
age, years

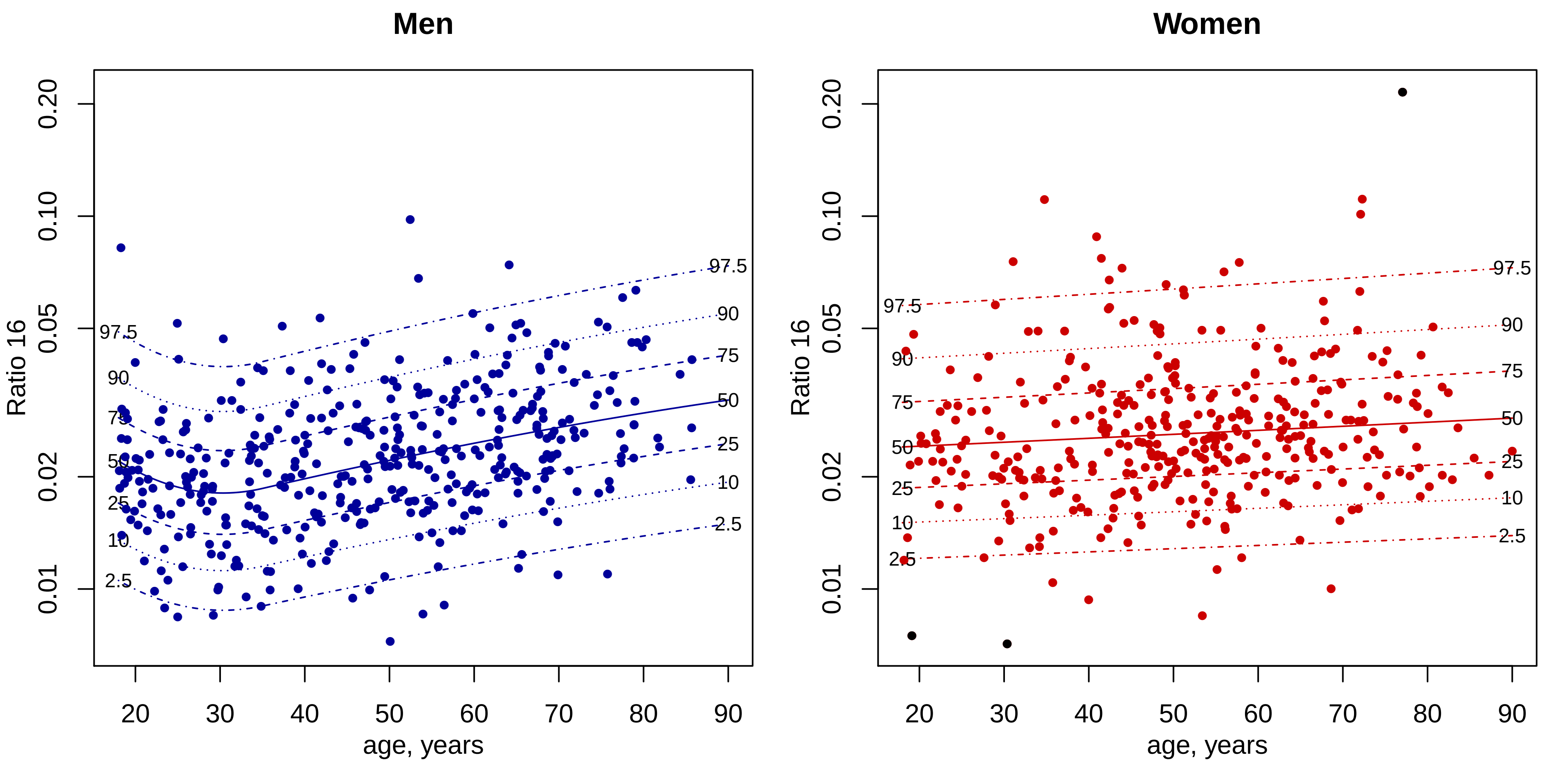

Men
Women
Ratio 16
0.20
0.10
0.05
0.02
0.01
97.5
90
75
50
25
10
2.5
20
30
40
50
60
70
80
90
age, years

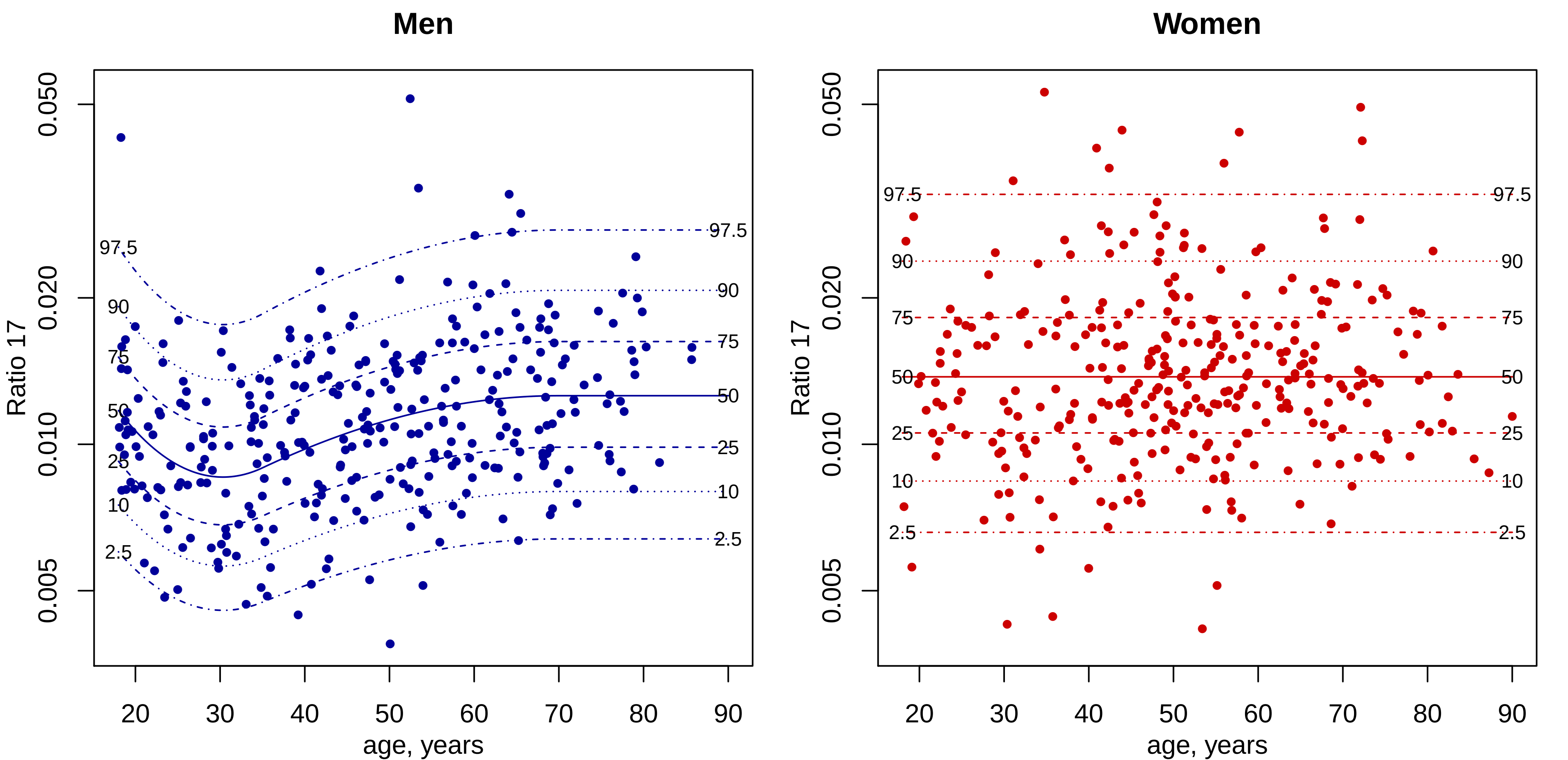

Men
Women
Ratio 17
0.050
0.020
0.010
0.005
97.5
90
75
50
25
10
2.5
20
30
40
50
60
70
80
90
age, years

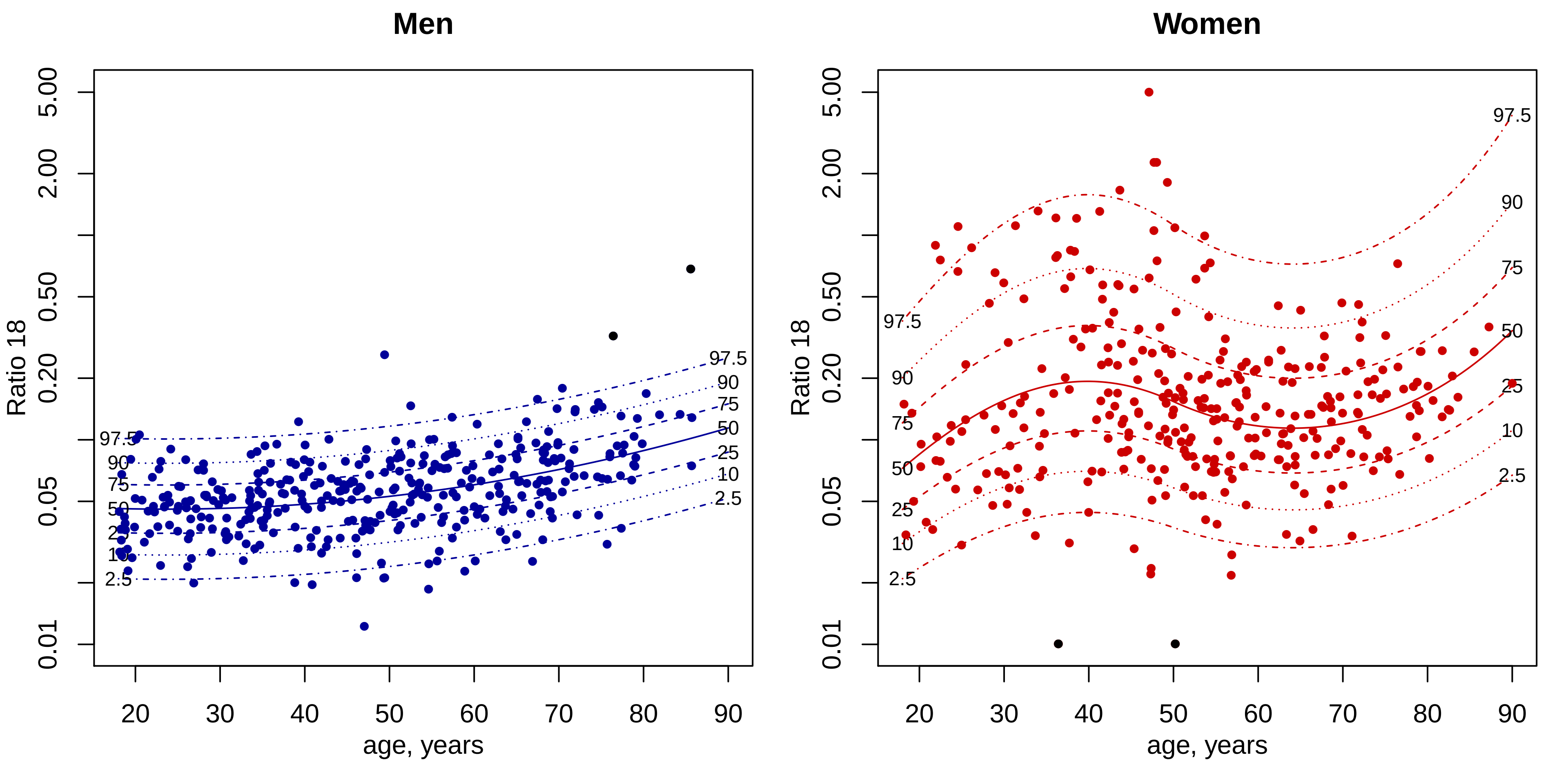
Men
Women
Ratio 18
5.00
2.00
0.50
0.20
0.05
0.01
age, years
20
30
40
50
60
70
80
90
97.5
90
75
50
25
10
2.5

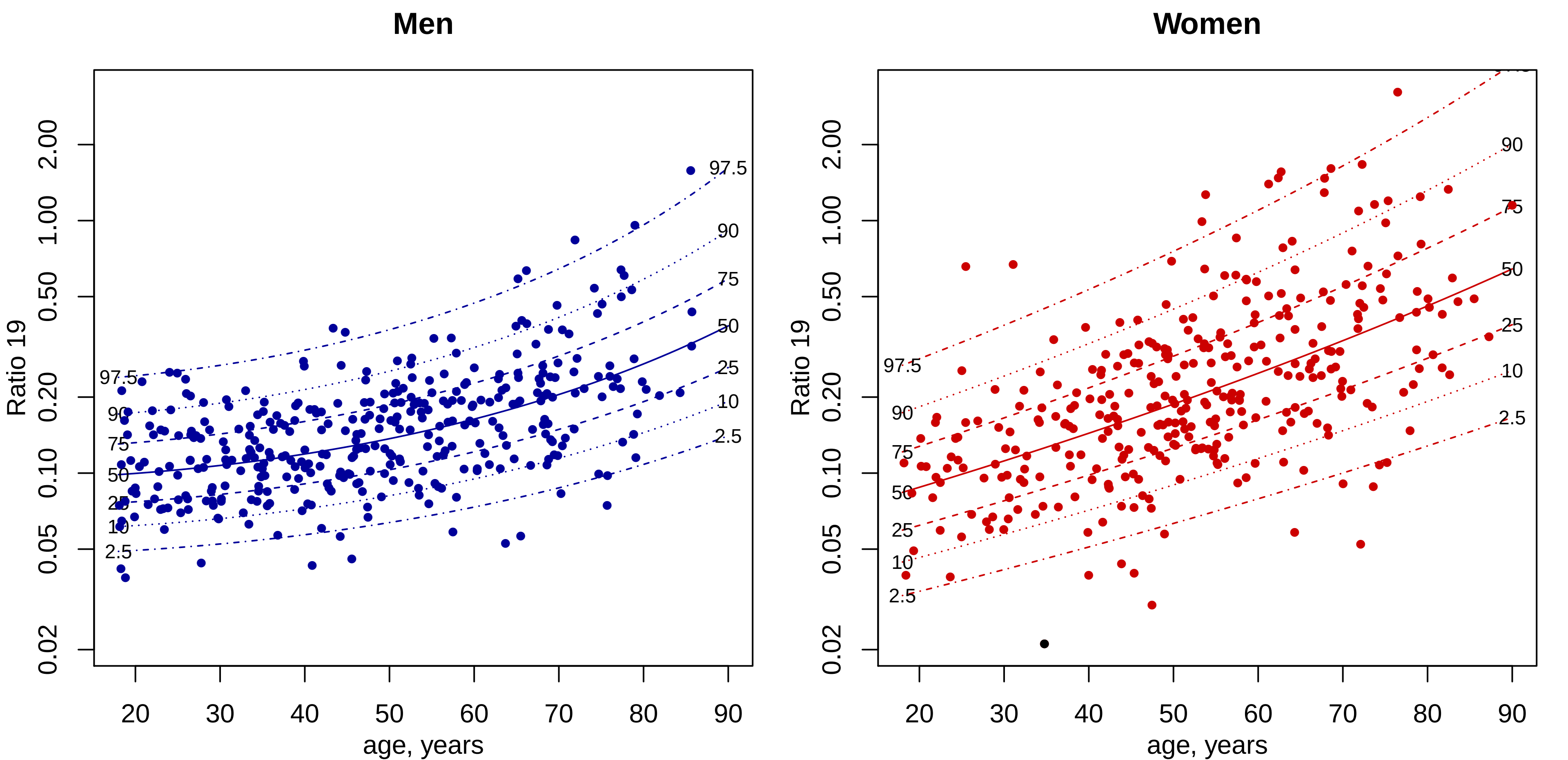
Men
Women
Ratio 19
2.00
1.00
0.50
0.20
0.10
0.05
0.02
age, years
20
30
40
50
60
70
80
90
97.5
90
75
50
25
10
2.5

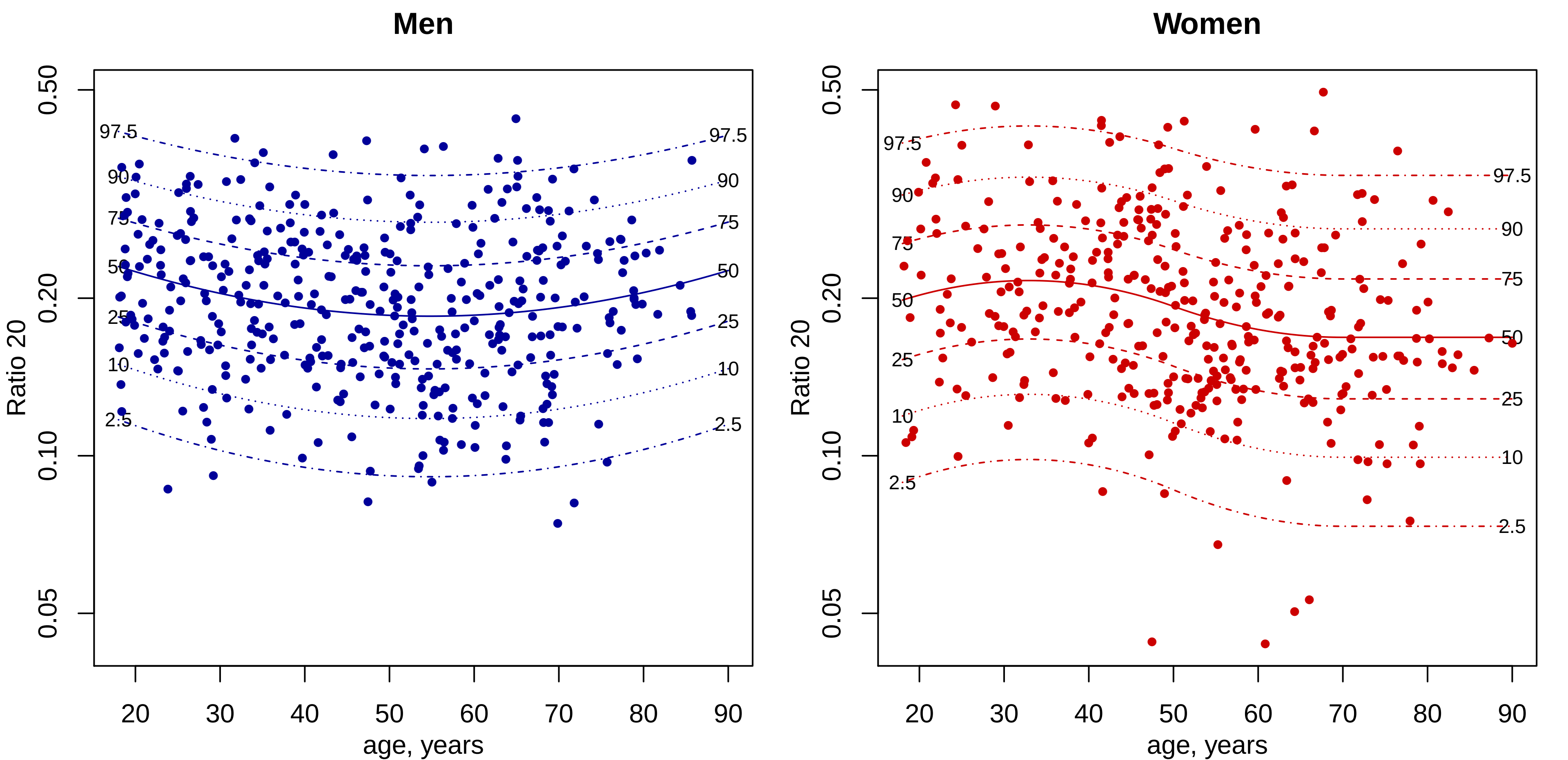

Men
Women
Ratio 20
0.50
0.20
0.10
0.05
age, years
20
30
40
50
60
70
80
90
97.5
90
75
50
25
10
2.5

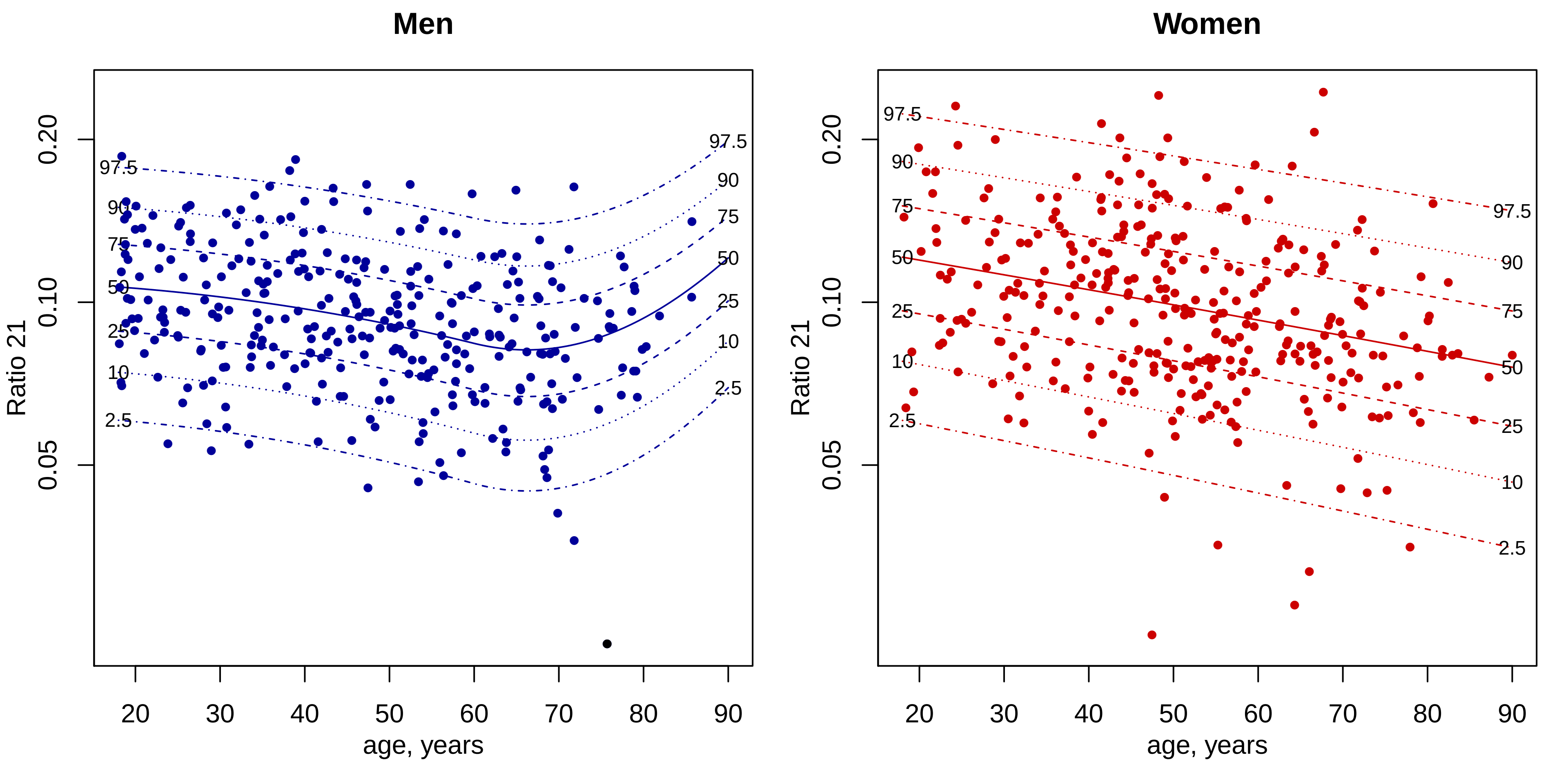
Men
Women
Ratio 21
0.20
0.10
0.05
age, years
20
30
40
50
60
70
80
90
97.5
90
75
50
25
10
2.5

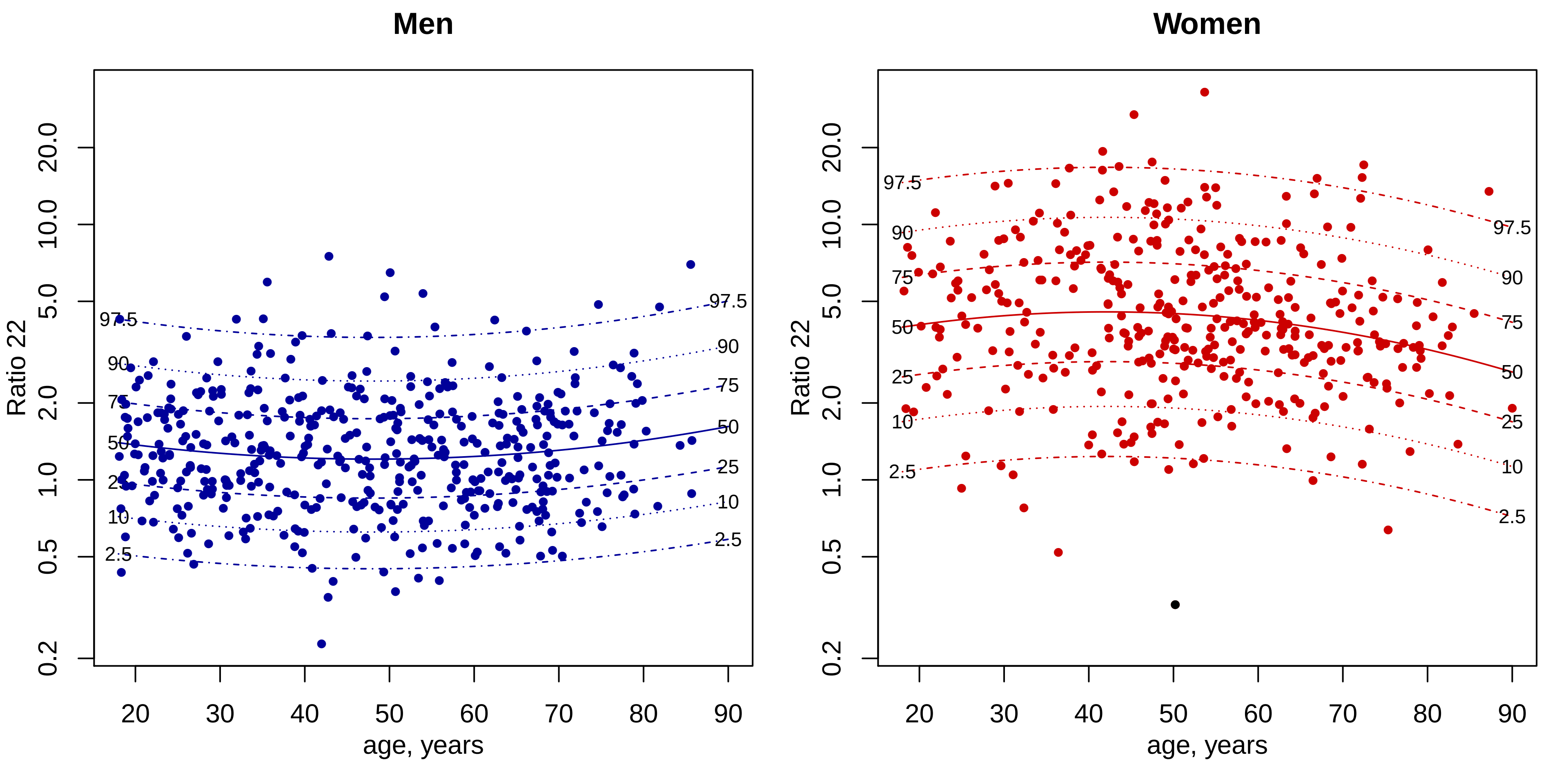

Men
Women
Ratio 22
age, years
0.2
0.5
1.0
2.0
5.0
10.0
20.0
20
30
40
50
60
70
80
90
97.5
90
75
50
25
10
2.5

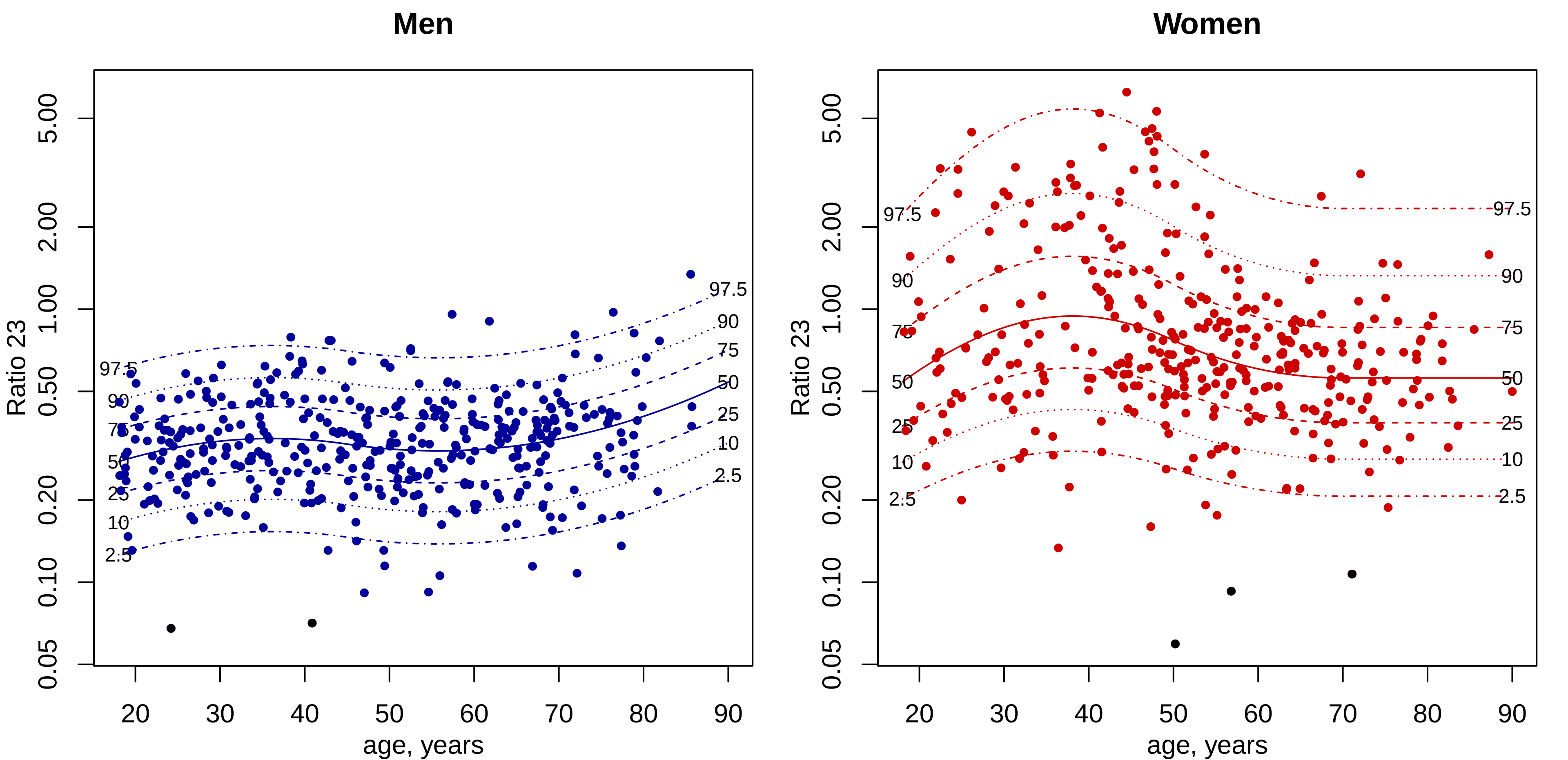
Men
Women
Ratio 23
5.00
2.00
1.00
0.50
0.20
0.10
0.05
20
30
40
50
60
70
80
90
age, years
97.5
90
75
50
25
10
2.5

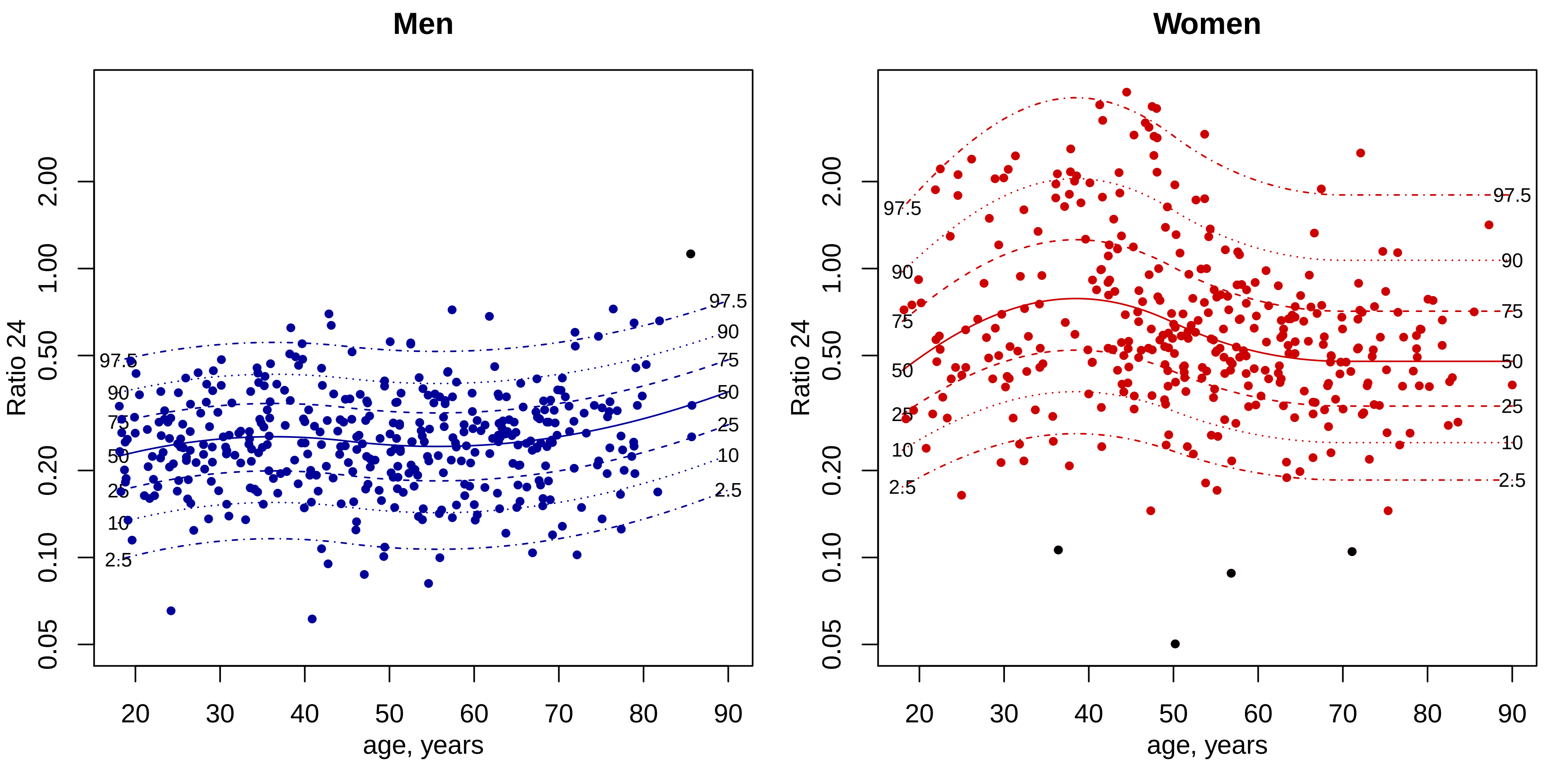

Men
Women
Ratio 24
0.05
0.10
0.20
0.50
1.00
2.00
20
30
40
50
60
70
80
90
age, years
97.5
90
75
50
25
10
2.5

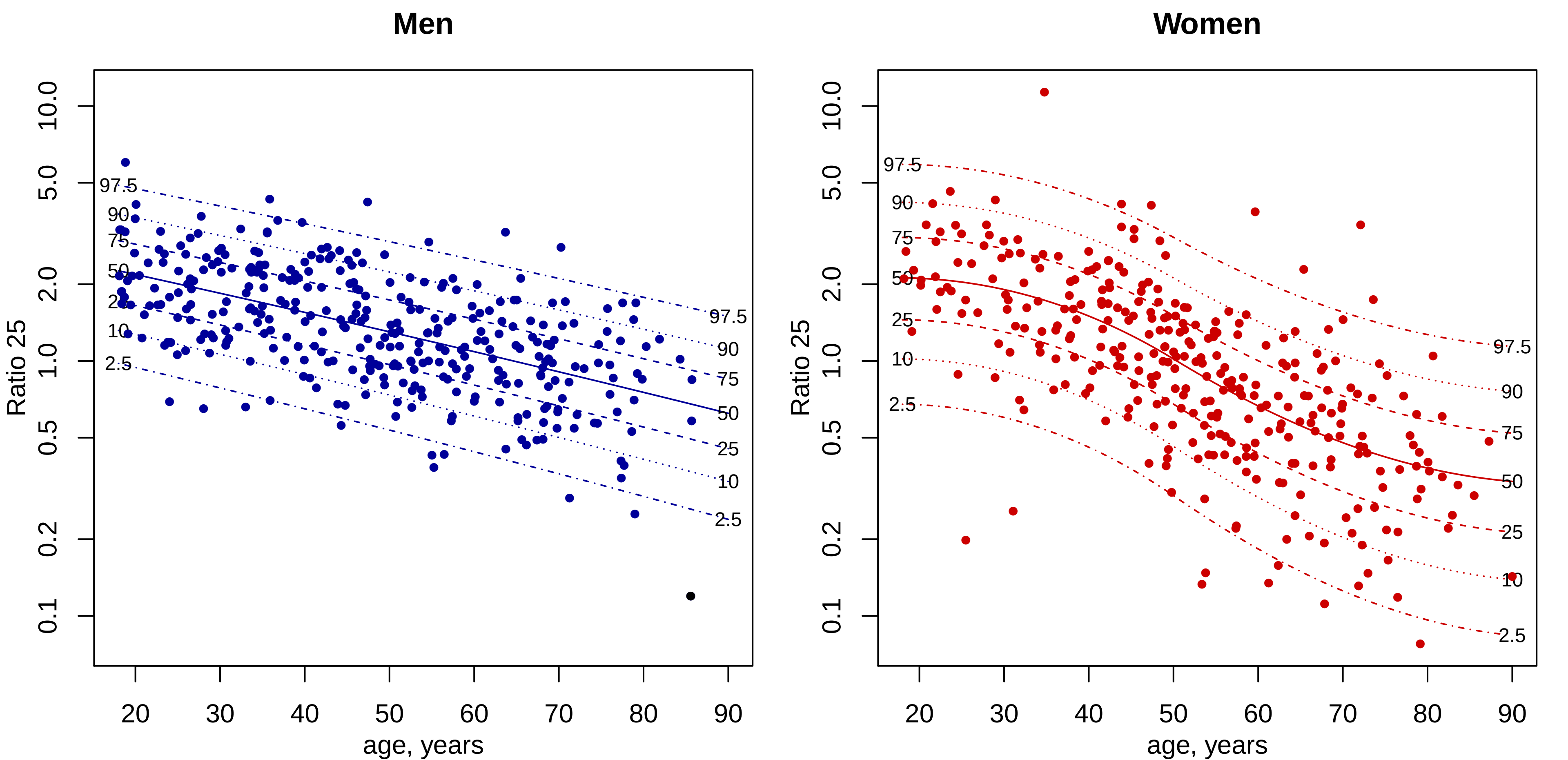
Men
Women
Ratio 25
age, years
10.0
5.0
2.0
1.0
0.5
0.2
0.1
20
30
40
50
60
70
80
90
97.5
90
75
50
25
10
2.5

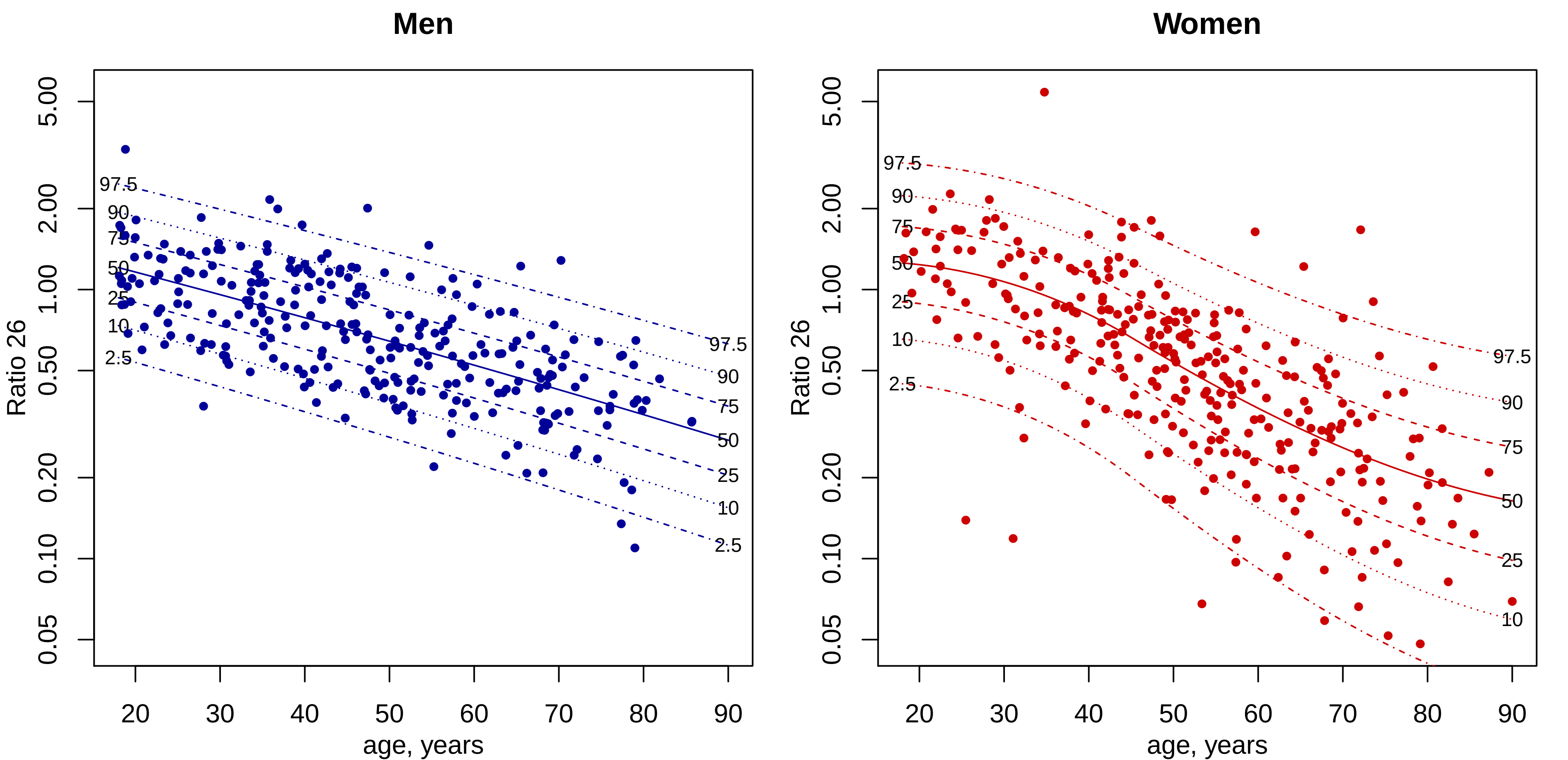

Men
Women
Ratio 26
age, years
5.00
2.00
1.00
0.50
0.20
0.10
0.05
20
30
40
50
60
70
80
90
97.5
90
75
50
25
10
2.5

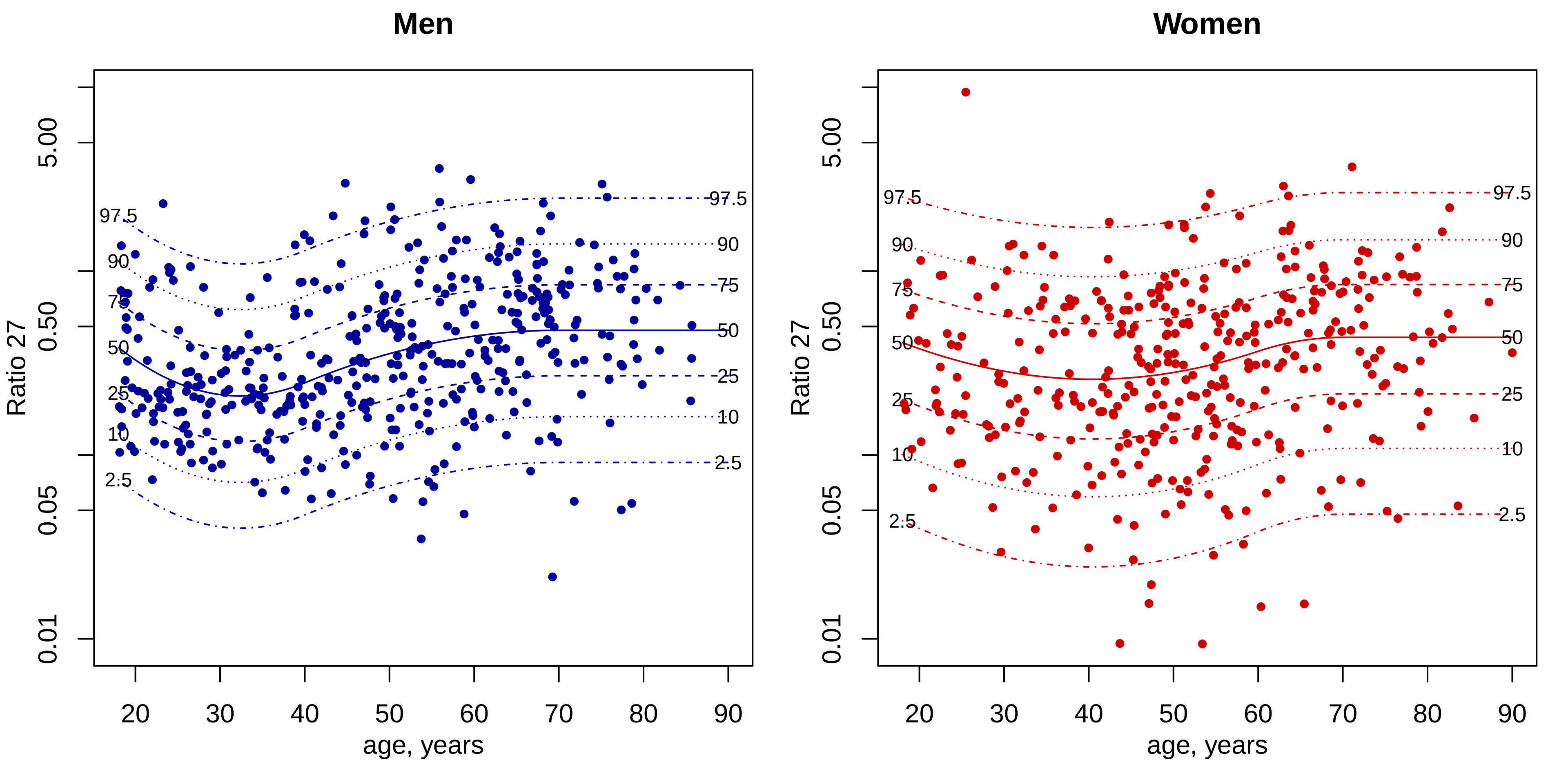
Men
Women
Ratio 27
age, years
5.00
0.50
0.05
0.01
20
30
40
50
60
70
80
90
97.5
90
75
50
25
10
2.5

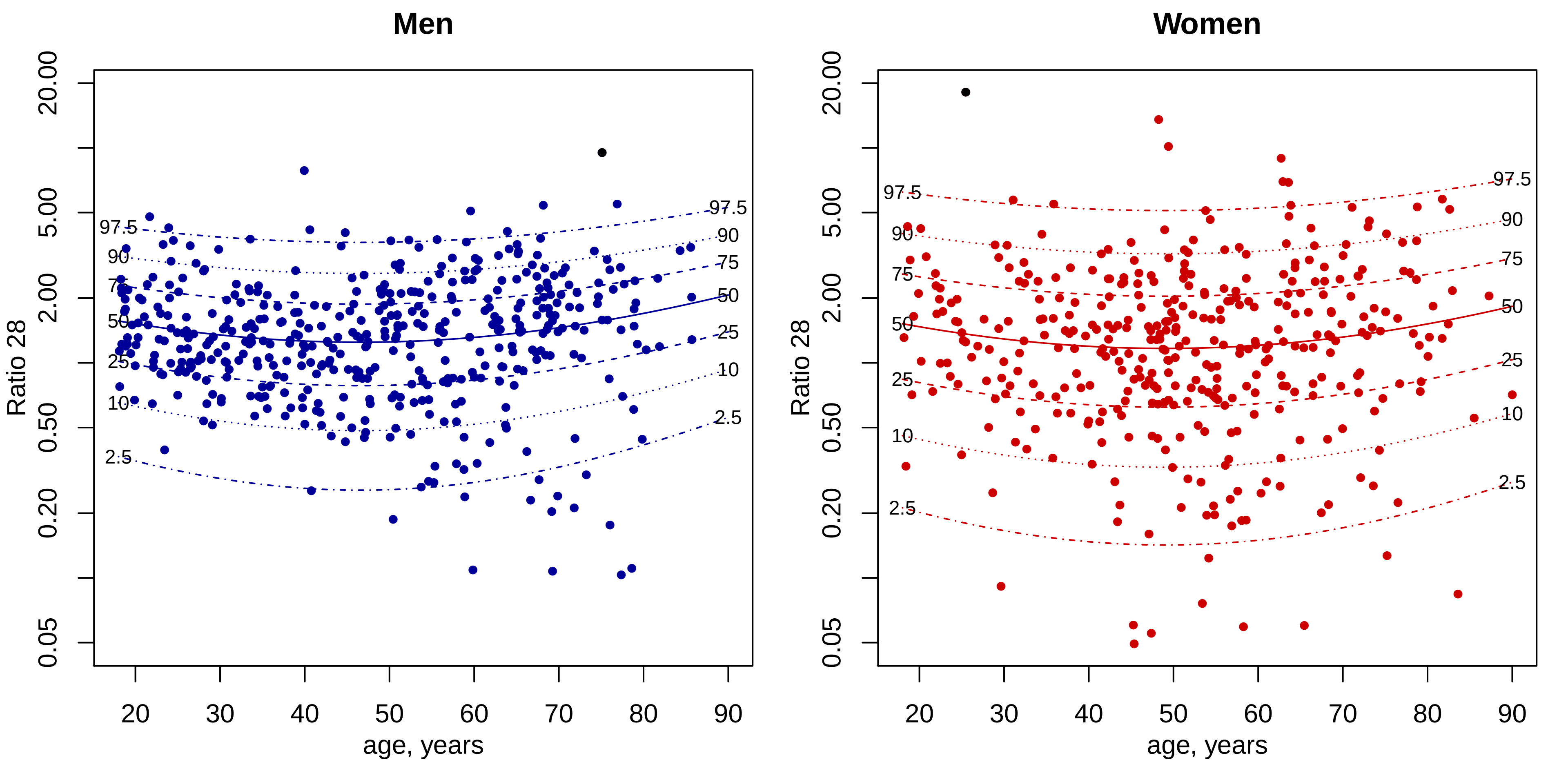

Men
Women
Ratio 28
20.00
5.00
2.00
0.50
0.20
0.05
age, years
20
30
40
50
60
70
80
90
97.5
90
75
50
25
10
2.5

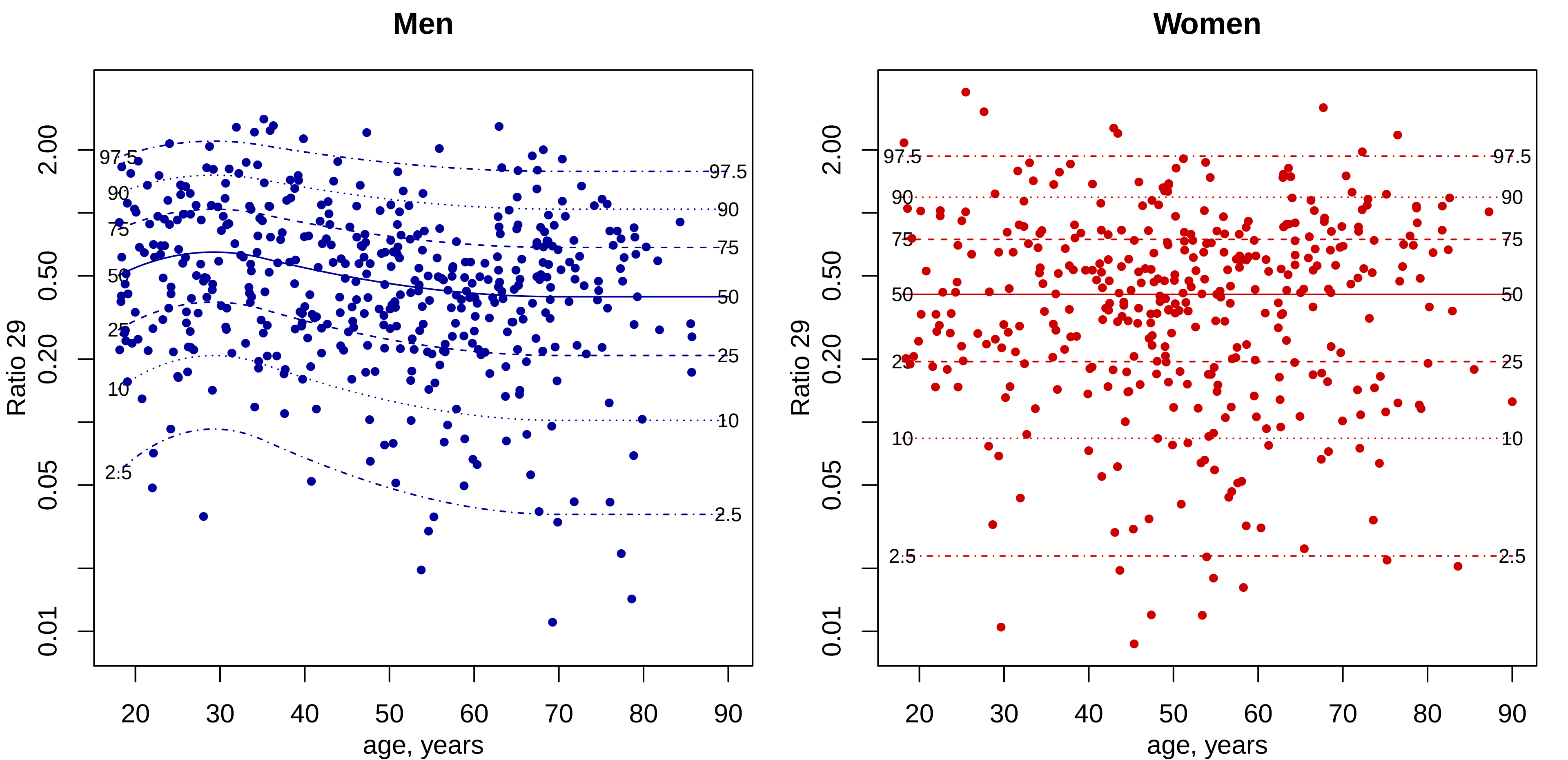

Men
Women
Ratio 29
age, years
2.00
0.50
0.20
0.05
0.01
20
30
40
50
60
70
80
90
97.5
90
75
50
25
10
2.5

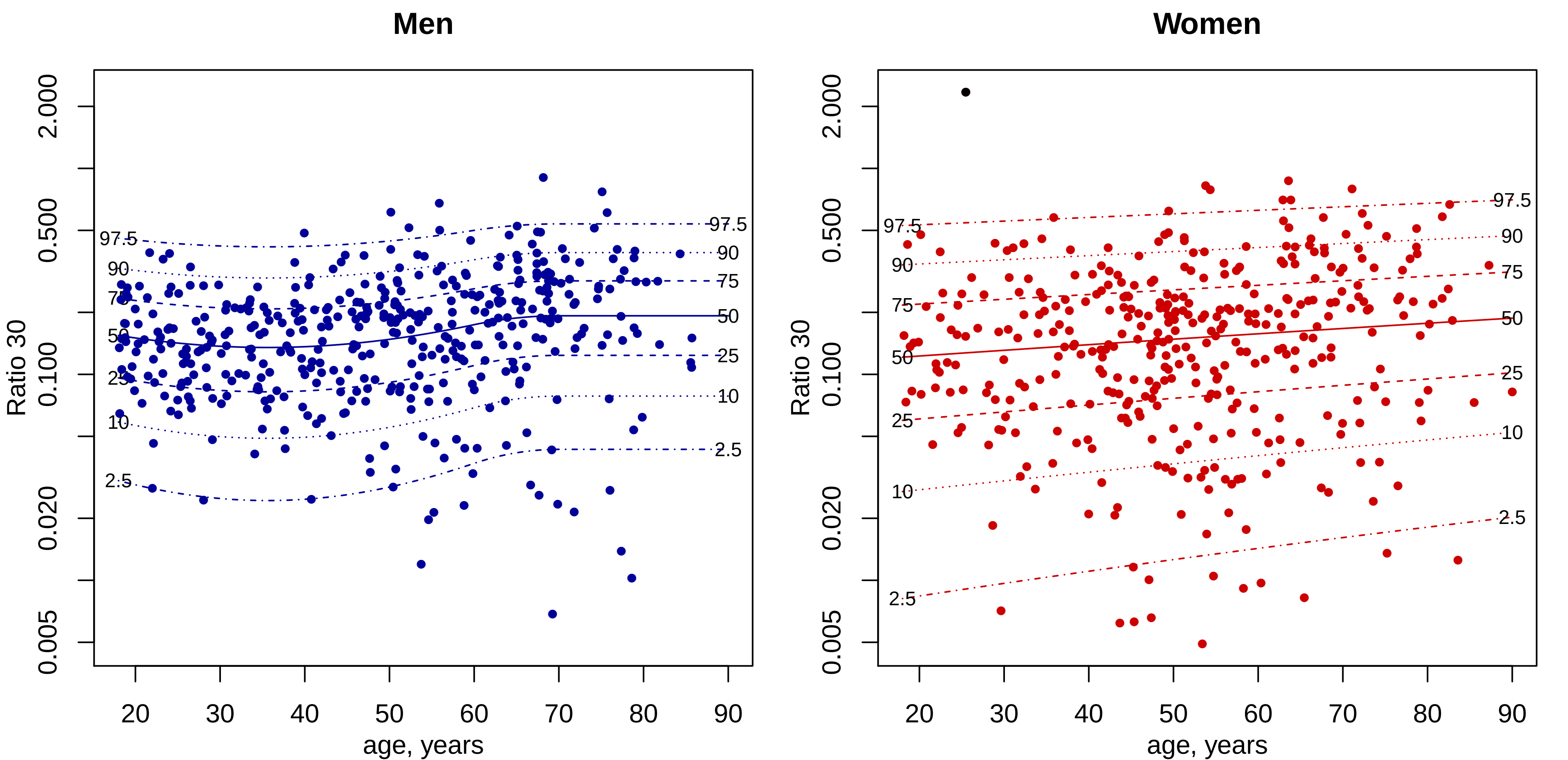

Men
Women
Ratio 30
2.000
0.500
0.100
0.020
0.005
97.5
90
75
50
25
10
2.5
20
30
40
50
60
70
80
90
age, years

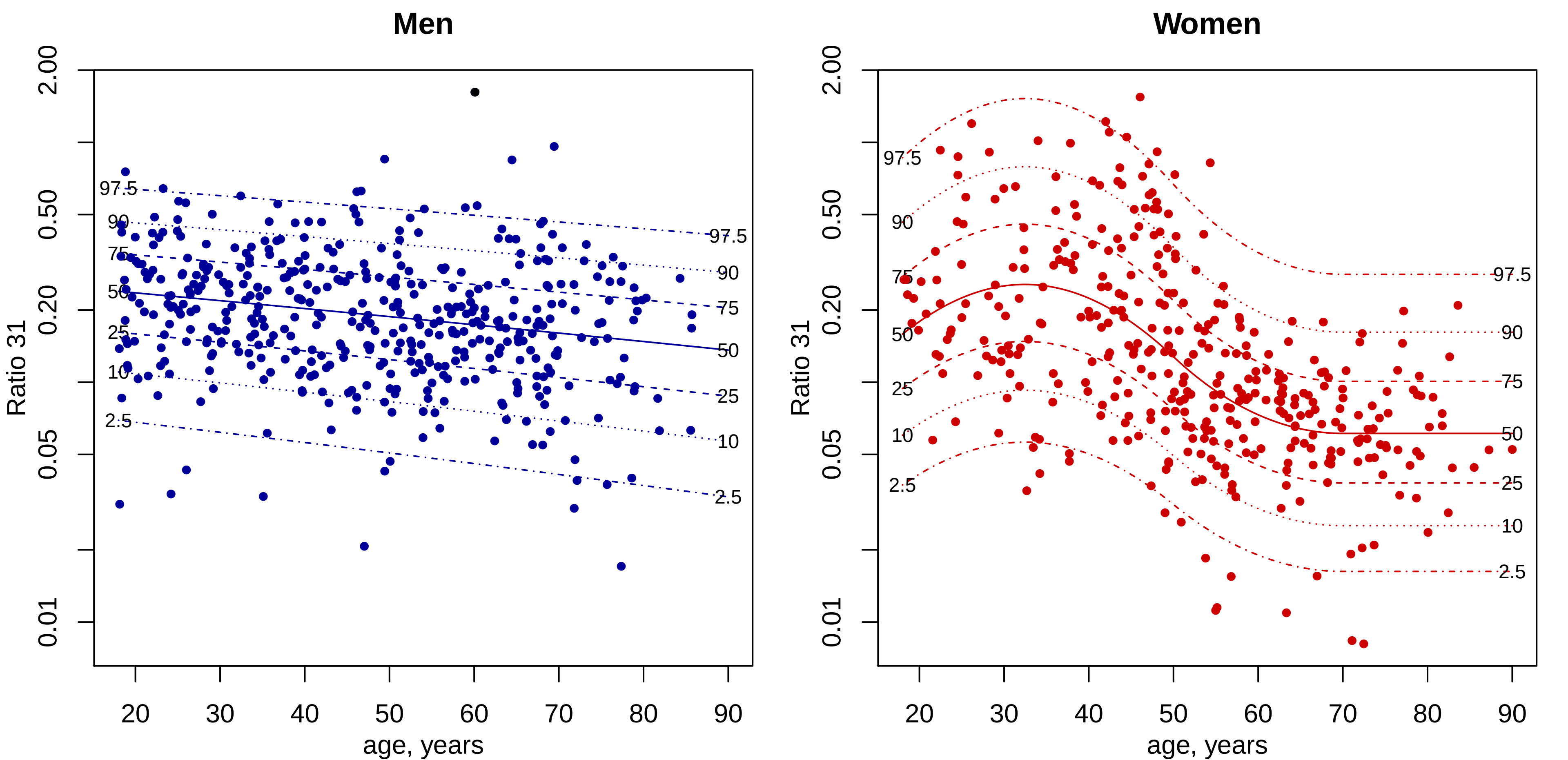

Men
Women
Ratio 31
age, years
2.00
0.50
0.20
0.05
0.01
20
30
40
50
60
70
80
90
97.5
75
50
25
10
2.5

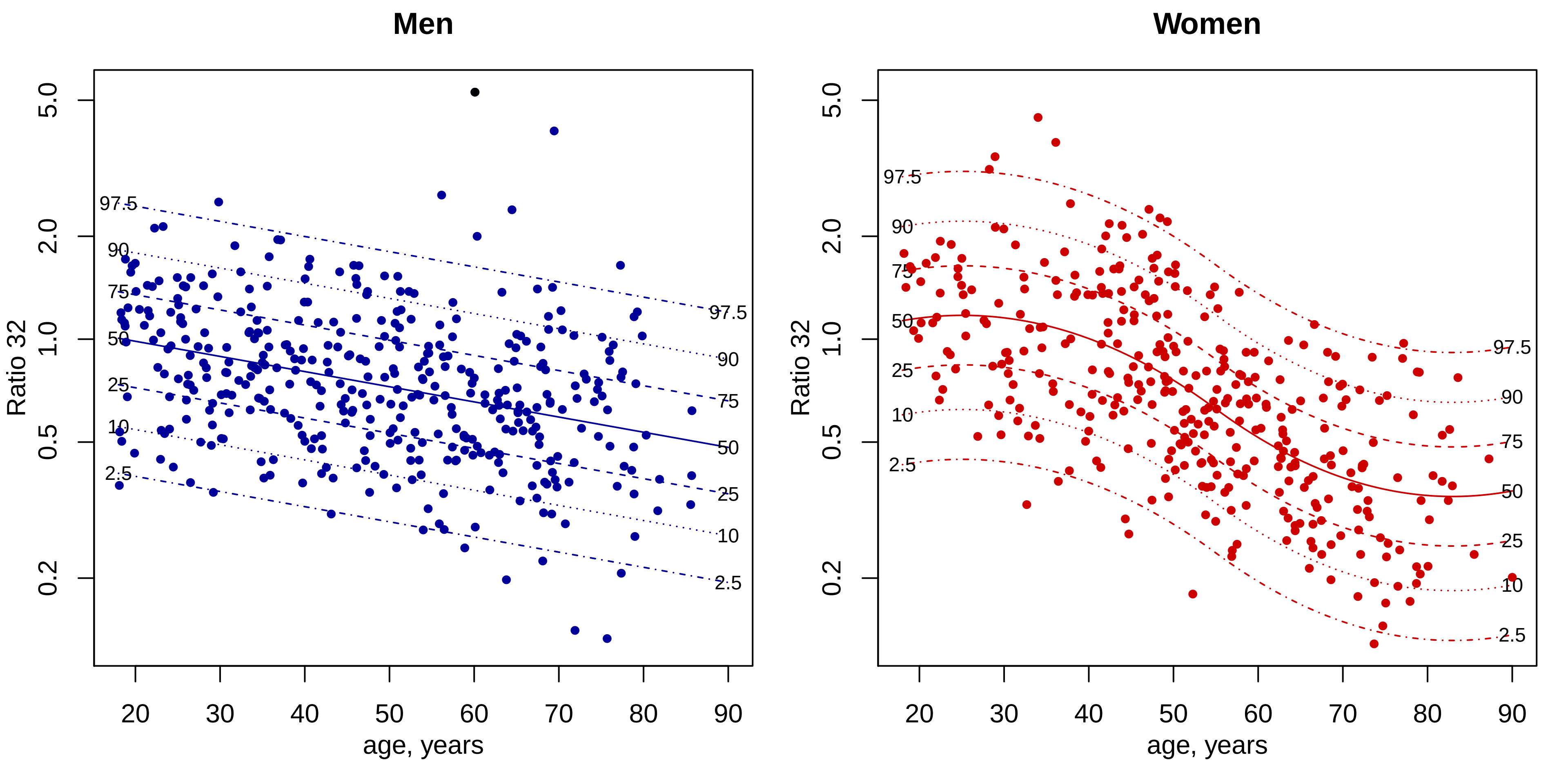

Men
Women
Ratio 32
0.2
0.5
1.0
2.0
5.0
20
30
40
50
60
70
80
90
age, years
97.5
90
75
50
25
10
2.5

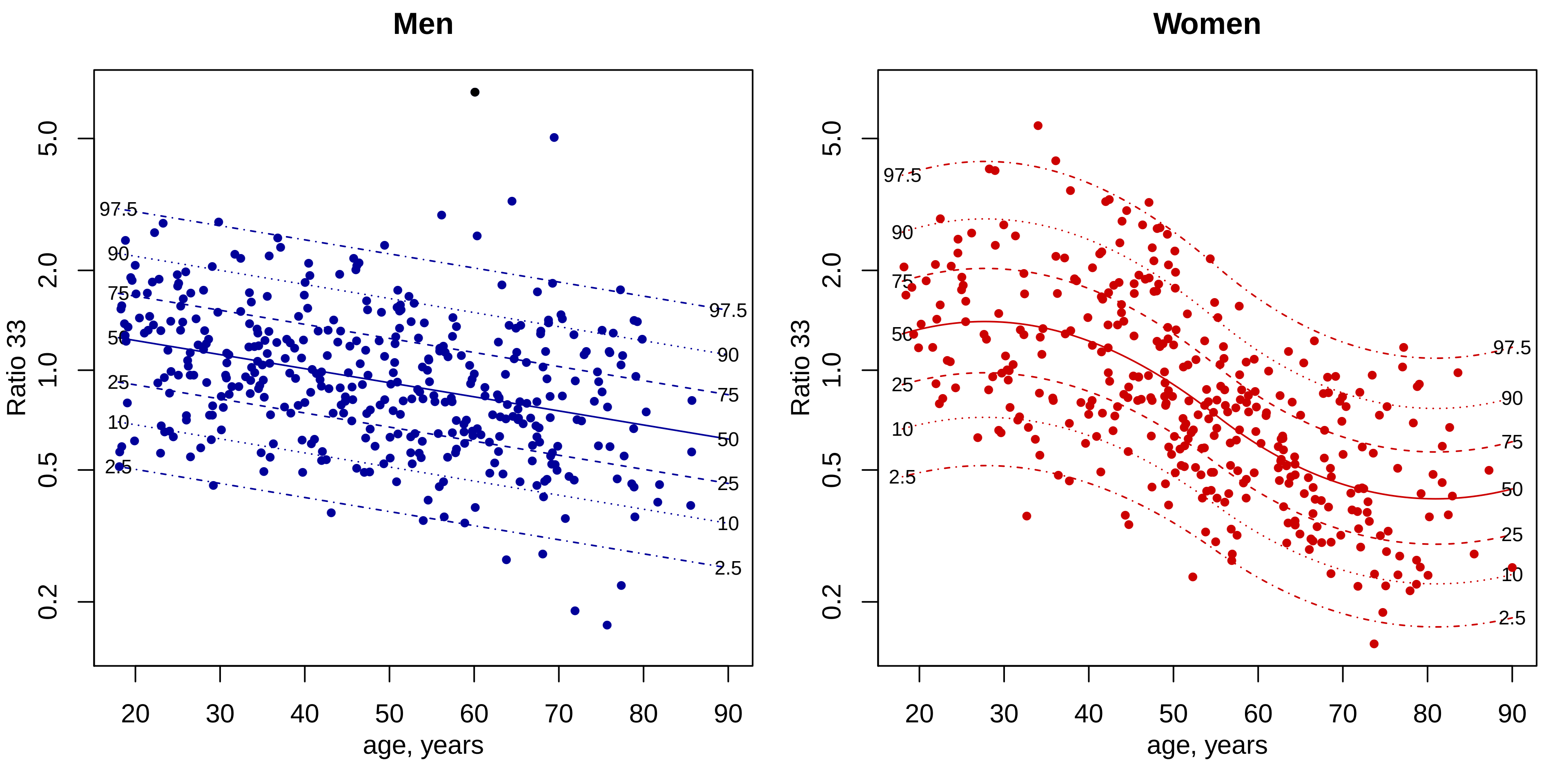

Men
Women
Ratio 33
5.0
2.0
1.0
0.5
0.2
20
30
40
50
60
70
80
90
age, years
97.5
90
75
50
25
10
2.5

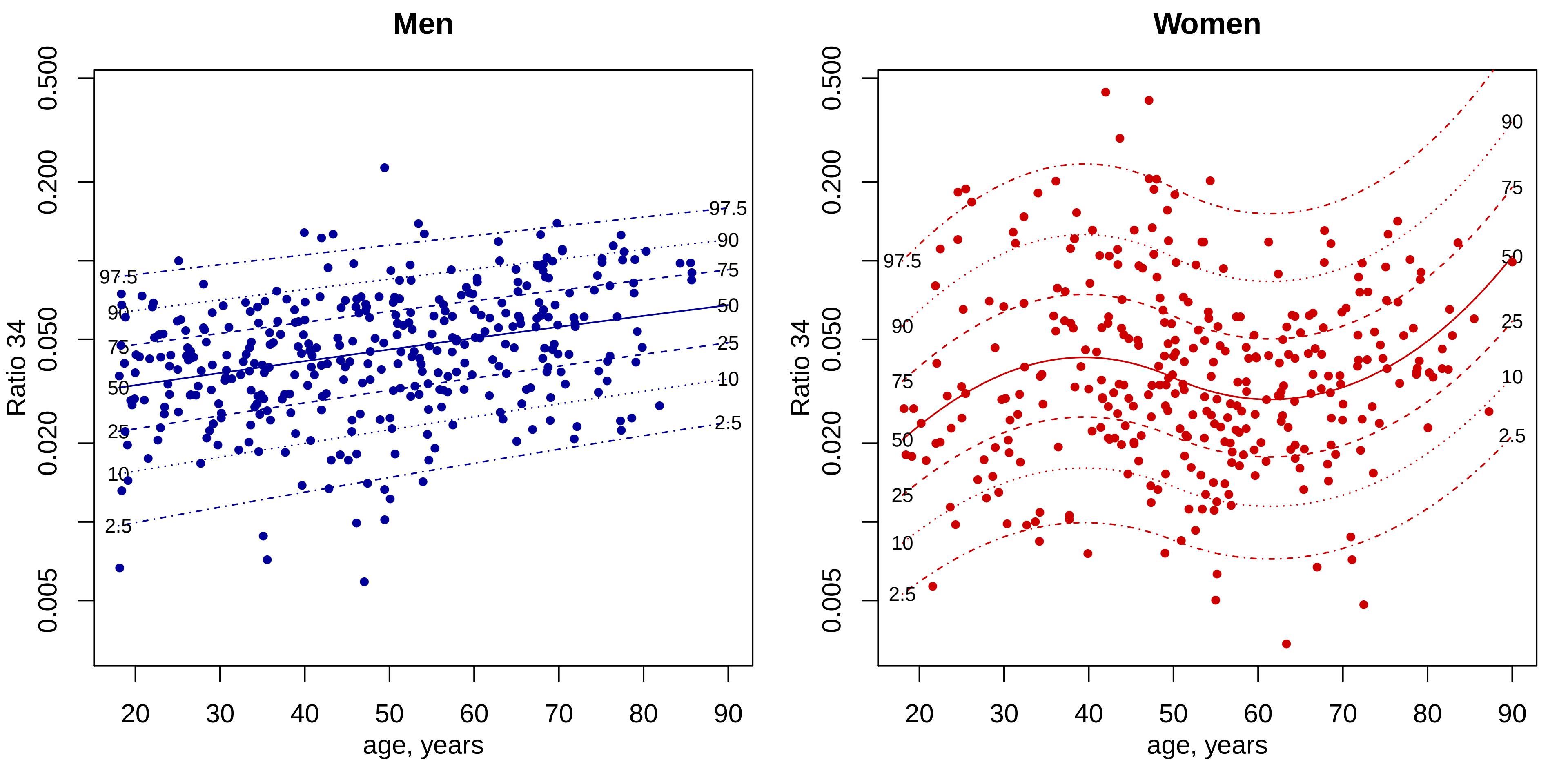

Men
Women
Ratio 34
0.500
0.200
0.050
0.020
0.005
age, years
20
30
40
50
60
70
80
90
97.5
90
75
50
25
10
2.5

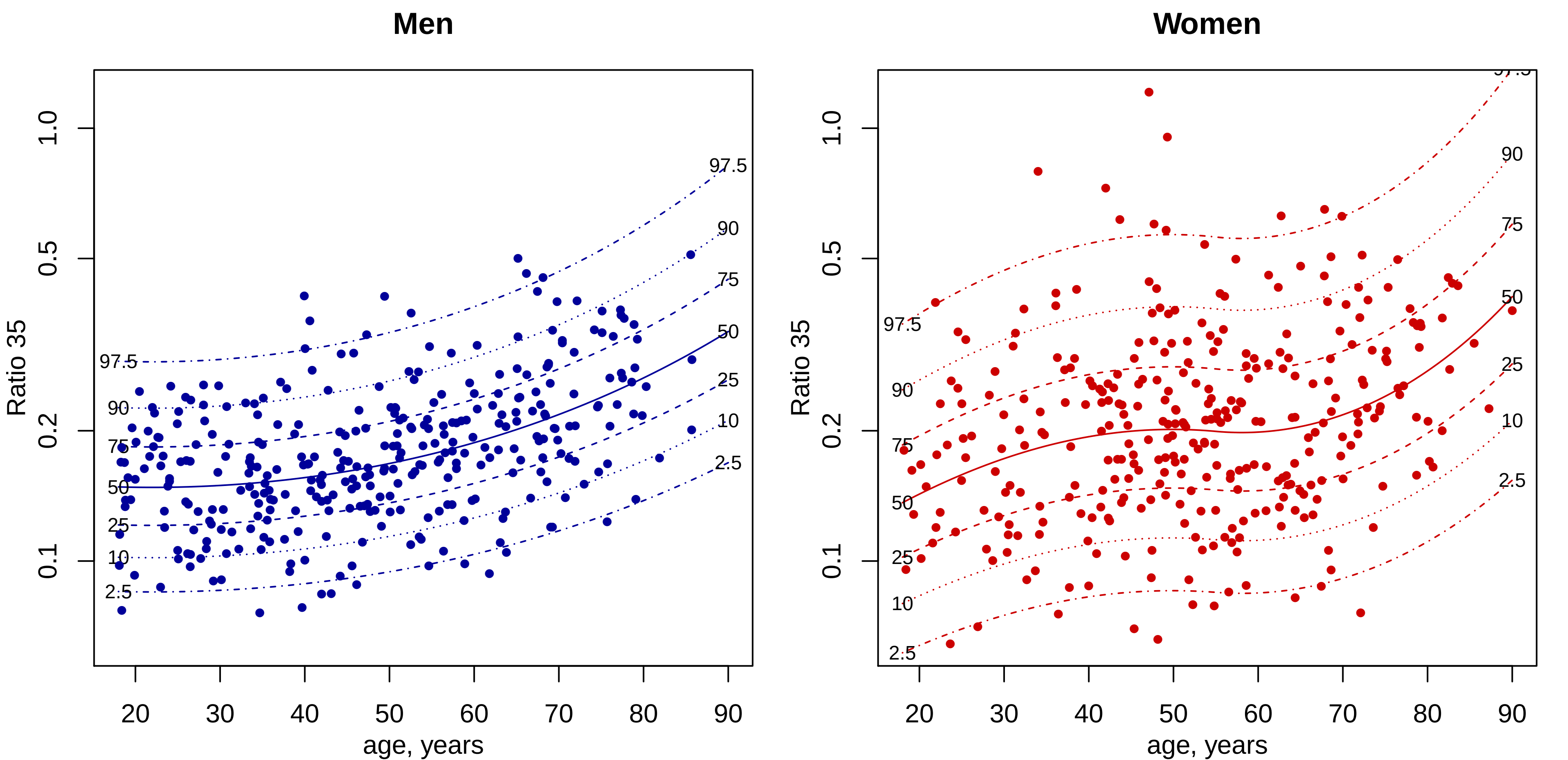

Men
Women
Ratio 35
age, years
97.5
90
75
50
25
10
2.5
0.1
0.2
0.5
1.0
20
30
40
50
60
70
80
90

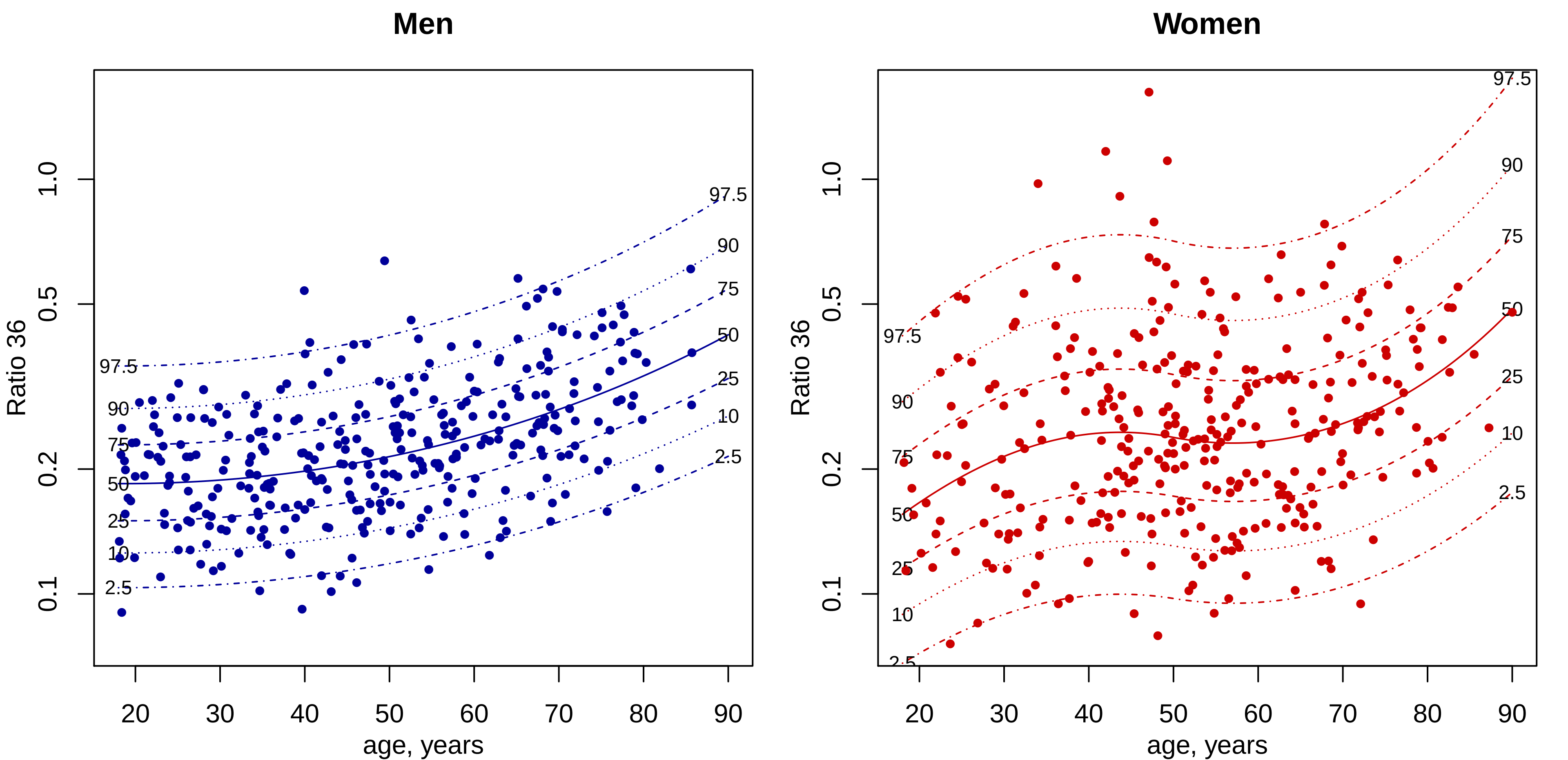

Men
Women
Ratio 36
age, years
1.0
0.5
0.2
0.1
20
30
40
50
60
70
80
90
97.5
90
75
50
25
10
2.5

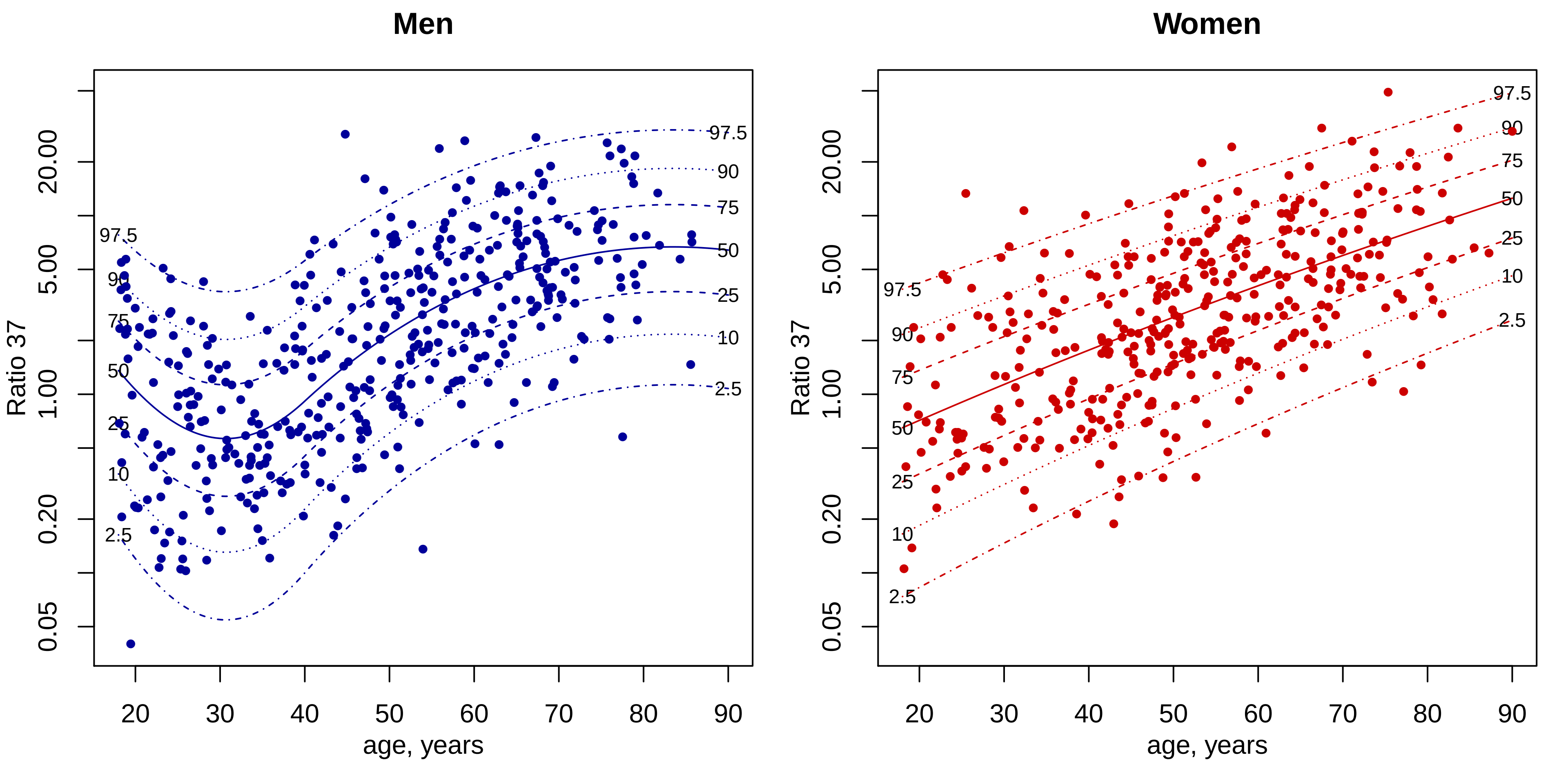
Men
Women
Ratio 37
age, years
20.00
5.00
1.00
0.20
0.05
20
30
40
50
60
70
80
90
97.5
90
75
50
25
10
2.5

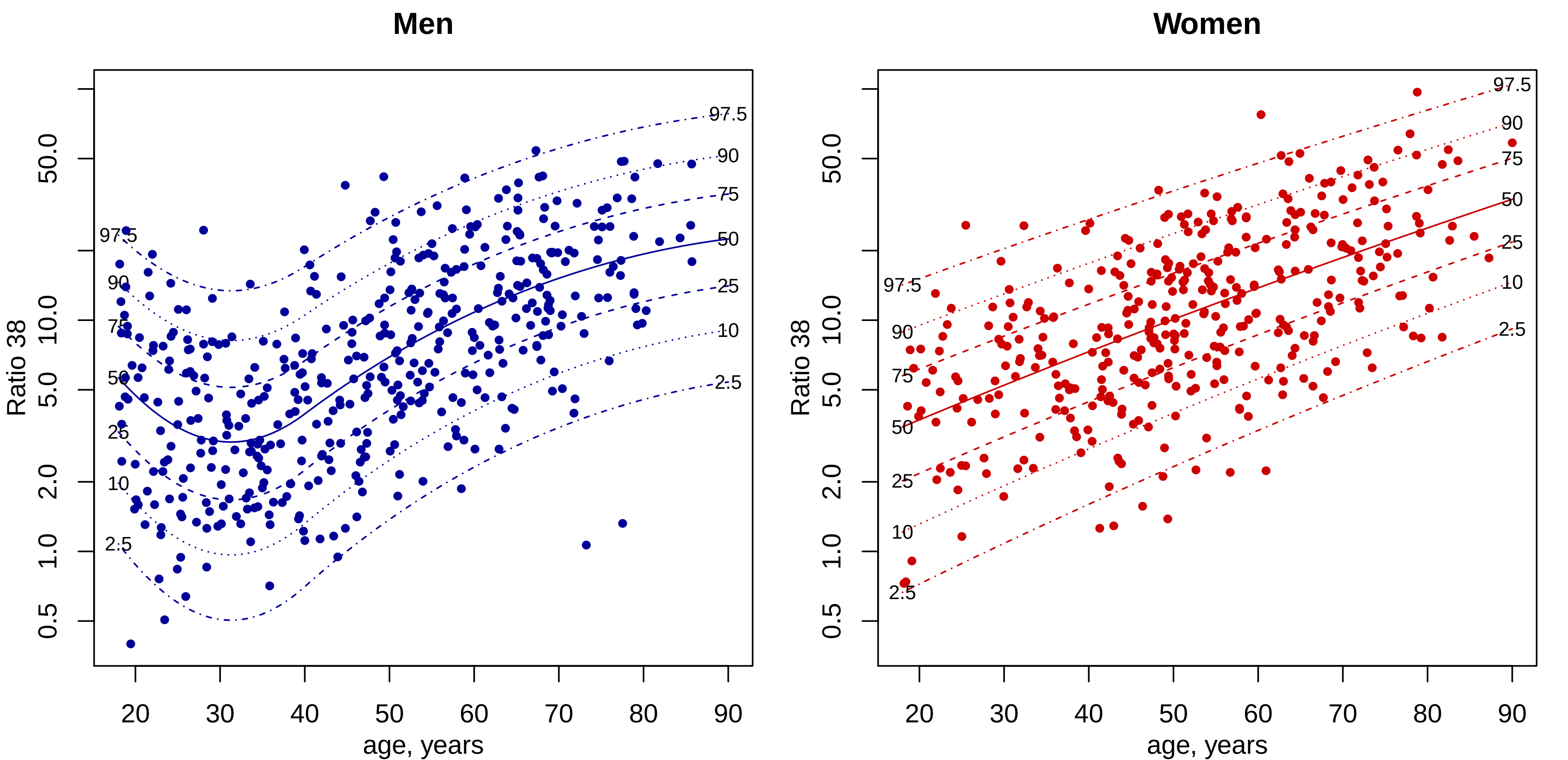
Men
Women
Ratio 38
age, years
50.0
10.0
5.0
2.0
1.0
0.5
20
30
40
50
60
70
80
90
97.5
90
75
50
25
10
2.5

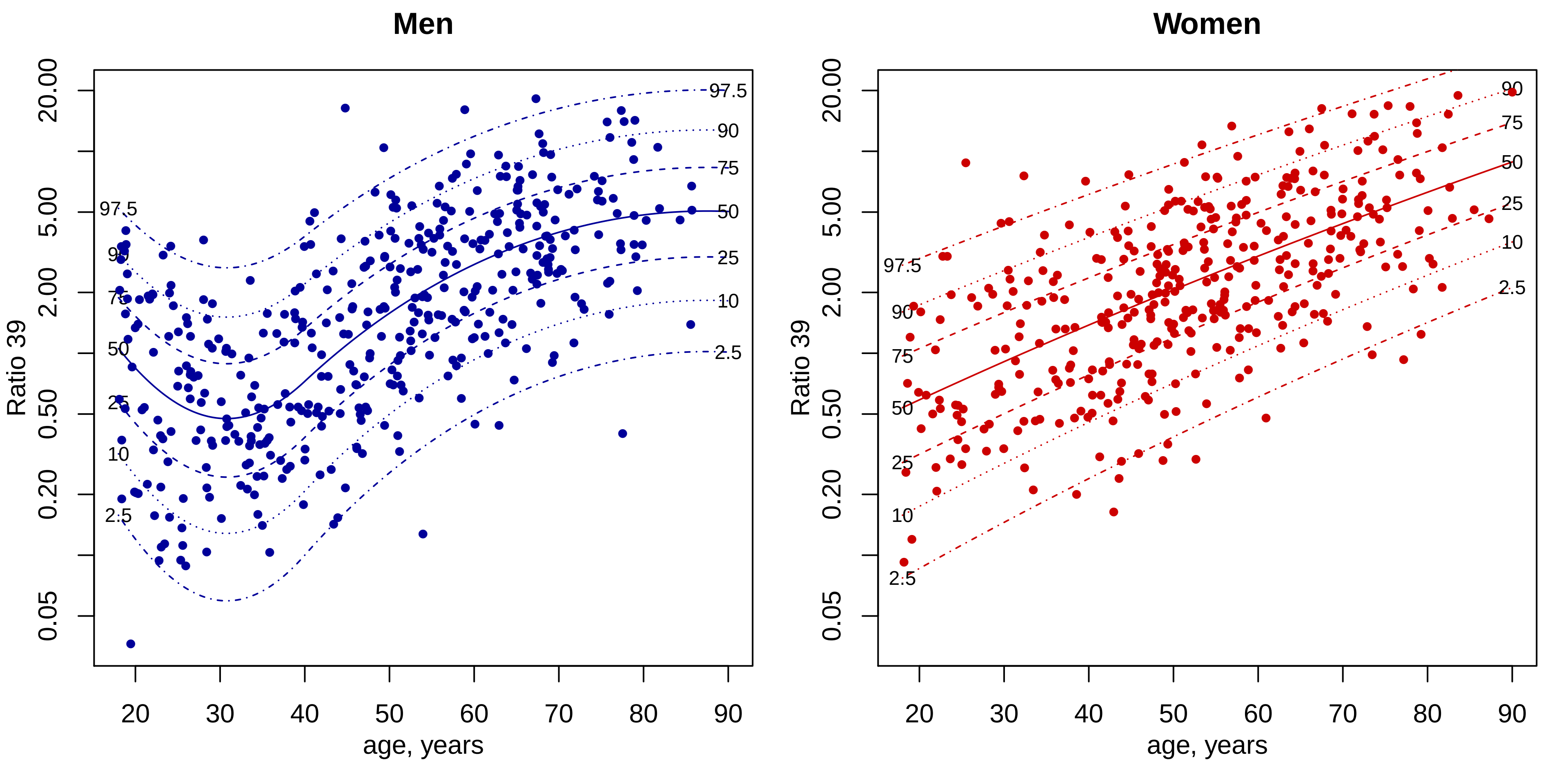

Men
Women
Ratio 39
age, years
20.00
5.00
2.00
0.50
0.20
0.05
20
30
40
50
60
70
80
90
97.5
90
75
50
25
10
2.5

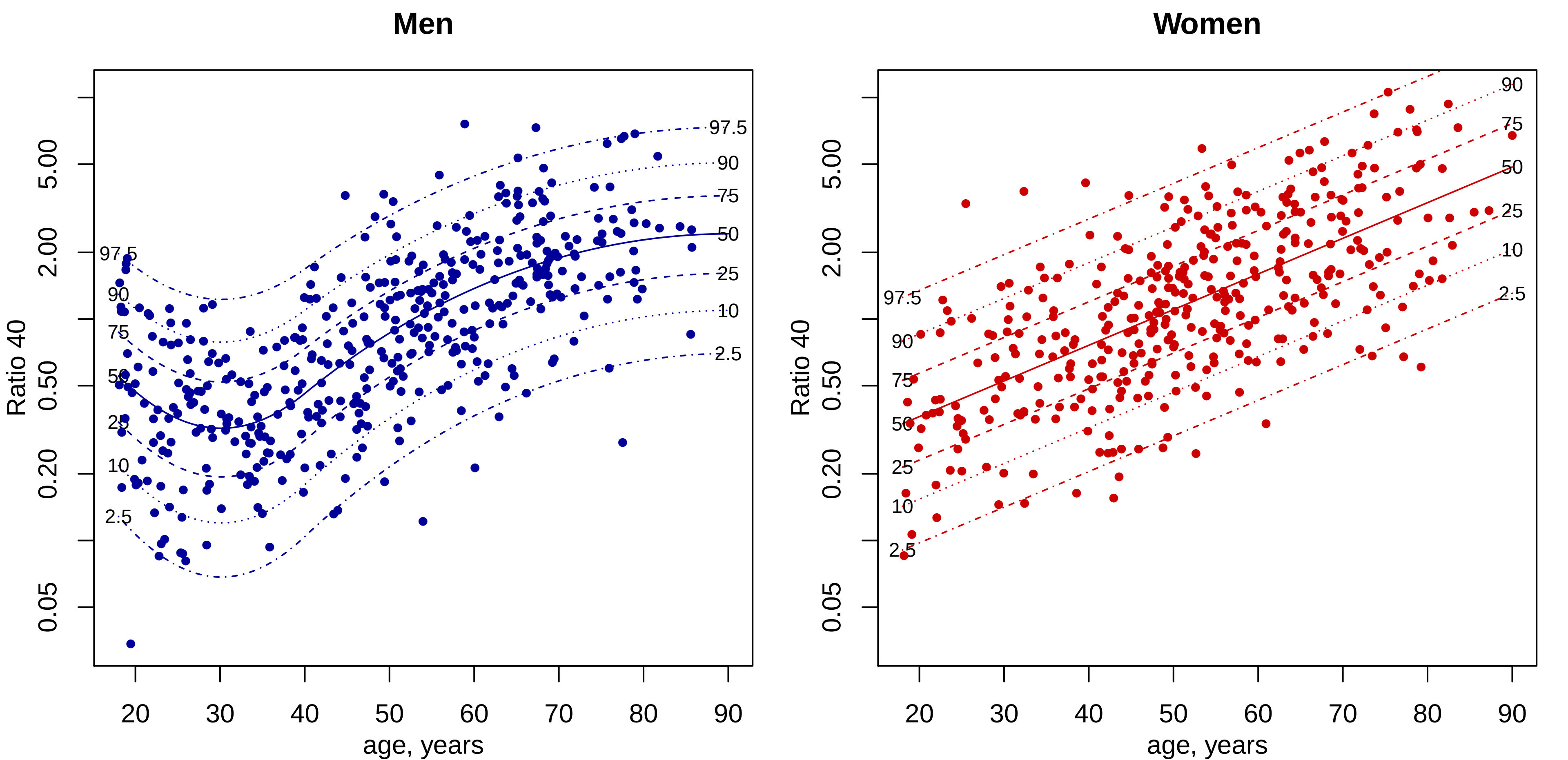

Men
Women
Ratio 40
5.00
2.00
0.50
0.20
0.05
age, years
20
30
40
50
60
70
80
90
97.5
90
75
50
25
10
2.5

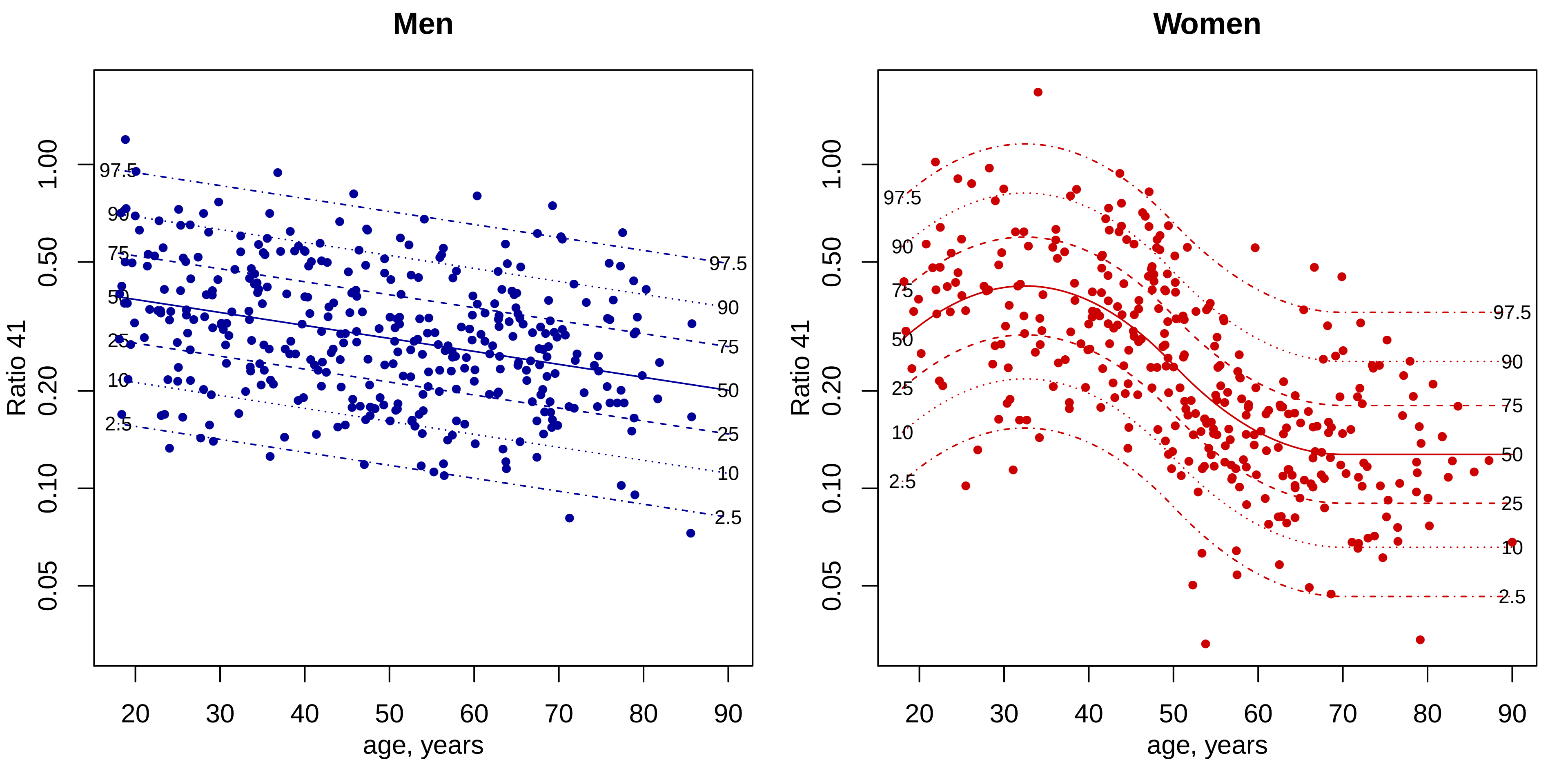

Men
Women
Ratio 41
1.00
0.50
0.20
0.10
0.05
age, years
20
30
40
50
60
70
80
90
97.5
90
75
50
25
10
2.5

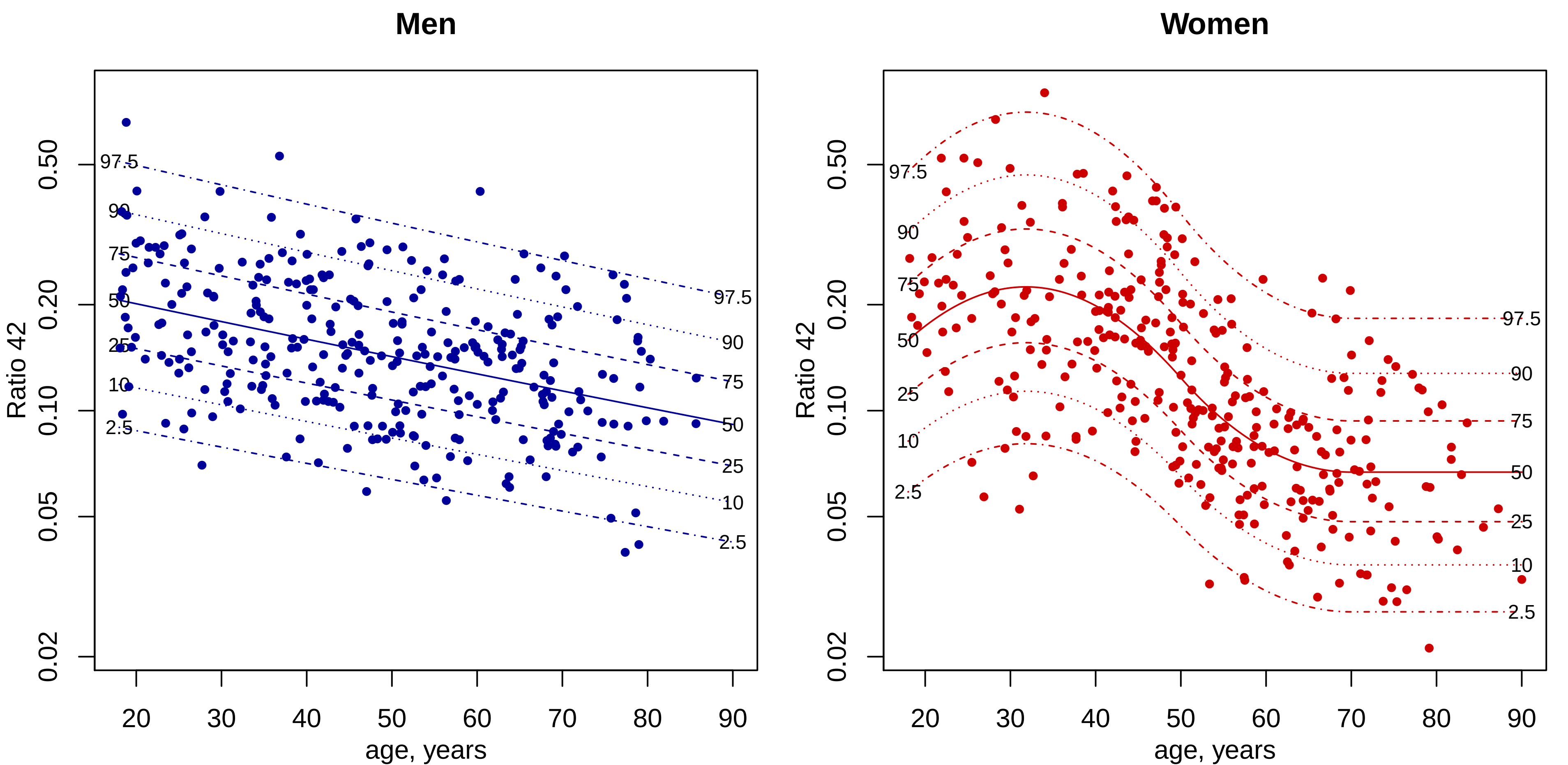
Men
Women
Ratio 42
age, years
0.02
0.05
0.10
0.20
0.50
20
30
40
50
60
70
80
90
97.5
90
75
50
25
10
2.5

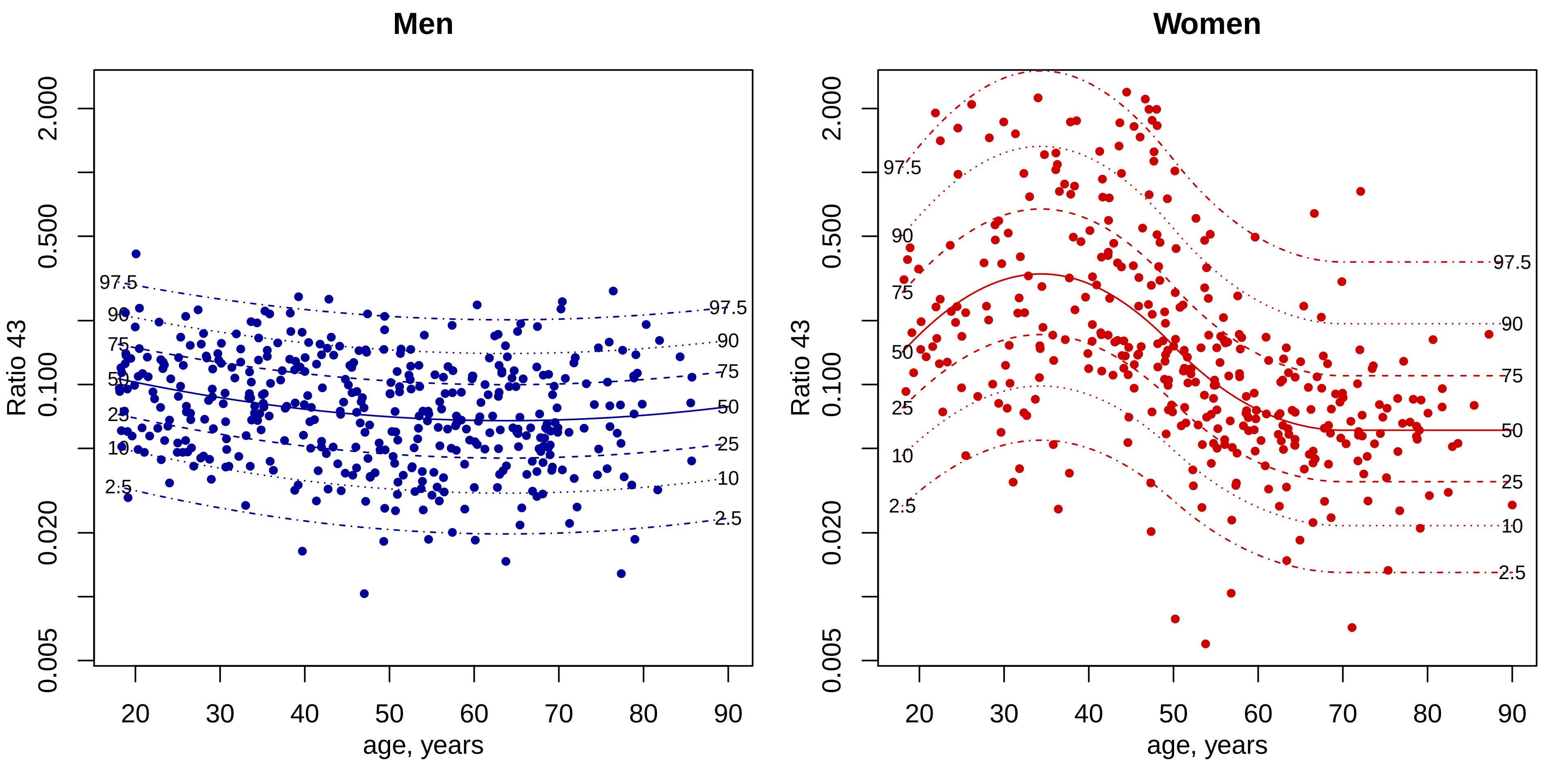

Men
Women
Ratio 43
2.000
0.500
0.100
0.020
0.005
age, years
20
30
40
50
60
70
80
90
97.5
90
75
50
25
10
2.5

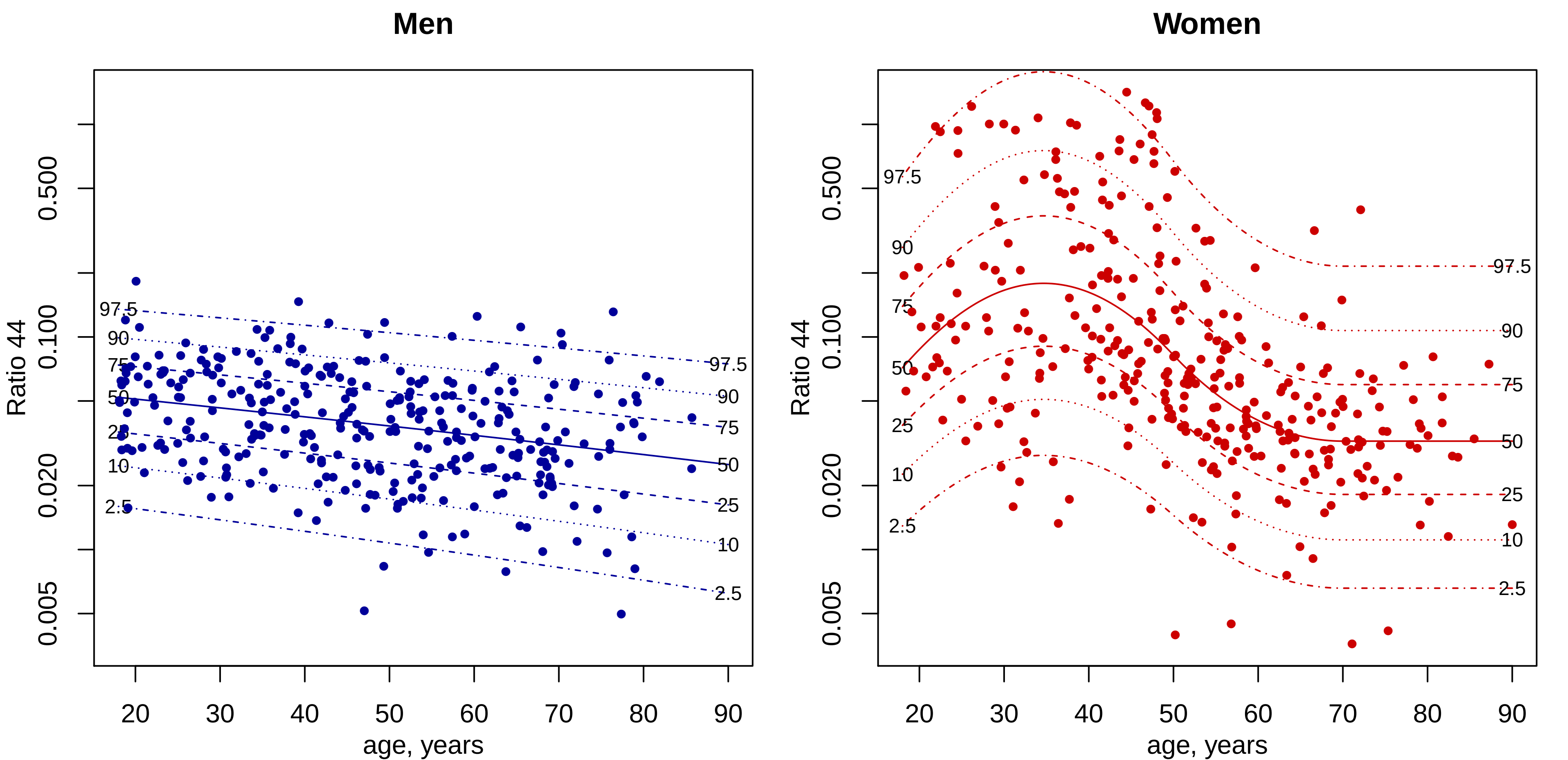
Men
Women
Ratio 44
0.005
0.020
0.100
0.500
97.5
90
75
50
25
10
2.5
20
30
40
50
60
70
80
90
age, years

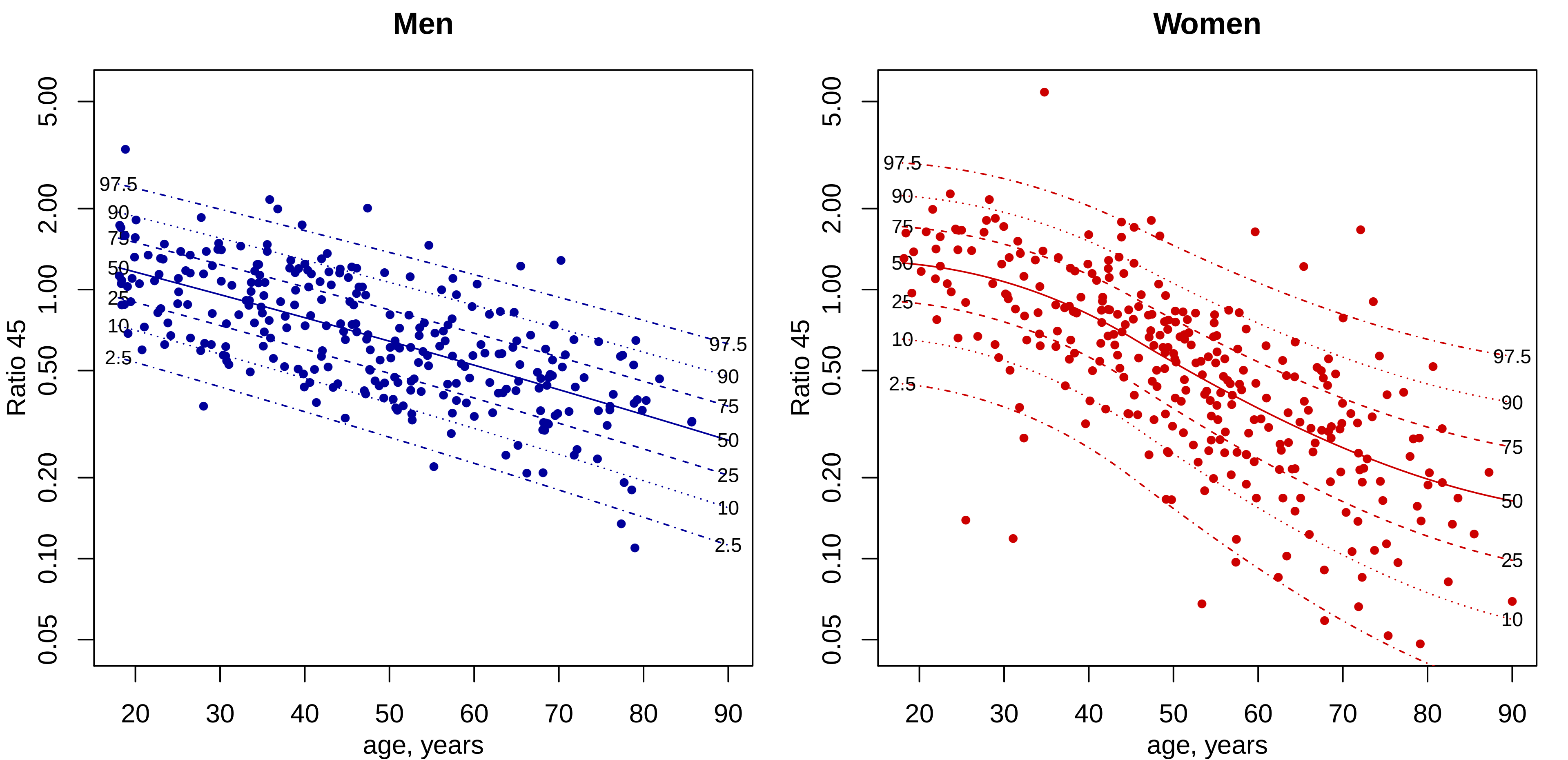

Men
Women
Ratio 45
age, years
5.00
2.00
1.00
0.50
0.20
0.10
0.05
20
30
40
50
60
70
80
90
97.5
90
75
50
25
10
2.5

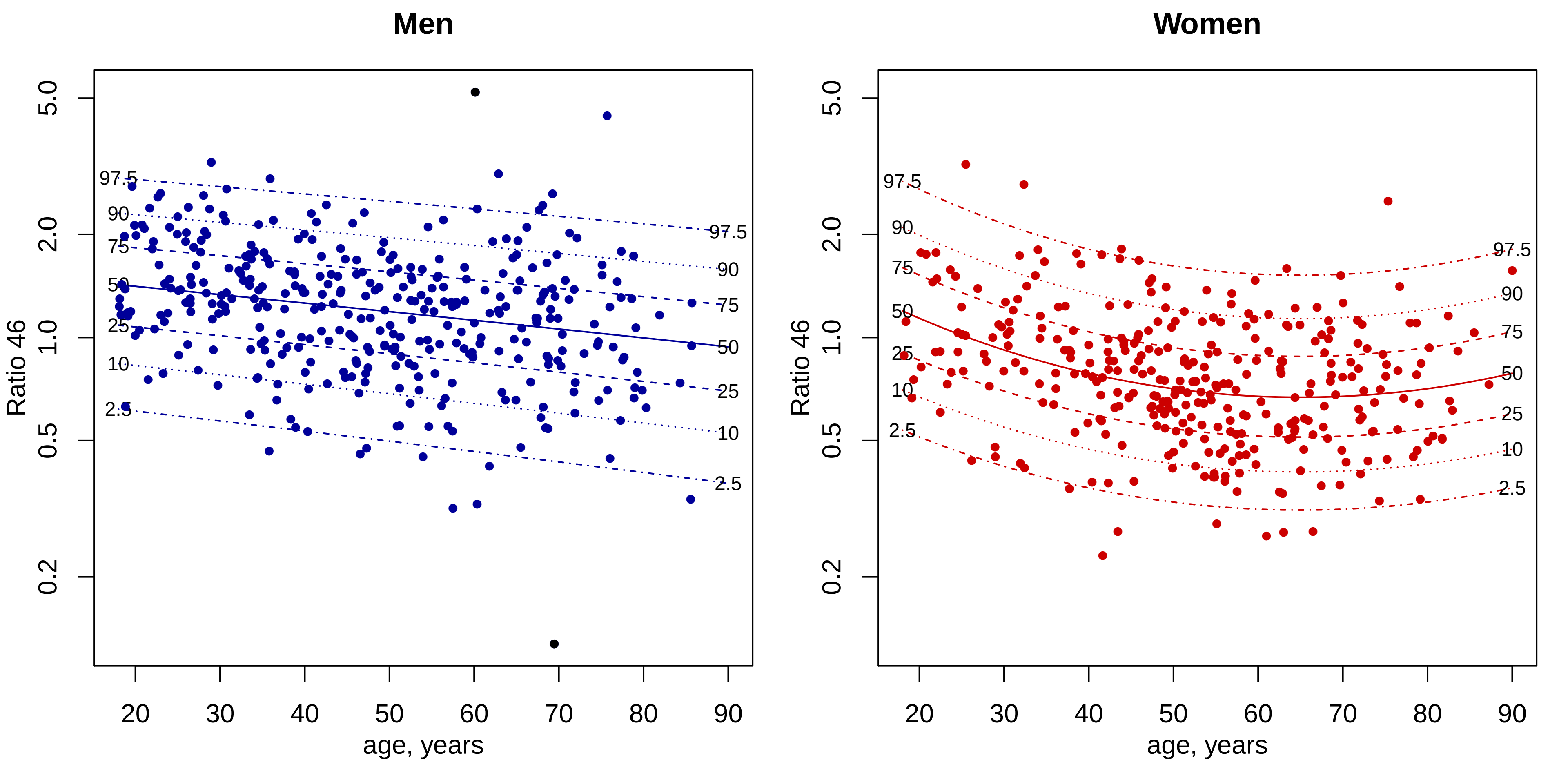

Men
Women
Ratio 46
5.0
2.0
1.0
0.5
0.2
20
30
40
50
60
70
80
90
age, years
97.5
90
75
50
25
10
2.5

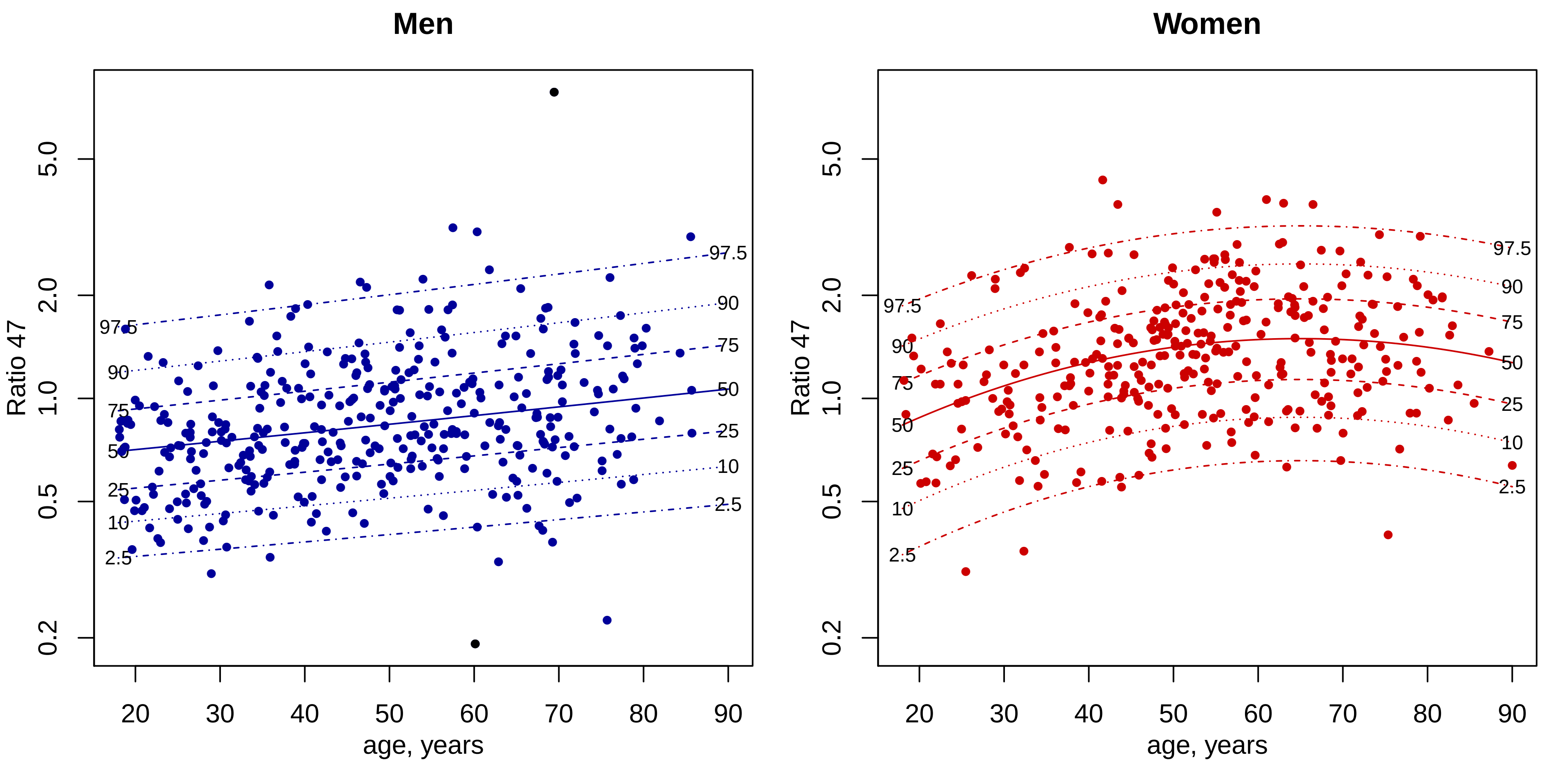

Men
Women
Ratio 47
age, years
0.2
0.5
1.0
2.0
5.0
20
30
40
50
60
70
80
90
97.5
90
75
50
25
10
2.5

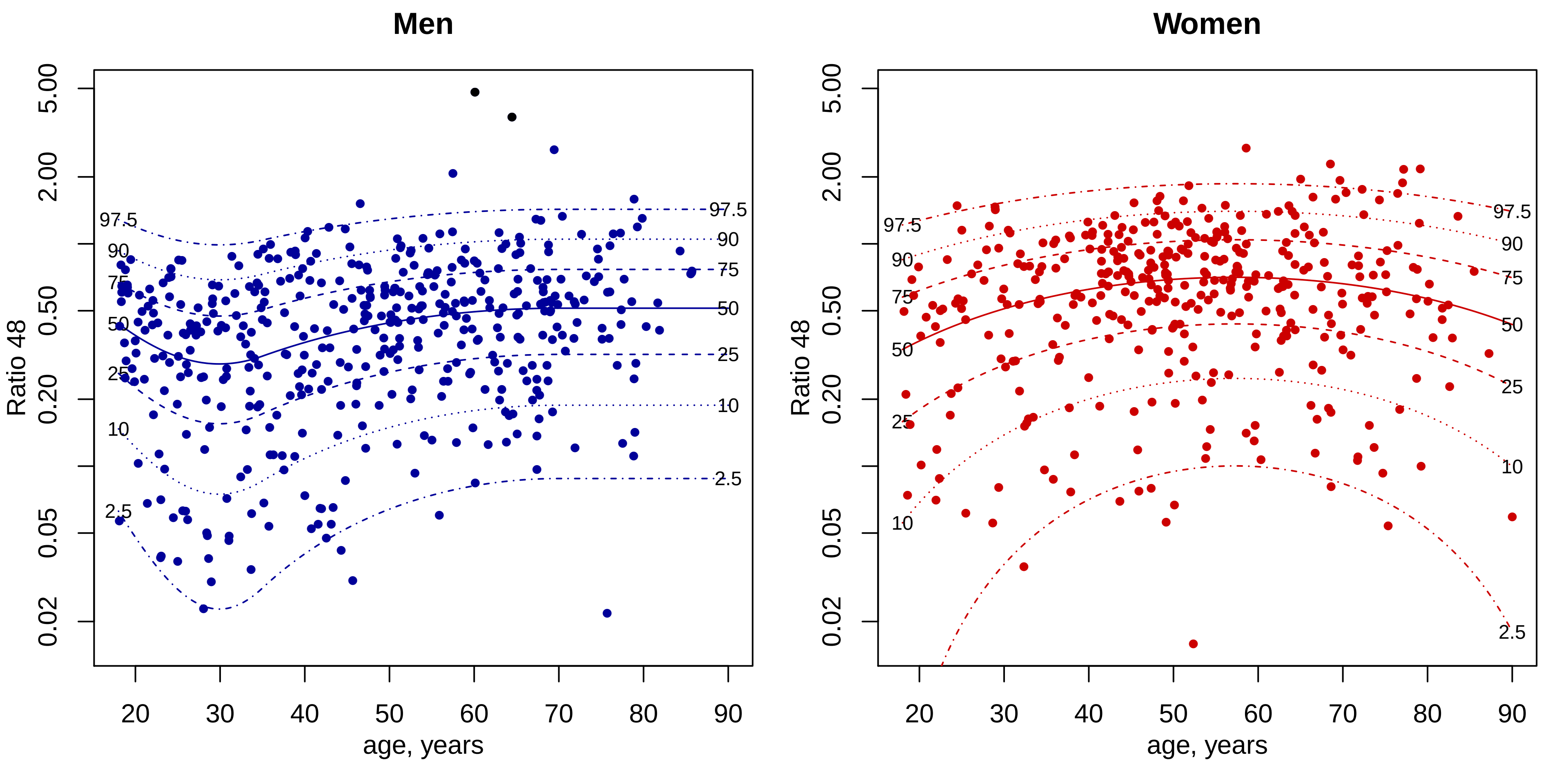

Men
Women
Ratio 48
age, years
5.00
2.00
0.50
0.20
0.05
0.02
20
30
40
50
60
70
80
90
97.5
90
75
50
25
10
2.5

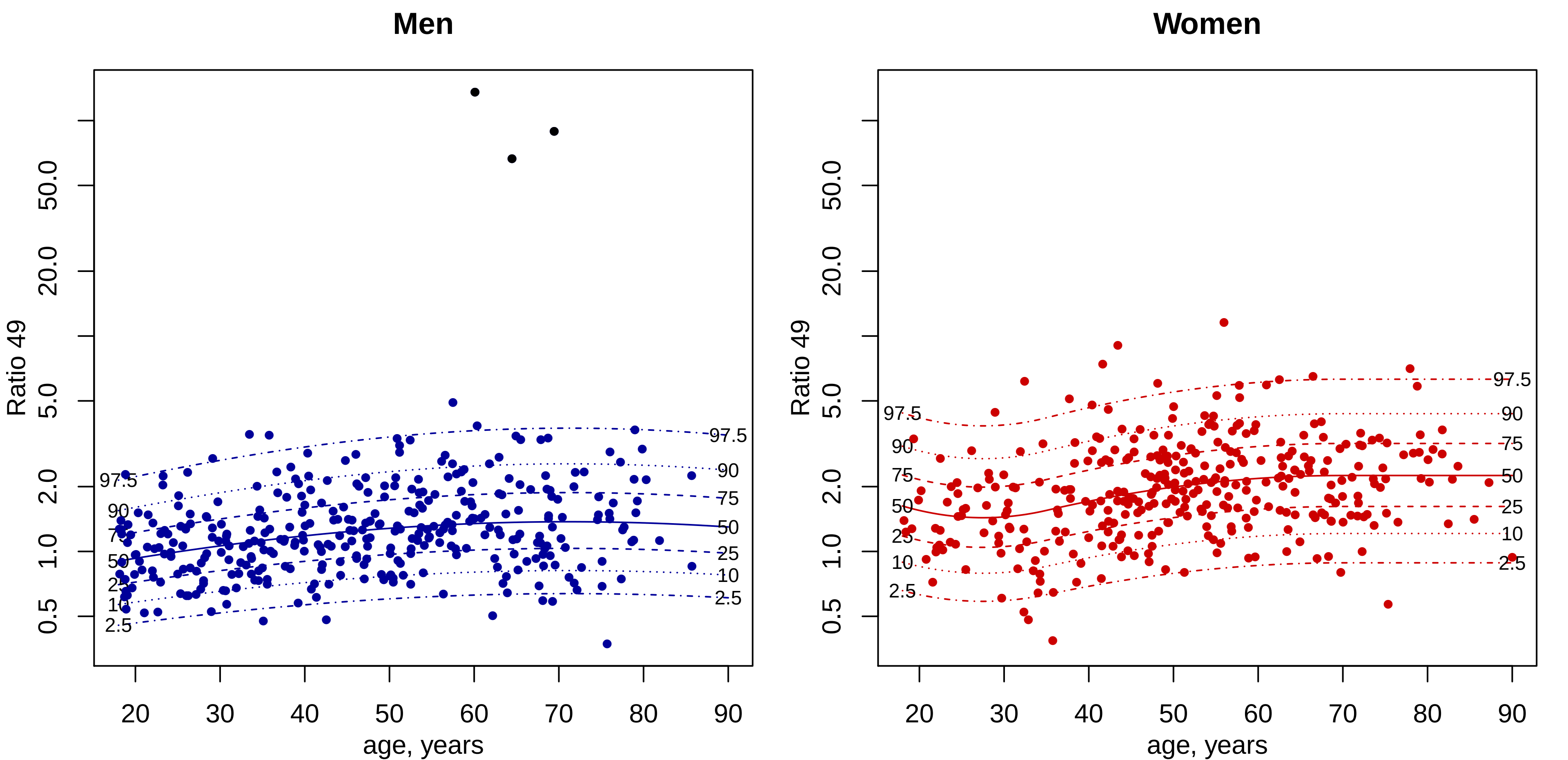
Men
Women
Ratio 49
Ratio 49
0.5
1.0
2.0
5.0
20.0
50.0
age, years
age, years
20
30
40
50
60
70
80
90
97.5
90
75
50
25
10
2.5

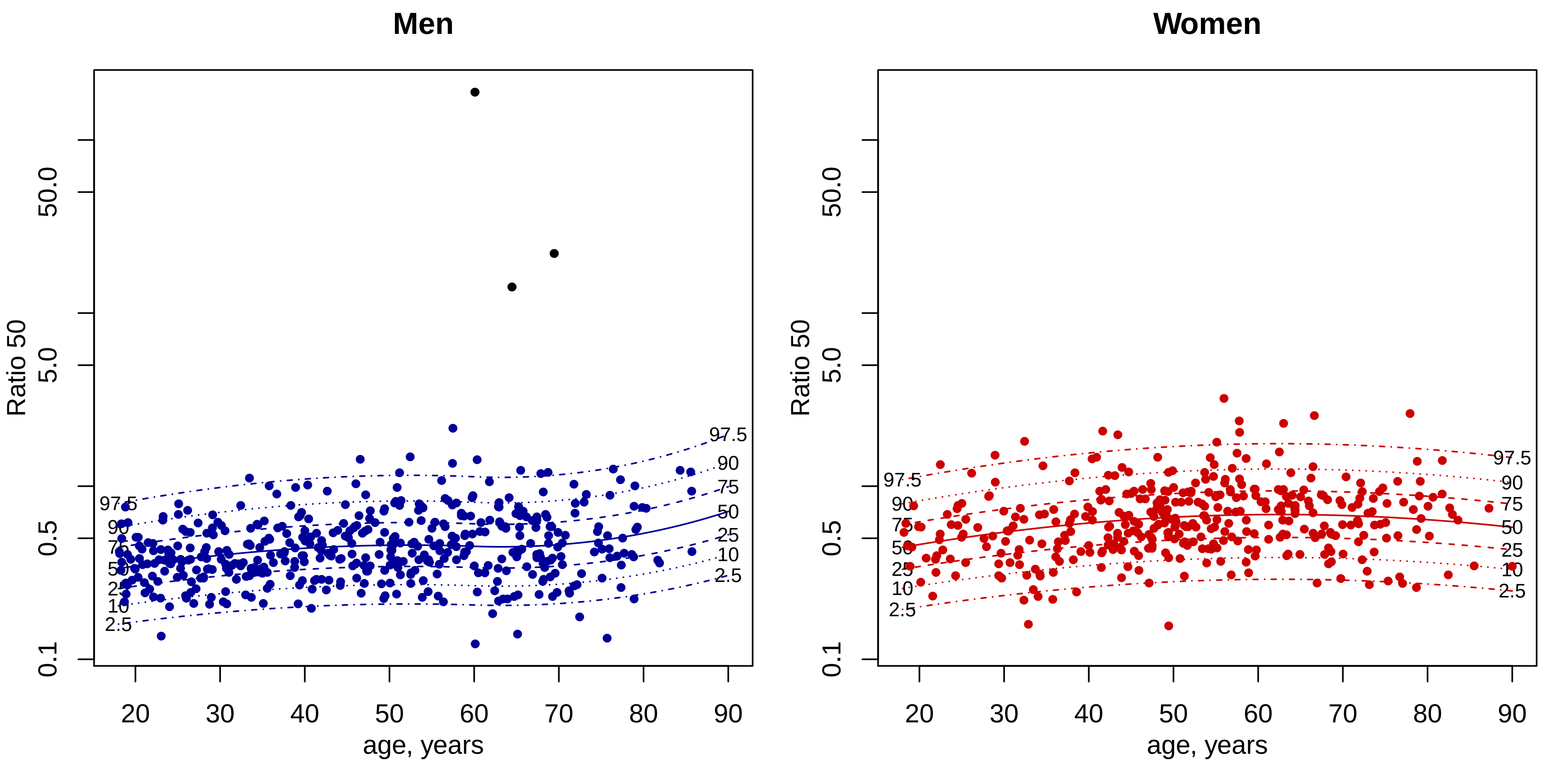
Men
Women
Ratio 50
age, years
50.0
5.0
0.5
0.1
20
30
40
50
60
70
80
90
97.5
90
75
50
25
10
2.5

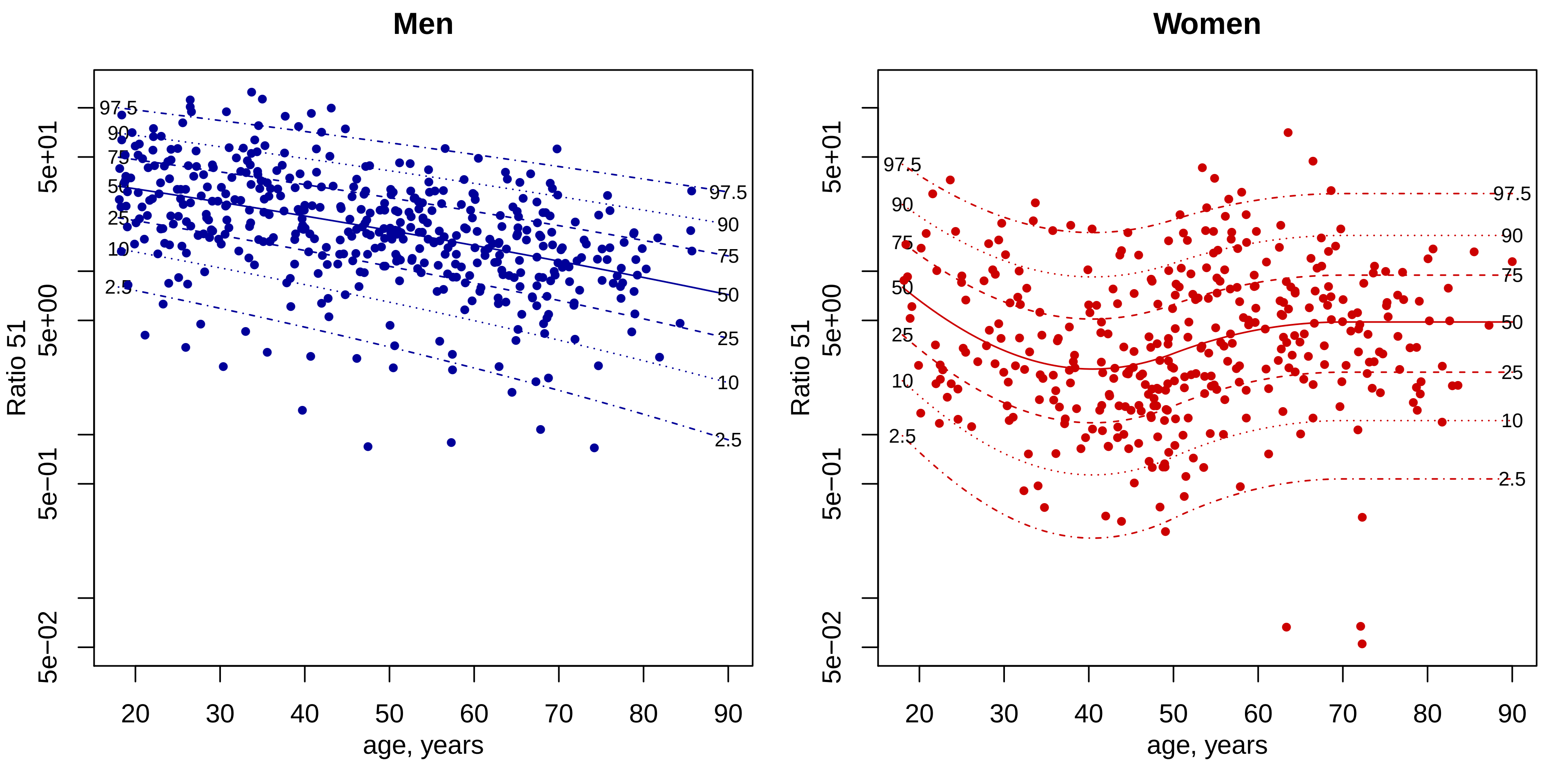

Men
Women
Ratio 51
5e+01
5e+00
5e−01
5e−02
age, years
20
30
40
50
60
70
80
90
97.5
90
75
50
25
10
2.5

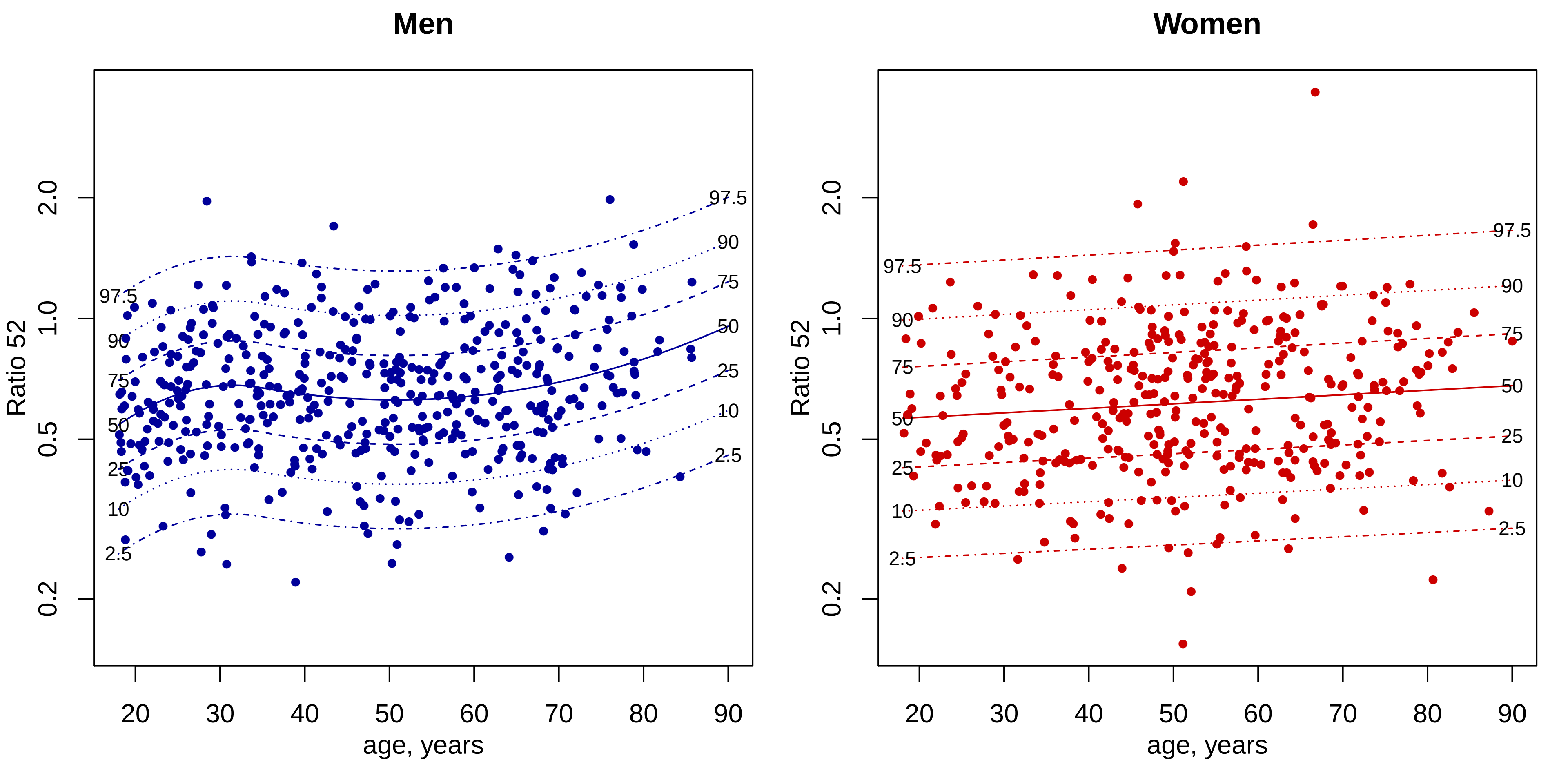

Men
Women
Ratio 52
0.2
0.5
1.0
2.0
age, years
20
30
40
50
60
70
80
90
97.5
90
75
50
25
10
2.5

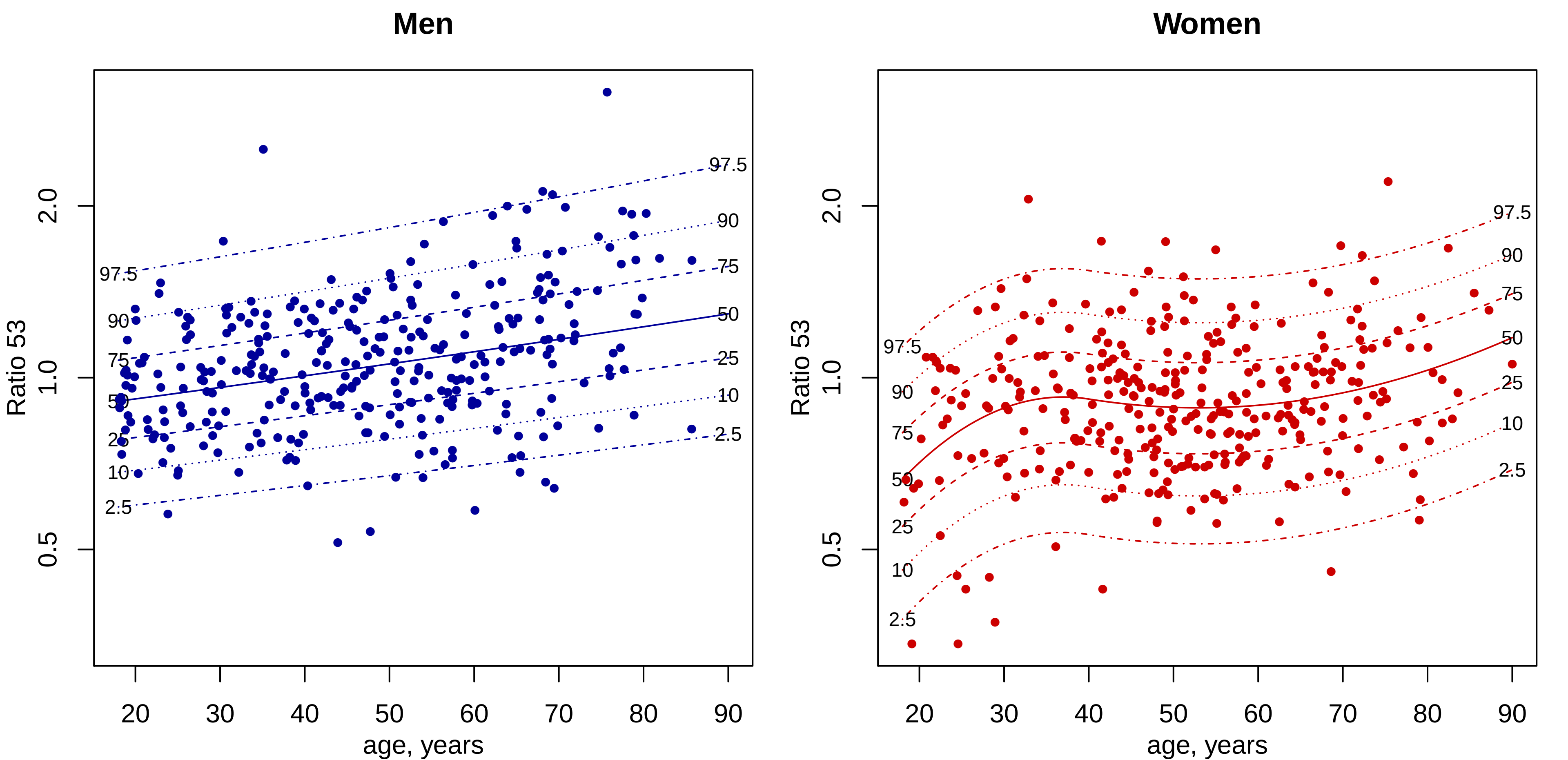

Men
Women
Ratio 53
age, years
2.0
1.0
0.5
20
30
40
50
60
70
80
90
97.5
90
75
50
25
10
2.5

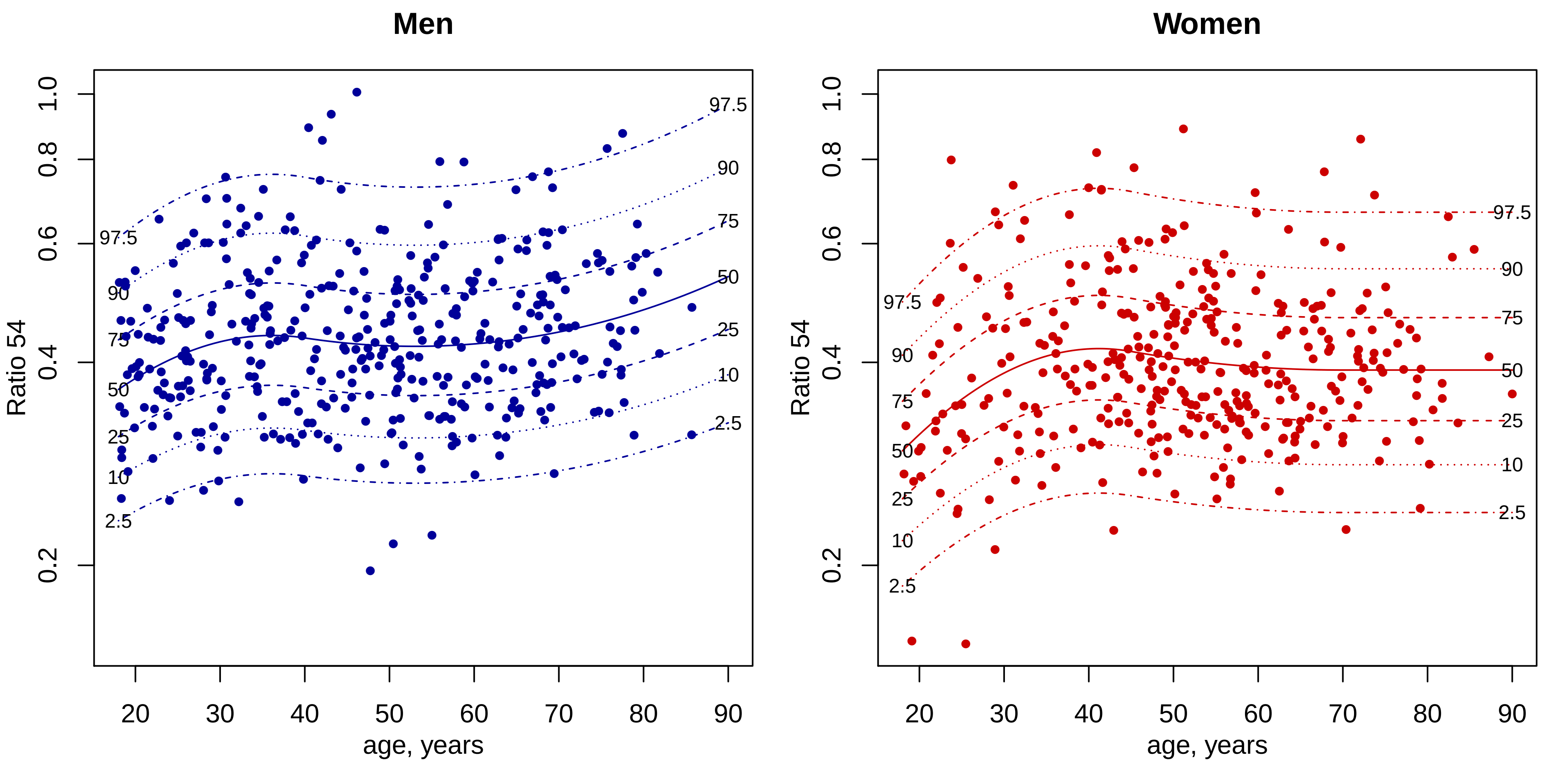

Men
Women
Ratio 54
age, years
1.0
0.8
0.6
0.4
0.2
20
30
40
50
60
70
80
90
97.5
90
75
50
25
10
2.5

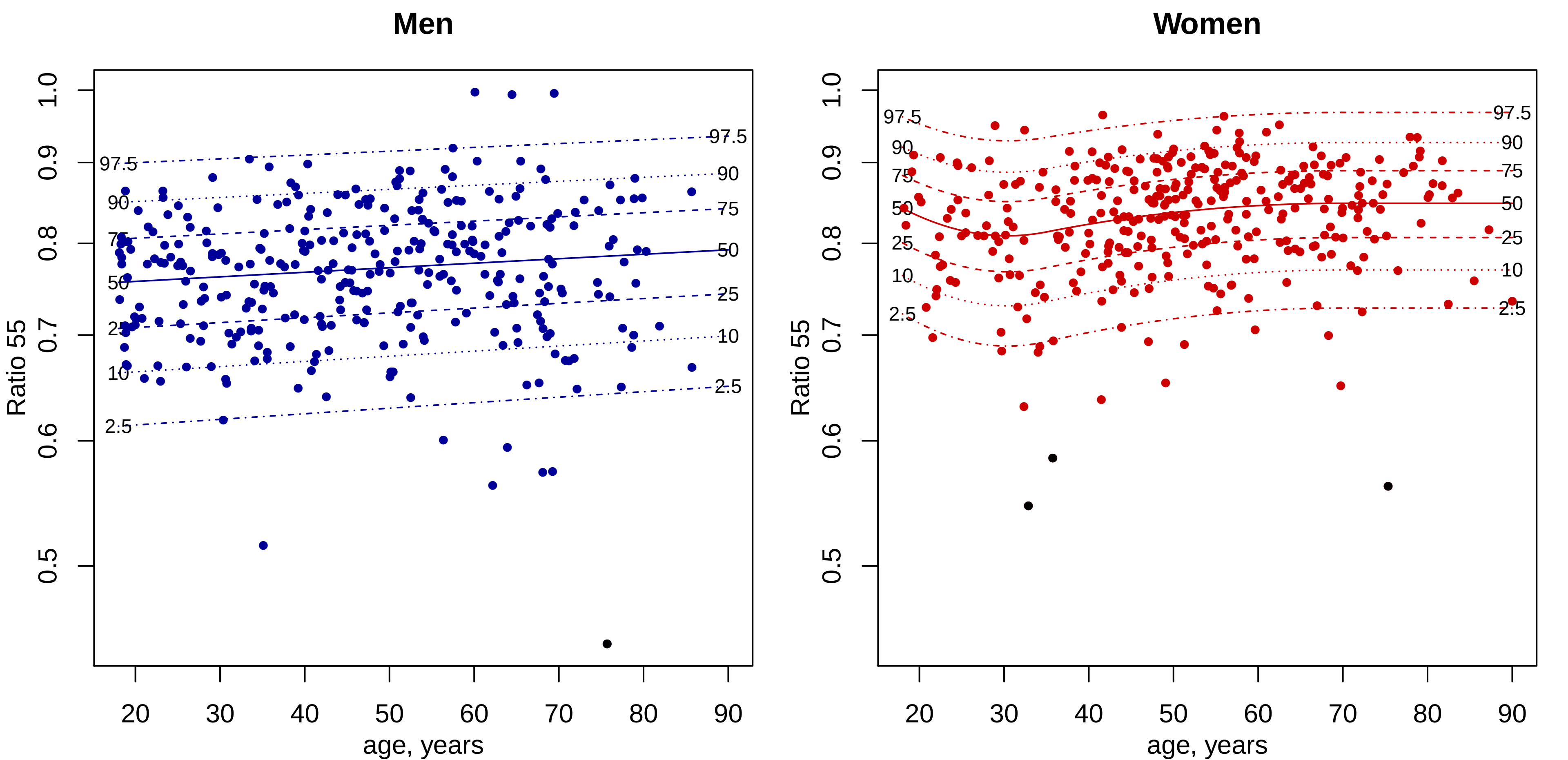
Men
Women
Ratio 55
age, years
97.5
90
75
50
25
10
2.5

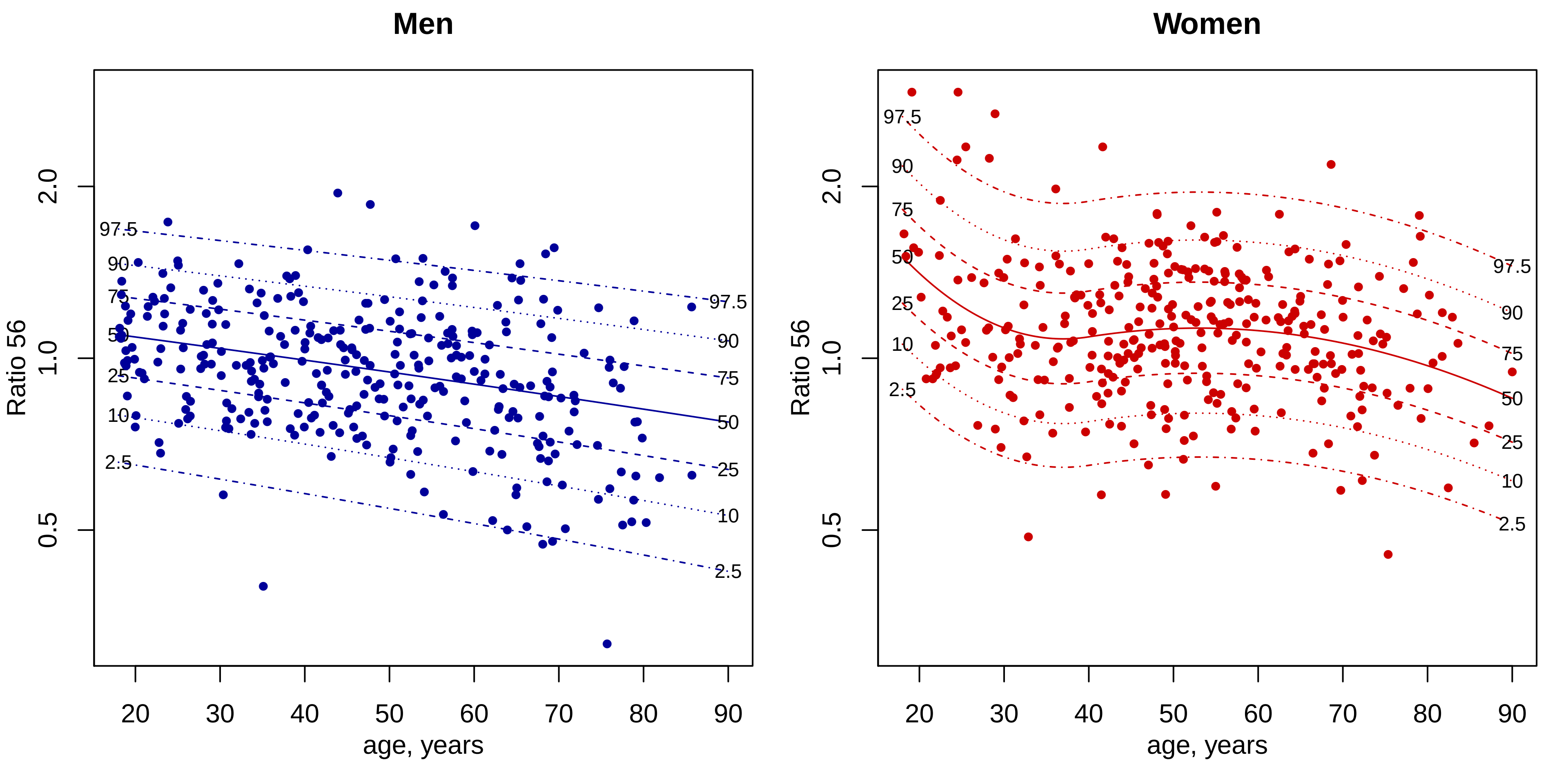
Men
Women
Ratio 56
age, years
0.5
1.0
2.0
20
30
40
50
60
70
80
90
97.5
90
75
50
25
10
2.5

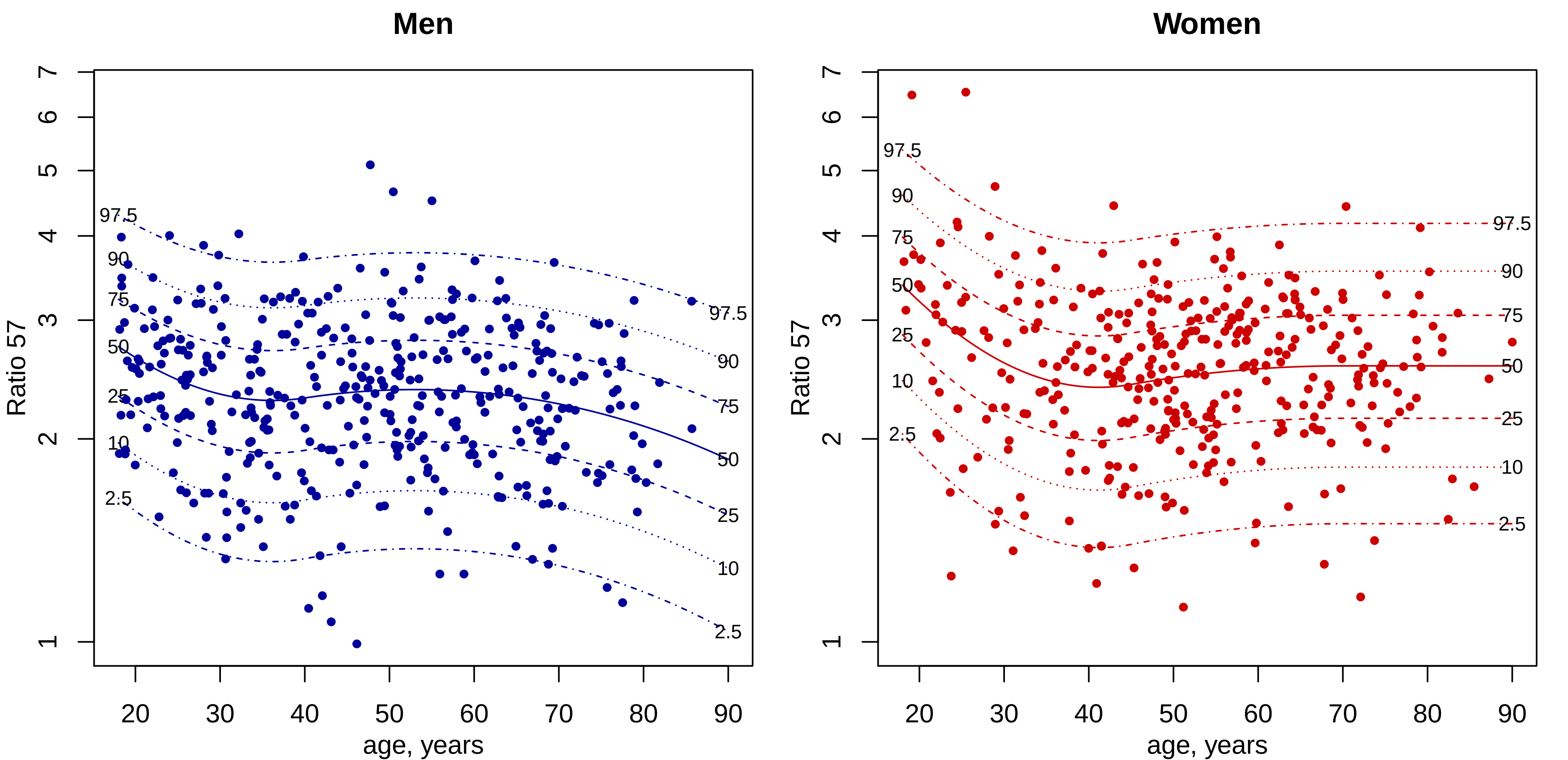
Men
Women
Ratio 57
age, years
97.5
90
75
50
25
10
2.5
1
2
3
4
5
6
7
20
30
40
50
60
70
80
90

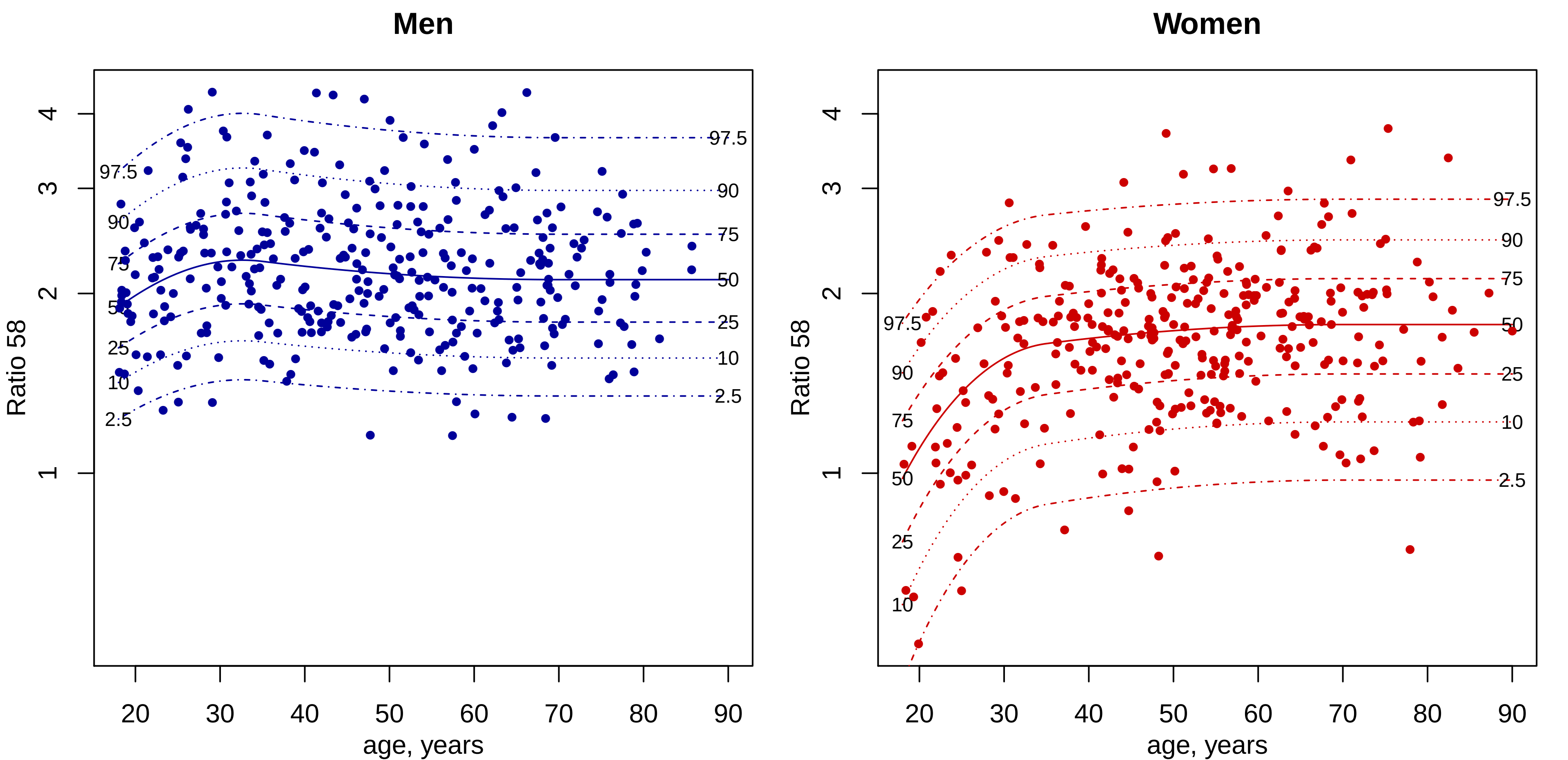
Men
Women
Ratio 58
age, years
97.5
90
75
50
25
10
2.5

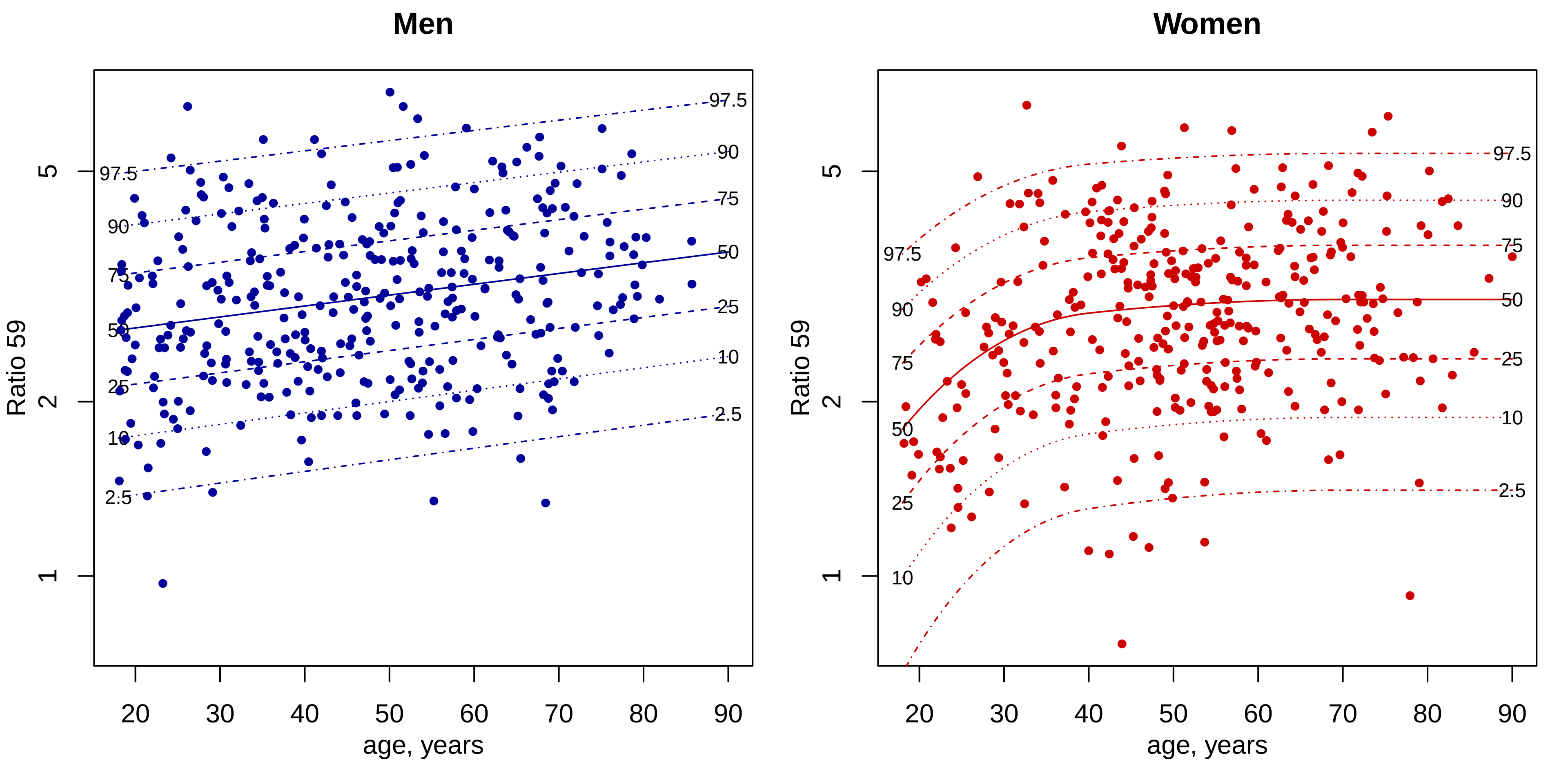

Men
Women
Ratio 59
age, years
97.5
90
75
50
25
10
2.5

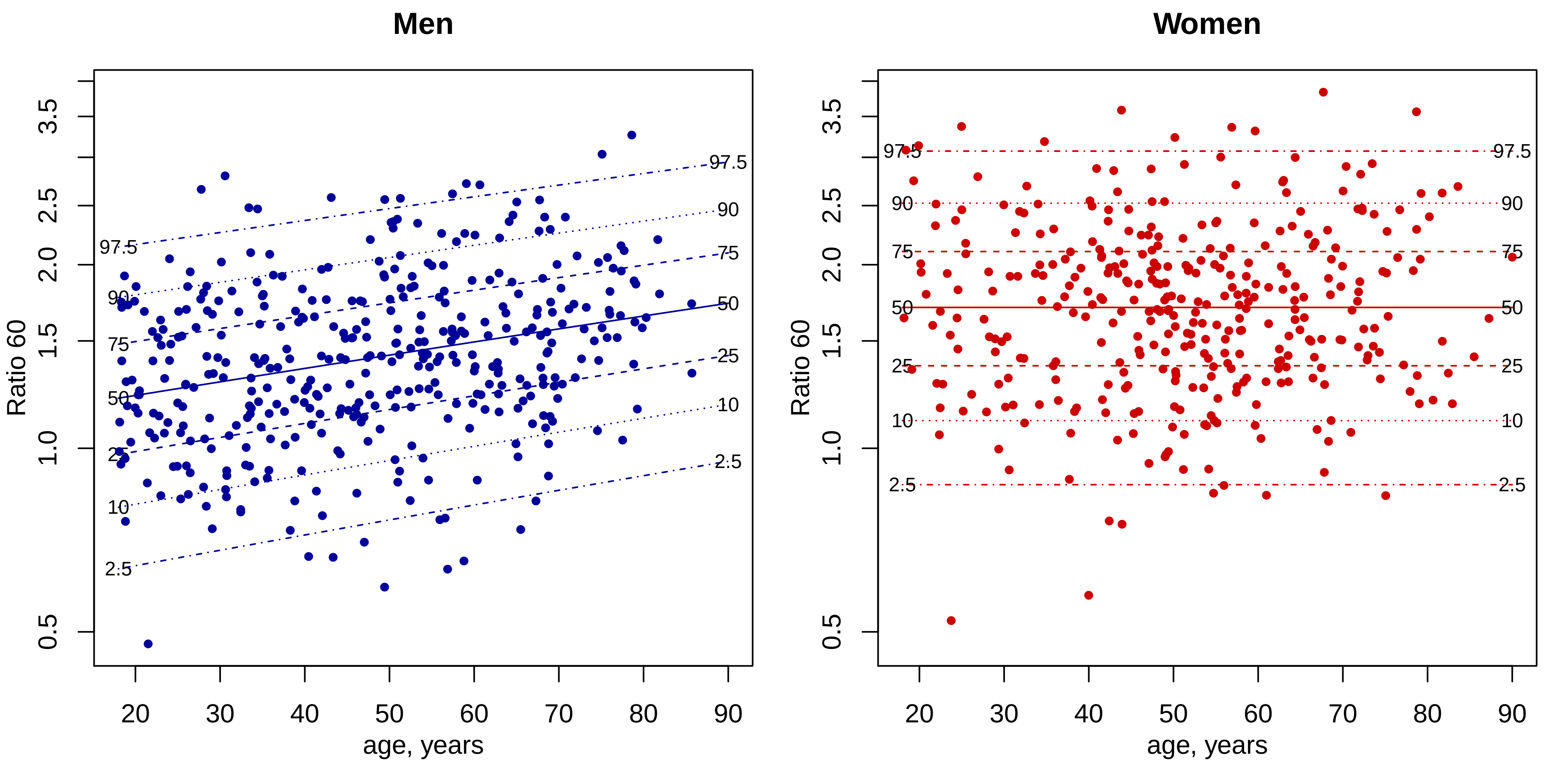

Men
Women
Ratio 60
age, years
0.5
1.0
1.5
2.0
2.5
3.5
20
30
40
50
60
70
80
90
97.5
90
75
50
25
10
2.5

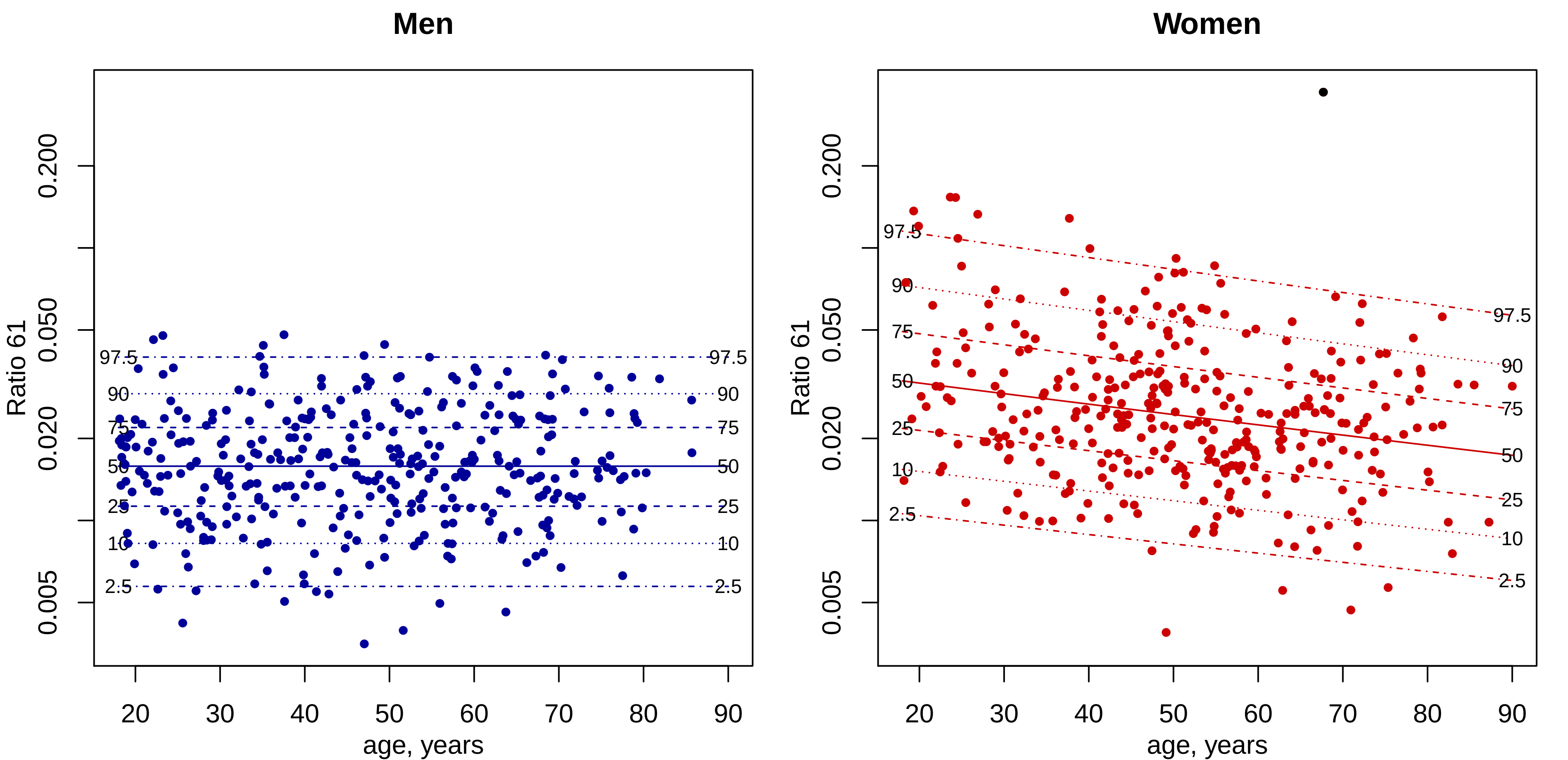
Men
Women
Ratio 61
0.200
0.050
0.020
0.005
97.5
90
75
50
25
10
2.5
20
30
40
50
60
70
80
90
age, years

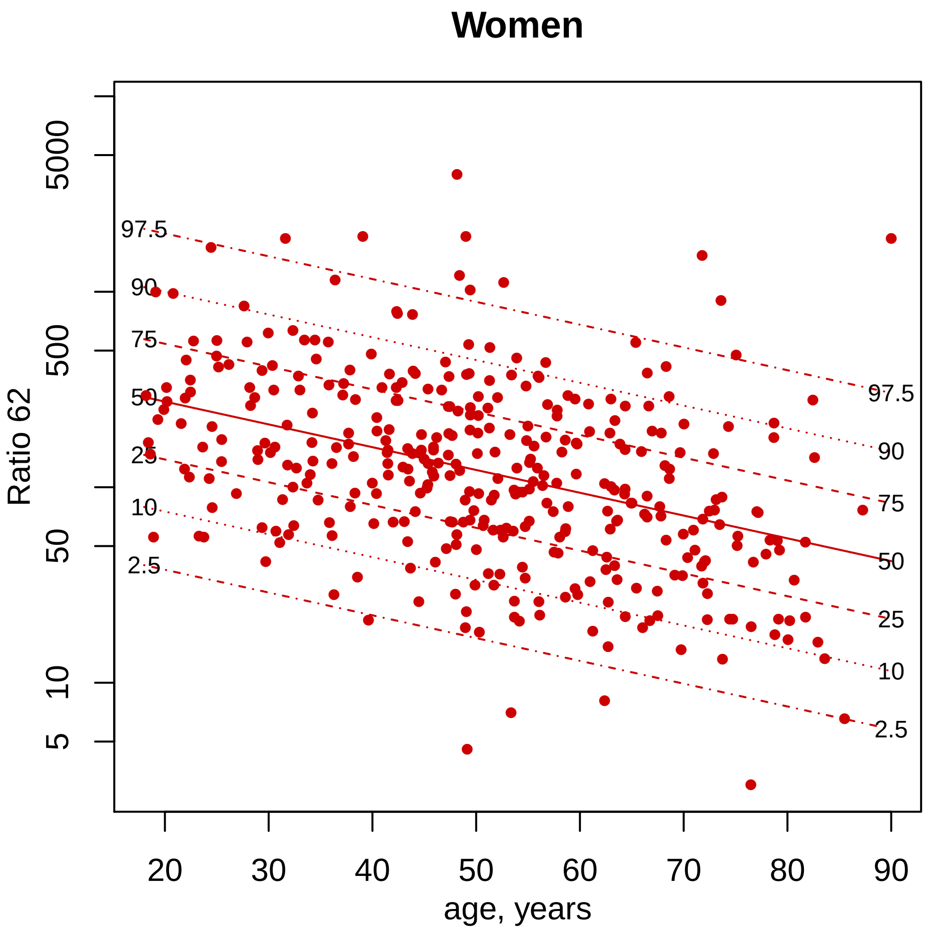

Women
Ratio 62
5000
500
50
10
5
97.5
90
75
50
25
10
2.5
20
30
40
50
60
70
80
90
age, years

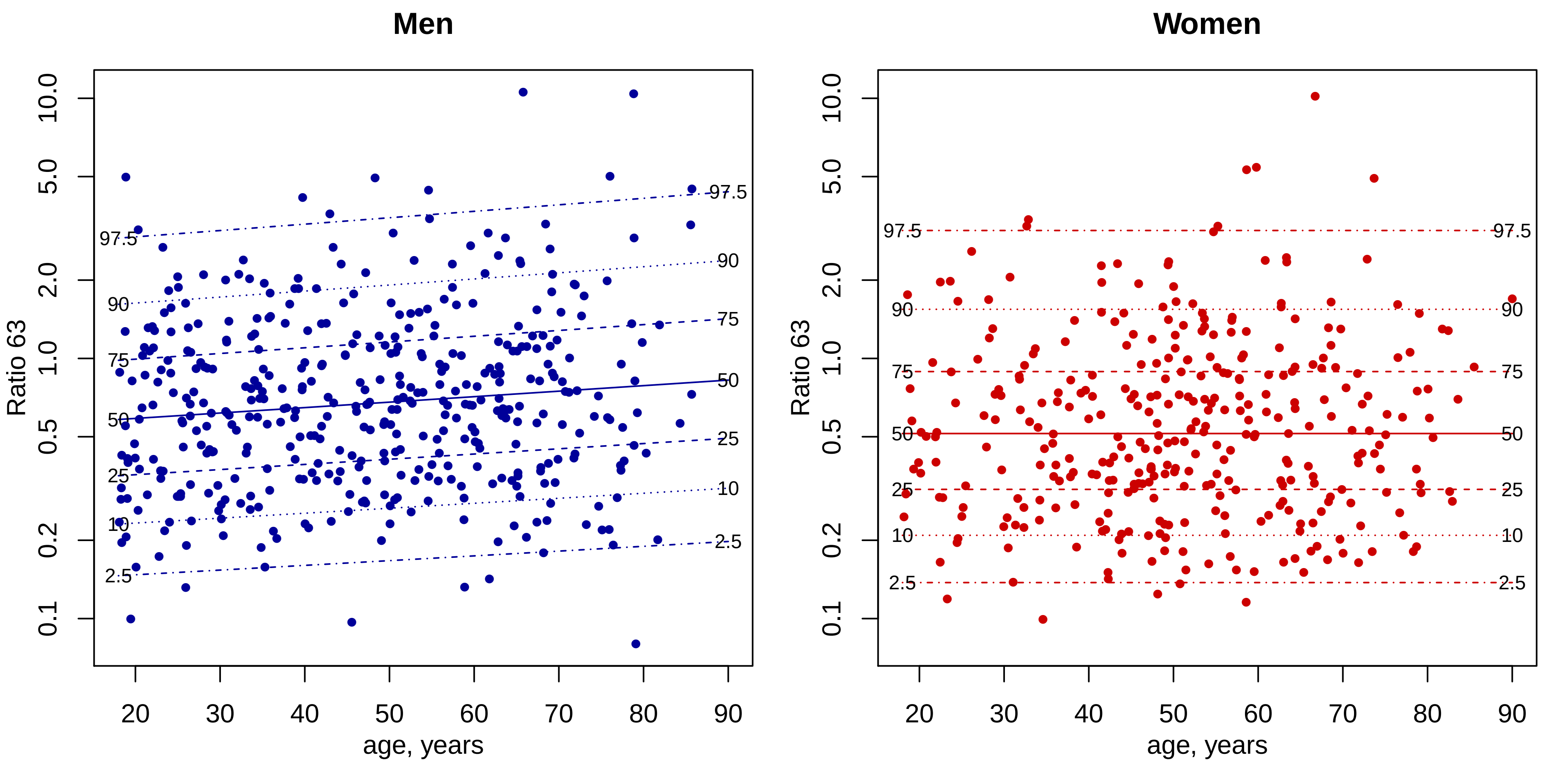

Men
Women
Ratio 63
age, years
10.0
5.0
2.0
1.0
0.5
0.2
0.1
20
30
40
50
60
70
80
90
97.5
90
75
50
25
10
2.5

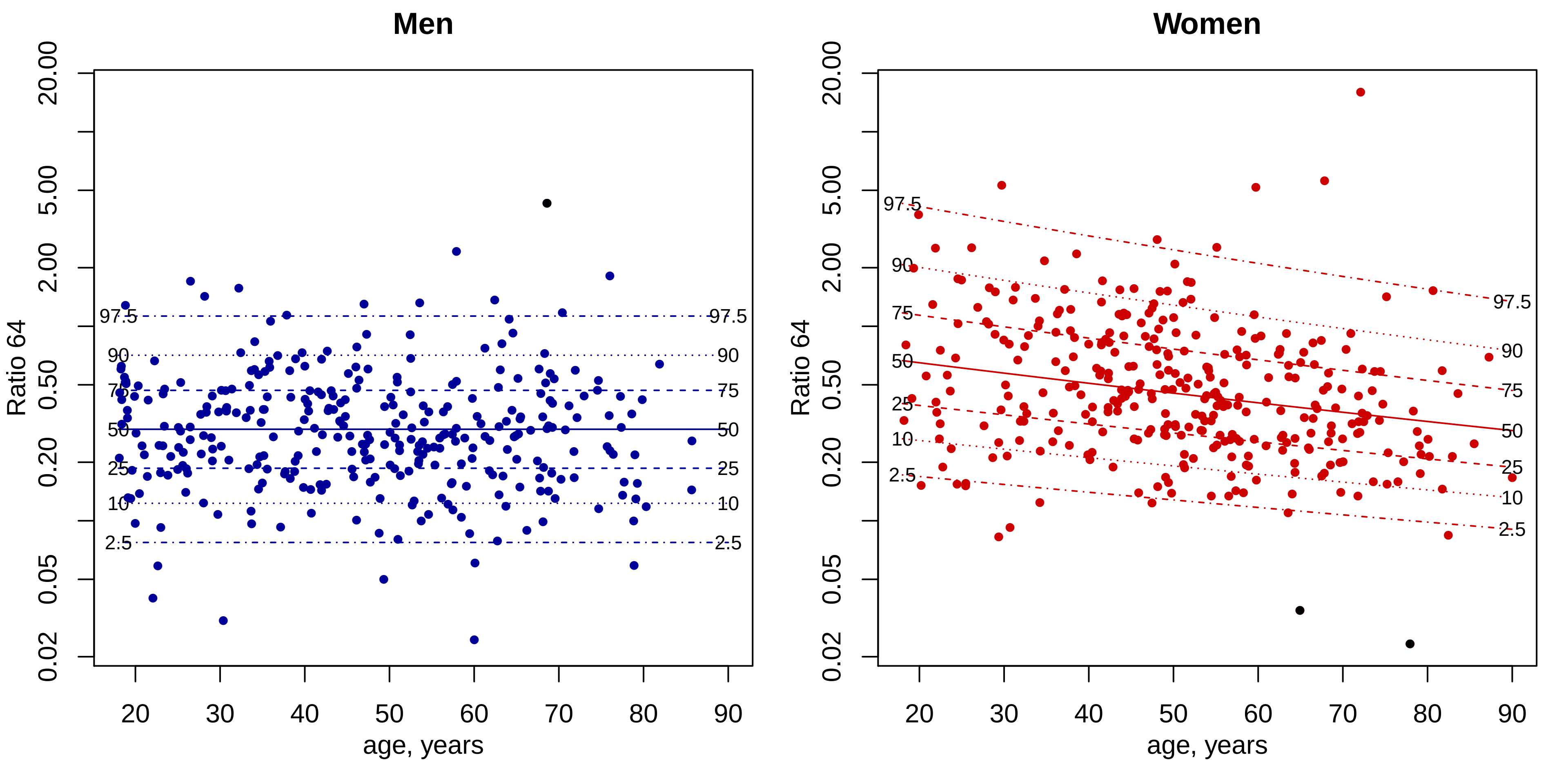
Men
Women
Ratio 64
20.00
5.00
2.00
0.50
0.20
0.05
0.02
97.5
90
75
50
25
10
2.5
20
30
40
50
60
70
80
90
age, years

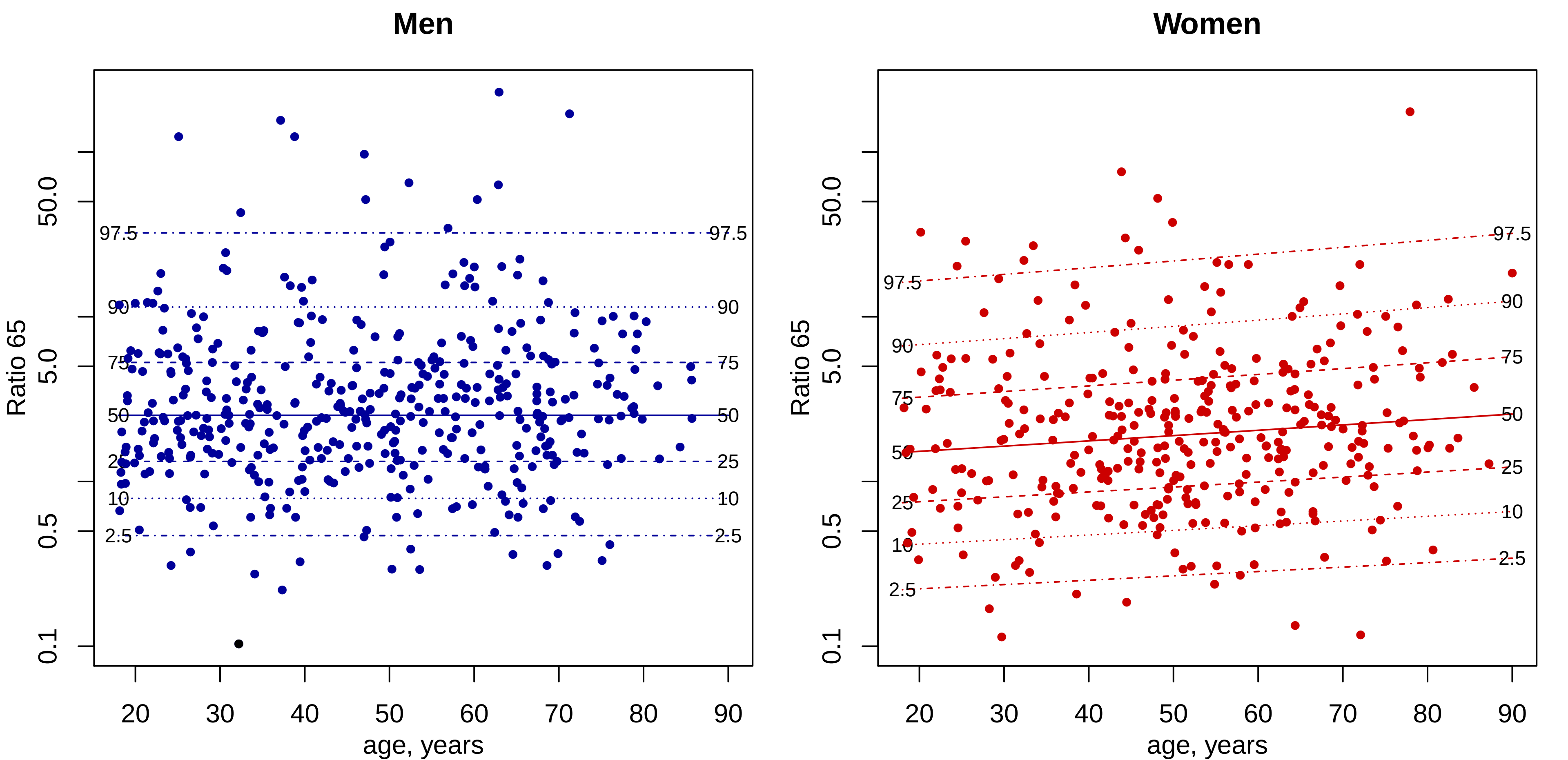

Men
Women
Ratio 65
0.1
0.5
5.0
50.0
97.5
90
75
50
25
10
2.5
20
30
40
50
60
70
80
90
age, years
