# Supplementary material for: Sex- and age-specific reference intervals for diagnostic ratios reflecting relative activity of steroidogenic enzymes and pathways in adults
Source: PLoS One. 2021 Jul 8;16(7):e0253975. doi: 10.1371/journal.pone.0253975 (PMC8266106; doi:10.1371/journal.pone.0253975)
Supplement: S1 Table — Underlying metabolites were mainly measured by gas chromatography–mass spectrometry (GC-MS). Ratios from healthy women (W) and men (M) have been considered since 1986. Ratios are dimensionless and have been described by descriptive statistics as indicated (type of data). The methods of analysis and the units in which metabolites have been measured in order to create ratios are also indicated. The population and subgroups ratios derives from are further described where available. Abbreviations: SD, standard deviation; SEM; standard error of the mean. (PDF) [file pone.0253975.s005.pdf]

Supporting Table 1. Published diagnostic ratios based on urinary steroid hormone metabolites. Underlying metabolites were mainly measured by gas chromatography–mass spectrometry (GC-MS). Ratios from healthy women (W) and men (M) have been considered since 1986. Abbreviations: SD, standard deviation; SEM; standard error of the mean

**First author, year, reference**		**Shackleton, 1986, [1]**		**Stewart, 1988, [2]**		**Weykamp, 1989, [3]**						**Farese, 1991, [4]**
**Sex**		W	M	W	M	W	W	W	M	M	M	M
**Age (years)**		22-50	22-50	18-55		17-50	51-70	> 70	17-50	51-70	> 70	
**Number of subjects**		13	17	6	16	24	24	24	24	24	24	17
**Type of data**		mean or range		mean±SD		2.5^{th}-97.5^{th} percentile						mean±SEM
**Measuring unit**		µg/24h	µg/24h	µg/24h		µmol/24h						
**Analysis method**		GC-MS		GC-MS		GC without MS						
**Population**		random sample corrected for mean daily creatinine excretion		normal controls		good health, no medication, female: neither pregnancy nor using oral contraceptives, at 24 institutions in The Netherland and Belgium (1 subject from each sex and age stratum per institution)						-
**Ratios**	**Ratio ID**											
PTO/(THE+THF+5αTHF)	2	0.02-0.014										
17HP/(THE+THF+5αTHF)	4	0.02-0.10										
PT/(THE+THF+5αTHF)	6	0.03-0.15										
5PT/(THE+THF+5αTHF)	10	0.08	0.08									
DHEA/(THE+THF+5αTHF)	12	0.10	0.35									
THS/(THE+THF+5αTHF)	17	0.01										
(THA+THB+5αTHB)/(AT+ET)	19	0.28	0.11									
(THA+THB+5αTHB)/(THE+THF+5αTHF)	21	0.33#1	0.19#1									
PD/PT	23	0.2#2	0.2									
(AT+ET)/(THE+THF+5αTHF)	26	0.66	0.86									
(AT+ET)/(THE+THF+5αTHF)	45	0.66	0.86									
ET/AT	47	1.73	1.24			0.7-1.9	0.8-2.6	0.6-3.5	0.4-1.8	0.4-1.7	0.5-1.7	
11βOHET/11βOHAT	48	0.51	0.35									
THF/5αTHF	49	2	1.56			0.7-3.2	1.0-3.9	0.9-3.7	0.9-2.4	1.1-2.6	0.8-2.8	1.40±0.70
THB/5αTHB	50	0.48	0.57									
(THF+5αTHF)/THE	53	0.70	1.03	0.97±0.30								
THE/(THF+5αTHF)	56	1.43	0.97			1.2-2.8#3	0.9-2.5#3	0.7-2.8#3	1.1-2.9#3	0.9-2.6#3	0.7-2.3#3	0.95±0.26
(αCl+βCl)/(αC+βC)	57											2.90±0.69
THALDO×100/(THE+THF+5αTHF)	64	4										
18OHTHA/THALDO	65	2.1										

#1 value from ratio without THA in the numerator and without THE in the denominator, #2 follicular phase level of PD, # 3value from ratio without 5αTHF in the denominator
Abbreviations of steroid hormone metabolites: Δ5diol, androstenediol; Δ5triol, androstenetriol, AT, androsterone; 11βOHAT, 11β-OH-androsterone; αC, α-cortol; βC, β-cortol; αCl, α-cortolone; βCl, β-cortolone; DHEA, dehydroepiandrosterone; 16OHDHEA, 16α-OH-dehydroepiandrosterone; E, cortisone; 20βDHE, 20β-DH-cortisone; F, cortisol; ET, etiocholanolone; 11βOHET, 11β-OH-etiocholanolone; 17HP, 17α-OH-pregnanolone; PD, pregnanediol; PT, pregnanetriol; 5PT, pregnenetriol; PTO, pregnanetriolone; THA, tetrahydro-11-dehydro-corticosterone; 18OHTHA, 18-OH-tetrahydro-11-dehydrocorticosterone; THB, tetrahydrocorticosterone; 5α-THB, 5α-tetrahydrocorticosterone; THE, tetrahydrocortisone; THF, tetrahydrocortisol; 5αTHF, 5α-tetrahydrocortisol; 18OHF, 18-OH-cortisol; THS, TH-11-deoxycortisol; THALDO, tetrahydroaldosterone

Supporting Table 1. (continued)

First author, year, reference		Soro, 1995, [5]		Finken, 1999, [6]				Shackleton, 2006, [7] and 2008, [8]
**Sex**		W	M	W			M	W and M
**Age (years)**		20-63		20-40			22-35	NA
**Number of subjects**		14	34	10 in menstrual phase (2-5 d after starting menstruation)	10 in follicular phase (19-16 d before the next expected menstruation)	10 in luteal phase (9-5 d before next menstruation)	10	26-34
**Type of data**		mean±SEM		Median [25th-75th percentile]				mean±SD/SEM or range
**Measuring unit**		µmol/24h		µg/24h				µg/24h
**Analysis method**		GC-MS		GC-MS				GC-MS
**Population**		control group for hypertensive subjects, normal blood pressure, no history of hypertension in first-degree relatives		white, 10 healthy men and 10 healthy women with regular endogenous menstrual cycles (between 24-34 days), all studies were completed during the months December to March				NA
**Ratios**	**Ratio ID**							
PTO/(THE+THF+5αTHF)	2							0.003
5PT/(THE+THF+5αTHF)	10							0.05
DHEA/(THE+THF+5αTHF)	12							0.065
5PT/PTO	15							26.2
(THA+THB+5αTHB)/(THE+THF+5αTHF)	21							0.14 [0.06-0.29]
(17HP+PT)/(THE+THF+5αTHF)	42							0.16
ET/AT	47			0.68 [0.55-0.98]#4	0.59 [0.47-0.85]#4	0.64 [0.56-0.77]#4	0.53 [0.35-0.68]#4	0.92±0.34
11βOHET/11βOHAT	48							0.48±0.54
THF/5αTHF	49	1.04±0.07		0.92 [0.69-1.30]	0.86 [0.70-1.00]	0.98 [0.79-1.07]	0.76 [0.66-1.16]	1.28±0.54 / 1.74 [0.35-2.76]
THB/5αTHB	50							0.51±0.19
F/E	52			1.27 [0.94-1.70]	1.16 [0.91-1.74]	0.92 [0.73-1.37]	0.94 [0.73-1.04]	0.46 [0.2-0.85]
(THF+5αTHF)/THE	53	0.97±0.04		1.90 [1.38-2.45]	1.78 [1.34-2.01]	2.06 [1.39-2.63]	1.99 [1.70-2.62]	1.0 [0.55-2.44] / [0.51-2.0]
THE/(THF+5αTHF)	56							1.0
(αCl+βCl)/(αC+βC)	57							2.6
F/18OHF	63							~4
THALDO×100/(THE+THF+5αTHF)	64							1-3

#4 value from ratio with epiandrosterone additionnally in the denominator

Abbreviations of steroid hormone metabolites: Δ5diol, androstenediol; Δ5triol, androstenetriol, AT, androsterone; 11βOHAT, 11β-OH-androsterone; αC, α-cortol; βC, β-cortol; αCl, α-cortolone; βCl, β-cortolone; DHEA, dehydroepiandrosterone; 16OHDHEA, 16α-OH-dehydroepiandrosterone; E, cortisone; 20βDHE, 20β-DH-cortisone; F, cortisol; ET, etiocholanolone; 11βOHET, 11β-OH-etiocholanolone; 17HP, 17α-OH-pregnanolone; PD, pregnanediol; PT, pregnanetriol; 5PT, pregnenetriol; PTO, pregnanetriolone; THA, tetrahydro-11-dehydro-corticosterone; 18OHTHA, 18-OH-tetrahydro-11-dehydrocorticosterone; THB, tetrahydrocorticosterone; 5α-THB, 5α-tetrahydrocorticosterone; THE, tetrahydrocortisone; THF, tetrahydrocortisol; 5αTHF, 5α-tetrahydrocortisol; 18OHF, 18-OH-cortisol; THS, TH-11-deoxycortisol; THALDO, tetrahydroaldosterone

Supporting Table 1. (continued)

First author, year, reference		Shackleton, 2008, [9]		Chan, 2008, [10]		de Jong, 2017, [11]											
**Sex**		W	M	W	M	W	W	W	W	W	W	M	M	M	M	M	M
**Age (years)**		NA		44.7 ±13.1 (mean±SD), range 20-85	41.6 ±12.9 (mean±SD), range 23-78	20-29	30-39	40-49	50-59	60-69	70-79	20-29	30-39	40-49	50-59	60-69	70-79
**Number of subjects**		26	24	89	81	20	20	20	20	20	20	20	20	20	20	20	20
**Type of data**		median and range		Median [2.5^{th}-97.5^{th} percentile] (the original publication also provides the 25th and 75th percentile)		2.5^{th}-97.5^{th}											
**Measuring unit**		µg/24h		µg/24h		µmol/24h											
**Analysis method**		GC-MS		GC-MS		GC-MS/MS											
**Population**		NA		Staff of the Department of Pathology, Queen Elizabeth Hospital, Hong Kong, their families, relatives and friends. Exclusion criteria included tobacco smoking, alcohol consumption (>14 units/week in female, >21 units/week in male), acute or chronic medical or mental illnesses, known malignancies, use of chronic medications, herbs or vitamin supplements, pregnant or lactatingwomen and miscarriage in the last 3 months.		healthy, no medication, body mass index 21-30 kg/m^2, selected from the LifeLines Cohort Study, a large population based cohort study in which inhabitants of the northern part of The Netherlands and their families were invited to participate.											
**Ratios**	**Ratio ID**																
PTO/(THE+THF+5αTHF)	2					0.0-0.02	0.0-0.01	0.0-0.01	0.0-0.005	0.0-0.02	0.0-0.01	0.0-0.004	0.0-0.004	0.0-0.004	0.0-0.007	0.0-0.01	0.0-0.01
PT/(THE+THF+5αTHF)	6					0.0-0.3	0.0-0.3	0.0-0.3	0.0-0.2	0.0-0.1	0.0-0.1	0.1-0.3	0.1-0.3	0.1-0.2	0.0-0.2	0.0-0.2	0.0-0.2
DHEA/(THE+THF+5αTHF)	12					0.0-0.7	0.0-0.5	0.0-0.4	0.0-0.1	0.0-0.04	0.0-0.03	0.0-0.8	0.0-0.6	0.0-0.4	0.0-0.2	0.0-0.1	0.0-0.2
THS/(THE+THF+5αTHF)	17					0.0-0.01	0.0-0.02	0.0-0.02	0.0-0.02	0.0-0.03	0.0-0.02	0.0-0.02	0.0-0.02	0.0-0.02	0.0-0.02	0.0-0.02	0.0-0.02
(THA+THB+5αTHB)/(AT+ET)	19					0.0-0.4	0.0-0.3	0.0-0.3	0.1-0.5	0.1-0.9	0.1-0.9	0.0-0.2	0.0-0.3	0.1-0.2	0.1-0.3	0.0-0.3	0.1-0.5
(THA+THB+5αTHB)/ (THE+THF+5αTHF)	21					0.1-0.2	0.0-0.2	0.0-0.2	0.0-0.1	0.0-0.1	0.0-0.1	0.0-0.1	0.0-0.2	0.0-0.2	0.0-0.1	0.0-0.1	0.0-0.1
(AT+ET)/(THE+THF+5αTHF)	26					0.1-2.2	0.2-1.7	0.3-1.7	0.2-0.9	0.1-0.8	0.0-0.5	0.5-1.9	0.3-1.4	0.4-1.2	0.2-1.1	0.2-0.9	0.2-1.0
PD/(THE+THF+5αTHF)	44					0.0-0.8	0.0-1.2	0.0-1.3	0.0-0.1	0.0-0.1	0.0-0.1	0.0-0.1	0.0-0.1	0.0-0.1	0.0-0.1	0.0-0.1	0.0-0.1
ET/AT	47					0.3-1.8	0.2-2.1	0.6-2.4	0.5-2.5	0.6-4.3	0.5-2.9	0.3-1.8	0.1-1.1	0.4-1.2	0.3-1.4	0.5-1.5	0.5-1.9
THF/5αTHF	49	1.48 [0.63-4.83]		0.91 [0.30-1.90]	1.41 [0.48-2.53]	0.5-4.1	0.5-3.7	0.7-4.1	0.7-4.2	0.7-7.4	0.7-6.1	0.3-2.8	0.4-1.8	0.4-1.5	0.5-1.8	0.6-2.7	0.7-3.1
THB/5αTHB	50					0.2-1.5	0.2-1.4	0.1-1.1	0.3-1.6	0.2-2.4	0.2-2.1	0.1-1.2	0.2-0.6	0.2-0.6	0.2-0.7	0.2-1.0	0.2-1.0
F/E	52	1.85 [1.02-7.69]															
(THF+5αTHF)/THE	53			0.83 [0.48-1.47]	1.03 [0.57-1.44]	0.4-1.2	0.4-1.6	0.5-1.2	0.5-1.1	0.5-1.3	0.5-1.3	0.5-2.4	0.7-1.6	0.7-1.8	0.6-1.7	0.5-1.7	0.7-1.8

Abbreviations of steroid hormone metabolites: Δ5diol, androstenediol; Δ5triol, androstenetriol, AT, androsterone; 11βOHAT, 11β-OH-androsterone; αC, α-cortol; βC, β-cortol; αCl, α-cortolone; βCl, β-cortolone; DHEA, dehydroepiandrosterone; 16OHDHEA, 16α-OH-dehydroepiandrosterone; E, cortisone; 20βDHE, 20β-DH-cortisone; F, cortisol; ET, etiocholanolone; 11βOHET, 11β-OH-etiocholanolone; 17HP, 17α-OH-pregnanolone; PD, pregnanediol; PT, pregnanetriol; 5PT, pregnenetriol; PTO, pregnanetriolone; THA, tetrahydro-11-dehydro-corticosterone; 18OHTHA, 18-OH-tetrahydro-11-dehydrocorticosterone; THB, tetrahydrocorticosterone; 5α-THB, 5α-tetrahydrocorticosterone; THE, tetrahydrocortisone; THF, tetrahydrocortisol; 5αTHF, 5α-tetrahydrocortisol; 18OHF, 18-OH-cortisol; THS, TH-11-deoxycortisol; THALDO, tetrahydroaldosterone

Supporting Table 1. (continued)

**First author, year, reference**		**Dhayat, 2018, [12]**	**Vulto, 2020, [13]**	
**Sex**		W	W	M
**Age (years)**		34; 28-42 (median; 25th-75th)	53±11 (mean±SD)	
**Number of subjects**		51-66	143	132
**Type of data**		median; 25th-75th	median and range	
**Measuring unit**		nmol/24h	µmol/24h	
**Analysis method**		GC-MS/MS	LC-MS/MS	
**Population**		healthy women, no medication intake, regular menstrual cycle, recruited from the general adult SWISS population	healthy subjects detected by a screening program before kidney donation, not treated with glucocorticoids, without history of kidney disease, diabetes or cardiovascular events, treated hypertension in some subjects	
**Ratios**	**Ratio ID**			
PTO/THE	1	0.005; 0.004-0.008		
PTO/(THE+THF+5αTHF)	2	0.003; 0.002-0.004		
17HP/THE	3	0.056; 0.036-0.091		
17HP/(THE+THF+5αTHF)	4	0.029; 0.02-0.042		
PT/THE	5	0.287; 0.211-0.387		
PT/(THE+THF+5αTHF)	6	0.155; 0.106-0.207		
(PTO+17HP+PT)/THE	7	0.355; 0.271-0.511		
(PTO+17HP+PT)/(THE+THF+5αTHF)	8	0.195; 0.136-0.239		
5PT/THE	9	0.06; 0.031-0.097		
5PT/(THE+THF+5αTHF)	10	0.027; 0.016-0.05		
DHEA/THE	11	0.052; 0.028-0.169		
DHEA/(THE+THF+5αTHF)	12	0.028; 0.015-0.096		
(DHEA+16OHDHEA)/THE	13	0.214; 0.092-0.436		
(DHEA+16OHDHEA)/(THE+THF+5αTHF)	14	0.12; 0.05-0.262		
THS/THE	16	0.023; 0.018-0.031		
THS/(THE+THF+5αTHF)	17	0.012; 0.009-0.017		
PD/(AT+ET)	18	0.147; 0.073-0.384		
(THA+THB+5αTHB)/(AT+ET)	19	0.15; 0.096-0.213		
(THA+THB+5αTHB)/THE	20	0.221; 0.176-0.279		
(THA+THB+5αTHB)/(THE+THF+5αTHF)	21	0.12; 0.086-0.138		
PD/17HP	22	4.77; 2.88-7.84		
PD/PT	23	0.807; 0.512-1.59		
PD/(PT+17HP)	24	0.681; 0.439-1.43		
(AT+ET)/THE	25	1.6; 1.1-2.17		
(AT+ET)/(THE+THF+5αTHF)	26	0.834; 0.624-1.24		
5PT/(DHEA+16OHDHEA)	27	0.23; 0.146-0.57		
5PT/Δ^5diol	28	1.36; 0.789-2.32		
17HP/11βOHAT	31	0.192; 0.119-0.357		
PT/11βOHAT	32	1.07; 0.693-1.49		
(17HP+PT)/11βOHAT	33	1.26; 0.811-1.83		
17HP/(AT+ET)	34	0.03; 0.02-0.066		
PT/(AT+ET)	35	0.18; 0.123-0.251		
(17HP+PT)/(AT+ET)	36	0.214; 0.14-0.337		
11βOHAT/(DHEA+16OHDHEA)	37	1.37; 0.696-2.47		
11βOHAT/Δ^5diol	38	7.03; 4.43-10.3		
(17HP+PT)/THE	41	0.345; 0.264-0.501		
(17HP+PT)/(THE+THF+5αTHF)	42	0.19; 0.132-0.236		
PD/THE	43	0.198; 0.127-0.479		
PD/(THE+THF+5αTHF)	44	0.107; 0.064-0.276		
(AT+ET)/(THE+THF+5αTHF)	45	0.834; 0.624-1.24		
AT/ET	46	0.918; 0.733-1.112		
ET/AT	47	1.09; 0.899-1.36		
11βOHET/11βOHAT	48	0.576; 0.361-0.886		
THF/5αTHF	49	1.54; 1.06-2.04		
THB/5αTHB	50	0.534; 0.383-0.68		
testosterone/17β-estradiol	51	2.8; 1.64-7.56		
F/E	52	0.618; 0.452-0.811	0.63 [0.54-0.74]	
(THF+5αTHF)/THE	53	0.929; 0.71-1.06	0.94 [0.79-1.0]	
(αC+βC)/(αCl+βCl)	54	0.372; 0.319-0.467		
(F+E)/(THF+5αTHF+THE)	55	0.061; 0.046-0.081		
THE/(THF+5αTHF)	56	1.08; 0.946-1.41		
(αCl+βCl)/(αC+βC)	57	2.69; 2.14-3.14		
(THF+5αTHF+THE)/(αC+αCl)	58	3.46; 2.90-4.07		
(THF+5αTHF+THE)/βC+βCl	59	5.43; 4.63-6.81		
(αC+αCl)/(βC+βCl)	60	1.64; 1.37-2.07		
20αDHF/(THF+5αTHF)	61	0.025; 0.017-0.04		
(androstanediol$^{1.5}$×20βDHE)/(20βDHE+[F×log(estriol)]	62	<435.9		

Abbreviations of steroid hormone metabolites: Δ5diol, androstenediol; Δ5triol, androstenetriol, AT, androsterone; 11βOHAT, 11β-OH-androsterone; αC, α-cortol; βC, β-cortol; αCl, α-cortolone; βCl, β-cortolone; DHEA, dehydroepiandrosterone; 16OHDHEA, 16α-OH-dehydroepiandrosterone; E, cortisone; 20βDHE, 20β-DH-cortisone; F, cortisol; ET, etiocholanolone; 11βOHET, 11β-OH-etiocholanolone; 17HP, 17α-OH-pregnanolone; PD, pregnanediol; PT, pregnanetriol; 5PT, pregnenetriol; PTO, pregnanetriolone; THA, tetrahydro-11-dehydro-corticosterone; 18OHTHA, 18-OH-tetrahydro-11-dehydrocorticosterone; THB, tetrahydrocorticosterone; 5α-THB, 5α-tetrahydrocorticosterone; THE, tetrahydrocortisone; THF, tetrahydrocortisol; 5αTHF, 5α-tetrahydrocortisol; 18OHF, 18-OH-cortisol; THS, TH-11-deoxycortisol; THALDO, tetrahydroaldosterone

References

1. Shackleton CH. Profiling steroid hormones and urinary steroids. Journal of chromatography. 1986;379:91-156. Epub 1986/06/20. PubMed PMID: 3525596.
2. Stewart PM, Corrie JE, Shackleton CH, Edwards CR. Syndrome of apparent mineralocorticoid excess. A defect in the cortisol-cortisone shuttle. J Clin Invest. 1988;82(1):340-9. doi: 10.1172/JCI113592. PubMed PMID: 3164727.
3. Weykamp CW, Penders TJ, Schmidt NA, Borburgh AJ, van de Calseyde JF, Wolthers BJ. Steroid profile for urine: reference values. Clinical chemistry. 1989;35(12):2281-4. Epub 1989/12/01. PubMed PMID: 2591044.
4. Farese RV, Biglieri EG, Shackleton CHL, Irony I, Gomez-Fontes R. Licorice-Induced Hypermineralocorticoidism. New England Journal of Medicine. 1991;325(17):1223-7. doi: 10.1056/nejm199110243251706. PubMed PMID: 1922210.
5. Soro A, Ingram MC, Tonolo G, Glorioso N, Fraser R. Evidence of coexisting changes in 11 beta-hydroxysteroid dehydrogenase and 5 beta-reductase activity in subjects with untreated essential hypertension. Hypertension. 1995;25(1):67-70. doi: 10.1161/01.hyp.25.1.67. PubMed PMID: 7843756.
6. Finken MJ, Andrews RC, Andrew R, Walker BR. Cortisol metabolism in healthy young adults: sexual dimorphism in activities of A-ring reductases, but not 11beta-hydroxysteroid dehydrogenases. The Journal of clinical endocrinology and metabolism. 1999;84(9):3316-21. Epub 1999/09/16. PubMed PMID: 10487705.
7. Shackleton CHJM. Hyphenated Methods. The Encyclopedia of Mass Spectrometry. 82006.
8. Shackleton C. Genetic Disorders of Steroid Metabolism Diagnosed by Mass Spectrometry. In: Blau N, Duran M, Gibson KM, editors. Laboratory Guide to the Methods in Biochemical Genetics: Springer Berlin Heidelberg; 2008. p. 549-605.
9. Shackleton CH, Neres MS, Hughes BA, Stewart PM, Kater CE. 17-Hydroxylase/C17,20-lyase (CYP17) is not the enzyme responsible for side-chain cleavage of cortisol and its metabolites. Steroids. 2008;73(6):652-6. Epub 2008/03/22. doi: 10.1016/j.steroids.2008.02.001. PubMed PMID: 18355883.
10. Chan AO, Taylor NF, Tiu SC, Shek CC. Reference intervals of urinary steroid metabolites using gas chromatography-mass spectrometry in Chinese adults. Steroids. 2008;73(8):828-37. doi: 10.1016/j.steroids.2008.03.004. PubMed PMID: 18452960.
11. de Jong WHA, Buitenwerf E, Pranger AT, Riphagen IJ, Wolffenbuttel BHR, Kerstens MN, et al. Determination of reference intervals for urinary steroid profiling using a newly validated GC-MS/MS method. Clinical chemistry and laboratory medicine. 2017;56(1):103-12. doi: 10.1515/cclm-2016-1072. PubMed PMID: 28598795.
12. Dhayat NA, Marti N, Kollmann Z, Troendle A, Bally L, Escher G, et al. Urinary steroid profiling in women hints at a diagnostic signature of the polycystic ovary syndrome: A pilot study considering neglected steroid metabolites. PloS one. 2018;13(10):e0203903. doi: 10.1371/journal.pone.0203903. PubMed PMID: 30308019; PubMed Central PMCID: PMC6181287.
13. Vulto A, Minovic I, de Vries LV, Timmermans AC, van Faassen M, Gomes Neto AW, et al. Endogenous urinary glucocorticoid metabolites and mortality in prednisolone-treated renal transplant recipients. Clinical transplantation. 2020;34(4):e13824. doi: 10.1111/ctr.13824. PubMed PMID: 32052523; PubMed Central PMCID: PMC7216873.
