# Supplementary material for: Sex- and age-specific reference intervals for diagnostic ratios reflecting relative activity of steroidogenic enzymes and pathways in adults
Source: PLoS One. 2021 Jul 8;16(7):e0253975. doi: 10.1371/journal.pone.0253975 (PMC8266106; doi:10.1371/journal.pone.0253975)
Supplement: S2 Table — The available number of participants is indicated for each diagnostic ratio. Diagnostic ratios are stratified for sex and are described by their median;25th-75th percentile. Between-group differences were determined by Mann–Whitney U test, and the corresponding p values are indicated. (PDF) [file pone.0253975.s006.pdf]

Supporting Table 2. Sex specific differences in diagnostic ratios based on steroid hormone metabolites measured in 24-hour urine.

Enzymes, pathways and disorders	Ratio	Ratio ID	Women		Men		
			N	Median;25th-75th	N	Median;25th-75th	*p*
**21-hydroxylase deficiency (21OHD)**	PTO/THE	1	360	0.00623;0.00433-0.00968	407	0.00533;0.00388-0.00822	0.002
	PTO/(THE+THF+5αTHF)	2	329	0.00316;0.00227-0.00534	321	0.00252;0.00192-0.00407	<0.001
	17HP/THE	3	356	0.0301;0.0166-0.0637	402	0.0571;0.0400-0.0854	<0.001
	17HP/(THE+THF+5αTHF)	4	328	0.0160;0.00903-0.0337	320	0.0290;0.0201-0.0420	<0.001
	PT/THE	5	348	0.180;0.116-0.302	362	0.232;0.162-0.324	<0.001
	PT/(THE+THF+5αTHF)	6	323	0.0992;0.0628-0.161	303	0.114;0.0814-0.161	0.005
	(PTO+17HP+PT)/THE	7	344	0.221;0.141-0.379	360	0.307;0.217-0.417	<0.001
	(PTO+17HP+PT)/(THE+THF+5αTHF)	8	322	0.121;0.0782-0.203	303	0.150;0.109-0.211	<0.001
**3β-hydroxysteroid dehydrogenase deficiency (3βHSDD)**	5PT/THE	9	359	0.0376;0.0162-0.0729	402	0.0658;0.0359-0.121	<0.001
	5PT/(THE+THF+5αTHF)	10	328	0.0193;0.00754-0.0370	321	0.0332;0.0160-0.0597	<0.001
	DHEA/THE	11	358	0.0342;0.0171-0.0880	395	0.0590;0.0245-0.233	<0.001
	DHEA/(THE+THF+5αTHF)	12	329	0.0186;0.00898-0.0462	315	0.0311;0.0114-0.119	<0.001
	(DHEA+16OHDHEA)/THE	13	358	0.0996;0.0513-0.219	389	0.158;0.0627-0.408	<0.001
	(DHEA+16OHDHEA)/(THE+THF+5αTHF)	14	329	0.0542;0.0274-0.119	312	0.0768;0.0298-0.237	<0.001
**Ratio to distinguish 3β-HSDD from 21OHD**	5PT/PTO	15	378	6.02;1.88-12.7	448	11.2;5.07-24.0	<0.001
**11β-hydroxylase deficiency (11βOHD)**	THS/THE	16	360	0.0252;0.0202-0.0331	407	0.0224;0.0172-0.0289	<0.001
	THS/(THE+THF+5αTHF)	17	329	0.0134;0.0106-0.0175	321	0.0109;0.00850-0.0142	<0.001
**CYP17A1 global deficiency (CYP17A1GD)[a]**	PD/(AT+ET)	18	327	0.137;0.0833-0.233	361	0.0547;0.0414-0.0758	<0.001
	(THA+THB+5αTHB)/(AT+ET)	19	330	0.193;0.123-0.326	359	0.142;0.102-0.200	<0.001
**17α-hydroxylase global deficiency (17αOHGD)**	(THA+THB+5αTHB)/THE	20	360	0.185;0.144-0.249	404	0.196;0.154-0.246	0.15
	(THA+THB+5αTHB)/(THE+THF+5αTHF)	21	329	0.0954;0.0771-0.124	320	0.0921;0.0768-0.114	0.10
**17α-hydroxylase Δ^4-pathway deficiency (17αOHΔ4D)**	PD/17HP	22	372	3.96;2.85-6.65	451	1.26;0.892-1.82	<0.001
	PD/PT	23	357	0.681;0.479-1.04	395	0.324;0.247-0.410	<0.001
	PD/(PT+17HP)	24	353	0.566;0.415-0.844	392	0.252;0.195-0.324	<0.001
**17,20-lyase global deficency (17,20LGD)**	(AT+ET)/THE	25	320	0.951;0.516-1.60	340	1.31;0.947-1.95	<0.001
	(AT+ET)/(THE+THF+5αTHF)	26[b]	297	0.517;0.281-0.838	280	0.651;0.455-1.03	<0.001
**17,20-lyase Δ^5-pathway deficency (17,20LΔ5D)**	5PT/(DHEA+16OHDHEA)	27	376	0.318;0.147-0.640	427	0.326;0.187-0.694	0.14
	5PT/Δ^5diol	28	377	1.27;0.727-2.10	446	1.34;0.893-2.00	0.26
	5PT/Δ^5triol	29	377	0.417;0.184-0.738	447	0.472;0.272-0.789	0.0080
	5PT/(DHEA+16OHDHEA+Δ^5diol+Δ^5triol)	30	375	0.153;0.0749-0.244	426	0.161;0.0988-0.235	0.24
**17,20-lyase Δ^4-pathway deficency (17,20LΔ4D)**	17HP/11βOHAT	31	372	0.106;0.0639-0.214	443	0.187;0.126-0.277	<0.001
	PT/11βOHAT	32	357	0.671;0.429-1.07	391	0.736;0.521-1.03	0.051
	(17HP+PT)/11βOHAT	33	353	0.799;0.491-1.26	389	0.932;0.655-1.31	<0.001
	17HP/(AT+ET)	34	328	0.0344;0.0209-0.0610	358	0.0443;0.0308-0.0641	<0.001
	PT/(AT+ET)	35	324	0.207;0.146-0.283	336	0.173;0.138-0.223	<0.001
	(17HP+PT)/(AT+ET)	36	322	0.243;0.170-0.348	334	0.220;0.173-0.284	0.021

The available number of participants is indicated for each diagnostic ratio. Diagnostic ratios are stratified for sex and are described by their median;25th-75th percentile. Between-group differences were determined by Mann–Whitney U test, and the corresponding *p* values are indicated.

[a]combined activity of 17α-hydroxylase and 17,20-lyase, [b]ratio 26 also used to assess 3βHSDD, [c]/apparent mineralocorticoid excess, [d]/cortisone reductase deficiency, [e]/familial hyperaldosteronism type I.

Abbreviations: Δ^5diol, androstenediol; Δ^5triol, androstenetriol, AT, androsterone; 11βOHAT, 11β-OH-androsterone; αC, α-cortol; βC, β-cortol; αCl, α-cortolone; βCl, β-cortolone; DHEA, dehydroepiandrosterone; 16OHDHEA, 16α-OH-dehydroepiandrosterone; E, cortisone; 20βDHE, 20β-DH-cortisone; F, cortisol; ET, etiocholanolone; 11βOHET, 11β-OH-etiocholanolone; 17HP, 17α-OH-pregnanolone; PD, pregnanediol; PT, pregnanetriol; 5PT, pregnenetriol; PTO, pregnanetriolone; THA, tetrahydro-11-dehydro-corticosterone; 18OHTHA, 18-OH-tetrahydro-11-dehydrocorticosterone; THB, tetrahydrocorticosterone; 5α-THB, 5α-tetrahydrocorticosterone; THE, tetrahydrocortisone; THF, tetrahydrocortisol; 5αTHF, 5α-tetrahydrocortisol; 18OHF, 18-OH-cortisol; THS, TH-11-deoxycortisol; THALDO, tetrahydroaldosterone

Supporting Table 2. (continued)

Enzymes, pathways and disorders	Ratio	Ratio ID	Women		Men		
			N	Median;25th-75th	N	Median;25th-75th	*p*
**CYP17A1 global Δ^4- vs. Δ^5-pathway (CYP17A1Δ4vs.Δ5)**	11βOHAT/(DHEA+16OHDHEA)	37	374	2.75;1.29-5.88	424	2.05;0.700-4.72	<0.001
	11βOHAT/Δ^5diol	38	375	10.0;5.65-18.7	445	6.78;3.03-13.1	<0.001
	11βOHAT/(DHEA+16OHDHEA+Δ^5diol)	39	374	2.11;1.06-4.33	424	1.56;0.566-3.45	<0.001
	11βOHAT/(DHEA+16OHDHEA+Δ^5diol+Δ^5triol)	40	373	1.15;0.638-2.15	423	0.810;0.412-1.58	<0.001
**P450 oxidoreductase deficency (PORD)**	(17HP+PT)/THE	41	344	0.211;0.133-0.362	360	0.298;0.208-0.408	<0.001
	(17HP+PT)/(THE+THF+5αTHF)	42	322	0.115;0.0725-0.198	303	0.145;0.106-0.206	<0.001
	PD/THE	43	358	0.120;0.0657-0.256	407	0.0750;0.0499-0.118	<0.001
	PD/(THE+THF+5αTHF)	44	327	0.0647;0.0357-0.131	321	0.0383;0.0255-0.0601	<0.001
**17β-hydroxysteroid dehydrogenase (17βHSD)**	(AT+ET)/(THE+THF+5αTHF)	45	297	0.517;0.281-0.838	280	0.651;0.455-1.03	<0.001
**Alternative androgen backdoor pathway vs. classic pathway (ABPvs.CP)**	AT/ET	46	330	0.765;0.563-1.01	361	1.23;0.913-1.55	<0.001
**5α-reductase deficiency (5αRD)**	ET/AT	47	330	1.31;0.990-1.78	361	0.812;0.645-1.10	<0.001
	11βOHET/11βOHAT	48	375	0.642;0.389-0.934	447	0.434;0.245-0.643	<0.001
	THF/5αTHF	49	339	1.92;1.37-2.71	351	1.19;0.897-1.64	<0.001
	THB/5αTHB	50	379	0.611;0.457-0.844	457	0.414;0.326-0.585	<0.001
**CYP19A1 / Aromatase deficiency (CYP19A1D)**	testosterone/17β-estradiol	51	366	3.48;1.88-7.80	451	17.9;11.2-29.8	<0.001
**11β-hydroxysteroid dehydrogenase type 2 deficiency (11βHSD2D)[c]**	F/E	52	379	0.642;0.457-0.848	456	0.656;0.513-0.839	0.049
	(THF+5αTHF)/THE	53	329	0.904;0.744-1.06	321	1.04;0.893-1.26	<0.001
	(αC+βC)/(αCl+βCl)	54	353	0.390;0.327-0.471	398	0.429;0.361-0.512	<0.001
	(F+E)/(THF+5αTHF+THE)	55	329	0.840;0.795-0.878	321	0.773;0.714-0.817	<0.001
**11β-hydroxysteroid dehydrogenase type 1 deficiency (11βHSD2D)[d]**	THE/(THF+5αTHF)	56	329	1.11;0.941-1.34	321	0.958;0.792-1.12	<0.001
	(αCl+βCl)/(αC+βC)	57	353	2.57;2.12-3.05	398	2.33;1.95-2.77	<0.001
**20α-hydroxysteroid dehydrogenase (20αHSD)**	(THF+5αTHF+THE)/(αC+αCl)	58	326	1.71;1.38-2.00	328	2.15;1.82-2.57	<0.001
**20β-hydroxysteroid dehydrogenase (20βHSD)**	(THF+5αTHF+THE)/βC+βCl	59	337	2.79;2.18-3.56	345	3.01;2.35-3.78	0.012
**20αHSD vs. 20βHSD**	(αC+αCl)/(βC+βCl)	60	353	1.69;1.35-2.07	398	1.40;1.14-1.72	<0.001
**3α-hydroxysteroid dehydrogenase (3αHSD)**	20αDHF/(THF+5αTHF)	61	339	0.0239;0.0165-0.0347	351	0.0158;0.0112-0.0232	<0.001
**Polycystic ovary syndrome (PCOS)**	(androstanediol$^{1.5}$×20βDHE)/(20βDHE+[F×log(estriol)]	62	367	122;57.5-255	-	-	-
**Glucocorticoid-remediable aldosteronism (GRA)[e]**	F/18OHF	63	344	0.512;0.305-0.918	424	0.660;0.382-1.11	<0.001
**Hyperaldosteronismus, Pseudohypoaldosteronismus**	THALDO×100/(THE+THF+5αTHF)	64	329	0.436;0.264-0.786	320	0.279;0.183-0.438	<0.001
**Hypoaldosteronism**	18OHTHA/THALDO	65	341	1.87;0.968-4.05	432	2.59;1.48-5.21	<0.001

The available number of participants is indicated for each diagnostic ratio. Diagnostic ratios are stratified for sex and are described by their median;25th-75th percentile. Between-group differences were determined by Mann–Whitney U test, and the corresponding *p* values are indicated.

[a]combined activity of 17α-hydroxylase and 17,20-lyase, [b]ratio 26 also used to assess 3βHSDD, [c]/apparent mineralocorticoid excess, [d]/cortisone reductase deficiency, [e]/familial hyperaldosteronism type I.

Abbreviations: Δ^5diol, androstenediol; Δ^5triol, androstenetriol, AT, androsterone; 11βOHAT, 11β-OH-androsterone; αC, α-cortol; βC, β-cortol; αCl, α-cortolone; βCl, β-cortolone; DHEA, dehydroepiandrosterone; 16OHDHEA, 16α-OH-dehydroepiandrosterone; E, cortisone; 20βDHE, 20β-DH-cortisone; F, cortisol; ET, etiocholanolone; 11βOHET, 11β-OH-etiocholanolone; 17HP, 17α-OH-pregnanolone; PD, pregnanediol; PT, pregnanetriol; 5PT, pregnenetriol; PTO, pregnanetriolone; THA, tetrahydro-11-dehydro-corticosterone; 18OHTHA, 18-OH-tetrahydro-11-dehydrocorticosterone; THB, tetrahydrocorticosterone; 5α-THB, 5α-tetrahydrocorticosterone; THE, tetrahydrocortisone; THF, tetrahydrocortisol; 5αTHF, 5α-tetrahydrocortisol; 18OHF, 18-OH-cortisol; THS, TH-11-deoxycortisol; THALDO, tetrahydroaldosterone
