# Supplementary material for: Sex- and age-specific reference intervals for diagnostic ratios reflecting relative activity of steroidogenic enzymes and pathways in adults
Source: PLoS One. 2021 Jul 8;16(7):e0253975. doi: 10.1371/journal.pone.0253975 (PMC8266106; doi:10.1371/journal.pone.0253975)
Supplement: S3 Table — The available number of participants is indicated for each diagnostic ratio stratified for sex. Diagnostic ratios are described by their median;25th-75th percentile. Within-sex differences were determined by Wilcoxon signed-rank test, and the corresponding p values are indicated. (PDF) [file pone.0253975.s007.pdf]

Supporting Table 3. Day- and night-time specific differences for diagnostic ratios based on steroid hormone metabolites measured in day- and night-time urine.

		Women					Men				
		Day		Night			Day		Night		
Ratio	Ratio ID	N	Median;25th-75th	N	Median;25th-75th	*p*	N	Median;25th-75th	N	Median;25th-75th	*p*
PTO/THE	1	368	0.00624;0.00434-0.00962	368	0.00634;0.00433-0.0102	<0.001	425	0.00524;0.00376-0.00790	435	0.00557;0.00386-0.00899	<0.001
PTO/(THE+THF+5αTHF)	2	344	0.00319;0.00231-0.00514	350	0.00329;0.00228-0.00582	<0.001	348	0.00246;0.00184-0.00382	388	0.00277;0.00193-0.00445	<0.001
17HP/THE	3	368	0.0291;0.0161-0.0599	364	0.0349;0.0186-0.0755	<0.001	424	0.0519;0.0372-0.0778	431	0.0763;0.0515-0.120	<0.001
17HP/(THE+THF+5αTHF)	4	344	0.0147;0.00847-0.0327	349	0.0189;0.00959-0.0403	<0.001	348	0.0262;0.0179-0.0376	387	0.0382;0.0249-0.0591	<0.001
PT/THE	5	362	0.166;0.106-0.275	358	0.236;0.144-0.408	<0.001	399	0.212;0.147-0.286	400	0.307;0.214-0.463	<0.001
PT/(THE+THF+5αTHF)	6	343	0.0900;0.0559-0.147	342	0.126;0.0777-0.220	<0.001	341	0.104;0.0753-0.137	365	0.153;0.106-0.229	<0.001
(PTO+17HP+PT)/THE	7	362	0.207;0.130-0.347	354	0.278;0.172-0.511	<0.001	399	0.280;0.194-0.379	398	0.397;0.272-0.586	<0.001
(PTO+17HP+PT)/(THE+THF+5αTHF)	8	343	0.110;0.0681-0.187	341	0.152;0.0941-0.265	<0.001	341	0.134;0.0986-0.186	365	0.197;0.140-0.291	<0.001
5PT/THE	9	368	0.0352;0.0143-0.0684	367	0.0444;0.0186-0.0827	<0.001	423	0.0616;0.0343-0.117	431	0.0830;0.0420-0.148	<0.001
5PT/(THE+THF+5αTHF)	10	344	0.0178;0.00667-0.0352	349	0.0228;0.00912-0.0434	<0.001	348	0.0305;0.0152-0.0563	387	0.0390;0.0197-0.0737	<0.001
DHEA/THE	11	367	0.0319;0.0158-0.0825	367	0.0380;0.0179-0.0987	<0.001	414	0.0534;0.0226-0.247	426	0.0651;0.0251-0.232	<0.001
DHEA/(THE+THF+5αTHF)	12	343	0.0169;0.00829-0.0456	350	0.0197;0.00972-0.0520	<0.001	342	0.0272;0.0106-0.124	382	0.0335;0.0119-0.114	<0.001
(DHEA+16OHDHEA)/THE	13	367	0.0914;0.0450-0.208	367	0.116;0.0533-0.261	<0.001	409	0.148;0.0584-0.398	421	0.180;0.0695-0.466	<0.001
(DHEA+16OHDHEA)/(THE+THF+5αTHF)	14	343	0.0484;0.0225-0.108	350	0.0629;0.0275-0.143	<0.001	340	0.0747;0.0281-0.232	379	0.0866;0.0338-0.228	<0.001
5PT/PTO	15	379	5.66;1.66-11.9	378	6.66;2.13-14.5	<0.001	455	10.5;5.05-23.5	452	12.7;5.44-28.9	<0.001
THS/THE	16	368	0.0244;0.0191-0.0319	368	0.0285;0.0226-0.0381	<0.001	425	0.0210;0.0164-0.0279	435	0.0245;0.0194-0.0331	<0.001
THS/(THE+THF+5αTHF)	17	344	0.0127;0.0102-0.0165	350	0.0155;0.0120-0.0204	<0.001	348	0.0101;0.00814-0.0131	388	0.0122;0.00962-0.0163	<0.001
PD/(AT+ET)	18	357	0.137;0.0821-0.243	342	0.135;0.0841-0.236	0.96	387	0.0557;0.0416-0.0746	390	0.0552;0.0389-0.0750	0.19
(THA+THB+5αTHB)/(AT+ET)	19	357	0.205;0.132-0.345	345	0.160;0.102-0.283	<0.001	384	0.156;0.109-0.219	389	0.109;0.0739-0.163	<0.001
(THA+THB+5αTHB)/THE	20	368	0.178;0.139-0.242	368	0.200;0.149-0.259	<0.001	421	0.195;0.152-0.247	434	0.204;0.161-0.256	<0.001
(THA+THB+5αTHB)/(THE+THF+5αTHF)	21	344	0.0924;0.0745-0.121	350	0.103;0.0809-0.135	<0.001	346	0.0912;0.0754-0.112	388	0.0981;0.0790-0.121	<0.001
PD/17HP	22	379	3.91;2.65-6.52	372	4.58;2.97-7.56	<0.001	457	1.25;0.870-1.80	454	1.25;0.885-1.88	0.22
PD/PT	23	370	0.683;0.487-1.08	362	0.653;0.459-1.03	0.014	424	0.321;0.241-0.412	411	0.313;0.236-0.409	<0.001
PD/(PT+17HP)	24	370	0.565;0.410-0.883	358	0.545;0.400-0.881	0.72	424	0.253;0.189-0.322	408	0.247;0.188-0.320	0.0063
(AT+ET)/THE	25	350	0.884;0.469-1.43	340	1.20;0.657-2.16	<0.001	371	1.21;0.863-1.74	380	1.83;1.22-2.74	<0.001
(AT+ET)/(THE+THF+5αTHF)	26	330	0.486;0.250-0.754	326	0.674;0.361-1.14	<0.001	313	0.607;0.416-0.883	352	0.887;0.587-1.38	<0.001
5PT/(DHEA+16OHDHEA)	27	378	0.313;0.143-0.672	377	0.312;0.141-0.640	0.23	439	0.315;0.177-0.665	436	0.340;0.188-0.714	<0.001
5PT/Δ5diol	28	379	1.28;0.713-2.19	377	1.18;0.632-2.02	<0.001	455	1.34;0.910-2.04	449	1.27;0.815-1.97	<0.001
5PT/Δ5triol	29	379	0.430;0.198-0.785	377	0.369;0.145-0.649	<0.001	455	0.489;0.273-0.856	451	0.424;0.240-0.697	<0.001
5PT/(DHEA+16OHDHEA+Δ5diol+Δ5triol)	30	378	0.156;0.0741-0.261	376	0.137;0.0674-0.242	<0.001	439	0.163;0.0971-0.239	435	0.154;0.0947-0.232	0.073
17HP/11βOHAT	31	376	0.104;0.0637-0.215	374	0.109;0.0668-0.246	<0.001	452	0.173;0.116-0.253	449	0.233;0.153-0.354	<0.001
PT/11βOHAT	32	367	0.624;0.401-0.992	364	0.795;0.506-1.33	<0.001	421	0.680;0.476-0.974	406	0.960;0.646-1.33	<0.001
(17HP+PT)/11βOHAT	33	367	0.735;0.475-1.19	360	0.908;0.584-1.56	<0.001	421	0.848;0.610-1.22	404	1.18;0.810-1.64	<0.001
17HP/(AT+ET)	34	357	0.0359;0.0225-0.0607	342	0.0311;0.0181-0.0535	<0.001	387	0.0452;0.0306-0.0653	387	0.0442;0.0311-0.0630	0.0043
PT/(AT+ET)	35	351	0.202;0.143-0.277	341	0.206;0.145-0.293	0.041	369	0.174;0.139-0.219	366	0.177;0.138-0.228	0.027
(17HP+PT)/(AT+ET)	36	351	0.243;0.167-0.350	338	0.241;0.163-0.352	0.57	369	0.224;0.174-0.285	364	0.222;0.173-0.283	0.16

The available number of participants is indicated for each diagnostic ratio stratified for sex. Diagnostic ratios are described by their median;25th-75th percentile. Within-sex differences were determined by Wilcoxon signed-rank test, and the corresponding *p* values are indicated.

Abbreviations: Δ5diol, androstenediol; Δ5triol, androstenetriol, AT, androsterone; 11βOHAT, 11β-OH-androsterone; αC, α-cortol; βC, β-cortol; αCl, α-cortolone; βCl, β-cortolone; DHEA, dehydroepiandrosterone; 16OHDHEA, 16α-OH-dehydroepiandrosterone; E, cortisone; 20βDHE, 20β-DH-cortisone; F, cortisol; ET, etiocholanolone; 11βOHET, 11β-OH-etiocholanolone; 17HP, 17α-OH-pregnanolone; PD, pregnanediol; PT, pregnanetriol; 5PT, pregnenetriol; PTO, pregnanetriolone; THA, tetrahydro-11-dehydro-corticosterone; 18OHTHA, 18-OH-tetrahydro-11-dehydrocorticosterone; THB, tetrahydrocorticosterone; 5α-THB, 5α-tetrahydrocorticosterone; THE, tetrahydrocortisone; THF, tetrahydrocortisol; 5αTHF, 5α-tetrahydrocortisol; 18OHF, 18-OH-cortisol; THS, TH-11-deoxycortisol; THALDO, tetrahydroaldosterone

Supporting Table 3. (continued)

		Women					Men				
		Day		Night			Day		Night		
**Ratio**	**Ratio ID**	**N**	**Median;25th-75th**	**N**	**Median;25th-75th**	***p***	**N**	**Median;25th-75th**	**N**	**Median;25th-75th**	***p***
11βOHAT/(DHEA+16OHDHEA)	37	375	2.99;1.36-6.26	377	2.63;1.17-5.20	<0.001	435	2.11;0.723-5.07	436	1.90;0.705-4.45	<0.001
11βOHAT/Δ^5diol	38	376	10.9;5.83-20.9	377	8.86;5.02-16.3	<0.001	452	7.31;3.32-14.4	451	6.00;2.69-11.3	<0.001
11βOHAT/(DHEA+16OHDHEA+Δ^5diol)	39	375	2.38;1.07-4.65	377	1.96;0.904-3.75	<0.001	435	1.55;0.582-3.64	436	1.45;0.570-3.11	<0.001
11βOHAT/(DHEA+16OHDHEA+Δ^5diol+Δ^5triol)	40	375	1.20;0.672-2.34	376	0.966;0.548-1.85	<0.001	435	0.820;0.413-1.70	435	0.704;0.357-1.35	<0.001
(17HP+PT)/THE	41	362	0.194;0.123-0.340	354	0.271;0.163-0.499	<0.001	399	0.272;0.188-0.372	398	0.391;0.266-0.570	<0.001
(17HP+PT)/(THE+THF+5αTHF)	42	343	0.106;0.0644-0.182	341	0.144;0.0919-0.260	<0.001	341	0.131;0.0962-0.181	365	0.192;0.135-0.287	<0.001
PD/THE	43	368	0.110;0.0578-0.235	366	0.149;0.0808-0.338	<0.001	425	0.0681;0.0445-0.103	435	0.104;0.0628-0.159	<0.001
PD/(THE+THF+5αTHF)	44	344	0.0597;0.0293-0.128	348	0.0798;0.0441-0.184	<0.001	348	0.0332;0.0233-0.0509	388	0.0505;0.0312-0.0794	<0.001
(AT+ET)/(THE+THF+5αTHF)	45	330	0.486;0.250-0.754	326	0.674;0.361-1.14	<0.001	313	0.607;0.416-0.883	352	0.887;0.587-1.38	<0.001
AT/ET	46	357	0.775;0.571-1.04	345	0.752;0.538-0.989	<0.001	387	1.26;0.913-1.60	390	1.20;0.880-1.49	<0.001
ET/AT	47	357	1.29;0.962-1.75	345	1.33;1.01-1.86	<0.001	387	0.796;0.625-1.10	390	0.830;0.673-1.14	<0.001
11βOHET/11βOHAT	48	375	0.571;0.354-0.853	378	0.779;0.436-1.15	<0.001	452	0.379;0.219-0.592	453	0.558;0.290-0.841	<0.001
THF/5αTHF	49	351	1.88;1.33-2.72	355	1.97;1.38-2.70	0.0036	371	1.20;0.900-1.59	401	1.16;0.856-1.60	0.074
THB/5αTHB	50	379	0.571;0.422-0.762	379	0.806;0.583-1.09	<0.001	458	0.383;0.297-0.537	459	0.539;0.406-0.739	<0.001
testosterone/17β-estradiol	51	373	3.37;1.76-7.89	369	3.43;1.68-7.57	0.22	455	17.9;11.0-29.8	454	18.4;10.6-29.8	0.80
F/E	52	379	0.617;0.458-0.856	379	0.600;0.442-0.799	<0.001	458	0.665;0.512-0.863	458	0.629;0.506-0.817	<0.001
(THF+5αTHF)/THE	53	344	0.905;0.758-1.08	350	0.862;0.729-1.02	<0.001	348	1.06;0.896-1.27	388	1.04;0.861-1.25	<0.001
(αC+βC)/(αCl+βCl)	54	362	0.380;0.327-0.457	366	0.412;0.340-0.499	<0.001	410	0.412;0.356-0.496	431	0.463;0.380-0.574	<0.001
(F+E)/(THF+5αTHF+THE)	55	344	0.838;0.791-0.879	350	0.845;0.800-0.884	<0.001	348	0.769;0.716-0.816	388	0.771;0.713-0.821	0.96
THE/(THF+5αTHF)	56	344	1.11;0.930-1.32	350	1.16;0.978-1.37	<0.001	348	0.947;0.785-1.12	388	0.959;0.800-1.16	<0.001
(αCl+βCl)/(αC+βC)	57	362	2.63;2.19-3.06	366	2.43;2.00-2.94	<0.001	410	2.43;2.01-2.81	431	2.16;1.74-2.63	<0.001
(THF+5αTHF+THE)/(αC+αCl)	58	341	1.72;1.38-2.03	350	1.67;1.34-1.98	0.0097	352	2.17;1.80-2.62	391	2.16;1.84-2.64	0.46
(THF+5αTHF+THE)/βC+βCl	59	350	2.92;2.28-3.70	354	2.56;1.87-3.41	<0.001	366	3.15;2.47-3.97	399	2.65;1.95-3.42	<0.001
(αC+αCl)/(βC+βCl)	60	362	1.77;1.42-2.14	366	1.57;1.18-2.02	<0.001	410	1.45;1.21-1.80	431	1.22;0.907-1.55	<0.001
20αDHF/(THF+5αTHF)	61	351	0.0231;0.0158-0.0341	355	0.0257;0.0174-0.0391	<0.001	371	0.0149;0.0108-0.0222	401	0.0160;0.0104-0.0241	0.19
(androstanediol$^{1.5}$×20βDHE)/(20βDHE+[F×log(estriol)]	62	373	73.7;32.6-138	370	32.9;14.2-68.8	<0.001	-	-	-	-	-
F/18OHF	63	367	0.511;0.284-0.947	349	0.570;0.341-1.11	<0.001	448	0.648;0.365-1.11	432	0.745;0.450-1.34	<0.001
THALDO×100/(THE+THF+5αTHF)	64	344	0.415;0.247-0.765	349	0.498;0.311-0.868	<0.001	348	0.263;0.174-0.424	387	0.323;0.222-0.543	<0.001
18OHTHA/THALDO	65	359	2.03;1.01-3.86	350	1.62;0.812-3.85	0.0037	449	2.72;1.50-5.00	439	2.53;1.44-5.19	0.095

The available number of participants is indicated for each diagnostic ratio stratified for sex. Diagnostic ratios are described by their median;25th-75th percentile. Within-sex differences were determined by Wilcoxon signed-rank test, and the corresponding *p* values are indicated.

Abbreviations: Δ^5diol, androstenediol; Δ^5triol, androstenetriol, AT, androsterone; 11βOHAT, 11β-OH-androsterone; αC, α-cortol; βC, β-cortol; αCl, α-cortolone; βCl, β-cortolone; DHEA, dehydroepiandrosterone; 16OHDHEA, 16α-OH-dehydroepiandrosterone; E, cortisone; 20βDHE, 20β-DH-cortisone; F, cortisol; ET, etiocholanolone; 11βOHET, 11β-OH-etiocholanolone; 17HP, 17α-OH-pregnanolone; PD, pregnanediol; PT, pregnanetriol; 5PT, pregnenetriol; PTO, pregnanetriolone; THA, tetrahydro-11-dehydro-corticosterone; 18OHTHA, 18-OH-tetrahydro-11-dehydrocorticosterone; THB, tetrahydrocorticosterone; 5α-THB, 5α-tetrahydrocorticosterone; THE, tetrahydrocortisone; THF, tetrahydrocortisol; 5αTHF, 5α-tetrahydrocortisol; 18OHF, 18-OH-cortisol; THS, TH-11-deoxycortisol; THALDO, tetrahydroaldosterone
