# Supplementary material for: Sex- and age-specific reference intervals for diagnostic ratios reflecting relative activity of steroidogenic enzymes and pathways in adults
Source: PLoS One. 2021 Jul 8;16(7):e0253975. doi: 10.1371/journal.pone.0253975 (PMC8266106; doi:10.1371/journal.pone.0253975)
Supplement: S4 Table — Percentiles were obtained from the described statistical models. Three significant digits were indicated for most percentiles. N represents the sample number per ratio. (PDF) [file pone.0253975.s008.pdf]

Supporting Table 4. 1st-50th-99th percentiles for diagnostic ratios of 24-hour urine steroid hormone metabolites in women of different ages. Percentiles were obtained from the described statistical models. Three significant digits were indicated for most percentiles. N represents the sample number per ratio.

**Women**		**Age, years**						
**Ratio ID**	**N**	**20**	**30**	**40**	**50**	**60**	**70**	**80**
**1**	360	0.0022-0.0072-0.0752	0.0020-0.0061-0.0495	0.0019-0.0056-0.0412	0.0019-0.0056-0.0415	0.0020-0.0061-0.0506	0.0022-0.0074-0.0786	0.0027-0.0099-0.183
**2**	329	0.0012-0.0039-0.0412	0.0011-0.0033-0.0272	0.0010-0.0030-0.0225	0.0010-0.0030-0.0223	0.0011-0.0033-0.0264	0.0012-0.0038-0.0390	0.0014-0.0050-0.0814
**3**	356	0.0081-0.0461-0.263	0.0124-0.0709-0.405	0.0115-0.0659-0.376	0.0065-0.0370-0.211	0.0035-0.0198-0.113	0.0028-0.0161-0.0920	0.0028-0.0161-0.0920
**4**	328	0.0046-0.0251-0.194	0.0066-0.0384-0.327	0.0060-0.0345-0.287	0.0036-0.0187-0.135	0.0021-0.0101-0.0640	0.0018-0.0083-0.0505	0.0018-0.0083-0.0505
**5**	348	0.0811-0.270-0.897	0.103-0.343-1.14	0.0934-0.311-1.03	0.0603-0.200-0.666	0.0382-0.127-0.422	0.0328-0.109-0.363	0.0328-0.109-0.363
**6**	323	0.0452-0.150-0.500	0.0553-0.184-0.612	0.0491-0.163-0.543	0.0317-0.105-0.350	0.0202-0.0673-0.224	0.0174-0.0579-0.193	0.0174-0.0579-0.193
**7**	344	0.103-0.334-1.08	0.132-0.425-1.37	0.120-0.386-1.25	0.0777-0.251-0.810	0.0496-0.160-0.517	0.0427-0.138-0.445	0.0427-0.138-0.445
**8**	322	0.0578-0.183-0.674	0.0702-0.228-0.864	0.0631-0.202-0.753	0.0424-0.129-0.454	0.0286-0.0835-0.277	0.0252-0.0725-0.236	0.0252-0.0725-0.236
**9**	359	0.0106-0.112-0.549	0.0053-0.0731-0.405	0.0026-0.0491-0.307	0.0014-0.0341-0.239	0.0007-0.0248-0.193	0.0004-0.0191-0.163	0.0003-0.0157-0.143
**10**	328	0.0059-0.0607-0.296	0.0028-0.0385-0.213	0.0013-0.0251-0.158	0.0006-0.0171-0.121	0.0003-0.0123-0.0973	0.0002-0.0094-0.0819	0.0001-0.0078-0.0727
**11**	358	0.0070-0.0772-1.83	0.0076-0.0856-2.11	0.0064-0.0693-1.58	0.0043-0.0418-0.794	0.0028-0.0242-0.384	0.0021-0.0167-0.236	0.0017-0.0135-0.179
**12**	329	0.0059-0.0775-2.53	0.0041-0.0489-1.32	0.0029-0.0314-0.721	0.0021-0.0206-0.406	0.0015-0.0137-0.236	0.0011-0.0093-0.141	0.0008-0.0064-0.0866
**13**	358	0.0256-0.244-2.32	0.0284-0.271-2.58	0.0215-0.205-1.95	0.0111-0.105-1.00	0.0058-0.0554-0.528	0.0047-0.0447-0.426	0.0047-0.0447-0.426
**14**	329	0.0165-0.165-1.64	0.0146-0.145-1.44	0.0103-0.103-1.02	0.0059-0.0588-0.586	0.0033-0.0329-0.328	0.0022-0.0219-0.219	0.0017-0.0174-0.173
**15**	378	0.435-11.5-81.3	0.588-13.4-90.5	0.355-10.3-75.7	0.0886-5.33-49.1	0.0256-3.20-35.7	0.0157-2.67-31.9	0.0157-2.67-31.9
**16**	360	0.0108-0.0242-0.0702	0.0110-0.0248-0.0726	0.0112-0.0254-0.0751	0.0114-0.0260-0.0777	0.0116-0.0266-0.0804	0.0119-0.0273-0.0833	0.0121-0.0280-0.0863
**17**	329	0.0058-0.0138-0.0391	0.0058-0.0138-0.0391	0.0058-0.0138-0.0391	0.0058-0.0138-0.0391	0.0058-0.0138-0.0391	0.0058-0.0138-0.0391	0.0058-0.0138-0.0391
**18**	327	0.0193-0.0849-0.708	0.0300-0.156-1.84	0.0350-0.193-2.64	0.0297-0.154-1.81	0.0244-0.117-1.16	0.0248-0.120-1.21	0.0316-0.168-2.09
**19**	330	0.0290-0.0879-0.365	0.0352-0.112-0.504	0.0429-0.144-0.712	0.0528-0.188-1.03	0.0656-0.249-1.54	0.0823-0.335-2.37	0.104-0.459-3.81
**20**	360	0.0774-0.203-0.454	0.0833-0.215-0.479	0.0817-0.212-0.472	0.0729-0.193-0.436	0.0644-0.174-0.400	0.0617-0.168-0.388	0.0617-0.168-0.388
**21**	329	0.0517-0.120-0.245	0.0480-0.112-0.232	0.0445-0.106-0.220	0.0413-0.0989-0.208	0.0382-0.0927-0.197	0.0353-0.0868-0.186	0.0326-0.0811-0.176
**22**	372	0.862-4.05-19.0	0.935-4.39-20.6	0.967-4.54-21.3	0.954-4.48-21.1	0.897-4.22-19.8	0.805-3.78-17.8	0.689-3.24-15.2
**23**	357	0.185-0.598-3.73	0.237-0.857-7.13	0.252-0.939-8.48	0.220-0.769-5.83	0.186-0.605-3.79	0.177-0.560-3.32	0.177-0.560-3.32
**24**	353	0.163-0.491-2.59	0.211-0.709-4.85	0.226-0.785-5.83	0.199-0.650-4.17	0.168-0.514-2.80	0.160-0.477-2.47	0.160-0.477-2.47
**25**	320	0.535-2.11-7.05	0.476-1.91-6.44	0.362-1.50-5.21	0.231-1.02-3.70	0.141-0.666-2.55	0.0956-0.478-1.91	0.0729-0.380-1.57
**26**	297	0.352-1.24-3.38	0.291-1.07-3.00	0.202-0.808-2.39	0.117-0.537-1.72	0.0680-0.362-1.27	0.0421-0.258-0.975	0.0285-0.198-0.795
**27**	376	0.0246-0.378-3.23	0.0170-0.286-2.58	0.0148-0.258-2.38	0.0166-0.281-2.54	0.0236-0.367-3.15	0.0296-0.436-3.63	0.0296-0.436-3.63
**28**	377	0.125-1.47-7.62	0.101-1.29-6.94	0.0884-1.19-6.56	0.0851-1.17-6.45	0.0900-1.21-6.61	0.104-1.32-7.04	0.131-1.52-7.79
**29**	377	0.0085-0.408-2.33	0.0085-0.408-2.33	0.0085-0.408-2.33	0.0085-0.408-2.33	0.0085-0.408-2.33	0.0085-0.408-2.33	0.0085-0.408-2.33
**30**	375	0.0035-0.123-0.660	0.0042-0.131-0.686	0.0050-0.139-0.713	0.0059-0.148-0.740	0.0069-0.157-0.768	0.0080-0.167-0.797	0.0092-0.177-0.827
**31**	372	0.0322-0.179-1.42	0.0429-0.252-2.17	0.0389-0.224-1.88	0.0242-0.128-0.940	0.0151-0.0731-0.478	0.0129-0.0611-0.385	0.0129-0.0611-0.385
**32**	357	0.365-1.15-3.64	0.365-1.15-3.65	0.318-1.00-3.17	0.240-0.758-2.40	0.163-0.516-1.63	0.124-0.393-1.24	0.110-0.349-1.10
**33**	353	0.412-1.33-5.01	0.430-1.40-5.30	0.383-1.23-4.56	0.292-0.905-3.23	0.201-0.596-2.01	0.157-0.453-1.48	0.144-0.410-1.32
**34**	328	0.0047-0.0234-0.161	0.0068-0.0369-0.280	0.0077-0.0426-0.334	0.0066-0.0356-0.268	0.0057-0.0295-0.213	0.0062-0.0328-0.242	0.0087-0.0492-0.399
**35**	324	0.0556-0.143-0.457	0.0650-0.173-0.580	0.0716-0.194-0.673	0.0739-0.201-0.708	0.0730-0.199-0.695	0.0787-0.218-0.782	0.0951-0.275-1.06
**36**	322	0.0624-0.166-0.562	0.0769-0.215-0.781	0.0851-0.243-0.917	0.0838-0.239-0.896	0.0821-0.232-0.866	0.0892-0.258-0.990	0.108-0.327-1.36
**37**	374	0.0523-0.714-5.66	0.0951-1.13-8.26	0.167-1.77-11.9	0.285-2.70-16.9	0.473-4.05-23.7	0.765-5.99-32.9	1.21-8.72-45.3
**38**	375	0.516-3.71-19.2	0.784-5.23-25.8	1.17-7.31-34.3	1.72-10.1-45.3	2.50-13.8-59.3	3.58-18.7-77.1	5.06-25.1-99.5
**39**	374	0.0574-0.588-3.88	0.0991-0.909-5.57	0.166-1.38-7.90	0.272-2.06-11.1	0.436-3.03-15.3	0.682-4.39-21.0	1.05-6.27-28.6
**40**	373	0.0763-0.363-1.72	0.111-0.525-2.50	0.160-0.761-3.62	0.232-1.10-5.24	0.336-1.60-7.59	0.487-2.31-11.0	0.706-3.35-15.9
**41**	344	0.0959-0.318-1.05	0.126-0.417-1.38	0.115-0.380-1.26	0.0726-0.241-0.799	0.0450-0.149-0.495	0.0384-0.127-0.422	0.0384-0.127-0.422
**42**	322	0.0536-0.174-0.661	0.0669-0.223-0.880	0.0603-0.199-0.769	0.0396-0.124-0.449	0.0261-0.0778-0.265	0.0228-0.0669-0.223	0.0228-0.0669-0.223
**43**	358	0.0236-0.172-2.06	0.0383-0.312-4.45	0.0370-0.299-4.20	0.0213-0.152-1.76	0.0120-0.0760-0.730	0.0100-0.0609-0.554	0.0100-0.0609-0.554
**44**	327	0.0114-0.0874-1.14	0.0191-0.166-2.62	0.0188-0.162-2.55	0.0108-0.0821-1.05	0.0060-0.0405-0.426	0.0050-0.0324-0.320	0.0050-0.0324-0.320
**45**	297	0.352-1.24-3.38	0.291-1.07-3.00	0.202-0.808-2.39	0.117-0.537-1.72	0.0680-0.362-1.27	0.0421-0.258-0.975	0.0285-0.198-0.795
**46**	330	0.444-1.14-3.21	0.366-0.919-2.54	0.317-0.786-2.13	0.289-0.709-1.90	0.275-0.673-1.80	0.275-0.673-1.80	0.289-0.709-1.90
**47**	330	0.311-0.879-2.25	0.394-1.09-2.73	0.469-1.27-3.15	0.526-1.41-3.47	0.556-1.49-3.63	0.556-1.49-3.63	0.526-1.41-3.47
**48**	375	0.0003-0.366-1.51	0.0085-0.510-1.79	0.0282-0.624-2.00	0.0439-0.692-2.11	0.0472-0.705-2.14	0.0365-0.661-2.06	0.0170-0.567-1.89
**49**	339	0.537-1.55-5.08	0.505-1.45-4.70	0.587-1.71-5.67	0.672-1.99-6.73	0.730-2.18-7.47	0.751-2.25-7.74	0.751-2.25-7.74
**50**	379	0.173-0.460-1.36	0.201-0.542-1.63	0.225-0.613-1.87	0.241-0.662-2.04	0.248-0.685-2.12	0.246-0.677-2.09	0.234-0.640-1.96
**51**	366	0.495-6.62-51.8	0.197-3.27-29.3	0.139-2.52-23.8	0.186-3.14-28.3	0.289-4.38-37.1	0.334-4.89-40.5	0.334-4.89-40.5
**52**	379	0.220-0.567-1.62	0.225-0.582-1.66	0.230-0.597-1.71	0.236-0.613-1.76	0.241-0.629-1.81	0.247-0.646-1.87	0.253-0.663-1.92
**53**	329	0.363-0.710-1.33	0.460-0.885-1.64	0.478-0.917-1.69	0.461-0.887-1.64	0.465-0.895-1.65	0.491-0.941-1.73	0.541-1.03-1.89
**54**	353	0.181-0.313-0.580	0.218-0.386-0.735	0.234-0.419-0.808	0.228-0.406-0.779	0.222-0.394-0.752	0.220-0.390-0.744	0.220-0.390-0.744
**55**	329	0.690-0.832-0.975	0.666-0.809-0.951	0.680-0.823-0.965	0.694-0.837-0.979	0.703-0.845-0.988	0.706-0.848-0.991	0.706-0.848-0.991
**56**	329	0.752-1.41-2.75	0.611-1.13-2.18	0.591-1.09-2.09	0.610-1.13-2.17	0.605-1.12-2.15	0.577-1.06-2.04	0.530-0.970-1.85
**57**	353	1.73-3.20-5.54	1.36-2.59-4.59	1.24-2.39-4.27	1.28-2.46-4.39	1.33-2.54-4.51	1.34-2.57-4.55	1.34-2.57-4.55
**58**	326	0.441-1.10-2.15	0.718-1.56-2.81	0.795-1.68-2.99	0.828-1.73-3.06	0.848-1.76-3.10	0.855-1.77-3.12	0.855-1.77-3.12
**59**	337	0.611-1.94-4.26	0.931-2.55-5.22	1.09-2.84-5.67	1.14-2.93-5.80	1.17-2.98-5.88	1.18-3.00-5.90	1.18-3.00-5.90
**60**	353	0.761-1.70-3.40	0.761-1.70-3.40	0.761-1.70-3.40	0.761-1.70-3.40	0.761-1.70-3.40	0.761-1.70-3.40	0.761-1.70-3.40
**61**	339	0.0086-0.0319-0.146	0.0079-0.0292-0.131	0.0073-0.0267-0.118	0.0068-0.0245-0.107	0.0063-0.0224-0.0966	0.0058-0.0206-0.0874	0.0054-0.0189-0.0792
**62**	367	26.1-274-2876	20.0-210-2199	15.3-160-1681	11.7-123-1285	8.93-93.7-983	6.83-71.6-751	5.22-54.8-575
**63**	344	0.111-0.514-4.73	0.111-0.514-4.73	0.111-0.514-4.73	0.111-0.514-4.73	0.111-0.514-4.73	0.111-0.514-4.73	0.111-0.514-4.73
**64**	329	0.136-0.649-6.39	0.124-0.574-5.27	0.114-0.509-4.38	0.104-0.453-3.66	0.0957-0.404-3.08	0.0880-0.361-2.60	0.0810-0.324-2.22
**65**	341	0.162-1.52-27.6	0.171-1.64-30.4	0.182-1.76-33.6	0.193-1.90-37.1	0.205-2.05-41.0	0.217-2.20-45.3	0.230-2.38-50.2
