# Supplementary material for: Sex- and age-specific reference intervals for diagnostic ratios reflecting relative activity of steroidogenic enzymes and pathways in adults
Source: PLoS One. 2021 Jul 8;16(7):e0253975. doi: 10.1371/journal.pone.0253975 (PMC8266106; doi:10.1371/journal.pone.0253975)
Supplement: S5 Table — Percentiles were obtained from the described statistical models. Three significant digits were indicated for most percentiles. N represents the sample number per ratio. (PDF) [file pone.0253975.s009.pdf]

Supporting Table 5. 1st-50th-99th percentiles for diagnostic ratios of 24-hour urine steroid hormone metabolites in men of different ages. Percentiles were obtained from the described statistical models. Three significant digits were indicated for most percentiles. N represents the sample number per ratio.

Men		Age, years						
**Ratio ID**	**N**	**20**	**30**	**40**	**50**	**60**	**70**	**80**
**1**	407	0.0017-0.0048-0.0282	0.0017-0.0048-0.0279	0.0018-0.0050-0.0296	0.0018-0.0053-0.0340	0.0020-0.0059-0.0428	0.0022-0.0069-0.0608	0.0025-0.0085-0.103
**2**	321	0.0009-0.0024-0.0140	0.0009-0.0025-0.0151	0.0009-0.0026-0.0163	0.0009-0.0027-0.0176	0.0010-0.0028-0.0191	0.0010-0.0029-0.0207	0.0010-0.0030-0.0226
**3**	402	0.0183-0.0709-0.275	0.0170-0.0660-0.256	0.0159-0.0615-0.239	0.0148-0.0573-0.222	0.0138-0.0534-0.207	0.0128-0.0497-0.193	0.0119-0.0463-0.180
**4**	320	0.0102-0.0374-0.138	0.0093-0.0341-0.126	0.0085-0.0311-0.115	0.0077-0.0284-0.105	0.0071-0.0260-0.0956	0.0064-0.0237-0.0872	0.0059-0.0216-0.0796
**5**	362	0.104-0.309-0.913	0.0946-0.280-0.828	0.0858-0.254-0.751	0.0778-0.230-0.681	0.0705-0.209-0.618	0.0640-0.189-0.560	0.0580-0.172-0.508
**6**	303	0.0596-0.162-0.490	0.0534-0.143-0.428	0.0479-0.127-0.374	0.0430-0.113-0.328	0.0387-0.100-0.288	0.0348-0.0893-0.253	0.0313-0.0796-0.223
**7**	360	0.135-0.387-1.11	0.124-0.355-1.02	0.114-0.326-0.933	0.104-0.299-0.856	0.0956-0.274-0.785	0.0877-0.251-0.720	0.0805-0.231-0.661
**8**	303	0.0777-0.204-0.595	0.0702-0.183-0.525	0.0634-0.163-0.464	0.0573-0.146-0.410	0.0519-0.131-0.364	0.0470-0.118-0.323	0.0426-0.106-0.287
**9**	402	0.0150-0.121-0.525	0.0192-0.142-0.593	0.0101-0.0935-0.434	0.0049-0.0597-0.313	0.0027-0.0418-0.243	0.0018-0.0329-0.206	0.0015-0.0296-0.191
**10**	321	0.0073-0.0623-0.278	0.0083-0.0677-0.295	0.0048-0.0475-0.228	0.0020-0.0274-0.153	0.0009-0.0177-0.113	0.0005-0.0133-0.0927	0.0004-0.0120-0.0863
**11**	395	0.0113-0.121-12.7	0.0173-0.243-96.0	0.0125-0.142-19.6	0.0071-0.0583-2.39	0.0048-0.0328-0.762	0.0038-0.0232-0.406	0.0034-0.0198-0.307
**12**	315	0.0053-0.0618-9.39	0.0077-0.116-66.5	0.0057-0.0688-12.7	0.0033-0.0287-1.45	0.0022-0.0157-0.419	0.0017-0.0104-0.194	0.0014-0.0080-0.122
**13**	389	0.0364-0.315-4.95	0.0553-0.531-9.92	0.0373-0.325-5.17	0.0192-0.143-1.78	0.0122-0.0825-0.879	0.0093-0.0597-0.585	0.0085-0.0531-0.505
**14**	312	0.0176-0.160-2.74	0.0263-0.265-5.39	0.0177-0.162-2.78	0.0090-0.0702-0.931	0.0055-0.0387-0.433	0.0040-0.0261-0.263	0.0033-0.0210-0.201
**15**	448	1.93-23.0-119	2.82-29.0-142	1.26-17.8-99.4	0.471-10.2-67.6	0.189-6.36-49.1	0.0883-4.43-38.7	0.0535-3.55-33.6
**16**	407	0.0087-0.0208-0.0536	0.0077-0.0181-0.0460	0.0084-0.0198-0.0507	0.0093-0.0221-0.0574	0.0102-0.0246-0.0645	0.0112-0.0271-0.0718	0.0121-0.0296-0.0792
**17**	321	0.0049-0.0107-0.0265	0.0041-0.0086-0.0204	0.0046-0.0098-0.0239	0.0052-0.0112-0.0282	0.0055-0.0122-0.0313	0.0057-0.0126-0.0324	0.0057-0.0126-0.0324
**18**	361	0.0180-0.0459-0.117	0.0181-0.0462-0.118	0.0190-0.0484-0.124	0.0207-0.0529-0.135	0.0236-0.0603-0.154	0.0280-0.0715-0.183	0.0347-0.0885-0.226
**19**	359	0.0440-0.0997-0.296	0.0466-0.107-0.327	0.0505-0.119-0.379	0.0562-0.137-0.463	0.0644-0.164-0.600	0.0762-0.205-0.841	0.0935-0.271-1.30
**20**	404	0.0995-0.225-0.453	0.0889-0.204-0.417	0.0824-0.192-0.394	0.0793-0.186-0.383	0.0794-0.186-0.384	0.0826-0.192-0.395	0.0893-0.205-0.418
**21**	320	0.0537-0.106-0.193	0.0515-0.102-0.187	0.0485-0.0972-0.179	0.0450-0.0910-0.169	0.0410-0.0839-0.157	0.0402-0.0825-0.155	0.0464-0.0935-0.173
**22**	451	0.423-1.37-5.21	0.395-1.27-4.78	0.380-1.22-4.55	0.377-1.21-4.50	0.385-1.23-4.62	0.405-1.31-4.93	0.438-1.43-5.46
**23**	395	0.113-0.288-0.731	0.129-0.329-0.834	0.131-0.333-0.845	0.121-0.308-0.781	0.120-0.306-0.776	0.132-0.335-0.850	0.160-0.405-1.03
**24**	392	0.0865-0.232-0.568	0.0971-0.257-0.624	0.0983-0.260-0.631	0.0921-0.245-0.598	0.0916-0.244-0.595	0.0990-0.262-0.635	0.116-0.302-0.724
**25**	340	0.799-2.18-5.45	0.662-1.84-4.66	0.547-1.55-3.99	0.450-1.30-3.40	0.369-1.09-2.89	0.301-0.907-2.45	0.245-0.753-2.07
**26**	280	0.461-1.16-2.71	0.373-0.958-2.27	0.300-0.786-1.89	0.240-0.643-1.57	0.192-0.524-1.31	0.152-0.425-1.08	0.120-0.343-0.887
**27**	427	0.0461-0.329-2.34	0.0299-0.213-1.52	0.0346-0.247-1.76	0.0499-0.356-2.54	0.0621-0.443-3.16	0.0668-0.477-3.40	0.0668-0.477-3.40
**28**	446	0.243-1.52-4.93	0.197-1.35-4.53	0.175-1.26-4.33	0.172-1.25-4.30	0.188-1.31-4.45	0.226-1.46-4.78	0.294-1.70-5.33
**29**	447	0.0374-0.547-2.37	0.0541-0.648-2.65	0.0373-0.546-2.37	0.0250-0.459-2.12	0.0192-0.412-1.98	0.0175-0.397-1.94	0.0175-0.397-1.94
**30**	426	0.0191-0.150-0.532	0.0160-0.137-0.500	0.0159-0.136-0.498	0.0185-0.148-0.527	0.0250-0.174-0.590	0.0298-0.192-0.632	0.0298-0.192-0.632
**31**	443	0.0519-0.236-0.752	0.0469-0.219-0.709	0.0423-0.203-0.668	0.0380-0.188-0.628	0.0341-0.174-0.591	0.0305-0.160-0.555	0.0273-0.148-0.521
**32**	391	0.335-0.986-2.90	0.302-0.890-2.62	0.273-0.804-2.37	0.247-0.726-2.14	0.223-0.655-1.93	0.201-0.592-1.74	0.182-0.534-1.57
**33**	389	0.425-1.23-3.56	0.385-1.11-3.22	0.349-1.01-2.92	0.317-0.917-2.65	0.287-0.831-2.40	0.261-0.754-2.18	0.236-0.683-1.98
**34**	358	0.0076-0.0334-0.104	0.0088-0.0371-0.113	0.0101-0.0412-0.123	0.0116-0.0457-0.134	0.0132-0.0505-0.145	0.0151-0.0558-0.157	0.0171-0.0615-0.170
**35**	336	0.0772-0.148-0.333	0.0779-0.150-0.337	0.0806-0.156-0.355	0.0857-0.168-0.391	0.0938-0.188-0.452	0.106-0.218-0.549	0.124-0.265-0.712
**36**	334	0.0936-0.185-0.405	0.0952-0.188-0.414	0.0994-0.198-0.439	0.107-0.214-0.483	0.118-0.240-0.552	0.134-0.279-0.658	0.157-0.336-0.821
**37**	424	0.0755-1.06-8.57	0.0330-0.566-5.16	0.0622-0.916-7.60	0.189-2.16-15.3	0.398-3.90-24.9	0.629-5.61-33.8	0.770-6.61-38.7
**38**	445	0.619-4.74-25.7	0.351-2.99-17.4	0.492-3.93-22.0	0.985-6.93-35.5	1.69-10.8-51.8	2.53-15.2-69.3	3.38-19.3-85.2
**39**	424	0.0797-0.844-5.69	0.0384-0.476-3.55	0.0658-0.726-5.02	0.175-1.58-9.57	0.348-2.75-15.2	0.545-3.97-20.8	0.694-4.84-24.6
**40**	423	0.0787-0.470-2.14	0.0498-0.321-1.55	0.0768-0.461-2.11	0.162-0.864-3.62	0.280-1.37-5.42	0.406-1.89-7.16	0.505-2.28-8.43
**41**	360	0.131-0.383-1.12	0.120-0.349-1.02	0.109-0.318-0.929	0.0995-0.290-0.847	0.0907-0.265-0.772	0.0827-0.241-0.704	0.0754-0.220-0.642
**42**	303	0.0757-0.201-0.595	0.0681-0.179-0.523	0.0613-0.160-0.460	0.0553-0.142-0.405	0.0499-0.127-0.357	0.0451-0.114-0.316	0.0407-0.102-0.280
**43**	407	0.0250-0.102-0.353	0.0206-0.0867-0.305	0.0178-0.0765-0.273	0.0162-0.0705-0.254	0.0155-0.0678-0.245	0.0156-0.0681-0.246	0.0165-0.0716-0.257
**44**	321	0.0120-0.0510-0.156	0.0106-0.0464-0.145	0.0093-0.0421-0.134	0.0081-0.0381-0.124	0.0071-0.0345-0.114	0.0061-0.0311-0.106	0.0053-0.0280-0.0972
**45**	280	0.461-1.16-2.71	0.373-0.958-2.27	0.300-0.786-1.89	0.240-0.643-1.57	0.192-0.524-1.31	0.152-0.425-1.08	0.120-0.343-0.887
**46**	361	0.512-1.41-3.27	0.478-1.33-3.12	0.446-1.26-2.98	0.416-1.19-2.84	0.387-1.12-2.70	0.360-1.06-2.57	0.334-0.997-2.45
**47**	361	0.306-0.709-1.95	0.320-0.750-2.09	0.336-0.794-2.24	0.353-0.841-2.41	0.370-0.891-2.59	0.389-0.945-2.78	0.409-1.00-2.99
**48**	447	0.0237-0.387-1.40	0.0084-0.288-1.18	0.0189-0.360-1.34	0.0347-0.441-1.52	0.0470-0.495-1.63	0.0517-0.513-1.66	0.0517-0.513-1.66
**49**	351	0.415-0.930-2.70	0.460-1.06-3.24	0.501-1.18-3.77	0.532-1.28-4.23	0.554-1.35-4.55	0.562-1.37-4.68	0.557-1.36-4.60
**50**	457	0.145-0.346-0.994	0.163-0.399-1.19	0.176-0.438-1.33	0.182-0.456-1.40	0.180-0.449-1.38	0.184-0.459-1.41	0.208-0.534-1.71
**51**	451	5.53-31.9-117	4.23-26.5-101	3.18-21.8-87.0	2.36-17.8-74.6	1.71-14.3823-63.7199	1.21-11.5315-54.1307	0.841-9.15-45.7323
**52**	456	0.240-0.578-1.39	0.283-0.680-1.64	0.269-0.647-1.56	0.261-0.627-1.51	0.267-0.642-1.54	0.288-0.694-1.67	0.330-0.793-1.91
**53**	321	0.557-0.918-1.72	0.578-0.961-1.82	0.601-1.01-1.94	0.624-1.06-2.06	0.650-1.11-2.20	0.676-1.17-2.35	0.704-1.23-2.51
**54**	398	0.223-0.379-0.713	0.248-0.429-0.830	0.250-0.435-0.843	0.245-0.423-0.816	0.246-0.426-0.822	0.255-0.444-0.864	0.272-0.479-0.948
**55**	321	0.587-0.757-0.926	0.592-0.762-0.932	0.597-0.767-0.937	0.603-0.772-0.942	0.608-0.777-0.947	0.613-0.782-0.952	0.618-0.787-0.957
**56**	321	0.583-1.09-1.80	0.549-1.04-1.73	0.516-0.993-1.67	0.485-0.946-1.60	0.455-0.901-1.54	0.426-0.857-1.48	0.398-0.814-1.42
**57**	398	1.40-2.64-4.49	1.21-2.33-4.04	1.19-2.30-4.00	1.23-2.36-4.09	1.22-2.35-4.07	1.16-2.25-3.93	1.05-2.09-3.68
**58**	328	1.19-1.98-3.79	1.32-2.26-4.49	1.30-2.22-4.39	1.27-2.16-4.23	1.25-2.12-4.13	1.25-2.11-4.10	1.25-2.11-4.10
**59**	345	1.21-2.68-5.59	1.27-2.80-5.82	1.33-2.93-6.06	1.40-3.06-6.31	1.46-3.19-6.57	1.53-3.33-6.84	1.61-3.48-7.12
**60**	398	0.564-1.22-2.38	0.599-1.29-2.49	0.635-1.35-2.61	0.673-1.42-2.72	0.713-1.49-2.85	0.755-1.57-2.97	0.799-1.65-3.10
**61**	351	0.0047-0.0158-0.0468	0.0047-0.0158-0.0468	0.0047-0.0158-0.0468	0.0047-0.0158-0.0468	0.0047-0.0158-0.0468	0.0047-0.0158-0.0468	0.0047-0.0158-0.0468
**63**	424	0.116-0.588-4.08	0.121-0.617-4.33	0.126-0.647-4.59	0.131-0.679-4.87	0.137-0.713-5.16	0.142-0.748-5.48	0.148-0.786-5.81
**64**	320	0.0602-0.295-1.45	0.0602-0.295-1.45	0.0602-0.295-1.45	0.0602-0.295-1.45	0.0602-0.295-1.45	0.0602-0.295-1.45	0.0602-0.295-1.45
**65**	432	0.362-2.52-62.7	0.362-2.52-62.7	0.362-2.52-62.7	0.362-2.52-62.7	0.362-2.52-62.7	0.362-2.52-62.7	0.362-2.52-62.7
