# Supplementary material for: Sex- and age-specific reference intervals for diagnostic ratios reflecting relative activity of steroidogenic enzymes and pathways in adults
Source: PLoS One. 2021 Jul 8;16(7):e0253975. doi: 10.1371/journal.pone.0253975 (PMC8266106; doi:10.1371/journal.pone.0253975)
Supplement: S1 Text — The statistical methods are described in detail. (DOCX) [file pone.0253975.s010.docx]

**Supporting Text 1. Description of statistical analysis**

**Statistical Methods.**

The statistical methods applied are described in detail. A similar statistical approach was chosen and described in a previous publication from our research group [1].

Data for 65 diagnostic ratios based on urinary steroid hormone metabolites measured in 24-hour urine collections were available for 379 women, and data for 64 diagnostic ratios were available for 459 men. The goal was to obtain reference intervals in function of age (between 18 and 90 years) and sex. In a first descriptive analysis conducted on the log-scale, nonparametric fits revealed a different kind of relationship to age for men and for women for most diagnostic ratios. We thus modeled separately the data for men and women, leaving us with 129 models to develop (65 for women and 64 for men).

To get reference values, one convenient approach is to look for a scale where near normality is achieved, enabling to derive any percentile of the distribution, and thus the calculation of standard deviation scores (SDS) values. This is in contrast to other approaches, such as regression quantile, where one has to restrict one’s interest on some particular percentiles. Since each diagnostic ratio R was often highly skewed, we started to correct for skewness via power transformation, yielding Y=sign(p)*R^p, where the optimal power p was selected on a fine grid ranging from -1 (corresponding to an inverse transformation) to 1 (corresponding to no transformation), and where the power 0 corresponded to a log-transformation [2], to minimize the absolute value of skewness of Y (this was again done separately for men and women). To minimize their influence, the transformation was selected by removing the obvious outliers, which were detected sequentially using a boxplot rule, eliminating just 1 of 10’000 observations in case of a perfect normality on the transformed scale.

The data Y hence transformed were fitted in a linear mixed model with two nested random effects, a family effect (since individuals were nested into families) and a center effect (since families were nested into centers). Parameters to capture the age effect were included as fixed effects. Five such models have been considered: a constant function (i.e. no age effect, just an intercept), a linear function (2 parameters), a quadratic function (3 parameters), a quadratic spline function with one knot (5 parameters including the position of the knot) and a quadratic spline function with two knots and a levelling-off (4 parameters since the second knot was fixed), i.e. a function which becomes constant after a certain age using B-splines [3]. The model minimizing AIC has been ultimately selected. The first knot was chosen on a grid between 35 and 60 years (which were close to the 25^th^ and the 75^th^ percentile of the age distribution), whereas a levelling-off was obtained by adding a second knot at the late age of 70 (which was close to the 90^th^ percentile of the age distribution).

SDS values were obtained for each measurement Y by subtracting the mean (which was age-dependent, noted MEAN(age)) and by dividing by the standard deviation SD provided by the fit (including the variations due to families and centers, as well as the residual variance), i.e. SDS=(Y-MEAN(age))/SD. These SDS values were expected to be approximately normally distributed. This was checked using a goodness of fit procedure, where we compared the number of observations falling below, between and above the 2.5^th,^ 10^th^, 25^th^, 50^th^, 75^th^, 90^th^ and 97.5^th^ percentile, assuming normality of SDS, with the theoretical values of a standardized normal distribution. Significant results would thus suggest that normality is not perfect. Note that while statistical models have been fitted without the outliers, goodness of fit procedures did include the outliers.

Let Zq the q^th^ percentile of a standardized normal distribution. The q^th^ percentile Sq for a diagnostic ratio R on the original variable at a given age can then be calculated as: Sq=sign(p)*(MEAN(age)+SD*Zq)^(1/p). If the selected power transformation is p=0, one calculates instead: Sq=exp(MEAN(age)+SD*Zq).

All calculations have been run using the free statistical R software, version 3.5.0 (R Foundation for Statistical Computing [4]), where linear mixed models have been fitted using the lmer function to be found in the lme4 library. B-splines have been implemented using the splines library.

**Results**

In our first descriptive analysis, we found significant differences between men and women for 59 out of the 65 diagnostic ratios of urinary steroid hormone metabolites using a non-parametric Mann-Whitney test. Even in case of a non-significant difference, the relationship to age might differ which motivated us to model the diagnostic ratios separately for men and for women, as discussed in the statistical methods section. We also compared diagnostic ratios during the day and during the night using a signed-rank Wilcoxon test and we found significant differences for 60 out of 65 diagnostic ratios in women and for 55 out of 64 diagnostic ratios in men.

The power transformation to correct for skewness was selected between -0.4 and +0.5 for all but one diagnostic ratios and both sex, with most of them (113/130) between -0.2 and +0.2, i.e. close to a log-transformation. Only for one diagnostic ratio no transformation was necessary.

Out of the 46’974 available observations, only 53 were detected as outliers and removed from subsequent analysis (apart from goodness of fit procedures).

A model without any age effect was selected for only 7 out of the 130 ratios of steroids (3 for men and 4 for women). Among the remaining 123 models, a linear age effect was selected in 40 cases, a quadratic effect in 21 cases, a quadratic spline in 32 cases and a quadratic spline with levelling-off in 30 cases, illustrating the variety of the possible relationships between the ratios of steroids and age.

Among the variance, which was not explained by the factor age, the variance due to family ranged (depending on the diagnostic ratios) between 7% and 44% (averaging 26%) for men, and between 6% and 53% (averaging 27%) for women. The variance due to the center ranged between 0% and 10% (averaging 1%) for men, and between 0% and 12% (averaging 1%) for women, the residual variance accounting on average for 73% (men) and for 71% (women) of the variability not due to age.

The goodness of fit statistics run at the 5% significance level did not reveal a contradiction between the model and the data for 121/130=93.% of our models, which was in turn not significantly different from the expected 95%. Thus, our models were flexible enough to provide a good approximation of the reality in most cases.

**References**

1. Ackermann D, Groessl M, Pruijm M, Ponte B, Escher G, d'Uscio CH, et al. Reference intervals for the urinary steroid metabolome: The impact of sex, age, day and night time on human adult steroidogenesis. PloS one. 2019;14(3):e0214549. doi: 10.1371/journal.pone.0214549. PubMed PMID: 30925175; PubMed Central PMCID: PMC6440635.

2. Box GEP, Cox DR. An Analysis of Transformations. Journal of the Royal Statistical Society: Series B (Methodological). 1964;26(2):211-43. doi: <https://doi.org/10.1111/j.2517-6161.1964.tb00553.x>.

3. Rousson V. Monotone fitting for developmental variables. Journal of Applied Statistics. 2008;35(6):659-70. doi: 10.1080/02664760801920960.

4. R Development Core Team. R: a language and environment for statistical computing. Available at: [www.R-project.org/](http://www.R-project.org/). Accessed 02 July, 2019.
